# Isofunctional Protein Subfamily Detection Using Data Integration and Spectral Clustering

**DOI:** 10.1371/journal.pcbi.1005001

**Published:** 2016-06-27

**Authors:** Elisa Boari de Lima, Wagner Meira, Raquel Cardoso de Melo-Minardi

**Affiliations:** 1 Department of Biochemistry and Immunology, Universidade Federal de Minas Gerais, Belo Horizonte, Minas Gerais, Brazil; 2 Department of Computer Science, Universidade Federal de Minas Gerais, Belo Horizonte, Minas Gerais, Brazil; Wake Forest University, UNITED STATES

## Abstract

As increasingly more genomes are sequenced, the vast majority of proteins may only be annotated computationally, given experimental investigation is extremely costly. This highlights the need for computational methods to determine protein functions quickly and reliably. We believe dividing a protein family into subtypes which share specific functions uncommon to the whole family reduces the function annotation problem’s complexity. Hence, this work’s purpose is to detect isofunctional subfamilies inside a family of unknown function, while identifying differentiating residues. Similarity between protein pairs according to various properties is interpreted as functional similarity evidence. Data are integrated using genetic programming and provided to a spectral clustering algorithm, which creates clusters of similar proteins. The proposed framework was applied to well-known protein families and to a family of unknown function, then compared to ASMC. Results showed our fully automated technique obtained better clusters than ASMC for two families, besides equivalent results for other two, including one whose clusters were manually defined. Clusters produced by our framework showed great correspondence with the known subfamilies, besides being more contrasting than those produced by ASMC. Additionally, for the families whose specificity determining positions are known, such residues were among those our technique considered most important to differentiate a given group. When run with the crotonase and enolase SFLD superfamilies, the results showed great agreement with this gold-standard. Best results consistently involved multiple data types, thus confirming our hypothesis that similarities according to different knowledge domains may be used as functional similarity evidence. Our main contributions are the proposed strategy for selecting and integrating data types, along with the ability to work with noisy and incomplete data; domain knowledge usage for detecting subfamilies in a family with different specificities, thus reducing the complexity of the experimental function characterization problem; and the identification of residues responsible for specificity.

## Author Summary

The knowledge of protein functions is central for understanding life at a molecular level and has huge biochemical and pharmaceutical implications. However, despite best research efforts, a substantial and ever-increasing number of proteins predicted by genome sequencing projects still lack functional annotations. Computational methods are required to determine protein functions quickly and reliably since experimental investigation is difficult and costly. Considering literature shows combining various types of information is crucial for functionally annotating proteins, such methods must be able to integrate data from different sources which may be scattered, non-standardized, incomplete, and noisy. Many protein families are composed of proteins with different folds and functions. In such cases, the division into subtypes which share specific functions uncommon to the family as a whole may lead to important information about the function and structure of a related protein of unknown function, as well as about the functional diversification acquired by the family during evolution. This work’s purpose is to automatically detect isofunctional subfamilies in a protein family of unknown function, as well as identify residues responsible for differentiation. We integrate data and then provide it to a clustering algorithm, which creates clusters of similar proteins we found correspond to same-specificity subfamilies.

## Introduction

Despite the best research efforts, a substantial and ever-increasing amount of predicted proteins still lack functional annotation [1]. Indeed, the unprecedented increase in the number of new protein sequences being produced by genomics and proteomics projects, as well as the copious amounts of structures for proteins of unknown functions being solved by structural genomics, directly highlight the need for computational methods to determine, quickly and accurately, the molecular and cellular functions of such proteins, given that experimental investigation is difficult, costly, and time-consuming [2, 3]. As the number of sequenced genomes rapidly increases, the vast majority of gene products may only be annotated computationally [4]. However, no high-throughput approaches currently exist capable of revealing the function of every hypothetical gene in the already sequenced genomes. This goal can only be reached per the efforts of several experimental, structural, and computational biologists [5]. The work presented herein is a computational effort aiming to take a step toward that goal.

The commonest protein function annotation approach is homology-based annotation transfer, which assumes proteins sufficiently alike in sequence and structure perform similar functions [3]. Such methods have various limitations due to this assumption [6]. On account of protein function plasticity and of the intrinsic imprecision in related databases, various aspects of function cannot be accurately transferred between similar sequences indiscriminately [7]. In fact, homology-based annotation transfer methods are considered one of the main sources of annotation errors due to an excessively liberal application of function inheritance [3], which fails when similar proteins cannot be identified or when they, too, lack reliable annotations [8, 9]. Moreover, such methods also fail for proteins that have the same function despite being different in sequence and structure (i.e., convergent evolution) [10], and also for those which are sequentially and/or structurally similar yet functionally diverged during evolution [9].

Automatic protein function annotation methods depend on a correlation between functional and sequential or structural similarity measures [11], the simplest of which explores global sequence similarity. Other measures commonly employed in the literature are local sequence motifs, global and local structural similarities, and 3D templates. Since such similarity measures focus on different protein features, one may expect they yield better functional annotations when combined [11]. In fact, literature shows using a single data type (e.g., sequence similarity) is insufficient to precisely annotate protein functions due to the immense amount of factors involved in determining a function, and to the consequent complexity of the automatic annotation problem [4, 7–10, 12–20]. A combined approach is usually more powerful than its individual components [3], so blending various data types is crucial in order to transfer annotations more reliably [21]. This attests to the great importance and need for automatic function analysis methods capable of integrating various data types.

Increasing the difficulty, one ought to consider attributing a function to a protein family is further complicated by the fact that many families are composed of proteins with multiple folds and/or functions. In such cases, determining possible subfamilies may lead to important information about a related protein’s function and structure, as well as about the functional diversification acquired by the family during evolution [7]. Therefore, a family of homologous proteins may be divided into subtypes which share specific functions uncommon to the family as a whole [22]. We believe determining such subfamilies to be a first step toward reducing the protein function annotation problem’s complexity. Hence, this work’s purpose is the detection of isofunctional subfamilies in a protein family of unknown function, along with the identification of residues responsible for subfamily differentiation.

Various methods have been proposed to identify amino acid conservation patterns which distinguish subgroups in a protein family [22–29]. In general, such methods have the considerable disadvantage that subfamilies must be known *a priori*. Apart from the scarcity of experimental information about subfamilies, this requirement is prohibitive when working with protein families of unknown function. To the best of our knowledge, a single method in the literature is similar to ours in that it first attempts to identify subfamilies in a Pfam [30] family through clustering and, then, to detect specificity determining residues which characterize them: Active Sites Modeling and Clustering (ASMC) [31], which clusters proteins according exclusively to active site composition. Simply put, given a Pfam family, ASMC first performs homology-based structural modeling of its members with a reference structure, later superposing such models to the structure in order to identify residues aligned to its active site. As a result, it builds a multiple sequence alignment (MSA) that represents the active site composition for each protein in the family. This MSA is then subjected to a hierarchical clustering, generating a tree whose nodes are protein groups and whose levels represent successive subdivisions of the family: the root of the tree has all proteins in the same group, whereas the leaves represent singleton clusters. Afterward, the authors manually cut this tree in order to obtain clusters which they find most interesting. The reported number of clusters in the family is, thus, manually defined. Finally, the authors perform a statistical significance analysis to determine the active site positions which were most important to differentiate among groups.

Considering the various challenges to automatic function annotation may be extended to the problem of detecting subfamilies, in order to overcome the previously mentioned major obstacles faced by homology-based methods, we adopt an approach that integrates various data types. For this purpose, the similarity between protein pairs according to different knowledge domains is interpreted as evidence, albeit weak, of functional similarity. We integrate such data types using genetic programming and, afterward, provide it as input to a spectral clustering algorithm

Our main goal is to propose a strategy for selecting and combining pieces of functional similarity evidence between protein pairs, and to analyze the manner in which integrating information from different knowledge domains is capable of directing a clustering process to detect, in a protein family of unknown function, isofunctional subfamilies, along with the residues that differentiate them. This goal was successfully achieved. The proposed framework’s capability of using diverse data types, even if incomplete or uncertain, is of remarkable importance for application scenarios such as this, since data from biological experiments are naturally imprecise, mainly due to the dynamic nature of the phenomena investigated as well as to experiment interpretation errors [4], and certain types of information are relatively scarce. Additionally, protein function is determined by various factors, and the complementarity of the different data sources allows for the algorithm to work with as much information as possible.

Our main contributions are the proposed strategy for selecting and integrating various data types, along with the ability to work with noisy and incomplete data; the possibility of using domain knowledge for detecting isofunctional subfamilies in a protein family with different specificities or even of unknown function, thus reducing the complexity of the experimental function characterization problem; and the identification of residues responsible for specificity.

## Methods

The proposed framework consists of five main steps, namely definition of the protein set to be studied, collection of pieces of functional similarity evidence, data integration, clustering, and quality evaluation.

### Protein family definition

Once a Pfam family of interest is defined, a filtering process is applied to obtain the protein set to be studied. First, we collect the family’s full sequence alignment from Pfam and extract the UniProt identifiers, together with the subsequences which contain the domain that characterizes the family. The protein set is later filtered by subsequence size, eliminating those whose lengths differ more than a standard deviation from the family average, as done by ASMC [31]. Afterward, we collect the structures associated to the family from PDB, separate the chains, and select the reference structures to be used as templates for structurally modeling the family sequences using Modeller [32], and also to search for pockets that are possible active sites using Fpocket [33]. We prioritize structures obtained by X-ray crystallography, with high resolution and which contain ligands. Next, the protein set is further filtered according to similarity with the reference structures, eliminating those with less than 30% identity to all structures, which is the minimum level accepted by Modeller for creating a structural model. For each sequence, we choose the model with the smallest energy, as done in [1] and [31]. By the end of this filtering process, the database contains the UniProt identifiers, amino acid subsequences containing the family domain, and structural models for all remaining proteins.

### Similarity evidence collection

The steps taken to collect and apply the various data types interpreted as evidence of functional similarity are described next. Any data that may be used to compare proteins pairs can be added to the process. Given the data integration method (i.e., genetic programming) is capable of learning which data types contribute to achieving good clusterings and filter out those of little use, even data types unlikely to be related to functional similarity were included.

#### Sequence-based data

Global and local pairwise sequence alignments are performed, respectively, applying the Needleman-Wunsch [34] and Smith-Waterman [35] algorithms implemented in R’s Biostrings package [36]. Both alignments are performed using amino acid substitution matrix BLOSUM62. The pairwise alignment scores define the global and local sequence similarity matrices.

#### Structure-based data

TM-Align [37] is used to perform pairwise structural alignments, from which three similarity matrices are derived, containing alignment sizes, identity percentages, and TM-scores. Another structure-based data type interpreted as evidence of functional similarity are the structural signatures produced by CSM [38], which represents a protein’s structure by an array of varying distances between α-carbons. In this work, the smaller the Euclidean distance between a pair of such arrays, the greater the similarity evidence, so this similarity matrix is calculated by transforming the corresponding distance matrix.

#### Genomic context data

For each protein pair, we collect from the STRING database [39] its scores for conserved genome neighborhood, gene fusion events, co-occurrence, and co-expression. Such data are used to identify genes which appear to be under common selective pressures during evolution, and which are therefore thought to be functionally associated [40]. Genomic context-based similarity matrices are generated for each of these four scores. This work used STRING version 9.1.

#### Protein properties

In order to annotate a protein’s function from its structure without using alignments, one needs to consider structural attributes which capture information relevant to functional differentiation [8]. For this reason, we collect from the following software and databases various protein features that may be function-related and, consequently, aid in clustering proteins into subfamilies. Given such properties are protein-specific, the following similarity matrices are calculated by comparing values for each protein pair.

From EMBOSS Pepstats [41], we collect molecular weights, isoelectric points, and molar percentages for each amino acid class (i.e., aliphatic, aromatic, non-polar, polar, charged, basic, and acidic). Corresponding similarity matrices are built from the differences between values for each protein pair. Amino acid composition by itself contains a surprising amount of information relevant to protein function [8], so we also collected the Dayhoff statistics for each amino acid and use these values to build, for each protein, an array that reflects its composition. The composition-based similarity matrix is calculated from the squared Euclidean distances between all pairs of arrays.

From ExPASy ProtParam [42], we collect instability and GRAVY indices, and the corresponding similarity matrices are again built from the differences between values for each protein pair. Since smaller differences and distances reflect stronger similarity evidence between the pair, these similarity matrices are calculated by transforming the corresponding distance matrices.

From InterPro [43], we collect all domains and motifs associated to each protein and consider that the more domains two proteins have in common, the stronger the evidence of functional similarity. The domain-based similarity matrix is then calculated by the number of InterPro annotations each pair has in common. Finally, from Gene Ontology [44], we collect all terms associated to each protein and create the GO-based similarity matrix by comparing the number of common terms between each protein pair.

#### Putative active sites

**Fig 1:**
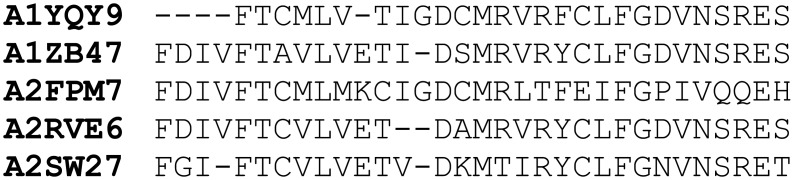
Example of MSA for a given reference structure’s pocket.

The active site is ASMC’s [31] main element, since its composing amino acids are the attributes used to describe the proteins to the clustering algorithm it employs. In order to compare the proposed framework to ASMC, the active site is included in this work and obtained by the same process as in [31]. Given a reference structure for the studied protein family, Fpocket [33] is used to detect its surface pockets. Subsequently, the structural models for the family’s proteins are superposed to the reference structure using MultiProt [45] in order to extract, for each protein, the residues which aligned with those belonging to the structure’s pockets. Positions for which no correspondence exists in the model are marked with a gap (-). For each detected pocket, a multiple sequence alignment (MSA) is generated containing its composition for each protein in the family, as illustrated in Fig 1.

Once the reference structures’ pockets are determined, one needs to be chosen as the putative active site for use in clustering. In [31], ASMC was run with all pockets, and results were presented for the most conserved one in each family, which the authors later found to be the correct active sites. In this work, we select the pocket which is presumably the active site by analyzing residue conservation in the family, since functionally important residues tend to be conserved. The algorithm for selecting such a pocket is simple: if there exist cavities with three or more residues which are conserved in at least 50% of the family, we choose the one with the largest Fpocket-calculated score. We use the minimum of three conserved residues based on the observation that each enzyme has an average of 3.5 catalytic residues [46].

After a pocket is chosen as the putative active site, the corresponding MSA is used to compare its composition for each protein pair in the family. The more similar the active site compositions are, the stronger we consider the evidence of functional similarity to be. Two active site-based similarity matrices are generated, using the identity and the BLOSUM62 score of the putative active sites for each protein pair. Besides being a source of functional similarity evidence, active site compositions are also used to create conservation patterns for the clusters, as done in ASMC, as well as to evaluate the quality of the resulting clusters, as will be described later on.

#### Resulting database

A database is built containing, for each protein pair, all similarity values according to the aforementioned data types. Table 1 shows the columns in the database, their respective data sources, and the identifiers for the corresponding similarity matrices. Such identifiers will be used to show the data combinations produced by the genetic programming system.

**Table 1:** Data sources and corresponding identifiers for the protein similarity matrices employed in this work.

Data Source	Name	Description
**Pfam/UniProt**	*uniprotA*	UniProt ID for protein A
	*uniprotB*	UniProt ID for protein B
**Needleman-Wunsch**	*seqAliG*	Global sequence alignment score
**Smith-Waterman**	*seqAliL*	Local sequence alignment score
**TM-Align**	*strAliSize*	Structural alignment size
	*strAliId*	Structural alignment identity percentage
	*strAliScr*	Structural alignment TM-score
**CSM**	*csmDist*	Structural signature array distances
**STRING**	*neighborhood*	Conserved gene neighborhood score
	*fusion*	Gene fusion score
	*cooccurrence*	Co-occurrence score
	*coexpression*	Co-expression score
**EMBOSS Pepstats**	*difMolWeight*	Difference in molecular weights
	*difIsoPoint*	Difference in isoelectric points
	*difAliphRes*	Difference in aliphatic residue contents
	*difAromRes*	Difference in aromatic residue contents
	*difPolarRes*	Difference in polar residue contents
	*difChargedRes*	Difference in charged residue contents
	*difBasicRes*	Difference in basic residue contents
	*difAcidicRes*	Difference in acid residue contents
	*aaCompDist*	Amino acid composition array distance
**ExPASy ProtParam**	*difInstab*	Difference in instability indices
	*difGRAVY*	Difference in GRAVY indices
**InterPro**	*interpro*	Number of common annotations
**Gene Ontology**	*go*	Number of common terms
**Putative active site**	*ASid*	Putative active site identity percentage
	*ASscr*	Putative active site BLOSUM62 score

### Data integration

Each column in the database corresponds to one of the aforementioned similarity matrices, each of which is normalized to [0, 1], or to [-1, 1] in case negative values exist. The data types for which smaller values indicate greater similarity, as is the case for those involving differences or distances, have their intervals reversed. Hence, all matrices may be interpreted in the same way: the higher the value, the larger the similarity between that protein pair according to the corresponding data type. In order to combine such primary similarity matrices into a single matrix to be provided as input to the clustering algorithm, we use genetic programming (GP).

GP is a natural computing technique that automatically solves problems without the user having to know or specify the form of the solution. Basically, in each generation, a population of individuals, each of which represents a combination of data types in this work, is stochastically transformed into a presumably better population [47]. The execution ends when a maximum number of generations is reached or when some other stopping criterion is met. Such transformations are accomplished by genetic operators of crossover, reproduction, and mutation, which recombine parts of individuals from one population to create individuals for the next [48]. Crossover works by randomly selecting parts of two individuals and switching them. For mutation, a random part of a single individual is replaced by new code, whereas in reproduction, an individual is selected and copied into the next generation [47]. Genetic operators are usually mutually exclusive, and their probability of application is called the operator rate. Individuals are selected to undergo such operations according to their fitness value. Thus, fitter individuals are more likely to be selected to “breed”, producing new individuals for the next generation.

This work’s GP system was implemented using the lil-gp library [48] in C. Starting from random data combinations, it learns, over generations, which ones yield better protein clusters. Each primary matrix is depicted by a variable, so individuals represent equations that combine different matrices through addition. For each individual in the population, the GP system calculates the final similarity matrix by applying its equation to each protein pair, and subsequently runs the spectral clustering algorithm with the calculated matrix, returning the quality of the yielded clustering as the individual’s fitness value. By evolving a population of equations that combine the various data sources, apart from the actual clusters generated, results will allow to check which types of information are most useful to discriminate among groups in a protein family.

Eq 1 shows an example of individual which calculates the similarity *s_ij_* between each protein pair *(i, j)* by adding the number of InterPro annotations they have in common, their conserved neighborhood score in STRING, and three times the TM-score of their structural alignments.

S=interpro+neighborhood+3strAliScr (1)

### Clustering

Clustering is a data mining technique which consists in dividing a set of objects into natural clusters, each of which represents a significant subpopulation, so that objects in the same group are very similar to each other, while different from those in other clusters [49]. In this work, we consider partitional clustering methods, in which none of *K* clusters are empty and each object belongs to a single cluster [50]. Among the various algorithms presented in the literature, we opted for employing spectral clustering, since it is capable of solving very complex problems such as the case that, when plotted, the objects from each cluster are positioned in intertwined spirals, which cannot be separated by something simple as a line or a curve. Such an algorithm was necessary for our application scenario since families of homologous proteins can rarely be separated into subfamilies easily.

Spectral clustering uses the eigenvectors and eigenvalues of the similarity matrix to reduce the number of dimensions before performing clustering in the reduced space. First, a similarity graph is built from the inputted similarity matrix. Next, its Laplacian matrix is calculated, along with its eigenvectors and eigenvalues. The eigenvectors corresponding to the *K* smallest eigenvalues are taken, each as a dimension in the new data set representation. This change in representation from the original space to a *K*-dimensional space accentuates the cluster properties in the data, so that clusters may be trivially detected in the new representation [51], so much so that a simple clustering algorithm like K-Means may be used.

In this work, given the similarity matrix calculated by a GP system individual, we define the adjacency matrix of the totally connected similarity graph, and calculate its normalized asymmetric Laplacian matrix, its eigenvalues and eigenvectors, taking the *K* first eigenvectors. This new *N*×*K* matrix, where *N* is the number of proteins in the family and *K* is the desired number of clusters, is then provided as input to the K-Means clustering algorithm.

### Experiment evaluation

Given our goal of detecting isofunctional subfamilies in a protein family, and considering each cluster is described by an active site composition-based profile, a quality measure which numerically reflects the differences among cluster profiles is required. We consider pointwise mutual information (PMI) [52], which measures the amount of information the occurrence of a specific value *x* contributes to making the correct classification of an object relative to cluster *y* [53]. The PMI is a measure of how much the event co-occurrence probability (*p(x, y)*) differs from expected based on the individual event probabilities and on the independence assumption (*p(x)p(y)*), and is calculated by Eq 2 [54]. If there exists a genuine association between the values, then *p*(*x*, *y*) ≫ *p*(*x*)*p*(*y*) and, consequently, *PMI*(*x*, *y*) ≫ 0. If no interesting relationship exists, then *p*(*x*, *y*) ≈ *p*(*x*)*p*(*y*) and *PMI*(*x*, *y*) ≈ 0. Finally, if *x* and *y* are in complementary distributions, then *p*(*x*, *y*) ≪ *p*(*x*)*p*(*y*), hence *PMI*(*x*, *y*) ≪ 0.

$$PMI\left(x,y\right)=ln\frac{p(x,y)}{p(x)p(y)}$$ (2)

PMI is “pointwise” because it is calculated for two values *x* and *y*, whereas mutual information (MI) is calculated for two variables *X* and *Y*, and corresponds to the expected PMI over all possible values, i.e., *MI*(*X*, *Y*) = ∑_*x*_∑_*y*_ *p*(*x*, *y*)*PMI*(*x*, *y*) [54]. MI measures the information dependence or overlap between two random variables, reaching maximum value when the variables are perfectly correlated [54, 55].

We consider a cluster to be interesting when it has (almost) exclusive residues for specific active site positions. Hence, we compare each cluster to the union of the others. For each position *p_i_*, residue *r_k_*’s importance for cluster *c_j_* is measured by *PMI*_*p*_*i*__(*c*_*j*_, *r*_*k*_), whereas its importance in the union of the remaining clusters ($\bar{c_{j}}$) is calculated by $PMI_{p_{i}}\left(\bar{c_{j}},r_{k}\right)$. This yields *MI*_*p*_*i*__(*c*_*j*_, *r*_*k*_), calculated by Eq 3, in which the *p*_*p*_*i*__(*c*_*j*_, *r*_*k*_) and $p_{p_{i}}\left(\bar{c_{j}},r_{k}\right)$ probabilities are estimated by residue *r_k_*’s frequency in cluster *c_j_* and in the other clusters at position *p_i_*. Because *PMI*_*p*_*i*__(*c*_*j*_, *r*_*k*_) and $PMI_{p_{i}}\left(\bar{c_{j}},r_{k}\right)$ values have opposite signs, and considering that we only deem important to a cluster those residues more frequent in it than in the other clusters, only residues for which *PMI*_*p*_*i*__(*c*_*j*_, *r*_*k*_) > 0 are considered. In case the residue also occurs in other clusters, then $PMI_{p_{i}}\left(\bar{c_{j}},r_{k}\right)<0$, and their addition will reduce *r_k_*’s importance for cluster *c_j_*. Finally, if *PMI*_*p*_*i*__(*c*_*j*_, *r*_*k*_) ≤ 0, we consider *MI*_*p*_*i*__(*c*_*j*_, *r*_*k*_) = 0.

$$MI_{p_{i}}\left(c_{j},r_{k}\right)=p_{p_{i}}\left(c_{j},r_{k}\right)PMI_{p_{i}}\left(c_{j},r_{k}\right)+p_{p_{i}}\left(\bar{c_{j}},r_{k}\right)PMI_{p_{i}}\left(\bar{c_{j}},r_{k}\right)$$ (3)

For a given cluster, there might be multiple residues in a specific active site position. Hence, considering *f_k_* as residue *r_k_*’s frequency in cluster *c_j_*, we have that *MI*_*p*_*i*__(*c*_*j*_) = ∑_*k*_ *f*_*k*_ *MI*_*p*_*i*__(*c*_*j*_, *r*_*k*_). Gaps are not considered in this calculation. Finally, the quality measure for the clustering as a whole is the overall average, calculated by Eq 4, where *P* is the total number of positions, and *C* is the number of clusters. The GP system uses this as fitness function. Thus, it attempts to maximize the mutual information between the active site residues and the clusters, which is equivalent to searching for clusters that present characteristic active site compositions.

$$MI=\frac{1}{P}\frac{1}{C}\sum_{i}\sum_{j}MI_{p_{i}}\left(c_{j}\right)$$ (4)

When a ground-truth exists such as the SFLD family classification, external validation measures may be used to calculate a clustering’s agreement with it. Pairwise measures consider the cluster labels and ground-truth classifications over all pairs of objects. For an object pair with the same ground-truth classification, the objects may be attributed to a same (true positive—TP) or different (false negative—FN) clusters. Analogously, a pair with different ground-truth classifications, may be assigned to a same (false positive—FP) or different (true negative—TN) clusters.

The precision (P) for a given clustering is the percentage of object pairs that are in a same cluster and actually have the same ground-truth classification ($P=\frac{TP}{TP+FP}$), while the recall (R) is the fraction of pairs with the same ground-truth classification that were assigned to a same cluster ($R=\frac{TP}{TP+FN}$). The F_1_ score tries to balance the precision and recall values by computing their harmonic mean, and is calculated as $F_{1}=2\times \frac{P\times R}{P+R}$. The Rand index measures the fraction of true positives and negatives over all object pairs, and is defined as $Rand=\frac{TP+TN}{TP+FP+FN+TN}$. It is symmetric in terms of true positives and negatives, and measures the fraction of pairs where the clustering and the ground-truth classification agree. The Rand index has a value between 0 and 1, with 0 indicating complete disagreement and 1 indicating the clusters are exactly the same as the ground-truth classification. The Jaccard coefficient measures the fraction of true positives when ignoring the true negatives. It is defined as $Jaccard=\frac{TP}{TP+FP+FN}$. Since it ignores true negatives, it is asymmetric in terms of the true positives and negatives. Thus, it emphasizes the similarity in terms of the object pairs that belong together in both the clustering and the ground-truth, but discounts the pairs that do not belong together [49]. The larger the values for these measures, the better the agreement of the clustering with the ground-truth classification. Additionally, the variation of information measures the amount of information not shared between the clustering and the ground-truth, and is calculated as *VI* = *H*(*S*) + *H*(*S*′) − 2*I*(*S*, *S*′), where H is the entropy of a data partition, and I is the mutual information between two partitions of the same data. Lastly, the edit distance is defined as the minimum number of split or merge operations required to transform the clustering into the ground-truth classification, where a split or merge affecting multiple objects is considered one operation. The edit distance between the ground-truth classification and a clustering, with class *k* and cluster *k’*, respectively, is calculated as *Edit* = 2(∑*r*_*k*,*k*′_) − *K* − *K*′, where *r_k,k’_* equals 1 if class *k* and cluster *k’* have items in common, and zero otherwise. *K* is the number of classes, while *K’* is the number of clusters [64]. The smaller the values for the variation of information and the edit distance, the more similar the clustering is to the ground-truth classification.

## Results

Since clustering is independent from supervision data such as class labels, the proposed framework may be applied to any protein family, even those of unknown function, as shown in this work. However, in order to evaluate our technique’s performance and to facilitate its comparison with similar literature method ASMC [31], we applied our technique to the same well-known families studied by its authors: nucleotidyl cyclases (Pfam family PF00211), serine proteases (PF00089), and protein kinases (PF00069 and PF07714). The same protein sets and subfamily labels were used, except for the removal of proteins which had since become obsolete in UniProt. We observed ASMC is unstable in terms of the clusters it produces, since this minor update to the protein sets caused the algorithm to yield clusters extremely different from those presented in [31] using the same parameter values. Given the purpose of detecting isofunctional subfamilies in protein families of unknown function, a fourth case study was performed on Pfam family DUF849, to which ASMC has also been applied in [1]. For comparison purposes, we employed the same structural models as in [1] and [31], which were obtained using the template structures listed in Table 2 along with their catalytic residues according to the Catalytic Site Atlas (CSA) [56].

**Table 2:** Structures used as templates for modeling the family sequences.

Family	Subfamily	Structure	CSA Residues
**Nucleotidyl cyclases**	Adenylate cyclases	1AB8:A	R1029
	Guanylate cyclases	3ET6:A	-
**Protein kinases**	Ser/Thr kinases	2CPK:E	D166, K168, E170, N171, T201
	Tyr kinases	1U46:A	D252, A254, R256, N257, V292
**Serine proteases**	Chymotrypsins	1AB9:(A, B, C, D)	H57, D102, G193, S195, G196
	Elastases	1EST:A	H57, D102, G193, S195, G196
	Trypsins	5PTP:A	H57, D102, G193, S195, G196, S214
**DUF849**	-	2Y7F:A, 3FA5:A, 3CHV:A, 3E49:A, 3E02:A, 3LOT:A, 3C6C:A	-

Structures are presented in format PDB code:chain (e.g., 1AB8:A indicates chain A of PDB structure 1AB8). Residues are presented in format residuePosition (e.g., R1029 represents an Arg residue in position 1029 of the corresponding structure).

In order to evaluate our technique’s performance against a gold standard, case studies were also performed on the crotonase and enolase superfamilies of the Structure Function Linkage Database (SFLD) [57], which hierarchically divides superfamilies into subgroups and families. The structural templates used for modeling the sequences and their respective catalytic residues according to the CSA are presented in Table 3.

**Table 3:** Structures used as templates for modeling the SFLD superfamily sequences.

Superfamily	Subgroup	Structure	CSA Residues
**Crotonases**	crotonase-like	1MJ3:A	A98, S118, H122, G141, E164, G172
**Enolases**	enolase	7ENL:A	E168, E211, K345, K396
	glucarate dehydratase	1ECQ:A	K205, K207, N237, H339
	mandelate racemase	1MDR:A	K166, D270, H297, E317
	mannonate dehydratase	3QKE:A	-
	methylaspartate ammonia-lyase	1KKR:A	-
	muconate cycloisomerase	3DG6:A	-

Structures are presented in format PDB code:chain (e.g., 1AB8:A indicates chain A of PDB structure 1AB8). Residues are presented in format residuePosition (e.g., R1029 represents an Arg residue in position 1029 of the corresponding structure).

The following subsections show results for these six case studies using the experiment configurations described in the S1 Text. The protein sets studied for each family are presented in the S2 Text. The MI values for the best results found in each of five runs of the experiments are presented in the S3 Text.

It is noticeable in both [31] and [1] that ASMC is usually employed to provide an initial hierarchical clustering of the protein family, which afterward is manually altered in order to obtain clusters the authors consider most interesting. This manipulation allows for different hierarchy levels to be considered for each tree branch, thus distorting the algorithm’s output. However, in order to compare ASMC to the proposed technique, for protein families nucleotidyl cyclases, serine proteases, and protein kinases, ASMC’s clustering step was rerun with the updated protein sets. For all case studies except for the DUF849 family, the trees produced by ASMC were cut at the first two levels, thus defining the number of clusters in a per-level basis. For the DUF849 family, our results were compared to the seven groups manually produced in [1] by the manipulation of ASMC’s output.

As previously discussed, the quality of the resulting clusters, used as fitness function by the GP system, is measured by the MI value, calculated by Eq 4. The larger the value, the better the clustering. Table 4 summarizes the differences among MI values for the clusterings produced by our framework and by ASMC for the same numbers of clusters. The primary similarity matrices, which are combined by the GP system to produce the final matrices provided as input to the spectral clustering algorithm, are denoted by their identifiers previously listed in Table 1. The logos which represent each cluster’s active site composition profile were generated by WebLogo [58]. Their color scheme represents amino acid chemical features: green for polar residues, purple for neutral, blue for basic, red for acidic, and black for hydrophobic. Each column in the logo corresponds to a position in the putative active site, and narrower columns denote the occurrence of gaps. A residue’s height in the logo is proportional to its frequency in the corresponding cluster.

**Table 4:** Comparison of Mutual Information (MI) values for the clusterings obtained by each technique for the studied protein families.

Family	Clusters	GP System	ASMC
**Nucleotidyl cyclases**	3	22.35	22.16
	6	16.13	14.11
**Protein kinases**	3	102.94	67.46
	7	50.70	45.99
**Serine proteases**	4	17.71	16.58
	11	12.09	10.59
**DUF849**	7	36.51	*14.05

* This value refers to the seven clusters defined in [1] by manipulating ASMC’s output.

### Case study I: Nucleotidyl cyclases

Nucleotidyl cyclases are a family of cytosolic or membrane-attached domains that catalyze the transformation of a nucleotide triphosphate into a cyclic nucleotide monophosphate [25]. These proteins have fundamental roles in a wide range of cellular processes, and two functional subfamilies exist, namely adenylate cyclases, which act on ATP to form cAMP, and guanylate cyclases, which catalyze the conversion of GTP to cGMP [59]. Mutations of only two residues (Glu-Lys and Cys-Asp) are sufficient to completely alter the specificity from GTP to ATP [25].

After removing, from the original set, 75 proteins that became obsolete in UniProt, 461 remained in this family, of which 186 are labeled as adenylate cyclases and 275, as guanylate cyclases, according to the labels employed in [31]. Thus, the GP system was run to divide this family into two clusters. However, with the same parameter values used in [31], ASMC produced, for the updated protein set, a hierarchical clustering whose first level divided the family into three clusters, and whose second level divided it into six. Hence, in order to compare results, the GP system was also run with three and six clusters.

Table 5 presents the data combinations produced by the GP system which yielded the best results for the nucleotidyl cyclases in five runs for each considered number of clusters. Since the MI is based on active site composition, it was expected that the related similarity matrices would be involved in the best results. Other data types which stood out were the global and local sequence alignments scores (*seqAliG* and *seqAliL*), the structural alignment identities (*strAliId*), and the differences in aliphatic residue content (*difAliphRes*). One may observe a large amount of data types was required by the GP system to partition the family into two clusters.

**Table 5:** Data combinations which yielded the best results for the nucleotidyl cyclases in five runs of the GP system.

Clusters	Run	Equation
2	1	4*ASid* + *ASscr* + *cooccurrence* + 4*csmDist* + *difAcidicRes* + 3*difAliphRes* + *difAromRes* + *difBasicRes* + *difChargedRes* + 2*difInstab* + *difIsoPoint* + 2*difPolarRes* + *interpro* + *neighborhood* + 3*seqAliG* + 2*strAliId* + 2*strAliSize*
	2	*ASid* + 3*ASscr* + *aaCompDist* + 2*coexpression* + 2*cooccurrence* + *csmDist* + 2*difAcidicRes* + *difAliphRes* + *difBasicRes* + *difChargedRes* + 2*difGRAVY* + 3*difInstab* + 2*difIsoPoint* + 2*difMolWeight* + 2*go* + *interpro* + 5*seqAliG* + *strAliId* + *strAliScr*
	3	*ASid* + 2*ASscr* + *aaCompDist* + *cooccurrence* + *difAcidicRes* + *difAliphRes* + *difAromRes* + *difBasicRes* + *difChargedRes* + *difIsoPoint* + *difMolWeight* + *difPolarRes* + 2*seqAliL* + *strAliId* + 4*strAliScr*
	4	4*ASid* + 4*ASscr* + 6*coexpression* + *cooccurrence* + 5*csmDist* + *difAcidicRes* + 4*difAliphRes* + 3*difAromRes* + *difBasicRes* + 4*difChargedRes* + *difGRAVY* + 3*difInstab* + 2*difIsoPoint* + *difMolWeight* + 5*difPolarRes* + 2*go* + 2*interpro* + 4*neighborhood* + *seqAliG* + 2*seqAliL* + 2*strAliId* + 3*strAliScr* + 3*strAliSize*
3	1	*ASscr* + *difMolWeight* + *seqAliG*
6	3	5*ASid* + *difAliphRes* + 2*go* + *seqAliG* + 3*seqAliL* + 2*strAliId*

#### Dividing the nucleotidyl cyclases into two clusters

**Fig 2:**
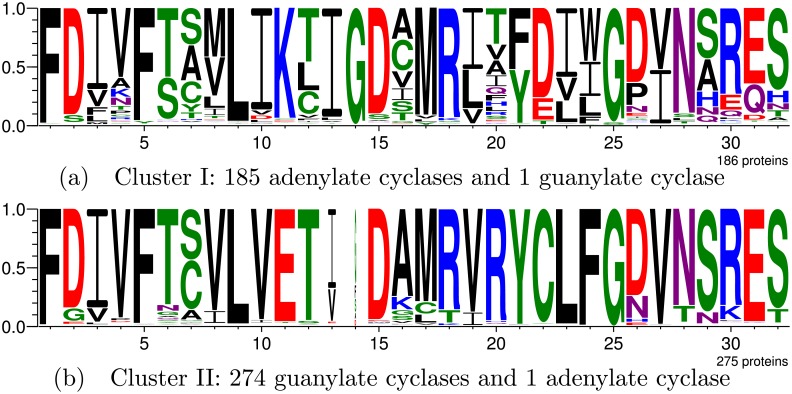
Nucleotidyl cyclase division into two clusters by the GP system. Subfigure (a) shows the active site logo for the adenylate cyclase cluster, while (b) shows that for the guanylate cyclase cluster.

When the GP system was run to divide the family into two clusters, as is the number of subfamilies, the four equations presented in Table 5 yielded the same results, whose logos and compositions in terms of subfamily labels are presented in Fig 2. Such clusters are nearly identical to the subfamily labels, except for two discrepancies, the first of which was of guanylate cyclase-labeled protein Q5UFR4, inserted into the adenylate cyclase cluster. Its unreviewed UniProt entry shows it has been annotated with GO terms *adenylate cyclase activity* and *cAMP biosynthetic process*, which suggest the GP system correctly inserted it into the adenylate cyclase cluster, and that the subfamily label adopted in [31] is inaccurate. The second divergence was for adenylate cyclase-labeled protein Q7RKA2, inserted into the guanylate cyclase cluster. Its also unreviewed UniProt entry lacks any subfamily-specific annotations. The only subfamily-related information is its submitted name of *Guanylyl cyclase enzyme-related*, which is a weak annotation, yet suggests this label may also be inaccurate and that, again, the GP system may have correctly clustered the protein.

We consider a group to be interesting if it contains, in specific active site positions, residues which are (almost) exclusive to the proteins in it. Ideally, such positions will correspond to known Specificity Determining Positions (SDPs). The partial MI values for each residue, each position, and each cluster, which compose the overall MI, enable the numerical evaluation of what residues and respective positions most distinguish a given cluster. The most important residues to discriminate between the two clusters generated by the GP system for the nucleotidyl cyclases are listed in Table 6, in which K11_523_, for example, indicates a Lys residue in active site position 11, which corresponds to position 523 in chain A of PDB structure 3ET6.

**Table 6:** Most important residues for the two nucleotidyl cyclase clusters produced by the GP system.

Cluster	Residues
**I**	**K11_523_**, I10_522_, G14_526_, **D22_592_**, I23_593_, F21_591_, I13_525_, W24_594_
**II**	**C22_592_**, **E11_523_**, F24_594_, V10_522_, R20_590_, L23_593_, V19_584_

Listed in decreasing order of partial MI value. Residues in bold correspond to known SDPs. Subscripted positions correspond to those in PDB structure 3ET6:A.

As previously mentioned, the mutation of two residues is sufficient to alter the specificity from guanylate to adenylate cyclase [25, 31]. Considering chain A of PDB structure 3ET6, such mutations are the substitution of the Glu523 and Cys592 in guanylate cyclases, for Lys and Asp in adenylate cyclases, respectively. Such positions in the structure correspond to active site positions 11 and 22. In fact, as listed in Table 6, K11_523_ and D22_592_ are among the residues considered most important to differentiate Cluster I (adenylate cyclases) according to partial MI values, while E11 and C22 were the two most important residues to distinguish Cluster II (guanylate cyclases). Other residues known to be conserved in each subfamily are, for positions in structure 3ET6, Arg590, Leu593, and Phe594 in guanylate cyclases, substituted by Gly, Ile, and Trp in adenylate cyclases [25, 31]. Such positions correspond to active site positions 20, 23, and 24. One may observe that, except for G20_590_ for Cluster I, all others mentioned are among those considered by the GP system most important to differentiate among the clusters. This shows the proposed framework was able to create clusters whose most distinguishing positions correspond to those which knowingly define subfamily specificities among nucleotidyl cyclases.

#### Further dividing the nucleotidyl cyclases

ASMC was unable to separate the two subfamilies. The first level of its hierarchical clustering divides the family into three clusters and, as shown in the S4 Text, instead of clusters related to the existing subfamilies, it prioritized adenylate cyclase subgroups, while the bulk of the family was put into the same cluster. When run with three clusters for comparison purposes, the proposed framework finds one of the adenylate subgroups found by ASMC, yet the subfamily division is maintained in the other two clusters, as shown in the S4 Text. The subfamilies were only separated by ASMC in the second level of its hierarchical clustering, whose six resulting clusters are presented in the S5 Text, along with a comparison to the proposed framework’s results.

When dividing the family into two clusters, the GP system placed adenylate cyclase-labeled protein Q7RKA2 along with the guanylate cyclases. However, when run with three clusters, this protein was placed in one of the adenylate cyclase clusters. This shows an advantage of the partitional clustering used in our framework relative to the hierarchical clustering employed by ASMC: once a node in the hierarchy is divided, a protein cannot move to a different tree branch. Therefore, in case a cluster is split erroneously during the process, the error will be propagated throughout the hierarchy. Meanwhile, for partitional clustering, proteins may migrate to a cluster that becomes more suitable as the number of clusters increases, which is equivalent to changing tree branches to repair a mistake.

#### Summary

This case study showed the proposed framework obtained clusters in better agreement with the family’s division into its two subfamilies than those produced by ASMC. Furthermore, results showed that, when there are more clusters than subfamilies, ASMC tends to subdivide already relatively uniform clusters, while our GP system produces more contrasting clusters. The proposed framework’s greater success in this case study suggests it is more suitable for finding isofunctional subfamilies in a protein family than ASMC.

### Case study II: DUF849

This Pfam family, defined by the presence of a conserved domain of unknown function, was studied in [1] because it contains the Kce protein, which was of interest to the authors for they had previously discovered an initial association between it and a formerly orphan enzyme activity. Such activity is involved in the lysine fermentation pathway and catalyzes the condensation of β-keto-5-amino-hexanoate (KAH) and acetyl-CoA to produce aminobutyryl-CoA and acetoacetate. Since the DUF849 proteins do not all come from organisms capable of fermenting lysine, this suggests there are various biochemical reactions catalyzed by different family members [1]. Hence, the authors considered DUF849 a good case study for discovering new activities in a protein family of unknown function, and named the family “BKACE”, which stands for β-keto acid cleavage enzyme.

**Fig 3:**
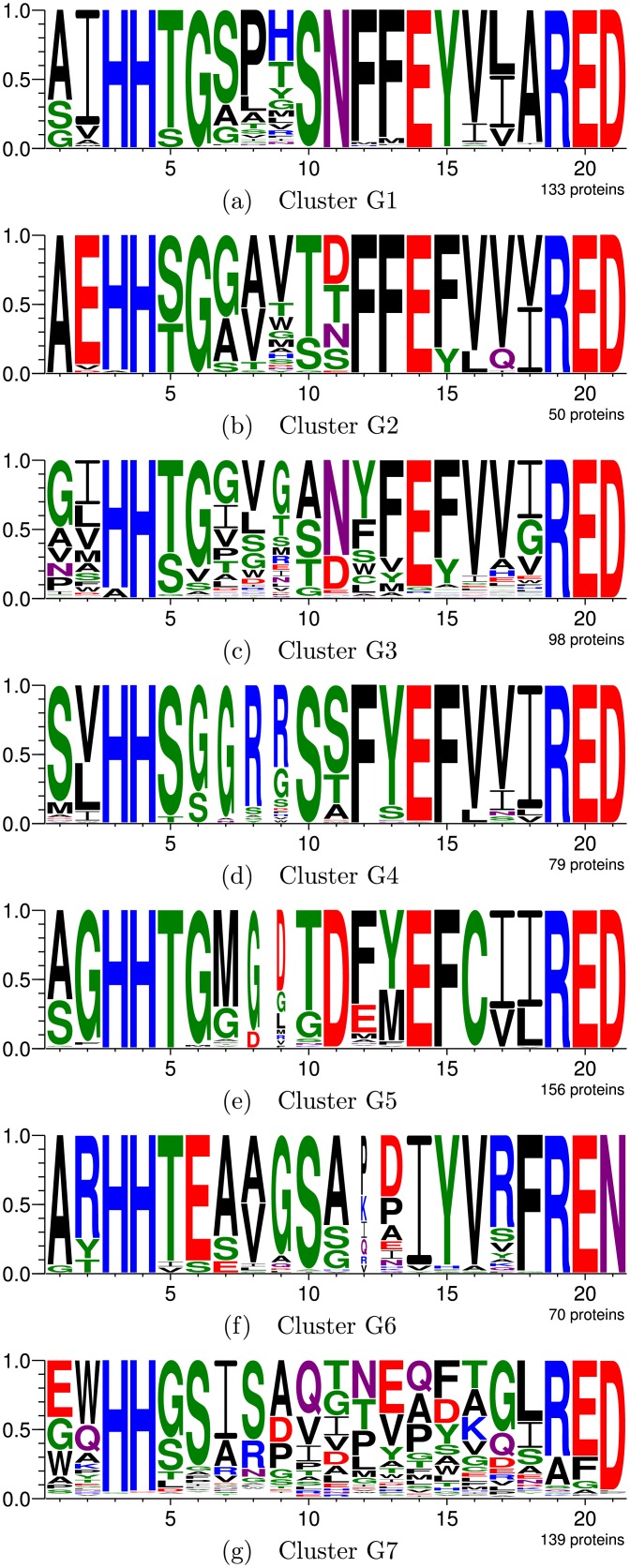
DUF849 division into seven clusters produced by manually altering ASMC’s hierarchical clustering in [1]. Subfigures (a) through (g) show the active site logos for clusters G1 through G7, respectively.

The main result presented in [1] is a division of the set of 725 proteins into seven groups, obtained by manually altering ASMC’s hierarchical clustering. This manipulation allows to consider different hierarchy levels for each tree branch, thus distorting the algorithm’s output to build clusters the authors consider most interesting. Group logos are presented in Fig 3. According to the authors, G3 presents five subgroups, and there is high correlation among the distribution of the proteins in the seven clusters and the nature of the compounds they transform, as presented in Table 7.

**Table 7:** Substrate nature in each group.

Group	Substrates
**G1**	hydrophobic and non-charged polar
**G2**	KAH
**G3**	β-ketoadipate
	benzoylacetate and β-ketohexanoate
	hydrophobic and polar
	mixed BKACE
	not BKACE
**G4**	negatively charged
**G5**	positively charged
**G6**	not BKACE, presenting decarboxylation activity
**G7**	not BKACE

Enzymatic activity distribution in these groups, however, is not as clear as depicted, since there are proteins which showed activity for substrates related to other clusters. Considering the substrates for which activities were tested in [1], S1 Table shows the number of times an activity was detected for each group, considering two repetitions for each test. The distribution, among the manually produced clusters, of the number of enzymes considered active for each substrate, shows the complexity of clustering this family into isofunctional subfamilies due to the promiscuity it presents.

**Fig 4:**
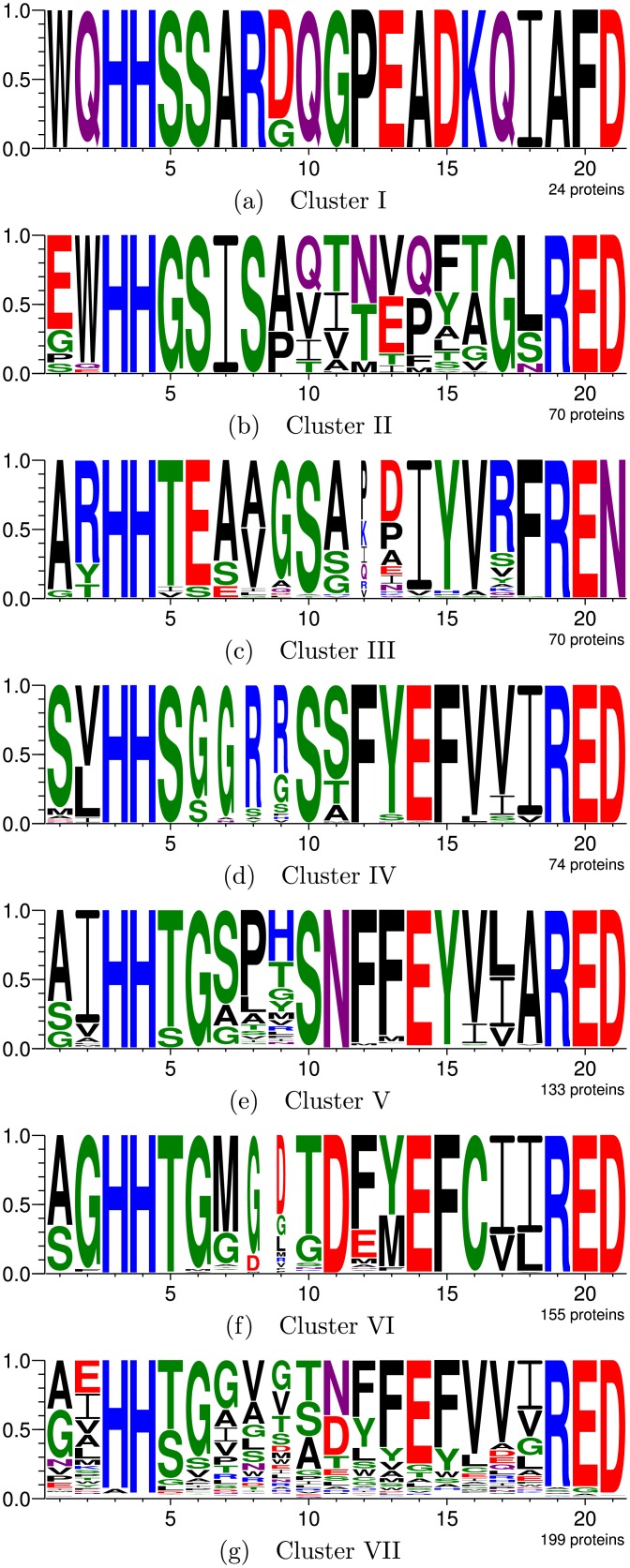
DUF849 division into seven clusters by the GP system. Subfigures (a) through (g) show the active site logos for clusters I through VII, respectively.

The manually defined groups have MI = 14.05. For comparison, the GP system was run to divide the DUF849 family into seven clusters. The best result has MI = 36.51 and uses equation 2*ASid* + *csmDist* + 2*neighborhood* + 2*seqAliL* + *strAliId*. Cluster logos are presented in Fig 4, while the residues that most distinguish each cluster are listed in S2 Table. Because this is a protein family of unknown function, result comparison is complicated. However, there is substantial correspondence between the two clusterings:

- Clusters I and II obtained by the GP system contain 24 and 70 proteins, respectively, and are related to G7 defined in [1], which contains 139 proteins. None of such clusters have proteins active for any of the tested substrates.
- Cluster III is exactly the same as G6, with seventy proteins, sixteen cases of β-ketoglutarate activity, and one 4-hydroxybenzoylacetate activity.
- Cluster IV, with 74 proteins, corresponds to G4, which contains 79 proteins. Both have exactly the same activity cases.
- Cluster V is related to G1. Both have 133 proteins and activity cases for sixteen substrates. However, Cluster V has three active cases for 4-hydroxybenzoylacetate, while G1 has two.
- Cluster VI, with 155 proteins, corresponds to G5, with 156 proteins. Both have the exact same activity cases.
- Cluster VII, containing 199 proteins, is related to G2 and G3, which contain, respectively, 50 and 98 proteins. In fact, one of ASMC’s hierarchical clustering branches was reportedly divided manually in [1] to produce these two clusters. The difference in activities is one 4-hydroxybenzoylacetate activity case in G2 which is not present in Cluster VII, since the corresponding enzyme was inserted into Cluster V along with other enzymes active for this substrate.

Despite being completely automatic, our framework was still able to obtain clusters very similar to those manually produced in [1], even better concentrating the enzymes active for 4-hydroxybenzoylacetate. The only problem in this case study was the GP system considered it to be more relevant, in terms of active site composition, to break group G7 into two relatively uniform clusters, while the authors in [1] opted not to divide this heterogeneous group since the corresponding enzymes are inactive for all tested substrates. Thus, we have successfully demonstrated the proposed framework’s ability and utility for detecting isofunctional subfamilies in families of unknown function.

### Case study III: Protein kinases

Protein kinases are enzymes that modify the functions of other proteins by adding phosphate groups usually removed from ATP, covalently binding them to the side chains of Ser, Thr, or Tyr residues [25]. They are one of the largest and most functionally diverse protein families, responsible for controlling the majority of biochemical pathways, performing key roles in regulating metabolic processes, cell differentiation, and proliferation of diverse cell types [60]. The main division in protein kinases is between Ser/Thr and Tyr kinases: Ser and Thr are similar in size and shape, while the reaction chemistry and substrate size are substantially different for Tyr [25]. The majority of kinases act upon Ser or Thr, while others are specific to Tyr, and some act upon all three. It is known that some positions confer specificity, such as subdomain VI, in which consensus sequence RDLKPEN is usually found in Ser/Thr kinases, while RDLAARN is typical of Tyr kinases [25].

After removing from the protein set used in [31] 314 proteins that became obsolete in UniProt, 3,087 remained in this family, of which 2,044 are labeled as Ser/Thr kinases and 1,043 as Tyr kinases, according to the labels employed in [31], in which a subgroup of 235 Tyr kinases labeled as Epidermal Growth Factor Receptors (EGFRs) was also reported. Using the same parameter values applied in [31] for the original protein set, ASMC produced, for the updated set, a hierarchical clustering with three and seven clusters in its first two levels. Given two main subfamilies exist, our framework was applied to divide the family into two, three, and seven clusters.

Table 8 presents the data combinations produced by the GP system which yielded the best results for protein kinases. Yet again, the presence of active site-related data is noticeable as expected due to the quality measure employed. Other outstanding data types were structural alignment identities (*strAliId*) and GO term similarities (*go*).

**Table 8:** Data combinations which yielded the best results for the protein kinases in five runs of the GP system.

Clusters	Run	Equation
2	1	*ASid* + *go* + *interpro*
3	2, 4	*ASid* + *strAliId*
7	3	*coexpression* + 2*go* + 2*strAliId* + *strAliSize*

#### Dividing the protein kinases into two clusters

**Fig 5:**
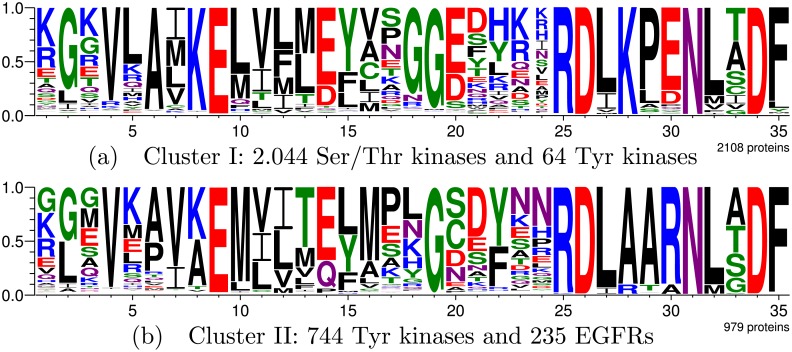
Protein kinase division into two clusters by the GP system. Subfigure (a) shows the active site logo for the cluster consisting mainly of Ser/Thr kinases, while (b) shows the logo for the cluster of Tyr kinases combined with the EGFR subcluster.

When the GP system is run to divide this family into two clusters, the best result involves three data types (Table 8). Fig 5 shows the cluster logos and compositions. One may observe the aforementioned consensus sequences RDLKPEN for Ser/Thr kinases and RDLAARN for Tyr kinases are clearly present. Table 9 lists the residues which most distinguish each cluster.

**Table 9:** Most important residues for the two protein kinase clusters produced by the GP system.

Cluster	Residues
**I**	**K28_254_**, **E30_256_**, **P29_255_**, G18_210_, E20_212_, L10_181_
**II**	**A28_254_**, **R30_256_**, **A29_255_**, M16_208_, M10_181_, V7_157_, A8_158_

Listed in decreasing order of partial MI value. Residues in bold correspond to known SDPs. Subscripted positions correspond to those in PDB structure 1U46:A.

Considering positions in chain A of PDB structure 1U46, it is known that the residues involved in substrate specificity differentiation in protein kinases are the substitution of Ala254, Ala255, and Arg256 in Tyr kinases, by Lys, Pro, and Glu in Ser/Thr kinases, respectively [25, 31]. Such positions correspond respectively to active site positions 28, 29, and 30. These are exactly the residues considered by the GP system as most important to differentiate each cluster due to the higher partial MI values. This shows our framework was able to create clusters whose most distinguishing residues correspond to those that knowingly define the subfamily specificities.

None of the 64 Tyr kinase-labeled proteins inserted into the Ser/Thr kinase cluster have been manually reviewed. However, existing annotations such as InterPro domains IPR008271 (*serine/threonine-protein kinase, active site*) and IPR002290 (*Ser/Thr/dual specificity protein kinase, catalytic domain*), as well as GO term *protein serine/threonine kinase activity*, suggest that either the subfamily labels used in [31] are inaccurate and the GP system clustered them correctly along with the Ser/Thr kinases, or these proteins may have dual specificity.

#### Further dividing the protein kinases

In the first level of ASMC’s hierarchical clustering, the protein kinases were divided into three clusters, presented in the S6 Text. The structural models for some of the family’s proteins did not align well with the reference structure’s active site, which yielded numerous gaps for positions uninvolved in the consensus sequences. ASMC created a cluster containing such proteins. However, gaps are not function-related, so such a clustering is uninformative. Additionally, despite creating an EGFR cluster, the two main subfamilies were mixed, and remained so even when considering the hierarchical clustering’s second level, whose seven clusters are shown in the S7 Text, along with a comparison to the proposed framework’s results.

When the GP system was run with three clusters for comparison purposes, all EGFR-labeled proteins, which are a subset of the Tyr kinases, were inserted into a same uniform cluster, and the other clusters comply almost completely with the subfamily labels, along with the consensus sequences typical of each subfamily, as shown in the S6 Text. Despite errors for 0.45% of the proteins, our framework was able to create clusters with almost total correspondence with the existing protein kinase subfamilies.

### Case study IV: Serine proteases

Proteases are a large enzyme family involved in peptide bond hydrolysis. Almost a third of all proteases are serine proteases, whose name derives from the Ser residue at the active site [61]. Serine proteases are involved in a huge number of biological processes, such as digestion, homeostasis, apoptosis, signal transduction, reproduction, immune response, and blood coagulation [61, 62]. They present a catalytic triad composed of a Ser, an Asp, and a His [31], whose 3D arrangement allows for moving protons in and out of the active site. All serine proteases act through a similar catalytic mechanism, but have different cleavage preferences due to active site changes [25, 31]. For chymotrypsins, the active site is lined with hydrophobic residues, so proteins containing hydrophobic residues such as Leu or Ile form strong bonds in the correct orientation for the triad to act. The cavity in trypsins contains a negatively charged Asp, so their substrates must have a specifically positioned positively charged residue such as Lys or Arg. In turn, elastases have smaller cavities, so only proteins containing short-chained residues such as Gly or Ala can be acted upon [62].

After removing 140 sequences that became obsolete in UniProt since being used in [31], 1,533 proteins remained, of which 43 are labeled as elastases, 26 as chymotrypsins, and 1,464 as trypsins, according to the subfamily labels employed in [31], in which a subgroup of 13 trypsins were found to be kallikreins. When ASMC is run on the updated protein set with the same parameter values used for the original set, the family is not divided. Hence, the main parameter (-C 0.25) was reduced in 0.05 decrements until a value which divided the family was found: 0.15, which yielded a hierarchical clustering with four clusters in its first level, and eleven in its second.

For comparison purposes, the proposed framework was run to divide the family into four and eleven clusters. Table 10 shows the data combinations that yielded the best results for the serine proteases in five runs for each considered number of clusters. Again, the active site-based similarity matrices showed strong utility, as expected. The other data type that stood out was the global sequence alignment score (*seqAliG*).

**Table 10:** Data combinations which yielded the best results for the serine proteases in five runs of the GP system.

Clusters	Run	Equation
4	1	*ASid* + *ASscr* + *seqAliG*
11	1	2*ASid* + *ASscr* + *seqAliG*

#### Dividing the serine proteases into four clusters

The first level of ASMC’s hierarchical clustering divided the family into four clusters, whose logos and compositions are presented in the S8 Text, along with a comparison of the proposed framework’s results. Both techniques were only able to separate the elastases, while chymotrypsins and kallikreins, which represent even smaller percentages of the family, were mixed in one of the three trypsin clusters. Due to the disparity in the amount of trypsins relative to the other subfamilies, and since their identification is hampered by the considerable variability among trypsins, a larger number of clusters is required by both techniques in order to isolate the small subfamilies in specific clusters.

#### Further dividing the serine proteases

The second level of the hierarchical clustering produced by ASMC has eleven clusters, whose logos and compositions are presented in the S9 Text. ASMC was able to separate chymotrypsins into their own cluster, and almost isolated the kallikrein-labeled proteins in another. However, the elastases, which composed a relatively uniform cluster, were broken into two. This shows ASMC divides a cluster even if it is already consistent relative to the others, which yields subgroups with virtually no difference.

Cluster logos and compositions for the best result obtained by the proposed framework for eleven clusters are presented in the S9 Text. The GP system was unable to isolate the smaller subfamilies in their own clusters. However, it is noticeable from the logos that the clusters are more significant than those produced by ASMC in terms of having larger differences among them, whereas ASMC subdivided relatively uniform clusters. In fact, the analysis of the UniProt entries for the proteins in each cluster revealed interesting points. In Cluster I, for example, there are 27 trypsin-labeled proteins; seven have been manually reviewed, six of which are annotated as prothrombins, and the other as coagulation factor VII, whose alternative name is *serum prothrombin conversion accelerator*. Among the twenty unreviewed entries, ten were named prothrombins, seven, thrombins, and one, coagulation factor VII. The remaining two proteins have names unrelated to these, but are annotated with InterPro domains IPR003966 (*Prothrombin/thrombin*) and IPR018992 (*Thrombin light chain*).

Among the 34 trypsin-labeled proteins inserted into Cluster IV along with the thirteen kallikreins, twenty have been manually reviewed, all of which are annotated, in fact, as kallikreins. Thirteen of the unreviewed proteins have suggested names related to kallikreins, such as *Prostatic kallikrein 2*, *Glandular kallikrein* and simply *Kallikrein*. The last one lacks any kallikrein-related annotations, yet corresponds to gene Klk1b1, the same gene that codes various manually annotated kallikreins. This shows the subfamily labels used in [31] are inconsistent and that the GP system was able to correctly group the family’s kallikreins in a same cluster, which ASMC was unable to do.

Although the proposed framework was unable to isolate chymotrypsins, it does so successfully when the family is divided into an extra group, totalizing twelve clusters. The best result, obtained using equation 3*ASid* + 2*ASscr* + *go*, maintains a single elastase cluster, along with the aforementioned kallikrein and prothrombin clusters, and is able to isolate the chymotrypsins in their own cluster, as shown in the S10 Text.

#### Summary

In this case study, the serine proteases proved to be difficult to separate into the known subfamilies for both techniques, due to the immense imbalance between them. This is due to the substantial residue variability among the trypsins, which represent 94.7% of the family, causing trypsin subgroups to be more easily found than the smaller subfamilies, simply because the first are larger. For this reason, both techniques were only able to find kallikrein and chymotrypsin-specific clusters after dividing the trypsins into various subgroups.

### Case study V: Crotonases

The crotonase superfamily enzymes catalyze a wide range of metabolic reactions. Some have been shown to display dehalogenase, hydratase, and isomerase activities, while others have been implicated in carbon-carbon bond formation and cleavage, as well as the hydrolysis of thioesters [63].

After applying the filtering process described in the “Protein family definition” subsection to the 7,908 crotonase superfamily proteins with known families in the SFLD [57], 2,694 proteins remained, distributed among twelve families, all of which are in the crotonase like subgroup. The superfamily distribution is presented in Table 11.

**Table 11:** Distribution of the crotonases among families.

Family	Amount
enoyl-CoA hydratase	1,507
methylglutaconyl-CoA hydratase	269
1,4-dihydroxy-2-napthoyl-CoA synthase	217
delta(3,5)-delta(2,4)-dienoyl-CoA isomerase	201
1,2-epoxyphenylacetyl-CoA isomerase	143
dodecenoyl-CoA delta-isomerase (mitochondrial)	87
dodecenoyl-CoA delta-isomerase (peroxisomal)	65
diffusible signal factor (DSF) synthase	55
crotonobetainyl-CoA hydratase	47
polyketide biosynthesis enoyl-CoA hydratase	40
feruloyl-CoA hydratase/lyase	33
methylmalonyl-CoA decarboxylase	30

The active site compositions were extracted from structurally aligning the models against reference structure 1MJ3’s active site. Given twelve families exist, the proposed framework was applied to divide the family into twelve clusters. The best result is obtained with equation *ASid* + *seqAliG* + *strAliId*, which yielded a clustering with MI = 52.18. Cluster logos and compositions in terms of SFLD family labels are presented in the S11 Text. The distribution of crotonase families among clusters is presented in Table 12.

**Table 12:** Distribution of families among the twelve crotonase superfamily clusters produced by the GP system.

Cluster	Size	Family	Amount
**I**	29	methylmalonyl-CoA decarboxylase	29/30
**II**	55	diffusible signal factor (DSF) synthase	55/55
**III**	58	dodecenoyl-CoA delta-isomerase (peroxisomal)	58/65
**IV**	68	polyketide biosynthesis enoyl-CoA hydratase	35/40
		feruloyl-CoA hydratase/lyase	33/33
**V**	84	dodecenoyl-CoA delta-isomerase (mitochondrial)	84/87
**VI**	178	1,2-epoxyphenylacetyl-CoA isomerase	143/143
		enoyl-CoA hydratase	34/1,507
		dodecenoyl-CoA delta-isomerase (peroxisomal)	1/65
**VII**	201	delta(3,5)-delta(2,4)-dienoyl-CoA isomerase	201/201
**VIII**	217	1,4-dihydroxy-2-napthoyl-CoA synthase	217/217
**IX**	253	methylglutaconyl-CoA hydratase 2	252/269
		polyketide biosynthesis enoyl-CoA hydratase	1/40
**X**	286	enoyl-CoA hydratase	227/1,507
		crotonobetainyl-CoA hydratase	47/47
		polyketide biosynthesis enoyl-CoA hydratase	4/40
		dodecenoyl-CoA delta-isomerase (peroxisomal)	3/65
		methylglutaconyl-CoA hydratase 2	3/269
		dodecenoyl-CoA delta-isomerase (mitochondrial)	1/87
		methylmalonyl-CoA decarboxylase	1/30
**XI**	404	enoyl-CoA hydratase	404/1,507
**XII**	861	enoyl-CoA hydratase	842/1,507
		methylglutaconyl-CoA hydratase 2	14/269
		dodecenoyl-CoA delta-isomerase (peroxisomal)	3/65
		dodecenoyl-CoA delta-isomerase (mitochondrial)	2/87

In comparison with SFLD’s family classification, the clustering produced by the GP system for dividing the crotonase superfamily into twelve clusters presents a Rand index of 0.80, a Jaccard coefficient of 0.44, 93.84% precision, 45.06% recall, an F_1_ score of 0.61, a variation of information of 0.80, and an edit distance of 26. These values indicate the clustering produced by the GP system is in high agreement with the SFLD family classification, yet this agreement is more related to precision (i.e., pairs that are in the same cluster and actually have the same classification) than to recall (i.e., pairs with the same classification that are actually in the same cluster). Given the enoyl-CoA hydratase, which accounts for 55.94% of the crotonase superfamily, had elements assigned to four different clusters, this greatly impacted the recall. Hence, the results suggest more clusters are required to properly separate the families, due to the existing variation among enoyl-CoA hydratases.

When the SCI-PHY classification method was applied to the crotonase superfamily in [64], the authors achieved a variation of information of 1.05, and an edit distance of 32. Although different protein sets were considered for each technique, this suggests the proposed framework outperforms SCI-PHY. Unfortunately, we were unable to properly compare the techniques on a same data set because the studied protein set is not presented in [64].

### Case study VI: Enolases

Enolase superfamily enzymes catalyze the abstraction of the α-proton of a carboxylic acid to form an enolic intermediate. This is mediated by conserved active site residues. Reactions catalyzed by these enzymes include racemization, β-elimination of water and of ammonia, and cycloisomerization. These enzymes have two structural domains: a N-terminal capping domain and a C-terminal TIM beta/alpha-barrel domain, both of which are required for function [65].

After applying the filtering process described in the “Protein family definition” subsection to the 31,182 enolase superfamily proteins with known families in the SFLD [57], 4,791 proteins remained, distributed among six subgroups and twelve families. The superfamily distribution is presented in Table 13.

**Table 13:** Distribution of the enolases among subgroups and families.

Subgroup	Family	Amount
enolase	enolase	2,492
mandelate racemase	D-galactonate dehydratase	474
	rhamnonate dehydratase	224
	L-fuconate dehydratase	183
	D-tartrate dehydratase	98
	L-talarate/galactarate dehydratase	98
muconate cycloisomerase	dipeptide epimerase	448
	o-succinylbenzoate synthase	370
	N-succinylamino acid racemase 2	70
glucarate dehydratase	glucarate dehydratase	193
mannonate dehydratase	mannonate dehydratase	84
methylaspartate ammonia-lyase	methylaspartate ammonia-lyase	57

The active site compositions were extracted from structurally aligning the sequence models against reference structure 1MDR’s active site, chosen according to the process described in the Methods section. Given twelve families exist, the proposed framework was applied to divide the family into twelve clusters. The best result is obtained with equation 3*APid* + *go* + 2*seqAliG*, which yielded a clustering with MI = 98.18. Cluster logos and compositions in terms of SFLD family labels are presented in the S12 Text. The distribution of enolase families among clusters is presented in Table 14.

**Table 14:** Distribution of families among the twelve enolase superfamily clusters produced by the GP system.

Cluster	Size	Family	Amount
**I**	80	mannonate dehydratase	80/84
**II**	87	rhamnonate dehydratase	87/224
**III**	92	D-tartrate dehydratase	92/98
**IV**	94	L-talarate/galactarate dehydratase	94/98
**V**	123	rhamnonate dehydratase	123/224
**VI**	140	o-succinylbenzoate synthase	140/370
**VII**	165	L-fuconate dehydratase	165/183
**VIII**	177	glucarate dehydratase	177/193
**IX**	443	dipeptide epimerase	363/448
		N-succinylamino acid racemase 2	61/70
		o-succinylbenzoate synthase	19/370
**X**	456	D-galactonate dehydratase	456/474
**XI**	942	enolase	513/2,492
		o-succinylbenzoate synthase	204/370
		dipeptide epimerase	82/448
		methylaspartate ammonia-lyase	57/57
		L-fuconate dehydratase	18/183
		D-galactonate dehydratase	17/474
		glucarate dehydratase	16/193
		rhamnonate dehydratase	12/224
		N-succinylamino acid racemase 2	9/70
		D-tartrate dehydratase	6/98
		L-talarate/galactarate dehydratase	4/98
		mannonate dehydratase	4/84
**XII**	1992	enolase	1,979/2,492
		o-succinylbenzoate synthase	7/370
		dipeptide epimerase	3/448
		rhamnonate dehydratase	2/224
		D-galactonate dehydratase	1/474

Although nine of twelve clusters are pure (i.e., contain a single family), the mixture in clusters IX, XI and XII shows that more clusters are required in order to properly separate the families. As was the case with the serine proteases and crotonases, this is likely caused by family imbalance: the enolase family accounts for 52% of the protein set, and variation among the enolases may dominate the smaller families. Interestingly, despite the mixture in Cluster IX, all three families are in the muconate cycloisomerase subgroup of the enolase superfamily. In comparison with SFLD’s family classification, the clustering presents a Rand Index of 0.87, a Jaccard coefficient of 0.62, 87.30% precision, 67.83% recall, an F_1_ measure of 0.76, a variation of information of 0.84, and an edit distance of 34. These values reflect the great agreement of the clustering produced by the GP system with the SFLD family classification. The somewhat low recall, however, further suggests more clusters are required to properly separate the families, likely due to the existing variation among the dominating enolase family.

When applying the SCI-PHY classification method to the enolase superfamily in [64], the authors achieved a variation of information of 1.37, and an edit distance of 70. Although the protein sets are different, this suggests the proposed framework outperforms SCI-PHY. However, experiments on a same dataset would be required to properly compare the techniques. Unfortunately, we were unable to perform such comparison since the studied protein set is not presented in [64].

## Discussion

The case study with nucleotidyl cyclases showed our technique successfully separated the proteins into its two known subfamilies, whereas ASMC prioritized subgroups of adenylate cyclases, while the majority of the family was put in the same cluster. Only in the second hierarchy level did ASMC create a guanylate cyclase-specific cluster, but at that point it had fragmented the adenylate cyclases into five subgroups, even though there wasn’t much variability in this subfamily. Another successful case study was with protein kinases, for which our framework yielded clusters whose correspondence with the known subfamilies was almost complete, despite some mistakes in the clustering of less than 1.5% of the family proteins. Meanwhile, ASMC ended up prioritizing a cluster of proteins containing multiple gaps, which are unrelated to function, and, even after increasing the number of clusters, it was unable to separate the two subfamilies.

The serine protease case study showed the tremendous imbalance between subfamilies to be a challenge for both techniques, since the substantial variability among trypsins, which account for 94.7% of the family, lead the methods to find trypsin subgroups more easily than the small subfamilies. Thus, both techniques were only able to find clusters specific to kallikreins and chymotrypsins after breaking trypsins into several subgroups. This is a data scarcity issue, which we are unable to tackle given we work with complete Pfam families. Nevertheless, the results showed this is not an issue for the MI measure, since the elastase, chymotrypsin, and kallikrein subfamilies could all be considered undersampled in comparison with the trypsin subfamily, and the proposed framework was still able to isolate the elastase subfamily even when considering four clusters, and, eventually, produced chymotrypsin and kallikrein-specific clusters when a larger number of clusters was considered.

The subgroups found by our technique were shown to be relevant, since it found a cluster containing exclusively prothrombins, along with a kallikrein cluster larger than the one found by ASMC. Furthermore, some association exists among the trypsin subclusters and the proteins’ species of origin, although the larger subclusters present mixtures, as shown in the S13 Text. A larger number of clusters is required in order to create subdivisions for specific phylogenetic clades. However, as presented in the S14 Text, the analysis of the Enzyme Commission number distribution among clusters has shown the existence of subclusters for the protein kinase and serine protease families is justified by there actually existing more specific classifications than those reflected by the subfamily labels employed in [31]. Thus, the clusters generated by the proposed framework are in accordance with the existing EC number annotations, although experiments with larger numbers of clusters are required in order to create EC number-specific clusters. However, the literature has shown that the EC system is unsuited for use as a ground truth classification due to the annotation errors caused by automatic annotation transference [66–69].

The case studies with well known protein families showed our technique produces clusters that are in better agreement with their division into subfamilies than those produced by ASMC. Furthermore, when there are more groups than subfamilies, our technique tends to produce clusters which are more different from each other than ASMC, which tends to subdivide clusters that are already relatively uniform.

A fourth case study involved the DUF849 protein family of unknown function, in which case we compared our technique’s results to the groups defined in [1] by manually altering ASMC’s hierarchical clustering. This proved to be a challenging family, due to the observed promiscuity. Still, the clustering produced by our totally automatic technique showed tremendous correspondence with the manually determined groups, which attests to the proposed framework’s utility and capacity for detecting possibly isofunctional subfamilies, even in families of unknown function.

The last two case studies on SFLD’s crotonase and enolase superfamilies were performed in order to analyze the proposed framework’s performance against this gold-standard. For both superfamilies, the GP system was able to create clusterings in great agreement with the ground-truth family classification, seemingly outperforming the SCI-PHY classification method presented in [64]. Unfortunately, a proper comparison of our technique to SCI-PHY was precluded due to the studied protein set not being listed in [64]. As was the case with the serine proteases, the results suggest more clusters are required in order to properly separate the families for both superfamilies, likely due to the existing family imbalance and variation among the dominating families, which impaired a perfect distribution of the twelve families that exist in each superfamily into twelve clusters.

Given our goal of finding isofunctional subfamilies, we consider a cluster to be interesting when it contains residues which are (almost) exclusive to its proteins for the different active site positions. Results showed our mutual information-based cluster quality measure successfully reflects this goal, since using it as the GP system’s fitness function caused contrasting clusters to be found. The better agreement of the clusters produced by our technique with the existing subfamilies was also reflected in the larger MI values. Unfortunately, the manner in which the measure is calculated, which involves partial values for each residue, each position, and each cluster, prevents its use for finding the ideal number of clusters in a protein family, since the value decreases as the number of clusters increases. However, such partial values allow us to numerically evaluate what residues, in which positions, most differentiate a cluster from the others. In fact, for the families whose specificity determining positions are known, such residues were in accordance with those considered by our technique as the most important to distinguish a given cluster.

It is standard practice in the related literature to perform multiple clusterings of a data set with different numbers of clusters and then choose the “optimal” number of clusters as the one with the best value for a given clustering quality measure. Many different such measures have been tested in this work in order to tackle the problem of identifying the ideal number of clusters in a protein family, such as the internal cluster validation measures Silhouette Coefficient, BetaCV, Normalized Cut, Dunn Index, (Pointwise) Mutual Information among clusters, Relative Entropy, Log-likelihood, as well as countless variations of such measures. However, this has proven to be a major challenge. Since we work with Pfam families, every protein has some degree of similarity with all other proteins in the family. So the clustering quality measure tends to be best when (almost) all proteins are put in a single cluster, thus yielding clusterings in which one cluster consists of the bulk of the family, while the others contain very few proteins. Although subfamilies are known to exist, the so far tested quality measures do not reflect this. Thus, despite our best efforts, we have been unable to find a measure that would allow us to compare clusterings with different numbers of clusters in order to determine the ideal number of clusters in a Pfam family. Hence, at this time, we use the MI to compare clusterings with the same number of clusters, and visually and manually inspect the cluster logos and compositions in order to compare clusterings with different numbers of clusters, as done for ASMC in [1] and [31].

Considering the GP system is capable of learning which data combinations produce good clusterings and filter out those of little use, even data types unlikely to be related to functional similarity were included in this work. Since the cluster quality measure is based on the active site, it was expected that data derived from it would be among the combinations that produced the best clusterings. The active site identities (*ASid*) are present in the data combinations for all families, while the scores (*ASscr*) are included for the serine proteases and nucleotidyl cyclases. However, the association of active site data with other data types contributed to improved results. The ones which stood out were the the sequence alignment scores (*seqAliG* and *seqAliL*), present for all families except the serine proteases; the GO term similarities (*go*), present for all but the DUF849 family and crotonase superfamily; the structural alignment identities (*strAliId*), present for all families except for serine proteases and enolases; and the genomic context data (*cooccurrence, coexpression, neighborhood*), present for the nucleotidyl cyclases, protein kinases and the DUF849 family. These data are commonly employed, separately, by homology-based function annotation methods, so their presence among the best data combinations is due to the correspondence, although imperfect, of the similarity according to such data types with the functional similarity.

Interestingly, our GP system was able to find similar clusterings with very different data combinations. This is likely due to the fact that the studied data types are not independent. Their redundancy made it virtually impossible to reach a conclusion about the semantics of the obtained equations. A correlation analysis is presented in the S15 Text, proving the existence of redundancy among some of the data types used as functional similarity evidence. Examples of highly correlated data types are the global and local sequence alignment scores (*seqAliG* and *seqAliL*, 0.96 correlation), the structural alignment scores and sizes (*strAliScr* and *strAliSize*, 0.82 correlation), and the active site identities and scores (*ASid* and *ASscr*, 0.79 correlation). Such correlations were to be expected given the data type pairs originate from the same sources. However, most data types are highly diversified, given they present low correlation to the others, which indicates they add important information to the clustering process. Indeed, the results showed an overall tendency that using more data types leads to better clusters. Since the best results involved the combination of multiple data types, this confirms our initial hypothesis that the similarity between proteins according to different knowledge domains may be interpreted as evidence of functional similarity.

In summary, results showed the proposed framework, which is fully automated, obtained better clusterings than ASMC for nucleotidyl cyclases and protein kinases, in addition to equivalent results for serine proteases and the DUF849 family, whose clustering was defined with manual intervention. In general, the clusters produced by our technique showed considerable correspondence with the known subfamilies and were more relevant than those produced by ASMC, given they show more contrasting differences among each other, whereas ASMC tends to subdivide clusters which are already uniform in comparison to the others. Furthermore, we observed ASMC is unstable in regards to the generated clusters, since the removal of a small number of proteins which became obsolete lead the algorithm to produce extremely different clusters with the same parameter values. Lastly, the crotonase and enolase case studies showed the proposed framework’s ability to create clusters in agreement with a gold-standard classification.

In addition, the hierarchical clustering algorithm employed by ASMC prevents occasional errors during the process from being repaired, since once a node in the hierarchy is subdivided, it is not possible for a protein to switch tree branches. Hence, if a subdivision is erroneously made, the error will be propagated to the following hierarchy levels and will never be fixed. The partitional clustering employed by our technique, on the other hand, allows proteins to migrate to a group that becomes more suitable as the number of clusters increases, which is equivalent to switching branches in ASMC’s hierarchy to repair an error.

Lastly, although structural information, which is very scarce relative to other data such as sequence information, is a central part of the proposed framework, results showed it is applicable even if a structure is only available for one subfamily. In the serine protease case study, for example, kallikrein and prothrombin clusters were found even though the family sequences were not modeled against structures from these subfamilies. Yet, the proposed framework was still able to detect clusters specific to these subfamilies. Additionally, for the crotonase and enolase superfamilies, active site compositions were extracted from structural alignments of the models against reference structures for the entire crotonase-like subgroup and for the mandelate racemase subgroup, respectively. Still, the GP system was able to find family-specific clusters. Thus, only one structure is required in order to apply the proposed framework to a given protein family.

In conclusion, the results presented herein have proven the proposed technique is useful and capable of detecting isofunctional subfamilies in protein families, even for those of unknown function. Hence, it may be widely applied to other protein families for which at least one reference structure is known, as well as altered to include different data types, even if available only for a subset of the studied family, as was the case for the genomic context-based data in this work. This type of framework, which integrates information from diverse and possibly incomplete sources, is of considerable interest for an application scenario such as ours, given that a protein’s molecular function is determined by numerous factors, and that the complementarity of the various data sources allows for the algorithms to work with as much information as possible.

### Future work

A deeper investigation is required into the semantics of the data combinations produced by the Genetic Programming (GP) system. A network of dependent or synonym variables should aid in better comprehending the redundancy among the data types employed as functional similarity evidence. Eliminating redundant data types from the GP system might ease the semantic analysis of the obtained data combinations, as well as improve the quality of the generated clusters. This requires experiments with different subsets of the studied data types. Additionally, we need to investigate the use of phylogenetic information as functional similarity evidence, given that proteins from a same species should subdivide a cluster into different, yet related, functions. This is extremely hampered, however, by data scarcity and the existing imbalance of known proteins among the species of origin.

Considering the difficulty in dividing the serine protease family, as well as the crotonase and enolase superfamilies, due to (sub)family imbalance, it would be interesting to apply sampling methods to families in which this occurs, in order to evaluate the technique’s performance in more well-balanced databases. Furthermore, due to the added complexity for promiscuous protein families, we need to investigate the possibility of adapting the proposed framework to using fuzzy clustering algorithms. Unlike partitional clustering, fuzzy clustering would output, for each protein, a level of membership in each cluster. Thus, a protein which performs multiple functions could belong, at the same time, to different clusters.

Lastly, further efforts are required in the pursuit of a clustering quality measure appropriate for this application scenario that will enable comparing clusterings with different numbers of clusters in order to determine the ideal number of clusters in a protein family.

## Supporting Information

S1 Text Experiment configuration. (PDF)

S2 Text Studied protein sets. (PDF)

S3 Text MI values for the best results produced by the GP system. (PDF)

S4 Text Dividing the nucleotidyl cyclases into three clusters. (PDF)

S5 Text Dividing the nucleotidyl cyclases into six clusters. (PDF)

S6 Text Dividing the protein kinases into three clusters. (PDF)

S7 Text Dividing the protein kinases into seven clusters. (PDF)

S8 Text Dividing the serine proteases into four clusters. (PDF)

S9 Text Dividing the serine proteases into eleven clusters. (PDF)

S10 Text Dividing the serine proteases into twelve clusters. (PDF)

S11 Text Dividing the crotonases into twelve clusters. (PDF)

S12 Text Dividing the enolases into twelve clusters. (PDF)

S13 Text Analysis of the species distribution among clusters. (PDF)

S14 Text Analysis of the EC Number distribution among clusters. (PDF)

S15 Text Analysis of the correlations among the studied data types. (PDF)

S1 Table Enzymatic activity distribution among the seven clusters produced by manually altering ASMC’s hierarchical clustering. (PDF)

S2 Table Most important residues for the seven DUF849 clusters produced by the GP system. (PDF)
