# Supplementary material for: Isofunctional Protein Subfamily Detection Using Data Integration and Spectral Clustering
Source: PLoS Comput Biol. 2016 Jun 27;12(6):e1005001. doi: 10.1371/journal.pcbi.1005001 (PMC4922564; doi:10.1371/journal.pcbi.1005001)
Supplement: S1 Text — (PDF) [file pcbi.1005001.s001.pdf]

Isofunctional Protein Subfamily Detection using Data Integration and Spectral Clustering

Elisa Boari de Lima[1,2,*], Wagner Meira Júnior[2], Raquel Cardoso de Melo-Minardi[2]

1 Department of Biochemistry and Immunology, Federal University of Minas Gerais, Belo Horizonte, MG, Brazil
2 Department of Computer Science, Federal University of Minas Gerais, Belo Horizonte, MG, Brazil

*** eblima@dcc.ufmg.br**

S1 Text: Experiment Configuration

Comparison of similarity graph construction methods

As discussed in the paper, the similarity graph used by the spectral clustering algorithm is built from the similarity matrix calculated by the genetic programming (GP) system. We consider the totally connected graph, whose edges must have non-negative weights. For this reason, we tested two manners of constructing the graph: ignoring negative values in the similarity matrix, or rescaling all its values to [0, 1]. We performed paired observation analysis to determine the best graph construction method for our application scenario.

An analysis of paired observations may be employed to compare two systems when there is a one-to-one correspondence between the ith experiment in System A and the ith experiment in System B [1]. In this work, we need to compare using the similarity graph built from only positive similarity matrix values (System A) with using the similarity graph built from all values (System B). In order to perform such analysis, we run, for each protein family, pairs of experiments with the same parameter values, only varying the graph construction method, thus establishing the required experiment correspondence. Afterward, we calculate the differences in mutual information (MI) values for each pair of experiments, along with the 95% confidence interval for such differences, which indicate that, with 95% probability, the difference in MI values will be in this interval [1]. Hence, if the interval includes zero, there is no statistically significant difference between the qualities of the clusterings yielded by the different graph construction methods, since the difference in MI values may be null. If the interval does not include zero, then it will indicate which construction method yielded significantly better clusters.

The confidence intervals for the differences in MI values of the clusterings obtained by employing each graph construction method are presented in Table S1.1 for each protein family and each amount of clusters tested. Intervals in bold indicate that, with 95% confidence, there are statistically significant differences between the results obtained by the different graph construction methods. Differences are calculated subtracting, from the MI value for the clustering yielded by the graph generated using only positive values (System A), the MI value for the clustering yielded by the graph that considers all values (System B). Thus, positive differences indicate System A yields better results (i.e., clusters with larger MI values), while negative differences indicate the results are better for System B.

Table S1.1. Confidence intervals for the differences in MI values of the clusterings yielded by each similarity graph construction method.

Protein Family	Number of Clusters	Confidence Interval
Nucleotidyl cyclases	2	[0.00, 0.01]
	3	[-0.01, 0.05]
	4	**[1.77, 2.73]**
	5	**[0.14, 0.32]**
	6	[-0.01, 0.05]
DUF849	7	**[0.02, 0.63]**
	9	**[0.11, 0.68]**
	32	**[0.10, 0.23]**
	84	[-0.01, 0.05]
Protein kinases	2	**[0.04, 0.21]**
	3	[-0.19, 0.06]
	4	**[0,66 1.03]**
	5	**[0.06, 0.67]**
	6	**[-0.84, -0.23]**
	7	[-0.28, 0.26]
Serine proteases	3	**[3.04, 3.37]**
	4	[-0.03, 0.89]
	5	**[0.33, 0.95]**
	6	**[0.18, 0.41]**
	7	[-0.04, 0.31]
	8	[-0.09, 0.21]
	9	**[0.25, 0.63]**
	10	[-0.17, 0.23]
	11	**[0.08, 0.31]**
	12	[-0.07, 0.15]
	13	[-0.07, 0.11]

One may observe in Table S1.1 that, for the cases in which the confidence interval does not include zero, i.e., when the graph construction methods lead to significantly different results, the confidence interval always has positive values. This shows that, when a difference exists, the better results are obtained by employing the similarity graph built using only the positive values in the similarity matrix. Hence, this was the graph construction method considered in the paper.

Comparison of Genetic Programming operator rates

GP is robust in practice, so it is likely that various parameter values work [2]. Crossover rates are usually around 90%, whereas mutation rates are typically about 1%. However, due to the limited population size in this work and considering the observation that mutation generally tends to be more successful in smaller populations [3], we considered much larger mutation rates. If the sum of the crossover and mutation rates is less than 100%, reproduction is applied with the complementary rate.

Nine different configurations were tested for the operator rates. For each protein family and each amount of clusters tested, the GP system was run five times for each parameter configuration and each of the similarity graph construction methods. Table S1.2 shows the genetic operator rate configurations employed in this work, as well as the number of times each of them appears among the experiments which produced the largest mutual information (MI) values for each protein families and each amount of clusters, respecting multiplicities in case of ties.

Table S1.2. Tested Genetic Programming operator rates and corresponding number of occurrences among the best results for each protein family and each amount of clusters.

Crossover	Reproduction	Mutation	Occurrences
70%	10%	20%	12
70%	20%	10%	7
80%	5%	15%	15
80%	15%	5%	6
80%	20%	0%	8
85%	5%	10%	6
85%	10%	5%	9
90%	5%	5%	8
90%	10%	0%	11

One may observe in Table S1.2 that the GP system operator rate configuration that most occurred among the top results was using operator rates of 80% for crossover, 5% for reproduction, and 15% for mutation. Hence, for sake of uniformity, the results presented in the paper correspond to those obtained with this operator rate configuration.

References

1. Jain RK. The Art of Computer Systems Performance Analysis: Techniques for Experimental Design, Measurement, Simulation, and Modeling. Wiley; 1991.

2. Poli R, Langdon WB, McPhee NF, Systems E, Sciences M, Koza JR. A Field Guide to Genetic Programming. Freely available at http:/www.gp-field-guide.org.uk; 2008.

3. Luke S, Spector L. A revised comparison of crossover and mutation in genetic programming. In: Koza JR, editor. Proceedings of the Third Annual conference in Genetic Programming; 1998. p. 208–213.
