# Supplementary material for: Isofunctional Protein Subfamily Detection Using Data Integration and Spectral Clustering
Source: PLoS Comput Biol. 2016 Jun 27;12(6):e1005001. doi: 10.1371/journal.pcbi.1005001 (PMC4922564; doi:10.1371/journal.pcbi.1005001)
Supplement: S2 Text — (PDF) [file pcbi.1005001.s002.pdf]

Isofunctional Protein Subfamily Detection using Data Integration and Spectral Clustering

Elisa Boari de Lima[1,2,*], Wagner Meira Júnior[2], Raquel Cardoso de Melo-Minardi[2]

1 Department of Biochemistry and Immunology, Federal University of Minas Gerais, Belo Horizonte, MG, Brazil
2 Department of Computer Science, Federal University of Minas Gerais, Belo Horizonte, MG, Brazil

*** eblima@dcc.ufmg.br**

S2 Text: Studied protein sets

This supplementary text lists the studied protein sets for each family. The proteins are referenced by their UniProt identifiers, and we show their subfamily labels, as well as the clusters to which they were attributed in our experiments, the source organisms, and the Enzyme Commission (EC) numbers they are annotated with according to UniProt.

Case study I: nucleotidyl cyclases

As mentioned in the main text, there are 461 proteins in this family, 186 of which are labeled as adenylate cyclases, and 275 as guanylate cyclases according to the subfamily labels employed in [1]. Table S2.1 lists the protein set, as well as corresponding subfamily labels and clusters they were placed in by the proposed framework when considering two, three and six clusters, the source organisms, and the existing EC number annotations according to UniProt.

Table S2.1. List of proteins in the nucleotidyl cyclase family.

UniProt	Label [1]	Two	Three	Six	Species	EC Number
A2D8T2	adenylate	I	I	I	Trichomonas vaginalis	
A2DBX9	adenylate	I	I	I	Trichomonas vaginalis	
A2DHG4	adenylate	I	I	I	Trichomonas vaginalis	
A2DJ01	adenylate	I	I	I	Trichomonas vaginalis	
A2DJS0	adenylate	I	I	I	Trichomonas vaginalis	
A2DKP6	adenylate	I	I	I	Trichomonas vaginalis	
A2DLQ6	adenylate	I	I	I	Trichomonas vaginalis	
A2DR64	adenylate	I	I	I	Trichomonas vaginalis	
A2DTD4	adenylate	I	I	I	Trichomonas vaginalis	
A2DWV8	adenylate	I	I	I	Trichomonas vaginalis	
A2DXS6	adenylate	I	I	I	Trichomonas vaginalis	
A2E4D6	adenylate	I	I	I	Trichomonas vaginalis	
A2EC33	adenylate	I	I	I	Trichomonas vaginalis	
A2ECD2	adenylate	I	I	I	Trichomonas vaginalis	
A2ES71	adenylate	I	I	I	Trichomonas vaginalis	
A2ESA8	adenylate	I	I	I	Trichomonas vaginalis	
A2ET16	adenylate	I	I	I	Trichomonas vaginalis	
A2EUD7	adenylate	I	I	I	Trichomonas vaginalis	
A2EZ60	adenylate	I	I	I	Trichomonas vaginalis	
A2EZ90	adenylate	I	I	I	Trichomonas vaginalis	
A2F6K2	adenylate	I	I	I	Trichomonas vaginalis	

Table S2.1. (continuation)

UniProt	Label [1]	Two	Three	Six	Species	EC Number
A2F6K8	adenylate	I	I	I	Trichomonas vaginalis	
A2FB45	adenylate	I	I	I	Trichomonas vaginalis	
A2FHK9	adenylate	I	I	I	Trichomonas vaginalis	
A2FM49	adenylate	I	I	I	Trichomonas vaginalis	
A2FPM7	adenylate	I	I	I	Trichomonas vaginalis	
A2FQQ4	adenylate	I	I	I	Trichomonas vaginalis	
A2FTS7	adenylate	I	I	I	Trichomonas vaginalis	
A2FXK2	adenylate	I	I	I	Trichomonas vaginalis	
A2FYJ1	adenylate	I	I	I	Trichomonas vaginalis	
A2FZG5	adenylate	I	I	I	Trichomonas vaginalis	
A2FZG6	adenylate	I	I	I	Trichomonas vaginalis	
A2G1D8	adenylate	I	I	I	Trichomonas vaginalis	
A2G3A5	adenylate	I	I	I	Trichomonas vaginalis	
A2G6L5	adenylate	I	I	I	Trichomonas vaginalis	
A2GHB4	adenylate	I	I	I	Trichomonas vaginalis	
A2H2H1	adenylate	I	I	I	Trichomonas vaginalis	
A2D795	adenylate	I	II	II	Trichomonas vaginalis	
A2DC46	adenylate	I	II	II	Trichomonas vaginalis	
A2DDF2	adenylate	I	II	II	Trichomonas vaginalis	
A2DFE4	adenylate	I	II	II	Trichomonas vaginalis	
A2DFM1	adenylate	I	II	II	Trichomonas vaginalis	
A2DJ96	adenylate	I	II	II	Trichomonas vaginalis	
A2DXM9	adenylate	I	II	II	Trichomonas vaginalis	
A2DY07	adenylate	I	II	II	Trichomonas vaginalis	
A2DYW0	adenylate	I	II	II	Trichomonas vaginalis	
A2E7P5	adenylate	I	II	II	Trichomonas vaginalis	
A2EEV5	adenylate	I	II	II	Trichomonas vaginalis	
A2EII5	adenylate	I	II	II	Trichomonas vaginalis	
A2EII6	adenylate	I	II	II	Trichomonas vaginalis	
A2EIJ6	adenylate	I	II	II	Trichomonas vaginalis	
A2EN10	adenylate	I	II	II	Trichomonas vaginalis	
A2EN44	adenylate	I	II	II	Trichomonas vaginalis	
A2EN84	adenylate	I	II	II	Trichomonas vaginalis	
A2EQD8	adenylate	I	II	II	Trichomonas vaginalis	
A2EQT4	adenylate	I	II	II	Trichomonas vaginalis	
A2EW55	adenylate	I	II	II	Trichomonas vaginalis	
A2EYR6	adenylate	I	II	II	Trichomonas vaginalis	
A2EZV4	adenylate	I	II	II	Trichomonas vaginalis	
A2F2E6	adenylate	I	II	II	Trichomonas vaginalis	
A2F3J3	adenylate	I	II	II	Trichomonas vaginalis	
A2F3K7	adenylate	I	II	II	Trichomonas vaginalis	
A2F3Y3	adenylate	I	II	II	Trichomonas vaginalis	
A2F426	adenylate	I	II	II	Trichomonas vaginalis	
A2FAE9	adenylate	I	II	II	Trichomonas vaginalis	
A2FBV8	adenylate	I	II	II	Trichomonas vaginalis	
A2FD08	adenylate	I	II	II	Trichomonas vaginalis	
A2FEF6	adenylate	I	II	II	Trichomonas vaginalis	
A2FET1	adenylate	I	II	II	Trichomonas vaginalis	
A2FFZ6	adenylate	I	II	II	Trichomonas vaginalis	
A2FG49	adenylate	I	II	II	Trichomonas vaginalis	
A2FIC7	adenylate	I	II	II	Trichomonas vaginalis	
A2FID0	adenylate	I	II	II	Trichomonas vaginalis	
A2FKW6	adenylate	I	II	II	Trichomonas vaginalis	
A2FLP2	adenylate	I	II	II	Trichomonas vaginalis	
A2FSM3	adenylate	I	II	II	Trichomonas vaginalis	
A2FVN7	adenylate	I	II	II	Trichomonas vaginalis	

Table S2.1. (continuation)

UniProt	Label [1]	Two	Three	Six	Species	EC Number
A2FW09	adenylate	I	II	II	Trichomonas vaginalis	
A2FWD6	adenylate	I	II	II	Trichomonas vaginalis	
A2FXW9	adenylate	I	II	II	Trichomonas vaginalis	
A2G426	adenylate	I	II	II	Trichomonas vaginalis	
A2G4Y5	adenylate	I	II	II	Trichomonas vaginalis	
A2G733	adenylate	I	II	II	Trichomonas vaginalis	
A2GA06	adenylate	I	II	II	Trichomonas vaginalis	
Q16W55	adenylate	I	II	III	Aedes aegypti	
A3HV63	adenylate	I	II	III	Algoriphagus machipongonensis	
A3HVA1	adenylate	I	II	III	Algoriphagus machipongonensis	
A3I1K5	adenylate	I	II	III	Algoriphagus machipongonensis	
Q8YZ26	adenylate	I	II	III	Anabaena sp	
A7BXS6	adenylate	I	II	III	Beggiatoa sp	
A7C2F8	adenylate	I	II	III	Beggiatoa sp	
Q89SW2	adenylate	I	II	III	Bradyrhizobium japonicum	
Q69HQ2	adenylate	I	II	III	Ciona intestinalis	
Q0K499	adenylate	I	II	III	Cupriavidus necator	4.6.1.1
Q0KDE7	adenylate	I	II	III	Cupriavidus necator	4.6.1.1
Q47AI8	adenylate	I	II	III	Dechloromonas aromatica	
Q1NSQ3	adenylate	I	II	III	delta proteobacterium	4.6.1.1
Q1NWK5	adenylate	I	II	III	delta proteobacterium	4.6.1.2
Q03101	adenylate	I	II	III	Dictyostelium discoideum	4.6.1.1
A8DXZ8	adenylate	I	II	III	Dictyostelium fasciculatum	
A8DXZ5	adenylate	I	II	III	Dictyostelium minutum	
A8DXZ9	adenylate	I	II	III	Dictyostelium mucoroides	
Q8MRM8	adenylate	I	II	III	Drosophila melanogaster	
Q8MZE0	adenylate	I	II	III	Drosophila melanogaster	
Q1PQ10	adenylate	I	II	III	Drosophila miranda	
A3WBP2	adenylate	I	II	III	Erythrobacter sp	
A0LXB4	adenylate	I	II	III	Gramella forsetii	4.6.1.-
Q68D02	adenylate	I	II	III	Homo sapiens	
Q71UM8	adenylate	I	II	III	Homo sapiens	
Q86YI0	adenylate	I	II	III	Homo sapiens	
Q8NBM1	adenylate	I	II	III	Homo sapiens	
Q28S34	adenylate	I	II	III	Jannaschia sp	
Q04QZ3	adenylate	I	II	III	Leptospira borgpetersenii	
Q72P78	adenylate	I	II	III	Leptospira interrogans	
Q72PY8	adenylate	I	II	III	Leptospira interrogans	
Q72TH6	adenylate	I	II	III	Leptospira interrogans	
Q8F1T7	adenylate	I	II	III	Leptospira interrogans	
Q8F690	adenylate	I	II	III	Leptospira interrogans	
Q8F778	adenylate	I	II	III	Leptospira interrogans	
A0YIQ8	adenylate	I	II	III	Lyngbya sp	
A0YL05	adenylate	I	II	III	Lyngbya sp	
A0YN57	adenylate	I	II	III	Lyngbya sp	
A0YPA5	adenylate	I	II	III	Lyngbya sp	
A0YPF4	adenylate	I	II	III	Lyngbya sp	
A0YQ82	adenylate	I	II	III	Lyngbya sp	
A0YS29	adenylate	I	II	III	Lyngbya sp	
A0YSC8	adenylate	I	II	III	Lyngbya sp	
A0YU66	adenylate	I	II	III	Lyngbya sp	
A0YVQ5	adenylate	I	II	III	Lyngbya sp	
A0LCD9	adenylate	I	II	III	Magnetococcus sp	
A4AWH6	adenylate	I	II	III	Maribacter sp	
Q1H0Z8	adenylate	I	II	III	Methylobacillus flagellatus	
A1ZFP4	adenylate	I	II	III	Microscilla marina	

Table S2.1. (continuation)

UniProt	Label [1]	Two	Three	Six	Species	EC Number
A1ZJP1	adenylate	I	II	III	Microscilla marina	
A1ZVN3	adenylate	I	II	III	Microscilla marina	
A1ZSW5	adenylate	I	II	III	Microscilla marina	4.6.1.1
A1ZW48	adenylate	I	II	III	Microscilla marina	4.6.1.1
A1ZXW2	adenylate	I	II	III	Microscilla marina	4.6.1.1
A1ZQH6	adenylate	I	II	III	Microscilla marina	4.6.1.2
A1ZSW4	adenylate	I	II	III	Microscilla marina	4.6.1.2
Q3UWC8	adenylate	I	II	III	Mus musculus	
Q5DTQ1	adenylate	I	II	III	Mus musculus	
Q6PG12	adenylate	I	II	III	Mus musculus	
Q80U07	adenylate	I	II	III	Mus musculus	
P0A4Y1	adenylate	I	II	III	Mycobacterium bovis	4.6.1.1
A4T9R0	adenylate	I	II	III	Mycobacterium gilvum	4.6.1.2
Q740N2	adenylate	I	II	III	Mycobacterium paratuberculosis	
A1UHH0	adenylate	I	II	III	Mycobacterium sp	
A1UK57	adenylate	I	II	III	Mycobacterium sp	
A2VIB4	adenylate	I	II	III	Mycobacterium tuberculosis	
A5U2Y1	adenylate	I	II	III	Mycobacterium tuberculosis	
A1T8Z6	adenylate	I	II	III	Mycobacterium vanbaalenii	
A1SD24	adenylate	I	II	III	Nocardioides sp	
Q33BP3	adenylate	I	II	III	Paralichthys olivaceus	
Q4XB02	adenylate	I	II	III	Plasmodium chabaudi	
O96856	adenylate	I	II	III	Plasmodium falciparum	
Q7RG96	adenylate	I	II	III	Plasmodium yoelii	
A8DXZ7	adenylate	I	II	III	Polysphondylium pallidum	
Q15T90	adenylate	I	II	III	Pseudoalteromonas atlantica	
A6V2K5	adenylate	I	II	III	Pseudomonas aeruginosa	
A4XTC1	adenylate	I	II	III	Pseudomonas mendocina	
Q46MB9	adenylate	I	II	III	Ralstonia eutropha	
Q7WWV9	adenylate	I	II	III	Ralstonia eutropha	
Q98GN7	adenylate	I	II	III	Rhizobium loti	
Q92WJ5	adenylate	I	II	III	Rhizobium meliloti	4.6.1.2
A7NLF3	adenylate	I	II	III	Roseiflexus castenholzii	
A5UTS7	adenylate	I	II	III	Roseiflexus sp	
Q5BXY8	adenylate	I	II	III	Schistosoma japonicum	
A6UFZ5	adenylate	I	II	III	Sinorhizobium medicae	4.6.1.2
Q9EXQ2	adenylate	I	II	III	Spirulina platensis	4.6.1.1
P40137	adenylate	I	II	III	Stigmatella aurantiaca	4.6.1.1
Q08SQ6	adenylate	I	II	III	Stigmatella aurantiaca	4.6.1.1
Q4RZV2	adenylate	I	II	III	Tetraodon nigroviridis	
Q4RZV3	adenylate	I	II	III	Tetraodon nigroviridis	
Q4S5M2	adenylate	I	II	III	Tetraodon nigroviridis	
Q73L79	adenylate	I	II	III	Treponema denticola	
Q73RD3	adenylate	I	II	III	Treponema denticola	
Q10XK2	adenylate	I	II	III	Trichodesmium erythraeum	
Q10XK8	adenylate	I	II	III	Trichodesmium erythraeum	
Q112E0	adenylate	I	II	III	Trichodesmium erythraeum	
A2G2Z5	adenylate	I	II	III	Trichomonas vaginalis	
A7RY93	adenylate	I	II	VI	Nematostella vectensis	
A0CM46	adenylate	I	III	III	Paramecium tetraurelia	
A0DT50	adenylate	I	III	III	Paramecium tetraurelia	
A0DT51	adenylate	I	III	IV	Paramecium tetraurelia	
Q7RKA2	adenylate	II	II	III	Plasmodium yoelii	
Q5UFR4	guanylate	I	II	III	Mycobacterium smegmatis	4.6.1.1
Q4YH96	guanylate	II	III	III	Plasmodium berghei	
Q4XVA2	guanylate	II	III	III	Plasmodium chabaudi	

Table S2.1. (continuation)

UniProt	Label [1]	Two	Three	Six	Species	EC Number
Q17L76	guanylate	II	III	IV	Aedes aegypti	
Q7QF31	guanylate	II	III	IV	Anopheles gambiae	
Q5W7P2	guanylate	II	III	IV	Apis mellifera	
Q7YWK7	guanylate	II	III	IV	Aplysia californica	
Q7YZQ2	guanylate	II	III	IV	Bactrocera dorsalis	
P19687	guanylate	II	III	IV	Bos taurus	4.6.1.2
Q86C56	guanylate	II	III	IV	Caenorhabditis elegans	4.6.1.2
Q4ZHS0	guanylate	II	III	IV	Canis familiaris	4.6.1.2
Q07093	guanylate	II	III	IV	Drosophila melanogaster	4.6.1.2
Q29BU7	guanylate	II	III	IV	Drosophila pseudoobscura	4.6.1.2
Q90VV5	guanylate	II	III	IV	Fugu rubripes	
Q9NNW8	guanylate	II	III	IV	Homo sapiens	
P33402	guanylate	II	III	IV	Homo sapiens	4.6.1.2
Q02108	guanylate	II	III	IV	Homo sapiens	4.6.1.2
Q7YW37	guanylate	II	III	IV	Limax marginatus	
O77105	guanylate	II	III	IV	Manduca sexta	
Q3TNN8	guanylate	II	III	IV	Mus musculus	
Q9ERL9	guanylate	II	III	IV	Mus musculus	4.6.1.2
A7RN71	guanylate	II	III	IV	Nematostella vectensis	
A7RN72	guanylate	II	III	IV	Nematostella vectensis	
Q7T040	guanylate	II	III	IV	Oryzias curvinotus	
Q7T041	guanylate	II	III	IV	Oryzias curvinotus	
Q7ZSZ5	guanylate	II	III	IV	Oryzias latipes	
Q9PWI2	guanylate	II	III	IV	Oryzias latipes	
P79997	guanylate	II	III	IV	Oryzias latipes	4.6.1.2
Q5U330	guanylate	II	III	IV	Rattus norvegicus	
Q8CH90	guanylate	II	III	IV	Rattus norvegicus	
Q8CJD2	guanylate	II	III	IV	Rattus norvegicus	
P19686	guanylate	II	III	IV	Rattus norvegicus	4.6.1.2
Q9WVI4	guanylate	II	III	IV	Rattus norvegicus	4.6.1.2
Q4ZHR8	guanylate	II	III	IV	Sus scrofa	
Q16E68	guanylate	II	III	V	Aedes aegypti	
Q17A19	guanylate	II	III	V	Aedes aegypti	
Q7KQ93	guanylate	II	III	V	Anopheles gambiae	
Q7PS01	guanylate	II	III	V	Anopheles gambiae	
Q5UAF0	guanylate	II	III	V	Apis mellifera	
Q6L5L6	guanylate	II	III	V	Apis mellifera	
P16068	guanylate	II	III	V	Bos taurus	4.6.1.2
Q4ZHR9	guanylate	II	III	V	Canis familiaris	4.6.1.2
Q24086	guanylate	II	III	V	Drosophila melanogaster	
Q9VA09	guanylate	II	III	V	Drosophila melanogaster	4.6.1.2
Q29CE1	guanylate	II	III	V	Drosophila pseudoobscura	4.6.1.2
Q90VY5	guanylate	II	III	V	Fugu rubripes	
A2SW27	guanylate	II	III	V	Gecarcinus lateralis	
A2SW28	guanylate	II	III	V	Gecarcinus lateralis	
Q95NK5	guanylate	II	III	V	Hemicentrotus pulcherrimus	
Q02153	guanylate	II	III	V	Homo sapiens	4.6.1.2
O77106	guanylate	II	III	V	Manduca sexta	
Q3UTI4	guanylate	II	III	V	Mus musculus	
Q80YP4	guanylate	II	III	V	Mus musculus	
A7RN69	guanylate	II	III	V	Nematostella vectensis	
A7RN70	guanylate	II	III	V	Nematostella vectensis	
A7RRE5	guanylate	II	III	V	Nematostella vectensis	
Q762I7	guanylate	II	III	V	Oncorhynchus nerka	
P79998	guanylate	II	III	V	Oryzias latipes	4.6.1.2
Q80WY4	guanylate	II	III	V	Rattus norvegicus	

Table S2.1. (continuation)

UniProt	Label [1]	Two	Three	Six	Species	EC Number
Q80WY5	guanylate	II	III	V	Rattus norvegicus	
Q8CH85	guanylate	II	III	V	Rattus norvegicus	
P20595	guanylate	II	III	V	Rattus norvegicus	4.6.1.2
Q4ZHR7	guanylate	II	III	V	Sus scrofa	
Q6DLY7	guanylate	II	III	V	Sus scrofa	
Q6INK9	guanylate	II	III	V	Xenopus laevis	
Q16G04	guanylate	II	III	VI	Aedes aegypti	
Q16JH8	guanylate	II	III	VI	Aedes aegypti	
Q16KE1	guanylate	II	III	VI	Aedes aegypti	
Q17BS5	guanylate	II	III	VI	Aedes aegypti	
Q17BS6	guanylate	II	III	VI	Aedes aegypti	
Q16KH2	guanylate	II	III	VI	Aedes aegypti	4.6.1.2
Q16QE2	guanylate	II	III	VI	Aedes aegypti	4.6.1.2
Q16YZ5	guanylate	II	III	VI	Aedes aegypti	4.6.1.2
Q16YZ6	guanylate	II	III	VI	Aedes aegypti	4.6.1.2
Q172J7	guanylate	II	III	VI	Aedes aegypti	4.6.1.2
Q174S2	guanylate	II	III	VI	Aedes aegypti	4.6.1.2
Q17AF9	guanylate	II	III	VI	Aedes aegypti	4.6.1.2
Q90YD3	guanylate	II	III	VI	Anguilla anguilla	4.6.1.2
O93490	guanylate	II	III	VI	Anguilla japonica	4.6.1.2
P55202	guanylate	II	III	VI	Anguilla japonica	4.6.1.2
Q1MX20	guanylate	II	III	VI	Anguilla japonica	4.6.1.2
Q7PYK9	guanylate	II	III	VI	Anopheles gambiae	
Q7Q9W6	guanylate	II	III	VI	Anopheles gambiae	
Q7Q9X3	guanylate	II	III	VI	Anopheles gambiae	
Q7Q9X4	guanylate	II	III	VI	Anopheles gambiae	
Q7PTL8	guanylate	II	III	VI	Anopheles gambiae	4.6.1.2
Q7PTP9	guanylate	II	III	VI	Anopheles gambiae	4.6.1.2
Q7PWU5	guanylate	II	III	VI	Anopheles gambiae	4.6.1.2
Q7PZZ2	guanylate	II	III	VI	Anopheles gambiae	4.6.1.2
Q7PZZ3	guanylate	II	III	VI	Anopheles gambiae	4.6.1.2
Q7Q8C9	guanylate	II	III	VI	Anopheles gambiae	4.6.1.2
Q5FAN0	guanylate	II	III	VI	Apis mellifera	
Q8TA72	guanylate	II	III	VI	Asterias amurensis	4.6.1.2
Q7Z0H3	guanylate	II	III	VI	Bactrocera dorsalis	4.6.1.2
Q9BPR0	guanylate	II	III	VI	Bombyx mori	4.6.1.2
O02740	guanylate	II	III	VI	Bos taurus	4.6.1.2
O77690	guanylate	II	III	VI	Bos taurus	4.6.1.2
P46197	guanylate	II	III	VI	Bos taurus	4.6.1.2
P55203	guanylate	II	III	VI	Bos taurus	4.6.1.2
O97052	guanylate	II	III	VI	Brissus agassizii	4.6.1.2
Q9I9N7	guanylate	II	III	VI	Bufo marinus	4.6.1.2
O02298	guanylate	II	III	VI	Caenorhabditis elegans	4.6.1.2
O16544	guanylate	II	III	VI	Caenorhabditis elegans	4.6.1.2
O16715	guanylate	II	III	VI	Caenorhabditis elegans	4.6.1.2
O62026	guanylate	II	III	VI	Caenorhabditis elegans	4.6.1.2
O62179	guanylate	II	III	VI	Caenorhabditis elegans	4.6.1.2
P90895	guanylate	II	III	VI	Caenorhabditis elegans	4.6.1.2
P91550	guanylate	II	III	VI	Caenorhabditis elegans	4.6.1.2
P92006	guanylate	II	III	VI	Caenorhabditis elegans	4.6.1.2
Q09435	guanylate	II	III	VI	Caenorhabditis elegans	4.6.1.2
Q10028	guanylate	II	III	VI	Caenorhabditis elegans	4.6.1.2
Q10029	guanylate	II	III	VI	Caenorhabditis elegans	4.6.1.2
Q18331	guanylate	II	III	VI	Caenorhabditis elegans	4.6.1.2
Q19187	guanylate	II	III	VI	Caenorhabditis elegans	4.6.1.2
Q19768	guanylate	II	III	VI	Caenorhabditis elegans	4.6.1.2

Table S2.1. (continuation)

UniProt	Label [1]	Two	Three	Six	Species	EC Number
Q23310	guanylate	II	III	VI	Caenorhabditis elegans	4.6.1.2
Q23681	guanylate	II	III	VI	Caenorhabditis elegans	4.6.1.2
Q23682	guanylate	II	III	VI	Caenorhabditis elegans	4.6.1.2
Q6DNF3	guanylate	II	III	VI	Caenorhabditis elegans	4.6.1.2
Q6DNF4	guanylate	II	III	VI	Caenorhabditis elegans	4.6.1.2
Q6DNF7	guanylate	II	III	VI	Caenorhabditis elegans	4.6.1.2
Q86GV3	guanylate	II	III	VI	Caenorhabditis elegans	4.6.1.2
Q8I4N4	guanylate	II	III	VI	Caenorhabditis elegans	4.6.1.2
Q8T8L6	guanylate	II	III	VI	Caenorhabditis elegans	4.6.1.2
Q9GYQ4	guanylate	II	III	VI	Caenorhabditis elegans	4.6.1.2
Q9XTY1	guanylate	II	III	VI	Caenorhabditis elegans	4.6.1.2
Q9XU42	guanylate	II	III	VI	Caenorhabditis elegans	4.6.1.2
Q24LS5	guanylate	II	III	VI	Callinectes sapidus	4.6.1.2
O19179	guanylate	II	III	VI	Canis familiaris	4.6.1.2
P70106	guanylate	II	III	VI	Cavia porcellus	4.6.1.2
Q5YLC2	guanylate	II	III	VI	Chlamydomonas reinhardtii	
Q94428	guanylate	II	III	VI	Ciona intestinalis	4.6.1.2
Q94429	guanylate	II	III	VI	Ciona intestinalis	4.6.1.2
Q90WX1	guanylate	II	III	VI	Danio rerio	
A2BFQ0	guanylate	II	III	VI	Danio rerio	4.6.1.2
Q1LX84	guanylate	II	III	VI	Danio rerio	4.6.1.2
Q9U8P5	guanylate	II	III	VI	Diadema setosum	4.6.1.2
Q6E6T4	guanylate	II	III	VI	Dictyocaulus viviparus	
Q6E6T5	guanylate	II	III	VI	Dictyocaulus viviparus	
A2RVE6	guanylate	II	III	VI	Drosophila melanogaster	
Q1EC29	guanylate	II	III	VI	Drosophila melanogaster	
A1ZB47	guanylate	II	III	VI	Drosophila melanogaster	4.6.1.2
A8DYZ3	guanylate	II	III	VI	Drosophila melanogaster	4.6.1.2
Q07553	guanylate	II	III	VI	Drosophila melanogaster	4.6.1.2
Q0KI72	guanylate	II	III	VI	Drosophila melanogaster	4.6.1.2
Q24051	guanylate	II	III	VI	Drosophila melanogaster	4.6.1.2
Q7JQ32	guanylate	II	III	VI	Drosophila melanogaster	4.6.1.2
Q8INF0	guanylate	II	III	VI	Drosophila melanogaster	4.6.1.2
Q8IQK2	guanylate	II	III	VI	Drosophila melanogaster	4.6.1.2
Q8MLX0	guanylate	II	III	VI	Drosophila melanogaster	4.6.1.2
Q961R7	guanylate	II	III	VI	Drosophila melanogaster	4.6.1.2
Q9VEU5	guanylate	II	III	VI	Drosophila melanogaster	4.6.1.2
Q9VEU6	guanylate	II	III	VI	Drosophila melanogaster	4.6.1.2
Q9VF17	guanylate	II	III	VI	Drosophila melanogaster	4.6.1.2
Q9VU79	guanylate	II	III	VI	Drosophila melanogaster	4.6.1.2
Q9W2P1	guanylate	II	III	VI	Drosophila melanogaster	4.6.1.2
Q296R5	guanylate	II	III	VI	Drosophila pseudoobscura	4.6.-.-
Q28X38	guanylate	II	III	VI	Drosophila pseudoobscura	4.6.1.2
Q296C7	guanylate	II	III	VI	Drosophila pseudoobscura	4.6.1.2
Q296C8	guanylate	II	III	VI	Drosophila pseudoobscura	4.6.1.2
Q296Q0	guanylate	II	III	VI	Drosophila pseudoobscura	4.6.1.2
Q29E17	guanylate	II	III	VI	Drosophila pseudoobscura	4.6.1.2
Q9NH71	guanylate	II	III	VI	Eptatretus cirrhatus	
O57480	guanylate	II	III	VI	Gallus gallus	4.6.1.2
A0SEL0	guanylate	II	III	VI	Gecarcinus lateralis	
A2SW29	guanylate	II	III	VI	Gecarcinus lateralis	4.6.1.2
A2SW30	guanylate	II	III	VI	Gecarcinus lateralis	4.6.1.2
A2SW31	guanylate	II	III	VI	Gecarcinus lateralis	4.6.1.2
Q27669	guanylate	II	III	VI	Hemicentrotus pulcherrimus	4.6.1.2
O96993	guanylate	II	III	VI	Heterodera glycines	4.6.1.2
Q9GPU1	guanylate	II	III	VI	Heterodera glycines	4.6.1.2

Table S2.1. (continuation)

UniProt	Label [1]	Two	Three	Six	Species	EC Number
Q9GT36	guanylate	II	III	VI	Heterodera glycines	4.6.1.2
Q9GT37	guanylate	II	III	VI	Heterodera glycines	4.6.1.2
O75343	guanylate	II	III	VI	Homo sapiens	4.6.1.2
P16066	guanylate	II	III	VI	Homo sapiens	4.6.1.2
P20594	guanylate	II	III	VI	Homo sapiens	4.6.1.2
P25092	guanylate	II	III	VI	Homo sapiens	4.6.1.2
P51841	guanylate	II	III	VI	Homo sapiens	4.6.1.2
Q02846	guanylate	II	III	VI	Homo sapiens	4.6.1.2
Q7YXG1	guanylate	II	III	VI	Limax marginatus	
Q7YXG2	guanylate	II	III	VI	Limax marginatus	
O76340	guanylate	II	III	VI	Manduca sexta	
O77149	guanylate	II	III	VI	Manduca sexta	
Q8IT60	guanylate	II	III	VI	Manduca sexta	4.6.1.2
Q6XE41	guanylate	II	III	VI	Mus musculus	
Q8BXH3	guanylate	II	III	VI	Mus musculus	
P18293	guanylate	II	III	VI	Mus musculus	4.6.1.2
P52785	guanylate	II	III	VI	Mus musculus	4.6.1.2
Q2TAY4	guanylate	II	III	VI	Mus musculus	4.6.1.2
Q3TZ08	guanylate	II	III	VI	Mus musculus	4.6.1.2
Q3UWA6	guanylate	II	III	VI	Mus musculus	4.6.1.2
Q3UY30	guanylate	II	III	VI	Mus musculus	4.6.1.2
Q5SDA5	guanylate	II	III	VI	Mus musculus	4.6.1.2
Q6TL19	guanylate	II	III	VI	Mus musculus	4.6.1.2
Q6VVW5	guanylate	II	III	VI	Mus musculus	4.6.1.2
Q8R1P8	guanylate	II	III	VI	Mus musculus	4.6.1.2
Q91X04	guanylate	II	III	VI	Mus musculus	4.6.1.2
A7RGN1	guanylate	II	III	VI	Nematostella vectensis	
A7RSZ0	guanylate	II	III	VI	Nematostella vectensis	
A7RZH8	guanylate	II	III	VI	Nematostella vectensis	
A7S7B2	guanylate	II	III	VI	Nematostella vectensis	
A7S7B3	guanylate	II	III	VI	Nematostella vectensis	
A7SPS8	guanylate	II	III	VI	Nematostella vectensis	
A7RUA8	guanylate	II	III	VI	Nematostella vectensis	4.6.1.2
A7SJA1	guanylate	II	III	VI	Nematostella vectensis	4.6.1.2
A7SJA3	guanylate	II	III	VI	Nematostella vectensis	4.6.1.2
A7SJA4	guanylate	II	III	VI	Nematostella vectensis	4.6.1.2
A7SPS9	guanylate	II	III	VI	Nematostella vectensis	4.6.1.2
A7SZ80	guanylate	II	III	VI	Nematostella vectensis	4.6.1.2
A7T7X3	guanylate	II	III	VI	Nematostella vectensis	4.6.1.2
Q75V79	guanylate	II	III	VI	Oryzias curvinotus	
Q75V83	guanylate	II	III	VI	Oryzias curvinotus	
O93253	guanylate	II	III	VI	Oryzias latipes	
Q75V85	guanylate	II	III	VI	Oryzias latipes	
O42129	guanylate	II	III	VI	Oryzias latipes	4.6.1.2
O42440	guanylate	II	III	VI	Oryzias latipes	4.6.1.2
P79999	guanylate	II	III	VI	Oryzias latipes	4.6.1.2
P87342	guanylate	II	III	VI	Oryzias latipes	4.6.1.2
P87343	guanylate	II	III	VI	Oryzias latipes	4.6.1.2
Q75Q01	guanylate	II	III	VI	Oryzias latipes	4.6.1.2
Q75V80	guanylate	II	III	VI	Oryzias latipes	4.6.1.2
Q7T2Q2	guanylate	II	III	VI	Oryzias latipes	4.6.1.2
Q7T3I2	guanylate	II	III	VI	Oryzias latipes	4.6.1.2
Q7ZZR6	guanylate	II	III	VI	Oryzias latipes	4.6.1.2
Q98UH9	guanylate	II	III	VI	Oryzias latipes	4.6.1.2
Q98UI0	guanylate	II	III	VI	Oryzias latipes	4.6.1.2
Q98UI1	guanylate	II	III	VI	Oryzias latipes	4.6.1.2

Table S2.1. (continuation)

UniProt	Label [1]	Two	Three	Six	Species	EC Number
Q9PVW4	guanylate	II	III	VI	Oryzias latipes	4.6.1.2
Q9PWG1	guanylate	II	III	VI	Oryzias latipes	4.6.1.2
Q9W614	guanylate	II	III	VI	Oryzias latipes	4.6.1.2
Q9W615	guanylate	II	III	VI	Oryzias latipes	4.6.1.2
Q9W616	guanylate	II	III	VI	Oryzias latipes	4.6.1.2
Q9YGW3	guanylate	II	III	VI	Oryzias latipes	4.6.1.2
Q6UCJ4	guanylate	II	III	VI	Procambarus clarkii	4.6.1.2
Q90YB7	guanylate	II	III	VI	Rana catesbeiana	4.6.1.2
Q8JGM3	guanylate	II	III	VI	Rana pipiens	4.6.1.2
Q9PUK0	guanylate	II	III	VI	Rana pipiens	4.6.1.2
Q80WX7	guanylate	II	III	VI	Rattus norvegicus	
Q80WX8	guanylate	II	III	VI	Rattus norvegicus	
Q80WX9	guanylate	II	III	VI	Rattus norvegicus	
Q80WY0	guanylate	II	III	VI	Rattus norvegicus	
Q91XJ7	guanylate	II	III	VI	Rattus norvegicus	
Q920Q1	guanylate	II	III	VI	Rattus norvegicus	
P16067	guanylate	II	III	VI	Rattus norvegicus	4.6.1.2
P18910	guanylate	II	III	VI	Rattus norvegicus	4.6.1.2
P22717	guanylate	II	III	VI	Rattus norvegicus	4.6.1.2
P23897	guanylate	II	III	VI	Rattus norvegicus	4.6.1.2
P51839	guanylate	II	III	VI	Rattus norvegicus	4.6.1.2
P51840	guanylate	II	III	VI	Rattus norvegicus	4.6.1.2
P51842	guanylate	II	III	VI	Rattus norvegicus	4.6.1.2
P55205	guanylate	II	III	VI	Rattus norvegicus	4.6.1.2
Q6LD45	guanylate	II	III	VI	Rattus sp	
Q9YI17	guanylate	II	III	VI	Squalus acanthias	4.6.1.2
O97053	guanylate	II	III	VI	Stichopus japonicus	4.6.1.2
P16065	guanylate	II	III	VI	Strongylocentrotus purpuratus	4.6.1.2
P55204	guanylate	II	III	VI	Sus scrofa	4.6.1.2
Q4T0M0	guanylate	II	III	VI	Tetraodon nigroviridis	
Q4TB76	guanylate	II	III	VI	Tetraodon nigroviridis	
Q4TGP7	guanylate	II	III	VI	Tetraodon nigroviridis	
Q4RYJ0	guanylate	II	III	VI	Tetraodon nigroviridis	4.6.1.2
Q4S8Y6	guanylate	II	III	VI	Tetraodon nigroviridis	4.6.1.2
Q4SW10	guanylate	II	III	VI	Tetraodon nigroviridis	4.6.1.2
Q4SY29	guanylate	II	III	VI	Tetraodon nigroviridis	4.6.1.2
Q4T342	guanylate	II	III	VI	Tetraodon nigroviridis	4.6.1.2
Q4T5G2	guanylate	II	III	VI	Tetraodon nigroviridis	4.6.1.2
Q4T608	guanylate	II	III	VI	Tetraodon nigroviridis	4.6.1.2
Q4T7S3	guanylate	II	III	VI	Tetraodon nigroviridis	4.6.1.2
Q4T9J5	guanylate	II	III	VI	Tetraodon nigroviridis	4.6.1.2
Q4TAT8	guanylate	II	III	VI	Tetraodon nigroviridis	4.6.1.2
A1YQY9	guanylate	II	III	VI	Volvox carteri	
A1L2S6	guanylate	II	III	VI	Xenopus laevis	4.6.1.2
P79991	guanylate	II	III	VI	Xenopus laevis	4.6.1.2
Q6P409	guanylate	II	III	VI	Xenopus laevis	4.6.1.2
Q9PWG9	guanylate	II	III	VI	Xenopus laevis	4.6.1.2
Q9PWH0	guanylate	II	III	VI	Xenopus laevis	4.6.1.2

Case study II: DUF849

The set of 725 proteins is listed in Table S2.2, along with the corresponding groups in [2], the clusters they were placed in by the proposed framework when considering seven clusters, as well as the source organisms and existing EC numbers according to UniProt.

Table S2.2. List of proteins in the DUF849 family.

UniProt	Group [2]	Cluster	Species	EC Number
A5G287	G1	V	Acidiphilium cryptum	
A8TUX9	G1	V	alpha proteobacterium	
O29058	G1	V	Archaeoglobus fulgidus	
Q1YGU1	G1	V	Aurantimonas manganoxydans	
A8IMP9	G1	V	Azorhizobium caulinodans	
A9I628	G1	V	Bordetella petrii	
B4W864	G1	V	Brevundimonas sp	
B1FHG8	G1	V	Burkholderia ambifaria	
B1T6J0	G1	V	Burkholderia ambifaria	
B1Z3A7	G1	V	Burkholderia ambifaria	
Q0B970	G1	V	Burkholderia ambifaria	
A0B091	G1	V	Burkholderia cenocepacia	
A2VZ04	G1	V	Burkholderia cenocepacia	
B1K866	G1	V	Burkholderia cenocepacia	
B4EJ15	G1	V	Burkholderia cenocepacia	
Q1BN83	G1	V	Burkholderia cenocepacia	
A2WET2	G1	V	Burkholderia dolosa	
B1G2Y1	G1	V	Burkholderia graminis	
B1G5Y9	G1	V	Burkholderia graminis	
B1G6N0	G1	V	Burkholderia graminis	
A2RYA7	G1	V	Burkholderia mallei	
A3MFH5	G1	V	Burkholderia mallei	
C4ANE6	G1	V	Burkholderia mallei	
Q62D63	G1	V	Burkholderia mallei	
A9AJY3	G1	V	Burkholderia multivorans	
A9AKI5	G1	V	Burkholderia multivorans	
B9B0H7	G1	V	Burkholderia multivorans	
B9B900	G1	V	Burkholderia multivorans	
B9BIH7	G1	V	Burkholderia multivorans	
B9BMP0	G1	V	Burkholderia multivorans	
B2T139	G1	V	Burkholderia phytofirmans	
A3NIF0	G1	V	Burkholderia pseudomallei	
A3P412	G1	V	Burkholderia pseudomallei	
A8EKN6	G1	V	Burkholderia pseudomallei	
B1H571	G1	V	Burkholderia pseudomallei	
C4I1Z3	G1	V	Burkholderia pseudomallei	
Q3JG15	G1	V	Burkholderia pseudomallei	
Q63M97	G1	V	Burkholderia pseudomallei	
Q39LC1	G1	V	Burkholderia sp	
Q13G66	G1	V	Burkholderia xenovorans	
Q13GE9	G1	V	Burkholderia xenovorans	
Q13GF8	G1	V	Burkholderia xenovorans	
Q13I95	G1	V	Burkholderia xenovorans	
B0T8Y7	G1	V	Caulobacter sp	
D0DDZ7	G1	V	Citreicella sp	
Q0KFN7	G1	V	Cupriavidus necator	
Q46N76	G1	V	Cupriavidus pinatubonensis	
Q471G6	G1	V	Cupriavidus pinatubonensis	
A9BLU8	G1	V	Delftia acidovorans	
B8FAE2	G1	V	Desulfatibacillum alkenivorans	
Q2N7W2	G1	V	Erythrobacter litoralis	
C6JMJ2	G1	V	Fusobacterium varium	
C6JPY2	G1	V	Fusobacterium varium	
D2S5G4	G1	V	Geodermatophilus obscurus	
D3AIC1	G1	V	Hungatella hathewayi	
A0NQY0	G1	V	Labrenzia aggregata	

Table S2.2. (continuation)

UniProt	Group [2]	Cluster	Species	EC Number
A0P135	G1	V	Labrenzia aggregata	
A3JCS3	G1	V	Marinobacter sp	
A2SJ75	G1	V	Methylibium petroleiphilum	
B8IQA1	G1	V	Methylobacterium nodulans	
B1M404	G1	V	Methylobacterium radiotolerans	
B0U8S2	G1	V	Methylobacterium sp	
A1SFV1	G1	V	Nocardioides sp	
Q2CA34	G1	V	Oceanicola granulosus	
Q2CEL3	G1	V	Oceanicola granulosus	
A6X2V8	G1	V	Ochrobactrum anthropi	
A1B802	G1	V	Paracoccus denitrificans	
A1BAQ1	G1	V	Paracoccus denitrificans	
A1BB15	G1	V	Paracoccus denitrificans	
Q0FNU4	G1	V	Pelagibaca bermudensis	
Q0FWB8	G1	V	Pelagibaca bermudensis	
Q15TE9	G1	V	Pseudoalteromonas atlantica	
C3K4S4	G1	V	Pseudomonas fluorescens	
Q4KGM4	G1	V	Pseudomonas fluorescens	
B1J5X8	G1	V	Pseudomonas putida	
B1JF47	G1	V	Pseudomonas putida	
Q4ZUE4	G1	V	Pseudomonas syringae	
Q1LCH6	G1	V	Ralstonia metallidurans	
Q98LG3	G1	V	Rhizobium loti	
Q98NK0	G1	V	Rhizobium loti	
A3PR57	G1	V	Rhodobacter sphaeroides	
A4WZE5	G1	V	Rhodobacter sphaeroides	
B9KX09	G1	V	Rhodobacter sphaeroides	
Q3IVD8	G1	V	Rhodobacter sphaeroides	
Q3IXV4	G1	V	Rhodobacter sphaeroides	
A3JMN2	G1	V	Rhodobacteraceae bacterium	
B6ASR2	G1	V	Rhodobacteraceae bacterium	
Q216X8	G1	V	Rhodopseudomonas palustris	
Q21D54	G1	V	Rhodopseudomonas palustris	
Q160J5	G1	V	Roseobacter denitrificans	
A3XAN7	G1	V	Roseobacter sp	
A6FVA7	G1	V	Roseobacter sp	
B7RSG6	G1	V	Roseobacter sp	
A3SJ19	G1	V	Roseovarius nubinhibens	
A3W4W3	G1	V	Roseovarius sp	
A6E0U6	G1	V	Roseovarius sp	
Q1ARS8	G1	V	Rubrobacter xylanophilus	
Q5LRW7	G1	V	Ruegeria pomeroyi	
A3K426	G1	V	Sagittula stellata	
A3K768	G1	V	Sagittula stellata	
A3K7W2	G1	V	Sagittula stellata	
Q1NHC2	G1	V	Sphingomonas sp	
A5V8P7	G1	V	Sphingomonas wittichii	
A5VAW0	G1	V	Sphingomonas wittichii	
A5VAX6	G1	V	Sphingomonas wittichii	
A5VBU5	G1	V	Sphingomonas wittichii	
A5VHA1	G1	V	Sphingomonas wittichii	
Q6SEY1	G1	V	uncultured marine	
A1WRZ4	G1	V	Verminephrobacter eiseniae	
A7ICE1	G1	V	Xanthobacter autotrophicus	
A4LHL9	G1	V		
A5JCV7	G1	V		

Table S2.2. (continuation)

UniProt	Group [2]	Cluster	Species	EC Number
A5TD73	G1	V		
A5XXU1	G1	V		
A8KR21	G1	V		
A9H7X2	G1	V		
A9HDK4	G1	V		
A9HUU5	G1	V		
A9JZD2	G1	V		
B2H6S6	G1	V		
B7CG13	G1	V		
B9C2I4	G1	V		
B9C654	G1	V		
C0Y1C7	G1	V		
C5ZSW7	G1	V		
C6U1U4	G1	V		
C8SFA5	G1	V		
C8SI24	G1	V		
D0XME6	G1	V		
D1T5K5	G1	V		
D1UU49	G1	V		
B0VLL9	G1	VII	Acinetobacter baumannii	
B0UH10	G1	VII	Methylobacterium sp	
B0MVB0	G2	VII	Alistipes putredinis	
A6TK30	G2	VII	Alkaliphilus metalliredigens	
A8MJZ6	G2	VII	Alkaliphilus oremlandii	
B8JC66	G2	VII	Anaeromyxobacter dehalogenans	
Q2IHY0	G2	VII	Anaeromyxobacter dehalogenans	
A7HCV1	G2	VII	Anaeromyxobacter sp	
B4UEN6	G2	VII	Anaeromyxobacter sp	
B7R880	G2	VII	Caldanaerobacter subterraneus	
B0VHH0	G2	VII	Cloacamonas acidaminovorans	2.3.1.247
C0GEB6	G2	VII	Dethiobacter alkaliphilus	
A7HJJ3	G2	VII	Fervidobacterium nodosum	
A5TV53	G2	VII	Fusobacterium nucleatum	
C7XS29	G2	VII	Fusobacterium nucleatum	
Q7P5H7	G2	VII	Fusobacterium nucleatum	
Q8RHX2	G2	VII	Fusobacterium nucleatum	2.3.1.247
C6JK84	G2	VII	Fusobacterium varium	
A3TRJ7	G2	VII	Janibacter sp	
C5CH08	G2	VII	Kosmotoga olearia	
D2Q1A4	G2	VII	Kribbella flavida	
A4AIW0	G2	VII	marine actinobacterium	
C4RCX9	G2	VII	Micromonospora sp	
Q1D466	G2	VII	Myxococcus xanthus	
B2A639	G2	VII	Natranaerobius thermophilus	
B2A657	G2	VII	Natranaerobius thermophilus	
A1SPI8	G2	VII	Nocardioides sp	
A9BG41	G2	VII	Petrotoga mobilis	
C3JBK7	G2	VII	Porphyromonas endodontalis	
B2RJY8	G2	VII	Porphyromonas gingivalis	
Q7MVJ3	G2	VII	Porphyromonas gingivalis	
C2M974	G2	VII	Porphyromonas uenonis	
Q21RL9	G2	VII	Rhodoferax ferrireducens	
A7NLI9	G2	VII	Roseiflexus castenholzii	
A5UTH8	G2	VII	Roseiflexus sp	
A8LZE0	G2	VII	Salinispora arenicola	
A4X1P9	G2	VII	Salinispora tropica	

Table S2.2. (continuation)

UniProt	Group [2]	Cluster	Species	EC Number
A9GGS5	G2	VII	Sorangium cellulosum	
Q08SN2	G2	VII	Stigmatella aurantiaca	
Q67TE4	G2	VII	Symbiobacterium thermophilum	
Q8RBU0	G2	VII	Thermoanaerobacter tengcongensis	
D1A4L8	G2	VII	Thermomonospora curvata	
B7IFQ1	G2	VII	Thermosipho africanus	
A6LJG6	G2	VII	Thermosipho melanesiensis	
A8F3R2	G2	VII	Thermotoga lettingae	
A1X0G5	G2	VII	unidentified	
C3WJN8	G2	VII		
C3WT25	G2	VII		
C3WZN8	G2	VII		
D0BS35	G2	VII		
D1S7G4	G2	VII		
D3CH25	G2	VII		
A1AZ89	G3	V	Paracoccus denitrificans	
Q21UM7	G3	VII	Albidiferax ferrireducens	
A8MJJ3	G3	VII	Alkaliphilus oremlandii	
Q1N164	G3	VII	Bermanella marisrubri	
Q89VR0	G3	VII	Bradyrhizobium japonicum	
A4Z2U4	G3	VII	Bradyrhizobium sp	
A5E9U7	G3	VII	Bradyrhizobium sp	
B1G422	G3	VII	Burkholderia graminis	
A3NNK3	G3	VII	Burkholderia pseudomallei	
A3P8Z7	G3	VII	Burkholderia pseudomallei	
A8EMV4	G3	VII	Burkholderia pseudomallei	
B1H9J5	G3	VII	Burkholderia pseudomallei	
C4I9S9	G3	VII	Burkholderia pseudomallei	
Q3JJE3	G3	VII	Burkholderia pseudomallei	
Q63IN4	G3	VII	Burkholderia pseudomallei	
Q2T8L2	G3	VII	Burkholderia thailandensis	
Q13GJ3	G3	VII	Burkholderia xenovorans	
C5ESS4	G3	VII	Clostridiales bacterium	
A8RXS1	G3	VII	Clostridium bolteae	
B7WZX3	G3	VII	Comamonas testosteroni	
D0IWE4	G3	VII	Comamonas testosteroni	
D0J5J6	G3	VII	Comamonas testosteroni	
D3F046	G3	VII	Conexibacter woesei	
D3F707	G3	VII	Conexibacter woesei	
Q0K095	G3	VII	Cupriavidus necator	
Q46MU0	G3	VII	Cupriavidus pinatubonensis	
Q46NT8	G3	VII	Cupriavidus pinatubonensis	
B3RAT1	G3	VII	Cupriavidus taiwanensis	
C9Y763	G3	VII	Curvibacter putative	
B8F8T5	G3	VII	Desulfatibacillum alkenivorans	
B8FJB3	G3	VII	Desulfatibacillum alkenivorans	
A8ZUE2	G3	VII	Desulfococcus oleovorans	
C6CDQ7	G3	VII	Dickeya dadantii	
Q0RJV2	G3	VII	Frankia alni	
C6JND5	G3	VII	Fusobacterium varium	
C6JR85	G3	VII	Fusobacterium varium	
A4IRU8	G3	VII	Geobacillus thermodenitrificans	
D0LLX8	G3	VII	Haliangium ochraceum	
A3YFY0	G3	VII	Marinomonas sp	
B8IC55	G3	VII	Methylobacterium nodulans	
B1MCN0	G3	VII	Mycobacterium abscessus	

Table S2.2. (continuation)

UniProt	Group [2]	Cluster	Species	EC Number
A1KJD5	G3	VII	Mycobacterium bovis	
Q7TZQ6	G3	VII	Mycobacterium bovis	
A0R5Z8	G3	VII	Mycobacterium smegmatis	
A2VIK1	G3	VII	Mycobacterium tuberculosis	
A5U377	G3	VII	Mycobacterium tuberculosis	
P71976	G3	VII	Mycobacterium tuberculosis	
Q3IQN5	G3	VII	Natronomonas pharaonis	
Q5YUM6	G3	VII	Nocardia farcinica	
A3UET5	G3	VII	Oceanicaulis sp	
A6G0K8	G3	VII	Plesiocystis pacifica	
Q12GL9	G3	VII	Polaromonas sp	
Q1LM37	G3	VII	Ralstonia metallidurans	
Q98JP6	G3	VII	Rhizobium loti	
Q92ZZ8	G3	VII	Rhizobium meliloti	
C0ZQT9	G3	VII	Rhodococcus erythropolis	
C1AXA8	G3	VII	Rhodococcus opacus	
B3QEN1	G3	VII	Rhodopseudomonas palustris	
Q07PE0	G3	VII	Rhodopseudomonas palustris	
Q138Q2	G3	VII	Rhodopseudomonas palustris	
Q212E1	G3	VII	Rhodopseudomonas palustris	
Q21BM8	G3	VII	Rhodopseudomonas palustris	
Q2IV02	G3	VII	Rhodopseudomonas palustris	
Q6N7W3	G3	VII	Rhodopseudomonas palustris	
B6IX62	G3	VII	Rhodospirillum centenum	
A3VWM0	G3	VII	Roseovarius sp	
A4FFZ2	G3	VII	Saccharopolyspora erythraea	
A9FJC5	G3	VII	Sorangium cellulosum	
Q7WSS9	G3	VII	Sphingomonas paucimobilis	
A5V776	G3	VII	Sphingomonas wittichii	
A5VGU8	G3	VII	Sphingomonas wittichii	
D2AVU6	G3	VII	Streptosporangium roseum	
D2B8M7	G3	VII	Streptosporangium roseum	
C5CW39	G3	VII	Variovorax paradoxus	
C5CW93	G3	VII	Variovorax paradoxus	
A1WJ40	G3	VII	Verminephrobacter eiseniae	
A7IPL4	G3	VII	Xanthobacter autotrophicus	
C4SJE4	G3	VII	Yersinia frederiksenii	
A4KHN0	G3	VII		
A4LG88	G3	VII		
A5WN37	G3	VII		
A8KE19	G3	VII		
B2HAS0	G3	VII		
B4BPT1	G3	VII		
B5KCM1	G3	VII		
B7CQ94	G3	VII		
C0Y7A1	G3	VII		
C1ANZ1	G3	VII		
C1M917	G3	VII		
C1XV06	G3	VII		
C1YG16	G3	VII		
C5ZQS7	G3	VII		
C6DRV4	G3	VII		
C6JPM7	G3	VII		
C6UAA0	G3	VII		
C8SWR6	G3	VII		
D2LG99	G3	VII		

Table S2.2. (continuation)

UniProt	Group [2]	Cluster	Species	EC Number
D2M5U7	G3	VII		
A8TRV9	G4	IV	alpha proteobacterium	
Q89J23	G4	IV	Bradyrhizobium japonicum	
B2JH31	G4	IV	Burkholderia phymatum	
B2T8L3	G4	IV	Burkholderia phytofirmans	
B5WB71	G4	IV	Burkholderia sp	
D0D2V4	G4	IV	Citreicella sp	
Q0KAA1	G4	IV	Cupriavidus necator	
Q46R50	G4	IV	Cupriavidus pinatubonensis	
Q471D6	G4	IV	Cupriavidus pinatubonensis	
A8ZUA9	G4	IV	Desulfococcus oleovorans	
A8LPE3	G4	IV	Dinoroseobacter shibae	
Q28JN0	G4	IV	Jannaschia sp	
A0P1F4	G4	IV	Labrenzia aggregata	
B9QST2	G4	IV	Labrenzia alexandrii	
B9QUK9	G4	IV	Labrenzia alexandrii	
A3V8D0	G4	IV	Loktanella vestfoldensis	
A3V8P7	G4	IV	Loktanella vestfoldensis	
Q6SJD0	G4	IV	marine alpha	
D1YV31	G4	IV	Methanocella paludicola	
A9W2E9	G4	IV	Methylobacterium extorquens	
A9W915	G4	IV	Methylobacterium extorquens	
B7KSR7	G4	IV	Methylobacterium extorquens	
B7KUX6	G4	IV	Methylobacterium extorquens	
C5AU40	G4	IV	Methylobacterium extorquens	
C5AXX3	G4	IV	Methylobacterium extorquens	
C7C936	G4	IV	Methylobacterium extorquens	
C7CJV2	G4	IV	Methylobacterium extorquens	
B8IBD6	G4	IV	Methylobacterium nodulans	
B8IUK2	G4	IV	Methylobacterium nodulans	
B1Z9T7	G4	IV	Methylobacterium populi	
B1LXE4	G4	IV	Methylobacterium radiotolerans	
B0U8T4	G4	IV	Methylobacterium sp	
B0UI16	G4	IV	Methylobacterium sp	
D2V036	G4	IV	Naegleria gruberi	
A9EEW9	G4	IV	Oceanibulbus indolifex	
A9EFA6	G4	IV	Oceanibulbus indolifex	
A3TVG2	G4	IV	Oceanicola batsensis	
Q2CGV3	G4	IV	Oceanicola granulosus	
A1B7S1	G4	IV	Paracoccus denitrificans	
Q0FH38	G4	IV	Pelagibaca bermudensis	
Q3A0G5	G4	IV	Pelobacter carbinolicus	
A4WY25	G4	IV	Rhodobacter sphaeroides	
A3JVW9	G4	IV	Rhodobacteraceae bacterium	
B6B2N6	G4	IV	Rhodobacteraceae bacterium	
B9NWJ0	G4	IV	Rhodobacteraceae bacterium	
B6B8L6	G4	IV	Rhodobacterales bacterium	
B6BG68	G4	IV	Rhodobacterales bacterium	
A4CMM5	G4	IV	Robiginitalea biformata	
Q165Z7	G4	IV	Roseobacter denitrificans	
A3X399	G4	IV	Roseobacter sp	
A3XE58	G4	IV	Roseobacter sp	
A4EGL1	G4	IV	Roseobacter sp	
A4EQI8	G4	IV	Roseobacter sp	
B7RK19	G4	IV	Roseobacter sp	
A6FU67	G4	IV	Roseobacter sp	6.4.1.2

Table S2.2. (continuation)

UniProt	Group [2]	Cluster	Species	EC Number
A3SR74	G4	IV	Roseovarius nubinhibens	
A3VWL5	G4	IV	Roseovarius sp	
A3VZD5	G4	IV	Roseovarius sp	
A3W8S2	G4	IV	Roseovarius sp	
A6E2X7	G4	IV	Roseovarius sp	
Q5LLI5	G4	IV	Ruegeria pomeroyi	
Q6SJB6	G4	IV	Ruegeria pomeroyi	
B7QSI3	G4	IV	Ruegeria sp	
Q1GJ77	G4	IV	Ruegeria sp	
A3JX59	G4	IV	Sagittula stellata	
Q7BLA5	G4	IV	Sagittula stellata	
C9CZ86	G4	IV	Silicibacter sp	
A3T0P7	G4	IV	Sulfitobacter sp	
A1HTD8	G4	IV	Thermosinus carboxydivorans	
A3SES5	G4	IV		
A9ET73	G4	IV		
A9GL07	G4	IV		
A9GTK1	G4	IV		
D1TCY9	G4	IV		
B3QMV9	G4	VII	Chlorobaculum parvum	
Q3ARY1	G4	VII	Chlorobium chlorochromatii	
B3EN00	G4	VII	Chlorobium phaeobacteroides	
B6BLL4	G4	VII	Sulfurimonas gotlandica	
B9L133	G4	VII	Thermomicrobium roseum	
B9JK98	G5	VI	Agrobacterium radiobacter	
Q7D3B4	G5	VI	Agrobacterium tumefaciens	
A8TMK9	G5	VI	alpha proteobacterium	
A8TST9	G5	VI	alpha proteobacterium	
A7GLV3	G5	VI	Bacillus cereus	
Q4V183	G5	VI	Bacillus cereus	
B1F8D7	G5	VI	Burkholderia ambifaria	
B1SWX4	G5	VI	Burkholderia ambifaria	
B1YZM5	G5	VI	Burkholderia ambifaria	
Q0B764	G5	VI	Burkholderia ambifaria	
A0B2A9	G5	VI	Burkholderia cenocepacia	
A2W3H3	G5	VI	Burkholderia cenocepacia	
B1K7C2	G5	VI	Burkholderia cenocepacia	
B4EH10	G5	VI	Burkholderia cenocepacia	
Q1BQ94	G5	VI	Burkholderia cenocepacia	
A2WG58	G5	VI	Burkholderia dolosa	
C5AMJ8	G5	VI	Burkholderia glumae	
B1G0K3	G5	VI	Burkholderia graminis	
A2RYM5	G5	VI	Burkholderia mallei	
C4B1L0	G5	VI	Burkholderia mallei	
Q62DG5	G5	VI	Burkholderia mallei	
A9AN52	G5	VI	Burkholderia multivorans	
B9AYI2	G5	VI	Burkholderia multivorans	
B9BRT6	G5	VI	Burkholderia multivorans	
B2JS64	G5	VI	Burkholderia phymatum	
B2TGS1	G5	VI	Burkholderia phytofirmans	
A3NHM9	G5	VI	Burkholderia pseudomallei	
A3P387	G5	VI	Burkholderia pseudomallei	
A8EJY3	G5	VI	Burkholderia pseudomallei	
B1HM01	G5	VI	Burkholderia pseudomallei	
C4I1B5	G5	VI	Burkholderia pseudomallei	
Q3JGM3	G5	VI	Burkholderia pseudomallei	

Table S2.2. (continuation)

UniProt	Group [2]	Cluster	Species	EC Number
Q63MT1	G5	VI	Burkholderia pseudomallei	
Q399Y5	G5	VI	Burkholderia sp	
Q2T457	G5	VI	Burkholderia thailandensis	
A4JJC8	G5	VI	Burkholderia vietnamiensis	
Q13LB2	G5	VI	Burkholderia xenovorans	
B6BQJ5	G5	VI	Candidatus Pelagibacter	
Q1QSP0	G5	VI	Chromohalobacter salexigens	
D0DBQ6	G5	VI	Citreicella sp	
C8X021	G5	VI	Desulfohalobium retbaense	
A9D0S4	G5	VI	Hoeflea phototrophica	
Q28KM0	G5	VI	Jannaschia sp	
A0NVX0	G5	VI	Labrenzia aggregata	
A0NZH0	G5	VI	Labrenzia aggregata	
B9QY13	G5	VI	Labrenzia alexandrii	
A3V8F2	G5	VI	Loktanella vestfoldensis	
A0YBG6	G5	VI	marine gamma	
A0Z403	G5	VI	marine gamma	
B7S2Z2	G5	VI	marine gamma	
A3YAS6	G5	VI	Marinomonas sp	
Q982J9	G5	VI	Mesorhizobium loti	
B8I9M0	G5	VI	Methylobacterium nodulans	
B1LTI6	G5	VI	Methylobacterium radiotolerans	
B0UNX4	G5	VI	Methylobacterium sp	
B2A0X8	G5	VI	Natranaerobius thermophilus	
A9DSW8	G5	VI	Oceanibulbus indolifex	
A3UHG3	G5	VI	Oceanicaulis sp	
A3U1X9	G5	VI	Oceanicola batsensis	
Q8CUW1	G5	VI	Oceanobacillus iheyensis	
A6X326	G5	VI	Ochrobactrum anthropi	
C4WM76	G5	VI	Ochrobactrum intermedium	
A1BC87	G5	VI	Paracoccus denitrificans	
Q0FWY7	G5	VI	Pelagibaca bermudensis	
B9Z8X9	G5	VI	Pseudogulbenkiania ferrooxidans	
A6VEJ9	G5	VI	Pseudomonas aeruginosa	
Q9HTH7	G5	VI	Pseudomonas aeruginosa	
Q1I3H7	G5	VI	Pseudomonas entomophila	
C3K3C1	G5	VI	Pseudomonas fluorescens	
Q3K5I6	G5	VI	Pseudomonas fluorescens	
Q4K4M2	G5	VI	Pseudomonas fluorescens	
A4XPK4	G5	VI	Pseudomonas mendocina	
A5VX86	G5	VI	Pseudomonas putida	
B0KI54	G5	VI	Pseudomonas putida	
B1JEE5	G5	VI	Pseudomonas putida	
Q88R31	G5	VI	Pseudomonas putida	
Q4ZSC1	G5	VI	Pseudomonas syringae	
Q2K1G9	G5	VI	Rhizobium etli	
B6A2S5	G5	VI	Rhizobium leguminosarum	
C6BAS3	G5	VI	Rhizobium leguminosarum	
Q1M7D2	G5	VI	Rhizobium leguminosarum	
Q98CR5	G5	VI	Rhizobium loti	
Q92NF6	G5	VI	Rhizobium meliloti	
C3MES7	G5	VI	Rhizobium sp	
C8RYA8	G5	VI	Rhodobacter sp	
A3JP16	G5	VI	Rhodobacteraceae bacterium	
B6AXB7	G5	VI	Rhodobacteraceae bacterium	
B6AZS8	G5	VI	Rhodobacteraceae bacterium	

Table S2.2. (continuation)

UniProt	Group [2]	Cluster	Species	EC Number
B9NQN4	G5	VI	Rhodobacteraceae bacterium	
B9NWP8	G5	VI	Rhodobacteraceae bacterium	
B6B9X9	G5	VI	Rhodobacterales bacterium	
Q0FAD0	G5	VI	Rhodobacterales bacterium	
Q166L4	G5	VI	Roseobacter denitrificans	
A3X579	G5	VI	Roseobacter sp	
A4EIR3	G5	VI	Roseobacter sp	
A4EUB3	G5	VI	Roseobacter sp	
A6FTU0	G5	VI	Roseobacter sp	
B7RH08	G5	VI	Roseobacter sp	
A3SRB7	G5	VI	Roseovarius nubinhibens	
A3VZI9	G5	VI	Roseovarius sp	
A6DWV1	G5	VI	Roseovarius sp	
D0CYW1	G5	VI	Ruegeria lacuscaerulensis	
Q5LPZ3	G5	VI	Ruegeria pomeroyi	
Q5LTH9	G5	VI	Ruegeria pomeroyi	
B7QWW3	G5	VI	Ruegeria sp	
Q1GEJ6	G5	VI	Ruegeria sp	
Q1GM47	G5	VI	Ruegeria sp	
C7MT24	G5	VI	Saccharomonospora viridis	
A4FGG1	G5	VI	Saccharopolyspora erythraea	
A3JZD9	G5	VI	Sagittula stellata	
A3JZU9	G5	VI	Sagittula stellata	
C9CVI1	G5	VI	Silicibacter sp	
A6UBH6	G5	VI	Sinorhizobium medicae	
B9DLH5	G5	VI	Staphylococcus carnosus	
Q5HKI4	G5	VI	Staphylococcus epidermidis	
Q8CTX4	G5	VI	Staphylococcus epidermidis	
Q4A025	G5	VI	Staphylococcus saprophyticus	
Q93RX4	G5	VI	Streptomyces coelicolor	
B1VLT8	G5	VI	Streptomyces griseus	
A3T1Q4	G5	VI	Sulfitobacter sp	
C7D9Z8	G5	VI	Thalassobium sp	
A1WPW8	G5	VI	Verminephrobacter eiseniae	
A1UWE2	G5	VI		
A3L1N9	G5	VI		
A3LFZ8	G5	VI		
A3MFU8	G5	VI		
A3S779	G5	VI		
A4LV75	G5	VI		
A5JAI3	G5	VI		
A5XWG8	G5	VI		
A8KI11	G5	VI		
A9F664	G5	VI		
A9GCI6	G5	VI		
A9HSN9	G5	VI		
A9K2V9	G5	VI		
B2H4E4	G5	VI		
B5JAA6	G5	VI		
B5KAL9	G5	VI		
B7CZZ8	G5	VI		
B7V5S9	G5	VI		
B9CDN3	G5	VI		
C0Y268	G5	VI		
C1YRM0	G5	VI		
C5NJG1	G5	VI		

Table S2.2. (continuation)

UniProt	Group [2]	Cluster	Species	EC Number
C5QB57	G5	VI		
C5QZ78	G5	VI		
C5ZRC4	G5	VI		
C6U9A9	G5	VI		
C8SII8	G5	VI		
D1TK71	G5	VI		
D1UCL6	G5	VI		
D1WPU5	G5	VI		
D1WSZ2	G5	VI		
Q02DX5	G5	VI		
Q89J26	G5	VII	Bradyrhizobium japonicum	
A0R3W1	G5	VII	Mycobacterium smegmatis	
C5T2N0	G6	III	Acidovorax delafieldii	
A8ID44	G6	III	Azorhizobium caulinodans	
Q7WJQ6	G6	III	Bordetella bronchiseptica	
Q7WAK5	G6	III	Bordetella parapertussis	
B7WVD7	G6	III	Comamonas testosteroni	
D0J611	G6	III	Comamonas testosteroni	
Q0K0N8	G6	III	Cupriavidus necator	
Q46U71	G6	III	Cupriavidus pinatubonensis	
B3RAY2	G6	III	Cupriavidus taiwanensis	
A9C1D2	G6	III	Delftia acidovorans	
D0ICR3	G6	III	Grimontia hollisae	
A0NXJ1	G6	III	Labrenzia aggregata	
B9QXY1	G6	III	Labrenzia alexandrii	
A3Y780	G6	III	Marinomonas sp	
A3VDL2	G6	III	Maritimibacter alkaliphilus	
A3VFQ2	G6	III	Maritimibacter alkaliphilus	
Q11LZ6	G6	III	Mesorhizobium sp	
A9W253	G6	III	Methylobacterium extorquens	
B7KRT6	G6	III	Methylobacterium extorquens	
C5AX97	G6	III	Methylobacterium extorquens	
C7CIL6	G6	III	Methylobacterium extorquens	
B8IJY2	G6	III	Methylobacterium nodulans	
B1ZC36	G6	III	Methylobacterium populi	
B1M737	G6	III	Methylobacterium radiotolerans	
B0UPA5	G6	III	Methylobacterium sp	
Q2BIH9	G6	III	Neptuniibacter caesariensis	
A9E7R3	G6	III	Oceanibulbus indolifex	
B6QY15	G6	III	Pseudovibrio sp	
Q1LRJ5	G6	III	Ralstonia metallidurans	
B2U785	G6	III	Ralstonia pickettii	
C6BI12	G6	III	Ralstonia pickettii	
A3RPW8	G6	III	Ralstonia solanacearum	
Q8Y397	G6	III	Ralstonia solanacearum	
Q92WR4	G6	III	Rhizobium meliloti	
A3PQF6	G6	III	Rhodobacter sphaeroides	
A4WYT8	G6	III	Rhodobacter sphaeroides	
B9KVB7	G6	III	Rhodobacter sphaeroides	
Q3IVM4	G6	III	Rhodobacter sphaeroides	
A3JLT5	G6	III	Rhodobacteraceae bacterium	
B9NSV7	G6	III	Rhodobacteraceae bacterium	
B6BCH2	G6	III	Rhodobacterales bacterium	
A3XEY4	G6	III	Roseobacter sp	
A4ENU1	G6	III	Roseobacter sp	
A6FTT2	G6	III	Roseobacter sp	

Table S2.2. (continuation)

UniProt	Group [2]	Cluster	Species	EC Number
A3SIM5	G6	III	Roseovarius nubinhibens	
D0CS11	G6	III	Ruegeria lacuscaerulensis	
Q5LTL4	G6	III	Ruegeria pomeroyi	
B7QYT4	G6	III	Ruegeria sp	
Q1GKC3	G6	III	Ruegeria sp	
C9CTV8	G6	III	Silicibacter sp	
A6UG88	G6	III	Sinorhizobium medicae	
A3T0G8	G6	III	Sulfitobacter sp	
A1WF90	G6	III	Verminephrobacter eiseniae	
D0WUA2	G6	III	Vibrio alginolyticus	
A6AQ57	G6	III	Vibrio campbellii	
A7MZD9	G6	III	Vibrio campbellii	
D0XDG1	G6	III	Vibrio harveyi	
A6B8E5	G6	III	Vibrio parahaemolyticus	
Q87RZ3	G6	III	Vibrio parahaemolyticus	
A6CXL1	G6	III	Vibrio shilonii	
A7K162	G6	III	Vibrio sp	
A7IMM6	G6	III	Xanthobacter autotrophicus	
A3SEJ8	G6	III		
A9EIV2	G6	III		
A9FWD6	G6	III		
B5S7G9	G6	III		
B5SEV3	G6	III		
C8SN56	G6	III		
C9NT32	G6	III		
Q1VAE1	G6	III		
A2RWD4	G7	I	Burkholderia mallei	
A3MEC9	G7	I	Burkholderia mallei	
C4B2Y3	G7	I	Burkholderia mallei	
Q62CH9	G7	I	Burkholderia mallei	
A3NKQ2	G7	I	Burkholderia pseudomallei	
A3P6B3	G7	I	Burkholderia pseudomallei	
A8EQ62	G7	I	Burkholderia pseudomallei	
B1H8Q3	G7	I	Burkholderia pseudomallei	
C4I4V6	G7	I	Burkholderia pseudomallei	
Q3JLL6	G7	I	Burkholderia pseudomallei	
Q63KK6	G7	I	Burkholderia pseudomallei	
Q2T6D1	G7	I	Burkholderia thailandensis	
A1UVS7	G7	I		
A4LQX4	G7	I		
A5J2F2	G7	I		
A5TFX4	G7	I		
A8KFW7	G7	I		
A9K371	G7	I		
B2H8J8	G7	I		
B7CZA7	G7	I		
C0YCX5	G7	I		
C5NA15	G7	I		
C5ZPT2	G7	I		
C6U4Z0	G7	I		
D0RZF2	G7	II	Acinetobacter calcoaceticus	
B1T2C9	G7	II	Burkholderia ambifaria	
B1YQF5	G7	II	Burkholderia ambifaria	
Q0BFC8	G7	II	Burkholderia ambifaria	
B4E9D8	G7	II	Burkholderia cenocepacia	
A2RZN4	G7	II	Burkholderia mallei	

Table S2.2. (continuation)

UniProt	Group [2]	Cluster	Species	EC Number
C4AV33	G7	II	Burkholderia mallei	
Q629S5	G7	II	Burkholderia mallei	
B2JUK7	G7	II	Burkholderia phymatum	
B2JWR2	G7	II	Burkholderia phymatum	
A3P9X0	G7	II	Burkholderia pseudomallei	
A8EAR1	G7	II	Burkholderia pseudomallei	
B1H4A2	G7	II	Burkholderia pseudomallei	
C4I4E8	G7	II	Burkholderia pseudomallei	
Q3JIK5	G7	II	Burkholderia pseudomallei	
Q63HX4	G7	II	Burkholderia pseudomallei	
B5WR28	G7	II	Burkholderia sp	
Q39GH9	G7	II	Burkholderia sp	
B7WRE0	G7	II	Comamonas testosteroni	
B7WWG9	G7	II	Comamonas testosteroni	
B7X4J4	G7	II	Comamonas testosteroni	
D0IUT6	G7	II	Comamonas testosteroni	
D0J0F6	G7	II	Comamonas testosteroni	
D0J581	G7	II	Comamonas testosteroni	
Q0JY66	G7	II	Cupriavidus necator	
Q0K0S2	G7	II	Cupriavidus necator	
Q0K2F1	G7	II	Cupriavidus necator	
Q0K2H8	G7	II	Cupriavidus necator	
Q46R35	G7	II	Cupriavidus pinatubonensis	
Q46S21	G7	II	Cupriavidus pinatubonensis	
Q46UC5	G7	II	Cupriavidus pinatubonensis	
Q46UI4	G7	II	Cupriavidus pinatubonensis	
Q46ZC6	G7	II	Cupriavidus pinatubonensis	
Q471H0	G7	II	Cupriavidus pinatubonensis	
Q477N1	G7	II	Cupriavidus pinatubonensis	
B3R962	G7	II	Cupriavidus taiwanensis	
B3RD55	G7	II	Cupriavidus taiwanensis	
A9BT82	G7	II	Delftia acidovorans	
A9BT86	G7	II	Delftia acidovorans	
D3CVE6	G7	II	Frankia sp	
B1Y2U8	G7	II	Leptothrix cholodnii	
A5W3U9	G7	II	Pseudomonas putida	
B0KU49	G7	II	Pseudomonas putida	
Q1LCI1	G7	II	Ralstonia metallidurans	
Q1LCZ7	G7	II	Ralstonia metallidurans	
Q1LD38	G7	II	Ralstonia metallidurans	
Q1LED3	G7	II	Ralstonia metallidurans	
Q1LML6	G7	II	Ralstonia metallidurans	
B2UHA4	G7	II	Ralstonia pickettii	
C6BMA3	G7	II	Ralstonia pickettii	
C0ZN26	G7	II	Rhodococcus erythropolis	
C3JPZ1	G7	II	Rhodococcus erythropolis	
C1B881	G7	II	Rhodococcus opacus	
Q0SA52	G7	II	Rhodococcus sp	
B0B4Y6	G7	II	Streptomyces collinus	
A1UXG3	G7	II		
A3MGW9	G7	II		
A4LP04	G7	II		
A5JBH8	G7	II		
A5XMK5	G7	II		
A8KGY0	G7	II		
A9K3M0	G7	II		

Table S2.2. (continuation)

UniProt	Group [2]	Cluster	Species	EC Number
B2H4R7	G7	II		
B5HBU6	G7	II		
B7CWJ1	G7	II		
C0YDK7	G7	II		
C5NK36	G7	II		
C5ZNX8	G7	II		
C6U4K2	G7	II		
D1TEB1	G7	II		
D1T2E2	G7	V		
A8LEN0	G7	VI	Frankia sp	
C6WPT3	G7	VII	Actinosynnema mirum	
A1R3D3	G7	VII	Arthrobacter aurescens	
C5AJX5	G7	VII	Burkholderia glumae	
A8RYF1	G7	VII	Clostridium bolteae	
A4A7D0	G7	VII	Congregibacter litoralis	
C1D3M5	G7	VII	Deinococcus deserti	
Q9RY98	G7	VII	Deinococcus radiodurans	
A8LMB1	G7	VII	Dinoroseobacter shibae	
B8KKC5	G7	VII	gamma proteobacterium	
D2S5A9	G7	VII	Geodermatophilus obscurus	
Q28MR0	G7	VII	Jannaschia sp	
B7RVE3	G7	VII	marine gamma	
B8IMG7	G7	VII	Methylobacterium nodulans	
B0U7K6	G7	VII	Methylobacterium sp	
A4T724	G7	VII	Mycobacterium gilvum	
A1ULF3	G7	VII	Mycobacterium sp	
A3Q5V2	G7	VII	Mycobacterium sp	
Q1B3P7	G7	VII	Mycobacterium sp	
A1TEW9	G7	VII	Mycobacterium vanbaalenii	
C8XE64	G7	VII	Nakamurella multipartita	
A6VAG3	G7	VII	Pseudomonas aeruginosa	
Q02HW6	G7	VII	Pseudomonas aeruginosa	
Q9I5C7	G7	VII	Pseudomonas aeruginosa	
C6AYH9	G7	VII	Rhizobium leguminosarum	
Q1MGF1	G7	VII	Rhizobium leguminosarum	
C3MI84	G7	VII	Rhizobium sp	
C3MI88	G7	VII	Rhizobium sp	
C0ZQU4	G7	VII	Rhodococcus erythropolis	
A4FQE8	G7	VII	Saccharopolyspora erythraea	
A5V766	G7	VII	Sphingomonas wittichii	
Q9S256	G7	VII	Streptomyces coelicolor	
B4VG76	G7	VII	Streptomyces sp	
C6KU05	G7	VII	uncultured bacterium	
D2DIR0	G7	VII	uncultured marine	
A3L579	G7	VII		
A3LM24	G7	VII		
B4B7L9	G7	VII		
B5KEI7	G7	VII		
B5KEK6	G7	VII		
B7UYE7	G7	VII		
D1SAE2	G7	VII		
D1XUM6	G7	VII		
D3C665	G7	VII		

Case study III: protein kinases

Of the 3,087 proteins in this family, 2,044 are labeled as Ser/Thr kinases and 1,043 as Tyr kinases, of which 235 are Epidermal Growth Factor Receptors (EGFRs) according to the labels employed in [1]. Table S2.3 lists the protein set, the corresponding subfamily labels, and the clusters they were placed in by the proposed framework when considering two, three and seven clusters, as well as the source organisms and existing EC number annotations according to UniProt.

Table S2.3. List of proteins in the protein kinase family.

UniProt	Label [1]	Two	Three	Seven	Species	EC Number
Q17D67	EGFR	II	I	IV	Aedes aegypti	2.7.10.1
Q7PPN5	EGFR	II	I	IV	Anopheles gambiae	2.7.10.1
Q9BIH9	EGFR	II	I	IV	Anopheles gambiae	2.7.10.1
Q64895	EGFR	II	I	IV	Avian erythroblastosis	
Q85468	EGFR	II	I	IV	Avian erythroblastosis	
Q86712	EGFR	II	I	IV	Avian rous-associated	
Q86714	EGFR	II	I	IV	Avian rous-associated	
Q4H3N2	EGFR	II	I	IV	Ciona intestinalis	
Q5EBY4	EGFR	II	I	IV	Danio rerio	
Q6VQA3	EGFR	II	I	IV	Danio rerio	
Q7SZF7	EGFR	II	I	IV	Danio rerio	
Q8MLW0	EGFR	II	I	IV	Drosophila melanogaster	2.7.10.-
Q6SA99	EGFR	II	I	IV	Drosophila melanogaster	2.7.10.1
Q6SAA0	EGFR	II	I	IV	Drosophila melanogaster	2.7.10.1
Q6SAA1	EGFR	II	I	IV	Drosophila melanogaster	2.7.10.1
Q6SAA2	EGFR	II	I	IV	Drosophila melanogaster	2.7.10.1
Q6SAA3	EGFR	II	I	IV	Drosophila melanogaster	2.7.10.1
Q6SAA4	EGFR	II	I	IV	Drosophila melanogaster	2.7.10.1
Q6SAA5	EGFR	II	I	IV	Drosophila melanogaster	2.7.10.1
Q6SAA7	EGFR	II	I	IV	Drosophila melanogaster	2.7.10.1
Q6SAA8	EGFR	II	I	IV	Drosophila melanogaster	2.7.10.1
Q6SAA9	EGFR	II	I	IV	Drosophila melanogaster	2.7.10.1
Q6SAB0	EGFR	II	I	IV	Drosophila melanogaster	2.7.10.1
Q6SAB3	EGFR	II	I	IV	Drosophila melanogaster	2.7.10.1
Q6SAB4	EGFR	II	I	IV	Drosophila melanogaster	2.7.10.1
Q6SAB6	EGFR	II	I	IV	Drosophila melanogaster	2.7.10.1
Q6SAB8	EGFR	II	I	IV	Drosophila melanogaster	2.7.10.1
Q6SAB9	EGFR	II	I	IV	Drosophila melanogaster	2.7.10.1
Q6SAC0	EGFR	II	I	IV	Drosophila melanogaster	2.7.10.1
Q6SAC1	EGFR	II	I	IV	Drosophila melanogaster	2.7.10.1
Q6SAC2	EGFR	II	I	IV	Drosophila melanogaster	2.7.10.1
Q6SAC4	EGFR	II	I	IV	Drosophila melanogaster	2.7.10.1
Q6SAC6	EGFR	II	I	IV	Drosophila melanogaster	2.7.10.1
Q6SAC7	EGFR	II	I	IV	Drosophila melanogaster	2.7.10.1
Q6SAC8	EGFR	II	I	IV	Drosophila melanogaster	2.7.10.1
Q6SAC9	EGFR	II	I	IV	Drosophila melanogaster	2.7.10.1
Q6SAD0	EGFR	II	I	IV	Drosophila melanogaster	2.7.10.1
Q6SAD1	EGFR	II	I	IV	Drosophila melanogaster	2.7.10.1
Q6SAD3	EGFR	II	I	IV	Drosophila melanogaster	2.7.10.1
Q6SAD5	EGFR	II	I	IV	Drosophila melanogaster	2.7.10.1
Q6SAD7	EGFR	II	I	IV	Drosophila melanogaster	2.7.10.1
Q6SAD8	EGFR	II	I	IV	Drosophila melanogaster	2.7.10.1
Q6SAD9	EGFR	II	I	IV	Drosophila melanogaster	2.7.10.1
Q6SAE0	EGFR	II	I	IV	Drosophila melanogaster	2.7.10.1
Q6SAE1	EGFR	II	I	IV	Drosophila melanogaster	2.7.10.1
Q6SAE2	EGFR	II	I	IV	Drosophila melanogaster	2.7.10.1
Q6SAE3	EGFR	II	I	IV	Drosophila melanogaster	2.7.10.1
Q6SAE4	EGFR	II	I	IV	Drosophila melanogaster	2.7.10.1

Table S2.3. (continuation)

UniProt	Label [1]	Two	Three	Seven	Species	EC Number
Q6SAE6	EGFR	II	I	IV	Drosophila melanogaster	2.7.10.1
Q6SAE8	EGFR	II	I	IV	Drosophila melanogaster	2.7.10.1
Q6SAF1	EGFR	II	I	IV	Drosophila melanogaster	2.7.10.1
Q6SAF2	EGFR	II	I	IV	Drosophila melanogaster	2.7.10.1
Q6SAF5	EGFR	II	I	IV	Drosophila melanogaster	2.7.10.1
Q6SAF6	EGFR	II	I	IV	Drosophila melanogaster	2.7.10.1
Q6SAG0	EGFR	II	I	IV	Drosophila melanogaster	2.7.10.1
Q6SAG1	EGFR	II	I	IV	Drosophila melanogaster	2.7.10.1
Q6SAG2	EGFR	II	I	IV	Drosophila melanogaster	2.7.10.1
Q6SAG3	EGFR	II	I	IV	Drosophila melanogaster	2.7.10.1
Q6SAG5	EGFR	II	I	IV	Drosophila melanogaster	2.7.10.1
Q6SAG6	EGFR	II	I	IV	Drosophila melanogaster	2.7.10.1
Q6SAG8	EGFR	II	I	IV	Drosophila melanogaster	2.7.10.1
Q6SAG9	EGFR	II	I	IV	Drosophila melanogaster	2.7.10.1
Q6SAH0	EGFR	II	I	IV	Drosophila melanogaster	2.7.10.1
Q6SAH1	EGFR	II	I	IV	Drosophila melanogaster	2.7.10.1
Q6SAH2	EGFR	II	I	IV	Drosophila melanogaster	2.7.10.1
Q6SAH4	EGFR	II	I	IV	Drosophila melanogaster	2.7.10.1
Q6SAH5	EGFR	II	I	IV	Drosophila melanogaster	2.7.10.1
Q6SAH7	EGFR	II	I	IV	Drosophila melanogaster	2.7.10.1
Q6SAH8	EGFR	II	I	IV	Drosophila melanogaster	2.7.10.1
Q6SAH9	EGFR	II	I	IV	Drosophila melanogaster	2.7.10.1
Q6SAI2	EGFR	II	I	IV	Drosophila melanogaster	2.7.10.1
Q6SAI4	EGFR	II	I	IV	Drosophila melanogaster	2.7.10.1
Q6SAI5	EGFR	II	I	IV	Drosophila melanogaster	2.7.10.1
Q6SAI6	EGFR	II	I	IV	Drosophila melanogaster	2.7.10.1
Q6SAI7	EGFR	II	I	IV	Drosophila melanogaster	2.7.10.1
Q6SAI8	EGFR	II	I	IV	Drosophila melanogaster	2.7.10.1
Q6SAI9	EGFR	II	I	IV	Drosophila melanogaster	2.7.10.1
Q6SAJ0	EGFR	II	I	IV	Drosophila melanogaster	2.7.10.1
Q6SAJ2	EGFR	II	I	IV	Drosophila melanogaster	2.7.10.1
Q6SAJ3	EGFR	II	I	IV	Drosophila melanogaster	2.7.10.1
Q6SAJ5	EGFR	II	I	IV	Drosophila melanogaster	2.7.10.1
Q6SAJ6	EGFR	II	I	IV	Drosophila melanogaster	2.7.10.1
Q6SAJ7	EGFR	II	I	IV	Drosophila melanogaster	2.7.10.1
Q6SAJ8	EGFR	II	I	IV	Drosophila melanogaster	2.7.10.1
Q6SAJ9	EGFR	II	I	IV	Drosophila melanogaster	2.7.10.1
Q6SAK0	EGFR	II	I	IV	Drosophila melanogaster	2.7.10.1
Q6SAK1	EGFR	II	I	IV	Drosophila melanogaster	2.7.10.1
Q6SAK2	EGFR	II	I	IV	Drosophila melanogaster	2.7.10.1
Q6SAK3	EGFR	II	I	IV	Drosophila melanogaster	2.7.10.1
Q6SAK4	EGFR	II	I	IV	Drosophila melanogaster	2.7.10.1
Q6SAK6	EGFR	II	I	IV	Drosophila melanogaster	2.7.10.1
Q6SAK9	EGFR	II	I	IV	Drosophila melanogaster	2.7.10.1
Q6SAL0	EGFR	II	I	IV	Drosophila melanogaster	2.7.10.1
Q6SAL2	EGFR	II	I	IV	Drosophila melanogaster	2.7.10.1
Q6SAL3	EGFR	II	I	IV	Drosophila melanogaster	2.7.10.1
Q6SAL4	EGFR	II	I	IV	Drosophila melanogaster	2.7.10.1
Q6SAL5	EGFR	II	I	IV	Drosophila melanogaster	2.7.10.1
Q6SAL6	EGFR	II	I	IV	Drosophila melanogaster	2.7.10.1
Q6SAL7	EGFR	II	I	IV	Drosophila melanogaster	2.7.10.1
Q6SAL8	EGFR	II	I	IV	Drosophila melanogaster	2.7.10.1
Q6SAL9	EGFR	II	I	IV	Drosophila melanogaster	2.7.10.1
Q6SAM0	EGFR	II	I	IV	Drosophila melanogaster	2.7.10.1
Q6SAM1	EGFR	II	I	IV	Drosophila melanogaster	2.7.10.1
Q6SAM2	EGFR	II	I	IV	Drosophila melanogaster	2.7.10.1

Table S2.3. (continuation)

UniProt	Label [1]	Two	Three	Seven	Species	EC Number
Q6SAM3	EGFR	II	I	IV	Drosophila melanogaster	2.7.10.1
Q6SAM5	EGFR	II	I	IV	Drosophila melanogaster	2.7.10.1
Q6SAM6	EGFR	II	I	IV	Drosophila melanogaster	2.7.10.1
Q6SAM7	EGFR	II	I	IV	Drosophila melanogaster	2.7.10.1
Q6SAM9	EGFR	II	I	IV	Drosophila melanogaster	2.7.10.1
Q6SAN0	EGFR	II	I	IV	Drosophila melanogaster	2.7.10.1
Q6SAN1	EGFR	II	I	IV	Drosophila melanogaster	2.7.10.1
Q6SAN2	EGFR	II	I	IV	Drosophila melanogaster	2.7.10.1
Q6SAN6	EGFR	II	I	IV	Drosophila melanogaster	2.7.10.1
Q6SAN7	EGFR	II	I	IV	Drosophila melanogaster	2.7.10.1
Q6SAN8	EGFR	II	I	IV	Drosophila melanogaster	2.7.10.1
Q6SAP0	EGFR	II	I	IV	Drosophila melanogaster	2.7.10.1
Q6SAP3	EGFR	II	I	IV	Drosophila melanogaster	2.7.10.1
Q6SAP4	EGFR	II	I	IV	Drosophila melanogaster	2.7.10.1
Q6SAP7	EGFR	II	I	IV	Drosophila melanogaster	2.7.10.1
Q6SAQ3	EGFR	II	I	IV	Drosophila melanogaster	2.7.10.1
Q6SAQ5	EGFR	II	I	IV	Drosophila melanogaster	2.7.10.1
Q6SAQ7	EGFR	II	I	IV	Drosophila melanogaster	2.7.10.1
Q6SAQ9	EGFR	II	I	IV	Drosophila melanogaster	2.7.10.1
Q6SAR2	EGFR	II	I	IV	Drosophila melanogaster	2.7.10.1
Q6SAR4	EGFR	II	I	IV	Drosophila melanogaster	2.7.10.1
Q6SAR7	EGFR	II	I	IV	Drosophila melanogaster	2.7.10.1
Q6SAR9	EGFR	II	I	IV	Drosophila melanogaster	2.7.10.1
Q6SAS0	EGFR	II	I	IV	Drosophila melanogaster	2.7.10.1
Q6SAS1	EGFR	II	I	IV	Drosophila melanogaster	2.7.10.1
Q6SAS6	EGFR	II	I	IV	Drosophila melanogaster	2.7.10.1
Q6SAS8	EGFR	II	I	IV	Drosophila melanogaster	2.7.10.1
Q6SAS9	EGFR	II	I	IV	Drosophila melanogaster	2.7.10.1
Q6SAT1	EGFR	II	I	IV	Drosophila melanogaster	2.7.10.1
Q6SAT3	EGFR	II	I	IV	Drosophila melanogaster	2.7.10.1
Q6SAT4	EGFR	II	I	IV	Drosophila melanogaster	2.7.10.1
Q6SAT5	EGFR	II	I	IV	Drosophila melanogaster	2.7.10.1
Q6SAT6	EGFR	II	I	IV	Drosophila melanogaster	2.7.10.1
Q6SAT7	EGFR	II	I	IV	Drosophila melanogaster	2.7.10.1
Q6SAT8	EGFR	II	I	IV	Drosophila melanogaster	2.7.10.1
Q6SAU1	EGFR	II	I	IV	Drosophila melanogaster	2.7.10.1
Q6SAU2	EGFR	II	I	IV	Drosophila melanogaster	2.7.10.1
Q6SAU3	EGFR	II	I	IV	Drosophila melanogaster	2.7.10.1
Q6SAU4	EGFR	II	I	IV	Drosophila melanogaster	2.7.10.1
Q6SAU5	EGFR	II	I	IV	Drosophila melanogaster	2.7.10.1
Q6SAU6	EGFR	II	I	IV	Drosophila melanogaster	2.7.10.1
Q6SAU7	EGFR	II	I	IV	Drosophila melanogaster	2.7.10.1
Q6SAU8	EGFR	II	I	IV	Drosophila melanogaster	2.7.10.1
Q6SAU9	EGFR	II	I	IV	Drosophila melanogaster	2.7.10.1
Q6SAV0	EGFR	II	I	IV	Drosophila melanogaster	2.7.10.1
Q6SAV1	EGFR	II	I	IV	Drosophila melanogaster	2.7.10.1
Q6SAV2	EGFR	II	I	IV	Drosophila melanogaster	2.7.10.1
Q6SAV3	EGFR	II	I	IV	Drosophila melanogaster	2.7.10.1
Q6SAV4	EGFR	II	I	IV	Drosophila melanogaster	2.7.10.1
Q6SAV5	EGFR	II	I	IV	Drosophila melanogaster	2.7.10.1
Q6SAV7	EGFR	II	I	IV	Drosophila melanogaster	2.7.10.1
Q6SAV8	EGFR	II	I	IV	Drosophila melanogaster	2.7.10.1
Q6SAV9	EGFR	II	I	IV	Drosophila melanogaster	2.7.10.1
Q6SAW0	EGFR	II	I	IV	Drosophila melanogaster	2.7.10.1
Q6SAW1	EGFR	II	I	IV	Drosophila melanogaster	2.7.10.1
Q6SAW2	EGFR	II	I	IV	Drosophila melanogaster	2.7.10.1

Table S2.3. (continuation)

UniProt	Label [1]	Two	Three	Seven	Species	EC Number
Q6SAW3	EGFR	II	I	IV	Drosophila melanogaster	2.7.10.1
Q6SAW4	EGFR	II	I	IV	Drosophila melanogaster	2.7.10.1
Q6SAW5	EGFR	II	I	IV	Drosophila melanogaster	2.7.10.1
Q6SAW8	EGFR	II	I	IV	Drosophila melanogaster	2.7.10.1
Q6SAW9	EGFR	II	I	IV	Drosophila melanogaster	2.7.10.1
Q6SAX0	EGFR	II	I	IV	Drosophila melanogaster	2.7.10.1
Q6SAX1	EGFR	II	I	IV	Drosophila melanogaster	2.7.10.1
Q6SAX2	EGFR	II	I	IV	Drosophila melanogaster	2.7.10.1
Q6SAX3	EGFR	II	I	IV	Drosophila melanogaster	2.7.10.1
Q6SAX4	EGFR	II	I	IV	Drosophila melanogaster	2.7.10.1
Q6SAX6	EGFR	II	I	IV	Drosophila melanogaster	2.7.10.1
Q6SAX7	EGFR	II	I	IV	Drosophila melanogaster	2.7.10.1
Q6SAX8	EGFR	II	I	IV	Drosophila melanogaster	2.7.10.1
Q6SAY1	EGFR	II	I	IV	Drosophila melanogaster	2.7.10.1
Q6SAY2	EGFR	II	I	IV	Drosophila melanogaster	2.7.10.1
Q6SAY3	EGFR	II	I	IV	Drosophila melanogaster	2.7.10.1
Q6SAY4	EGFR	II	I	IV	Drosophila melanogaster	2.7.10.1
Q6SAY6	EGFR	II	I	IV	Drosophila melanogaster	2.7.10.1
Q6SAY7	EGFR	II	I	IV	Drosophila melanogaster	2.7.10.1
Q6SAY8	EGFR	II	I	IV	Drosophila melanogaster	2.7.10.1
Q6SAY9	EGFR	II	I	IV	Drosophila melanogaster	2.7.10.1
Q6SAZ0	EGFR	II	I	IV	Drosophila melanogaster	2.7.10.1
Q6SAZ1	EGFR	II	I	IV	Drosophila melanogaster	2.7.10.1
Q6SAZ2	EGFR	II	I	IV	Drosophila melanogaster	2.7.10.1
Q6SAZ3	EGFR	II	I	IV	Drosophila melanogaster	2.7.10.1
Q6SAZ4	EGFR	II	I	IV	Drosophila melanogaster	2.7.10.1
Q6SAZ6	EGFR	II	I	IV	Drosophila melanogaster	2.7.10.1
Q6SAZ7	EGFR	II	I	IV	Drosophila melanogaster	2.7.10.1
Q6SAZ8	EGFR	II	I	IV	Drosophila melanogaster	2.7.10.1
Q6SAZ9	EGFR	II	I	IV	Drosophila melanogaster	2.7.10.1
Q6SB00	EGFR	II	I	IV	Drosophila melanogaster	2.7.10.1
Q6SB01	EGFR	II	I	IV	Drosophila melanogaster	2.7.10.1
Q86NZ2	EGFR	II	I	IV	Drosophila melanogaster	2.7.10.1
Q6SAE9	EGFR	II	I	IV	Drosophila melanogaster	
Q6SAF4	EGFR	II	I	IV	Drosophila melanogaster	
Q6SAF7	EGFR	II	I	IV	Drosophila melanogaster	
Q6SAH3	EGFR	II	I	IV	Drosophila melanogaster	
Q6SAK7	EGFR	II	I	IV	Drosophila melanogaster	
Q6SAS3	EGFR	II	I	IV	Drosophila melanogaster	
Q6SAS4	EGFR	II	I	IV	Drosophila melanogaster	
Q6SAS7	EGFR	II	I	IV	Drosophila melanogaster	
Q6SAT0	EGFR	II	I	IV	Drosophila melanogaster	
Q6SAT2	EGFR	II	I	IV	Drosophila melanogaster	
Q6SAT9	EGFR	II	I	IV	Drosophila melanogaster	
Q6SAU0	EGFR	II	I	IV	Drosophila melanogaster	
Q6SAV6	EGFR	II	I	IV	Drosophila melanogaster	
Q6SAW6	EGFR	II	I	IV	Drosophila melanogaster	
Q6SAW7	EGFR	II	I	IV	Drosophila melanogaster	
Q6SAX5	EGFR	II	I	IV	Drosophila melanogaster	
Q8SZW1	EGFR	II	I	IV	Drosophila melanogaster	
Q28XM9	EGFR	II	I	IV	Drosophila pseudoobscura	2.7.10.1
Q69AY2	EGFR	II	I	IV	Drosophila simulans	2.7.10.1
Q20C69	EGFR	II	I	IV	Drosophila virilis	2.7.10.1
Q49LT4	EGFR	II	I	IV	Felis catus	
Q68KJ7	EGFR	II	I	IV	Felis catus	
Q9W6F6	EGFR	II	I	IV	Gallus gallus	2.7.10.1

Table S2.3. (continuation)

UniProt	Label [1]	Two	Three	Seven	Species	EC Number
Q2EJ72	EGFR	II	I	IV	Gallus gallus	
Q4PLA4	EGFR	II	I	IV	Gallus gallus	
Q4PLA5	EGFR	II	I	IV	Gallus gallus	
Q59FL8	EGFR	II	I	IV	Homo sapiens	2.7.10.1
Q2TTR7	EGFR	II	I	IV	Homo sapiens	
Q504U8	EGFR	II	I	IV	Homo sapiens	
Q8C0E7	EGFR	II	I	IV	Mus musculus	2.7.10.1
Q9EP98	EGFR	II	I	IV	Mus musculus	
Q8K3F9	EGFR	II	I	IV	Rattus norvegicus	
Q9QX70	EGFR	II	I	IV	Rattus norvegicus	
A5A753	EGFR	II	I	IV	Sus scrofa	2.7.10.1
Q8MIL8	EGFR	II	I	IV	Sus scrofa	
Q4RWY4	EGFR	II	I	IV	Tetraodon nigroviridis	2.7.10.1
Q153D7	EGFR	II	I	IV	Xenopus laevis	2.7.10.1
Q153D8	EGFR	II	I	IV	Xenopus laevis	
Q153D9	EGFR	II	I	IV	Xenopus laevis	
Q6XJV8	EGFR	II	I	IV	Xiphophorus xiphidium	
Q9YH40	EGFR	II	I	IV	Xiphophorus xiphidium	
Q2HZD7	EGFR	II	I	V	Gallus gallus	2.7.10.1
Q6NYW1	Ser/Thr	I	II	V	Danio rerio	2.7.11.25
Q54TM7	Ser/Thr	I	II	V	Dictyostelium discoideum	2.7.11.1
A7SFG8	Ser/Thr	I	II	V	Nematostella vectensis	
Q0DE32	Ser/Thr	I	II	V	Oryza sativa	
Q5SMJ0	Ser/Thr	I	II	V	Oryza sativa	
Q5RCD1	Ser/Thr	I	II	V	Pongo abelii	2.7.11.25
Q4RX00	Ser/Thr	I	II	V	Tetraodon nigroviridis	
Q4S5N1	Ser/Thr	I	II	V	Tetraodon nigroviridis	
A7J1T0	Ser/Thr	I	II	V	Xenopus laevis	2.7.11.25
A7J1T1	Ser/Thr	I	II	V	Xenopus laevis	
A7J1T3	Ser/Thr	I	II	V	Xenopus laevis	
Q17M58	Ser/Thr	I	III	III	Aedes aegypti	2.7.11.13
Q17N56	Ser/Thr	I	III	III	Aedes aegypti	2.7.11.13
Q16VR1	Ser/Thr	I	III	III	Aedes aegypti	
Q16XQ9	Ser/Thr	I	III	III	Aedes aegypti	
Q17GR4	Ser/Thr	I	III	III	Aedes aegypti	
Q17KS0	Ser/Thr	I	III	III	Aedes aegypti	
Q868D7	Ser/Thr	I	III	III	Aedes aegypti	
A6QZ29	Ser/Thr	I	III	III	Ajellomyces capsulata	2.7.11.13
Q7QCP8	Ser/Thr	I	III	III	Anopheles gambiae	2.7.11.13
A7UR89	Ser/Thr	I	III	III	Anopheles gambiae	
Q7PTH7	Ser/Thr	I	III	III	Anopheles gambiae	
Q7Q042	Ser/Thr	I	III	III	Anopheles gambiae	
Q7QK56	Ser/Thr	I	III	III	Anopheles gambiae	
Q66XT6	Ser/Thr	I	III	III	Anopheles stephensi	
Q9U5H6	Ser/Thr	I	III	III	Apis mellifera	
Q75BT0	Ser/Thr	I	III	III	Ashbya gossypii	2.7.11.13
A1CBE3	Ser/Thr	I	III	III	Aspergillus clavatus	2.7.11.13
Q4WVG0	Ser/Thr	I	III	III	Aspergillus fumigatus	2.7.11.13
A2RA98	Ser/Thr	I	III	III	Aspergillus niger	2.7.11.13
Q2U6A7	Ser/Thr	I	III	III	Aspergillus oryzae	2.7.11.13
Q0CW56	Ser/Thr	I	III	III	Aspergillus terreus	2.7.11.13
Q95YJ0	Ser/Thr	I	III	III	Asterina pectinifera	
Q9HF10	Ser/Thr	I	III	III	Blumeria graminis	2.7.11.13
Q4AED5	Ser/Thr	I	III	III	Bombyx mori	2.7.11.13
Q4AED6	Ser/Thr	I	III	III	Bombyx mori	2.7.11.13
A0JN97	Ser/Thr	I	III	III	Bos taurus	2.7.11.13

Table S2.3. (continuation)

UniProt	Label [1]	Two	Three	Seven	Species	EC Number
A0JNH7	Ser/Thr	I	III	III	Bos taurus	2.7.11.13
A1A4I4	Ser/Thr	I	III	III	Bos taurus	2.7.11.13
Q0P5H4	Ser/Thr	I	III	III	Bos taurus	2.7.11.13
Q9UVJ5	Ser/Thr	I	III	III	Botrytis cinerea	
Q86M17	Ser/Thr	I	III	III	Branchiostoma lanceolatum	
O76850	Ser/Thr	I	III	III	Calliphora vicina	2.7.11.13
Q5ANK2	Ser/Thr	I	III	III	Candida albicans	2.7.11.13
Q6FJ43	Ser/Thr	I	III	III	Candida glabrata	2.7.11.13
Q5I9W4	Ser/Thr	I	III	III	Canis familiaris	
Q5I9W5	Ser/Thr	I	III	III	Canis familiaris	
Q2H265	Ser/Thr	I	III	III	Chaetomium globosum	2.7.11.13
Q55VU6	Ser/Thr	I	III	III	Cryptococcus neoformans	2.7.11.13
Q5KKE3	Ser/Thr	I	III	III	Cryptococcus neoformans	2.7.11.13
Q6UB96	Ser/Thr	I	III	III	Cryptococcus neoformans	2.7.11.13
Q6UB97	Ser/Thr	I	III	III	Cryptococcus neoformans	2.7.11.13
A7MBL8	Ser/Thr	I	III	III	Danio rerio	2.7.11.13
Q4JG04	Ser/Thr	I	III	III	Danio rerio	2.7.11.13
Q5TZD4	Ser/Thr	I	III	III	Danio rerio	2.7.11.13
Q6DI53	Ser/Thr	I	III	III	Danio rerio	2.7.11.13
Q7SY24	Ser/Thr	I	III	III	Danio rerio	2.7.11.13
Q7T2C5	Ser/Thr	I	III	III	Danio rerio	2.7.11.13
Q7ZUC5	Ser/Thr	I	III	III	Danio rerio	2.7.11.13
A2BIH5	Ser/Thr	I	III	III	Danio rerio	
Q49HN1	Ser/Thr	I	III	III	Danio rerio	
Q801L4	Ser/Thr	I	III	III	Danio rerio	
Q802Y3	Ser/Thr	I	III	III	Danio rerio	
Q8UUX0	Ser/Thr	I	III	III	Danio rerio	
Q6BI27	Ser/Thr	I	III	III	Debaryomyces hansenii	2.7.11.13
A1Z7T1	Ser/Thr	I	III	III	Drosophila melanogaster	2.7.11.-; 2.7.11.13
A1Z7T2	Ser/Thr	I	III	III	Drosophila melanogaster	2.7.11.-; 2.7.11.13
A1Z7T3	Ser/Thr	I	III	III	Drosophila melanogaster	2.7.11.-; 2.7.11.13
A1Z7T4	Ser/Thr	I	III	III	Drosophila melanogaster	2.7.11.-; 2.7.11.13
A8DY76	Ser/Thr	I	III	III	Drosophila melanogaster	2.7.11.-; 2.7.11.13
A1Z7T0	Ser/Thr	I	III	III	Drosophila melanogaster	2.7.11.13
A1Z9X0	Ser/Thr	I	III	III	Drosophila melanogaster	2.7.11.13
A8DYG9	Ser/Thr	I	III	III	Drosophila melanogaster	2.7.11.13
Q4QQA3	Ser/Thr	I	III	III	Drosophila melanogaster	2.7.11.13
Q95T78	Ser/Thr	I	III	III	Drosophila melanogaster	2.7.11.13
Q7YU74	Ser/Thr	I	III	III	Drosophila melanogaster	
Q960L7	Ser/Thr	I	III	III	Drosophila melanogaster	
Q28YC5	Ser/Thr	I	III	III	Drosophila pseudoobscura	2.7.11.13
Q290K2	Ser/Thr	I	III	III	Drosophila pseudoobscura	2.7.11.13
Q5BH74	Ser/Thr	I	III	III	Emericella nidulans	2.7.11.13
Q76G54	Ser/Thr	I	III	III	Emericella nidulans	2.7.11.13
Q8JFZ9	Ser/Thr	I	III	III	Fugu rubripes	2.7.11.13
Q5F3H2	Ser/Thr	I	III	III	Gallus gallus	2.7.11.13
Q5F3X1	Ser/Thr	I	III	III	Gallus gallus	2.7.11.13
Q5ZKE2	Ser/Thr	I	III	III	Gallus gallus	2.7.11.13
O57513	Ser/Thr	I	III	III	Gallus gallus	
O96997	Ser/Thr	I	III	III	Geodia cydonium	2.7.11.13
Q2TSD3	Ser/Thr	I	III	III	Homo sapiens	2.7.11.13
Q15523	Ser/Thr	I	III	III	Homo sapiens	
Q56A86	Ser/Thr	I	III	III	Homo sapiens	
Q59FI5	Ser/Thr	I	III	III	Homo sapiens	
O01715	Ser/Thr	I	III	III	Hydra attenuata	2.7.11.13
O01669	Ser/Thr	I	III	III	Hydra attenuata	

Table S2.3. (continuation)

UniProt	Label [1]	Two	Three	Seven	Species	EC Number
O01716	Ser/Thr	I	III	III	Hydra attenuata	
Q8MUA5	Ser/Thr	I	III	III	Hydra attenuata	
Q8MUA6	Ser/Thr	I	III	III	Hydra attenuata	
Q8J213	Ser/Thr	I	III	III	Kluyveromyces lactis	2.7.11.13
Q873Y9	Ser/Thr	I	III	III	Leptosphaeria maculans	
Q8MXB6	Ser/Thr	I	III	III	Limulus polyphemus	2.7.11.13
A5DZ73	Ser/Thr	I	III	III	Lodderomyces elongisporus	2.7.11.13
Q6UIN1	Ser/Thr	I	III	III	Macaca mulatta	
Q9Y7C1	Ser/Thr	I	III	III	Magnaporthe grisea	
A5DM82	Ser/Thr	I	III	III	Meyerozyma guilliermondii	2.7.11.13
Q1MX40	Ser/Thr	I	III	III	Mus musculus	2.7.11.13
Q1MX41	Ser/Thr	I	III	III	Mus musculus	2.7.11.13
Q1MX42	Ser/Thr	I	III	III	Mus musculus	2.7.11.13
Q1MX43	Ser/Thr	I	III	III	Mus musculus	2.7.11.13
Q2NKI4	Ser/Thr	I	III	III	Mus musculus	2.7.11.13
Q3TGE4	Ser/Thr	I	III	III	Mus musculus	2.7.11.13
Q3TJJ5	Ser/Thr	I	III	III	Mus musculus	2.7.11.13
Q3U1I0	Ser/Thr	I	III	III	Mus musculus	2.7.11.13
Q3UKY1	Ser/Thr	I	III	III	Mus musculus	2.7.11.13
Q3UMD5	Ser/Thr	I	III	III	Mus musculus	2.7.11.13
Q3UN66	Ser/Thr	I	III	III	Mus musculus	2.7.11.13
Q3UNG2	Ser/Thr	I	III	III	Mus musculus	2.7.11.13
Q4VA93	Ser/Thr	I	III	III	Mus musculus	2.7.11.13
Q53YN4	Ser/Thr	I	III	III	Mus musculus	2.7.11.13
Q8CAV6	Ser/Thr	I	III	III	Mus musculus	2.7.11.13
Q3TQ39	Ser/Thr	I	III	III	Mus musculus	
Q3TY95	Ser/Thr	I	III	III	Mus musculus	
Q3UGW9	Ser/Thr	I	III	III	Mus musculus	
Q3UHM5	Ser/Thr	I	III	III	Mus musculus	
Q3UQS3	Ser/Thr	I	III	III	Mus musculus	
Q3V341	Ser/Thr	I	III	III	Mus musculus	
Q5DTK3	Ser/Thr	I	III	III	Mus musculus	
Q6NXW0	Ser/Thr	I	III	III	Mus musculus	
Q6PF82	Ser/Thr	I	III	III	Mus musculus	
Q7TST7	Ser/Thr	I	III	III	Mus musculus	
Q8BS26	Ser/Thr	I	III	III	Mus musculus	
Q8BW55	Ser/Thr	I	III	III	Mus musculus	
Q8CE74	Ser/Thr	I	III	III	Mus musculus	
Q920N8	Ser/Thr	I	III	III	Mus musculus	
Q86M16	Ser/Thr	I	III	III	Myxine glutinosa	
A7RU22	Ser/Thr	I	III	III	Nematostella vectensis	2.7.11.13
A7SL27	Ser/Thr	I	III	III	Nematostella vectensis	2.7.11.13
A7RUL8	Ser/Thr	I	III	III	Nematostella vectensis	
A7RYZ0	Ser/Thr	I	III	III	Nematostella vectensis	
A8DVA9	Ser/Thr	I	III	III	Nematostella vectensis	
A1DDZ6	Ser/Thr	I	III	III	Neosartorya fischeri	2.7.11.13
Q5I6U5	Ser/Thr	I	III	III	Oryctolagus cuniculus	
Q6UIN2	Ser/Thr	I	III	III	Pan troglodytes	
Q801L6	Ser/Thr	I	III	III	Petromyzon marinus	
Q52PI7	Ser/Thr	I	III	III	Phallusia mammilata	
Q86ZV2	Ser/Thr	I	III	III	Pichia pastoris	2.7.11.13
O44393	Ser/Thr	I	III	III	Pisaster ochraceus	
Q5R944	Ser/Thr	I	III	III	Pongo abelii	
Q6EE98	Ser/Thr	I	III	III	Protopterus dolloi	
Q6EEA0	Ser/Thr	I	III	III	Protopterus dolloi	
Q6DUV1	Ser/Thr	I	III	III	Rattus norvegicus	2.7.11.13

Table S2.3. (continuation)

UniProt	Label [1]	Two	Three	Seven	Species	EC Number
Q3HSE5	Ser/Thr	I	III	III	Rattus norvegicus	
Q63432	Ser/Thr	I	III	III	Rattus norvegicus	
Q9R1X8	Ser/Thr	I	III	III	Rattus norvegicus	
O96942	Ser/Thr	I	III	III	Rhabdocalyptus dawsoni	2.7.11.13
A6ZKJ9	Ser/Thr	I	III	III	Saccharomyces cerevisiae	2.7.11.13
Q69G16	Ser/Thr	I	III	III	Schistosoma mansoni	2.7.11.13
A7F8U5	Ser/Thr	I	III	III	Sclerotinia sclerotiorum	
Q6EE44	Ser/Thr	I	III	III	Scyliorhinus canicula	
Q801L5	Ser/Thr	I	III	III	Scyliorhinus canicula	
Q9Y792	Ser/Thr	I	III	III	Sporothrix schenckii	
O62567	Ser/Thr	I	III	III	Suberites domuncula	2.7.11.13
O62569	Ser/Thr	I	III	III	Suberites domuncula	2.7.11.13
O62570	Ser/Thr	I	III	III	Suberites domuncula	
O61224	Ser/Thr	I	III	III	Sycon raphanus	2.7.11.13
O61225	Ser/Thr	I	III	III	Sycon raphanus	2.7.11.13
Q4RWN4	Ser/Thr	I	III	III	Tetraodon nigroviridis	2.7.11.13
Q4RLN6	Ser/Thr	I	III	III	Tetraodon nigroviridis	
Q4RM42	Ser/Thr	I	III	III	Tetraodon nigroviridis	
Q4RPP3	Ser/Thr	I	III	III	Tetraodon nigroviridis	
Q4RXV3	Ser/Thr	I	III	III	Tetraodon nigroviridis	
Q4RZQ9	Ser/Thr	I	III	III	Tetraodon nigroviridis	
Q4S9I8	Ser/Thr	I	III	III	Tetraodon nigroviridis	
Q4SBF1	Ser/Thr	I	III	III	Tetraodon nigroviridis	
Q4SG88	Ser/Thr	I	III	III	Tetraodon nigroviridis	
Q4SLA7	Ser/Thr	I	III	III	Tetraodon nigroviridis	
Q4SN64	Ser/Thr	I	III	III	Tetraodon nigroviridis	
Q4SZG9	Ser/Thr	I	III	III	Tetraodon nigroviridis	
Q4T021	Ser/Thr	I	III	III	Tetraodon nigroviridis	
Q9HGK8	Ser/Thr	I	III	III	Tuber borchii	2.7.11.13
Q96VF6	Ser/Thr	I	III	III	Tuber magnatum	
A7TMA2	Ser/Thr	I	III	III	Vanderwaltozyma polyspora	
Q498G7	Ser/Thr	I	III	III	Xenopus laevis	2.7.11.13
Q6AZF7	Ser/Thr	I	III	III	Xenopus laevis	2.7.11.13
Q6DCJ8	Ser/Thr	I	III	III	Xenopus laevis	2.7.11.13
Q6GNZ7	Ser/Thr	I	III	III	Xenopus laevis	2.7.11.13
Q7LZQ8	Ser/Thr	I	III	III	Xenopus laevis	2.7.11.13
Q7LZQ9	Ser/Thr	I	III	III	Xenopus laevis	2.7.11.13
Q7SZH7	Ser/Thr	I	III	III	Xenopus laevis	2.7.11.13
Q7SZH8	Ser/Thr	I	III	III	Xenopus laevis	2.7.11.13
Q91569	Ser/Thr	I	III	III	Xenopus laevis	2.7.11.13
O42262	Ser/Thr	I	III	III	Xenopus laevis	
Q91872	Ser/Thr	I	III	III	Xenopus laevis	
Q91948	Ser/Thr	I	III	III	Xenopus sp	
A0JM65	Ser/Thr	I	III	III	Xenopus tropicalis	2.7.11.13
Q08BT6	Ser/Thr	I	III	III	Xenopus tropicalis	2.7.11.13
Q28EN9	Ser/Thr	I	III	III	Xenopus tropicalis	2.7.11.13
Q5EGD6	Ser/Thr	I	III	III	Xenopus tropicalis	2.7.11.13
Q6C292	Ser/Thr	I	III	III	Yarrowia lipolytica	
Q16KG9	Ser/Thr	I	III	V	Aedes aegypti	
Q173R9	Ser/Thr	I	III	V	Aedes aegypti	
Q2UMV8	Ser/Thr	I	III	V	Aspergillus oryzae	
A6QQU8	Ser/Thr	I	III	V	Bos taurus	
Q4H2Q7	Ser/Thr	I	III	V	Ciona intestinalis	
Q4H378	Ser/Thr	I	III	V	Ciona intestinalis	
Q29GL6	Ser/Thr	I	III	V	Drosophila pseudoobscura	
Q6V675	Ser/Thr	I	III	V	Drosophila simulans	

Table S2.3. (continuation)

UniProt	Label [1]	Two	Three	Seven	Species	EC Number
Q6V679	Ser/Thr	I	III	V	Drosophila simulans	
Q6V681	Ser/Thr	I	III	V	Drosophila simulans	
Q6V4A0	Ser/Thr	I	III	V	Drosophila yakuba	
Q5AUJ7	Ser/Thr	I	III	V	Emericella nidulans	
O60030	Ser/Thr	I	III	V	Kluyveromyces lactis	
Q4Q5Q9	Ser/Thr	I	III	V	Leishmania major	2.7.11.1
Q4R987	Ser/Thr	I	III	V	Macaca fascicularis	2.7.11.25
Q1S5M5	Ser/Thr	I	III	V	Medicago truncatula	
A7RMS1	Ser/Thr	I	III	V	Nematostella vectensis	
A7SIN7	Ser/Thr	I	III	V	Nematostella vectensis	
A2DAP2	Ser/Thr	I	III	V	Trichomonas vaginalis	
A2DXP4	Ser/Thr	I	III	V	Trichomonas vaginalis	
A2EHA2	Ser/Thr	I	III	V	Trichomonas vaginalis	
A2EJW7	Ser/Thr	I	III	V	Trichomonas vaginalis	
Q388G0	Ser/Thr	I	III	V	Trypanosoma brucei	2.7.11.1
Q4E2T3	Ser/Thr	I	III	V	Trypanosoma cruzi	2.7.11.1
Q0IG70	Ser/Thr	I	III	VI	Aedes aegypti	
Q16E67	Ser/Thr	I	III	VI	Aedes aegypti	
Q16I98	Ser/Thr	I	III	VI	Aedes aegypti	
Q16UJ8	Ser/Thr	I	III	VI	Aedes aegypti	
Q170F5	Ser/Thr	I	III	VI	Aedes aegypti	
Q171I9	Ser/Thr	I	III	VI	Aedes aegypti	
Q173G6	Ser/Thr	I	III	VI	Aedes aegypti	
Q175B8	Ser/Thr	I	III	VI	Aedes aegypti	
Q17E44	Ser/Thr	I	III	VI	Aedes aegypti	
Q17E90	Ser/Thr	I	III	VI	Aedes aegypti	
Q17M45	Ser/Thr	I	III	VI	Aedes aegypti	
Q5BU47	Ser/Thr	I	III	VI	Aedes aegypti	
A6RGW3	Ser/Thr	I	III	VI	Ajellomyces capsulatus	
Q95UF4	Ser/Thr	I	III	VI	Ancylostoma caninum	
Q7QGS5	Ser/Thr	I	III	VI	Anopheles gambiae	2.7.11.1
A0NES6	Ser/Thr	I	III	VI	Anopheles gambiae	
Q5TMZ4	Ser/Thr	I	III	VI	Anopheles gambiae	
Q7PV95	Ser/Thr	I	III	VI	Anopheles gambiae	
Q7PY54	Ser/Thr	I	III	VI	Anopheles gambiae	
Q7Q3F8	Ser/Thr	I	III	VI	Anopheles gambiae	
Q7Q5I9	Ser/Thr	I	III	VI	Anopheles gambiae	
Q7QCI6	Ser/Thr	I	III	VI	Anopheles gambiae	
Q7QCL4	Ser/Thr	I	III	VI	Anopheles gambiae	
Q7QLC7	Ser/Thr	I	III	VI	Anopheles gambiae	
Q9U5H7	Ser/Thr	I	III	VI	Apis mellifera	
Q9M9B8	Ser/Thr	I	III	VI	Arabidopsis thaliana	2.7.11.-
O22971	Ser/Thr	I	III	VI	Arabidopsis thaliana	2.7.11.1
O65554	Ser/Thr	I	III	VI	Arabidopsis thaliana	2.7.11.1
O80902	Ser/Thr	I	III	VI	Arabidopsis thaliana	2.7.11.1
Q2V452	Ser/Thr	I	III	VI	Arabidopsis thaliana	2.7.11.1
Q5HZ38	Ser/Thr	I	III	VI	Arabidopsis thaliana	2.7.11.1
Q84VQ3	Ser/Thr	I	III	VI	Arabidopsis thaliana	2.7.11.1
Q8W1D5	Ser/Thr	I	III	VI	Arabidopsis thaliana	2.7.11.1
Q93V58	Ser/Thr	I	III	VI	Arabidopsis thaliana	2.7.11.1
Q93VD3	Ser/Thr	I	III	VI	Arabidopsis thaliana	2.7.11.1
Q94CG0	Ser/Thr	I	III	VI	Arabidopsis thaliana	2.7.11.1
Q9C562	Ser/Thr	I	III	VI	Arabidopsis thaliana	2.7.11.1
Q9FJ54	Ser/Thr	I	III	VI	Arabidopsis thaliana	2.7.11.1
Q9FJ55	Ser/Thr	I	III	VI	Arabidopsis thaliana	2.7.11.1
Q9LEU7	Ser/Thr	I	III	VI	Arabidopsis thaliana	2.7.11.1

Table S2.3. (continuation)

UniProt	Label [1]	Two	Three	Seven	Species	EC Number
Q9LP51	Ser/Thr	I	III	VI	Arabidopsis thaliana	2.7.11.1
Q9MAM1	Ser/Thr	I	III	VI	Arabidopsis thaliana	2.7.11.1
Q9SEZ7	Ser/Thr	I	III	VI	Arabidopsis thaliana	2.7.11.1
Q9SMQ4	Ser/Thr	I	III	VI	Arabidopsis thaliana	2.7.11.1
Q9SN43	Ser/Thr	I	III	VI	Arabidopsis thaliana	2.7.11.1
Q9STV4	Ser/Thr	I	III	VI	Arabidopsis thaliana	2.7.11.1
Q9SUL7	Ser/Thr	I	III	VI	Arabidopsis thaliana	2.7.11.1
Q9XIW0	Ser/Thr	I	III	VI	Arabidopsis thaliana	2.7.11.1
Q9ZV15	Ser/Thr	I	III	VI	Arabidopsis thaliana	2.7.11.1
A1IKU5	Ser/Thr	I	III	VI	Arabidopsis thaliana	
Q8S9D1	Ser/Thr	I	III	VI	Arabidopsis thaliana	
Q9FLZ3	Ser/Thr	I	III	VI	Arabidopsis thaliana	
Q9ZRA0	Ser/Thr	I	III	VI	Arabidopsis thaliana	
A1E371	Ser/Thr	I	III	VI	Artemia franciscana	
Q75CB5	Ser/Thr	I	III	VI	Ashbya gossypii	2.7.11.21
Q758J2	Ser/Thr	I	III	VI	Ashbya gossypii	
Q75BY7	Ser/Thr	I	III	VI	Ashbya gossypii	
Q75DQ1	Ser/Thr	I	III	VI	Ashbya gossypii	
A1CFH3	Ser/Thr	I	III	VI	Aspergillus clavatus	
Q4WII8	Ser/Thr	I	III	VI	Aspergillus fumigatus	2.7.1.-
A2QGW4	Ser/Thr	I	III	VI	Aspergillus niger	2.7.1.-
A2QKG1	Ser/Thr	I	III	VI	Aspergillus niger	
Q2TZA2	Ser/Thr	I	III	VI	Aspergillus oryzae	
Q2U7N6	Ser/Thr	I	III	VI	Aspergillus oryzae	
Q0CDL2	Ser/Thr	I	III	VI	Aspergillus terreus	
A7ANH6	Ser/Thr	I	III	VI	Babesia bovis	
Q86S94	Ser/Thr	I	III	VI	Babesia rodhaini	
A5YRY8	Ser/Thr	I	III	VI	Bombyx mori	
A6QNL2	Ser/Thr	I	III	VI	Bos taurus	
A8E649	Ser/Thr	I	III	VI	Bos taurus	
Q08DI2	Ser/Thr	I	III	VI	Bos taurus	
Q08DN4	Ser/Thr	I	III	VI	Bos taurus	
Q0P5A8	Ser/Thr	I	III	VI	Bos taurus	
Q0Z7W4	Ser/Thr	I	III	VI	Bos taurus	
Q58DJ9	Ser/Thr	I	III	VI	Bos taurus	
Q58DM2	Ser/Thr	I	III	VI	Bos taurus	
A2IBU0	Ser/Thr	I	III	VI	Brassica juncea	2.7.11.1
Q8W2D7	Ser/Thr	I	III	VI	Brassica napus	2.7.11.1
Q9ZPD6	Ser/Thr	I	III	VI	Brassica napus	
Q19469	Ser/Thr	I	III	VI	Caenorhabditis elegans	2.7.11.1
Q21017	Ser/Thr	I	III	VI	Caenorhabditis elegans	2.7.11.1
Q9TW45	Ser/Thr	I	III	VI	Caenorhabditis elegans	2.7.11.1
O62305	Ser/Thr	I	III	VI	Caenorhabditis elegans	2.7.11.17
Q9TXJ0	Ser/Thr	I	III	VI	Caenorhabditis elegans	2.7.11.17
O44747	Ser/Thr	I	III	VI	Caenorhabditis elegans	
Q9N3L4	Ser/Thr	I	III	VI	Caenorhabditis elegans	
Q59W62	Ser/Thr	I	III	VI	Candida albicans	2.7.11.1
Q5ABG0	Ser/Thr	I	III	VI	Candida albicans	2.7.11.21
Q5A1R0	Ser/Thr	I	III	VI	Candida albicans	
Q5A1W7	Ser/Thr	I	III	VI	Candida albicans	
Q6FJZ8	Ser/Thr	I	III	VI	Candida glabrata	
Q6FM66	Ser/Thr	I	III	VI	Candida glabrata	
Q2HGW9	Ser/Thr	I	III	VI	Chaetomium globosum	
Q6V8K6	Ser/Thr	I	III	VI	Chlamydomonas reinhardtii	
Q86SC3	Ser/Thr	I	III	VI	Ciona intestinalis	
Q9Y880	Ser/Thr	I	III	VI	Cochliobolus carbonum	

Table S2.3. (continuation)

UniProt	Label [1]	Two	Three	Seven	Species	EC Number
Q5CTW4	Ser/Thr	I	III	VI	Cryptosporidium parvum	
Q6S4W0	Ser/Thr	I	III	VI	Cryptosporidium parvum	
P93113	Ser/Thr	I	III	VI	Cucumis sativus	
A6H8T5	Ser/Thr	I	III	VI	Danio rerio	2.7.11.21
A1L290	Ser/Thr	I	III	VI	Danio rerio	
A2BHA2	Ser/Thr	I	III	VI	Danio rerio	
A2CEF7	Ser/Thr	I	III	VI	Danio rerio	
A5PN50	Ser/Thr	I	III	VI	Danio rerio	
A5WUM0	Ser/Thr	I	III	VI	Danio rerio	
A5WVQ5	Ser/Thr	I	III	VI	Danio rerio	
Q05AN8	Ser/Thr	I	III	VI	Danio rerio	
Q1MTK5	Ser/Thr	I	III	VI	Danio rerio	
Q4V9P8	Ser/Thr	I	III	VI	Danio rerio	
Q502F3	Ser/Thr	I	III	VI	Danio rerio	
Q6DBZ4	Ser/Thr	I	III	VI	Danio rerio	
Q6DHU9	Ser/Thr	I	III	VI	Danio rerio	
Q6P954	Ser/Thr	I	III	VI	Danio rerio	
Q6P965	Ser/Thr	I	III	VI	Danio rerio	
Q6PC33	Ser/Thr	I	III	VI	Danio rerio	
Q802W0	Ser/Thr	I	III	VI	Danio rerio	
Q6BLX3	Ser/Thr	I	III	VI	Debaryomyces hansenii	2.7.11.21
Q6BM35	Ser/Thr	I	III	VI	Debaryomyces hansenii	
Q6BPA5	Ser/Thr	I	III	VI	Debaryomyces hansenii	
Q54DF2	Ser/Thr	I	III	VI	Dictyostelium discoideum	2.7.11.1
Q54TA3	Ser/Thr	I	III	VI	Dictyostelium discoideum	2.7.11.1
Q54WX4	Ser/Thr	I	III	VI	Dictyostelium discoideum	2.7.11.1
Q54YF2	Ser/Thr	I	III	VI	Dictyostelium discoideum	2.7.11.1
Q55FT4	Ser/Thr	I	III	VI	Dictyostelium discoideum	2.7.11.1
Q76P07	Ser/Thr	I	III	VI	Dictyostelium discoideum	2.7.11.1
Q869W6	Ser/Thr	I	III	VI	Dictyostelium discoideum	2.7.11.18
Q5I4J6	Ser/Thr	I	III	VI	Drosophila americana	
Q5I4J2	Ser/Thr	I	III	VI	Drosophila ezoana	
A7Y4I9	Ser/Thr	I	III	VI	Drosophila littoralis	
Q0E981	Ser/Thr	I	III	VI	Drosophila melanogaster	2.7.11.-
Q4QQA7	Ser/Thr	I	III	VI	Drosophila melanogaster	2.7.11.-
Q9VGF9	Ser/Thr	I	III	VI	Drosophila melanogaster	2.7.11.-
Q9VGP9	Ser/Thr	I	III	VI	Drosophila melanogaster	2.7.11.-
Q9VGQ0	Ser/Thr	I	III	VI	Drosophila melanogaster	2.7.11.-
Q9VUV4	Ser/Thr	I	III	VI	Drosophila melanogaster	2.7.11.-
Q9W532	Ser/Thr	I	III	VI	Drosophila melanogaster	2.7.11.-
O18645	Ser/Thr	I	III	VI	Drosophila melanogaster	2.7.11.-; 2.7.11.16
A4V133	Ser/Thr	I	III	VI	Drosophila melanogaster	2.7.11.-; 2.7.11.17
A4V134	Ser/Thr	I	III	VI	Drosophila melanogaster	2.7.11.-; 2.7.11.17
Q0KIF7	Ser/Thr	I	III	VI	Drosophila melanogaster	2.7.11.-; 2.7.11.17
A1ZBL5	Ser/Thr	I	III	VI	Drosophila melanogaster	2.7.11.-; 2.7.11.26
A1ZBL7	Ser/Thr	I	III	VI	Drosophila melanogaster	2.7.11.-; 2.7.11.26
A1ZBL9	Ser/Thr	I	III	VI	Drosophila melanogaster	2.7.11.-; 2.7.11.26
Q6NPA6	Ser/Thr	I	III	VI	Drosophila melanogaster	2.7.11.-; 2.7.11.26
Q7YU80	Ser/Thr	I	III	VI	Drosophila melanogaster	2.7.11.-; 2.7.11.26
Q963E5	Ser/Thr	I	III	VI	Drosophila melanogaster	2.7.11.-; 2.7.11.26
Q963E6	Ser/Thr	I	III	VI	Drosophila melanogaster	2.7.11.-; 2.7.11.26
Q9V8V8	Ser/Thr	I	III	VI	Drosophila melanogaster	2.7.11.-; 2.7.11.26
Q7PLI7	Ser/Thr	I	III	VI	Drosophila melanogaster	2.7.11.1
O17048	Ser/Thr	I	III	VI	Drosophila melanogaster	
O17049	Ser/Thr	I	III	VI	Drosophila melanogaster	
O77268	Ser/Thr	I	III	VI	Drosophila melanogaster	

Table S2.3. (continuation)

UniProt	Label [1]	Two	Three	Seven	Species	EC Number
Q058V4	Ser/Thr	I	III	VI	Drosophila melanogaster	
Q27393	Ser/Thr	I	III	VI	Drosophila melanogaster	
Q5U118	Ser/Thr	I	III	VI	Drosophila melanogaster	
Q8IGW6	Ser/Thr	I	III	VI	Drosophila melanogaster	
Q8SWX3	Ser/Thr	I	III	VI	Drosophila melanogaster	
Q8T473	Ser/Thr	I	III	VI	Drosophila melanogaster	
Q95U75	Ser/Thr	I	III	VI	Drosophila melanogaster	
Q960G5	Ser/Thr	I	III	VI	Drosophila melanogaster	
Q9VYV7	Ser/Thr	I	III	VI	Drosophila melanogaster	
Q1PQ60	Ser/Thr	I	III	VI	Drosophila miranda	
A7Y4J2	Ser/Thr	I	III	VI	Drosophila montana	
Q29IZ7	Ser/Thr	I	III	VI	Drosophila pseudoobscura	2.7.11.-
Q29GZ7	Ser/Thr	I	III	VI	Drosophila pseudoobscura	2.7.11.1
Q28X63	Ser/Thr	I	III	VI	Drosophila pseudoobscura	
Q293S6	Ser/Thr	I	III	VI	Drosophila pseudoobscura	
Q295U1	Ser/Thr	I	III	VI	Drosophila pseudoobscura	
Q2M0V5	Ser/Thr	I	III	VI	Drosophila pseudoobscura	
Q5I4J8	Ser/Thr	I	III	VI	Drosophila virilis	
Q6XIL0	Ser/Thr	I	III	VI	Drosophila yakuba	
Q8SWM6	Ser/Thr	I	III	VI	Encephalitozoon cuniculi	2.7.11.21
Q2LGF9	Ser/Thr	I	III	VI	Equus caballus	
Q2LGG0	Ser/Thr	I	III	VI	Equus caballus	
Q70CF4	Ser/Thr	I	III	VI	Fagus sylvatica	2.7.1.-
Q70AB5	Ser/Thr	I	III	VI	Fagus sylvatica	
Q70CF2	Ser/Thr	I	III	VI	Fagus sylvatica	
Q8J2N0	Ser/Thr	I	III	VI	Fusarium oxysporum	
Q5F3M9	Ser/Thr	I	III	VI	Gallus gallus	2.7.11.13
A1EAT1	Ser/Thr	I	III	VI	Gallus gallus	
Q2LAI0	Ser/Thr	I	III	VI	Gallus gallus	
Q2PUH1	Ser/Thr	I	III	VI	Gallus gallus	
Q5ZL85	Ser/Thr	I	III	VI	Gallus gallus	
A8BHW1	Ser/Thr	I	III	VI	Giardia intestinalis	
A8BIZ4	Ser/Thr	I	III	VI	Giardia intestinalis	
A8BU43	Ser/Thr	I	III	VI	Giardia intestinalis	
Q8LK24	Ser/Thr	I	III	VI	Glycine max	2.7.11.1
Q9XF25	Ser/Thr	I	III	VI	Glycine max	
A5YAI9	Ser/Thr	I	III	VI	Gossypium hirsutum	2.7.11.1
Q6DXR4	Ser/Thr	I	III	VI	Gossypium hirsutum	2.7.11.1
Q98RL9	Ser/Thr	I	III	VI	Guillardia theta	
Q5ILR0	Ser/Thr	I	III	VI	Haemonchus contortus	
Q5ILR1	Ser/Thr	I	III	VI	Haemonchus contortus	
Q5ILR2	Ser/Thr	I	III	VI	Haemonchus contortus	
Q5ILR3	Ser/Thr	I	III	VI	Haemonchus contortus	
Q8MVW9	Ser/Thr	I	III	VI	Haemonchus contortus	
Q8MVX0	Ser/Thr	I	III	VI	Haemonchus contortus	
Q8MVX1	Ser/Thr	I	III	VI	Haemonchus contortus	
Q8MVX2	Ser/Thr	I	III	VI	Haemonchus contortus	
Q8MVX3	Ser/Thr	I	III	VI	Haemonchus contortus	
Q8MVX4	Ser/Thr	I	III	VI	Haemonchus contortus	
O61298	Ser/Thr	I	III	VI	Halocynthia roretzi	
A0MZF5	Ser/Thr	I	III	VI	Homo sapiens	
A1A5A9	Ser/Thr	I	III	VI	Homo sapiens	
Q53GX0	Ser/Thr	I	III	VI	Homo sapiens	
Q59GD0	Ser/Thr	I	III	VI	Homo sapiens	
Q59GQ4	Ser/Thr	I	III	VI	Homo sapiens	
Q5SQQ7	Ser/Thr	I	III	VI	Homo sapiens	

Table S2.3. (continuation)

UniProt	Label [1]	Two	Three	Seven	Species	EC Number
Q5SWX3	Ser/Thr	I	III	VI	Homo sapiens	
Q5Y191	Ser/Thr	I	III	VI	Homo sapiens	
Q8N2N5	Ser/Thr	I	III	VI	Homo sapiens	
Q8N7M6	Ser/Thr	I	III	VI	Homo sapiens	
Q8WU40	Ser/Thr	I	III	VI	Homo sapiens	
Q96E92	Ser/Thr	I	III	VI	Homo sapiens	
A5HLX6	Ser/Thr	I	III	VI	Hordeum brevisubulatum	2.7.11.1
O81992	Ser/Thr	I	III	VI	Hordeum vulgare	
Q40029	Ser/Thr	I	III	VI	Hordeum vulgare	
Q40030	Ser/Thr	I	III	VI	Hordeum vulgare	
Q43475	Ser/Thr	I	III	VI	Hordeum vulgare	
Q6CUB8	Ser/Thr	I	III	VI	Kluyveromyces lactis	2.7.11.21
P87209	Ser/Thr	I	III	VI	Kluyveromyces lactis	
Q6CIX7	Ser/Thr	I	III	VI	Kluyveromyces lactis	
Q6CKE4	Ser/Thr	I	III	VI	Kluyveromyces lactis	
Q7Z865	Ser/Thr	I	III	VI	Kluyveromyces lactis	
Q9N2N1	Ser/Thr	I	III	VI	Leishmania major	2.7.1.37
Q4QIJ7	Ser/Thr	I	III	VI	Leishmania major	2.7.11.1
Q4QDX7	Ser/Thr	I	III	VI	Leishmania major	
Q4QFJ2	Ser/Thr	I	III	VI	Leishmania major	
Q4QGB0	Ser/Thr	I	III	VI	Leishmania major	
Q9GRT3	Ser/Thr	I	III	VI	Leishmania mexicana	
A5DYF0	Ser/Thr	I	III	VI	Lodderomyces elongisporus	
A5E2E8	Ser/Thr	I	III	VI	Lodderomyces elongisporus	
Q53UK3	Ser/Thr	I	III	VI	Lotus japonicus	2.7.11.1
Q53UK5	Ser/Thr	I	III	VI	Lotus japonicus	2.7.11.1
Q53VD9	Ser/Thr	I	III	VI	Lotus japonicus	2.7.11.1
Q53VE1	Ser/Thr	I	III	VI	Lotus japonicus	2.7.11.1
Q53VM2	Ser/Thr	I	III	VI	Lotus japonicus	2.7.11.1
Q53VM3	Ser/Thr	I	III	VI	Lotus japonicus	2.7.11.1
Q53UK2	Ser/Thr	I	III	VI	Lotus japonicus	
Q53UK4	Ser/Thr	I	III	VI	Lotus japonicus	
Q53VM4	Ser/Thr	I	III	VI	Lotus japonicus	
Q4R7Q6	Ser/Thr	I	III	VI	Macaca fascicularis	2.7.11.21
Q4R9G2	Ser/Thr	I	III	VI	Macaca fascicularis	
Q1HGK2	Ser/Thr	I	III	VI	Magnaporthe grisea	
Q3ZZY3	Ser/Thr	I	III	VI	Marthasterias glacialis	
A1E4B7	Ser/Thr	I	III	VI	Meleagris gallopavo	
Q9XFJ3	Ser/Thr	I	III	VI	Mesembryanthemum crystallinum	2.7.11.1
A5DLX5	Ser/Thr	I	III	VI	Meyerozyma guilliermondii	2.7.11.21
A5DC34	Ser/Thr	I	III	VI	Meyerozyma guilliermondii	
Q3TPZ2	Ser/Thr	I	III	VI	Mus musculus	2.7.11.21
Q3UG81	Ser/Thr	I	III	VI	Mus musculus	2.7.11.21
Q548A9	Ser/Thr	I	III	VI	Mus musculus	2.7.11.21
Q8K226	Ser/Thr	I	III	VI	Mus musculus	2.7.11.21
A2ALM3	Ser/Thr	I	III	VI	Mus musculus	
A2AQX6	Ser/Thr	I	III	VI	Mus musculus	
Q14DQ3	Ser/Thr	I	III	VI	Mus musculus	
Q1EDH1	Ser/Thr	I	III	VI	Mus musculus	
Q3T9A3	Ser/Thr	I	III	VI	Mus musculus	
Q3TDM2	Ser/Thr	I	III	VI	Mus musculus	
Q3TJF3	Ser/Thr	I	III	VI	Mus musculus	
Q3TU15	Ser/Thr	I	III	VI	Mus musculus	
Q3TUQ7	Ser/Thr	I	III	VI	Mus musculus	
Q3TY93	Ser/Thr	I	III	VI	Mus musculus	
Q3TYC1	Ser/Thr	I	III	VI	Mus musculus	

Table S2.3. (continuation)

UniProt	Label [1]	Two	Three	Seven	Species	EC Number
Q3U3A1	Ser/Thr	I	III	VI	Mus musculus	
Q3UH04	Ser/Thr	I	III	VI	Mus musculus	
Q3UMB0	Ser/Thr	I	III	VI	Mus musculus	
Q3UT86	Ser/Thr	I	III	VI	Mus musculus	
Q3UTA8	Ser/Thr	I	III	VI	Mus musculus	
Q3UYI0	Ser/Thr	I	III	VI	Mus musculus	
Q497X5	Ser/Thr	I	III	VI	Mus musculus	
Q571I6	Ser/Thr	I	III	VI	Mus musculus	
Q571J8	Ser/Thr	I	III	VI	Mus musculus	
Q5DTG3	Ser/Thr	I	III	VI	Mus musculus	
Q5U440	Ser/Thr	I	III	VI	Mus musculus	
Q6P209	Ser/Thr	I	III	VI	Mus musculus	
Q6ZWS7	Ser/Thr	I	III	VI	Mus musculus	
Q8BLX0	Ser/Thr	I	III	VI	Mus musculus	
Q8BQN2	Ser/Thr	I	III	VI	Mus musculus	
Q8C1B3	Ser/Thr	I	III	VI	Mus musculus	
Q8CCN4	Ser/Thr	I	III	VI	Mus musculus	
Q8CHG1	Ser/Thr	I	III	VI	Mus musculus	
Q9JKE5	Ser/Thr	I	III	VI	Mus musculus	
Q9JLM6	Ser/Thr	I	III	VI	Mus musculus	
Q8WMW5	Ser/Thr	I	III	VI	Mustela putorius	
Q8WMW6	Ser/Thr	I	III	VI	Mustela putorius	
Q8WMW7	Ser/Thr	I	III	VI	Mustela putorius	
Q95262	Ser/Thr	I	III	VI	Mustela putorius	
Q95263	Ser/Thr	I	III	VI	Mustela putorius	
Q95265	Ser/Thr	I	III	VI	Mustela putorius	
A7RF52	Ser/Thr	I	III	VI	Nematostella vectensis	
A7RR22	Ser/Thr	I	III	VI	Nematostella vectensis	
A7RRL1	Ser/Thr	I	III	VI	Nematostella vectensis	
A7RV77	Ser/Thr	I	III	VI	Nematostella vectensis	
A7RVB0	Ser/Thr	I	III	VI	Nematostella vectensis	
A7RVS7	Ser/Thr	I	III	VI	Nematostella vectensis	
A7S1C2	Ser/Thr	I	III	VI	Nematostella vectensis	
A7S1X1	Ser/Thr	I	III	VI	Nematostella vectensis	
A7S4Z5	Ser/Thr	I	III	VI	Nematostella vectensis	
A7S6E0	Ser/Thr	I	III	VI	Nematostella vectensis	
A7SI81	Ser/Thr	I	III	VI	Nematostella vectensis	
A7SJ85	Ser/Thr	I	III	VI	Nematostella vectensis	
A7SJE6	Ser/Thr	I	III	VI	Nematostella vectensis	
A7SL08	Ser/Thr	I	III	VI	Nematostella vectensis	
A7SLH0	Ser/Thr	I	III	VI	Nematostella vectensis	
A7SXJ7	Ser/Thr	I	III	VI	Nematostella vectensis	
A7SZV3	Ser/Thr	I	III	VI	Nematostella vectensis	
A7T0H5	Ser/Thr	I	III	VI	Nematostella vectensis	
A1CYL6	Ser/Thr	I	III	VI	Neosartorya fischeri	
A1DG71	Ser/Thr	I	III	VI	Neosartorya fischeri	
Q7RVR5	Ser/Thr	I	III	VI	Neurospora crassa	
Q872H0	Ser/Thr	I	III	VI	Neurospora crassa	
Q5QIT6	Ser/Thr	I	III	VI	Nicotiana attenuata	
Q2LAG3	Ser/Thr	I	III	VI	Nicotiana tabacum	2.7.11.1
Q84QE0	Ser/Thr	I	III	VI	Nicotiana tabacum	2.7.11.1
Q40544	Ser/Thr	I	III	VI	Nicotiana tabacum	
Q9ZNW3	Ser/Thr	I	III	VI	Nicotiana tabacum	
Q3LDS4	Ser/Thr	I	III	VI	Nyctotherus ovalis	
Q8WQ18	Ser/Thr	I	III	VI	Nyctotherus ovalis	
Q675U2	Ser/Thr	I	III	VI	Oikopleura dioica	

Table S2.3. (continuation)

UniProt	Label [1]	Two	Three	Seven	Species	EC Number
Q64HW3	Ser/Thr	I	III	VI	Oncorhynchus mykiss	
O77707	Ser/Thr	I	III	VI	Oryctolagus cuniculus	
A2WLW3	Ser/Thr	I	III	VI	Oryza sativa	2.7.11.1
A2WLW5	Ser/Thr	I	III	VI	Oryza sativa	2.7.11.1
A2WV99	Ser/Thr	I	III	VI	Oryza sativa	2.7.11.1
A2X165	Ser/Thr	I	III	VI	Oryza sativa	2.7.11.1
A2Y1H9	Ser/Thr	I	III	VI	Oryza sativa	2.7.11.1
A2Y3A6	Ser/Thr	I	III	VI	Oryza sativa	2.7.11.1
A2Y6H1	Ser/Thr	I	III	VI	Oryza sativa	2.7.11.1
A2YDV4	Ser/Thr	I	III	VI	Oryza sativa	2.7.11.1
A2YPX9	Ser/Thr	I	III	VI	Oryza sativa	2.7.11.1
A2YPY3	Ser/Thr	I	III	VI	Oryza sativa	2.7.11.1
A2YQ36	Ser/Thr	I	III	VI	Oryza sativa	2.7.11.1
A2YVM1	Ser/Thr	I	III	VI	Oryza sativa	2.7.11.1
A2Z1A6	Ser/Thr	I	III	VI	Oryza sativa	2.7.11.1
A2Z1A7	Ser/Thr	I	III	VI	Oryza sativa	2.7.11.1
Q0JI49	Ser/Thr	I	III	VI	Oryza sativa	2.7.11.1
Q10LQ2	Ser/Thr	I	III	VI	Oryza sativa	2.7.11.1
Q10M83	Ser/Thr	I	III	VI	Oryza sativa	2.7.11.1
Q10SC8	Ser/Thr	I	III	VI	Oryza sativa	2.7.11.1
Q2QY53	Ser/Thr	I	III	VI	Oryza sativa	2.7.11.1
Q2QYM3	Ser/Thr	I	III	VI	Oryza sativa	2.7.11.1
Q2RAX3	Ser/Thr	I	III	VI	Oryza sativa	2.7.11.1
Q2RBF0	Ser/Thr	I	III	VI	Oryza sativa	2.7.11.1
Q304W8	Ser/Thr	I	III	VI	Oryza sativa	2.7.11.1
Q5JLD8	Ser/Thr	I	III	VI	Oryza sativa	2.7.11.1
Q5JLQ9	Ser/Thr	I	III	VI	Oryza sativa	2.7.11.1
Q5JLS2	Ser/Thr	I	III	VI	Oryza sativa	2.7.11.1
Q5QNM6	Ser/Thr	I	III	VI	Oryza sativa	2.7.11.1
Q5W736	Ser/Thr	I	III	VI	Oryza sativa	2.7.11.1
Q5Z6X0	Ser/Thr	I	III	VI	Oryza sativa	2.7.11.1
Q60EY8	Ser/Thr	I	III	VI	Oryza sativa	2.7.11.1
Q68Y49	Ser/Thr	I	III	VI	Oryza sativa	2.7.11.1
Q69Q47	Ser/Thr	I	III	VI	Oryza sativa	2.7.11.1
Q6ERS0	Ser/Thr	I	III	VI	Oryza sativa	2.7.11.1
Q6ERS4	Ser/Thr	I	III	VI	Oryza sativa	2.7.11.1
Q6ERS5	Ser/Thr	I	III	VI	Oryza sativa	2.7.11.1
Q6ETM9	Ser/Thr	I	III	VI	Oryza sativa	2.7.11.1
Q6H7U5	Ser/Thr	I	III	VI	Oryza sativa	2.7.11.1
Q6Z9F4	Ser/Thr	I	III	VI	Oryza sativa	2.7.11.1
Q6ZLP5	Ser/Thr	I	III	VI	Oryza sativa	2.7.11.1
Q75L42	Ser/Thr	I	III	VI	Oryza sativa	2.7.11.1
Q7X996	Ser/Thr	I	III	VI	Oryza sativa	2.7.11.1
Q7XIW5	Ser/Thr	I	III	VI	Oryza sativa	2.7.11.1
Q8LIG4	Ser/Thr	I	III	VI	Oryza sativa	2.7.11.1
Q9LGV5	Ser/Thr	I	III	VI	Oryza sativa	2.7.11.1
Q9LWM4	Ser/Thr	I	III	VI	Oryza sativa	2.7.11.1
A2XFF4	Ser/Thr	I	III	VI	Oryza sativa	
A6N1J0	Ser/Thr	I	III	VI	Oryza sativa	
Q0DGI1	Ser/Thr	I	III	VI	Oryza sativa	
Q10M84	Ser/Thr	I	III	VI	Oryza sativa	
Q10M85	Ser/Thr	I	III	VI	Oryza sativa	
Q10MY9	Ser/Thr	I	III	VI	Oryza sativa	
Q10NI6	Ser/Thr	I	III	VI	Oryza sativa	
Q10NI8	Ser/Thr	I	III	VI	Oryza sativa	
Q10NI9	Ser/Thr	I	III	VI	Oryza sativa	

Table S2.3. (continuation)

UniProt	Label [1]	Two	Three	Seven	Species	EC Number
Q10SC7	Ser/Thr	I	III	VI	Oryza sativa	
Q40740	Ser/Thr	I	III	VI	Oryza sativa	
Q852N6	Ser/Thr	I	III	VI	Oryza sativa	
Q852Q0	Ser/Thr	I	III	VI	Oryza sativa	
Q852Q1	Ser/Thr	I	III	VI	Oryza sativa	
Q852Q2	Ser/Thr	I	III	VI	Oryza sativa	
Q9ZNT4	Ser/Thr	I	III	VI	Oryza sativa	
Q9ZRJ1	Ser/Thr	I	III	VI	Oryza sativa	
Q9ZRJ2	Ser/Thr	I	III	VI	Oryza sativa	
Q9ZRJ3	Ser/Thr	I	III	VI	Oryza sativa	
Q9ZTF6	Ser/Thr	I	III	VI	Oryza sativa	
A4RR81	Ser/Thr	I	III	VI	Ostreococcus lucimarinus	
A4RZA4	Ser/Thr	I	III	VI	Ostreococcus lucimarinus	
A4S3J2	Ser/Thr	I	III	VI	Ostreococcus lucimarinus	
A4S5M2	Ser/Thr	I	III	VI	Ostreococcus lucimarinus	
A4S845	Ser/Thr	I	III	VI	Ostreococcus lucimarinus	
Q010G9	Ser/Thr	I	III	VI	Ostreococcus tauri	
A8D245	Ser/Thr	I	III	VI	Ovis aries	
Q6Y242	Ser/Thr	I	III	VI	Pagrus major	
A0BBI3	Ser/Thr	I	III	VI	Paramecium tetraurelia	
A0BCL5	Ser/Thr	I	III	VI	Paramecium tetraurelia	
A0BE53	Ser/Thr	I	III	VI	Paramecium tetraurelia	
A0BER8	Ser/Thr	I	III	VI	Paramecium tetraurelia	
A0BFB7	Ser/Thr	I	III	VI	Paramecium tetraurelia	
A0BFM2	Ser/Thr	I	III	VI	Paramecium tetraurelia	
A0BH55	Ser/Thr	I	III	VI	Paramecium tetraurelia	
A0BJ15	Ser/Thr	I	III	VI	Paramecium tetraurelia	
A0BJ35	Ser/Thr	I	III	VI	Paramecium tetraurelia	
A0BJ43	Ser/Thr	I	III	VI	Paramecium tetraurelia	
A0BLB9	Ser/Thr	I	III	VI	Paramecium tetraurelia	
A0BLG1	Ser/Thr	I	III	VI	Paramecium tetraurelia	
A0BM97	Ser/Thr	I	III	VI	Paramecium tetraurelia	
A0BMJ2	Ser/Thr	I	III	VI	Paramecium tetraurelia	
A0BN81	Ser/Thr	I	III	VI	Paramecium tetraurelia	
A0BR89	Ser/Thr	I	III	VI	Paramecium tetraurelia	
A0BS70	Ser/Thr	I	III	VI	Paramecium tetraurelia	
A0BS82	Ser/Thr	I	III	VI	Paramecium tetraurelia	
A0BSF6	Ser/Thr	I	III	VI	Paramecium tetraurelia	
A0BSF7	Ser/Thr	I	III	VI	Paramecium tetraurelia	
A0BSN3	Ser/Thr	I	III	VI	Paramecium tetraurelia	
A0BSS3	Ser/Thr	I	III	VI	Paramecium tetraurelia	
A0BTJ3	Ser/Thr	I	III	VI	Paramecium tetraurelia	
A0BTX5	Ser/Thr	I	III	VI	Paramecium tetraurelia	
A0BUS3	Ser/Thr	I	III	VI	Paramecium tetraurelia	
A0BVJ1	Ser/Thr	I	III	VI	Paramecium tetraurelia	
A0BWA1	Ser/Thr	I	III	VI	Paramecium tetraurelia	
A0BX15	Ser/Thr	I	III	VI	Paramecium tetraurelia	
A0BXP1	Ser/Thr	I	III	VI	Paramecium tetraurelia	
A0BXY8	Ser/Thr	I	III	VI	Paramecium tetraurelia	
A0BZI1	Ser/Thr	I	III	VI	Paramecium tetraurelia	
A0C041	Ser/Thr	I	III	VI	Paramecium tetraurelia	
A0C1X6	Ser/Thr	I	III	VI	Paramecium tetraurelia	
A0C2X0	Ser/Thr	I	III	VI	Paramecium tetraurelia	
A0C2X8	Ser/Thr	I	III	VI	Paramecium tetraurelia	
A0C3X2	Ser/Thr	I	III	VI	Paramecium tetraurelia	
A0C4B8	Ser/Thr	I	III	VI	Paramecium tetraurelia	

Table S2.3. (continuation)

UniProt	Label [1]	Two	Three	Seven	Species	EC Number
A0C5A2	Ser/Thr	I	III	VI	Paramecium tetraurelia	
A0C5H8	Ser/Thr	I	III	VI	Paramecium tetraurelia	
A0C6Y8	Ser/Thr	I	III	VI	Paramecium tetraurelia	
A0C7X3	Ser/Thr	I	III	VI	Paramecium tetraurelia	
A0C8T4	Ser/Thr	I	III	VI	Paramecium tetraurelia	
A0C9A8	Ser/Thr	I	III	VI	Paramecium tetraurelia	
A0C9H3	Ser/Thr	I	III	VI	Paramecium tetraurelia	
A0CDF8	Ser/Thr	I	III	VI	Paramecium tetraurelia	
A0CDN5	Ser/Thr	I	III	VI	Paramecium tetraurelia	
A0CDU1	Ser/Thr	I	III	VI	Paramecium tetraurelia	
A0CET1	Ser/Thr	I	III	VI	Paramecium tetraurelia	
A0CGU4	Ser/Thr	I	III	VI	Paramecium tetraurelia	
A0CHZ8	Ser/Thr	I	III	VI	Paramecium tetraurelia	
A0CJ09	Ser/Thr	I	III	VI	Paramecium tetraurelia	
A0CJW6	Ser/Thr	I	III	VI	Paramecium tetraurelia	
A0CKC0	Ser/Thr	I	III	VI	Paramecium tetraurelia	
A0CKQ4	Ser/Thr	I	III	VI	Paramecium tetraurelia	
A0CKV0	Ser/Thr	I	III	VI	Paramecium tetraurelia	
A0CLD6	Ser/Thr	I	III	VI	Paramecium tetraurelia	
A0CM89	Ser/Thr	I	III	VI	Paramecium tetraurelia	
A0CN03	Ser/Thr	I	III	VI	Paramecium tetraurelia	
A0CNF5	Ser/Thr	I	III	VI	Paramecium tetraurelia	
A0CNT8	Ser/Thr	I	III	VI	Paramecium tetraurelia	
A0CQL3	Ser/Thr	I	III	VI	Paramecium tetraurelia	
A0CSC5	Ser/Thr	I	III	VI	Paramecium tetraurelia	
A0CSK1	Ser/Thr	I	III	VI	Paramecium tetraurelia	
A0CSP9	Ser/Thr	I	III	VI	Paramecium tetraurelia	
A0CVG6	Ser/Thr	I	III	VI	Paramecium tetraurelia	
A0CW47	Ser/Thr	I	III	VI	Paramecium tetraurelia	
A0CWP3	Ser/Thr	I	III	VI	Paramecium tetraurelia	
A0CWQ7	Ser/Thr	I	III	VI	Paramecium tetraurelia	
A0CXS8	Ser/Thr	I	III	VI	Paramecium tetraurelia	
A0CYG9	Ser/Thr	I	III	VI	Paramecium tetraurelia	
A0CZA6	Ser/Thr	I	III	VI	Paramecium tetraurelia	
A0CZC4	Ser/Thr	I	III	VI	Paramecium tetraurelia	
A0CZE9	Ser/Thr	I	III	VI	Paramecium tetraurelia	
A0CZH4	Ser/Thr	I	III	VI	Paramecium tetraurelia	
A0CZN6	Ser/Thr	I	III	VI	Paramecium tetraurelia	
A0D0C4	Ser/Thr	I	III	VI	Paramecium tetraurelia	
A0D0M2	Ser/Thr	I	III	VI	Paramecium tetraurelia	
A0D0Y6	Ser/Thr	I	III	VI	Paramecium tetraurelia	
A0D1M9	Ser/Thr	I	III	VI	Paramecium tetraurelia	
A0D293	Ser/Thr	I	III	VI	Paramecium tetraurelia	
A0D2F7	Ser/Thr	I	III	VI	Paramecium tetraurelia	
A0D361	Ser/Thr	I	III	VI	Paramecium tetraurelia	
A0D4Q6	Ser/Thr	I	III	VI	Paramecium tetraurelia	
A0D4T8	Ser/Thr	I	III	VI	Paramecium tetraurelia	
A0D592	Ser/Thr	I	III	VI	Paramecium tetraurelia	
A0D5Z5	Ser/Thr	I	III	VI	Paramecium tetraurelia	
A0D753	Ser/Thr	I	III	VI	Paramecium tetraurelia	
A0D7Z8	Ser/Thr	I	III	VI	Paramecium tetraurelia	
A0D8X0	Ser/Thr	I	III	VI	Paramecium tetraurelia	
A0D9N7	Ser/Thr	I	III	VI	Paramecium tetraurelia	
A0DAC1	Ser/Thr	I	III	VI	Paramecium tetraurelia	
A0DAJ2	Ser/Thr	I	III	VI	Paramecium tetraurelia	
A0DAR9	Ser/Thr	I	III	VI	Paramecium tetraurelia	

Table S2.3. (continuation)

UniProt	Label [1]	Two	Three	Seven	Species	EC Number
A0DAT1	Ser/Thr	I	III	VI	Paramecium tetraurelia	
A0DBE9	Ser/Thr	I	III	VI	Paramecium tetraurelia	
A0DBM2	Ser/Thr	I	III	VI	Paramecium tetraurelia	
A0DBN0	Ser/Thr	I	III	VI	Paramecium tetraurelia	
A0DC30	Ser/Thr	I	III	VI	Paramecium tetraurelia	
A0DCM0	Ser/Thr	I	III	VI	Paramecium tetraurelia	
A0DD45	Ser/Thr	I	III	VI	Paramecium tetraurelia	
A0DE09	Ser/Thr	I	III	VI	Paramecium tetraurelia	
A0DEL1	Ser/Thr	I	III	VI	Paramecium tetraurelia	
A0DF62	Ser/Thr	I	III	VI	Paramecium tetraurelia	
A0DFC5	Ser/Thr	I	III	VI	Paramecium tetraurelia	
A0DFJ6	Ser/Thr	I	III	VI	Paramecium tetraurelia	
A0DGC5	Ser/Thr	I	III	VI	Paramecium tetraurelia	
A0DGI8	Ser/Thr	I	III	VI	Paramecium tetraurelia	
A0DGK1	Ser/Thr	I	III	VI	Paramecium tetraurelia	
A0DH53	Ser/Thr	I	III	VI	Paramecium tetraurelia	
A0DIB8	Ser/Thr	I	III	VI	Paramecium tetraurelia	
A0DJR9	Ser/Thr	I	III	VI	Paramecium tetraurelia	
A0DK50	Ser/Thr	I	III	VI	Paramecium tetraurelia	
A0DKG9	Ser/Thr	I	III	VI	Paramecium tetraurelia	
A0DMA4	Ser/Thr	I	III	VI	Paramecium tetraurelia	
A0DNY9	Ser/Thr	I	III	VI	Paramecium tetraurelia	
A0DP01	Ser/Thr	I	III	VI	Paramecium tetraurelia	
A0DPB9	Ser/Thr	I	III	VI	Paramecium tetraurelia	
A0DQA1	Ser/Thr	I	III	VI	Paramecium tetraurelia	
A0DQU6	Ser/Thr	I	III	VI	Paramecium tetraurelia	
A0DRR2	Ser/Thr	I	III	VI	Paramecium tetraurelia	
A0DSY0	Ser/Thr	I	III	VI	Paramecium tetraurelia	
A0DT38	Ser/Thr	I	III	VI	Paramecium tetraurelia	
A0DTJ2	Ser/Thr	I	III	VI	Paramecium tetraurelia	
A0DUI6	Ser/Thr	I	III	VI	Paramecium tetraurelia	
A0DV61	Ser/Thr	I	III	VI	Paramecium tetraurelia	
A0DVV0	Ser/Thr	I	III	VI	Paramecium tetraurelia	
A0DWU2	Ser/Thr	I	III	VI	Paramecium tetraurelia	
A0DWV0	Ser/Thr	I	III	VI	Paramecium tetraurelia	
A0DXK6	Ser/Thr	I	III	VI	Paramecium tetraurelia	
A0DYZ4	Ser/Thr	I	III	VI	Paramecium tetraurelia	
A0DZ12	Ser/Thr	I	III	VI	Paramecium tetraurelia	
A0DZ23	Ser/Thr	I	III	VI	Paramecium tetraurelia	
A0DZI9	Ser/Thr	I	III	VI	Paramecium tetraurelia	
A0DZN1	Ser/Thr	I	III	VI	Paramecium tetraurelia	
A0E091	Ser/Thr	I	III	VI	Paramecium tetraurelia	
A0E0N1	Ser/Thr	I	III	VI	Paramecium tetraurelia	
A0E0Z2	Ser/Thr	I	III	VI	Paramecium tetraurelia	
A0E1C0	Ser/Thr	I	III	VI	Paramecium tetraurelia	
A0E1E2	Ser/Thr	I	III	VI	Paramecium tetraurelia	
A0E1Z0	Ser/Thr	I	III	VI	Paramecium tetraurelia	
A0E2F9	Ser/Thr	I	III	VI	Paramecium tetraurelia	
A0E4B8	Ser/Thr	I	III	VI	Paramecium tetraurelia	
A0E569	Ser/Thr	I	III	VI	Paramecium tetraurelia	
A0E5P8	Ser/Thr	I	III	VI	Paramecium tetraurelia	
A0E675	Ser/Thr	I	III	VI	Paramecium tetraurelia	
A0E712	Ser/Thr	I	III	VI	Paramecium tetraurelia	
A0E864	Ser/Thr	I	III	VI	Paramecium tetraurelia	
A0E8K6	Ser/Thr	I	III	VI	Paramecium tetraurelia	
A0E8Y3	Ser/Thr	I	III	VI	Paramecium tetraurelia	

Table S2.3. (continuation)

UniProt	Label [1]	Two	Three	Seven	Species	EC Number
A0E900	Ser/Thr	I	III	VI	Paramecium tetraurelia	
A0EBP2	Ser/Thr	I	III	VI	Paramecium tetraurelia	
A0EBQ8	Ser/Thr	I	III	VI	Paramecium tetraurelia	
A0ED21	Ser/Thr	I	III	VI	Paramecium tetraurelia	
A0EDH4	Ser/Thr	I	III	VI	Paramecium tetraurelia	
A0EFI7	Ser/Thr	I	III	VI	Paramecium tetraurelia	
A0EG44	Ser/Thr	I	III	VI	Paramecium tetraurelia	
A0EGK5	Ser/Thr	I	III	VI	Paramecium tetraurelia	
A0EI02	Ser/Thr	I	III	VI	Paramecium tetraurelia	
O15872	Ser/Thr	I	III	VI	Paramecium tetraurelia	
Q6BFK9	Ser/Thr	I	III	VI	Paramecium tetraurelia	
Q6BG36	Ser/Thr	I	III	VI	Paramecium tetraurelia	
Q8H2C2	Ser/Thr	I	III	VI	Persea americana	2.7.11.1
Q6V7K0	Ser/Thr	I	III	VI	Phaeosphaeria nodorum	
Q6V8Y3	Ser/Thr	I	III	VI	Physcomitrella patens	
Q6V8Y5	Ser/Thr	I	III	VI	Physcomitrella patens	
Q84XC0	Ser/Thr	I	III	VI	Pisum sativum	2.7.11.1
Q4VYF5	Ser/Thr	I	III	VI	Pisum sativum	
Q4YG60	Ser/Thr	I	III	VI	Plasmodium berghei	
Q4XW47	Ser/Thr	I	III	VI	Plasmodium chabaudi	
Q8IDV5	Ser/Thr	I	III	VI	Plasmodium falciparum	2.7.11.17
A5K3L6	Ser/Thr	I	III	VI	Plasmodium vivax	
A5K822	Ser/Thr	I	III	VI	Plasmodium vivax	
A5K8Q2	Ser/Thr	I	III	VI	Plasmodium vivax	
Q7R833	Ser/Thr	I	III	VI	Plasmodium yoelii	
Q7RMR9	Ser/Thr	I	III	VI	Plasmodium yoelii	
Q5R446	Ser/Thr	I	III	VI	Pongo abelii	2.7.11.21
A0MNI9	Ser/Thr	I	III	VI	Populus trichocarpa	2.7.11.1
A0MNJ0	Ser/Thr	I	III	VI	Populus trichocarpa	2.7.11.1
A0MNJ1	Ser/Thr	I	III	VI	Populus trichocarpa	2.7.11.1
A0MNJ2	Ser/Thr	I	III	VI	Populus trichocarpa	2.7.11.1
A0MNJ4	Ser/Thr	I	III	VI	Populus trichocarpa	2.7.11.1
A0MNJ6	Ser/Thr	I	III	VI	Populus trichocarpa	2.7.11.1
A0MNJ8	Ser/Thr	I	III	VI	Populus trichocarpa	2.7.11.1
A0MNJ9	Ser/Thr	I	III	VI	Populus trichocarpa	2.7.11.1
A0MNK0	Ser/Thr	I	III	VI	Populus trichocarpa	2.7.11.1
A0MNK1	Ser/Thr	I	III	VI	Populus trichocarpa	2.7.11.1
A0MNK2	Ser/Thr	I	III	VI	Populus trichocarpa	2.7.11.1
A0MNK3	Ser/Thr	I	III	VI	Populus trichocarpa	2.7.11.1
A0MNK4	Ser/Thr	I	III	VI	Populus trichocarpa	2.7.11.1
A0MNK5	Ser/Thr	I	III	VI	Populus trichocarpa	2.7.11.1
A0MNK6	Ser/Thr	I	III	VI	Populus trichocarpa	2.7.11.1
A0MNK7	Ser/Thr	I	III	VI	Populus trichocarpa	2.7.11.1
A0MNK8	Ser/Thr	I	III	VI	Populus trichocarpa	2.7.11.1
A0MNK9	Ser/Thr	I	III	VI	Populus trichocarpa	2.7.11.1
A0MNL0	Ser/Thr	I	III	VI	Populus trichocarpa	2.7.11.1
A0MNL1	Ser/Thr	I	III	VI	Populus trichocarpa	2.7.11.1
A0MNL2	Ser/Thr	I	III	VI	Populus trichocarpa	2.7.11.1
A0MNL4	Ser/Thr	I	III	VI	Populus trichocarpa	2.7.11.1
A0MNL5	Ser/Thr	I	III	VI	Populus trichocarpa	2.7.11.1
Q3LRT3	Ser/Thr	I	III	VI	Rattus norvegicus	
Q3MHU2	Ser/Thr	I	III	VI	Rattus norvegicus	
Q4V8D4	Ser/Thr	I	III	VI	Rattus norvegicus	
Q5FVC6	Ser/Thr	I	III	VI	Rattus norvegicus	
A6ZM62	Ser/Thr	I	III	VI	Saccharomyces cerevisiae	2.7.11.21
A6ZWA4	Ser/Thr	I	III	VI	Saccharomyces cerevisiae	

Table S2.3. (continuation)

UniProt	Label [1]	Two	Three	Seven	Species	EC Number
A6ZZ66	Ser/Thr	I	III	VI	Saccharomyces cerevisiae	
Q5BXK0	Ser/Thr	I	III	VI	Schistosoma japonicum	
Q5C1Z4	Ser/Thr	I	III	VI	Schistosoma japonicum	
Q5D962	Ser/Thr	I	III	VI	Schistosoma japonicum	
Q5UES2	Ser/Thr	I	III	VI	Schistosoma mansoni	2.7.11.21
Q6LEP6	Ser/Thr	I	III	VI	Schizosaccharomyces pombe	
A7EYL0	Ser/Thr	I	III	VI	Sclerotinia sclerotiorum	
Q9Y7V4	Ser/Thr	I	III	VI	Sclerotinia sclerotiorum	
Q4W3B3	Ser/Thr	I	III	VI	Solanum lycopersicum	2.7.11.1
A1IKU3	Ser/Thr	I	III	VI	Solanum lycopersicum	
Q6XNM3	Ser/Thr	I	III	VI	Solanum lycopersicum	
Q9M726	Ser/Thr	I	III	VI	Solanum lycopersicum	
O04122	Ser/Thr	I	III	VI	Solanum tuberosum	
Q41485	Ser/Thr	I	III	VI	Solanum tuberosum	
O24342	Ser/Thr	I	III	VI	Sorghum bicolor	2.7.11.1
O24343	Ser/Thr	I	III	VI	Sorghum bicolor	2.7.11.1
O82051	Ser/Thr	I	III	VI	Sorghum bicolor	2.7.11.1
A5HNE9	Ser/Thr	I	III	VI	Sorghum bicolor	
Q9BDP8	Ser/Thr	I	III	VI	Sus scrofa	2.7.11.21
A4UTN8	Ser/Thr	I	III	VI	Sus scrofa	
Q4R1K4	Ser/Thr	I	III	VI	Sus scrofa	
Q7JFN2	Ser/Thr	I	III	VI	Sus scrofa	
Q7JFN3	Ser/Thr	I	III	VI	Sus scrofa	
Q7JFN4	Ser/Thr	I	III	VI	Sus scrofa	
A4VDB2	Ser/Thr	I	III	VI	Tetrahymena thermophila	
Q229R8	Ser/Thr	I	III	VI	Tetrahymena thermophila	
Q22C77	Ser/Thr	I	III	VI	Tetrahymena thermophila	
Q22DW7	Ser/Thr	I	III	VI	Tetrahymena thermophila	
Q22S16	Ser/Thr	I	III	VI	Tetrahymena thermophila	
Q22SK1	Ser/Thr	I	III	VI	Tetrahymena thermophila	
Q22UZ4	Ser/Thr	I	III	VI	Tetrahymena thermophila	
Q22W22	Ser/Thr	I	III	VI	Tetrahymena thermophila	
Q22XQ2	Ser/Thr	I	III	VI	Tetrahymena thermophila	
Q22Z71	Ser/Thr	I	III	VI	Tetrahymena thermophila	
Q238Z8	Ser/Thr	I	III	VI	Tetrahymena thermophila	
Q23FI7	Ser/Thr	I	III	VI	Tetrahymena thermophila	
Q23FR5	Ser/Thr	I	III	VI	Tetrahymena thermophila	
Q23ML0	Ser/Thr	I	III	VI	Tetrahymena thermophila	
Q23QZ5	Ser/Thr	I	III	VI	Tetrahymena thermophila	
Q23RV4	Ser/Thr	I	III	VI	Tetrahymena thermophila	
Q23WP1	Ser/Thr	I	III	VI	Tetrahymena thermophila	
Q240T9	Ser/Thr	I	III	VI	Tetrahymena thermophila	
Q240Y9	Ser/Thr	I	III	VI	Tetrahymena thermophila	
Q248B0	Ser/Thr	I	III	VI	Tetrahymena thermophila	
Q24HK9	Ser/Thr	I	III	VI	Tetrahymena thermophila	
Q4RL54	Ser/Thr	I	III	VI	Tetraodon nigroviridis	2.7.11.21
Q4RM17	Ser/Thr	I	III	VI	Tetraodon nigroviridis	2.7.11.21
Q4RK91	Ser/Thr	I	III	VI	Tetraodon nigroviridis	
Q4RNM0	Ser/Thr	I	III	VI	Tetraodon nigroviridis	
Q4RTH5	Ser/Thr	I	III	VI	Tetraodon nigroviridis	
Q4RVL2	Ser/Thr	I	III	VI	Tetraodon nigroviridis	
Q4S0J0	Ser/Thr	I	III	VI	Tetraodon nigroviridis	
Q4S6F4	Ser/Thr	I	III	VI	Tetraodon nigroviridis	
Q4S8A8	Ser/Thr	I	III	VI	Tetraodon nigroviridis	
Q4SG03	Ser/Thr	I	III	VI	Tetraodon nigroviridis	
Q4SIP8	Ser/Thr	I	III	VI	Tetraodon nigroviridis	

Table S2.3. (continuation)

UniProt	Label [1]	Two	Three	Seven	Species	EC Number
Q4SL09	Ser/Thr	I	III	VI	Tetraodon nigroviridis	
Q4SMN8	Ser/Thr	I	III	VI	Tetraodon nigroviridis	
Q4SPC4	Ser/Thr	I	III	VI	Tetraodon nigroviridis	
Q4SQH4	Ser/Thr	I	III	VI	Tetraodon nigroviridis	
Q4SRC8	Ser/Thr	I	III	VI	Tetraodon nigroviridis	
Q4STJ7	Ser/Thr	I	III	VI	Tetraodon nigroviridis	
Q4SUU0	Ser/Thr	I	III	VI	Tetraodon nigroviridis	
Q4T208	Ser/Thr	I	III	VI	Tetraodon nigroviridis	
Q4T2N1	Ser/Thr	I	III	VI	Tetraodon nigroviridis	
Q4T8P5	Ser/Thr	I	III	VI	Tetraodon nigroviridis	
Q4UEA3	Ser/Thr	I	III	VI	Theileria annulata	2.7.1.123
Q4N1C8	Ser/Thr	I	III	VI	Theileria parva	2.7.1.-
Q4N591	Ser/Thr	I	III	VI	Theileria parva	
Q96WI7	Ser/Thr	I	III	VI	Trichoderma reesei	
A2DIN7	Ser/Thr	I	III	VI	Trichomonas vaginalis	2.7.11.21
A2EB11	Ser/Thr	I	III	VI	Trichomonas vaginalis	2.7.11.21
A2FN87	Ser/Thr	I	III	VI	Trichomonas vaginalis	2.7.11.21
A2D7H7	Ser/Thr	I	III	VI	Trichomonas vaginalis	
A2D7M3	Ser/Thr	I	III	VI	Trichomonas vaginalis	
A2DBR9	Ser/Thr	I	III	VI	Trichomonas vaginalis	
A2DBY9	Ser/Thr	I	III	VI	Trichomonas vaginalis	
A2DCS5	Ser/Thr	I	III	VI	Trichomonas vaginalis	
A2DHE8	Ser/Thr	I	III	VI	Trichomonas vaginalis	
A2DHM8	Ser/Thr	I	III	VI	Trichomonas vaginalis	
A2DHV0	Ser/Thr	I	III	VI	Trichomonas vaginalis	
A2DI34	Ser/Thr	I	III	VI	Trichomonas vaginalis	
A2DLZ6	Ser/Thr	I	III	VI	Trichomonas vaginalis	
A2DPD2	Ser/Thr	I	III	VI	Trichomonas vaginalis	
A2DPY7	Ser/Thr	I	III	VI	Trichomonas vaginalis	
A2DRR6	Ser/Thr	I	III	VI	Trichomonas vaginalis	
A2DTF0	Ser/Thr	I	III	VI	Trichomonas vaginalis	
A2DW97	Ser/Thr	I	III	VI	Trichomonas vaginalis	
A2DY61	Ser/Thr	I	III	VI	Trichomonas vaginalis	
A2E0G9	Ser/Thr	I	III	VI	Trichomonas vaginalis	
A2E2X1	Ser/Thr	I	III	VI	Trichomonas vaginalis	
A2E3M4	Ser/Thr	I	III	VI	Trichomonas vaginalis	
A2E6I9	Ser/Thr	I	III	VI	Trichomonas vaginalis	
A2E705	Ser/Thr	I	III	VI	Trichomonas vaginalis	
A2E7I6	Ser/Thr	I	III	VI	Trichomonas vaginalis	
A2E9I7	Ser/Thr	I	III	VI	Trichomonas vaginalis	
A2EA15	Ser/Thr	I	III	VI	Trichomonas vaginalis	
A2EAS8	Ser/Thr	I	III	VI	Trichomonas vaginalis	
A2EBI4	Ser/Thr	I	III	VI	Trichomonas vaginalis	
A2ECN9	Ser/Thr	I	III	VI	Trichomonas vaginalis	
A2EI81	Ser/Thr	I	III	VI	Trichomonas vaginalis	
A2EJD6	Ser/Thr	I	III	VI	Trichomonas vaginalis	
A2EKR5	Ser/Thr	I	III	VI	Trichomonas vaginalis	
A2EM22	Ser/Thr	I	III	VI	Trichomonas vaginalis	
A2EMW5	Ser/Thr	I	III	VI	Trichomonas vaginalis	
A2EN63	Ser/Thr	I	III	VI	Trichomonas vaginalis	
A2EP26	Ser/Thr	I	III	VI	Trichomonas vaginalis	
A2ERI4	Ser/Thr	I	III	VI	Trichomonas vaginalis	
A2ESJ5	Ser/Thr	I	III	VI	Trichomonas vaginalis	
A2ESP5	Ser/Thr	I	III	VI	Trichomonas vaginalis	
A2ESQ5	Ser/Thr	I	III	VI	Trichomonas vaginalis	
A2ET53	Ser/Thr	I	III	VI	Trichomonas vaginalis	

Table S2.3. (continuation)

UniProt	Label [1]	Two	Three	Seven	Species	EC Number
A2ETF3	Ser/Thr	I	III	VI	Trichomonas vaginalis	
A2ETG3	Ser/Thr	I	III	VI	Trichomonas vaginalis	
A2ETK3	Ser/Thr	I	III	VI	Trichomonas vaginalis	
A2EU91	Ser/Thr	I	III	VI	Trichomonas vaginalis	
A2EWJ0	Ser/Thr	I	III	VI	Trichomonas vaginalis	
A2EWU5	Ser/Thr	I	III	VI	Trichomonas vaginalis	
A2EYI5	Ser/Thr	I	III	VI	Trichomonas vaginalis	
A2EZM5	Ser/Thr	I	III	VI	Trichomonas vaginalis	
A2F3L7	Ser/Thr	I	III	VI	Trichomonas vaginalis	
A2F496	Ser/Thr	I	III	VI	Trichomonas vaginalis	
A2F5P8	Ser/Thr	I	III	VI	Trichomonas vaginalis	
A2F807	Ser/Thr	I	III	VI	Trichomonas vaginalis	
A2F9P2	Ser/Thr	I	III	VI	Trichomonas vaginalis	
A2FA80	Ser/Thr	I	III	VI	Trichomonas vaginalis	
A2FBB6	Ser/Thr	I	III	VI	Trichomonas vaginalis	
A2FCN9	Ser/Thr	I	III	VI	Trichomonas vaginalis	
A2FGW3	Ser/Thr	I	III	VI	Trichomonas vaginalis	
A2FHU2	Ser/Thr	I	III	VI	Trichomonas vaginalis	
A2FK34	Ser/Thr	I	III	VI	Trichomonas vaginalis	
A2FMX7	Ser/Thr	I	III	VI	Trichomonas vaginalis	
A2FQP0	Ser/Thr	I	III	VI	Trichomonas vaginalis	
A2FT91	Ser/Thr	I	III	VI	Trichomonas vaginalis	
A2FV14	Ser/Thr	I	III	VI	Trichomonas vaginalis	
A2FZA3	Ser/Thr	I	III	VI	Trichomonas vaginalis	
A2G1L2	Ser/Thr	I	III	VI	Trichomonas vaginalis	
A2G3D8	Ser/Thr	I	III	VI	Trichomonas vaginalis	
A2GAV1	Ser/Thr	I	III	VI	Trichomonas vaginalis	
A2GB48	Ser/Thr	I	III	VI	Trichomonas vaginalis	
A2GCT3	Ser/Thr	I	III	VI	Trichomonas vaginalis	
Q2PET0	Ser/Thr	I	III	VI	Trifolium pratense	2.7.11.1
Q41592	Ser/Thr	I	III	VI	Triticum aestivum	2.7.11.1
Q385X0	Ser/Thr	I	III	VI	Trypanosoma brucei	2.7.1.-
Q388P9	Ser/Thr	I	III	VI	Trypanosoma brucei	2.7.1.-
Q57YE4	Ser/Thr	I	III	VI	Trypanosoma brucei	2.7.1.-
Q57ZV8	Ser/Thr	I	III	VI	Trypanosoma brucei	2.7.1.-
Q582S9	Ser/Thr	I	III	VI	Trypanosoma brucei	2.7.1.-
Q57V67	Ser/Thr	I	III	VI	Trypanosoma brucei	2.7.1.37; 2.7.11.1
Q4CNP2	Ser/Thr	I	III	VI	Trypanosoma cruzi	
Q4CQ74	Ser/Thr	I	III	VI	Trypanosoma cruzi	
Q4D3R7	Ser/Thr	I	III	VI	Trypanosoma cruzi	
Q4DDT6	Ser/Thr	I	III	VI	Trypanosoma cruzi	
Q4DPG9	Ser/Thr	I	III	VI	Trypanosoma cruzi	
Q4E3J2	Ser/Thr	I	III	VI	Trypanosoma cruzi	
Q4E5I0	Ser/Thr	I	III	VI	Trypanosoma cruzi	
A7TKN7	Ser/Thr	I	III	VI	Vanderwaltozyma polyspora	
Q4VYF6	Ser/Thr	I	III	VI	Vicia faba	
A7BJ79	Ser/Thr	I	III	VI	Vigna unguiculata	2.7.11.1
A5AWY5	Ser/Thr	I	III	VI	Vitis vinifera	2.7.11.1
A5BHI9	Ser/Thr	I	III	VI	Vitis vinifera	2.7.11.1
A5BJJ8	Ser/Thr	I	III	VI	Vitis vinifera	2.7.11.1
A5BKH1	Ser/Thr	I	III	VI	Vitis vinifera	2.7.11.1
A5BQA6	Ser/Thr	I	III	VI	Vitis vinifera	2.7.11.1
A5BZV3	Ser/Thr	I	III	VI	Vitis vinifera	2.7.11.1
A5C5K7	Ser/Thr	I	III	VI	Vitis vinifera	2.7.11.1
A5C5T4	Ser/Thr	I	III	VI	Vitis vinifera	2.7.11.1
A5AIM3	Ser/Thr	I	III	VI	Vitis vinifera	

Table S2.3. (continuation)

UniProt	Label [1]	Two	Three	Seven	Species	EC Number
A5AQ39	Ser/Thr	I	III	VI	Vitis vinifera	
A5B461	Ser/Thr	I	III	VI	Vitis vinifera	
A5BPE0	Ser/Thr	I	III	VI	Vitis vinifera	
A5C257	Ser/Thr	I	III	VI	Vitis vinifera	
Q91821	Ser/Thr	I	III	VI	Xenopus laevis	2.7.11.1
Q90XS4	Ser/Thr	I	III	VI	Xenopus laevis	2.7.11.21
Q2TAE5	Ser/Thr	I	III	VI	Xenopus laevis	
Q5U5B2	Ser/Thr	I	III	VI	Xenopus laevis	
Q5U5E3	Ser/Thr	I	III	VI	Xenopus laevis	
Q66KS2	Ser/Thr	I	III	VI	Xenopus laevis	
Q6GM90	Ser/Thr	I	III	VI	Xenopus laevis	
Q6INT7	Ser/Thr	I	III	VI	Xenopus laevis	
Q6PA64	Ser/Thr	I	III	VI	Xenopus laevis	
Q7ZYL7	Ser/Thr	I	III	VI	Xenopus laevis	
Q801N3	Ser/Thr	I	III	VI	Xenopus laevis	
Q804T1	Ser/Thr	I	III	VI	Xenopus laevis	
Q804T2	Ser/Thr	I	III	VI	Xenopus laevis	
Q8AYR2	Ser/Thr	I	III	VI	Xenopus laevis	
Q8AYR3	Ser/Thr	I	III	VI	Xenopus laevis	
Q8QGV3	Ser/Thr	I	III	VI	Xenopus laevis	
Q8UVW8	Ser/Thr	I	III	VI	Xenopus laevis	
Q9DFZ8	Ser/Thr	I	III	VI	Xenopus laevis	
Q9DFZ9	Ser/Thr	I	III	VI	Xenopus laevis	
Q9DG00	Ser/Thr	I	III	VI	Xenopus laevis	
Q9DG01	Ser/Thr	I	III	VI	Xenopus laevis	
Q28GW8	Ser/Thr	I	III	VI	Xenopus tropicalis	2.7.11.1
Q0P4W7	Ser/Thr	I	III	VI	Xenopus tropicalis	
Q28C86	Ser/Thr	I	III	VI	Xenopus tropicalis	
Q28FC8	Ser/Thr	I	III	VI	Xenopus tropicalis	
Q28FG8	Ser/Thr	I	III	VI	Xenopus tropicalis	
Q28HZ5	Ser/Thr	I	III	VI	Xenopus tropicalis	
Q5BL60	Ser/Thr	I	III	VI	Xenopus tropicalis	
Q5BL77	Ser/Thr	I	III	VI	Xenopus tropicalis	
Q6C7R4	Ser/Thr	I	III	VI	Yarrowia lipolytica	2.7.11.21
Q6CAK0	Ser/Thr	I	III	VI	Yarrowia lipolytica	
A1Z195	Ser/Thr	I	III	VI	Zea mays	2.7.11.1
A1Z196	Ser/Thr	I	III	VI	Zea mays	2.7.11.1
Q9SEG2	Ser/Thr	I	III	VI	Zea mays	2.7.11.1
Q6RXY1	Ser/Thr	I	III	VI	Zea mays	
Q17ND6	Ser/Thr	I	III	VII	Aedes aegypti	2.7.11.1
Q535V4	Ser/Thr	I	III	VII	Aedes aegypti	2.7.11.1
Q170R4	Ser/Thr	I	III	VII	Aedes aegypti	2.7.11.12
Q0IGD3	Ser/Thr	I	III	VII	Aedes aegypti	
Q16F70	Ser/Thr	I	III	VII	Aedes aegypti	
Q16JU3	Ser/Thr	I	III	VII	Aedes aegypti	
Q16NW2	Ser/Thr	I	III	VII	Aedes aegypti	
Q176L3	Ser/Thr	I	III	VII	Aedes aegypti	
Q177I7	Ser/Thr	I	III	VII	Aedes aegypti	
Q178W2	Ser/Thr	I	III	VII	Aedes aegypti	
Q179S5	Ser/Thr	I	III	VII	Aedes aegypti	
Q17JG1	Ser/Thr	I	III	VII	Aedes aegypti	
Q1HQW4	Ser/Thr	I	III	VII	Aedes aegypti	
A6R8K2	Ser/Thr	I	III	VII	Ajellomyces capsulata	
A6RA35	Ser/Thr	I	III	VII	Ajellomyces capsulata	
A6QRR5	Ser/Thr	I	III	VII	Ajellomyces capsulatus	
A6QRT1	Ser/Thr	I	III	VII	Ajellomyces capsulatus	

Table S2.3. (continuation)

UniProt	Label [1]	Two	Three	Seven	Species	EC Number
A6QTK8	Ser/Thr	I	III	VII	Ajellomyces capsulatus	
A6QTZ6	Ser/Thr	I	III	VII	Ajellomyces capsulatus	
A6R2N1	Ser/Thr	I	III	VII	Ajellomyces capsulatus	
A6R5Y2	Ser/Thr	I	III	VII	Ajellomyces capsulatus	
A6RCS9	Ser/Thr	I	III	VII	Ajellomyces capsulatus	
A6RGF6	Ser/Thr	I	III	VII	Ajellomyces capsulatus	
A6RHI7	Ser/Thr	I	III	VII	Ajellomyces capsulatus	
O97114	Ser/Thr	I	III	VII	Amblyomma americanum	
O97115	Ser/Thr	I	III	VII	Amblyomma americanum	
O97116	Ser/Thr	I	III	VII	Amblyomma americanum	
Q4R0Q0	Ser/Thr	I	III	VII	Amblyomma hebraeum	2.7.1.37
Q4R0Q1	Ser/Thr	I	III	VII	Amblyomma hebraeum	2.7.1.37
Q4R0Q2	Ser/Thr	I	III	VII	Amblyomma hebraeum	2.7.1.37
A5A3E2	Ser/Thr	I	III	VII	Ameiurus melas	
Q16933	Ser/Thr	I	III	VII	Ancylostoma caninum	
Q7QJ74	Ser/Thr	I	III	VII	Anopheles gambiae	2.7.11.1
Q5TVN7	Ser/Thr	I	III	VII	Anopheles gambiae	
Q7PQK8	Ser/Thr	I	III	VII	Anopheles gambiae	
Q7PWT0	Ser/Thr	I	III	VII	Anopheles gambiae	
Q7PXF7	Ser/Thr	I	III	VII	Anopheles gambiae	
Q7PZC6	Ser/Thr	I	III	VII	Anopheles gambiae	
Q7PZQ1	Ser/Thr	I	III	VII	Anopheles gambiae	
Q7Q1G7	Ser/Thr	I	III	VII	Anopheles gambiae	
Q7Q3T7	Ser/Thr	I	III	VII	Anopheles gambiae	
Q7Q8J6	Ser/Thr	I	III	VII	Anopheles gambiae	
Q6E6E4	Ser/Thr	I	III	VII	Antonospora locustae	
Q8SSX4	Ser/Thr	I	III	VII	Apis mellifera	2.7.11.12
Q9NAS6	Ser/Thr	I	III	VII	Apis mellifera	
Q9BMX7	Ser/Thr	I	III	VII	Aplysia californica	2.7.11.1
Q6USR9	Ser/Thr	I	III	VII	Aplysia californica	2.7.11.12
Q16957	Ser/Thr	I	III	VII	Aplysia californica	
Q16958	Ser/Thr	I	III	VII	Aplysia californica	
Q9BMX6	Ser/Thr	I	III	VII	Aplysia californica	
O82649	Ser/Thr	I	III	VII	Arabidopsis thaliana	2.7.1.37
Q2QAV0	Ser/Thr	I	III	VII	Arabidopsis thaliana	2.7.11.1
Q4V3C8	Ser/Thr	I	III	VII	Arabidopsis thaliana	2.7.11.1
Q9XF67	Ser/Thr	I	III	VII	Arabidopsis thaliana	2.7.11.1
Q0V851	Ser/Thr	I	III	VII	Arabidopsis thaliana	
Q8W582	Ser/Thr	I	III	VII	Arabidopsis thaliana	
Q9LPH2	Ser/Thr	I	III	VII	Arabidopsis thaliana	
Q9ZRF7	Ser/Thr	I	III	VII	Arabidopsis thaliana	
Q9GQB3	Ser/Thr	I	III	VII	Artemia sanfranciscana	2.7.11.1
Q753H7	Ser/Thr	I	III	VII	Ashbya gossypii	
Q755B5	Ser/Thr	I	III	VII	Ashbya gossypii	
Q757U5	Ser/Thr	I	III	VII	Ashbya gossypii	
Q758E1	Ser/Thr	I	III	VII	Ashbya gossypii	
Q759V5	Ser/Thr	I	III	VII	Ashbya gossypii	
Q75BF3	Ser/Thr	I	III	VII	Ashbya gossypii	
Q75DP5	Ser/Thr	I	III	VII	Ashbya gossypii	
Q9M3V7	Ser/Thr	I	III	VII	Asparagus officinalis	
A1C6B1	Ser/Thr	I	III	VII	Aspergillus clavatus	
A1C6Q4	Ser/Thr	I	III	VII	Aspergillus clavatus	
A1CAK0	Ser/Thr	I	III	VII	Aspergillus clavatus	
A1CKA9	Ser/Thr	I	III	VII	Aspergillus clavatus	
A1CR23	Ser/Thr	I	III	VII	Aspergillus clavatus	
A1CSI5	Ser/Thr	I	III	VII	Aspergillus clavatus	

Table S2.3. (continuation)

UniProt	Label [1]	Two	Three	Seven	Species	EC Number
A1CTI4	Ser/Thr	I	III	VII	Aspergillus clavatus	
Q4WR13	Ser/Thr	I	III	VII	Aspergillus fumigatus	2.7.1.-
Q4WXM9	Ser/Thr	I	III	VII	Aspergillus fumigatus	2.7.1.-
Q4X1A8	Ser/Thr	I	III	VII	Aspergillus fumigatus	2.7.1.-
Q4X262	Ser/Thr	I	III	VII	Aspergillus fumigatus	2.7.1.-
Q4WJD1	Ser/Thr	I	III	VII	Aspergillus fumigatus	2.7.11.1
Q4X0V1	Ser/Thr	I	III	VII	Aspergillus fumigatus	2.7.11.1
Q4WMP1	Ser/Thr	I	III	VII	Aspergillus fumigatus	
Q70KQ0	Ser/Thr	I	III	VII	Aspergillus fumigatus	
Q8J129	Ser/Thr	I	III	VII	Aspergillus fumigatus	
Q96UM3	Ser/Thr	I	III	VII	Aspergillus fumigatus	
A2QCP3	Ser/Thr	I	III	VII	Aspergillus niger	
A2QD52	Ser/Thr	I	III	VII	Aspergillus niger	
A2QNB6	Ser/Thr	I	III	VII	Aspergillus niger	
A2QWG0	Ser/Thr	I	III	VII	Aspergillus niger	
A2R7K5	Ser/Thr	I	III	VII	Aspergillus niger	
A2RB37	Ser/Thr	I	III	VII	Aspergillus niger	
P87077	Ser/Thr	I	III	VII	Aspergillus niger	
Q2U061	Ser/Thr	I	III	VII	Aspergillus oryzae	
Q2U6J7	Ser/Thr	I	III	VII	Aspergillus oryzae	
Q2UEY6	Ser/Thr	I	III	VII	Aspergillus oryzae	
Q2UL56	Ser/Thr	I	III	VII	Aspergillus oryzae	
Q2UNA7	Ser/Thr	I	III	VII	Aspergillus oryzae	
Q0CHH8	Ser/Thr	I	III	VII	Aspergillus terreus	
Q0CHZ9	Ser/Thr	I	III	VII	Aspergillus terreus	
Q0CP51	Ser/Thr	I	III	VII	Aspergillus terreus	
Q0CTK2	Ser/Thr	I	III	VII	Aspergillus terreus	
Q0CXS3	Ser/Thr	I	III	VII	Aspergillus terreus	
Q0CYS7	Ser/Thr	I	III	VII	Aspergillus terreus	
Q76BX2	Ser/Thr	I	III	VII	Asterina pectinifera	
A5A493	Ser/Thr	I	III	VII	Avena sativa	
Q43380	Ser/Thr	I	III	VII	Avena sativa	
A7AWS2	Ser/Thr	I	III	VII	Babesia bovis	2.7.11.1
A7AWX2	Ser/Thr	I	III	VII	Babesia bovis	2.7.11.1
A7ANK3	Ser/Thr	I	III	VII	Babesia bovis	
Q12741	Ser/Thr	I	III	VII	Blastocladiella emersonii	
Q7LIG8	Ser/Thr	I	III	VII	Blastocladiella emersonii	
Q9HGU6	Ser/Thr	I	III	VII	Blumeria graminis	
Q8ST45	Ser/Thr	I	III	VII	Bombyx mori	2.7.11.12
Q8T9W9	Ser/Thr	I	III	VII	Bombyx mori	2.7.11.12
A5JNM1	Ser/Thr	I	III	VII	Bombyx mori	
A7MB74	Ser/Thr	I	III	VII	Bos taurus	2.7.11.1
A2VDX4	Ser/Thr	I	III	VII	Bos taurus	
A6QNX4	Ser/Thr	I	III	VII	Bos taurus	
A6QP95	Ser/Thr	I	III	VII	Bos taurus	
Q0VD39	Ser/Thr	I	III	VII	Bos taurus	
Q9HGS0	Ser/Thr	I	III	VII	Botryotinia fuckeliana	
Q7ZTW4	Ser/Thr	I	III	VII	Brachydanio rerio	2.7.11.1
Q802Y7	Ser/Thr	I	III	VII	Brachydanio rerio	2.7.11.1
O01583	Ser/Thr	I	III	VII	Caenorhabditis elegans	2.7.11.1
P92199	Ser/Thr	I	III	VII	Caenorhabditis elegans	2.7.11.1
Q2PJ68	Ser/Thr	I	III	VII	Caenorhabditis elegans	2.7.11.1
Q9NAH6	Ser/Thr	I	III	VII	Caenorhabditis elegans	2.7.11.1
Q7JP68	Ser/Thr	I	III	VII	Caenorhabditis elegans	2.7.11.11
O76360	Ser/Thr	I	III	VII	Caenorhabditis elegans	2.7.11.12
Q21694	Ser/Thr	I	III	VII	Caenorhabditis elegans	

Table S2.3. (continuation)

UniProt	Label [1]	Two	Three	Seven	Species	EC Number
Q8MQE2	Ser/Thr	I	III	VII	Caenorhabditis elegans	
Q5A3P6	Ser/Thr	I	III	VII	Candida albicans	2.7.11.1
Q5AHG6	Ser/Thr	I	III	VII	Candida albicans	2.7.11.1
Q59QB9	Ser/Thr	I	III	VII	Candida albicans	
Q59Z23	Ser/Thr	I	III	VII	Candida albicans	
Q5AAN6	Ser/Thr	I	III	VII	Candida albicans	
Q5AP71	Ser/Thr	I	III	VII	Candida albicans	
Q9HEW0	Ser/Thr	I	III	VII	Candida albicans	
Q9P932	Ser/Thr	I	III	VII	Candida albicans	
Q6FJ83	Ser/Thr	I	III	VII	Candida glabrata	
Q6FMJ9	Ser/Thr	I	III	VII	Candida glabrata	
Q6FN22	Ser/Thr	I	III	VII	Candida glabrata	
Q6FQB9	Ser/Thr	I	III	VII	Candida glabrata	
Q6FSP1	Ser/Thr	I	III	VII	Candida glabrata	
Q6FT84	Ser/Thr	I	III	VII	Candida glabrata	
Q6FTT2	Ser/Thr	I	III	VII	Candida glabrata	
Q6FXN8	Ser/Thr	I	III	VII	Candida glabrata	
Q56SE2	Ser/Thr	I	III	VII	Canis familiaris	
Q2GVX0	Ser/Thr	I	III	VII	Chaetomium globosum	
Q2GXX4	Ser/Thr	I	III	VII	Chaetomium globosum	
Q2H1D1	Ser/Thr	I	III	VII	Chaetomium globosum	
Q2H4D3	Ser/Thr	I	III	VII	Chaetomium globosum	
Q2H4R5	Ser/Thr	I	III	VII	Chaetomium globosum	
Q2H8S8	Ser/Thr	I	III	VII	Chaetomium globosum	
Q2HEF5	Ser/Thr	I	III	VII	Chaetomium globosum	
Q2HEF7	Ser/Thr	I	III	VII	Chaetomium globosum	
Q6UPR4	Ser/Thr	I	III	VII	Chlamydomonas reinhardtii	2.7.1.-
Q695H0	Ser/Thr	I	III	VII	Chlamydomonas reinhardtii	2.7.1.37
Q0PKT8	Ser/Thr	I	III	VII	Colletotrichum gloeosporioides	
O42793	Ser/Thr	I	III	VII	Colletotrichum trifolii	
O42795	Ser/Thr	I	III	VII	Colletotrichum trifolii	
Q96VR1	Ser/Thr	I	III	VII	Cryphonectria parasitica	
Q55HT7	Ser/Thr	I	III	VII	Cryptococcus neoformans	
Q55MZ9	Ser/Thr	I	III	VII	Cryptococcus neoformans	
Q55U80	Ser/Thr	I	III	VII	Cryptococcus neoformans	
Q55WE5	Ser/Thr	I	III	VII	Cryptococcus neoformans	
Q55ZX5	Ser/Thr	I	III	VII	Cryptococcus neoformans	
Q5K7C2	Ser/Thr	I	III	VII	Cryptococcus neoformans	
Q5KAN2	Ser/Thr	I	III	VII	Cryptococcus neoformans	
Q5KBC8	Ser/Thr	I	III	VII	Cryptococcus neoformans	
Q5KI66	Ser/Thr	I	III	VII	Cryptococcus neoformans	
Q5KJV3	Ser/Thr	I	III	VII	Cryptococcus neoformans	
Q5KM47	Ser/Thr	I	III	VII	Cryptococcus neoformans	
Q5KP84	Ser/Thr	I	III	VII	Cryptococcus neoformans	
Q8J0I6	Ser/Thr	I	III	VII	Cryptococcus neoformans	
Q8NIK9	Ser/Thr	I	III	VII	Cryptococcus neoformans	
Q8NIL1	Ser/Thr	I	III	VII	Cryptococcus neoformans	
Q8NKF8	Ser/Thr	I	III	VII	Cryptococcus neoformans	
Q9HFW0	Ser/Thr	I	III	VII	Cryptococcus neoformans	
Q5CWD4	Ser/Thr	I	III	VII	Cryptosporidium parvum	
Q8MMZ6	Ser/Thr	I	III	VII	Cryptosporidium parvum	
Q2V2M2	Ser/Thr	I	III	VII	Cyprinus carpio	
Q2V2M3	Ser/Thr	I	III	VII	Cyprinus carpio	
Q2V2M4	Ser/Thr	I	III	VII	Cyprinus carpio	
Q98UH5	Ser/Thr	I	III	VII	Cyprinus carpio	
Q98UH6	Ser/Thr	I	III	VII	Cyprinus carpio	

Table S2.3. (continuation)

UniProt	Label [1]	Two	Three	Seven	Species	EC Number
Q5TZ37	Ser/Thr	I	III	VII	Danio rerio	2.7.11.1
Q5TZ42	Ser/Thr	I	III	VII	Danio rerio	2.7.11.1
Q90Y37	Ser/Thr	I	III	VII	Danio rerio	2.7.11.1
A2AVJ3	Ser/Thr	I	III	VII	Danio rerio	2.7.11.12
Q7T2E5	Ser/Thr	I	III	VII	Danio rerio	2.7.11.12
Q1XHL7	Ser/Thr	I	III	VII	Danio rerio	2.7.11.14; 2.7.11.16
Q49HM9	Ser/Thr	I	III	VII	Danio rerio	2.7.11.14; 2.7.11.16
Q08BB4	Ser/Thr	I	III	VII	Danio rerio	
Q08BG3	Ser/Thr	I	III	VII	Danio rerio	
Q08BW4	Ser/Thr	I	III	VII	Danio rerio	
Q08CJ6	Ser/Thr	I	III	VII	Danio rerio	
Q1XHM0	Ser/Thr	I	III	VII	Danio rerio	
Q3ZB92	Ser/Thr	I	III	VII	Danio rerio	
Q49HM8	Ser/Thr	I	III	VII	Danio rerio	
Q49HN0	Ser/Thr	I	III	VII	Danio rerio	
Q567A3	Ser/Thr	I	III	VII	Danio rerio	
Q6DBV8	Ser/Thr	I	III	VII	Danio rerio	
Q6NZV1	Ser/Thr	I	III	VII	Danio rerio	
Q7T374	Ser/Thr	I	III	VII	Danio rerio	
Q6BHW6	Ser/Thr	I	III	VII	Debaryomyces hansenii	
Q6BI24	Ser/Thr	I	III	VII	Debaryomyces hansenii	
Q6BIK8	Ser/Thr	I	III	VII	Debaryomyces hansenii	
Q6BK94	Ser/Thr	I	III	VII	Debaryomyces hansenii	
Q6BPN3	Ser/Thr	I	III	VII	Debaryomyces hansenii	
Q6BSR0	Ser/Thr	I	III	VII	Debaryomyces hansenii	
Q6BWC6	Ser/Thr	I	III	VII	Debaryomyces hansenii	
Q06BQ9	Ser/Thr	I	III	VII	Diabrotica virgifera	2.7.11.12
Q54IF2	Ser/Thr	I	III	VII	Dictyostelium discoideum	2.7.11.1
Q54PK9	Ser/Thr	I	III	VII	Dictyostelium discoideum	2.7.11.1
Q54UZ1	Ser/Thr	I	III	VII	Dictyostelium discoideum	2.7.11.1
Q54XJ4	Ser/Thr	I	III	VII	Dictyostelium discoideum	2.7.11.1
Q8MYF1	Ser/Thr	I	III	VII	Dictyostelium discoideum	2.7.11.1
Q9U567	Ser/Thr	I	III	VII	Dictyostelium discoideum	
Q9VA47	Ser/Thr	I	III	VII	Drosophila melanogaster	2.7.11.-
Q9VUQ9	Ser/Thr	I	III	VII	Drosophila melanogaster	2.7.11.-
Q9VL34	Ser/Thr	I	III	VII	Drosophila melanogaster	2.7.11.-; 2.7.11.12
P91656	Ser/Thr	I	III	VII	Drosophila melanogaster	2.7.11.1
Q94533	Ser/Thr	I	III	VII	Drosophila melanogaster	2.7.11.1
Q961D4	Ser/Thr	I	III	VII	Drosophila melanogaster	2.7.11.1
Q9U779	Ser/Thr	I	III	VII	Drosophila melanogaster	2.7.11.1
Q9VXE3	Ser/Thr	I	III	VII	Drosophila melanogaster	2.7.11.1
O96630	Ser/Thr	I	III	VII	Drosophila melanogaster	
Q194S5	Ser/Thr	I	III	VII	Drosophila melanogaster	
Q53XD8	Ser/Thr	I	III	VII	Drosophila melanogaster	
Q8SWT5	Ser/Thr	I	III	VII	Drosophila melanogaster	
Q9VWQ2	Ser/Thr	I	III	VII	Drosophila melanogaster	
Q29CB0	Ser/Thr	I	III	VII	Drosophila pseudoobscura	2.7.11.-
Q29CB1	Ser/Thr	I	III	VII	Drosophila pseudoobscura	2.7.11.-
Q29IT7	Ser/Thr	I	III	VII	Drosophila pseudoobscura	2.7.11.-
Q29NH6	Ser/Thr	I	III	VII	Drosophila pseudoobscura	2.7.11.-
Q2M1D0	Ser/Thr	I	III	VII	Drosophila pseudoobscura	2.7.11.-
Q29E52	Ser/Thr	I	III	VII	Drosophila pseudoobscura	2.7.11.1
Q29LM5	Ser/Thr	I	III	VII	Drosophila pseudoobscura	2.7.11.12
Q295G1	Ser/Thr	I	III	VII	Drosophila pseudoobscura	
Q29PJ0	Ser/Thr	I	III	VII	Drosophila pseudoobscura	
Q6XIA7	Ser/Thr	I	III	VII	Drosophila yakuba	

Table S2.3. (continuation)

UniProt	Label [1]	Two	Three	Seven	Species	EC Number
Q8MMZ5	Ser/Thr	I	III	VII	Eimeria maxima	
Q8MMZ8	Ser/Thr	I	III	VII	Eimeria tenella	
Q5B0F7	Ser/Thr	I	III	VII	Emericella nidulans	
Q5B0W5	Ser/Thr	I	III	VII	Emericella nidulans	
Q5B3A0	Ser/Thr	I	III	VII	Emericella nidulans	
Q5B413	Ser/Thr	I	III	VII	Emericella nidulans	
Q5BH36	Ser/Thr	I	III	VII	Emericella nidulans	
Q8NIK8	Ser/Thr	I	III	VII	Emericella nidulans	
Q9P472	Ser/Thr	I	III	VII	Emericella nidulans	
Q8SSJ0	Ser/Thr	I	III	VII	Encephalitozoon cuniculi	
Q8MMN3	Ser/Thr	I	III	VII	Encephalitozoon intestinalis	
Q26334	Ser/Thr	I	III	VII	Entamoeba histolytica	
Q761W9	Ser/Thr	I	III	VII	Entamoeba histolytica	
Q8WQH7	Ser/Thr	I	III	VII	Entamoeba histolytica	
Q9UUS9	Ser/Thr	I	III	VII	Erysiphe graminis	
Q9SXP9	Ser/Thr	I	III	VII	Euglena gracilis	
Q5Q0U5	Ser/Thr	I	III	VII	Fundulus heteroclitus	2.7.11.1
Q5ZLW5	Ser/Thr	I	III	VII	Gallus gallus	2.7.11.1
Q6U1I9	Ser/Thr	I	III	VII	Gallus gallus	2.7.11.1
O73685	Ser/Thr	I	III	VII	Gallus gallus	
Q5F3U4	Ser/Thr	I	III	VII	Gallus gallus	
Q5ZJB8	Ser/Thr	I	III	VII	Gallus gallus	
Q5ZJQ4	Ser/Thr	I	III	VII	Gallus gallus	
Q5ZKL2	Ser/Thr	I	III	VII	Gallus gallus	
Q98SN6	Ser/Thr	I	III	VII	Gallus gallus	
Q9PUJ3	Ser/Thr	I	III	VII	Gallus gallus	
Q1H8W8	Ser/Thr	I	III	VII	Giardia intestinalis	
Q9GU84	Ser/Thr	I	III	VII	Giardia lamblia	
Q9U020	Ser/Thr	I	III	VII	Giardia lamblia	
Q75T36	Ser/Thr	I	III	VII	Glomerella lagenarium	
Q39908	Ser/Thr	I	III	VII	Gonyaulax polyedra	
Q25115	Ser/Thr	I	III	VII	Hemicentrotus pulcherrimus	
Q6A1A2	Ser/Thr	I	III	VII	Homo sapiens	2.7.11.1
Q9BRS0	Ser/Thr	I	III	VII	Homo sapiens	2.7.11.1
A0JLR0	Ser/Thr	I	III	VII	Homo sapiens	
Q15136	Ser/Thr	I	III	VII	Homo sapiens	
Q53EW6	Ser/Thr	I	III	VII	Homo sapiens	
Q53HJ9	Ser/Thr	I	III	VII	Homo sapiens	
Q59EB4	Ser/Thr	I	III	VII	Homo sapiens	
Q59FU6	Ser/Thr	I	III	VII	Homo sapiens	
Q5H8Y5	Ser/Thr	I	III	VII	Homo sapiens	
Q5H9Q5	Ser/Thr	I	III	VII	Homo sapiens	
Q6FHV7	Ser/Thr	I	III	VII	Homo sapiens	
Q86XZ8	Ser/Thr	I	III	VII	Homo sapiens	
Q96AD6	Ser/Thr	I	III	VII	Homo sapiens	
Q9UPJ8	Ser/Thr	I	III	VII	Homo sapiens	
O17474	Ser/Thr	I	III	VII	Hydra oligactis	2.7.11.12
A5AA56	Ser/Thr	I	III	VII	Hydra vulgaris	
Q4AC21	Ser/Thr	I	III	VII	Hydroides elegans	
O60042	Ser/Thr	I	III	VII	Kluyveromyces lactis	
Q6CIR3	Ser/Thr	I	III	VII	Kluyveromyces lactis	
Q6CS82	Ser/Thr	I	III	VII	Kluyveromyces lactis	
Q6CSR2	Ser/Thr	I	III	VII	Kluyveromyces lactis	
Q6CTI4	Ser/Thr	I	III	VII	Kluyveromyces lactis	
Q6CVE2	Ser/Thr	I	III	VII	Kluyveromyces lactis	
Q6CW38	Ser/Thr	I	III	VII	Kluyveromyces lactis	

Table S2.3. (continuation)

UniProt	Label [1]	Two	Three	Seven	Species	EC Number
Q6CWJ5	Ser/Thr	I	III	VII	Kluyveromyces lactis	
Q9GN16	Ser/Thr	I	III	VII	Leishmania major	2.7.1.37
Q9GNR4	Ser/Thr	I	III	VII	Leishmania major	2.7.1.37
Q9GRU4	Ser/Thr	I	III	VII	Leishmania major	2.7.1.37
Q27687	Ser/Thr	I	III	VII	Leishmania major	2.7.1.37; 2.7.11.1
Q4Q7M5	Ser/Thr	I	III	VII	Leishmania major	2.7.11.1
Q4Q9K2	Ser/Thr	I	III	VII	Leishmania major	2.7.11.1
Q0Z847	Ser/Thr	I	III	VII	Lobesia botrana	2.7.11.12
A5DT22	Ser/Thr	I	III	VII	Lodderomyces elongisporus	
A5DT69	Ser/Thr	I	III	VII	Lodderomyces elongisporus	
A5DTG0	Ser/Thr	I	III	VII	Lodderomyces elongisporus	
A5DV85	Ser/Thr	I	III	VII	Lodderomyces elongisporus	
A5DYL9	Ser/Thr	I	III	VII	Lodderomyces elongisporus	
A5E1X6	Ser/Thr	I	III	VII	Lodderomyces elongisporus	
Q4JIV3	Ser/Thr	I	III	VII	Lymnaea stagnalis	
Q4R5I9	Ser/Thr	I	III	VII	Macaca fascicularis	2.7.11.1
Q4R633	Ser/Thr	I	III	VII	Macaca fascicularis	2.7.11.1
Q9BGT4	Ser/Thr	I	III	VII	Macaca fascicularis	
Q01143	Ser/Thr	I	III	VII	Magnaporthe grisea	
Q86ZN6	Ser/Thr	I	III	VII	Magnaporthe grisea	
Q9Y777	Ser/Thr	I	III	VII	Metarhizium anisopliae	
A5DAZ1	Ser/Thr	I	III	VII	Meyerozyma guilliermondii	
A5DES7	Ser/Thr	I	III	VII	Meyerozyma guilliermondii	
A5DGR1	Ser/Thr	I	III	VII	Meyerozyma guilliermondii	
A5DHJ5	Ser/Thr	I	III	VII	Meyerozyma guilliermondii	
A5DM86	Ser/Thr	I	III	VII	Meyerozyma guilliermondii	
A5DME0	Ser/Thr	I	III	VII	Meyerozyma guilliermondii	
A5DR02	Ser/Thr	I	III	VII	Meyerozyma guilliermondii	
A5DR74	Ser/Thr	I	III	VII	Meyerozyma guilliermondii	
Q3UXD8	Ser/Thr	I	III	VII	Mus musculus	2.7.11.1
Q8BND1	Ser/Thr	I	III	VII	Mus musculus	2.7.11.12
Q8C4R2	Ser/Thr	I	III	VII	Mus musculus	2.7.11.12
Q8CAH8	Ser/Thr	I	III	VII	Mus musculus	2.7.11.12
O70296	Ser/Thr	I	III	VII	Mus musculus	
Q05CL1	Ser/Thr	I	III	VII	Mus musculus	
Q3TR46	Ser/Thr	I	III	VII	Mus musculus	
Q3TRL2	Ser/Thr	I	III	VII	Mus musculus	
Q3TST4	Ser/Thr	I	III	VII	Mus musculus	
Q3TTW2	Ser/Thr	I	III	VII	Mus musculus	
Q3U1V3	Ser/Thr	I	III	VII	Mus musculus	
Q3U907	Ser/Thr	I	III	VII	Mus musculus	
Q3U9B2	Ser/Thr	I	III	VII	Mus musculus	
Q3U9H2	Ser/Thr	I	III	VII	Mus musculus	
Q3UDT1	Ser/Thr	I	III	VII	Mus musculus	
Q3UE22	Ser/Thr	I	III	VII	Mus musculus	
Q3UEW8	Ser/Thr	I	III	VII	Mus musculus	
Q3UGN6	Ser/Thr	I	III	VII	Mus musculus	
Q3UHZ0	Ser/Thr	I	III	VII	Mus musculus	
Q3UK88	Ser/Thr	I	III	VII	Mus musculus	
Q3UMT5	Ser/Thr	I	III	VII	Mus musculus	
Q3URY8	Ser/Thr	I	III	VII	Mus musculus	
Q3UW73	Ser/Thr	I	III	VII	Mus musculus	
Q3V226	Ser/Thr	I	III	VII	Mus musculus	
Q4V9Z9	Ser/Thr	I	III	VII	Mus musculus	
Q6DI71	Ser/Thr	I	III	VII	Mus musculus	
Q6NXX3	Ser/Thr	I	III	VII	Mus musculus	

Table S2.3. (continuation)

UniProt	Label [1]	Two	Three	Seven	Species	EC Number
Q792R0	Ser/Thr	I	III	VII	Mus musculus	
Q7TS64	Ser/Thr	I	III	VII	Mus musculus	
Q810Z4	Ser/Thr	I	III	VII	Mus musculus	
Q8BVT9	Ser/Thr	I	III	VII	Mus musculus	
Q8K3L3	Ser/Thr	I	III	VII	Mus musculus	
Q8VEK1	Ser/Thr	I	III	VII	Mus musculus	
Q9CST0	Ser/Thr	I	III	VII	Mus musculus	
Q9EP84	Ser/Thr	I	III	VII	Mus musculus	
Q1KTF1	Ser/Thr	I	III	VII	Mycosphaerella graminicola	
A7SCZ7	Ser/Thr	I	III	VII	Nematostella vectensis	2.7.11.12
A7RIL6	Ser/Thr	I	III	VII	Nematostella vectensis	
A7RKQ3	Ser/Thr	I	III	VII	Nematostella vectensis	
A7RLB8	Ser/Thr	I	III	VII	Nematostella vectensis	
A7S134	Ser/Thr	I	III	VII	Nematostella vectensis	
A7S521	Ser/Thr	I	III	VII	Nematostella vectensis	
A7SCT2	Ser/Thr	I	III	VII	Nematostella vectensis	
A7SCY8	Ser/Thr	I	III	VII	Nematostella vectensis	
A7SEW2	Ser/Thr	I	III	VII	Nematostella vectensis	
A7SF23	Ser/Thr	I	III	VII	Nematostella vectensis	
A7SM62	Ser/Thr	I	III	VII	Nematostella vectensis	
A7SW88	Ser/Thr	I	III	VII	Nematostella vectensis	
A1D2X8	Ser/Thr	I	III	VII	Neosartorya fischeri	
A1D3Y6	Ser/Thr	I	III	VII	Neosartorya fischeri	
A1D760	Ser/Thr	I	III	VII	Neosartorya fischeri	
A1DEX4	Ser/Thr	I	III	VII	Neosartorya fischeri	
A1DGY6	Ser/Thr	I	III	VII	Neosartorya fischeri	
A1DHC5	Ser/Thr	I	III	VII	Neosartorya fischeri	
A1DMX3	Ser/Thr	I	III	VII	Neosartorya fischeri	
Q1K4T2	Ser/Thr	I	III	VII	Neurospora crassa	
Q7RVX6	Ser/Thr	I	III	VII	Neurospora crassa	
Q7RYM0	Ser/Thr	I	III	VII	Neurospora crassa	
Q7S6G1	Ser/Thr	I	III	VII	Neurospora crassa	
Q7SHX1	Ser/Thr	I	III	VII	Neurospora crassa	
Q873K1	Ser/Thr	I	III	VII	Neurospora crassa	
Q876Z5	Ser/Thr	I	III	VII	Neurospora crassa	
Q9P466	Ser/Thr	I	III	VII	Neurospora crassa	
Q5QFC4	Ser/Thr	I	III	VII	Oikopleura dioica	
Q800K1	Ser/Thr	I	III	VII	Oncorhynchus mykiss	
A7UJ13	Ser/Thr	I	III	VII	Origanum onites	
Q95J97	Ser/Thr	I	III	VII	Oryctolagus cuniculus	
A2WLL4	Ser/Thr	I	III	VII	Oryza sativa	
A2XGD6	Ser/Thr	I	III	VII	Oryza sativa	
A2YPZ7	Ser/Thr	I	III	VII	Oryza sativa	
Q0D3L1	Ser/Thr	I	III	VII	Oryza sativa	
Q10EP9	Ser/Thr	I	III	VII	Oryza sativa	
Q10LV1	Ser/Thr	I	III	VII	Oryza sativa	
Q4R1K7	Ser/Thr	I	III	VII	Oryza sativa	
Q5NAR7	Ser/Thr	I	III	VII	Oryza sativa	
Q5SNH4	Ser/Thr	I	III	VII	Oryza sativa	
Q7XHW9	Ser/Thr	I	III	VII	Oryza sativa	
Q7Y0C4	Ser/Thr	I	III	VII	Oryza sativa	
Q8RUE8	Ser/Thr	I	III	VII	Oryza sativa	
Q9XF68	Ser/Thr	I	III	VII	Oryza sativa	
Q75PY3	Ser/Thr	I	III	VII	Oryzias latipes	2.7.11.12
Q75PY4	Ser/Thr	I	III	VII	Oryzias latipes	2.7.11.12
O73658	Ser/Thr	I	III	VII	Oryzias latipes	

Table S2.3. (continuation)

UniProt	Label [1]	Two	Three	Seven	Species	EC Number
O73659	Ser/Thr	I	III	VII	Oryzias latipes	
Q5NUF5	Ser/Thr	I	III	VII	Oryzias latipes	
A4RT89	Ser/Thr	I	III	VII	Ostreococcus lucimarinus	
A4RW01	Ser/Thr	I	III	VII	Ostreococcus lucimarinus	
A4RX83	Ser/Thr	I	III	VII	Ostreococcus lucimarinus	
A4S0B2	Ser/Thr	I	III	VII	Ostreococcus lucimarinus	
A4S353	Ser/Thr	I	III	VII	Ostreococcus lucimarinus	
A4S6E2	Ser/Thr	I	III	VII	Ostreococcus lucimarinus	
A4S904	Ser/Thr	I	III	VII	Ostreococcus lucimarinus	
Q00Z65	Ser/Thr	I	III	VII	Ostreococcus tauri	
Q01B06	Ser/Thr	I	III	VII	Ostreococcus tauri	
Q5XXD0	Ser/Thr	I	III	VII	Pan troglodytes	
O00843	Ser/Thr	I	III	VII	Paramecium primaurelia	
Q869J9	Ser/Thr	I	III	VII	Paramecium tetraurelia	2.7.1.37
Q869K0	Ser/Thr	I	III	VII	Paramecium tetraurelia	2.7.1.37
A0BB08	Ser/Thr	I	III	VII	Paramecium tetraurelia	
A0BCA7	Ser/Thr	I	III	VII	Paramecium tetraurelia	
A0BCG0	Ser/Thr	I	III	VII	Paramecium tetraurelia	
A0BCQ1	Ser/Thr	I	III	VII	Paramecium tetraurelia	
A0BCT1	Ser/Thr	I	III	VII	Paramecium tetraurelia	
A0BD03	Ser/Thr	I	III	VII	Paramecium tetraurelia	
A0BDJ1	Ser/Thr	I	III	VII	Paramecium tetraurelia	
A0BDJ5	Ser/Thr	I	III	VII	Paramecium tetraurelia	
A0BDM6	Ser/Thr	I	III	VII	Paramecium tetraurelia	
A0BDU3	Ser/Thr	I	III	VII	Paramecium tetraurelia	
A0BDZ8	Ser/Thr	I	III	VII	Paramecium tetraurelia	
A0BE58	Ser/Thr	I	III	VII	Paramecium tetraurelia	
A0BEY5	Ser/Thr	I	III	VII	Paramecium tetraurelia	
A0BFR1	Ser/Thr	I	III	VII	Paramecium tetraurelia	
A0BGU9	Ser/Thr	I	III	VII	Paramecium tetraurelia	
A0BI00	Ser/Thr	I	III	VII	Paramecium tetraurelia	
A0BI56	Ser/Thr	I	III	VII	Paramecium tetraurelia	
A0BJB9	Ser/Thr	I	III	VII	Paramecium tetraurelia	
A0BJM1	Ser/Thr	I	III	VII	Paramecium tetraurelia	
A0BJU7	Ser/Thr	I	III	VII	Paramecium tetraurelia	
A0BJX5	Ser/Thr	I	III	VII	Paramecium tetraurelia	
A0BKL5	Ser/Thr	I	III	VII	Paramecium tetraurelia	
A0BKM5	Ser/Thr	I	III	VII	Paramecium tetraurelia	
A0BKN3	Ser/Thr	I	III	VII	Paramecium tetraurelia	
A0BKR3	Ser/Thr	I	III	VII	Paramecium tetraurelia	
A0BL00	Ser/Thr	I	III	VII	Paramecium tetraurelia	
A0BMX1	Ser/Thr	I	III	VII	Paramecium tetraurelia	
A0BN99	Ser/Thr	I	III	VII	Paramecium tetraurelia	
A0BNB1	Ser/Thr	I	III	VII	Paramecium tetraurelia	
A0BNB6	Ser/Thr	I	III	VII	Paramecium tetraurelia	
A0BNJ0	Ser/Thr	I	III	VII	Paramecium tetraurelia	
A0BNY9	Ser/Thr	I	III	VII	Paramecium tetraurelia	
A0BP84	Ser/Thr	I	III	VII	Paramecium tetraurelia	
A0BPA1	Ser/Thr	I	III	VII	Paramecium tetraurelia	
A0BPA4	Ser/Thr	I	III	VII	Paramecium tetraurelia	
A0BPB4	Ser/Thr	I	III	VII	Paramecium tetraurelia	
A0BQE1	Ser/Thr	I	III	VII	Paramecium tetraurelia	
A0BQV0	Ser/Thr	I	III	VII	Paramecium tetraurelia	
A0BR46	Ser/Thr	I	III	VII	Paramecium tetraurelia	
A0BRU5	Ser/Thr	I	III	VII	Paramecium tetraurelia	
A0BSE9	Ser/Thr	I	III	VII	Paramecium tetraurelia	

Table S2.3. (continuation)

UniProt	Label [1]	Two	Three	Seven	Species	EC Number
A0BSF2	Ser/Thr	I	III	VII	Paramecium tetraurelia	
A0BT25	Ser/Thr	I	III	VII	Paramecium tetraurelia	
A0BTC1	Ser/Thr	I	III	VII	Paramecium tetraurelia	
A0BUZ8	Ser/Thr	I	III	VII	Paramecium tetraurelia	
A0BVM5	Ser/Thr	I	III	VII	Paramecium tetraurelia	
A0BVW4	Ser/Thr	I	III	VII	Paramecium tetraurelia	
A0BW98	Ser/Thr	I	III	VII	Paramecium tetraurelia	
A0BWD7	Ser/Thr	I	III	VII	Paramecium tetraurelia	
A0BWJ4	Ser/Thr	I	III	VII	Paramecium tetraurelia	
A0BWM2	Ser/Thr	I	III	VII	Paramecium tetraurelia	
A0BX01	Ser/Thr	I	III	VII	Paramecium tetraurelia	
A0BY36	Ser/Thr	I	III	VII	Paramecium tetraurelia	
A0BYR7	Ser/Thr	I	III	VII	Paramecium tetraurelia	
A0BYV3	Ser/Thr	I	III	VII	Paramecium tetraurelia	
A0BZ62	Ser/Thr	I	III	VII	Paramecium tetraurelia	
A0BZE4	Ser/Thr	I	III	VII	Paramecium tetraurelia	
A0BZL2	Ser/Thr	I	III	VII	Paramecium tetraurelia	
A0BZV2	Ser/Thr	I	III	VII	Paramecium tetraurelia	
A0C0W1	Ser/Thr	I	III	VII	Paramecium tetraurelia	
A0C1S2	Ser/Thr	I	III	VII	Paramecium tetraurelia	
A0C235	Ser/Thr	I	III	VII	Paramecium tetraurelia	
A0C2B1	Ser/Thr	I	III	VII	Paramecium tetraurelia	
A0C3V8	Ser/Thr	I	III	VII	Paramecium tetraurelia	
A0C404	Ser/Thr	I	III	VII	Paramecium tetraurelia	
A0C4H5	Ser/Thr	I	III	VII	Paramecium tetraurelia	
A0C512	Ser/Thr	I	III	VII	Paramecium tetraurelia	
A0C580	Ser/Thr	I	III	VII	Paramecium tetraurelia	
A0C5F6	Ser/Thr	I	III	VII	Paramecium tetraurelia	
A0C5R9	Ser/Thr	I	III	VII	Paramecium tetraurelia	
A0C6P2	Ser/Thr	I	III	VII	Paramecium tetraurelia	
A0C6X0	Ser/Thr	I	III	VII	Paramecium tetraurelia	
A0C7N8	Ser/Thr	I	III	VII	Paramecium tetraurelia	
A0C8I8	Ser/Thr	I	III	VII	Paramecium tetraurelia	
A0C8J6	Ser/Thr	I	III	VII	Paramecium tetraurelia	
A0C9B7	Ser/Thr	I	III	VII	Paramecium tetraurelia	
A0C9E5	Ser/Thr	I	III	VII	Paramecium tetraurelia	
A0C9N7	Ser/Thr	I	III	VII	Paramecium tetraurelia	
A0C9W0	Ser/Thr	I	III	VII	Paramecium tetraurelia	
A0CAR8	Ser/Thr	I	III	VII	Paramecium tetraurelia	
A0CB56	Ser/Thr	I	III	VII	Paramecium tetraurelia	
A0CBK4	Ser/Thr	I	III	VII	Paramecium tetraurelia	
A0CC53	Ser/Thr	I	III	VII	Paramecium tetraurelia	
A0CC65	Ser/Thr	I	III	VII	Paramecium tetraurelia	
A0CDF0	Ser/Thr	I	III	VII	Paramecium tetraurelia	
A0CDJ0	Ser/Thr	I	III	VII	Paramecium tetraurelia	
A0CFD9	Ser/Thr	I	III	VII	Paramecium tetraurelia	
A0CG15	Ser/Thr	I	III	VII	Paramecium tetraurelia	
A0CGN9	Ser/Thr	I	III	VII	Paramecium tetraurelia	
A0CGV1	Ser/Thr	I	III	VII	Paramecium tetraurelia	
A0CI44	Ser/Thr	I	III	VII	Paramecium tetraurelia	
A0CIA0	Ser/Thr	I	III	VII	Paramecium tetraurelia	
A0CIC7	Ser/Thr	I	III	VII	Paramecium tetraurelia	
A0CID7	Ser/Thr	I	III	VII	Paramecium tetraurelia	
A0CIP0	Ser/Thr	I	III	VII	Paramecium tetraurelia	
A0CJE1	Ser/Thr	I	III	VII	Paramecium tetraurelia	
A0CK96	Ser/Thr	I	III	VII	Paramecium tetraurelia	

Table S2.3. (continuation)

UniProt	Label [1]	Two	Three	Seven	Species	EC Number
A0CKQ1	Ser/Thr	I	III	VII	Paramecium tetraurelia	
A0CLB9	Ser/Thr	I	III	VII	Paramecium tetraurelia	
A0CMB4	Ser/Thr	I	III	VII	Paramecium tetraurelia	
A0CMG7	Ser/Thr	I	III	VII	Paramecium tetraurelia	
A0CMK1	Ser/Thr	I	III	VII	Paramecium tetraurelia	
A0CMK7	Ser/Thr	I	III	VII	Paramecium tetraurelia	
A0CNC9	Ser/Thr	I	III	VII	Paramecium tetraurelia	
A0CNT4	Ser/Thr	I	III	VII	Paramecium tetraurelia	
A0CP93	Ser/Thr	I	III	VII	Paramecium tetraurelia	
A0CQJ9	Ser/Thr	I	III	VII	Paramecium tetraurelia	
A0CQT7	Ser/Thr	I	III	VII	Paramecium tetraurelia	
A0CQZ9	Ser/Thr	I	III	VII	Paramecium tetraurelia	
A0CRL6	Ser/Thr	I	III	VII	Paramecium tetraurelia	
A0CS44	Ser/Thr	I	III	VII	Paramecium tetraurelia	
A0CSN3	Ser/Thr	I	III	VII	Paramecium tetraurelia	
A0CST1	Ser/Thr	I	III	VII	Paramecium tetraurelia	
A0CT39	Ser/Thr	I	III	VII	Paramecium tetraurelia	
A0CUG4	Ser/Thr	I	III	VII	Paramecium tetraurelia	
A0CV45	Ser/Thr	I	III	VII	Paramecium tetraurelia	
A0CVL9	Ser/Thr	I	III	VII	Paramecium tetraurelia	
A0CW61	Ser/Thr	I	III	VII	Paramecium tetraurelia	
A0CW99	Ser/Thr	I	III	VII	Paramecium tetraurelia	
A0CWG2	Ser/Thr	I	III	VII	Paramecium tetraurelia	
A0CWH2	Ser/Thr	I	III	VII	Paramecium tetraurelia	
A0CWY4	Ser/Thr	I	III	VII	Paramecium tetraurelia	
A0CX71	Ser/Thr	I	III	VII	Paramecium tetraurelia	
A0CX89	Ser/Thr	I	III	VII	Paramecium tetraurelia	
A0CYA4	Ser/Thr	I	III	VII	Paramecium tetraurelia	
A0CYI7	Ser/Thr	I	III	VII	Paramecium tetraurelia	
A0CZM6	Ser/Thr	I	III	VII	Paramecium tetraurelia	
A0D032	Ser/Thr	I	III	VII	Paramecium tetraurelia	
A0D0C8	Ser/Thr	I	III	VII	Paramecium tetraurelia	
A0D1D1	Ser/Thr	I	III	VII	Paramecium tetraurelia	
A0D329	Ser/Thr	I	III	VII	Paramecium tetraurelia	
A0D5C7	Ser/Thr	I	III	VII	Paramecium tetraurelia	
A0D680	Ser/Thr	I	III	VII	Paramecium tetraurelia	
A0D712	Ser/Thr	I	III	VII	Paramecium tetraurelia	
A0D863	Ser/Thr	I	III	VII	Paramecium tetraurelia	
A0D8K8	Ser/Thr	I	III	VII	Paramecium tetraurelia	
A0D915	Ser/Thr	I	III	VII	Paramecium tetraurelia	
A0D9C4	Ser/Thr	I	III	VII	Paramecium tetraurelia	
A0DAI4	Ser/Thr	I	III	VII	Paramecium tetraurelia	
A0DAI8	Ser/Thr	I	III	VII	Paramecium tetraurelia	
A0DBE5	Ser/Thr	I	III	VII	Paramecium tetraurelia	
A0DBI7	Ser/Thr	I	III	VII	Paramecium tetraurelia	
A0DBI9	Ser/Thr	I	III	VII	Paramecium tetraurelia	
A0DBL2	Ser/Thr	I	III	VII	Paramecium tetraurelia	
A0DCJ0	Ser/Thr	I	III	VII	Paramecium tetraurelia	
A0DDH0	Ser/Thr	I	III	VII	Paramecium tetraurelia	
A0DDP9	Ser/Thr	I	III	VII	Paramecium tetraurelia	
A0DDW7	Ser/Thr	I	III	VII	Paramecium tetraurelia	
A0DE77	Ser/Thr	I	III	VII	Paramecium tetraurelia	
A0DEV3	Ser/Thr	I	III	VII	Paramecium tetraurelia	
A0DFG8	Ser/Thr	I	III	VII	Paramecium tetraurelia	
A0DFK5	Ser/Thr	I	III	VII	Paramecium tetraurelia	
A0DG95	Ser/Thr	I	III	VII	Paramecium tetraurelia	

Table S2.3. (continuation)

UniProt	Label [1]	Two	Three	Seven	Species	EC Number
A0DGH3	Ser/Thr	I	III	VII	Paramecium tetraurelia	
A0DGQ2	Ser/Thr	I	III	VII	Paramecium tetraurelia	
A0DGS5	Ser/Thr	I	III	VII	Paramecium tetraurelia	
A0DHV9	Ser/Thr	I	III	VII	Paramecium tetraurelia	
A0DI12	Ser/Thr	I	III	VII	Paramecium tetraurelia	
A0DI25	Ser/Thr	I	III	VII	Paramecium tetraurelia	
A0DIF4	Ser/Thr	I	III	VII	Paramecium tetraurelia	
A0DII8	Ser/Thr	I	III	VII	Paramecium tetraurelia	
A0DJL5	Ser/Thr	I	III	VII	Paramecium tetraurelia	
A0DJV5	Ser/Thr	I	III	VII	Paramecium tetraurelia	
A0DK75	Ser/Thr	I	III	VII	Paramecium tetraurelia	
A0DLX8	Ser/Thr	I	III	VII	Paramecium tetraurelia	
A0DN73	Ser/Thr	I	III	VII	Paramecium tetraurelia	
A0DP02	Ser/Thr	I	III	VII	Paramecium tetraurelia	
A0DP63	Ser/Thr	I	III	VII	Paramecium tetraurelia	
A0DPF6	Ser/Thr	I	III	VII	Paramecium tetraurelia	
A0DPR8	Ser/Thr	I	III	VII	Paramecium tetraurelia	
A0DPW4	Ser/Thr	I	III	VII	Paramecium tetraurelia	
A0DQ36	Ser/Thr	I	III	VII	Paramecium tetraurelia	
A0DQT2	Ser/Thr	I	III	VII	Paramecium tetraurelia	
A0DS68	Ser/Thr	I	III	VII	Paramecium tetraurelia	
A0DT28	Ser/Thr	I	III	VII	Paramecium tetraurelia	
A0DTA2	Ser/Thr	I	III	VII	Paramecium tetraurelia	
A0DTB0	Ser/Thr	I	III	VII	Paramecium tetraurelia	
A0DTK8	Ser/Thr	I	III	VII	Paramecium tetraurelia	
A0DTL3	Ser/Thr	I	III	VII	Paramecium tetraurelia	
A0DTN5	Ser/Thr	I	III	VII	Paramecium tetraurelia	
A0DUD9	Ser/Thr	I	III	VII	Paramecium tetraurelia	
A0DUR1	Ser/Thr	I	III	VII	Paramecium tetraurelia	
A0DUX2	Ser/Thr	I	III	VII	Paramecium tetraurelia	
A0DVC5	Ser/Thr	I	III	VII	Paramecium tetraurelia	
A0DVL2	Ser/Thr	I	III	VII	Paramecium tetraurelia	
A0DVZ3	Ser/Thr	I	III	VII	Paramecium tetraurelia	
A0DWP4	Ser/Thr	I	III	VII	Paramecium tetraurelia	
A0DX28	Ser/Thr	I	III	VII	Paramecium tetraurelia	
A0DXD7	Ser/Thr	I	III	VII	Paramecium tetraurelia	
A0DXJ1	Ser/Thr	I	III	VII	Paramecium tetraurelia	
A0DXM5	Ser/Thr	I	III	VII	Paramecium tetraurelia	
A0DXV5	Ser/Thr	I	III	VII	Paramecium tetraurelia	
A0DXX0	Ser/Thr	I	III	VII	Paramecium tetraurelia	
A0DXZ0	Ser/Thr	I	III	VII	Paramecium tetraurelia	
A0DYJ0	Ser/Thr	I	III	VII	Paramecium tetraurelia	
A0DYS1	Ser/Thr	I	III	VII	Paramecium tetraurelia	
A0DYT2	Ser/Thr	I	III	VII	Paramecium tetraurelia	
A0DZB2	Ser/Thr	I	III	VII	Paramecium tetraurelia	
A0DZJ1	Ser/Thr	I	III	VII	Paramecium tetraurelia	
A0DZK2	Ser/Thr	I	III	VII	Paramecium tetraurelia	
A0DZL0	Ser/Thr	I	III	VII	Paramecium tetraurelia	
A0DZS8	Ser/Thr	I	III	VII	Paramecium tetraurelia	
A0DZW3	Ser/Thr	I	III	VII	Paramecium tetraurelia	
A0E058	Ser/Thr	I	III	VII	Paramecium tetraurelia	
A0E0C0	Ser/Thr	I	III	VII	Paramecium tetraurelia	
A0E0G1	Ser/Thr	I	III	VII	Paramecium tetraurelia	
A0E0Q7	Ser/Thr	I	III	VII	Paramecium tetraurelia	
A0E1G0	Ser/Thr	I	III	VII	Paramecium tetraurelia	
A0E1G8	Ser/Thr	I	III	VII	Paramecium tetraurelia	

Table S2.3. (continuation)

UniProt	Label [1]	Two	Three	Seven	Species	EC Number
A0E1S7	Ser/Thr	I	III	VII	Paramecium tetraurelia	
A0E271	Ser/Thr	I	III	VII	Paramecium tetraurelia	
A0E2H7	Ser/Thr	I	III	VII	Paramecium tetraurelia	
A0E2Q8	Ser/Thr	I	III	VII	Paramecium tetraurelia	
A0E2T3	Ser/Thr	I	III	VII	Paramecium tetraurelia	
A0E2W5	Ser/Thr	I	III	VII	Paramecium tetraurelia	
A0E3G7	Ser/Thr	I	III	VII	Paramecium tetraurelia	
A0E4C0	Ser/Thr	I	III	VII	Paramecium tetraurelia	
A0E4D3	Ser/Thr	I	III	VII	Paramecium tetraurelia	
A0E4E1	Ser/Thr	I	III	VII	Paramecium tetraurelia	
A0E4K1	Ser/Thr	I	III	VII	Paramecium tetraurelia	
A0E4Z6	Ser/Thr	I	III	VII	Paramecium tetraurelia	
A0E525	Ser/Thr	I	III	VII	Paramecium tetraurelia	
A0E5D2	Ser/Thr	I	III	VII	Paramecium tetraurelia	
A0E5S2	Ser/Thr	I	III	VII	Paramecium tetraurelia	
A0E6L8	Ser/Thr	I	III	VII	Paramecium tetraurelia	
A0E7B2	Ser/Thr	I	III	VII	Paramecium tetraurelia	
A0E888	Ser/Thr	I	III	VII	Paramecium tetraurelia	
A0E8C9	Ser/Thr	I	III	VII	Paramecium tetraurelia	
A0E8L1	Ser/Thr	I	III	VII	Paramecium tetraurelia	
A0E901	Ser/Thr	I	III	VII	Paramecium tetraurelia	
A0E941	Ser/Thr	I	III	VII	Paramecium tetraurelia	
A0E9E6	Ser/Thr	I	III	VII	Paramecium tetraurelia	
A0E9N3	Ser/Thr	I	III	VII	Paramecium tetraurelia	
A0E9U0	Ser/Thr	I	III	VII	Paramecium tetraurelia	
A0EAF2	Ser/Thr	I	III	VII	Paramecium tetraurelia	
A0EAI5	Ser/Thr	I	III	VII	Paramecium tetraurelia	
A0EAJ3	Ser/Thr	I	III	VII	Paramecium tetraurelia	
A0EB53	Ser/Thr	I	III	VII	Paramecium tetraurelia	
A0ECJ7	Ser/Thr	I	III	VII	Paramecium tetraurelia	
A0ED59	Ser/Thr	I	III	VII	Paramecium tetraurelia	
A0EDB6	Ser/Thr	I	III	VII	Paramecium tetraurelia	
A0EDP3	Ser/Thr	I	III	VII	Paramecium tetraurelia	
A0EDW8	Ser/Thr	I	III	VII	Paramecium tetraurelia	
A0EEZ4	Ser/Thr	I	III	VII	Paramecium tetraurelia	
A0EF60	Ser/Thr	I	III	VII	Paramecium tetraurelia	
A0EFE8	Ser/Thr	I	III	VII	Paramecium tetraurelia	
A0EFF7	Ser/Thr	I	III	VII	Paramecium tetraurelia	
A0EFH0	Ser/Thr	I	III	VII	Paramecium tetraurelia	
A0EGM3	Ser/Thr	I	III	VII	Paramecium tetraurelia	
A0EHG7	Ser/Thr	I	III	VII	Paramecium tetraurelia	
A0EHH9	Ser/Thr	I	III	VII	Paramecium tetraurelia	
A0EHI5	Ser/Thr	I	III	VII	Paramecium tetraurelia	
A0EHV4	Ser/Thr	I	III	VII	Paramecium tetraurelia	
A0EI85	Ser/Thr	I	III	VII	Paramecium tetraurelia	
A0EIN6	Ser/Thr	I	III	VII	Paramecium tetraurelia	
Q3M0W6	Ser/Thr	I	III	VII	Paramecium tetraurelia	
Q3SDX8	Ser/Thr	I	III	VII	Paramecium tetraurelia	
Q3SDX9	Ser/Thr	I	III	VII	Paramecium tetraurelia	
Q3SDY0	Ser/Thr	I	III	VII	Paramecium tetraurelia	
Q3SDY1	Ser/Thr	I	III	VII	Paramecium tetraurelia	
Q3SDY2	Ser/Thr	I	III	VII	Paramecium tetraurelia	
Q3SDY3	Ser/Thr	I	III	VII	Paramecium tetraurelia	
Q3SDY4	Ser/Thr	I	III	VII	Paramecium tetraurelia	
Q3SDY5	Ser/Thr	I	III	VII	Paramecium tetraurelia	
Q3SDY6	Ser/Thr	I	III	VII	Paramecium tetraurelia	

Table S2.3. (continuation)

UniProt	Label [1]	Two	Three	Seven	Species	EC Number
Q3SDY7	Ser/Thr	I	III	VII	Paramecium tetraurelia	
Q3SEC4	Ser/Thr	I	III	VII	Paramecium tetraurelia	
Q3SEC5	Ser/Thr	I	III	VII	Paramecium tetraurelia	
Q3SEC6	Ser/Thr	I	III	VII	Paramecium tetraurelia	
Q3SEC7	Ser/Thr	I	III	VII	Paramecium tetraurelia	
Q3SEC8	Ser/Thr	I	III	VII	Paramecium tetraurelia	
Q3SEC9	Ser/Thr	I	III	VII	Paramecium tetraurelia	
Q3SEL4	Ser/Thr	I	III	VII	Paramecium tetraurelia	
Q3SEL5	Ser/Thr	I	III	VII	Paramecium tetraurelia	
Q3SEL7	Ser/Thr	I	III	VII	Paramecium tetraurelia	
Q3SEL8	Ser/Thr	I	III	VII	Paramecium tetraurelia	
Q3SEL9	Ser/Thr	I	III	VII	Paramecium tetraurelia	
Q3SEM0	Ser/Thr	I	III	VII	Paramecium tetraurelia	
Q3SEM1	Ser/Thr	I	III	VII	Paramecium tetraurelia	
Q3SEM2	Ser/Thr	I	III	VII	Paramecium tetraurelia	
Q3SEM3	Ser/Thr	I	III	VII	Paramecium tetraurelia	
Q3SEM5	Ser/Thr	I	III	VII	Paramecium tetraurelia	
Q3SEM6	Ser/Thr	I	III	VII	Paramecium tetraurelia	
Q3SEM9	Ser/Thr	I	III	VII	Paramecium tetraurelia	
Q3SEN0	Ser/Thr	I	III	VII	Paramecium tetraurelia	
Q3SEN1	Ser/Thr	I	III	VII	Paramecium tetraurelia	
Q3SEN2	Ser/Thr	I	III	VII	Paramecium tetraurelia	
Q3SEN3	Ser/Thr	I	III	VII	Paramecium tetraurelia	
Q3SEN4	Ser/Thr	I	III	VII	Paramecium tetraurelia	
Q3SEN5	Ser/Thr	I	III	VII	Paramecium tetraurelia	
Q3SEN6	Ser/Thr	I	III	VII	Paramecium tetraurelia	
Q3SEN7	Ser/Thr	I	III	VII	Paramecium tetraurelia	
Q3SEN8	Ser/Thr	I	III	VII	Paramecium tetraurelia	
Q3SEN9	Ser/Thr	I	III	VII	Paramecium tetraurelia	
Q3SEP0	Ser/Thr	I	III	VII	Paramecium tetraurelia	
Q3SEP1	Ser/Thr	I	III	VII	Paramecium tetraurelia	
Q3SEP2	Ser/Thr	I	III	VII	Paramecium tetraurelia	
Q3SEP3	Ser/Thr	I	III	VII	Paramecium tetraurelia	
Q3SEP4	Ser/Thr	I	III	VII	Paramecium tetraurelia	
Q6BFL9	Ser/Thr	I	III	VII	Paramecium tetraurelia	
Q6BFN6	Ser/Thr	I	III	VII	Paramecium tetraurelia	
Q6BFR4	Ser/Thr	I	III	VII	Paramecium tetraurelia	
Q6BGL9	Ser/Thr	I	III	VII	Paramecium tetraurelia	
Q0U3Q5	Ser/Thr	I	III	VII	Phaeosphaeria nodorum	
Q0UF87	Ser/Thr	I	III	VII	Phaeosphaeria nodorum	
Q0UIS3	Ser/Thr	I	III	VII	Phaeosphaeria nodorum	
Q7DMT0	Ser/Thr	I	III	VII	Pisum sativum	
Q4YJL2	Ser/Thr	I	III	VII	Plasmodium berghei	
Q4YQB2	Ser/Thr	I	III	VII	Plasmodium berghei	
Q4YVB8	Ser/Thr	I	III	VII	Plasmodium berghei	
Q4YVG2	Ser/Thr	I	III	VII	Plasmodium berghei	
Q4YW20	Ser/Thr	I	III	VII	Plasmodium berghei	
Q4XMV3	Ser/Thr	I	III	VII	Plasmodium chabaudi	
Q4XNV1	Ser/Thr	I	III	VII	Plasmodium chabaudi	
Q4XUH0	Ser/Thr	I	III	VII	Plasmodium chabaudi	
Q8I719	Ser/Thr	I	III	VII	Plasmodium falciparum	2.7.11.12
O15906	Ser/Thr	I	III	VII	Plasmodium falciparum	
O44020	Ser/Thr	I	III	VII	Plasmodium falciparum	
Q6PLK2	Ser/Thr	I	III	VII	Plasmodium falciparum	
Q7K6A0	Ser/Thr	I	III	VII	Plasmodium falciparum	
Q8I4W3	Ser/Thr	I	III	VII	Plasmodium falciparum	

Table S2.3. (continuation)

UniProt	Label [1]	Two	Three	Seven	Species	EC Number
Q8MMZ4	Ser/Thr	I	III	VII	Plasmodium falciparum	
A5K0N4	Ser/Thr	I	III	VII	Plasmodium vivax	
A5K101	Ser/Thr	I	III	VII	Plasmodium vivax	
A5K8X1	Ser/Thr	I	III	VII	Plasmodium vivax	
A5KE97	Ser/Thr	I	III	VII	Plasmodium vivax	
Q26217	Ser/Thr	I	III	VII	Plasmodium yoelii	2.7.11.11
Q7PDS2	Ser/Thr	I	III	VII	Plasmodium yoelii	
Q7RE33	Ser/Thr	I	III	VII	Plasmodium yoelii	
Q7RSF6	Ser/Thr	I	III	VII	Plasmodium yoelii	
Q5RT63	Ser/Thr	I	III	VII	Pogonomyrmex barbatus	
Q5R472	Ser/Thr	I	III	VII	Pongo abelii	
Q5R9Y7	Ser/Thr	I	III	VII	Pongo abelii	
Q5RAZ7	Ser/Thr	I	III	VII	Pongo abelii	
Q5RC73	Ser/Thr	I	III	VII	Pongo abelii	
Q5RDZ9	Ser/Thr	I	III	VII	Pongo abelii	
Q5REF7	Ser/Thr	I	III	VII	Pongo abelii	
A1L1M0	Ser/Thr	I	III	VII	Rattus norvegicus	
P97548	Ser/Thr	I	III	VII	Rattus norvegicus	
P97549	Ser/Thr	I	III	VII	Rattus norvegicus	
Q05759	Ser/Thr	I	III	VII	Rattus norvegicus	
Q5BK52	Ser/Thr	I	III	VII	Rattus norvegicus	
Q66HL7	Ser/Thr	I	III	VII	Rattus norvegicus	
Q68G05	Ser/Thr	I	III	VII	Rattus norvegicus	
Q792R1	Ser/Thr	I	III	VII	Rattus norvegicus	
Q8TFJ3	Ser/Thr	I	III	VII	Rhizomucor racemosus	
A6ZKX3	Ser/Thr	I	III	VII	Saccharomyces cerevisiae	
A6ZMG0	Ser/Thr	I	III	VII	Saccharomyces cerevisiae	
A6ZQG8	Ser/Thr	I	III	VII	Saccharomyces cerevisiae	
A6ZTB2	Ser/Thr	I	III	VII	Saccharomyces cerevisiae	
A6ZTR2	Ser/Thr	I	III	VII	Saccharomyces cerevisiae	
A6ZW46	Ser/Thr	I	III	VII	Saccharomyces cerevisiae	
A6ZW51	Ser/Thr	I	III	VII	Saccharomyces cerevisiae	
A6ZZ79	Ser/Thr	I	III	VII	Saccharomyces cerevisiae	
A6ZZF8	Ser/Thr	I	III	VII	Saccharomyces cerevisiae	
A6ZZJ4	Ser/Thr	I	III	VII	Saccharomyces cerevisiae	
Q5BXD3	Ser/Thr	I	III	VII	Schistosoma japonicum	
Q5BXH5	Ser/Thr	I	III	VII	Schistosoma japonicum	
Q5C089	Ser/Thr	I	III	VII	Schistosoma japonicum	
Q5C098	Ser/Thr	I	III	VII	Schistosoma japonicum	
Q5C0U4	Ser/Thr	I	III	VII	Schistosoma japonicum	
Q5C2X7	Ser/Thr	I	III	VII	Schistosoma japonicum	
Q5C3E6	Ser/Thr	I	III	VII	Schistosoma japonicum	
Q5DC90	Ser/Thr	I	III	VII	Schistosoma japonicum	
Q5DDU1	Ser/Thr	I	III	VII	Schistosoma japonicum	
Q5DF14	Ser/Thr	I	III	VII	Schistosoma japonicum	
Q33DK6	Ser/Thr	I	III	VII	Schizophyllum commune	
Q33DK8	Ser/Thr	I	III	VII	Schizophyllum commune	
Q33DX3	Ser/Thr	I	III	VII	Schizophyllum commune	
Q33DX4	Ser/Thr	I	III	VII	Schizophyllum commune	
A7E748	Ser/Thr	I	III	VII	Sclerotinia sclerotiorum	
A7ECY2	Ser/Thr	I	III	VII	Sclerotinia sclerotiorum	
A7ETJ7	Ser/Thr	I	III	VII	Sclerotinia sclerotiorum	
A7F7E8	Ser/Thr	I	III	VII	Sclerotinia sclerotiorum	
A7F7J7	Ser/Thr	I	III	VII	Sclerotinia sclerotiorum	
A7FA24	Ser/Thr	I	III	VII	Sclerotinia sclerotiorum	
Q6QE15	Ser/Thr	I	III	VII	Sclerotinia sclerotiorum	

Table S2.3. (continuation)

UniProt	Label [1]	Two	Three	Seven	Species	EC Number
Q0KIH5	Ser/Thr	I	III	VII	Solanum lycopersicum	
Q56R02	Ser/Thr	I	III	VII	Solanum lycopersicum	
Q5I6E8	Ser/Thr	I	III	VII	Solanum lycopersicum	
Q50JB6	Ser/Thr	I	III	VII	Sordaria macrospora	
O73926	Ser/Thr	I	III	VII	Squalus acanthias	
O73927	Ser/Thr	I	III	VII	Squalus acanthias	
A0MLS8	Ser/Thr	I	III	VII	Sus scrofa	2.7.11.12
Q3YJM5	Ser/Thr	I	III	VII	Sus scrofa	2.7.11.12
P79276	Ser/Thr	I	III	VII	Sus scrofa	
Q22A18	Ser/Thr	I	III	VII	Tetrahymena thermophila	
Q22DD1	Ser/Thr	I	III	VII	Tetrahymena thermophila	
Q22GZ5	Ser/Thr	I	III	VII	Tetrahymena thermophila	
Q22HC2	Ser/Thr	I	III	VII	Tetrahymena thermophila	
Q22LX8	Ser/Thr	I	III	VII	Tetrahymena thermophila	
Q22RI5	Ser/Thr	I	III	VII	Tetrahymena thermophila	
Q231B5	Ser/Thr	I	III	VII	Tetrahymena thermophila	
Q231C9	Ser/Thr	I	III	VII	Tetrahymena thermophila	
Q233E6	Ser/Thr	I	III	VII	Tetrahymena thermophila	
Q233N6	Ser/Thr	I	III	VII	Tetrahymena thermophila	
Q234E6	Ser/Thr	I	III	VII	Tetrahymena thermophila	
Q237P3	Ser/Thr	I	III	VII	Tetrahymena thermophila	
Q23DN8	Ser/Thr	I	III	VII	Tetrahymena thermophila	
Q23G03	Ser/Thr	I	III	VII	Tetrahymena thermophila	
Q23KG5	Ser/Thr	I	III	VII	Tetrahymena thermophila	
Q23NJ5	Ser/Thr	I	III	VII	Tetrahymena thermophila	
Q23PX4	Ser/Thr	I	III	VII	Tetrahymena thermophila	
Q23TE4	Ser/Thr	I	III	VII	Tetrahymena thermophila	
Q23YB5	Ser/Thr	I	III	VII	Tetrahymena thermophila	
Q240P0	Ser/Thr	I	III	VII	Tetrahymena thermophila	
Q245J7	Ser/Thr	I	III	VII	Tetrahymena thermophila	
Q24BY8	Ser/Thr	I	III	VII	Tetrahymena thermophila	
Q24DD1	Ser/Thr	I	III	VII	Tetrahymena thermophila	
Q24FD5	Ser/Thr	I	III	VII	Tetrahymena thermophila	
Q24GE7	Ser/Thr	I	III	VII	Tetrahymena thermophila	
Q24HP6	Ser/Thr	I	III	VII	Tetrahymena thermophila	
Q4SGU4	Ser/Thr	I	III	VII	Tetraodon nigroviridis	2.7.11.1
Q4RI65	Ser/Thr	I	III	VII	Tetraodon nigroviridis	
Q4RR21	Ser/Thr	I	III	VII	Tetraodon nigroviridis	
Q4RY94	Ser/Thr	I	III	VII	Tetraodon nigroviridis	
Q4S021	Ser/Thr	I	III	VII	Tetraodon nigroviridis	
Q4S0Y4	Ser/Thr	I	III	VII	Tetraodon nigroviridis	
Q4S1K3	Ser/Thr	I	III	VII	Tetraodon nigroviridis	
Q4S1T0	Ser/Thr	I	III	VII	Tetraodon nigroviridis	
Q4S4R0	Ser/Thr	I	III	VII	Tetraodon nigroviridis	
Q4S6S5	Ser/Thr	I	III	VII	Tetraodon nigroviridis	
Q4S7C1	Ser/Thr	I	III	VII	Tetraodon nigroviridis	
Q4S7T8	Ser/Thr	I	III	VII	Tetraodon nigroviridis	
Q4S7Y9	Ser/Thr	I	III	VII	Tetraodon nigroviridis	
Q4S8Z2	Ser/Thr	I	III	VII	Tetraodon nigroviridis	
Q4S9F4	Ser/Thr	I	III	VII	Tetraodon nigroviridis	
Q4SBL9	Ser/Thr	I	III	VII	Tetraodon nigroviridis	
Q4SE16	Ser/Thr	I	III	VII	Tetraodon nigroviridis	
Q4SFC2	Ser/Thr	I	III	VII	Tetraodon nigroviridis	
Q4SFH3	Ser/Thr	I	III	VII	Tetraodon nigroviridis	
Q4SHY0	Ser/Thr	I	III	VII	Tetraodon nigroviridis	
Q4SK04	Ser/Thr	I	III	VII	Tetraodon nigroviridis	

Table S2.3. (continuation)

UniProt	Label [1]	Two	Three	Seven	Species	EC Number
Q4SL71	Ser/Thr	I	III	VII	Tetraodon nigroviridis	
Q4SLK1	Ser/Thr	I	III	VII	Tetraodon nigroviridis	
Q4SLR2	Ser/Thr	I	III	VII	Tetraodon nigroviridis	
Q4SP51	Ser/Thr	I	III	VII	Tetraodon nigroviridis	
Q4SYX7	Ser/Thr	I	III	VII	Tetraodon nigroviridis	
Q4SYY0	Ser/Thr	I	III	VII	Tetraodon nigroviridis	
Q4T3F0	Ser/Thr	I	III	VII	Tetraodon nigroviridis	
Q4T5U2	Ser/Thr	I	III	VII	Tetraodon nigroviridis	
Q4T638	Ser/Thr	I	III	VII	Tetraodon nigroviridis	
Q4T7Y9	Ser/Thr	I	III	VII	Tetraodon nigroviridis	
Q4T8X6	Ser/Thr	I	III	VII	Tetraodon nigroviridis	
Q4TBD9	Ser/Thr	I	III	VII	Tetraodon nigroviridis	
Q4UF82	Ser/Thr	I	III	VII	Theileria annulata	2.7.1.37
Q4UBS1	Ser/Thr	I	III	VII	Theileria annulata	
Q4UE84	Ser/Thr	I	III	VII	Theileria annulata	
Q4MZK5	Ser/Thr	I	III	VII	Theileria parva	2.7.11.1
Q4N5B2	Ser/Thr	I	III	VII	Theileria parva	
Q58G65	Ser/Thr	I	III	VII	Toxoplasma gondii	
Q8MMP4	Ser/Thr	I	III	VII	Toxoplasma gondii	
Q8MMZ7	Ser/Thr	I	III	VII	Toxoplasma gondii	
Q9BMY6	Ser/Thr	I	III	VII	Toxoplasma gondii	
Q99012	Ser/Thr	I	III	VII	Trichoderma reesei	
A2D9C1	Ser/Thr	I	III	VII	Trichomonas vaginalis	
A2DA20	Ser/Thr	I	III	VII	Trichomonas vaginalis	
A2DB15	Ser/Thr	I	III	VII	Trichomonas vaginalis	
A2DHQ7	Ser/Thr	I	III	VII	Trichomonas vaginalis	
A2DJV4	Ser/Thr	I	III	VII	Trichomonas vaginalis	
A2DL59	Ser/Thr	I	III	VII	Trichomonas vaginalis	
A2DLD2	Ser/Thr	I	III	VII	Trichomonas vaginalis	
A2DNC5	Ser/Thr	I	III	VII	Trichomonas vaginalis	
A2DP41	Ser/Thr	I	III	VII	Trichomonas vaginalis	
A2DRK1	Ser/Thr	I	III	VII	Trichomonas vaginalis	
A2DS63	Ser/Thr	I	III	VII	Trichomonas vaginalis	
A2DV83	Ser/Thr	I	III	VII	Trichomonas vaginalis	
A2DVS0	Ser/Thr	I	III	VII	Trichomonas vaginalis	
A2DW86	Ser/Thr	I	III	VII	Trichomonas vaginalis	
A2DYS1	Ser/Thr	I	III	VII	Trichomonas vaginalis	
A2E224	Ser/Thr	I	III	VII	Trichomonas vaginalis	
A2E382	Ser/Thr	I	III	VII	Trichomonas vaginalis	
A2E474	Ser/Thr	I	III	VII	Trichomonas vaginalis	
A2E5K8	Ser/Thr	I	III	VII	Trichomonas vaginalis	
A2E8A4	Ser/Thr	I	III	VII	Trichomonas vaginalis	
A2E8U1	Ser/Thr	I	III	VII	Trichomonas vaginalis	
A2E987	Ser/Thr	I	III	VII	Trichomonas vaginalis	
A2ECT2	Ser/Thr	I	III	VII	Trichomonas vaginalis	
A2EE93	Ser/Thr	I	III	VII	Trichomonas vaginalis	
A2EED1	Ser/Thr	I	III	VII	Trichomonas vaginalis	
A2EEZ5	Ser/Thr	I	III	VII	Trichomonas vaginalis	
A2EI78	Ser/Thr	I	III	VII	Trichomonas vaginalis	
A2EJ98	Ser/Thr	I	III	VII	Trichomonas vaginalis	
A2ELF7	Ser/Thr	I	III	VII	Trichomonas vaginalis	
A2EPG5	Ser/Thr	I	III	VII	Trichomonas vaginalis	
A2ET74	Ser/Thr	I	III	VII	Trichomonas vaginalis	
A2ETC0	Ser/Thr	I	III	VII	Trichomonas vaginalis	
A2ETU6	Ser/Thr	I	III	VII	Trichomonas vaginalis	
A2EV93	Ser/Thr	I	III	VII	Trichomonas vaginalis	

Table S2.3. (continuation)

UniProt	Label [1]	Two	Three	Seven	Species	EC Number
A2EWI0	Ser/Thr	I	III	VII	Trichomonas vaginalis	
A2EX40	Ser/Thr	I	III	VII	Trichomonas vaginalis	
A2EY79	Ser/Thr	I	III	VII	Trichomonas vaginalis	
A2EZG8	Ser/Thr	I	III	VII	Trichomonas vaginalis	
A2F0I9	Ser/Thr	I	III	VII	Trichomonas vaginalis	
A2F4A4	Ser/Thr	I	III	VII	Trichomonas vaginalis	
A2F8E8	Ser/Thr	I	III	VII	Trichomonas vaginalis	
A2F9U6	Ser/Thr	I	III	VII	Trichomonas vaginalis	
A2FA91	Ser/Thr	I	III	VII	Trichomonas vaginalis	
A2FAW3	Ser/Thr	I	III	VII	Trichomonas vaginalis	
A2FBN5	Ser/Thr	I	III	VII	Trichomonas vaginalis	
A2FDD2	Ser/Thr	I	III	VII	Trichomonas vaginalis	
A2FHJ2	Ser/Thr	I	III	VII	Trichomonas vaginalis	
A2FIJ5	Ser/Thr	I	III	VII	Trichomonas vaginalis	
A2FKC9	Ser/Thr	I	III	VII	Trichomonas vaginalis	
A2FLX7	Ser/Thr	I	III	VII	Trichomonas vaginalis	
A2FMG9	Ser/Thr	I	III	VII	Trichomonas vaginalis	
A2FNQ5	Ser/Thr	I	III	VII	Trichomonas vaginalis	
A2FPF2	Ser/Thr	I	III	VII	Trichomonas vaginalis	
A2FY70	Ser/Thr	I	III	VII	Trichomonas vaginalis	
A2FYJ4	Ser/Thr	I	III	VII	Trichomonas vaginalis	
A2FYT0	Ser/Thr	I	III	VII	Trichomonas vaginalis	
A2FZ96	Ser/Thr	I	III	VII	Trichomonas vaginalis	
A2FZY1	Ser/Thr	I	III	VII	Trichomonas vaginalis	
A2G0T7	Ser/Thr	I	III	VII	Trichomonas vaginalis	
A2G1Q9	Ser/Thr	I	III	VII	Trichomonas vaginalis	
A2G288	Ser/Thr	I	III	VII	Trichomonas vaginalis	
A2G3J0	Ser/Thr	I	III	VII	Trichomonas vaginalis	
A2G3R6	Ser/Thr	I	III	VII	Trichomonas vaginalis	
A2GBV0	Ser/Thr	I	III	VII	Trichomonas vaginalis	
A2GEQ5	Ser/Thr	I	III	VII	Trichomonas vaginalis	
Q582V7	Ser/Thr	I	III	VII	Trypanosoma brucei	2.7.1.-
Q388U5	Ser/Thr	I	III	VII	Trypanosoma brucei	2.7.11.1
Q384A2	Ser/Thr	I	III	VII	Trypanosoma brucei	
Q38DR5	Ser/Thr	I	III	VII	Trypanosoma brucei	
Q38DS1	Ser/Thr	I	III	VII	Trypanosoma brucei	
Q3S1K7	Ser/Thr	I	III	VII	Trypanosoma brucei	
Q3S1L1	Ser/Thr	I	III	VII	Trypanosoma brucei	
Q584T1	Ser/Thr	I	III	VII	Trypanosoma brucei	
Q8WSK3	Ser/Thr	I	III	VII	Trypanosoma brucei	
Q965D1	Ser/Thr	I	III	VII	Trypanosoma brucei	
Q4D4Z9	Ser/Thr	I	III	VII	Trypanosoma cruzi	2.7.11.1
O60947	Ser/Thr	I	III	VII	Trypanosoma cruzi	
Q4D6D3	Ser/Thr	I	III	VII	Trypanosoma cruzi	
Q4DL90	Ser/Thr	I	III	VII	Trypanosoma cruzi	
Q4DSM2	Ser/Thr	I	III	VII	Trypanosoma cruzi	
Q4DSQ1	Ser/Thr	I	III	VII	Trypanosoma cruzi	
Q4DXB5	Ser/Thr	I	III	VII	Trypanosoma cruzi	
Q4E2L0	Ser/Thr	I	III	VII	Trypanosoma cruzi	
Q4E4S9	Ser/Thr	I	III	VII	Trypanosoma cruzi	
Q4E4T3	Ser/Thr	I	III	VII	Trypanosoma cruzi	
Q8WQR2	Ser/Thr	I	III	VII	Trypanosoma cruzi	
O13367	Ser/Thr	I	III	VII	Ustilago maydis	
Q7LVQ2	Ser/Thr	I	III	VII	Ustilago maydis	
Q99079	Ser/Thr	I	III	VII	Ustilago maydis	
A7TE02	Ser/Thr	I	III	VII	Vanderwaltozyma polyspora	

Table S2.3. (continuation)

UniProt	Label [1]	Two	Three	Seven	Species	EC Number
A7TFJ0	Ser/Thr	I	III	VII	Vanderwaltozyma polyspora	
A7TJ45	Ser/Thr	I	III	VII	Vanderwaltozyma polyspora	
A7TL28	Ser/Thr	I	III	VII	Vanderwaltozyma polyspora	
A7TPV4	Ser/Thr	I	III	VII	Vanderwaltozyma polyspora	
A7TT78	Ser/Thr	I	III	VII	Vanderwaltozyma polyspora	
A7TTQ0	Ser/Thr	I	III	VII	Vanderwaltozyma polyspora	
A1YTU8	Ser/Thr	I	III	VII	Vespula vulgaris	2.7.11.12
Q8H935	Ser/Thr	I	III	VII	Vicia faba	
A5BB68	Ser/Thr	I	III	VII	Vitis vinifera	
A5C7A9	Ser/Thr	I	III	VII	Vitis vinifera	
O73732	Ser/Thr	I	III	VII	Xenopus laevis	2.7.11.1
Q6DCH1	Ser/Thr	I	III	VII	Xenopus laevis	2.7.11.1
Q6GLY8	Ser/Thr	I	III	VII	Xenopus laevis	2.7.11.1
Q6GNN5	Ser/Thr	I	III	VII	Xenopus laevis	2.7.11.1
Q6GPN6	Ser/Thr	I	III	VII	Xenopus laevis	2.7.11.1
Q9W6Y9	Ser/Thr	I	III	VII	Xenopus laevis	2.7.11.1
Q6GPV8	Ser/Thr	I	III	VII	Xenopus laevis	2.7.11.12
Q00603	Ser/Thr	I	III	VII	Xenopus laevis	
Q4V7S7	Ser/Thr	I	III	VII	Xenopus laevis	
Q52KZ3	Ser/Thr	I	III	VII	Xenopus laevis	
Q5XH04	Ser/Thr	I	III	VII	Xenopus laevis	
Q68F06	Ser/Thr	I	III	VII	Xenopus laevis	
Q6DCW4	Ser/Thr	I	III	VII	Xenopus laevis	
Q6DE61	Ser/Thr	I	III	VII	Xenopus laevis	
Q6GQ72	Ser/Thr	I	III	VII	Xenopus laevis	
Q7ZWV0	Ser/Thr	I	III	VII	Xenopus laevis	
Q8AVM0	Ser/Thr	I	III	VII	Xenopus laevis	
Q90WN2	Ser/Thr	I	III	VII	Xenopus laevis	
Q90WN3	Ser/Thr	I	III	VII	Xenopus laevis	
Q28DP9	Ser/Thr	I	III	VII	Xenopus tropicalis	2.7.11.1
Q5BKK4	Ser/Thr	I	III	VII	Xenopus tropicalis	2.7.11.1
A0JM25	Ser/Thr	I	III	VII	Xenopus tropicalis	
A0JM68	Ser/Thr	I	III	VII	Xenopus tropicalis	
Q0VFF1	Ser/Thr	I	III	VII	Xenopus tropicalis	
Q28DL5	Ser/Thr	I	III	VII	Xenopus tropicalis	
Q28GZ8	Ser/Thr	I	III	VII	Xenopus tropicalis	
Q28J06	Ser/Thr	I	III	VII	Xenopus tropicalis	
Q6DF20	Ser/Thr	I	III	VII	Xenopus tropicalis	
Q6P2Z5	Ser/Thr	I	III	VII	Xenopus tropicalis	
Q6C6T3	Ser/Thr	I	III	VII	Yarrowia lipolytica	
Q6C936	Ser/Thr	I	III	VII	Yarrowia lipolytica	
Q6CBU3	Ser/Thr	I	III	VII	Yarrowia lipolytica	
Q6CCL9	Ser/Thr	I	III	VII	Yarrowia lipolytica	
Q6CD34	Ser/Thr	I	III	VII	Yarrowia lipolytica	
Q6CFM7	Ser/Thr	I	III	VII	Yarrowia lipolytica	
Q0QVS5	Ser/Thr	I	III	VII	Zea mays	
Q6TQF8	Ser/Thr	I	III	VII	Zea mays	
Q0WM41	Tyr	I	II	V	Arabidopsis thaliana	
Q0WUI6	Tyr	I	II	V	Arabidopsis thaliana	
Q56WL1	Tyr	I	II	V	Arabidopsis thaliana	
Q8L625	Tyr	I	II	V	Arabidopsis thaliana	
Q8LPH3	Tyr	I	II	V	Arabidopsis thaliana	
Q93YU0	Tyr	I	II	V	Arabidopsis thaliana	
Q9C833	Tyr	I	II	V	Arabidopsis thaliana	
Q9C902	Tyr	I	II	V	Arabidopsis thaliana	
Q9C903	Tyr	I	II	V	Arabidopsis thaliana	

Table S2.3. (continuation)

UniProt	Label [1]	Two	Three	Seven	Species	EC Number
Q9C9U5	Tyr	I	II	V	Arabidopsis thaliana	
Q9C9V5	Tyr	I	II	V	Arabidopsis thaliana	
Q9LYI8	Tyr	I	II	V	Arabidopsis thaliana	
Q7X8K7	Tyr	I	II	V	Brassica juncea	
Q7XAV0	Tyr	I	II	V	Cucumis sativus	
Q90ZY8	Tyr	I	II	V	Danio rerio	
Q7X9G6	Tyr	I	II	V	Delphinium 'MagicFountains	
Q70YI7	Tyr	I	II	V	Hordeum vulgare	
Q4R8A9	Tyr	I	II	V	Macaca fascicularis	
A2T3V2	Tyr	I	II	V	Malus domestica	
A2T3V3	Tyr	I	II	V	Malus domestica	
A2T3V4	Tyr	I	II	V	Malus domestica	
Q4VRV4	Tyr	I	II	V	Malus domestica	
Q1SMZ9	Tyr	I	II	V	Medicago truncatula	
Q3TRG2	Tyr	I	II	V	Mus musculus	
A2XCS1	Tyr	I	II	V	Oryza sativa	
A2YAZ9	Tyr	I	II	V	Oryza sativa	
Q0DDD4	Tyr	I	II	V	Oryza sativa	
Q0E0S0	Tyr	I	II	V	Oryza sativa	
Q0IWW6	Tyr	I	II	V	Oryza sativa	
Q10RG4	Tyr	I	II	V	Oryza sativa	
Q337J8	Tyr	I	II	V	Oryza sativa	
Q650V1	Tyr	I	II	V	Oryza sativa	
Q67UL6	Tyr	I	II	V	Oryza sativa	
Q67W58	Tyr	I	II	V	Oryza sativa	
Q6H6Q5	Tyr	I	II	V	Oryza sativa	
Q6YW44	Tyr	I	II	V	Oryza sativa	
Q7XYY6	Tyr	I	II	V	Oryza sativa	
Q8LNV7	Tyr	I	II	V	Oryza sativa	
Q9FPR5	Tyr	I	II	V	Oryza sativa	
A4RXJ0	Tyr	I	II	V	Ostreococcus lucimarinus	
A4S5I9	Tyr	I	II	V	Ostreococcus lucimarinus	
Q0VJB8	Tyr	I	II	V	Platanus acerifolia	
A1INL8	Tyr	I	II	V	Prunus domestica	
Q2M489	Tyr	I	II	V	Prunus persica	
Q93XL9	Tyr	I	II	V	Rosa hybrid	
Q9ARG9	Tyr	I	II	V	Rosa hybrid	
O24027	Tyr	I	II	V	Solanum lycopersicum	
Q5YKK5	Tyr	I	II	V	Solanum lycopersicum	
Q5YKK6	Tyr	I	II	V	Solanum lycopersicum	
Q9ZSD8	Tyr	I	II	V	Solanum lycopersicum	
Q9ZSD9	Tyr	I	II	V	Solanum lycopersicum	
Q4RNR9	Tyr	I	II	V	Tetraodon nigroviridis	
A2EJK9	Tyr	I	II	V	Trichomonas vaginalis	
A2EY26	Tyr	I	II	V	Trichomonas vaginalis	
A5AP74	Tyr	I	II	V	Vitis vinifera	
A5AQH5	Tyr	I	II	V	Vitis vinifera	
A5ATQ8	Tyr	I	II	V	Vitis vinifera	
A5BNJ1	Tyr	I	II	V	Vitis vinifera	
A0JN96	Tyr	I	II	VI	Bos taurus	
Q69U56	Tyr	I	II	VI	Oryza sativa	
Q5RAR7	Tyr	I	II	VI	Pongo abelii	
Q4T0K5	Tyr	I	II	VI	Tetraodon nigroviridis	
A2DGV6	Tyr	I	III	VI	Trichomonas vaginalis	
Q6K3D4	Tyr	I	III	VII	Oryza sativa	
Q17BM1	Tyr	II	II	I	Aedes aegypti	

Table S2.3. (continuation)

UniProt	Label [1]	Two	Three	Seven	Species	EC Number
Q17J18	Tyr	II	II	I	Aedes aegypti	
Q5TTJ6	Tyr	II	II	I	Anopheles gambiae	
Q1JUB8	Tyr	II	II	I	Bombyx mori	2.7.10.1
Q95M13	Tyr	II	II	I	Bos taurus	2.7.10.1
A7E310	Tyr	II	II	I	Bos taurus	
Q95N25	Tyr	II	II	I	Bos taurus	
Q2EKC1	Tyr	II	II	I	Branchiostoma belcheri	2.7.10.1
Q9TT07	Tyr	II	II	I	Canis familiaris	2.7.10.1
Q14TE4	Tyr	II	II	I	Cynops pyrrhogaster	
Q805B9	Tyr	II	II	I	Danio rerio	2.7.10.1
Q8AYP3	Tyr	II	II	I	Danio rerio	2.7.10.1
Q4VBK2	Tyr	II	II	I	Danio rerio	
Q307W2	Tyr	II	II	I	Felis catus	
Q90749	Tyr	II	II	I	Gallus gallus	2.7.10.1
A8E633	Tyr	II	II	I	Homo sapiens	
Q0IJ44	Tyr	II	II	I	Homo sapiens	
Q59F30	Tyr	II	II	I	Homo sapiens	
Q8NI15	Tyr	II	II	I	Homo sapiens	
Q8NI16	Tyr	II	II	I	Homo sapiens	
A1YYM3	Tyr	II	II	I	Mus musculus	2.7.10.1
A1YYM4	Tyr	II	II	I	Mus musculus	2.7.10.1
A1YYM5	Tyr	II	II	I	Mus musculus	2.7.10.1
A1YYM6	Tyr	II	II	I	Mus musculus	2.7.10.1
A1YYM7	Tyr	II	II	I	Mus musculus	2.7.10.1
A1YYM8	Tyr	II	II	I	Mus musculus	2.7.10.1
A1YYM9	Tyr	II	II	I	Mus musculus	2.7.10.1
A1YYN0	Tyr	II	II	I	Mus musculus	2.7.10.1
A1YYN2	Tyr	II	II	I	Mus musculus	2.7.10.1
A1YYN5	Tyr	II	II	I	Mus musculus	2.7.10.1
A1YYN6	Tyr	II	II	I	Mus musculus	2.7.10.1
A1YYN7	Tyr	II	II	I	Mus musculus	2.7.10.1
A1YYN9	Tyr	II	II	I	Mus musculus	2.7.10.1
A1YYP0	Tyr	II	II	I	Mus musculus	2.7.10.1
A1YYP1	Tyr	II	II	I	Mus musculus	2.7.10.1
Q3UPE1	Tyr	II	II	I	Mus musculus	2.7.10.1
Q60818	Tyr	II	II	I	Mus musculus	2.7.10.1
Q61563	Tyr	II	II	I	Mus musculus	2.7.10.1
Q7TSI8	Tyr	II	II	I	Mus musculus	2.7.10.1
Q8CIM9	Tyr	II	II	I	Mus musculus	2.7.10.1
Q99052	Tyr	II	II	I	Mus musculus	2.7.10.1
A1YYN1	Tyr	II	II	I	Mus musculus	
A1YYN4	Tyr	II	II	I	Mus musculus	
A1YYN8	Tyr	II	II	I	Mus musculus	
Q3TJ05	Tyr	II	II	I	Mus musculus	
Q3TQL1	Tyr	II	II	I	Mus musculus	
Q8CBY7	Tyr	II	II	I	Mus musculus	
A7RZ71	Tyr	II	II	I	Nematostella vectensis	2.7.10.1
A7RHA0	Tyr	II	II	I	Nematostella vectensis	
A7RHA4	Tyr	II	II	I	Nematostella vectensis	
Q91150	Tyr	II	II	I	Notophthalmus viridescens	2.7.10.1
Q91146	Tyr	II	II	I	Notophthalmus viridescens	
Q9TTZ3	Tyr	II	II	I	Oryctolagus cuniculus	2.7.10.1
Q5XUI1	Tyr	II	II	I	Ovis aries	
Q5XUI2	Tyr	II	II	I	Ovis aries	
A0SLB7	Tyr	II	II	I	Paracentrotus lividus	
Q5R8Q3	Tyr	II	II	I	Pongo abelii	2.7.10.1

Table S2.3. (continuation)

UniProt	Label [1]	Two	Three	Seven	Species	EC Number
Q5RF81	Tyr	II	II	I	Pongo abelii	
Q63827	Tyr	II	II	I	Rattus norvegicus	2.7.10.1
Q9JHX9	Tyr	II	II	I	Rattus norvegicus	2.7.10.1
Q64334	Tyr	II	II	I	Rattus norvegicus	
Q63710	Tyr	II	II	I	Rattus rattus	
Q63711	Tyr	II	II	I	Rattus rattus	
Q63709	Tyr	II	II	I	Rattus sp	
Q1JUB7	Tyr	II	II	I	Spodoptera frugiperda	2.7.10.1
A5A754	Tyr	II	II	I	Sus scrofa	2.7.10.1
Q4SPY1	Tyr	II	II	I	Tetraodon nigroviridis	2.7.10.1
Q6DD66	Tyr	II	II	I	Xenopus laevis	2.7.10.1
Q6GNP8	Tyr	II	II	I	Xenopus laevis	2.7.10.1
Q6GNS5	Tyr	II	II	I	Xenopus laevis	2.7.10.1
Q91742	Tyr	II	II	I	Xenopus laevis	2.7.10.1
Q91897	Tyr	II	II	I	Xenopus laevis	2.7.10.1
Q9DGK3	Tyr	II	II	I	Xenopus laevis	2.7.10.1
Q9PS96	Tyr	II	II	I	Xenopus laevis	2.7.10.1
Q9PSV8	Tyr	II	II	I	Xenopus laevis	2.7.10.1
Q9PSV9	Tyr	II	II	I	Xenopus laevis	2.7.10.1
Q28GC1	Tyr	II	II	I	Xenopus tropicalis	2.7.10.1
Q28J96	Tyr	II	II	I	Xenopus tropicalis	2.7.10.1
Q28CQ6	Tyr	II	II	I	Xenopus tropicalis	
Q17CE8	Tyr	II	II	II	Aedes aegypti	2.7.10.2
Q17I40	Tyr	II	II	II	Aedes aegypti	2.7.10.2
Q16XS6	Tyr	II	II	II	Aedes aegypti	
Q7PPB4	Tyr	II	II	II	Anopheles gambiae	2.7.10.2
Q7Q5D3	Tyr	II	II	II	Anopheles gambiae	2.7.10.2
A7UTI5	Tyr	II	II	II	Anopheles gambiae	
Q7Q7W3	Tyr	II	II	II	Anopheles gambiae	
O77050	Tyr	II	II	II	Anthocidaris crassispina	2.7.10.2
Q6R1Y3	Tyr	II	II	II	Asterina miniata	2.7.10.2
Q6R1Y4	Tyr	II	II	II	Asterina miniata	2.7.10.2
Q6R1Y5	Tyr	II	II	II	Asterina miniata	2.7.10.2
Q64817	Tyr	II	II	II	Avian sarcoma	
A5PKG9	Tyr	II	II	II	Bos taurus	2.7.10.2
A7MB57	Tyr	II	II	II	Bos taurus	2.7.10.2
Q08DU6	Tyr	II	II	II	Bos taurus	2.7.10.2
Q3ZCM0	Tyr	II	II	II	Bos taurus	2.7.10.2
Q9U8V1	Tyr	II	II	II	Branchiostoma belcheri	
Q9U8V2	Tyr	II	II	II	Branchiostoma belcheri	
Q9U8V3	Tyr	II	II	II	Branchiostoma belcheri	
O45539	Tyr	II	II	II	Caenorhabditis elegans	2.7.10.2
A4UX72	Tyr	II	II	II	Carassius auratus	2.7.10.2
A4UX73	Tyr	II	II	II	Carassius auratus	2.7.10.2
A1A5H8	Tyr	II	II	II	Danio rerio	2.7.10.2
A5WWJ6	Tyr	II	II	II	Danio rerio	2.7.10.2
Q08BY6	Tyr	II	II	II	Danio rerio	2.7.10.2
Q1JPZ3	Tyr	II	II	II	Danio rerio	2.7.10.2
Q1RLY5	Tyr	II	II	II	Danio rerio	2.7.10.2
Q5TYU7	Tyr	II	II	II	Danio rerio	2.7.10.2
Q66HZ1	Tyr	II	II	II	Danio rerio	2.7.10.2
Q66I04	Tyr	II	II	II	Danio rerio	2.7.10.2
Q6EWH2	Tyr	II	II	II	Danio rerio	2.7.10.2
Q6TPQ4	Tyr	II	II	II	Danio rerio	2.7.10.2
Q8AWF1	Tyr	II	II	II	Danio rerio	2.7.10.2
Q5U175	Tyr	II	II	II	Drosophila melanogaster	

Table S2.3. (continuation)

UniProt	Label [1]	Two	Three	Seven	Species	EC Number
Q29D50	Tyr	II	II	II	Drosophila pseudoobscura	2.7.10.2
Q29EB5	Tyr	II	II	II	Drosophila pseudoobscura	2.7.10.2
Q8WSU2	Tyr	II	II	II	Ephydatia fluviatilis	2.7.10.2
Q8WSU3	Tyr	II	II	II	Ephydatia fluviatilis	2.7.10.2
Q8WSU4	Tyr	II	II	II	Ephydatia fluviatilis	2.7.10.2
Q8WSU5	Tyr	II	II	II	Ephydatia fluviatilis	2.7.10.2
Q9Y1Z1	Tyr	II	II	II	Ephydatia fluviatilis	2.7.10.2
Q9U8V4	Tyr	II	II	II	Eptatretus burgeri	
Q9U8V5	Tyr	II	II	II	Eptatretus burgeri	
Q9U8V6	Tyr	II	II	II	Eptatretus burgeri	
Q28414	Tyr	II	II	II	Feline sarcoma	
Q8QGJ9	Tyr	II	II	II	Fugu rubripes	2.7.10.2
Q5ZMB9	Tyr	II	II	II	Gallus gallus	2.7.10.2
Q05D26	Tyr	II	II	II	Homo sapiens	2.7.10.2
Q573B4	Tyr	II	II	II	Homo sapiens	2.7.10.2
Q6NUK7	Tyr	II	II	II	Homo sapiens	2.7.10.2
Q9H7V3	Tyr	II	II	II	Homo sapiens	
Q95M31	Tyr	II	II	II	Hylobates sp	2.7.10.2
Q95M32	Tyr	II	II	II	Hylobates sp	2.7.10.2
Q9PVU9	Tyr	II	II	II	Lampetra reissneri	
Q9PVV0	Tyr	II	II	II	Lampetra reissneri	
Q4R6L8	Tyr	II	II	II	Macaca fascicularis	2.7.10.2
Q7YZH8	Tyr	II	II	II	Monosiga brevicollis	
Q0PDJ2	Tyr	II	II	II	Monosiga ovata	
Q0PDJ3	Tyr	II	II	II	Monosiga ovata	
Q0PDJ4	Tyr	II	II	II	Monosiga ovata	
Q05DV0	Tyr	II	II	II	Mus musculus	2.7.10.2
Q3SYK5	Tyr	II	II	II	Mus musculus	2.7.10.2
Q3TAT8	Tyr	II	II	II	Mus musculus	2.7.10.2
Q3TCS3	Tyr	II	II	II	Mus musculus	2.7.10.2
Q3TJI7	Tyr	II	II	II	Mus musculus	2.7.10.2
Q3TLX4	Tyr	II	II	II	Mus musculus	2.7.10.2
Q3U6Q5	Tyr	II	II	II	Mus musculus	2.7.10.2
Q3UKD6	Tyr	II	II	II	Mus musculus	2.7.10.2
Q6GTF2	Tyr	II	II	II	Mus musculus	2.7.10.2
Q80XU2	Tyr	II	II	II	Mus musculus	2.7.10.2
Q8BPC1	Tyr	II	II	II	Mus musculus	2.7.10.2
Q8C762	Tyr	II	II	II	Mus musculus	2.7.10.2
Q8CBP1	Tyr	II	II	II	Mus musculus	2.7.10.2
Q8CEI0	Tyr	II	II	II	Mus musculus	2.7.10.2
Q3TM13	Tyr	II	II	II	Mus musculus	
Q3V2W1	Tyr	II	II	II	Mus musculus	
A7RN18	Tyr	II	II	II	Nematostella vectensis	
Q1X7P3	Tyr	II	II	II	Oncorhynchus mykiss	2.7.10.2
Q1X7P4	Tyr	II	II	II	Oncorhynchus mykiss	2.7.10.2
Q5REC4	Tyr	II	II	II	Pongo abelii	2.7.10.2
A0F037	Tyr	II	II	II	Psetta maxima	2.7.10.2
Q4FZR6	Tyr	II	II	II	Rattus norvegicus	2.7.10.2
Q4KM97	Tyr	II	II	II	Rattus norvegicus	2.7.10.2
Q6AXQ3	Tyr	II	II	II	Rattus norvegicus	2.7.10.2
Q6P6U0	Tyr	II	II	II	Rattus norvegicus	2.7.10.2
Q99PW1	Tyr	II	II	II	Rattus norvegicus	2.7.10.2
O92806	Tyr	II	II	II	Rous sarcoma	
O92957	Tyr	II	II	II	Rous sarcoma	
O93080	Tyr	II	II	II	Rous sarcoma	
Q07461	Tyr	II	II	II	Rous sarcoma	

Table S2.3. (continuation)

UniProt	Label [1]	Two	Three	Seven	Species	EC Number
Q60567	Tyr	II	II	II	Rous sarcoma	
Q64993	Tyr	II	II	II	Rous sarcoma	
Q64994	Tyr	II	II	II	Rous sarcoma	
Q85477	Tyr	II	II	II	Rous sarcoma	
Q86362	Tyr	II	II	II	Rous sarcoma	
Q86363	Tyr	II	II	II	Rous sarcoma	
Q9DDK6	Tyr	II	II	II	Salmo salar	2.7.10.2
Q70DV1	Tyr	II	II	II	Schistosoma mansoni	2.7.10.2
Q9NH62	Tyr	II	II	II	Schistosoma mansoni	2.7.10.2
Q8WQM5	Tyr	II	II	II	Strongylocentrotus purpuratus	2.7.10.2
Q6E5A6	Tyr	II	II	II	Suberites domuncula	2.7.10.2
Q6E5A7	Tyr	II	II	II	Suberites domuncula	2.7.10.2
Q6E5A8	Tyr	II	II	II	Suberites domuncula	2.7.10.2
Q6E5A9	Tyr	II	II	II	Suberites domuncula	2.7.10.2
Q6E5B0	Tyr	II	II	II	Suberites domuncula	2.7.10.2
A1Y2K2	Tyr	II	II	II	Sus scrofa	
Q4RAT6	Tyr	II	II	II	Tetraodon nigroviridis	2.7.10.2
Q4RH41	Tyr	II	II	II	Tetraodon nigroviridis	2.7.10.2
Q4RML6	Tyr	II	II	II	Tetraodon nigroviridis	2.7.10.2
Q4RML8	Tyr	II	II	II	Tetraodon nigroviridis	2.7.10.2
Q4SJH9	Tyr	II	II	II	Tetraodon nigroviridis	2.7.10.2
Q4SY83	Tyr	II	II	II	Tetraodon nigroviridis	2.7.10.2
Q4T0U2	Tyr	II	II	II	Tetraodon nigroviridis	2.7.10.2
Q4RIJ4	Tyr	II	II	II	Tetraodon nigroviridis	
Q4RN20	Tyr	II	II	II	Tetraodon nigroviridis	
Q4RR72	Tyr	II	II	II	Tetraodon nigroviridis	
Q7LZH0	Tyr	II	II	II	Torpedo californica	2.7.10.2
Q7LZH1	Tyr	II	II	II	Torpedo californica	2.7.10.2
A1L2U4	Tyr	II	II	II	Xenopus laevis	2.7.10.2
O13064	Tyr	II	II	II	Xenopus laevis	2.7.10.2
O93411	Tyr	II	II	II	Xenopus laevis	2.7.10.2
Q2TAR1	Tyr	II	II	II	Xenopus laevis	2.7.10.2
Q32N74	Tyr	II	II	II	Xenopus laevis	2.7.10.2
Q498G3	Tyr	II	II	II	Xenopus laevis	2.7.10.2
Q6P282	Tyr	II	II	II	Xenopus laevis	2.7.10.2
Q6PF70	Tyr	II	II	II	Xenopus laevis	2.7.10.2
Q7ZX73	Tyr	II	II	II	Xenopus laevis	2.7.10.2
Q7ZYH5	Tyr	II	II	II	Xenopus laevis	2.7.10.2
Q28CN2	Tyr	II	II	II	Xenopus tropicalis	2.7.10.2
Q28IV0	Tyr	II	II	II	Xenopus tropicalis	2.7.10.2
Q5FW27	Tyr	II	II	II	Xenopus tropicalis	2.7.10.2
Q5MAS9	Tyr	II	II	II	Xenopus tropicalis	2.7.10.2
Q640S9	Tyr	II	II	II	Xenopus tropicalis	2.7.10.2
Q91952	Tyr	II	II	II	Xiphophorus xiphidium	2.7.10.2
O92809	Tyr	II	II	V	Abelson murine	
Q9PWS3	Tyr	II	II	V	Abelson murine	
Q16T43	Tyr	II	II	V	Aedes aegypti	2.7.10.1
Q170Z1	Tyr	II	II	V	Aedes aegypti	2.7.10.1
Q17E33	Tyr	II	II	V	Aedes aegypti	2.7.10.1
Q16G61	Tyr	II	II	V	Aedes aegypti	2.7.10.2
Q16L66	Tyr	II	II	V	Aedes aegypti	2.7.10.2
Q16XX9	Tyr	II	II	V	Aedes aegypti	2.7.10.2
Q17MF5	Tyr	II	II	V	Aedes aegypti	2.7.10.2
Q16FU4	Tyr	II	II	V	Aedes aegypti	
Q16R80	Tyr	II	II	V	Aedes aegypti	
Q16UV1	Tyr	II	II	V	Aedes aegypti	

Table S2.3. (continuation)

UniProt	Label [1]	Two	Three	Seven	Species	EC Number
Q173L5	Tyr	II	II	V	Aedes aegypti	
Q17C76	Tyr	II	II	V	Aedes aegypti	
Q17N20	Tyr	II	II	V	Aedes aegypti	
Q5TUS8	Tyr	II	II	V	Anopheles gambiae	2.7.10.1
Q7PV77	Tyr	II	II	V	Anopheles gambiae	2.7.10.1
Q7Q0F6	Tyr	II	II	V	Anopheles gambiae	2.7.10.1
Q7Q5T3	Tyr	II	II	V	Anopheles gambiae	2.7.10.1
Q7PML5	Tyr	II	II	V	Anopheles gambiae	2.7.10.2
Q7Q4A9	Tyr	II	II	V	Anopheles gambiae	2.7.10.2
Q7QE10	Tyr	II	II	V	Anopheles gambiae	2.7.10.2
A0NG93	Tyr	II	II	V	Anopheles gambiae	
Q7PRV9	Tyr	II	II	V	Anopheles gambiae	
Q7PT15	Tyr	II	II	V	Anopheles gambiae	
Q7PWP1	Tyr	II	II	V	Anopheles gambiae	
Q7PZ80	Tyr	II	II	V	Anopheles gambiae	
Q7Q4D9	Tyr	II	II	V	Anopheles gambiae	
Q7QAK4	Tyr	II	II	V	Anopheles gambiae	
Q7QHB8	Tyr	II	II	V	Anopheles gambiae	
Q7QIP0	Tyr	II	II	V	Anopheles gambiae	
O76148	Tyr	II	II	V	Anopheles sp	2.7.10.1
Q66XT2	Tyr	II	II	V	Anopheles stephensi	
Q5D184	Tyr	II	II	V	Apis mellifera	
Q9BKL8	Tyr	II	II	V	Aplysia californica	2.7.10.1
A4UU47	Tyr	II	II	V	Aplysia californica	
Q5IJ68	Tyr	II	II	V	Aplysia californica	
Q6R1Y6	Tyr	II	II	V	Asterina miniata	2.7.10.2
Q9NJV5	Tyr	II	II	V	Biomphalaria glabrata	2.7.10.1
Q8ITG6	Tyr	II	II	V	Biomphalaria glabrata	
Q9U5A8	Tyr	II	II	V	Bombyx mori	2.7.10.1
A6QR62	Tyr	II	II	V	Bos taurus	2.7.10.1
A7Z039	Tyr	II	II	V	Bos taurus	2.7.10.2
Q32PK0	Tyr	II	II	V	Bos taurus	2.7.10.2
Q3ZC95	Tyr	II	II	V	Bos taurus	2.7.10.2
Q58D16	Tyr	II	II	V	Bos taurus	2.7.10.2
Q5E9H3	Tyr	II	II	V	Bos taurus	2.7.10.2
Q17R13	Tyr	II	II	V	Bos taurus	2.7.10.2; 2.7.11.1
A6QR59	Tyr	II	II	V	Bos taurus	
O93597	Tyr	II	II	V	Brachydanio rerio	2.7.1.112
O73879	Tyr	II	II	V	Brachydanio rerio	2.7.10.1
Q9YI66	Tyr	II	II	V	Brachydanio rerio	2.7.10.1
O42200	Tyr	II	II	V	Brachydanio rerio	2.7.10.2
O73876	Tyr	II	II	V	Brachydanio rerio	
O73877	Tyr	II	II	V	Brachydanio rerio	
O73878	Tyr	II	II	V	Brachydanio rerio	
Q9U8V7	Tyr	II	II	V	Branchiostoma belcheri	
Q9U8V8	Tyr	II	II	V	Branchiostoma belcheri	
Q52V39	Tyr	II	II	V	Branchiostoma floridae	2.7.10.1
Q52V40	Tyr	II	II	V	Branchiostoma floridae	2.7.10.1
Q5I4E2	Tyr	II	II	V	Brugia malayi	
Q6TYB9	Tyr	II	II	V	Caenorhabditis briggsae	
Q38G54	Tyr	II	II	V	Caenorhabditis elegans	2.7.10.1
Q968Y9	Tyr	II	II	V	Caenorhabditis elegans	2.7.10.1
O01325	Tyr	II	II	V	Caenorhabditis elegans	2.7.10.2
O01798	Tyr	II	II	V	Caenorhabditis elegans	2.7.10.2
Q19726	Tyr	II	II	V	Caenorhabditis elegans	2.7.10.2
Q22146	Tyr	II	II	V	Caenorhabditis elegans	2.7.10.2

Table S2.3. (continuation)

UniProt	Label [1]	Two	Three	Seven	Species	EC Number
Q22243	Tyr	II	II	V	Caenorhabditis elegans	2.7.10.2
Q22765	Tyr	II	II	V	Caenorhabditis elegans	2.7.10.2
Q23102	Tyr	II	II	V	Caenorhabditis elegans	2.7.10.2
Q2EEM6	Tyr	II	II	V	Caenorhabditis elegans	2.7.10.2
Q8IAA6	Tyr	II	II	V	Caenorhabditis elegans	2.7.10.2
Q9N3S5	Tyr	II	II	V	Caenorhabditis elegans	2.7.10.2
Q9U3B5	Tyr	II	II	V	Caenorhabditis elegans	2.7.10.2
Q8T879	Tyr	II	II	V	Caenorhabditis elegans	
Q95YD4	Tyr	II	II	V	Caenorhabditis elegans	
Q95ZK1	Tyr	II	II	V	Caenorhabditis elegans	
Q9XVQ7	Tyr	II	II	V	Caenorhabditis elegans	
Q23821	Tyr	II	II	V	Caenorhabditis vulgaris	
A6MLH3	Tyr	II	II	V	Callithrix jacchus	
Q6DTW4	Tyr	II	II	V	Canis familiaris	
Q9IAA2	Tyr	II	II	V	Carassius auratus	2.7.10.1
Q07E11	Tyr	II	II	V	Cavia porcellus	
Q14UF1	Tyr	II	II	V	Cercopithecus aethiops	
Q4H3A3	Tyr	II	II	V	Ciona intestinalis	2.7.10.2
Q008W6	Tyr	II	II	V	Ciona intestinalis	
Q4H3M3	Tyr	II	II	V	Ciona intestinalis	
Q4H3M4	Tyr	II	II	V	Ciona intestinalis	
Q4H3M5	Tyr	II	II	V	Ciona intestinalis	
Q4H3M6	Tyr	II	II	V	Ciona intestinalis	
Q4H3M7	Tyr	II	II	V	Ciona intestinalis	
Q4H3N3	Tyr	II	II	V	Ciona intestinalis	
Q70W06	Tyr	II	II	V	Ciona intestinalis	
Q70W07	Tyr	II	II	V	Ciona intestinalis	
Q70W08	Tyr	II	II	V	Ciona intestinalis	
Q70W09	Tyr	II	II	V	Ciona intestinalis	
Q70W10	Tyr	II	II	V	Ciona intestinalis	
Q95YL0	Tyr	II	II	V	Ciona savignyi	
A6P6W2	Tyr	II	II	V	Coturnix coturnix	2.7.10.1
A6P6W3	Tyr	II	II	V	Coturnix coturnix	2.7.10.1
Q91356	Tyr	II	II	V	Coturnix coturnix	
Q7YT64	Tyr	II	II	V	Crassostrea gigas	2.7.10.1
Q5QJV8	Tyr	II	II	V	Culex tritaeniorhynchus	
A2IRM8	Tyr	II	II	V	Cynops pyrrhogaster	2.7.10.1
Q910A3	Tyr	II	II	V	Cyprinus carpio	2.7.10.2
Q9PTN6	Tyr	II	II	V	Cyprinus carpio	2.7.10.2
Q7T2P8	Tyr	II	II	V	Danio rerio	2.7.1.112
Q1LVG4	Tyr	II	II	V	Danio rerio	2.7.10.1
Q1LWJ4	Tyr	II	II	V	Danio rerio	2.7.10.1
Q5G254	Tyr	II	II	V	Danio rerio	2.7.10.1
Q8AW81	Tyr	II	II	V	Danio rerio	2.7.10.1
Q8JFV0	Tyr	II	II	V	Danio rerio	2.7.10.1
Q8JID1	Tyr	II	II	V	Danio rerio	2.7.10.1
Q8UUY9	Tyr	II	II	V	Danio rerio	2.7.10.1
A0JMN4	Tyr	II	II	V	Danio rerio	2.7.10.2
A2CES6	Tyr	II	II	V	Danio rerio	2.7.10.2
O93596	Tyr	II	II	V	Danio rerio	2.7.10.2
Q503P3	Tyr	II	II	V	Danio rerio	2.7.10.2
Q7ZU16	Tyr	II	II	V	Danio rerio	2.7.10.2
Q910A2	Tyr	II	II	V	Danio rerio	2.7.10.2
A0JMM7	Tyr	II	II	V	Danio rerio	
A2BFZ9	Tyr	II	II	V	Danio rerio	
A2BG00	Tyr	II	II	V	Danio rerio	

Table S2.3. (continuation)

UniProt	Label [1]	Two	Three	Seven	Species	EC Number
A2CEJ3	Tyr	II	II	V	Danio rerio	
Q1MT83	Tyr	II	II	V	Danio rerio	
Q4KMK2	Tyr	II	II	V	Danio rerio	
Q5EGE5	Tyr	II	II	V	Danio rerio	
Q5J1R8	Tyr	II	II	V	Danio rerio	
Q5J1R9	Tyr	II	II	V	Danio rerio	
Q5VSJ4	Tyr	II	II	V	Danio rerio	
Q5ZEW1	Tyr	II	II	V	Danio rerio	
Q64GK4	Tyr	II	II	V	Danio rerio	
Q6NZS1	Tyr	II	II	V	Danio rerio	
Q6W5B1	Tyr	II	II	V	Danio rerio	
Q7T2V4	Tyr	II	II	V	Danio rerio	
Q7ZZ92	Tyr	II	II	V	Danio rerio	
Q90ZN9	Tyr	II	II	V	Danio rerio	
Q98SN4	Tyr	II	II	V	Danio rerio	
Q2TGR3	Tyr	II	II	V	Drosophila biarmipes	
Q2TGR1	Tyr	II	II	V	Drosophila elegans	
Q2TGH9	Tyr	II	II	V	Drosophila ficusphila	
Q2TGI1	Tyr	II	II	V	Drosophila jambulina	
Q0KIF4	Tyr	II	II	V	Drosophila melanogaster	2.7.10.-; 2.7.10.1
Q59DQ4	Tyr	II	II	V	Drosophila melanogaster	2.7.10.-; 2.7.10.1
Q8IMC2	Tyr	II	II	V	Drosophila melanogaster	2.7.10.-; 2.7.10.1
Q9V4E5	Tyr	II	II	V	Drosophila melanogaster	2.7.10.-; 2.7.10.1
Q7KJ08	Tyr	II	II	V	Drosophila melanogaster	2.7.10.-; 2.7.10.1; 2.7.10.2
Q0E917	Tyr	II	II	V	Drosophila melanogaster	2.7.10.-; 2.7.10.2
Q9VZI2	Tyr	II	II	V	Drosophila melanogaster	2.7.10.-; 2.7.10.2
Q9U5Y2	Tyr	II	II	V	Drosophila melanogaster	2.7.10.1
Q6NP28	Tyr	II	II	V	Drosophila melanogaster	2.7.10.2
Q7KSQ2	Tyr	II	II	V	Drosophila melanogaster	2.7.10.2
Q9VGK8	Tyr	II	II	V	Drosophila melanogaster	2.7.10.2
O96435	Tyr	II	II	V	Drosophila melanogaster	
Q2TGI0	Tyr	II	II	V	Drosophila melanogaster	
Q5BIG9	Tyr	II	II	V	Drosophila melanogaster	
Q8SWV4	Tyr	II	II	V	Drosophila melanogaster	
Q9U472	Tyr	II	II	V	Drosophila melanogaster	
Q9U531	Tyr	II	II	V	Drosophila melanogaster	
Q9XZL6	Tyr	II	II	V	Drosophila melanogaster	
Q9Y1J0	Tyr	II	II	V	Drosophila melanogaster	
Q2TGI2	Tyr	II	II	V	Drosophila prostipennis	
Q29D86	Tyr	II	II	V	Drosophila pseudoobscura	2.7.10.-
Q293W0	Tyr	II	II	V	Drosophila pseudoobscura	2.7.10.1
Q291J2	Tyr	II	II	V	Drosophila pseudoobscura	2.7.10.2
Q296K9	Tyr	II	II	V	Drosophila pseudoobscura	2.7.10.2
Q29H22	Tyr	II	II	V	Drosophila pseudoobscura	2.7.10.2
Q2TGH8	Tyr	II	II	V	Drosophila pseudoobscura	
Q2TGR2	Tyr	II	II	V	Drosophila simulans	
Q2TGR0	Tyr	II	II	V	Drosophila takahashii	
Q9Y1X9	Tyr	II	II	V	Ephydatia fluviatilis	2.7.10.2
Q9Y1Y1	Tyr	II	II	V	Ephydatia fluviatilis	2.7.10.2
Q9Y1Y2	Tyr	II	II	V	Ephydatia fluviatilis	2.7.10.2
Q9Y1Z0	Tyr	II	II	V	Ephydatia fluviatilis	2.7.10.2
Q9Y1X8	Tyr	II	II	V	Ephydatia fluviatilis	
Q9Y1Y0	Tyr	II	II	V	Ephydatia fluviatilis	
Q9Y1Y3	Tyr	II	II	V	Ephydatia fluviatilis	
Q9Y1Y5	Tyr	II	II	V	Ephydatia fluviatilis	
Q9Y1Y8	Tyr	II	II	V	Ephydatia fluviatilis	

Table S2.3. (continuation)

UniProt	Label [1]	Two	Three	Seven	Species	EC Number
Q9Y1Y9	Tyr	II	II	V	Ephydatia fluviatilis	
Q5DIB7	Tyr	II	II	V	Epinephelus coioides	2.7.10.1
Q5DIB6	Tyr	II	II	V	Epinephelus coioides	
Q60I21	Tyr	II	II	V	Eptatretus burgeri	2.7.10.2
Q9U8V9	Tyr	II	II	V	Eptatretus burgeri	
Q9U8W0	Tyr	II	II	V	Eptatretus burgeri	
Q9U8W1	Tyr	II	II	V	Eptatretus burgeri	
Q9U8W2	Tyr	II	II	V	Eptatretus burgeri	
Q08JT2	Tyr	II	II	V	Eudromia elegans	
P79754	Tyr	II	II	V	Fugu rubripes	2.7.10.1
Q90YI6	Tyr	II	II	V	Fugu rubripes	2.7.10.2
Q8UW42	Tyr	II	II	V	Fugu rubripes	
Q9YGM5	Tyr	II	II	V	Fugu rubripes	
Q9YGM7	Tyr	II	II	V	Fugu rubripes	
Q9YGN0	Tyr	II	II	V	Fugu rubripes	
O92807	Tyr	II	II	V	Fujinami sarcoma	
Q90699	Tyr	II	II	V	Gallus gallus	2.7.10.1
Q98949	Tyr	II	II	V	Gallus gallus	2.7.10.1
Q9PWN6	Tyr	II	II	V	Gallus gallus	2.7.10.1
Q5ZI88	Tyr	II	II	V	Gallus gallus	2.7.10.2
Q5ZLF2	Tyr	II	II	V	Gallus gallus	2.7.10.2
Q75R65	Tyr	II	II	V	Gallus gallus	2.7.10.2
Q90778	Tyr	II	II	V	Gallus gallus	2.7.10.2
Q9PWM9	Tyr	II	II	V	Gallus gallus	2.7.10.2
A0M8T9	Tyr	II	II	V	Gallus gallus	
Q08757	Tyr	II	II	V	Gallus gallus	
Q5F3X2	Tyr	II	II	V	Gallus gallus	
Q705C2	Tyr	II	II	V	Gallus gallus	
Q90601	Tyr	II	II	V	Gallus gallus	
Q90777	Tyr	II	II	V	Gallus gallus	
Q90943	Tyr	II	II	V	Gallus gallus	
Q90975	Tyr	II	II	V	Gallus gallus	
Q98TD0	Tyr	II	II	V	Gallus gallus	
Q9PS75	Tyr	II	II	V	Gallus gallus	
O97191	Tyr	II	II	V	Geodia cydonium	2.7.10.1
O18433	Tyr	II	II	V	Geodia cydonium	
Q8T6I3	Tyr	II	II	V	Halocynthia roretzi	
A6P4T4	Tyr	II	II	V	Homo sapiens	2.7.10.1
A6P4V4	Tyr	II	II	V	Homo sapiens	2.7.10.1
Q4LE53	Tyr	II	II	V	Homo sapiens	2.7.10.1
Q59F17	Tyr	II	II	V	Homo sapiens	2.7.10.1
Q59FT4	Tyr	II	II	V	Homo sapiens	2.7.10.1
Q8TDJ5	Tyr	II	II	V	Homo sapiens	2.7.10.1
Q16176	Tyr	II	II	V	Homo sapiens	2.7.10.2
Q3MS96	Tyr	II	II	V	Homo sapiens	2.7.10.2
Q4LDX3	Tyr	II	II	V	Homo sapiens	2.7.10.2
Q506Q0	Tyr	II	II	V	Homo sapiens	2.7.10.2
Q53EL3	Tyr	II	II	V	Homo sapiens	2.7.10.2
Q59FK4	Tyr	II	II	V	Homo sapiens	2.7.10.2
Q8IXP2	Tyr	II	II	V	Homo sapiens	2.7.10.2
A1L4F5	Tyr	II	II	V	Homo sapiens	
A2VCQ3	Tyr	II	II	V	Homo sapiens	
Q12787	Tyr	II	II	V	Homo sapiens	
Q15300	Tyr	II	II	V	Homo sapiens	
Q15850	Tyr	II	II	V	Homo sapiens	
Q1RMG3	Tyr	II	II	V	Homo sapiens	

Table S2.3. (continuation)

UniProt	Label [1]	Two	Three	Seven	Species	EC Number
Q2VJ45	Tyr	II	II	V	Homo sapiens	
Q2VXS9	Tyr	II	II	V	Homo sapiens	
Q3MS92	Tyr	II	II	V	Homo sapiens	
Q4JFK8	Tyr	II	II	V	Homo sapiens	
Q53R53	Tyr	II	II	V	Homo sapiens	
Q53T57	Tyr	II	II	V	Homo sapiens	
Q53TA0	Tyr	II	II	V	Homo sapiens	
Q541P7	Tyr	II	II	V	Homo sapiens	
Q58F15	Tyr	II	II	V	Homo sapiens	
Q59EB3	Tyr	II	II	V	Homo sapiens	
Q59F19	Tyr	II	II	V	Homo sapiens	
Q59FM9	Tyr	II	II	V	Homo sapiens	
Q59FQ5	Tyr	II	II	V	Homo sapiens	
Q59FX1	Tyr	II	II	V	Homo sapiens	
Q59GM4	Tyr	II	II	V	Homo sapiens	
Q59GM6	Tyr	II	II	V	Homo sapiens	
Q59GN8	Tyr	II	II	V	Homo sapiens	
Q658W2	Tyr	II	II	V	Homo sapiens	
Q6P4R6	Tyr	II	II	V	Homo sapiens	
Q6ZNH1	Tyr	II	II	V	Homo sapiens	
Q6ZRA8	Tyr	II	II	V	Homo sapiens	
Q75MF2	Tyr	II	II	V	Homo sapiens	
Q8N6J3	Tyr	II	II	V	Homo sapiens	
Q8N9D7	Tyr	II	II	V	Homo sapiens	
Q8WTZ8	Tyr	II	II	V	Homo sapiens	
Q96HF4	Tyr	II	II	V	Homo sapiens	
Q96L35	Tyr	II	II	V	Homo sapiens	
Q9NPI2	Tyr	II	II	V	Homo sapiens	
Q9NPI3	Tyr	II	II	V	Homo sapiens	
Q9NSQ6	Tyr	II	II	V	Homo sapiens	
Q9P0L3	Tyr	II	II	V	Homo sapiens	
Q9P0L5	Tyr	II	II	V	Homo sapiens	
Q9P0L6	Tyr	II	II	V	Homo sapiens	
Q9P0L7	Tyr	II	II	V	Homo sapiens	
Q9P0M0	Tyr	II	II	V	Homo sapiens	
Q9UMQ4	Tyr	II	II	V	Homo sapiens	
O77132	Tyr	II	II	V	Hydra attenuata	2.7.10.2
O77440	Tyr	II	II	V	Hydra attenuata	2.7.10.2
O61731	Tyr	II	II	V	Hydra attenuata	
Q5MB01	Tyr	II	II	V	Hydra magnipapillata	
Q5MCM8	Tyr	II	II	V	Hydractinia echinata	
Q9PVV1	Tyr	II	II	V	Lampetra reissneri	
Q9PVV2	Tyr	II	II	V	Lampetra reissneri	
Q7YWD8	Tyr	II	II	V	Lymnaea stagnalis	
Q7Z1D3	Tyr	II	II	V	Lytechinus variegatus	
Q2PFX3	Tyr	II	II	V	Macaca fascicularis	2.7.10.1
Q95K57	Tyr	II	II	V	Macaca fascicularis	
Q1HKZ4	Tyr	II	II	V	Macaca mulatta	
Q6VU50	Tyr	II	II	V	Manduca sexta	
Q7YZH7	Tyr	II	II	V	Monosiga brevicollis	
Q8WRF4	Tyr	II	II	V	Monosiga brevicollis	
Q0VGZ5	Tyr	II	II	V	Mus musculus	2.7.10.1
Q3U1L4	Tyr	II	II	V	Mus musculus	2.7.10.1
Q3UVJ3	Tyr	II	II	V	Mus musculus	2.7.10.1
Q60669	Tyr	II	II	V	Mus musculus	2.7.10.1
Q6P5F1	Tyr	II	II	V	Mus musculus	2.7.10.1

Table S2.3. (continuation)

UniProt	Label [1]	Two	Three	Seven	Species	EC Number
A1A560	Tyr	II	II	V	Mus musculus	2.7.10.2
P70223	Tyr	II	II	V	Mus musculus	2.7.10.2
P97423	Tyr	II	II	V	Mus musculus	2.7.10.2
Q05AA8	Tyr	II	II	V	Mus musculus	2.7.10.2
Q0VBH4	Tyr	II	II	V	Mus musculus	2.7.10.2
Q0VBP6	Tyr	II	II	V	Mus musculus	2.7.10.2
Q2M4G5	Tyr	II	II	V	Mus musculus	2.7.10.2
Q3TDN4	Tyr	II	II	V	Mus musculus	2.7.10.2
Q3TH41	Tyr	II	II	V	Mus musculus	2.7.10.2
Q3TIS9	Tyr	II	II	V	Mus musculus	2.7.10.2
Q3TXE3	Tyr	II	II	V	Mus musculus	2.7.10.2
Q3U436	Tyr	II	II	V	Mus musculus	2.7.10.2
Q3U447	Tyr	II	II	V	Mus musculus	2.7.10.2
Q3U5G1	Tyr	II	II	V	Mus musculus	2.7.10.2
Q3U8P8	Tyr	II	II	V	Mus musculus	2.7.10.2
Q3UPF7	Tyr	II	II	V	Mus musculus	2.7.10.2
Q3UVH2	Tyr	II	II	V	Mus musculus	2.7.10.2
Q52KQ2	Tyr	II	II	V	Mus musculus	2.7.10.2
Q5STT8	Tyr	II	II	V	Mus musculus	2.7.10.2
Q6P1E0	Tyr	II	II	V	Mus musculus	2.7.10.2
Q7TMU1	Tyr	II	II	V	Mus musculus	2.7.10.2
Q8BQI4	Tyr	II	II	V	Mus musculus	2.7.10.2
Q8CAW3	Tyr	II	II	V	Mus musculus	2.7.10.2
Q8CFK4	Tyr	II	II	V	Mus musculus	2.7.10.2
Q9D6H7	Tyr	II	II	V	Mus musculus	2.7.10.2
A2ARN2	Tyr	II	II	V	Mus musculus	
A2BDQ4	Tyr	II	II	V	Mus musculus	
A4ZVL1	Tyr	II	II	V	Mus musculus	
Q32S49	Tyr	II	II	V	Mus musculus	
Q32S50	Tyr	II	II	V	Mus musculus	
Q32SD3	Tyr	II	II	V	Mus musculus	
Q32SD4	Tyr	II	II	V	Mus musculus	
Q3TI10	Tyr	II	II	V	Mus musculus	
Q3TJU8	Tyr	II	II	V	Mus musculus	
Q3TTM4	Tyr	II	II	V	Mus musculus	
Q3UDE9	Tyr	II	II	V	Mus musculus	
Q3V1K8	Tyr	II	II	V	Mus musculus	
Q497X0	Tyr	II	II	V	Mus musculus	
Q5DTX7	Tyr	II	II	V	Mus musculus	
Q5F2B4	Tyr	II	II	V	Mus musculus	
Q60515	Tyr	II	II	V	Mus musculus	
Q61055	Tyr	II	II	V	Mus musculus	
Q62121	Tyr	II	II	V	Mus musculus	
Q6PE80	Tyr	II	II	V	Mus musculus	
Q6PFV6	Tyr	II	II	V	Mus musculus	
Q8BIS9	Tyr	II	II	V	Mus musculus	
Q8BNP9	Tyr	II	II	V	Mus musculus	
Q8BRB1	Tyr	II	II	V	Mus musculus	
Q8C2G0	Tyr	II	II	V	Mus musculus	
Q8C3U1	Tyr	II	II	V	Mus musculus	
Q8C7S3	Tyr	II	II	V	Mus musculus	
Q8C8K1	Tyr	II	II	V	Mus musculus	
Q8CD05	Tyr	II	II	V	Mus musculus	
Q8CE52	Tyr	II	II	V	Mus musculus	
Q8K272	Tyr	II	II	V	Mus musculus	
Q8R381	Tyr	II	II	V	Mus musculus	

Table S2.3. (continuation)

UniProt	Label [1]	Two	Three	Seven	Species	EC Number
Q91YM0	Tyr	II	II	V	Mus musculus	
Q99KA8	Tyr	II	II	V	Mus musculus	
Q64103	Tyr	II	II	V	Mus sp	2.7.10.2
A7RLS3	Tyr	II	II	V	Nematostella vectensis	2.7.10.1
A1IKE0	Tyr	II	II	V	Nematostella vectensis	
A7RFY7	Tyr	II	II	V	Nematostella vectensis	
A7RGW8	Tyr	II	II	V	Nematostella vectensis	
A7RKA9	Tyr	II	II	V	Nematostella vectensis	
A7RL37	Tyr	II	II	V	Nematostella vectensis	
A7RL50	Tyr	II	II	V	Nematostella vectensis	
A7RN88	Tyr	II	II	V	Nematostella vectensis	
A7RN91	Tyr	II	II	V	Nematostella vectensis	
A7RNB1	Tyr	II	II	V	Nematostella vectensis	
A7RVU0	Tyr	II	II	V	Nematostella vectensis	
A7RZ69	Tyr	II	II	V	Nematostella vectensis	
A7S358	Tyr	II	II	V	Nematostella vectensis	
A7S5K8	Tyr	II	II	V	Nematostella vectensis	
A7S6K5	Tyr	II	II	V	Nematostella vectensis	
A7S933	Tyr	II	II	V	Nematostella vectensis	
A7SAX2	Tyr	II	II	V	Nematostella vectensis	
A7SEI8	Tyr	II	II	V	Nematostella vectensis	
A7SFD8	Tyr	II	II	V	Nematostella vectensis	
A7SL46	Tyr	II	II	V	Nematostella vectensis	
A7SLJ9	Tyr	II	II	V	Nematostella vectensis	
A7SQB4	Tyr	II	II	V	Nematostella vectensis	
A7SRD2	Tyr	II	II	V	Nematostella vectensis	
A7SRD3	Tyr	II	II	V	Nematostella vectensis	
A7SRD7	Tyr	II	II	V	Nematostella vectensis	
A7SYB5	Tyr	II	II	V	Nematostella vectensis	
A7SZN9	Tyr	II	II	V	Nematostella vectensis	
A7T4Y3	Tyr	II	II	V	Nematostella vectensis	
O73844	Tyr	II	II	V	Oncorhynchus mykiss	2.7.10.1
O73845	Tyr	II	II	V	Oncorhynchus mykiss	2.7.10.1
O73846	Tyr	II	II	V	Oncorhynchus mykiss	2.7.10.1
Q6VGS6	Tyr	II	II	V	Oryctolagus cuniculus	2.7.10.1
Q9XS54	Tyr	II	II	V	Oryctolagus cuniculus	
Q8MI23	Tyr	II	II	V	Ovis aries	
Q9N111	Tyr	II	II	V	Ovis aries	
Q9N112	Tyr	II	II	V	Ovis aries	
Q9N1B7	Tyr	II	II	V	Ovis aries	
Q4QXL2	Tyr	II	II	V	Oxyuranus scutellatus	
Q8UW83	Tyr	II	II	V	Paralichthys olivaceus	2.7.10.1
Q8UW84	Tyr	II	II	V	Paralichthys olivaceus	2.7.10.1
Q8UW85	Tyr	II	II	V	Paralichthys olivaceus	2.7.10.1
Q8UW86	Tyr	II	II	V	Paralichthys olivaceus	2.7.10.1
Q2TTN5	Tyr	II	II	V	Pimephales promelas	
Q5RCL1	Tyr	II	II	V	Pongo abelii	2.7.10.1
Q5RB23	Tyr	II	II	V	Pongo abelii	2.7.10.2
Q5R7F6	Tyr	II	II	V	Pongo abelii	
Q5RDU1	Tyr	II	II	V	Pongo abelii	
Q5RFB5	Tyr	II	II	V	Pongo abelii	
O42455	Tyr	II	II	V	Raja eglanteria	2.7.10.2
Q6Q2A2	Tyr	II	II	V	Rattus norvegicus	2.7.10.1
Q2PYT3	Tyr	II	II	V	Rattus norvegicus	2.7.10.2
Q2PYT4	Tyr	II	II	V	Rattus norvegicus	2.7.10.2
Q501W1	Tyr	II	II	V	Rattus norvegicus	2.7.10.2

Table S2.3. (continuation)

UniProt	Label [1]	Two	Three	Seven	Species	EC Number
Q5FVG7	Tyr	II	II	V	Rattus norvegicus	2.7.10.2
Q5S255	Tyr	II	II	V	Rattus norvegicus	2.7.10.2
Q63614	Tyr	II	II	V	Rattus norvegicus	2.7.10.2
Q9EQ78	Tyr	II	II	V	Rattus norvegicus	2.7.10.2
Q5U2X5	Tyr	II	II	V	Rattus norvegicus	2.7.10.2; 2.7.11.1
Q2IBC7	Tyr	II	II	V	Rattus norvegicus	
Q5FVF5	Tyr	II	II	V	Rattus norvegicus	
Q5FVN9	Tyr	II	II	V	Rattus norvegicus	
Q8VI99	Tyr	II	II	V	Rattus norvegicus	
Q8VIA0	Tyr	II	II	V	Rattus norvegicus	
Q5BXN9	Tyr	II	II	V	Schistosoma japonicum	
Q7YSY1	Tyr	II	II	V	Schistosoma mansoni	2.7.10.2
Q8WPV5	Tyr	II	II	V	Schistosoma mansoni	2.7.10.2
O93457	Tyr	II	II	V	Scophthalmus maximus	2.7.10.1
Q9YGH8	Tyr	II	II	V	Scophthalmus maximus	2.7.10.1
Q2LC18	Tyr	II	II	V	Strongylocentrotus purpuratus	2.7.10.1
Q26633	Tyr	II	II	V	Strongylocentrotus purpuratus	
Q08JU8	Tyr	II	II	V	Struthio camelus	
Q7YZX2	Tyr	II	II	V	Suberites domuncula	2.7.10.2
Q5K4G5	Tyr	II	II	V	Suberites domuncula	
O19064	Tyr	II	II	V	Sus scrofa	2.7.10.2
Q684M7	Tyr	II	II	V	Sus scrofa	2.7.10.2
Q9TTJ1	Tyr	II	II	V	Sus scrofa	2.7.10.2
O76978	Tyr	II	II	V	Sycon raphanus	2.7.10.2
Q6B515	Tyr	II	II	V	Taeniopygia guttata	2.7.10.1
O57612	Tyr	II	II	V	Tetraodon fluviatilis	2.7.10.2
Q6Y4Q0	Tyr	II	II	V	Tetraodon fluviatilis	2.7.10.2
Q9PVI2	Tyr	II	II	V	Tetraodon fluviatilis	2.7.10.2
Q9PWD0	Tyr	II	II	V	Tetraodon fluviatilis	2.7.10.2
Q9PWD1	Tyr	II	II	V	Tetraodon fluviatilis	2.7.10.2
Q4REV4	Tyr	II	II	V	Tetraodon nigroviridis	2.7.10.1
Q4RV12	Tyr	II	II	V	Tetraodon nigroviridis	2.7.10.1
Q4S8B8	Tyr	II	II	V	Tetraodon nigroviridis	2.7.10.1
Q4SEJ9	Tyr	II	II	V	Tetraodon nigroviridis	2.7.10.1
Q4SK07	Tyr	II	II	V	Tetraodon nigroviridis	2.7.10.1
Q4SMF1	Tyr	II	II	V	Tetraodon nigroviridis	2.7.10.1
Q4SP88	Tyr	II	II	V	Tetraodon nigroviridis	2.7.10.1
Q4RJ39	Tyr	II	II	V	Tetraodon nigroviridis	2.7.10.2
Q4RS96	Tyr	II	II	V	Tetraodon nigroviridis	2.7.10.2
Q4RTE9	Tyr	II	II	V	Tetraodon nigroviridis	2.7.10.2
Q4RU24	Tyr	II	II	V	Tetraodon nigroviridis	2.7.10.2
Q4RUC5	Tyr	II	II	V	Tetraodon nigroviridis	2.7.10.2
Q4RUE3	Tyr	II	II	V	Tetraodon nigroviridis	2.7.10.2
Q4S351	Tyr	II	II	V	Tetraodon nigroviridis	2.7.10.2
Q4S8A6	Tyr	II	II	V	Tetraodon nigroviridis	2.7.10.2
Q4SF48	Tyr	II	II	V	Tetraodon nigroviridis	2.7.10.2
Q4SFG7	Tyr	II	II	V	Tetraodon nigroviridis	2.7.10.2
Q4SII9	Tyr	II	II	V	Tetraodon nigroviridis	2.7.10.2
Q4SS18	Tyr	II	II	V	Tetraodon nigroviridis	2.7.10.2
Q4T1R9	Tyr	II	II	V	Tetraodon nigroviridis	2.7.10.2
Q4T9C6	Tyr	II	II	V	Tetraodon nigroviridis	2.7.10.2
Q4TEC2	Tyr	II	II	V	Tetraodon nigroviridis	2.7.10.2
Q4RH14	Tyr	II	II	V	Tetraodon nigroviridis	
Q4RHE1	Tyr	II	II	V	Tetraodon nigroviridis	
Q4RJ43	Tyr	II	II	V	Tetraodon nigroviridis	
Q4RJ80	Tyr	II	II	V	Tetraodon nigroviridis	

Table S2.3. (continuation)

UniProt	Label [1]	Two	Three	Seven	Species	EC Number
Q4RPK6	Tyr	II	II	V	Tetraodon nigroviridis	
Q4RQW0	Tyr	II	II	V	Tetraodon nigroviridis	
Q4RS82	Tyr	II	II	V	Tetraodon nigroviridis	
Q4RXA0	Tyr	II	II	V	Tetraodon nigroviridis	
Q4RXV8	Tyr	II	II	V	Tetraodon nigroviridis	
Q4RY69	Tyr	II	II	V	Tetraodon nigroviridis	
Q4RYX7	Tyr	II	II	V	Tetraodon nigroviridis	
Q4S1M5	Tyr	II	II	V	Tetraodon nigroviridis	
Q4SC90	Tyr	II	II	V	Tetraodon nigroviridis	
Q4SF69	Tyr	II	II	V	Tetraodon nigroviridis	
Q4SFJ9	Tyr	II	II	V	Tetraodon nigroviridis	
Q4SH73	Tyr	II	II	V	Tetraodon nigroviridis	
Q4SJ17	Tyr	II	II	V	Tetraodon nigroviridis	
Q4SMB8	Tyr	II	II	V	Tetraodon nigroviridis	
Q4SP07	Tyr	II	II	V	Tetraodon nigroviridis	
Q4SQX3	Tyr	II	II	V	Tetraodon nigroviridis	
Q4SWL0	Tyr	II	II	V	Tetraodon nigroviridis	
Q4T0T4	Tyr	II	II	V	Tetraodon nigroviridis	
Q4T272	Tyr	II	II	V	Tetraodon nigroviridis	
Q4TGU3	Tyr	II	II	V	Tetraodon nigroviridis	
Q4THV2	Tyr	II	II	V	Tetraodon nigroviridis	
Q07153	Tyr	II	II	V	Torpedo californica	
A5ADU7	Tyr	II	II	V	Vitis vinifera	
Q91373	Tyr	II	II	V	Xenopus	
A6H8K1	Tyr	II	II	V	Xenopus laevis	2.7.10.1
O57458	Tyr	II	II	V	Xenopus laevis	2.7.10.1
Q6KF80	Tyr	II	II	V	Xenopus laevis	2.7.10.1
Q6PA47	Tyr	II	II	V	Xenopus laevis	2.7.10.1
Q8QFP9	Tyr	II	II	V	Xenopus laevis	2.7.10.1
Q91776	Tyr	II	II	V	Xenopus laevis	2.7.10.1
Q9PST9	Tyr	II	II	V	Xenopus laevis	2.7.10.1
Q9YH43	Tyr	II	II	V	Xenopus laevis	2.7.10.1
Q9YH44	Tyr	II	II	V	Xenopus laevis	2.7.10.1
O73786	Tyr	II	II	V	Xenopus laevis	2.7.10.2
Q2TAS4	Tyr	II	II	V	Xenopus laevis	2.7.10.2
Q6DCV6	Tyr	II	II	V	Xenopus laevis	2.7.10.2
Q6DE64	Tyr	II	II	V	Xenopus laevis	2.7.10.2
Q6GNQ8	Tyr	II	II	V	Xenopus laevis	2.7.10.2
Q6GP63	Tyr	II	II	V	Xenopus laevis	2.7.10.2
Q6P6Z9	Tyr	II	II	V	Xenopus laevis	2.7.10.2
O42570	Tyr	II	II	V	Xenopus laevis	
P70003	Tyr	II	II	V	Xenopus laevis	
P79950	Tyr	II	II	V	Xenopus laevis	
Q32NP2	Tyr	II	II	V	Xenopus laevis	
Q5FWW9	Tyr	II	II	V	Xenopus laevis	
Q6DFG4	Tyr	II	II	V	Xenopus laevis	
Q6IR54	Tyr	II	II	V	Xenopus laevis	
Q6KF79	Tyr	II	II	V	Xenopus laevis	
Q6NRE9	Tyr	II	II	V	Xenopus laevis	
Q6NTV5	Tyr	II	II	V	Xenopus laevis	
Q6PA07	Tyr	II	II	V	Xenopus laevis	
Q7ZYM7	Tyr	II	II	V	Xenopus laevis	
Q91734	Tyr	II	II	V	Xenopus laevis	
Q9DDA2	Tyr	II	II	V	Xenopus laevis	
Q9DGL0	Tyr	II	II	V	Xenopus laevis	
Q9I9L1	Tyr	II	II	V	Xenopus laevis	

Table S2.3. (continuation)

UniProt	Label [1]	Two	Three	Seven	Species	EC Number
Q9PWR5	Tyr	II	II	V	Xenopus laevis	
Q9W650	Tyr	II	II	V	Xenopus laevis	
A0JM20	Tyr	II	II	V	Xenopus tropicalis	2.7.10.1
Q08D53	Tyr	II	II	V	Xenopus tropicalis	2.7.10.1
A0JM01	Tyr	II	II	V	Xenopus tropicalis	2.7.10.2
Q6DF54	Tyr	II	II	V	Xenopus tropicalis	2.7.10.2
A0JP83	Tyr	II	II	V	Xenopus tropicalis	
Q0P4R9	Tyr	II	II	V	Xenopus tropicalis	
Q28CP6	Tyr	II	II	V	Xenopus tropicalis	
Q5XH98	Tyr	II	II	V	Xenopus tropicalis	
Q6DII4	Tyr	II	II	V	Xenopus tropicalis	
Q788R0	Tyr	II	II	V	Xiphophorus maculatus	
Q788R1	Tyr	II	II	V	Xiphophorus maculatus	
Q99162	Tyr	II	II	V	Xiphophorus maculatus	
O42291	Tyr	II	III	V	Gallus gallus	2.7.10.2

Case study IV: serine proteases

Of the 1,533 proteins in this family, 43 are labeled as elastases, 25 as chymotrypsins, and 1,464 as trypsins, of which 13 are kallikreins, according to the labels employed in [1]. Table S2.4 lists the protein set, along with the corresponding subfamily labels and the clusters they were placed in by the proposed framework when considering four, eleven and twelve clusters, as well as the source organisms and existing EC number annotations according to UniProt.

Table S2.4. List of proteins in the serine protease family.

UniProt	Label [1]	Four	Eleven	Twelve	Species	EC Number
P00766	chymotrypsin	IV	X	I	Bos taurus	3.4.21.1
P04813	chymotrypsin	IV	X	I	Canis familiaris	3.4.21.1
A2VD37	chymotrypsin	IV	X	I	Danio rerio	
Q1JPZ1	chymotrypsin	IV	X	I	Danio rerio	
Q66HW9	chymotrypsin	IV	X	I	Danio rerio	
Q6AZC2	chymotrypsin	IV	X	I	Danio rerio	
P47796	chymotrypsin	IV	X	I	Gadus morhua	3.4.21.1
Q53FV9	chymotrypsin	IV	X	I	Homo sapiens	
Q8IUW0	chymotrypsin	IV	X	I	Homo sapiens	
P40313	chymotrypsin	IV	X	I	Homo sapiens	3.4.21.-
P17538	chymotrypsin	IV	X	I	Homo sapiens	3.4.21.1
Q9D7P8	chymotrypsin	IV	X	I	Mus musculus	
Q9D960	chymotrypsin	IV	X	I	Mus musculus	
Q9ER05	chymotrypsin	IV	X	I	Mus musculus	
Q9W7Q3	chymotrypsin	IV	X	I	Paralichthys olivaceus	
Q9W7Q4	chymotrypsin	IV	X	I	Paralichthys olivaceus	
Q9EQZ8	chymotrypsin	IV	X	I	Rattus norvegicus	
Q4QY77	chymotrypsin	IV	X	I	Sparus aurata	
Q0GYP6	chymotrypsin	IV	X	I	Sparus aurata	3.4.21.1
Q4QY78	chymotrypsin	IV	X	I	Sparus aurata	3.4.21.1
Q4RHR8	chymotrypsin	IV	X	I	Tetraodon nigroviridis	
Q6GNF7	chymotrypsin	IV	X	I	Xenopus laevis	
Q6PGS4	chymotrypsin	IV	X	I	Xenopus laevis	
Q7SYS4	chymotrypsin	IV	X	I	Xenopus laevis	
Q5HZD0	chymotrypsin	IV	X	I	Xenopus tropicalis	
Q5I029	chymotrypsin	IV	X	I	Xenopus tropicalis	
A7YWU4	elastase	I	III	III	Bos taurus	
P05805	elastase	I	III	III	Bos taurus	
Q95KW7	elastase	I	III	III	Bos taurus	
Q28153	elastase	I	III	III	Bos taurus	3.4.21.36

Table S2.4. (continuation)

UniProt	Label [1]	Four	Eleven	Twelve	Species	EC Number
Q29461	elastase	I	III	III	Bos taurus	3.4.21.71
Q867B0	elastase	I	III	III	Canis familiaris	3.4.21.36
Q4QRH5	elastase	I	III	III	Danio rerio	
Q6AZC0	elastase	I	III	III	Danio rerio	
Q803Z4	elastase	I	III	III	Danio rerio	
Q5R1M5	elastase	I	III	III	Felis catus	3.4.21.36
Q92077	elastase	I	III	III	Gadus morhua	3.4.21.37
Q6ICV2	elastase	I	III	III	Homo sapiens	
Q6ISN8	elastase	I	III	III	Homo sapiens	
Q6ISU5	elastase	I	III	III	Homo sapiens	
Q96QL8	elastase	I	III	III	Homo sapiens	
Q9UNI1	elastase	I	III	III	Homo sapiens	3.4.21.36
P08861	elastase	I	III	III	Homo sapiens	3.4.21.70
P09093	elastase	I	III	III	Homo sapiens	3.4.21.70
P08217	elastase	I	III	III	Homo sapiens	3.4.21.71
O46644	elastase	I	III	III	Macaca fascicularis	3.4.21.36
O19023	elastase	I	III	III	Macaca mulatta	3.4.21.70
Q91X79	elastase	I	III	III	Mus musculus	3.4.21.36
Q9CQ52	elastase	I	III	III	Mus musculus	3.4.21.70
P05208	elastase	I	III	III	Mus musculus	3.4.21.71
Q9W7P9	elastase	I	III	III	Paralichthys olivaceus	
Q9W7Q1	elastase	I	III	III	Paralichthys olivaceus	
P00773	elastase	I	III	III	Rattus norvegicus	3.4.21.36
P00774	elastase	I	III	III	Rattus norvegicus	3.4.21.71
Q7SIG3	elastase	I	III	III	Salmo salar	3.4.21.36
Q4QY80	elastase	I	III	III	Sparus aurata	
Q4QY81	elastase	I	III	III	Sparus aurata	
P00772	elastase	I	III	III	Sus scrofa	3.4.21.36
P08419	elastase	I	III	III	Sus scrofa	3.4.21.71
Q4RZ78	elastase	I	III	III	Tetraodon nigroviridis	
Q4S848	elastase	I	III	III	Tetraodon nigroviridis	
Q4FZQ4	elastase	I	III	III	Xenopus laevis	
Q6GNG0	elastase	I	III	III	Xenopus laevis	
Q6GPY5	elastase	I	III	III	Xenopus laevis	
Q8QGF6	elastase	I	III	III	Xenopus laevis	
Q562D3	elastase	I	III	III	Xenopus tropicalis	
Q5BKG0	elastase	I	III	III	Xenopus tropicalis	
Q5HZU5	elastase	I	III	III	Xenopus tropicalis	
Q5I025	elastase	I	III	III	Xenopus tropicalis	
Q3I1X8	kallikrein	II	IV	V	Cercopithecus cephus	
Q3I1X9	kallikrein	II	IV	V	Erythrocebus patas	
Q546G3	kallikrein	II	IV	V	Homo sapiens	
Q6LDS3	kallikrein	II	IV	V	Homo sapiens	
Q8NCW4	kallikrein	II	IV	V	Homo sapiens	
P07288	kallikrein	II	IV	V	Homo sapiens	3.4.21.77
Q6DT45	kallikrein	II	IV	V	Macaca fascicularis	3.4.21.77
Q3I1Y0	kallikrein	II	IV	V	Macaca mulatta	
P33619	kallikrein	II	IV	V	Macaca mulatta	3.4.21.77
Q3I1Y4	kallikrein	II	IV	V	Pan paniscus	
Q3I1Y5	kallikrein	II	IV	V	Pan troglodytes	
Q3I1X7	kallikrein	II	IV	V	Papio anubis	
Q3I1Y2	kallikrein	II	IV	V	Pongo pygmaeus	
Q5FBW1	trypsin	II	IV	V	Blarina brevicauda	3.4.21.-
Q5FBW2	trypsin	II	IV	V	Blarina brevicauda	3.4.21.-
Q76B45	trypsin	II	IV	V	Blarina brevicauda	3.4.21.-
Q6H320	trypsin	II	IV	V	Bos taurus	

Table S2.4. (continuation)

UniProt	Label [1]	Four	Eleven	Twelve	Species	EC Number
P09582	trypsin	II	IV	V	Canis familiaris	3.4.21.35
Q29474	trypsin	II	IV	V	Canis familiaris	3.4.21.35
Q3I210	trypsin	II	IV	V	Cercopithecus cephus	
Q6H322	trypsin	II	IV	V	Equus caballus	
Q3I211	trypsin	II	IV	V	Erythrocebus patas	
P20151	trypsin	II	IV	V	Homo sapiens	3.4.21.35
Q8C232	trypsin	II	IV	V	Mus musculus	
P00755	trypsin	II	IV	V	Mus musculus	3.4.21.35
P00756	trypsin	II	IV	V	Mus musculus	3.4.21.35
P15945	trypsin	II	IV	V	Mus musculus	3.4.21.35
P15946	trypsin	II	IV	V	Mus musculus	3.4.21.35
P15947	trypsin	II	IV	V	Mus musculus	3.4.21.35
P15949	trypsin	II	IV	V	Mus musculus	3.4.21.35
Q61754	trypsin	II	IV	V	Mus musculus	3.4.21.35
A5A2L9	trypsin	II	IV	V	Ovis aries	
Q3I217	trypsin	II	IV	V	Pan paniscus	
Q3I218	trypsin	II	IV	V	Pan troglodytes	
Q3I212	trypsin	II	IV	V	Papio anubis	
Q3I216	trypsin	II	IV	V	Pongo pygmaeus	
P32824	trypsin	II	IV	V	Praomys natalensis	3.4.21.35
Q63274	trypsin	II	IV	V	Rattus norvegicus	
Q63275	trypsin	II	IV	V	Rattus norvegicus	
Q6IE61	trypsin	II	IV	V	Rattus norvegicus	
P00758	trypsin	II	IV	V	Rattus norvegicus	3.4.21.35
P36373	trypsin	II	IV	V	Rattus norvegicus	3.4.21.35
P36374	trypsin	II	IV	V	Rattus norvegicus	3.4.21.35
P36375	trypsin	II	IV	V	Rattus norvegicus	3.4.21.35
Q9YGS1	trypsin	II	IX	VIII	Agkistrodon acutus	3.4.21.-
Q3T0A3	trypsin	II	IX	VIII	Bos taurus	3.4.21.46
A7MCG1	trypsin	II	IX	VIII	Danio rerio	
Q6DBS8	trypsin	II	IX	VIII	Danio rerio	
O34289	trypsin	II	IX	VIII	Salvelinus fontinalis	
Q177E4	trypsin	II	IX	XI	Aedes aegypti	
Q4VB17	trypsin	II	IX	XI	Homo sapiens	
Q96JE0	trypsin	II	IX	XI	Homo sapiens	
Q96JE1	trypsin	II	IX	XI	Homo sapiens	
Q96JE2	trypsin	II	IX	XI	Homo sapiens	
Q9Y5K2	trypsin	II	IX	XI	Homo sapiens	3.4.21.-
Q6IE12	trypsin	II	IX	XI	Rattus norvegicus	
Q2XXN1	trypsin	II	IX	XII	Varanus acanthurus	
Q2XXN0	trypsin	II	IX	XII	Varanus mitchelli	
Q90Z47	trypsin	II	V	VII	Agkistrodon acutus	
Q98TT5	trypsin	II	V	VII	Agkistrodon acutus	
Q9I961	trypsin	II	V	VII	Agkistrodon acutus	
Q9W7S1	trypsin	II	V	VII	Agkistrodon acutus	
Q9I8X0	trypsin	II	V	VII	Agkistrodon acutus	3.4.21.-
Q9I8X1	trypsin	II	V	VII	Agkistrodon acutus	3.4.21.-
Q9I8X2	trypsin	II	V	VII	Agkistrodon acutus	3.4.21.-
Q9PSN3	trypsin	II	V	VII	Agkistrodon bilineatus	3.4.21.-
O42207	trypsin	II	V	VII	Agkistrodon caliginosus	3.4.21.-
P09872	trypsin	II	V	VII	Agkistrodon contortrix	3.4.21.-
P82981	trypsin	II	V	VII	Agkistrodon contortrix	3.4.21.-
O73800	trypsin	II	V	VII	Agkistrodon halys	3.4.21.-
O93421	trypsin	II	V	VII	Agkistrodon halys	3.4.21.-
P81176	trypsin	II	V	VII	Agkistrodon halys	3.4.21.-
Q802F0	trypsin	II	V	VII	Agkistrodon halys	3.4.21.-

Table S2.4. (continuation)

UniProt	Label [1]	Four	Eleven	Twelve	Species	EC Number
Q9PT51	trypsin	II	V	VII	Agkistrodon halys	3.4.21.-
Q9PTL3	trypsin	II	V	VII	Agkistrodon halys	3.4.21.-
Q9YGI6	trypsin	II	V	VII	Agkistrodon halys	3.4.21.-
Q9YGJ2	trypsin	II	V	VII	Agkistrodon halys	3.4.21.-
Q9YGJ8	trypsin	II	V	VII	Agkistrodon halys	3.4.21.-
Q9YGJ9	trypsin	II	V	VII	Agkistrodon halys	3.4.21.-
P47797	trypsin	II	V	VII	Agkistrodon rhodostoma	3.4.21.74
Q8QG86	trypsin	II	V	VII	Bothrops insularis	3.4.21.-
O13069	trypsin	II	V	VII	Bothrops jararaca	3.4.21.-
Q5W959	trypsin	II	V	VII	Bothrops jararaca	3.4.21.-
Q5W960	trypsin	II	V	VII	Bothrops jararaca	3.4.21.-
Q9PTU8	trypsin	II	V	VII	Bothrops jararaca	3.4.21.-
Q8QHK2	trypsin	II	V	VII	Crotalus atrox	3.4.21.-
Q2QA04	trypsin	II	V	VII	Crotalus durissus	3.4.21.-
Q58G94	trypsin	II	V	VII	Crotalus durissus	3.4.21.-
A1E235	trypsin	II	V	VII	Deinagkistrodon acutus	
A1E236	trypsin	II	V	VII	Deinagkistrodon acutus	
A1E237	trypsin	II	V	VII	Deinagkistrodon acutus	
A1E239	trypsin	II	V	VII	Deinagkistrodon acutus	
A1E2S1	trypsin	II	V	VII	Deinagkistrodon acutus	
A1E2S2	trypsin	II	V	VII	Deinagkistrodon acutus	
A1E2S3	trypsin	II	V	VII	Deinagkistrodon acutus	
A1E2S4	trypsin	II	V	VII	Deinagkistrodon acutus	
Q5I2B5	trypsin	II	V	VII	Deinagkistrodon acutus	
Q7SZE1	trypsin	II	V	VII	Gloydius saxatilis	3.4.21.-
P0C5B4	trypsin	II	V	VII	Gloydius shedaoensis	3.4.21.-
Q7SZE2	trypsin	II	V	VII	Gloydius ussuriensis	3.4.21.-
Q8UUJ1	trypsin	II	V	VII	Gloydius ussuriensis	3.4.21.-
Q8UUJ2	trypsin	II	V	VII	Gloydius ussuriensis	3.4.21.-
Q8UVX1	trypsin	II	V	VII	Gloydius ussuriensis	3.4.21.-
Q27J47	trypsin	II	V	VII	Lachesis muta	3.4.21.-
Q072L7	trypsin	II	V	VII	Lachesis stenophrys	3.4.21.-
Q2XXM3	trypsin	II	V	VII	Philodryas olfersii	
Q09GK1	trypsin	II	V	VII	Philodryas olfersii	3.4.21.-
P84787	trypsin	II	V	VII	Protobothrops elegans	3.4.21.-
P84788	trypsin	II	V	VII	Protobothrops elegans	3.4.21.-
Q6IWF1	trypsin	II	V	VII	Rhinocerophis alternatus	3.4.21.-
A7LAC6	trypsin	II	V	VII	Trimeresurus albolabris	3.4.21.-
A7LAC7	trypsin	II	V	VII	Trimeresurus albolabris	3.4.21.-
O13058	trypsin	II	V	VII	Trimeresurus flavoviridis	3.4.21.-
P05620	trypsin	II	V	VII	Trimeresurus flavoviridis	3.4.21.74
O13059	trypsin	II	V	VII	Trimeresurus gramineus	3.4.21.-
O13062	trypsin	II	V	VII	Trimeresurus gramineus	3.4.21.-
O13063	trypsin	II	V	VII	Trimeresurus gramineus	3.4.21.-
Q9DF66	trypsin	II	V	VII	Trimeresurus jerdonii	3.4.21.-
Q9DF67	trypsin	II	V	VII	Trimeresurus jerdonii	3.4.21.-
Q91507	trypsin	II	V	VII	Trimeresurus mucrosquamatus	3.4.21.-
Q91508	trypsin	II	V	VII	Trimeresurus mucrosquamatus	3.4.21.-
Q91509	trypsin	II	V	VII	Trimeresurus mucrosquamatus	3.4.21.-
Q91510	trypsin	II	V	VII	Trimeresurus mucrosquamatus	3.4.21.-
Q91511	trypsin	II	V	VII	Trimeresurus mucrosquamatus	3.4.21.-
Q9DG83	trypsin	II	V	VII	Trimeresurus mucrosquamatus	3.4.21.-
Q9DG84	trypsin	II	V	VII	Trimeresurus mucrosquamatus	3.4.21.-
Q71QH8	trypsin	II	V	VII	Trimeresurus stejnegeri	
Q71QH6	trypsin	II	V	VII	Trimeresurus stejnegeri	3.4.21.-
Q71QH7	trypsin	II	V	VII	Trimeresurus stejnegeri	3.4.21.-

Table S2.4. (continuation)

UniProt	Label [1]	Four	Eleven	Twelve	Species	EC Number
Q71QI1	trypsin	II	V	VII	Trimeresurus stejnegeri	3.4.21.-
Q71QI3	trypsin	II	V	VII	Trimeresurus stejnegeri	3.4.21.-
Q71QI8	trypsin	II	V	VII	Trimeresurus stejnegeri	3.4.21.-
Q71QJ0	trypsin	II	V	VII	Trimeresurus stejnegeri	3.4.21.-
Q8AY78	trypsin	II	V	VII	Trimeresurus stejnegeri	3.4.21.-
Q8AY79	trypsin	II	V	VII	Trimeresurus stejnegeri	3.4.21.-
Q8AY80	trypsin	II	V	VII	Trimeresurus stejnegeri	3.4.21.-
Q8AY81	trypsin	II	V	VII	Trimeresurus stejnegeri	3.4.21.-
Q91516	trypsin	II	V	VII	Trimeresurus stejnegeri	3.4.21.-
Q8JH85	trypsin	II	V	VII	Vipera lebetina	3.4.21.-
Q71QH5	trypsin	II	V	VII	Viridovipera stejnegeri	3.4.21.-
Q71QI5	trypsin	II	V	VII	Viridovipera stejnegeri	3.4.21.-
Q71QI7	trypsin	II	V	VII	Viridovipera stejnegeri	3.4.21.-
Q71QJ1	trypsin	II	V	VII	Viridovipera stejnegeri	3.4.21.-
Q71QJ3	trypsin	II	V	VII	Viridovipera stejnegeri	3.4.21.-
A1E238	trypsin	II	V	VIII	Deinagkistrodon acutus	
Q5XG53	trypsin	II	VII	VII	Xenopus laevis	
Q5MCS0	trypsin	II	VIII	VII	Hydrophis hardwickii	3.4.21.-
Q2PQJ3	trypsin	II	VIII	VIII	Bothrops jararacussu	3.4.21.-
Q91VE3	trypsin	II	XI	V	Mus musculus	3.4.21.117
O60259	trypsin	III	IV	V	Homo sapiens	3.4.21.118
Q61955	trypsin	III	IV	V	Mus musculus	3.4.21.118
O88780	trypsin	III	IV	V	Rattus norvegicus	3.4.21.118
O01309	trypsin	III	VII	X	Botryllus schlosseri	
O01310	trypsin	III	VII	X	Botryllus schlosseri	
A7SGX2	trypsin	III	VII	X	Nematostella vectensis	
Q3V5L6	trypsin	III	VII	X	Polyandrocarpa misakiensis	
A7T3C0	trypsin	III	VII	XII	Nematostella vectensis	
Q9UBX7	trypsin	III	VIII	IX	Homo sapiens	3.4.21.-
Q9UKR3	trypsin	III	VIII	IX	Homo sapiens	3.4.21.-
Q8CGR6	trypsin	III	VIII	IX	Mus musculus	
Q9QYN3	trypsin	III	VIII	IX	Mus musculus	3.4.21.-
Q0VCZ4	trypsin	III	VIII	V	Bos taurus	
Q6ICR6	trypsin	III	VIII	V	Homo sapiens	
O43240	trypsin	III	VIII	V	Homo sapiens	3.4.21.-
Q16ZR2	trypsin	III	VIII	VIII	Aedes aegypti	
Q8QGW3	trypsin	III	VIII	X	Anguilla japonica	3.4.21.4
A5PJB4	trypsin	III	VIII	X	Bos taurus	
A6QQ95	trypsin	III	VIII	X	Bos taurus	
A7YWU9	trypsin	III	VIII	X	Bos taurus	
Q547S4	trypsin	III	VIII	X	Bos taurus	
P00760	trypsin	III	VIII	X	Bos taurus	3.4.21.4
Q29463	trypsin	III	VIII	X	Bos taurus	3.4.21.4
P06871	trypsin	III	VIII	X	Canis familiaris	3.4.21.4
P06872	trypsin	III	VIII	X	Canis familiaris	3.4.21.4
A0FGS8	trypsin	III	VIII	X	Canis lupus	
A3FEW6	trypsin	III	VIII	X	Cavia porcellus	
A3FEW7	trypsin	III	VIII	X	Cavia porcellus	
Q9XY52	trypsin	III	VIII	X	Ctenocephalides felis	
Q2YDQ2	trypsin	III	VIII	X	Danio rerio	
Q561Z7	trypsin	III	VIII	X	Danio rerio	
Q7SX90	trypsin	III	VIII	X	Danio rerio	
Q8AV83	trypsin	III	VIII	X	Danio rerio	
Q1AMP9	trypsin	III	VIII	X	Dissostichus mawsoni	
Q9W6J8	trypsin	III	VIII	X	Dissostichus mawsoni	
Q9W6J9	trypsin	III	VIII	X	Dissostichus mawsoni	

Table S2.4. (continuation)

UniProt	Label [1]	Four	Eleven	Twelve	Species	EC Number
Q788V0	trypsin	III	VIII	X	Dissostichus mawsoni	3.4.21.4
Q92046	trypsin	III	VIII	X	Dissostichus mawsoni	3.4.21.4
Q98TG9	trypsin	III	VIII	X	Engraulis japonicus	
Q98TH0	trypsin	III	VIII	X	Engraulis japonicus	
Q91515	trypsin	III	VIII	X	Fugu rubripes	
Q8JFQ7	trypsin	III	VIII	X	Gadus morhua	
P16049	trypsin	III	VIII	X	Gadus morhua	3.4.21.4
Q91041	trypsin	III	VIII	X	Gadus morhua	3.4.21.4
A2JDL7	trypsin	III	VIII	X	Gallus gallus	
Q90627	trypsin	III	VIII	X	Gallus gallus	3.4.21.4
Q90628	trypsin	III	VIII	X	Gallus gallus	3.4.21.4
Q90629	trypsin	III	VIII	X	Gallus gallus	3.4.21.4
Q1M2M8	trypsin	III	VIII	X	Glycyphagus domesticus	
A1A508	trypsin	III	VIII	X	Homo sapiens	
A6XGL3	trypsin	III	VIII	X	Homo sapiens	
A6XMV8	trypsin	III	VIII	X	Homo sapiens	
A6XMV9	trypsin	III	VIII	X	Homo sapiens	
Q2XQG6	trypsin	III	VIII	X	Homo sapiens	
Q3SY19	trypsin	III	VIII	X	Homo sapiens	
Q3SY20	trypsin	III	VIII	X	Homo sapiens	
Q53F68	trypsin	III	VIII	X	Homo sapiens	
Q5NV56	trypsin	III	VIII	X	Homo sapiens	
Q7Z5F3	trypsin	III	VIII	X	Homo sapiens	
Q7Z5F4	trypsin	III	VIII	X	Homo sapiens	
Q8N2U3	trypsin	III	VIII	X	Homo sapiens	
Q96RQ0	trypsin	III	VIII	X	Homo sapiens	
Q92876	trypsin	III	VIII	X	Homo sapiens	3.4.21.-
Q9H2R5	trypsin	III	VIII	X	Homo sapiens	3.4.21.-
Q9P0G3	trypsin	III	VIII	X	Homo sapiens	3.4.21.-
Q9UKQ9	trypsin	III	VIII	X	Homo sapiens	3.4.21.-
Q9Y337	trypsin	III	VIII	X	Homo sapiens	3.4.21.-
P07477	trypsin	III	VIII	X	Homo sapiens	3.4.21.4
P07478	trypsin	III	VIII	X	Homo sapiens	3.4.21.4
P35030	trypsin	III	VIII	X	Homo sapiens	3.4.21.4
Q8NHM4	trypsin	III	VIII	X	Homo sapiens	3.4.21.4
A7LD79	trypsin	III	VIII	X	Kareius bicoloratus	
Q4A4I3	trypsin	III	VIII	X	Lepeophtheirus salmonis	
Q4A4I5	trypsin	III	VIII	X	Lepeophtheirus salmonis	
Q8T4P1	trypsin	III	VIII	X	Lepeophtheirus salmonis	
Q8T4P2	trypsin	III	VIII	X	Lepeophtheirus salmonis	
Q8T4P3	trypsin	III	VIII	X	Lepeophtheirus salmonis	
Q8T4P4	trypsin	III	VIII	X	Lepeophtheirus salmonis	
Q8T4P5	trypsin	III	VIII	X	Lepeophtheirus salmonis	
Q8T4P6	trypsin	III	VIII	X	Lepeophtheirus salmonis	
Q8T4P7	trypsin	III	VIII	X	Lepeophtheirus salmonis	
Q4A4I4	trypsin	III	VIII	X	Lepeophtheirus salmonis	3.4.21.4
Q6QX62	trypsin	III	VIII	X	Lepeophtheirus salmonis	3.4.21.4
Q7YSS9	trypsin	III	VIII	X	Lepeophtheirus salmonis	3.4.21.4
Q7Z1D5	trypsin	III	VIII	X	Lepeophtheirus salmonis	3.4.21.4
Q7Z1D6	trypsin	III	VIII	X	Lepeophtheirus salmonis	3.4.21.4
Q1M2L7	trypsin	III	VIII	X	Lepidoglyphus destructor	
Q5H728	trypsin	III	VIII	X	Macaca mulatta	
Q5H729	trypsin	III	VIII	X	Macaca mulatta	
Q5H730	trypsin	III	VIII	X	Macaca mulatta	
Q5H731	trypsin	III	VIII	X	Macaca mulatta	
Q5H732	trypsin	III	VIII	X	Macaca mulatta	

Table S2.4. (continuation)

UniProt	Label [1]	Four	Eleven	Twelve	Species	EC Number
Q5H733	trypsin	III	VIII	X	Macaca mulatta	
Q5H734	trypsin	III	VIII	X	Macaca mulatta	
O88301	trypsin	III	VIII	X	Mus musculus	
Q32M27	trypsin	III	VIII	X	Mus musculus	
Q3B856	trypsin	III	VIII	X	Mus musculus	
Q3V2E0	trypsin	III	VIII	X	Mus musculus	
Q3V2G3	trypsin	III	VIII	X	Mus musculus	
Q4G0C2	trypsin	III	VIII	X	Mus musculus	
Q792Y6	trypsin	III	VIII	X	Mus musculus	
Q792Y8	trypsin	III	VIII	X	Mus musculus	
Q792Y9	trypsin	III	VIII	X	Mus musculus	
Q792Z0	trypsin	III	VIII	X	Mus musculus	
Q792Z1	trypsin	III	VIII	X	Mus musculus	
Q7M754	trypsin	III	VIII	X	Mus musculus	
Q7TT42	trypsin	III	VIII	X	Mus musculus	
Q8CGR4	trypsin	III	VIII	X	Mus musculus	
Q91Y82	trypsin	III	VIII	X	Mus musculus	
Q99M20	trypsin	III	VIII	X	Mus musculus	
Q9CPN7	trypsin	III	VIII	X	Mus musculus	
Q9CPN9	trypsin	III	VIII	X	Mus musculus	
Q9D140	trypsin	III	VIII	X	Mus musculus	
Q9D7Y7	trypsin	III	VIII	X	Mus musculus	
Q9DBQ8	trypsin	III	VIII	X	Mus musculus	
Q9QUK9	trypsin	III	VIII	X	Mus musculus	
Q9R0T7	trypsin	III	VIII	X	Mus musculus	
Q9Z1R9	trypsin	III	VIII	X	Mus musculus	
Q8CGR5	trypsin	III	VIII	X	Mus musculus	3.4.21.-
P07146	trypsin	III	VIII	X	Mus musculus	3.4.21.4
A4ZX98	trypsin	III	VIII	X	Myxocyprinus asiaticus	
A7S0M2	trypsin	III	VIII	X	Nematostella vectensis	
Q9W6K0	trypsin	III	VIII	X	Notothenia coriiceps	
Q8AV11	trypsin	III	VIII	X	Oncorhynchus keta	
Q5XUG4	trypsin	III	VIII	X	Oreochromis aureus	
Q6R670	trypsin	III	VIII	X	Oreochromis aureus	
Q5XUG5	trypsin	III	VIII	X	Oreochromis niloticus	
Q6R671	trypsin	III	VIII	X	Oreochromis niloticus	
A4UWM7	trypsin	III	VIII	X	Oryzias latipes	
Q7T1R8	trypsin	III	VIII	X	Pangasianodon hypophthalmus	
A7LD78	trypsin	III	VIII	X	Paralichthys olivaceus	
Q9W7Q5	trypsin	III	VIII	X	Paralichthys olivaceus	
Q9W7Q6	trypsin	III	VIII	X	Paralichthys olivaceus	
Q9W7Q7	trypsin	III	VIII	X	Paralichthys olivaceus	
Q92099	trypsin	III	VIII	X	Paranotothenia magellanica	3.4.21.4
O42158	trypsin	III	VIII	X	Petromyzon marinus	
O42159	trypsin	III	VIII	X	Petromyzon marinus	
O42160	trypsin	III	VIII	X	Petromyzon marinus	
O42608	trypsin	III	VIII	X	Petromyzon marinus	
P35034	trypsin	III	VIII	X	Pleuronectes platessa	3.4.21.4
O93265	trypsin	III	VIII	X	Pseudopleuronectes americanus	3.4.21.4
O93266	trypsin	III	VIII	X	Pseudopleuronectes americanus	3.4.21.4
Q5BN44	trypsin	III	VIII	X	Pyrocoelia rufa	
Q8IT49	trypsin	III	VIII	X	Pyrocoelia rufa	
O54854	trypsin	III	VIII	X	Rattus norvegicus	
Q6IE66	trypsin	III	VIII	X	Rattus norvegicus	
P00762	trypsin	III	VIII	X	Rattus norvegicus	3.4.21.4
P00763	trypsin	III	VIII	X	Rattus norvegicus	3.4.21.4

Table S2.4. (continuation)

UniProt	Label [1]	Four	Eleven	Twelve	Species	EC Number
P08426	trypsin	III	VIII	X	Rattus norvegicus	3.4.21.4
P12788	trypsin	III	VIII	X	Rattus norvegicus	3.4.21.4
P32821	trypsin	III	VIII	X	Rattus norvegicus	3.4.21.4
P32822	trypsin	III	VIII	X	Rattus norvegicus	3.4.21.4
Q7JIG6	trypsin	III	VIII	X	Saguinus oedipus	3.4.21.-
P35031	trypsin	III	VIII	X	Salmo salar	3.4.21.4
P35032	trypsin	III	VIII	X	Salmo salar	3.4.21.4
P35033	trypsin	III	VIII	X	Salmo salar	3.4.21.4
Q4ADV4	trypsin	III	VIII	X	Seriola quinqueradiata	
A7VMR4	trypsin	III	VIII	X	Solea senegalensis	
A7VMR5	trypsin	III	VIII	X	Solea senegalensis	
A7VMR6	trypsin	III	VIII	X	Solea senegalensis	
A7VMR7	trypsin	III	VIII	X	Solea senegalensis	
A7VMR8	trypsin	III	VIII	X	Solea senegalensis	
A7VMR9	trypsin	III	VIII	X	Solea senegalensis	
Q4QY73	trypsin	III	VIII	X	Sparus aurata	
Q4QY79	trypsin	III	VIII	X	Sparus aurata	
Q0GYP4	trypsin	III	VIII	X	Sparus aurata	3.4.21.4
P00764	trypsin	III	VIII	X	Squalus acanthias	3.4.21.4
Q6GYJ5	trypsin	III	VIII	X	Struthio camelus	
P00761	trypsin	III	VIII	X	Sus scrofa	3.4.21.4
Q66PG8	trypsin	III	VIII	X	Takifugu rubripes	
Q66PG9	trypsin	III	VIII	X	Takifugu rubripes	
Q6RI79	trypsin	III	VIII	X	Tautogolabrus adspersus	3.4.21.4
Q4SH18	trypsin	III	VIII	X	Tetraodon nigroviridis	
Q4SH19	trypsin	III	VIII	X	Tetraodon nigroviridis	
Q4T8C0	trypsin	III	VIII	X	Tetraodon nigroviridis	
Q8WZM5	trypsin	III	VIII	X	Trichoderma harzianum	
Q05AV3	trypsin	III	VIII	X	Xenopus laevis	
Q3B898	trypsin	III	VIII	X	Xenopus laevis	
Q3KQ12	trypsin	III	VIII	X	Xenopus laevis	
Q4QR60	trypsin	III	VIII	X	Xenopus laevis	
Q66L05	trypsin	III	VIII	X	Xenopus laevis	
Q6GNU2	trypsin	III	VIII	X	Xenopus laevis	
Q6GPX7	trypsin	III	VIII	X	Xenopus laevis	
Q7SZ06	trypsin	III	VIII	X	Xenopus laevis	
Q7SZT1	trypsin	III	VIII	X	Xenopus laevis	
P19799	trypsin	III	VIII	X	Xenopus laevis	3.4.21.4
P70059	trypsin	III	VIII	X	Xenopus laevis	3.4.21.4
Q5EBE2	trypsin	III	VIII	X	Xenopus tropicalis	
Q5I0Q6	trypsin	III	VIII	X	Xenopus tropicalis	
Q5M8T8	trypsin	III	VIII	X	Xenopus tropicalis	
Q5M902	trypsin	III	VIII	X	Xenopus tropicalis	
Q5M910	trypsin	III	VIII	X	Xenopus tropicalis	
Q5M959	trypsin	III	VIII	X	Xenopus tropicalis	
Q5M976	trypsin	III	VIII	X	Xenopus tropicalis	
Q6DIW2	trypsin	III	VIII	X	Xenopus tropicalis	
Q5TNT2	trypsin	III	VIII	XI	Anopheles gambiae	
Q6QX60	trypsin	III	X	X	Lepeophtheirus salmonis	3.4.21.4
Q6QX61	trypsin	III	X	X	Lepeophtheirus salmonis	3.4.21.4
Q16TM1	trypsin	III	X	XI	Aedes aegypti	
P29787	trypsin	III	X	XI	Aedes aegypti	3.4.21.4
Q08LX6	trypsin	III	XI	IX	Patiria pectinifera	
Q9UKR0	trypsin	III	XI	X	Homo sapiens	3.4.21.-
Q9XYX9	trypsin	III	XI	X	Rhyzopertha dominica	
A7UNU1	trypsin	III	XI	XII	Aleuroglyphus ovatus	

Table S2.4. (continuation)

UniProt	Label [1]	Four	Eleven	Twelve	Species	EC Number
Q5XIZ0	trypsin	III	XI	XII	Danio rerio	
Q9CV76	trypsin	III	XI	XII	Mus musculus	
Q90244	trypsin	IV	I	IV	Acipenser transmontanus	
P00735	trypsin	IV	I	IV	Bos taurus	3.4.21.5
Q90387	trypsin	IV	I	IV	Cynops pyrrhogaster	
Q7SXH8	trypsin	IV	I	IV	Danio rerio	
Q90504	trypsin	IV	I	IV	Eptatretus stoutii	
Q804W7	trypsin	IV	I	IV	Fugu rubripes	3.4.21.5
Q91001	trypsin	IV	I	IV	Gallus gallus	3.4.21.5
Q91004	trypsin	IV	I	IV	Gecko gecko	
Q69EZ8	trypsin	IV	I	IV	Homo sapiens	
P00734	trypsin	IV	I	IV	Homo sapiens	3.4.21.5
A0N064	trypsin	IV	I	IV	Macaca mulatta	3.4.21.5
Q542C2	trypsin	IV	I	IV	Mus musculus	
P70375	trypsin	IV	I	IV	Mus musculus	3.4.21.21
P19221	trypsin	IV	I	IV	Mus musculus	3.4.21.5
Q3TJ94	trypsin	IV	I	IV	Mus musculus	3.4.21.5
Q91218	trypsin	IV	I	IV	Oncorhynchus mykiss	
Q5NKF9	trypsin	IV	I	IV	Oncorhynchus mykiss	3.4.21.5
Q28731	trypsin	IV	I	IV	Oryctolagus cuniculus	
Q5R537	trypsin	IV	I	IV	Pongo abelii	3.4.21.5
P18292	trypsin	IV	I	IV	Rattus norvegicus	3.4.21.5
Q9PTW7	trypsin	IV	I	IV	Struthio camelus	3.4.21.5
Q19AZ8	trypsin	IV	I	IV	Sus scrofa	3.4.21.5
Q4SUA7	trypsin	IV	I	IV	Tetraodon nigroviridis	
Q6GNK4	trypsin	IV	I	IV	Xenopus laevis	
Q4QR53	trypsin	IV	I	IV	Xenopus laevis	3.4.21.5
Q6DFJ5	trypsin	IV	I	IV	Xenopus laevis	3.4.21.5
Q5FVW1	trypsin	IV	I	IV	Xenopus tropicalis	3.4.21.5
Q9TXE6	trypsin	IV	II	II	Bombyx mori	
P35042	trypsin	IV	II	II	Choristoneura fumiferana	3.4.21.4
Q27540	trypsin	IV	II	II	Choristoneura fumiferana	3.4.21.4
Q961Y0	trypsin	IV	II	II	Galleria mellonella	
O18434	trypsin	IV	II	II	Helicoverpa armigera	
O18435	trypsin	IV	II	II	Helicoverpa armigera	
O18436	trypsin	IV	II	II	Helicoverpa armigera	
O18440	trypsin	IV	II	II	Helicoverpa armigera	
O18441	trypsin	IV	II	II	Helicoverpa armigera	
O18442	trypsin	IV	II	II	Helicoverpa armigera	
O18447	trypsin	IV	II	II	Helicoverpa armigera	
Q9NGY5	trypsin	IV	II	II	Heliothis virescens	
O76954	trypsin	IV	II	II	Lacanobia oleracea	
P35045	trypsin	IV	II	II	Manduca sexta	3.4.21.4
P35046	trypsin	IV	II	II	Manduca sexta	3.4.21.4
P35047	trypsin	IV	II	II	Manduca sexta	3.4.21.4
Q2I0D8	trypsin	IV	II	II	Ostrinia furnacalis	
Q56IB4	trypsin	IV	II	II	Ostrinia nubilalis	
Q56IB5	trypsin	IV	II	II	Ostrinia nubilalis	
Q56IB6	trypsin	IV	II	II	Ostrinia nubilalis	
Q56IB7	trypsin	IV	II	II	Ostrinia nubilalis	
Q56IB8	trypsin	IV	II	II	Ostrinia nubilalis	
Q6R561	trypsin	IV	II	II	Ostrinia nubilalis	
O62598	trypsin	IV	II	II	Plodia interpunctella	
Q9U4I6	trypsin	IV	II	II	Plodia interpunctella	
Q9U4I7	trypsin	IV	II	II	Plodia interpunctella	
Q4L1K4	trypsin	IV	II	II	Sesamia nonagrioides	3.4.21.4

Table S2.4. (continuation)

UniProt	Label [1]	Four	Eleven	Twelve	Species	EC Number
Q4L1K6	trypsin	IV	II	II	Sesamia nonagrioides	3.4.21.4
Q4L1K8	trypsin	IV	II	II	Sesamia nonagrioides	3.4.21.4
Q4L1L8	trypsin	IV	II	II	Sesamia nonagrioides	3.4.21.4
Q4L1L9	trypsin	IV	II	II	Sesamia nonagrioides	3.4.21.4
Q4L1M0	trypsin	IV	II	II	Sesamia nonagrioides	3.4.21.4
Q6R559	trypsin	IV	II	XI	Ostrinia nubilalis	
A8DZF9	trypsin	IV	IX	IX	Danio rerio	
Q0C799	trypsin	IV	IX	VIII	Aedes aegypti	
Q0C7A5	trypsin	IV	IX	VIII	Aedes aegypti	
Q0IEV1	trypsin	IV	IX	VIII	Aedes aegypti	
Q16EL5	trypsin	IV	IX	VIII	Aedes aegypti	
Q16H67	trypsin	IV	IX	VIII	Aedes aegypti	
Q16IK2	trypsin	IV	IX	VIII	Aedes aegypti	
Q16IK3	trypsin	IV	IX	VIII	Aedes aegypti	
Q16NE9	trypsin	IV	IX	VIII	Aedes aegypti	
Q16YZ2	trypsin	IV	IX	VIII	Aedes aegypti	
Q16ZR4	trypsin	IV	IX	VIII	Aedes aegypti	
Q170T9	trypsin	IV	IX	VIII	Aedes aegypti	
Q171W3	trypsin	IV	IX	VIII	Aedes aegypti	
Q175S3	trypsin	IV	IX	VIII	Aedes aegypti	
Q175S4	trypsin	IV	IX	VIII	Aedes aegypti	
Q7QCX2	trypsin	IV	IX	VIII	Anopheles gambiae	
A7UNZ4	trypsin	IV	IX	VIII	Bombyx mandarina	
A2TGR7	trypsin	IV	IX	VIII	Bombyx mori	
A7TVD3	trypsin	IV	IX	VIII	Bombyx mori	
Q7YRZ7	trypsin	IV	IX	VIII	Bos taurus	3.4.21.78
Q484F0	trypsin	IV	IX	VIII	Colwellia psychrerythraea	3.4.21.-
Q502L4	trypsin	IV	IX	VIII	Danio rerio	
O76920	trypsin	IV	IX	VIII	Drosophila melanogaster	
Q4V4I7	trypsin	IV	IX	VIII	Drosophila melanogaster	
Q4V4P3	trypsin	IV	IX	VIII	Drosophila melanogaster	
Q9VVT3	trypsin	IV	IX	VIII	Drosophila melanogaster	
A1Z7M7	trypsin	IV	IX	VIII	Drosophila melanogaster	3.4.21.-
Q7JZK6	trypsin	IV	IX	VIII	Drosophila melanogaster	3.4.21.-
Q8MRL2	trypsin	IV	IX	VIII	Drosophila melanogaster	3.4.21.-
Q9VDV1	trypsin	IV	IX	VIII	Drosophila melanogaster	3.4.21.-
Q28ZD1	trypsin	IV	IX	VIII	Drosophila pseudoobscura	3.4.21.-
Q298Z7	trypsin	IV	IX	VIII	Drosophila pseudoobscura	3.4.21.-
Q2M0D7	trypsin	IV	IX	VIII	Drosophila pseudoobscura	3.4.21.-
Q2S742	trypsin	IV	IX	VIII	Hahella chejuensis	
Q8I6K0	trypsin	IV	IX	VIII	Holotrichia diomphalia	
A6XNE2	trypsin	IV	IX	VIII	Homo sapiens	
Q6FHW3	trypsin	IV	IX	VIII	Homo sapiens	
P49863	trypsin	IV	IX	VIII	Homo sapiens	3.4.21.-
P00746	trypsin	IV	IX	VIII	Homo sapiens	3.4.21.46
Q5S1X0	trypsin	IV	IX	VIII	Ixodes scapularis	
A4FUV8	trypsin	IV	IX	VIII	Mus musculus	
Q9R0K0	trypsin	IV	IX	VIII	Mus musculus	
O35205	trypsin	IV	IX	VIII	Mus musculus	3.4.21.-
Q2Z1R4	trypsin	IV	IX	VIII	Oryzias latipes	
Q8CJF4	trypsin	IV	IX	VIII	Rattus norvegicus	
A5UZS7	trypsin	IV	IX	VIII	Roseiflexus sp	
P51779	trypsin	IV	IX	VIII	Sus scrofa	3.4.21.46
Q4STJ9	trypsin	IV	IX	VIII	Tetraodon nigroviridis	
Q63ZK0	trypsin	IV	IX	VIII	Xenopus laevis	3.4.21.-
Q5PPM3	trypsin	IV	IX	VIII	Xenopus tropicalis	

Table S2.4. (continuation)

UniProt	Label [1]	Four	Eleven	Twelve	Species	EC Number
Q6P326	trypsin	IV	IX	VIII	Xenopus tropicalis	3.4.21.-
Q0GC72	trypsin	IV	IX	X	Carassius auratus	
Q16GK0	trypsin	IV	IX	XI	Aedes aegypti	
Q16H66	trypsin	IV	IX	XI	Aedes aegypti	
Q16H68	trypsin	IV	IX	XI	Aedes aegypti	
Q16L19	trypsin	IV	IX	XI	Aedes aegypti	
Q16TD7	trypsin	IV	IX	XI	Aedes aegypti	
Q175C8	trypsin	IV	IX	XI	Aedes aegypti	
Q17B40	trypsin	IV	IX	XI	Aedes aegypti	
Q17EX4	trypsin	IV	IX	XI	Aedes aegypti	
Q17GI5	trypsin	IV	IX	XI	Aedes aegypti	
Q17J63	trypsin	IV	IX	XI	Aedes aegypti	
Q17J64	trypsin	IV	IX	XI	Aedes aegypti	
Q17LU0	trypsin	IV	IX	XI	Aedes aegypti	
Q1DGG8	trypsin	IV	IX	XI	Aedes aegypti	
Q1HRE9	trypsin	IV	IX	XI	Aedes aegypti	
Q1HRH0	trypsin	IV	IX	XI	Aedes aegypti	
Q9TXD8	trypsin	IV	IX	XI	Agelenopsis aperta	5.-.-.-
A4K8J0	trypsin	IV	IX	XI	Anopheles dirus	
A4K8J1	trypsin	IV	IX	XI	Anopheles dirus	
A0NG87	trypsin	IV	IX	XI	Anopheles gambiae	
O17489	trypsin	IV	IX	XI	Anopheles gambiae	
Q5TNA8	trypsin	IV	IX	XI	Anopheles gambiae	
Q5TQD6	trypsin	IV	IX	XI	Anopheles gambiae	
Q7PEV7	trypsin	IV	IX	XI	Anopheles gambiae	
Q7PQR9	trypsin	IV	IX	XI	Anopheles gambiae	
Q7PVP7	trypsin	IV	IX	XI	Anopheles gambiae	
Q7PVQ3	trypsin	IV	IX	XI	Anopheles gambiae	
Q7PWE5	trypsin	IV	IX	XI	Anopheles gambiae	
Q7PWT2	trypsin	IV	IX	XI	Anopheles gambiae	
Q7PX73	trypsin	IV	IX	XI	Anopheles gambiae	
Q7PXJ5	trypsin	IV	IX	XI	Anopheles gambiae	
Q7Q483	trypsin	IV	IX	XI	Anopheles gambiae	
Q7Q619	trypsin	IV	IX	XI	Anopheles gambiae	
Q7QCV0	trypsin	IV	IX	XI	Anopheles gambiae	
Q9NAS9	trypsin	IV	IX	XI	Anopheles gambiae	
Q9NFU1	trypsin	IV	IX	XI	Anopheles gambiae	
Q9Y1K7	trypsin	IV	IX	XI	Anopheles gambiae	
Q2VG86	trypsin	IV	IX	XI	Bombyx mori	
Q8I924	trypsin	IV	IX	XI	Bombyx mori	
Q8MP08	trypsin	IV	IX	XI	Bombyx mori	
A5PK33	trypsin	IV	IX	XI	Bos taurus	
Q8QGV1	trypsin	IV	IX	XI	Cyprinus carpio	
Q6DHH4	trypsin	IV	IX	XI	Danio rerio	
Q8I7W8	trypsin	IV	IX	XI	Dermacentor andersoni	
Q8I862	trypsin	IV	IX	XI	Dermacentor variabilis	
O76498	trypsin	IV	IX	XI	Diaprepes abbreviatus	
A0JQ30	trypsin	IV	IX	XI	Drosophila melanogaster	
A7DZ29	trypsin	IV	IX	XI	Drosophila melanogaster	
Q4V3F4	trypsin	IV	IX	XI	Drosophila melanogaster	
Q4V3K5	trypsin	IV	IX	XI	Drosophila melanogaster	
Q4V3X9	trypsin	IV	IX	XI	Drosophila melanogaster	
Q8MR95	trypsin	IV	IX	XI	Drosophila melanogaster	
Q8SXG6	trypsin	IV	IX	XI	Drosophila melanogaster	
Q9V3Z2	trypsin	IV	IX	XI	Drosophila melanogaster	3.4.16.-; 3.4.21.-
Q9VQ97	trypsin	IV	IX	XI	Drosophila melanogaster	3.4.16.-; 3.4.21.-

Table S2.4. (continuation)

UniProt	Label [1]	Four	Eleven	Twelve	Species	EC Number
A1Z7D1	trypsin	IV	IX	XI	Drosophila melanogaster	3.4.21.-
P05049	trypsin	IV	IX	XI	Drosophila melanogaster	3.4.21.-
Q86PB3	trypsin	IV	IX	XI	Drosophila melanogaster	3.4.21.-
Q8IRK5	trypsin	IV	IX	XI	Drosophila melanogaster	3.4.21.-
Q8SY35	trypsin	IV	IX	XI	Drosophila melanogaster	3.4.21.-
Q8SYS8	trypsin	IV	IX	XI	Drosophila melanogaster	3.4.21.-
Q9VA44	trypsin	IV	IX	XI	Drosophila melanogaster	3.4.21.-
Q9VB68	trypsin	IV	IX	XI	Drosophila melanogaster	3.4.21.-
Q9VDU8	trypsin	IV	IX	XI	Drosophila melanogaster	3.4.21.-
Q9VS86	trypsin	IV	IX	XI	Drosophila melanogaster	3.4.21.-
Q9VS87	trypsin	IV	IX	XI	Drosophila melanogaster	3.4.21.-
Q9VZH5	trypsin	IV	IX	XI	Drosophila melanogaster	3.4.21.-
Q28X41	trypsin	IV	IX	XI	Drosophila pseudoobscura	3.4.21.-
Q293L7	trypsin	IV	IX	XI	Drosophila pseudoobscura	3.4.21.-
Q294N1	trypsin	IV	IX	XI	Drosophila pseudoobscura	3.4.21.-
Q299R1	trypsin	IV	IX	XI	Drosophila pseudoobscura	3.4.21.-
Q29AC5	trypsin	IV	IX	XI	Drosophila pseudoobscura	3.4.21.-
Q29C29	trypsin	IV	IX	XI	Drosophila pseudoobscura	3.4.21.-
Q29D81	trypsin	IV	IX	XI	Drosophila pseudoobscura	3.4.21.-
Q2M0F8	trypsin	IV	IX	XI	Drosophila pseudoobscura	3.4.21.-
Q2M121	trypsin	IV	IX	XI	Drosophila pseudoobscura	3.4.21.-
Q2M122	trypsin	IV	IX	XI	Drosophila pseudoobscura	3.4.21.-
Q7SZC3	trypsin	IV	IX	XI	Gallus gallus	
O97366	trypsin	IV	IX	XI	Holotrichia diomphalia	
P12544	trypsin	IV	IX	XI	Homo sapiens	3.4.21.78
Q8I925	trypsin	IV	IX	XI	Hyphantria cunea	
Q5MGE4	trypsin	IV	IX	XI	Lonomia obliqua	
O44332	trypsin	IV	IX	XI	Manduca sexta	
O77102	trypsin	IV	IX	XI	Manduca sexta	
Q5DI99	trypsin	IV	IX	XI	Manduca sexta	
Q5MPB7	trypsin	IV	IX	XI	Manduca sexta	
Q5MPB8	trypsin	IV	IX	XI	Manduca sexta	
Q5MPC4	trypsin	IV	IX	XI	Manduca sexta	
Q5MPC6	trypsin	IV	IX	XI	Manduca sexta	
Q7M4I3	trypsin	IV	IX	XI	Megabombus pennsylvanicus	3.4.21.-
Q3U0N0	trypsin	IV	IX	XI	Mus musculus	
Q3UK79	trypsin	IV	IX	XI	Mus musculus	
Q9JIS2	trypsin	IV	IX	XI	Mus musculus	
Q9Z0M1	trypsin	IV	IX	XI	Mus musculus	
P11032	trypsin	IV	IX	XI	Mus musculus	3.4.21.78
Q5W1K5	trypsin	IV	IX	XI	Nilaparvata lugens	
Q6W741	trypsin	IV	IX	XI	Pediculus humanus	
Q6BDA8	trypsin	IV	IX	XI	Penaeus japonicus	
A0JCK6	trypsin	IV	IX	XI	Plutella xylostella	
Q7Z269	trypsin	IV	IX	XI	Polistes dominula	3.4.21.-
P49864	trypsin	IV	IX	XI	Rattus norvegicus	3.4.21.-
A3SQQ6	trypsin	IV	IX	XI	Roseovarius nubinhibens	
O96442	trypsin	IV	IX	XI	Strongylocentrotus purpuratus	
Q9XSN6	trypsin	IV	IX	XI	Sus scrofa	
P91817	trypsin	IV	IX	XI	Tachypleus tridentatus	
Q27083	trypsin	IV	IX	XI	Tachypleus tridentatus	
Q27081	trypsin	IV	IX	XI	Tachypleus tridentatus	3.4.21.85
P21902	trypsin	IV	IX	XI	Tachypleus tridentatus	3.4.21.86
Q4RVJ6	trypsin	IV	IX	XI	Tetraodon nigroviridis	
Q4SQ11	trypsin	IV	IX	XI	Tetraodon nigroviridis	
O96871	trypsin	IV	IX	XI	Trichinella spiralis	

Table S2.4. (continuation)

UniProt	Label [1]	Four	Eleven	Twelve	Species	EC Number
Q6RUJ3	trypsin	IV	IX	XI	Trichinella spiralis	
Q9BJL7	trypsin	IV	IX	XI	Trichinella spiralis	
Q4FZN4	trypsin	IV	IX	XI	Xenopus laevis	
Q640E1	trypsin	IV	IX	XI	Xenopus laevis	
Q7T0T6	trypsin	IV	IX	XI	Xenopus laevis	
Q7Z155	trypsin	IV	IX	XII	Chiromantes haematocheir	
Q8T3A1	trypsin	IV	IX	XII	Ciona intestinalis	
Q4V675	trypsin	IV	IX	XII	Drosophila melanogaster	
Q8IRB8	trypsin	IV	IX	XII	Drosophila melanogaster	3.4.21.-
Q9VQ98	trypsin	IV	IX	XII	Drosophila melanogaster	3.4.21.-
Q9VR15	trypsin	IV	IX	XII	Drosophila melanogaster	3.4.21.-
Q9VUF0	trypsin	IV	IX	XII	Drosophila melanogaster	3.4.21.-
Q29LA1	trypsin	IV	IX	XII	Drosophila pseudoobscura	3.4.21.-
Q2LZS9	trypsin	IV	IX	XII	Drosophila pseudoobscura	3.4.21.-
Q2M157	trypsin	IV	IX	XII	Drosophila pseudoobscura	3.4.21.-
A7DZ33	trypsin	IV	IX	XII	Drosophila simulans	
A7DZ38	trypsin	IV	IX	XII	Drosophila simulans	
A7DZ34	trypsin	IV	IX	XII	Drosophila simulans	3.4.21.-
Q4PMM2	trypsin	IV	IX	XII	Ixodes scapularis	
Q95P15	trypsin	IV	IX	XII	Lygus hesperus	
Q6Y1Y7	trypsin	IV	IX	XII	Lygus lineolaris	
Q6Y1Y9	trypsin	IV	IX	XII	Lygus lineolaris	
Q6Y1Z0	trypsin	IV	IX	XII	Lygus lineolaris	
Q8WPE4	trypsin	IV	IX	XII	Lygus lineolaris	
O15944	trypsin	IV	IX	XII	Sarcophaga peregrina	
Q49QW1	trypsin	IV	IX	XII	Spodoptera litura	
Q4RH74	trypsin	IV	IX	XII	Tetraodon nigroviridis	
P22457	trypsin	IV	VI	IV	Bos taurus	3.4.21.21
P00745	trypsin	IV	VI	IV	Bos taurus	3.4.21.69
Q38J75	trypsin	IV	VI	IV	Canis familiaris	
Q28278	trypsin	IV	VI	IV	Canis familiaris	3.4.21.69
Q804X0	trypsin	IV	VI	IV	Fugu rubripes	3.4.21.21
P04070	trypsin	IV	VI	IV	Homo sapiens	3.4.21.69
P33587	trypsin	IV	VI	IV	Mus musculus	3.4.21.69
Q28661	trypsin	IV	VI	IV	Oryctolagus cuniculus	3.4.21.69
Q68FY8	trypsin	IV	VI	IV	Rattus norvegicus	
P31394	trypsin	IV	VI	IV	Rattus norvegicus	3.4.21.69
Q9GLP2	trypsin	IV	VI	IV	Sus scrofa	3.4.21.69
Q4SB50	trypsin	IV	VI	IV	Tetraodon nigroviridis	
Q4SB52	trypsin	IV	VI	IV	Tetraodon nigroviridis	
Q4SU99	trypsin	IV	VI	IV	Tetraodon nigroviridis	
A7SQF1	trypsin	IV	VI	IX	Nematostella vectensis	
A6QPZ2	trypsin	IV	VI	VI	Bos taurus	
Q3MHW2	trypsin	IV	VI	VI	Bos taurus	
P00741	trypsin	IV	VI	VI	Bos taurus	3.4.21.22
P00743	trypsin	IV	VI	VI	Bos taurus	3.4.21.6
P19540	trypsin	IV	VI	VI	Canis familiaris	3.4.21.22
P16295	trypsin	IV	VI	VI	Cavia porcellus	3.4.21.22
A4QP82	trypsin	IV	VI	VI	Danio rerio	
Q1RLV2	trypsin	IV	VI	VI	Danio rerio	
Q4V971	trypsin	IV	VI	VI	Danio rerio	
Q504H3	trypsin	IV	VI	VI	Danio rerio	
Q504K1	trypsin	IV	VI	VI	Danio rerio	
Q6PGW7	trypsin	IV	VI	VI	Danio rerio	
Q8AYE4	trypsin	IV	VI	VI	Danio rerio	
Q8JHD0	trypsin	IV	VI	VI	Danio rerio	

Table S2.4. (continuation)

UniProt	Label [1]	Four	Eleven	Twelve	Species	EC Number
Q90YK1	trypsin	IV	VI	VI	Danio rerio	
Q6SA95	trypsin	IV	VI	VI	Felis silvestris	3.4.21.22
Q804X2	trypsin	IV	VI	VI	Fugu rubripes	3.4.21.21
Q804W8	trypsin	IV	VI	VI	Fugu rubripes	3.4.21.22
Q804W9	trypsin	IV	VI	VI	Fugu rubripes	3.4.21.6
Q804X6	trypsin	IV	VI	VI	Gallus gallus	3.4.21.22
P25155	trypsin	IV	VI	VI	Gallus gallus	3.4.21.6
Q5JVE7	trypsin	IV	VI	VI	Homo sapiens	
P00740	trypsin	IV	VI	VI	Homo sapiens	3.4.21.22
P00742	trypsin	IV	VI	VI	Homo sapiens	3.4.21.6
A8CZ27	trypsin	IV	VI	VI	Macaca mulatta	
A0N065	trypsin	IV	VI	VI	Macaca mulatta	3.4.21.6
A0JLY3	trypsin	IV	VI	VI	Mus musculus	
Q3TBR2	trypsin	IV	VI	VI	Mus musculus	
Q3TDB9	trypsin	IV	VI	VI	Mus musculus	
Q3U3V1	trypsin	IV	VI	VI	Mus musculus	
Q4FJS7	trypsin	IV	VI	VI	Mus musculus	
P16294	trypsin	IV	VI	VI	Mus musculus	3.4.21.22
O88947	trypsin	IV	VI	VI	Mus musculus	3.4.21.6
Q58L94	trypsin	IV	VI	VI	Notechis scutatus	3.4.21.6
Q9GMD9	trypsin	IV	VI	VI	Ornithorhynchus anatinus	
P16292	trypsin	IV	VI	VI	Oryctolagus cuniculus	3.4.21.22
O19045	trypsin	IV	VI	VI	Oryctolagus cuniculus	3.4.21.6
P16291	trypsin	IV	VI	VI	Ovis aries	3.4.21.22
Q58L95	trypsin	IV	VI	VI	Oxyuranus microlepidotus	3.4.21.6
Q58L96	trypsin	IV	VI	VI	Oxyuranus scutellatus	3.4.21.6
Q95ND6	trypsin	IV	VI	VI	Pan troglodytes	
Q95ND7	trypsin	IV	VI	VI	Pan troglodytes	3.4.21.22
Q58L93	trypsin	IV	VI	VI	Pseudechis porphyriacus	3.4.21.6
Q6IT10	trypsin	IV	VI	VI	Pseudonaja textilis	
Q1L658	trypsin	IV	VI	VI	Pseudonaja textilis	3.4.21.6
Q1L659	trypsin	IV	VI	VI	Pseudonaja textilis	3.4.21.6
Q56VR3	trypsin	IV	VI	VI	Pseudonaja textilis	3.4.21.6
P16296	trypsin	IV	VI	VI	Rattus norvegicus	3.4.21.22
Q63207	trypsin	IV	VI	VI	Rattus norvegicus	3.4.21.6
Q19AZ6	trypsin	IV	VI	VI	Sus scrofa	
P16293	trypsin	IV	VI	VI	Sus scrofa	3.4.21.22
Q4RP66	trypsin	IV	VI	VI	Tetraodon nigroviridis	
Q4SUA2	trypsin	IV	VI	VI	Tetraodon nigroviridis	
Q4SVF9	trypsin	IV	VI	VI	Tetraodon nigroviridis	
P81428	trypsin	IV	VI	VI	Tropidechis carinatus	3.4.21.6
Q4QXT9	trypsin	IV	VI	VI	Tropidechis carinatus	3.4.21.6
A4VCE8	trypsin	IV	VI	VI	Xenopus laevis	
Q6PAG2	trypsin	IV	VI	VI	Xenopus laevis	
Q5FW21	trypsin	IV	VI	VI	Xenopus tropicalis	
Q5M8Y0	trypsin	IV	VI	VI	Xenopus tropicalis	
Q5DVT1	trypsin	IV	VI	VIII	Eptatretus burgeri	
A7RKX8	trypsin	IV	VI	VIII	Nematostella vectensis	
Q868H6	trypsin	IV	VI	XI	Branchiostoma belcheri	
Q868H5	trypsin	IV	VI	XII	Branchiostoma belcheri	
Q868H7	trypsin	IV	VI	XII	Branchiostoma belcheri	
Q7T3B6	trypsin	IV	VI	XII	Danio rerio	
P48740	trypsin	IV	VI	XII	Homo sapiens	3.4.21.-
Q9Y7A9	trypsin	IV	VI	XII	Metarhizium anisopliae	
P98064	trypsin	IV	VI	XII	Mus musculus	3.4.21.-
A7RXZ9	trypsin	IV	VI	XII	Nematostella vectensis	

Table S2.4. (continuation)

UniProt	Label [1]	Four	Eleven	Twelve	Species	EC Number
A7SSR9	trypsin	IV	VI	XII	Nematostella vectensis	
Q8CHN8	trypsin	IV	VI	XII	Rattus norvegicus	3.4.21.-
A6ANQ8	trypsin	IV	VI	XII	Vibrio campbellii	
Q6GPF9	trypsin	IV	VI	XII	Xenopus laevis	
Q9PU71	trypsin	IV	VI	XII	Xenopus laevis	
Q5FVZ2	trypsin	IV	VI	XII	Xenopus tropicalis	
A5HTZ6	trypsin	IV	VII	IX	Anas platyrhynchos	
A2VDV7	trypsin	IV	VII	IX	Bos taurus	
P79343	trypsin	IV	VII	IX	Bos taurus	3.4.21.10
Q28198	trypsin	IV	VII	IX	Bos taurus	3.4.21.68
A5HTZ8	trypsin	IV	VII	IX	Coturnix coturnix	
A5PF55	trypsin	IV	VII	IX	Danio rerio	
Q5PRA6	trypsin	IV	VII	IX	Danio rerio	
P15638	trypsin	IV	VII	IX	Desmodus rotundus	3.4.21.68
P49150	trypsin	IV	VII	IX	Desmodus rotundus	3.4.21.68
P98119	trypsin	IV	VII	IX	Desmodus rotundus	3.4.21.68
P98121	trypsin	IV	VII	IX	Desmodus rotundus	3.4.21.68
Q2VWB9	trypsin	IV	VII	IX	Gadus morhua	
Q3I1V3	trypsin	IV	VII	IX	Gorilla gorilla	
Q5G270	trypsin	IV	VII	IX	Gorilla gorilla	3.4.21.-
Q1RMF8	trypsin	IV	VII	IX	Homo sapiens	
Q96T73	trypsin	IV	VII	IX	Homo sapiens	
O15393	trypsin	IV	VII	IX	Homo sapiens	3.4.21.-
P56730	trypsin	IV	VII	IX	Homo sapiens	3.4.21.-
P57727	trypsin	IV	VII	IX	Homo sapiens	3.4.21.-
Q86WS5	trypsin	IV	VII	IX	Homo sapiens	3.4.21.-
Q9BYE2	trypsin	IV	VII	IX	Homo sapiens	3.4.21.-
P03951	trypsin	IV	VII	IX	Homo sapiens	3.4.21.27
P00750	trypsin	IV	VII	IX	Homo sapiens	3.4.21.68
Q5G3K8	trypsin	IV	VII	IX	Hoolock hoolock	
Q3I1U8	trypsin	IV	VII	IX	Hylobates syndactylus	
Q3I1U4	trypsin	IV	VII	IX	Macaca mulatta	
Q5G267	trypsin	IV	VII	IX	Macaca mulatta	3.4.21.-
Q2M1G4	trypsin	IV	VII	IX	Mus musculus	
Q3TZ06	trypsin	IV	VII	IX	Mus musculus	
Q3UKE3	trypsin	IV	VII	IX	Mus musculus	
Q3ZB06	trypsin	IV	VII	IX	Mus musculus	
O08762	trypsin	IV	VII	IX	Mus musculus	3.4.21.-
Q3V0Q7	trypsin	IV	VII	IX	Mus musculus	3.4.21.-
Q5U405	trypsin	IV	VII	IX	Mus musculus	3.4.21.-
Q8K1T0	trypsin	IV	VII	IX	Mus musculus	3.4.21.-
Q9JIQ8	trypsin	IV	VII	IX	Mus musculus	3.4.21.-
P23578	trypsin	IV	VII	IX	Mus musculus	3.4.21.10
Q3ZB05	trypsin	IV	VII	IX	Mus musculus	3.4.21.10
P11214	trypsin	IV	VII	IX	Mus musculus	3.4.21.68
A7S1T0	trypsin	IV	VII	IX	Nematostella vectensis	
A7S9K4	trypsin	IV	VII	IX	Nematostella vectensis	
A7S9K6	trypsin	IV	VII	IX	Nematostella vectensis	
A7SNJ9	trypsin	IV	VII	IX	Nematostella vectensis	
Q5G268	trypsin	IV	VII	IX	Nomascus leucogenys	3.4.21.-
Q8WPM7	trypsin	IV	VII	IX	Oikopleura dioica	
Q8MKB1	trypsin	IV	VII	IX	Oryctolagus cuniculus	3.4.21.68
Q9GL10	trypsin	IV	VII	IX	Ovis aries	3.4.21.10
Q3I1V4	trypsin	IV	VII	IX	Pan paniscus	
Q3I1V5	trypsin	IV	VII	IX	Pan troglodytes	
Q5G271	trypsin	IV	VII	IX	Pan troglodytes	3.4.21.-

Table S2.4. (continuation)

UniProt	Label [1]	Four	Eleven	Twelve	Species	EC Number
Q59IT0	trypsin	IV	VII	IX	Paralichthys olivaceus	
Q5RDX7	trypsin	IV	VII	IX	Pongo abelii	
Q5R8J0	trypsin	IV	VII	IX	Pongo abelii	3.4.21.68
Q5G269	trypsin	IV	VII	IX	Pongo pygmaeus	3.4.21.-
Q5G3K5	trypsin	IV	VII	IX	Pygathrix nemaeus	
Q6P7D7	trypsin	IV	VII	IX	Rattus norvegicus	
Q920K3	trypsin	IV	VII	IX	Rattus norvegicus	
P83748	trypsin	IV	VII	IX	Rattus norvegicus	3.4.21.-
P29293	trypsin	IV	VII	IX	Rattus norvegicus	3.4.21.10
P19637	trypsin	IV	VII	IX	Rattus norvegicus	3.4.21.68
Q5G3K7	trypsin	IV	VII	IX	Rhinopithecus bieti	
Q5G265	trypsin	IV	VII	IX	Saguinus labiatus	3.4.21.-
Q9XZM7	trypsin	IV	VII	IX	Strongylocentrotus purpuratus	
A7VK02	trypsin	IV	VII	IX	Sus scrofa	
P08001	trypsin	IV	VII	IX	Sus scrofa	3.4.21.10
Q8SQ23	trypsin	IV	VII	IX	Sus scrofa	3.4.21.68
Q4SPG0	trypsin	IV	VII	IX	Tetraodon nigroviridis	
Q4T7E2	trypsin	IV	VII	IX	Tetraodon nigroviridis	
Q5G3K6	trypsin	IV	VII	IX	Trachypithecus francoisi	
Q5G266	trypsin	IV	VII	IX	Trachypithecus phayrei	3.4.21.-
Q4V7J4	trypsin	IV	VII	IX	Xenopus laevis	
Q6NUF5	trypsin	IV	VII	IX	Xenopus laevis	
Q9DGR2	trypsin	IV	VII	IX	Xenopus laevis	
Q16QB1	trypsin	IV	VII	VIII	Aedes aegypti	
Q17PV5	trypsin	IV	VII	VIII	Aedes aegypti	
Q7PWE2	trypsin	IV	VII	VIII	Anopheles gambiae	
Q7Q5Z6	trypsin	IV	VII	VIII	Anopheles gambiae	
P06868	trypsin	IV	VII	VIII	Bos taurus	3.4.21.7
P80009	trypsin	IV	VII	VIII	Canis familiaris	3.4.21.7
Q60491	trypsin	IV	VII	VIII	Cavia porcellus	
Q8T3A0	trypsin	IV	VII	VIII	Ciona intestinalis	
Q8T3A3	trypsin	IV	VII	VIII	Ciona intestinalis	
Q9XY63	trypsin	IV	VII	VIII	Ctenocephalides felis	
A4FVH9	trypsin	IV	VII	VIII	Danio rerio	
Q5BL24	trypsin	IV	VII	VIII	Danio rerio	
Q7ZZ80	trypsin	IV	VII	VIII	Danio rerio	
Q8AVB0	trypsin	IV	VII	VIII	Danio rerio	
Q6PBA6	trypsin	IV	VII	VIII	Danio rerio	3.4.21.7
A1Z7M5	trypsin	IV	VII	VIII	Drosophila melanogaster	3.4.21.-
Q28ZD3	trypsin	IV	VII	VIII	Drosophila pseudoobscura	3.4.21.-
Q2M0M7	trypsin	IV	VII	VIII	Drosophila pseudoobscura	3.4.21.-
P80010	trypsin	IV	VII	VIII	Equus caballus	3.4.21.7
Q29485	trypsin	IV	VII	VIII	Erinaceus europaeus	3.4.21.7
Q1HP67	trypsin	IV	VII	VIII	Homo sapiens	
Q68DS2	trypsin	IV	VII	VIII	Homo sapiens	
P08519	trypsin	IV	VII	VIII	Homo sapiens	3.4.21.-
P10323	trypsin	IV	VII	VIII	Homo sapiens	3.4.21.10
P00747	trypsin	IV	VII	VIII	Homo sapiens	3.4.21.7
P12545	trypsin	IV	VII	VIII	Macaca mulatta	3.4.21.7
O18783	trypsin	IV	VII	VIII	Macropus eugenii	3.4.21.7
Q5MPB3	trypsin	IV	VII	VIII	Manduca sexta	
Q5MPC1	trypsin	IV	VII	VIII	Manduca sexta	
Q2UVH8	trypsin	IV	VII	VIII	Meleagris gallopavo	3.4.21.10
P20918	trypsin	IV	VII	VIII	Mus musculus	3.4.21.7
Q3V1T9	trypsin	IV	VII	VIII	Mus musculus	3.4.21.7
A7RU68	trypsin	IV	VII	VIII	Nematostella vectensis	

Table S2.4. (continuation)

UniProt	Label [1]	Four	Eleven	Twelve	Species	EC Number
A5HTZ7	trypsin	IV	VII	VIII	Numida meleagris	
Q5DVP8	trypsin	IV	VII	VIII	Oncorhynchus mykiss	3.4.21.7
P48038	trypsin	IV	VII	VIII	Oryctolagus cuniculus	3.4.21.10
A4UWM5	trypsin	IV	VII	VIII	Oryzias latipes	
A4UWM6	trypsin	IV	VII	VIII	Oryzias latipes	
Q50LG6	trypsin	IV	VII	VIII	Oryzias latipes	3.4.21.7
P81286	trypsin	IV	VII	VIII	Ovis aries	3.4.21.7
O46507	trypsin	IV	VII	VIII	Papio hamadryas	
Q2TJC1	trypsin	IV	VII	VIII	Phlebotomus ariasi	
Q5R8X6	trypsin	IV	VII	VIII	Pongo abelii	3.4.21.7
Q01177	trypsin	IV	VII	VIII	Rattus norvegicus	3.4.21.7
P06867	trypsin	IV	VII	VIII	Sus scrofa	3.4.21.7
Q2JM42	trypsin	IV	VII	VIII	Synechococcus sp	
Q2JXR1	trypsin	IV	VII	VIII	Synechococcus sp	
Q4SGT4	trypsin	IV	VII	VIII	Tetraodon nigroviridis	
A7RKX5	trypsin	IV	VII	X	Nematostella vectensis	
A7S8Y5	trypsin	IV	VII	X	Nematostella vectensis	
Q0IFC0	trypsin	IV	VII	XI	Aedes aegypti	
Q7QCS5	trypsin	IV	VII	XI	Anopheles gambiae	
Q6B4R4	trypsin	IV	VII	XI	Bos taurus	
P98072	trypsin	IV	VII	XI	Bos taurus	3.4.21.9
Q19MT4	trypsin	IV	VII	XI	Bubalus bubalis	
Q294V2	trypsin	IV	VII	XI	Drosophila pseudoobscura	3.4.21.-
P98073	trypsin	IV	VII	XI	Homo sapiens	3.4.21.9
Q8CAN9	trypsin	IV	VII	XI	Mus musculus	
Q9QYZ9	trypsin	IV	VII	XI	Mus musculus	3.4.21.-
P97435	trypsin	IV	VII	XI	Mus musculus	3.4.21.9
P98074	trypsin	IV	VII	XI	Sus scrofa	3.4.21.9
A7UNU8	trypsin	IV	VII	XI	Tyrophagus putrescentiae	
A0RZI1	trypsin	IV	VII	XII	Azumapecten farreri	
A0S0P9	trypsin	IV	VII	XII	Azumapecten farreri	
Q6Q0I7	trypsin	IV	VII	XII	Bos taurus	
Q5NTB3	trypsin	IV	VII	XII	Bos taurus	3.4.21.27
Q2KJ63	trypsin	IV	VII	XII	Bos taurus	3.4.21.34
A8E5D9	trypsin	IV	VII	XII	Danio rerio	
Q5BAR4	trypsin	IV	VII	XII	Emericella nidulans	
Q7RTZ1	trypsin	IV	VII	XII	Homo sapiens	3.4.21.-
Q9NRS4	trypsin	IV	VII	XII	Homo sapiens	3.4.21.-
P03952	trypsin	IV	VII	XII	Homo sapiens	3.4.21.34
Q8VCA5	trypsin	IV	VII	XII	Mus musculus	3.4.21.-
Q91Y47	trypsin	IV	VII	XII	Mus musculus	3.4.21.27
P26262	trypsin	IV	VII	XII	Mus musculus	3.4.21.34
A7RGS8	trypsin	IV	VII	XII	Nematostella vectensis	
A7RR41	trypsin	IV	VII	XII	Nematostella vectensis	
A7RYF8	trypsin	IV	VII	XII	Nematostella vectensis	
A7S5M4	trypsin	IV	VII	XII	Nematostella vectensis	
A7SGX1	trypsin	IV	VII	XII	Nematostella vectensis	
A7SNB8	trypsin	IV	VII	XII	Nematostella vectensis	
A7SX50	trypsin	IV	VII	XII	Nematostella vectensis	
Q95ME7	trypsin	IV	VII	XII	Oryctolagus cuniculus	
Q5R502	trypsin	IV	VII	XII	Pongo abelii	
Q5R976	trypsin	IV	VII	XII	Pongo abelii	
Q5FVS2	trypsin	IV	VII	XII	Rattus norvegicus	
P14272	trypsin	IV	VII	XII	Rattus norvegicus	3.4.21.34
Q82LH6	trypsin	IV	VII	XII	Streptomyces avermitilis	
O97506	trypsin	IV	VII	XII	Sus scrofa	

Table S2.4. (continuation)

UniProt	Label [1]	Four	Eleven	Twelve	Species	EC Number
Q6AZS7	trypsin	IV	VII	XII	Xenopus laevis	
Q28GN1	trypsin	IV	VII	XII	Xenopus tropicalis	
A7SQE8	trypsin	IV	VIII	X	Nematostella vectensis	
A7SB63	trypsin	IV	VIII	XI	Nematostella vectensis	
A7SQF0	trypsin	IV	VIII	XI	Nematostella vectensis	
Q8K4I7	trypsin	IV	X	IX	Mus musculus	
Q175S1	trypsin	IV	X	VIII	Aedes aegypti	
Q17IQ0	trypsin	IV	X	VIII	Aedes aegypti	
Q7QC30	trypsin	IV	X	VIII	Anopheles gambiae	
Q9NFY2	trypsin	IV	X	VIII	Anopheles gambiae	
Q94FS3	trypsin	IV	X	VIII	Aphanomyces astaci	
A6QPI9	trypsin	IV	X	VIII	Bos taurus	
Q8HYJ2	trypsin	IV	X	VIII	Bos taurus	
A6QQ05	trypsin	IV	X	VIII	Bos taurus	3.4.21.59
Q29464	trypsin	IV	X	VIII	Bos taurus	3.4.21.59
Q17800	trypsin	IV	X	VIII	Caenorhabditis elegans	
P15944	trypsin	IV	X	VIII	Canis familiaris	3.4.21.59
Q9XY55	trypsin	IV	X	VIII	Ctenocephalides felis	
Q7K1E3	trypsin	IV	X	VIII	Drosophila melanogaster	3.4.21.-
Q95RS6	trypsin	IV	X	VIII	Drosophila melanogaster	3.4.21.-
Q9VW19	trypsin	IV	X	VIII	Drosophila melanogaster	3.4.21.-
Q7YS62	trypsin	IV	X	VIII	Equus caballus	3.4.21.59
A8C6G1	trypsin	IV	X	VIII	Gorilla gorilla	
A8C6G4	trypsin	IV	X	VIII	Gorilla gorilla	
A8C6G6	trypsin	IV	X	VIII	Gorilla gorilla	
Q6B051	trypsin	IV	X	VIII	Homo sapiens	
Q6FHB8	trypsin	IV	X	VIII	Homo sapiens	
Q6NZY1	trypsin	IV	X	VIII	Homo sapiens	
Q86TM8	trypsin	IV	X	VIII	Homo sapiens	
Q86UA5	trypsin	IV	X	VIII	Homo sapiens	
Q96RZ6	trypsin	IV	X	VIII	Homo sapiens	
Q6UWB4	trypsin	IV	X	VIII	Homo sapiens	3.4.21.-
P20231	trypsin	IV	X	VIII	Homo sapiens	3.4.21.59
Q15661	trypsin	IV	X	VIII	Homo sapiens	3.4.21.59
Q9UI38	trypsin	IV	X	VIII	Homo sapiens	3.4.25.-
A8C738	trypsin	IV	X	VIII	Macaca fascicularis	
A8C740	trypsin	IV	X	VIII	Macaca fascicularis	
Q5IRX0	trypsin	IV	X	VIII	Mayetiola destructor	
P50342	trypsin	IV	X	VIII	Meriones unguiculatus	3.4.21.59
A1Z090	trypsin	IV	X	VIII	Mus musculus	
Q921N4	trypsin	IV	X	VIII	Mus musculus	
Q14BX2	trypsin	IV	X	VIII	Mus musculus	3.4.21.-
Q920S2	trypsin	IV	X	VIII	Mus musculus	3.4.21.-
P21845	trypsin	IV	X	VIII	Mus musculus	3.4.21.59
Q02844	trypsin	IV	X	VIII	Mus musculus	3.4.21.59
A7RLC0	trypsin	IV	X	VIII	Nematostella vectensis	
Q9XSM2	trypsin	IV	X	VIII	Ovis aries	3.4.21.59
A8C590	trypsin	IV	X	VIII	Pan troglodytes	
O97399	trypsin	IV	X	VIII	Phaedon cochleariae	3.4.21.4
A8CXJ3	trypsin	IV	X	VIII	Pongo abelii	
A8CXJ5	trypsin	IV	X	VIII	Pongo abelii	
A8CXJ6	trypsin	IV	X	VIII	Pongo abelii	
A8CXJ8	trypsin	IV	X	VIII	Pongo abelii	
Q6P6W8	trypsin	IV	X	VIII	Rattus norvegicus	
P27435	trypsin	IV	X	VIII	Rattus norvegicus	3.4.21.59
P50343	trypsin	IV	X	VIII	Rattus norvegicus	3.4.21.59

Table S2.4. (continuation)

UniProt	Label [1]	Four	Eleven	Twelve	Species	EC Number
Q9N2D1	trypsin	IV	X	VIII	Sus scrofa	3.4.21.59
Q9BK47	trypsin	IV	X	X	Luidia foliolata	
Q0IF82	trypsin	IV	X	XI	Aedes aegypti	
Q16G07	trypsin	IV	X	XI	Aedes aegypti	
Q16G08	trypsin	IV	X	XI	Aedes aegypti	
Q16ID8	trypsin	IV	X	XI	Aedes aegypti	
Q16PS2	trypsin	IV	X	XI	Aedes aegypti	
Q16TL8	trypsin	IV	X	XI	Aedes aegypti	
Q171W0	trypsin	IV	X	XI	Aedes aegypti	
Q171W1	trypsin	IV	X	XI	Aedes aegypti	
Q175R9	trypsin	IV	X	XI	Aedes aegypti	
Q175S0	trypsin	IV	X	XI	Aedes aegypti	
Q179B1	trypsin	IV	X	XI	Aedes aegypti	
Q179I4	trypsin	IV	X	XI	Aedes aegypti	
Q179I5	trypsin	IV	X	XI	Aedes aegypti	
Q179I6	trypsin	IV	X	XI	Aedes aegypti	
Q179I7	trypsin	IV	X	XI	Aedes aegypti	
Q17BD9	trypsin	IV	X	XI	Aedes aegypti	
Q17BG4	trypsin	IV	X	XI	Aedes aegypti	
Q17BS3	trypsin	IV	X	XI	Aedes aegypti	
Q17HP8	trypsin	IV	X	XI	Aedes aegypti	
Q17HQ4	trypsin	IV	X	XI	Aedes aegypti	
Q17LZ9	trypsin	IV	X	XI	Aedes aegypti	
Q17PV4	trypsin	IV	X	XI	Aedes aegypti	
Q8T9U6	trypsin	IV	X	XI	Aedes aegypti	
P29786	trypsin	IV	X	XI	Aedes aegypti	3.4.21.4
Q17036	trypsin	IV	X	XI	Anopheles gambiae	
Q17039	trypsin	IV	X	XI	Anopheles gambiae	
Q7PV63	trypsin	IV	X	XI	Anopheles gambiae	
Q7PWE3	trypsin	IV	X	XI	Anopheles gambiae	
Q7Q153	trypsin	IV	X	XI	Anopheles gambiae	
Q7Q432	trypsin	IV	X	XI	Anopheles gambiae	
Q7Q8F9	trypsin	IV	X	XI	Anopheles gambiae	
Q7Q9W2	trypsin	IV	X	XI	Anopheles gambiae	
Q7Q9W5	trypsin	IV	X	XI	Anopheles gambiae	
Q7Q9X5	trypsin	IV	X	XI	Anopheles gambiae	
Q64ID1	trypsin	IV	X	XI	Anthonomus grandis	
Q8I9P2	trypsin	IV	X	XI	Aplysina fistularis	
P00765	trypsin	IV	X	XI	Astacus fluviatilis	3.4.21.4
Q52V24	trypsin	IV	X	XI	Astacus leptodactylus	
Q6MJY6	trypsin	IV	X	XI	Bdellovibrio bacteriovorus	
Q1N1S5	trypsin	IV	X	XI	Bermanella marisrubri	
Q9GQ02	trypsin	IV	X	XI	Biomphalaria glabrata	
Q3Y9L9	trypsin	IV	X	XI	Blattella germanica	
O16126	trypsin	IV	X	XI	Boltenia villosa	
A2VE36	trypsin	IV	X	XI	Bos taurus	3.4.21.-
Q6Q6S3	trypsin	IV	X	XI	Callinectes sapidus	
P19236	trypsin	IV	X	XI	Canis familiaris	3.4.21.-
Q9XY51	trypsin	IV	X	XI	Ctenocephalides felis	
Q9XY53	trypsin	IV	X	XI	Ctenocephalides felis	
Q9XY56	trypsin	IV	X	XI	Ctenocephalides felis	
Q9XY57	trypsin	IV	X	XI	Ctenocephalides felis	
Q9XY59	trypsin	IV	X	XI	Ctenocephalides felis	
Q9GTK2	trypsin	IV	X	XI	Culex quinquefasciatus	
Q5QBF4	trypsin	IV	X	XI	Culicoides sonorensis	
Q5QBG0	trypsin	IV	X	XI	Culicoides sonorensis	

Table S2.4. (continuation)

UniProt	Label [1]	Four	Eleven	Twelve	Species	EC Number
Q5QBG2	trypsin	IV	X	XI	Culicoides sonorensis	
A3KMS5	trypsin	IV	X	XI	Danio rerio	
A4FUL1	trypsin	IV	X	XI	Danio rerio	
Q1ED24	trypsin	IV	X	XI	Danio rerio	
Q4V8T5	trypsin	IV	X	XI	Danio rerio	
Q504J5	trypsin	IV	X	XI	Danio rerio	
Q8JHC9	trypsin	IV	X	XI	Danio rerio	
A1KXH3	trypsin	IV	X	XI	Dermatophagoides farinae	
P49275	trypsin	IV	X	XI	Dermatophagoides farinae	3.4.21.-
A7UNT7	trypsin	IV	X	XI	Dermatophagoides pteronyssinus	
Q8MWR5	trypsin	IV	X	XI	Dermatophagoides pteronyssinus	
P39675	trypsin	IV	X	XI	Dermatophagoides pteronyssinus	3.4.21.-
Q4V440	trypsin	IV	X	XI	Drosophila melanogaster	
Q8MQM9	trypsin	IV	X	XI	Drosophila melanogaster	
Q8T8X4	trypsin	IV	X	XI	Drosophila melanogaster	
A1Z7H8	trypsin	IV	X	XI	Drosophila melanogaster	3.4.21.-
A8DYG0	trypsin	IV	X	XI	Drosophila melanogaster	3.4.21.-
Q05319	trypsin	IV	X	XI	Drosophila melanogaster	3.4.21.-
Q8IQ89	trypsin	IV	X	XI	Drosophila melanogaster	3.4.21.-
Q8MS52	trypsin	IV	X	XI	Drosophila melanogaster	3.4.21.-
Q9I7V4	trypsin	IV	X	XI	Drosophila melanogaster	3.4.21.-
Q9VBY4	trypsin	IV	X	XI	Drosophila melanogaster	3.4.21.-
Q9VK10	trypsin	IV	X	XI	Drosophila melanogaster	3.4.21.-
Q9VUG2	trypsin	IV	X	XI	Drosophila melanogaster	3.4.21.-
Q9VZT0	trypsin	IV	X	XI	Drosophila melanogaster	3.4.21.-
Q9W0Z7	trypsin	IV	X	XI	Drosophila melanogaster	3.4.21.-
Q297G1	trypsin	IV	X	XI	Drosophila pseudoobscura	
Q28WQ9	trypsin	IV	X	XI	Drosophila pseudoobscura	3.4.21.-
Q28Y63	trypsin	IV	X	XI	Drosophila pseudoobscura	3.4.21.-
Q28Z30	trypsin	IV	X	XI	Drosophila pseudoobscura	3.4.21.-
Q28Z63	trypsin	IV	X	XI	Drosophila pseudoobscura	3.4.21.-
Q28Z76	trypsin	IV	X	XI	Drosophila pseudoobscura	3.4.21.-
Q29DF3	trypsin	IV	X	XI	Drosophila pseudoobscura	3.4.21.-
Q29KI7	trypsin	IV	X	XI	Drosophila pseudoobscura	3.4.21.-
Q2M165	trypsin	IV	X	XI	Drosophila pseudoobscura	3.4.21.-
O97370	trypsin	IV	X	XI	Euroglyphus maynei	3.4.21.-
Q804X1	trypsin	IV	X	XI	Fugu rubripes	3.4.21.21
P35049	trypsin	IV	X	XI	Fusarium oxysporum	3.4.21.4
Q804X7	trypsin	IV	X	XI	Gallus gallus	3.4.21.21
Q804X5	trypsin	IV	X	XI	Gallus gallus	3.4.21.69
Q2S709	trypsin	IV	X	XI	Hahella chejuensis	
Q2SHS3	trypsin	IV	X	XI	Hahella chejuensis	
Q966V4	trypsin	IV	X	XI	Halocynthia roretzi	
Q25101	trypsin	IV	X	XI	Herdmania momus	
Q9GRW0	trypsin	IV	X	XI	Holotrichia diomphalia	
A3RKG7	trypsin	IV	X	XI	Homo sapiens	
Q8WVC1	trypsin	IV	X	XI	Homo sapiens	
Q7RTY3	trypsin	IV	X	XI	Homo sapiens	3.4.21.-
O00187	trypsin	IV	X	XI	Homo sapiens	3.4.21.104
Q9Y5Y6	trypsin	IV	X	XI	Homo sapiens	3.4.21.109
P08709	trypsin	IV	X	XI	Homo sapiens	3.4.21.21
Q9TY16	trypsin	IV	X	XI	Litopenaeus vannamei	3.4.21.4
A0N0C5	trypsin	IV	X	XI	Macaca mulatta	
Q6RUT2	trypsin	IV	X	XI	Mus musculus	
Q80UR4	trypsin	IV	X	XI	Mus musculus	
Q99MS4	trypsin	IV	X	XI	Mus musculus	3.4.21.-

Table S2.4. (continuation)

UniProt	Label [1]	Four	Eleven	Twelve	Species	EC Number
P56677	trypsin	IV	X	XI	Mus musculus	3.4.21.109
Q543E3	trypsin	IV	X	XI	Mus musculus	3.4.21.109
Q1DBS1	trypsin	IV	X	XI	Myxococcus xanthus	3.4.21.-
A7SWQ5	trypsin	IV	X	XI	Nematostella vectensis	
Q8MNY6	trypsin	IV	X	XI	Nilaparvata lugens	
Q9XSM1	trypsin	IV	X	XI	Ovis aries	3.4.21.59
O46151	trypsin	IV	X	XI	Pacifastacus leniusculus	3.4.21.4
Q2F9P4	trypsin	IV	X	XI	Pan paniscus	3.4.21.21
Q2F9P2	trypsin	IV	X	XI	Pan troglodytes	3.4.21.21
Q967X8	trypsin	IV	X	XI	Panulirus argus	
O62561	trypsin	IV	X	XI	Penaeus vannamei	3.4.21.4
O62562	trypsin	IV	X	XI	Penaeus vannamei	3.4.21.4
Q27761	trypsin	IV	X	XI	Penaeus vannamei	3.4.21.4
Q7Z0G3	trypsin	IV	X	XI	Phlebotomus papatasi	
Q2M412	trypsin	IV	X	XI	Phytophthora infestans	
Q2F9N4	trypsin	IV	X	XI	Pongo pygmaeus	
Q2F9N5	trypsin	IV	X	XI	Pongo pygmaeus	
A1SY68	trypsin	IV	X	XI	Psychromonas ingrahamii	
A1ED51	trypsin	IV	X	XI	Radix peregra	
Q9JJI7	trypsin	IV	X	XI	Rattus norvegicus	3.4.21.109
Q6VPU6	trypsin	IV	X	XI	Sarcoptes scabiei	
A7EMI6	trypsin	IV	X	XI	Sclerotinia sclerotiorum	
P35048	trypsin	IV	X	XI	Simulium vittatum	3.4.21.4
Q8SQ44	trypsin	IV	X	XI	Sus scrofa	
Q8I6J9	trypsin	IV	X	XI	Tenebrio molitor	
Q4SUA1	trypsin	IV	X	XI	Tetraodon nigroviridis	
A4V8W4	trypsin	IV	X	XI	Trichoderma harzianum	
Q6V1Q1	trypsin	IV	X	XI	Verticillium dahliae	
A7K038	trypsin	IV	X	XI	Vibrio antiquarius	
A7K6I7	trypsin	IV	X	XI	Vibrio antiquarius	
A7MT30	trypsin	IV	X	XI	Vibrio campbellii	
Q9KSQ6	trypsin	IV	X	XI	Vibrio cholerae	
A6AY61	trypsin	IV	X	XI	Vibrio parahaemolyticus	
Q87P76	trypsin	IV	X	XI	Vibrio parahaemolyticus	
Q7MK56	trypsin	IV	X	XI	Vibrio vulnificus	
Q7ML92	trypsin	IV	X	XI	Vibrio vulnificus	
A5L636	trypsin	IV	X	XI	Vibrionales bacterium	
A2VD99	trypsin	IV	X	XI	Xenopus laevis	
Q6GNA2	trypsin	IV	X	XI	Xenopus laevis	
Q6GNK3	trypsin	IV	X	XI	Xenopus laevis	
Q0V9B6	trypsin	IV	X	XI	Xenopus tropicalis	
Q28EV7	trypsin	IV	X	XI	Xenopus tropicalis	
Q171W2	trypsin	IV	X	XII	Aedes aegypti	
Q17IP8	trypsin	IV	X	XII	Aedes aegypti	
Q17IP9	trypsin	IV	X	XII	Aedes aegypti	
P35040	trypsin	IV	X	XII	Anopheles gambiae	3.4.21.4
P91894	trypsin	IV	X	XII	Arenicola marina	
O02570	trypsin	IV	X	XII	Culex quinquefasciatus	
Q5QBG5	trypsin	IV	X	XII	Culicoides sonorensis	
Q24091	trypsin	IV	X	XII	Drosophila melanogaster	
Q29QP6	trypsin	IV	X	XII	Drosophila melanogaster	
Q8IPY7	trypsin	IV	X	XII	Drosophila melanogaster	3.4.21.-
Q9VLF5	trypsin	IV	X	XII	Drosophila melanogaster	3.4.21.-
Q9W5U8	trypsin	IV	X	XII	Drosophila melanogaster	3.4.21.-
Q29JR8	trypsin	IV	X	XII	Drosophila pseudoobscura	3.4.21.-
Q9BQR3	trypsin	IV	X	XII	Homo sapiens	3.4.21.-

Table S2.4. (continuation)

UniProt	Label [1]	Four	Eleven	Twelve	Species	EC Number
Q6QX59	trypsin	IV	X	XII	Lepeophtheirus salmonis	3.4.21.4
Q5IY40	trypsin	IV	X	XII	Mayetiola destructor	
Q5IY43	trypsin	IV	X	XII	Mayetiola destructor	
Q5IY44	trypsin	IV	X	XII	Mayetiola destructor	
Q3UP47	trypsin	IV	X	XII	Mus musculus	
Q8BJR6	trypsin	IV	X	XII	Mus musculus	3.4.21.-
P03953	trypsin	IV	X	XII	Mus musculus	3.4.21.46
Q675S0	trypsin	IV	X	XII	Oikopleura dioica	
Q8WR10	trypsin	IV	X	XII	Paralithodes camtschaticus	
A3E0P9	trypsin	IV	X	XII	Penaeus monodon	
A4GE68	trypsin	IV	X	XII	Penaeus monodon	
O74696	trypsin	IV	X	XII	Phaeosphaeria nodorum	
A2I7J3	trypsin	IV	X	XII	Portunus pelagicus	
Q6IE60	trypsin	IV	X	XII	Rattus norvegicus	
Q6BEA2	trypsin	IV	X	XII	Rattus norvegicus	3.4.21.-
Q9XYY0	trypsin	IV	X	XII	Rhyzopertha dominica	
Q9XYY1	trypsin	IV	X	XII	Rhyzopertha dominica	
O96899	trypsin	IV	X	XII	Scolopendra subspinipes	
O76519	trypsin	IV	X	XII	Stomoxys calcitrans	
Q82A51	trypsin	IV	X	XII	Streptomyces avermitilis	
P80420	trypsin	IV	X	XII	Streptomyces exfoliatus	3.4.21.-
Q54179	trypsin	IV	X	XII	Streptomyces glaucescens	3.4.21.-
Q6I698	trypsin	IV	X	XII	Streptomyces griseus	
Q6PPC0	trypsin	IV	X	XII	Streptomyces griseus	
P00775	trypsin	IV	X	XII	Streptomyces griseus	3.4.21.4
Q9KRJ1	trypsin	IV	X	XII	Vibrio cholerae	
Q4KLE1	trypsin	IV	X	XII	Xenopus laevis	
Q640F8	trypsin	IV	X	XII	Xenopus laevis	
Q9DGR3	trypsin	IV	X	XII	Xenopus laevis	
A7S9G1	trypsin	IV	XI	IV	Nematostella vectensis	
P98139	trypsin	IV	XI	IV	Oryctolagus cuniculus	3.4.21.21
Q8K3U6	trypsin	IV	XI	IV	Rattus norvegicus	3.4.21.21
Q19AZ7	trypsin	IV	XI	IV	Sus scrofa	
Q16WL3	trypsin	IV	XI	IX	Aedes aegypti	
Q17PV2	trypsin	IV	XI	IX	Aedes aegypti	
Q9NB91	trypsin	IV	XI	IX	Agrotis ipsilon	
Q7PIQ7	trypsin	IV	XI	IX	Anopheles gambiae	
Q7PNR7	trypsin	IV	XI	IX	Anopheles gambiae	
Q7Q530	trypsin	IV	XI	IX	Anopheles gambiae	
Q9NAT0	trypsin	IV	XI	IX	Anopheles gambiae	
Q9NJS5	trypsin	IV	XI	IX	Anopheles gambiae	
A1L5C6	trypsin	IV	XI	IX	Bos taurus	
Q05589	trypsin	IV	XI	IX	Bos taurus	3.4.21.73
Q23528	trypsin	IV	XI	IX	Caenorhabditis elegans	
Q6QNF4	trypsin	IV	XI	IX	Canis familiaris	3.4.21.-
Q8QGV0	trypsin	IV	XI	IX	Cyprinus carpio	
A0JMD5	trypsin	IV	XI	IX	Danio rerio	
A1L2D9	trypsin	IV	XI	IX	Danio rerio	
A3KP18	trypsin	IV	XI	IX	Danio rerio	
A5D6S2	trypsin	IV	XI	IX	Danio rerio	
A8DZG9	trypsin	IV	XI	IX	Danio rerio	
Q08CS9	trypsin	IV	XI	IX	Danio rerio	
Q0P416	trypsin	IV	XI	IX	Danio rerio	
Q1JQ29	trypsin	IV	XI	IX	Danio rerio	
Q1RLR1	trypsin	IV	XI	IX	Danio rerio	
Q502D2	trypsin	IV	XI	IX	Danio rerio	

Table S2.4. (continuation)

UniProt	Label [1]	Four	Eleven	Twelve	Species	EC Number
Q8MRY3	trypsin	IV	XI	IX	Drosophila melanogaster	
Q960G6	trypsin	IV	XI	IX	Drosophila melanogaster	
Q9BI19	trypsin	IV	XI	IX	Drosophila melanogaster	
Q9U1I2	trypsin	IV	XI	IX	Drosophila melanogaster	
Q9U1I3	trypsin	IV	XI	IX	Drosophila melanogaster	
A1Z7M3	trypsin	IV	XI	IX	Drosophila melanogaster	3.4.21.-
Q8IQB8	trypsin	IV	XI	IX	Drosophila melanogaster	3.4.21.-
Q9VSU2	trypsin	IV	XI	IX	Drosophila melanogaster	3.4.21.-
Q9W2C8	trypsin	IV	XI	IX	Drosophila melanogaster	3.4.21.-
P35004	trypsin	IV	XI	IX	Drosophila melanogaster	3.4.21.4
Q28XQ9	trypsin	IV	XI	IX	Drosophila pseudoobscura	3.4.21.-
Q28XR0	trypsin	IV	XI	IX	Drosophila pseudoobscura	3.4.21.-
Q9NBC9	trypsin	IV	XI	IX	Glossina morsitans	
Q0P513	trypsin	IV	XI	IX	Homo sapiens	
Q0P514	trypsin	IV	XI	IX	Homo sapiens	
Q0X0F2	trypsin	IV	XI	IX	Homo sapiens	
Q5PY49	trypsin	IV	XI	IX	Homo sapiens	
Q8IZZ5	trypsin	IV	XI	IX	Homo sapiens	
Q96EF3	trypsin	IV	XI	IX	Homo sapiens	
O60235	trypsin	IV	XI	IX	Homo sapiens	3.4.21.-
Q04756	trypsin	IV	XI	IX	Homo sapiens	3.4.21.-
Q4W5P3	trypsin	IV	XI	IX	Homo sapiens	3.4.21.-
Q6ZMR5	trypsin	IV	XI	IX	Homo sapiens	3.4.21.-
Q6ZWK6	trypsin	IV	XI	IX	Homo sapiens	3.4.21.-
Q9GZN4	trypsin	IV	XI	IX	Homo sapiens	3.4.21.-
Q9H3S3	trypsin	IV	XI	IX	Homo sapiens	3.4.21.-
Q9UL52	trypsin	IV	XI	IX	Homo sapiens	3.4.21.-
P05981	trypsin	IV	XI	IX	Homo sapiens	3.4.21.106
P00748	trypsin	IV	XI	IX	Homo sapiens	3.4.21.38
P00749	trypsin	IV	XI	IX	Homo sapiens	3.4.21.73
Q25082	trypsin	IV	XI	IX	Hypoderma lineatum	
P35587	trypsin	IV	XI	IX	Hypoderma lineatum	3.4.21.-
P35044	trypsin	IV	XI	IX	Lucilia cuprina	3.4.21.4
Q800Y7	trypsin	IV	XI	IX	Meleagris gallopavo	
A2RSL0	trypsin	IV	XI	IX	Mus musculus	
Q0VBA8	trypsin	IV	XI	IX	Mus musculus	
Q0X0F1	trypsin	IV	XI	IX	Mus musculus	
Q3TDU8	trypsin	IV	XI	IX	Mus musculus	
Q3U0U6	trypsin	IV	XI	IX	Mus musculus	
Q545J3	trypsin	IV	XI	IX	Mus musculus	
Q7TML0	trypsin	IV	XI	IX	Mus musculus	
Q8CDR0	trypsin	IV	XI	IX	Mus musculus	
Q8VCS4	trypsin	IV	XI	IX	Mus musculus	
Q147T9	trypsin	IV	XI	IX	Mus musculus	3.4.21.-
Q14C59	trypsin	IV	XI	IX	Mus musculus	3.4.21.-
Q1JRP2	trypsin	IV	XI	IX	Mus musculus	3.4.21.-
Q3UQ41	trypsin	IV	XI	IX	Mus musculus	3.4.21.-
Q5S248	trypsin	IV	XI	IX	Mus musculus	3.4.21.-
Q8BHM9	trypsin	IV	XI	IX	Mus musculus	3.4.21.-
Q8BZ10	trypsin	IV	XI	IX	Mus musculus	3.4.21.-
Q9ER04	trypsin	IV	XI	IX	Mus musculus	3.4.21.-
Q9R098	trypsin	IV	XI	IX	Mus musculus	3.4.21.-
O35453	trypsin	IV	XI	IX	Mus musculus	3.4.21.106
Q80YC5	trypsin	IV	XI	IX	Mus musculus	3.4.21.38
P06869	trypsin	IV	XI	IX	Mus musculus	3.4.21.73
A7RP61	trypsin	IV	XI	IX	Nematostella vectensis	

Table S2.4. (continuation)

UniProt	Label [1]	Four	Eleven	Twelve	Species	EC Number
A7SSS0	trypsin	IV	XI	IX	Nematostella vectensis	
Q8MHY7	trypsin	IV	XI	IX	Oryctolagus cuniculus	3.4.21.73
P16227	trypsin	IV	XI	IX	Papio cynocephalus	3.4.21.73
Q9Y1V3	trypsin	IV	XI	IX	Polyandrocarpa misakiensis	
Q5R5E8	trypsin	IV	XI	IX	Pongo abelii	3.4.21.106
Q5RF29	trypsin	IV	XI	IX	Pongo abelii	3.4.21.73
Q0X0F0	trypsin	IV	XI	IX	Rattus norvegicus	
Q3KR76	trypsin	IV	XI	IX	Rattus norvegicus	
Q5EBA7	trypsin	IV	XI	IX	Rattus norvegicus	
Q8CJ16	trypsin	IV	XI	IX	Rattus norvegicus	
Q8CJ17	trypsin	IV	XI	IX	Rattus norvegicus	
Q5QSK2	trypsin	IV	XI	IX	Rattus norvegicus	3.4.21.-
Q6IE14	trypsin	IV	XI	IX	Rattus norvegicus	3.4.21.-
Q6IE15	trypsin	IV	XI	IX	Rattus norvegicus	3.4.21.-
Q8VHJ4	trypsin	IV	XI	IX	Rattus norvegicus	3.4.21.-
Q05511	trypsin	IV	XI	IX	Rattus norvegicus	3.4.21.106
P29598	trypsin	IV	XI	IX	Rattus norvegicus	3.4.21.73
O88781	trypsin	IV	XI	IX	Rattus rattus	
A4F927	trypsin	IV	XI	IX	Saccharopolyspora erythraea	
Q54137	trypsin	IV	XI	IX	Saccharopolyspora erythraea	
P24664	trypsin	IV	XI	IX	Saccharopolyspora erythraea	3.4.21.4
Q6I695	trypsin	IV	XI	IX	Streptomyces griseus	
P04185	trypsin	IV	XI	IX	Sus scrofa	3.4.21.73
Q4RG83	trypsin	IV	XI	IX	Tetraodon nigroviridis	
Q4RQD7	trypsin	IV	XI	IX	Tetraodon nigroviridis	
Q8JIS1	trypsin	IV	XI	IX	Triakis scyllium	
Q03711	trypsin	IV	XI	IX	Xenopus laevis	
Q7ZTR2	trypsin	IV	XI	IX	Xenopus laevis	
Q28DA4	trypsin	IV	XI	IX	Xenopus tropicalis	
Q4V785	trypsin	IV	XI	IX	Xenopus tropicalis	
Q5FVX1	trypsin	IV	XI	IX	Xenopus tropicalis	
Q7PXG5	trypsin	IV	XI	VIII	Anopheles gambiae	
Q95VT4	trypsin	IV	XI	VIII	Homarus americanus	
Q16ID2	trypsin	IV	XI	XI	Aedes aegypti	
Q174G2	trypsin	IV	XI	XI	Aedes aegypti	
Q17HM6	trypsin	IV	XI	XI	Aedes aegypti	
Q7PZ84	trypsin	IV	XI	XI	Anopheles gambiae	
Q00344	trypsin	IV	XI	XI	Cochliobolus carbonum	
Q7RTY9	trypsin	IV	XI	XI	Homo sapiens	3.4.21.-
A4UCN5	trypsin	IV	XI	XI	Lutzomyia longipalpis	
Q9U0G3	trypsin	IV	XI	XI	Pacifastacus leniusculus	
Q0UQI6	trypsin	IV	XI	XI	Phaeosphaeria nodorum	
Q5I8R5	trypsin	IV	XI	XI	Zoophthora radicans	
Q0IF78	trypsin	IV	XI	XII	Aedes aegypti	
Q0IF79	trypsin	IV	XI	XII	Aedes aegypti	
Q0IF80	trypsin	IV	XI	XII	Aedes aegypti	
Q0IF81	trypsin	IV	XI	XII	Aedes aegypti	
Q0IF84	trypsin	IV	XI	XII	Aedes aegypti	
Q0IF85	trypsin	IV	XI	XII	Aedes aegypti	
Q16ID3	trypsin	IV	XI	XII	Aedes aegypti	
Q16ID4	trypsin	IV	XI	XII	Aedes aegypti	
Q16KW4	trypsin	IV	XI	XII	Aedes aegypti	
Q16PR8	trypsin	IV	XI	XII	Aedes aegypti	
Q16SA2	trypsin	IV	XI	XII	Aedes aegypti	
Q171L4	trypsin	IV	XI	XII	Aedes aegypti	
Q171W4	trypsin	IV	XI	XII	Aedes aegypti	

Table S2.4. (continuation)

UniProt	Label [1]	Four	Eleven	Twelve	Species	EC Number
Q176G7	trypsin	IV	XI	XII	Aedes aegypti	
Q179I8	trypsin	IV	XI	XII	Aedes aegypti	
Q179I9	trypsin	IV	XI	XII	Aedes aegypti	
Q17J66	trypsin	IV	XI	XII	Aedes aegypti	
Q17KI3	trypsin	IV	XI	XII	Aedes aegypti	
Q17PX9	trypsin	IV	XI	XII	Aedes aegypti	
Q17PY0	trypsin	IV	XI	XII	Aedes aegypti	
Q9NB92	trypsin	IV	XI	XII	Agrotis ipsilon	
A0NFQ3	trypsin	IV	XI	XII	Anopheles gambiae	
A0NH77	trypsin	IV	XI	XII	Anopheles gambiae	
A7UVQ7	trypsin	IV	XI	XII	Anopheles gambiae	
Q17035	trypsin	IV	XI	XII	Anopheles gambiae	
Q7PQB3	trypsin	IV	XI	XII	Anopheles gambiae	
Q7PUB9	trypsin	IV	XI	XII	Anopheles gambiae	
Q7Q2Q8	trypsin	IV	XI	XII	Anopheles gambiae	
Q7Q344	trypsin	IV	XI	XII	Anopheles gambiae	
Q7Q554	trypsin	IV	XI	XII	Anopheles gambiae	
Q7QCV2	trypsin	IV	XI	XII	Anopheles gambiae	
Q7QGL1	trypsin	IV	XI	XII	Anopheles gambiae	
Q7QIH5	trypsin	IV	XI	XII	Anopheles gambiae	
Q7QIM7	trypsin	IV	XI	XII	Anopheles gambiae	
Q7QJ48	trypsin	IV	XI	XII	Anopheles gambiae	
P35035	trypsin	IV	XI	XII	Anopheles gambiae	3.4.21.4
P35036	trypsin	IV	XI	XII	Anopheles gambiae	3.4.21.4
P35037	trypsin	IV	XI	XII	Anopheles gambiae	3.4.21.4
P35038	trypsin	IV	XI	XII	Anopheles gambiae	3.4.21.4
P35039	trypsin	IV	XI	XII	Anopheles gambiae	3.4.21.4
P35041	trypsin	IV	XI	XII	Anopheles gambiae	3.4.21.4
O16133	trypsin	IV	XI	XII	Anopheles stephensi	
Q17086	trypsin	IV	XI	XII	Anopheles stephensi	
Q9GQ03	trypsin	IV	XI	XII	Biomphalaria glabrata	
A1KXI1	trypsin	IV	XI	XII	Blomia tropicalis	
Q8I916	trypsin	IV	XI	XII	Blomia tropicalis	
Q1HPZ8	trypsin	IV	XI	XII	Bombyx mori	
Q45RG0	trypsin	IV	XI	XII	Bombyx mori	
Q589Y5	trypsin	IV	XI	XII	Bombyx mori	
Q8WSJ2	trypsin	IV	XI	XII	Bombyx mori	
Q07943	trypsin	IV	XI	XII	Bombyx mori	3.4.21.-
A7E330	trypsin	IV	XI	XII	Bos taurus	
Q08D90	trypsin	IV	XI	XII	Bos taurus	
Q08DU0	trypsin	IV	XI	XII	Bos taurus	
Q32PI4	trypsin	IV	XI	XII	Bos taurus	
Q0IIH7	trypsin	IV	XI	XII	Bos taurus	3.4.21.109
P98140	trypsin	IV	XI	XII	Bos taurus	3.4.21.38
Q04962	trypsin	IV	XI	XII	Cavia porcellus	3.4.21.38
Q9XY60	trypsin	IV	XI	XII	Ctenocephalides felis	
Q56GM3	trypsin	IV	XI	XII	Culex pipiens	
Q962G7	trypsin	IV	XI	XII	Culex pipiens	
O02569	trypsin	IV	XI	XII	Culex quinquefasciatus	
A0JMD7	trypsin	IV	XI	XII	Danio rerio	
A2BGB9	trypsin	IV	XI	XII	Danio rerio	
A5PLB6	trypsin	IV	XI	XII	Danio rerio	
A1A5H5	trypsin	IV	XI	XII	Danio rerio	3.4.21.109
A5PMY0	trypsin	IV	XI	XII	Danio rerio	3.4.21.109
Q1RLP8	trypsin	IV	XI	XII	Danio rerio	3.4.21.109
P54624	trypsin	IV	XI	XII	Drosophila erecta	3.4.21.4

Table S2.4. (continuation)

UniProt	Label [1]	Four	Eleven	Twelve	Species	EC Number
P54625	trypsin	IV	XI	XII	Drosophila erecta	3.4.21.4
P54626	trypsin	IV	XI	XII	Drosophila erecta	3.4.21.4
P54627	trypsin	IV	XI	XII	Drosophila erecta	3.4.21.4
P54628	trypsin	IV	XI	XII	Drosophila erecta	3.4.21.4
P54629	trypsin	IV	XI	XII	Drosophila erecta	3.4.21.4
P54630	trypsin	IV	XI	XII	Drosophila erecta	3.4.21.4
A0JQ46	trypsin	IV	XI	XII	Drosophila melanogaster	
A8E6I8	trypsin	IV	XI	XII	Drosophila melanogaster	
Q24019	trypsin	IV	XI	XII	Drosophila melanogaster	
Q494H7	trypsin	IV	XI	XII	Drosophila melanogaster	
Q960I5	trypsin	IV	XI	XII	Drosophila melanogaster	
Q9VZH2	trypsin	IV	XI	XII	Drosophila melanogaster	3.4.-.-; 3.4.21.-
Q7JPN9	trypsin	IV	XI	XII	Drosophila melanogaster	3.4.21.-; 3.4.21.4
A1Z709	trypsin	IV	XI	XII	Drosophila melanogaster	3.4.21.-
A1Z8J8	trypsin	IV	XI	XII	Drosophila melanogaster	3.4.21.-
A1ZA64	trypsin	IV	XI	XII	Drosophila melanogaster	3.4.21.-
P98159	trypsin	IV	XI	XII	Drosophila melanogaster	3.4.21.-
Q0E8E2	trypsin	IV	XI	XII	Drosophila melanogaster	3.4.21.-
Q4V3S6	trypsin	IV	XI	XII	Drosophila melanogaster	3.4.21.-
Q4V5J3	trypsin	IV	XI	XII	Drosophila melanogaster	3.4.21.-
Q7KVM3	trypsin	IV	XI	XII	Drosophila melanogaster	3.4.21.-
Q8IQ10	trypsin	IV	XI	XII	Drosophila melanogaster	3.4.21.-
Q8IRE0	trypsin	IV	XI	XII	Drosophila melanogaster	3.4.21.-
Q8IRE2	trypsin	IV	XI	XII	Drosophila melanogaster	3.4.21.-
Q8IRR3	trypsin	IV	XI	XII	Drosophila melanogaster	3.4.21.-
Q9VAG3	trypsin	IV	XI	XII	Drosophila melanogaster	3.4.21.-
Q9VV38	trypsin	IV	XI	XII	Drosophila melanogaster	3.4.21.-
Q9W453	trypsin	IV	XI	XII	Drosophila melanogaster	3.4.21.-
Q9W454	trypsin	IV	XI	XII	Drosophila melanogaster	3.4.21.-
P04814	trypsin	IV	XI	XII	Drosophila melanogaster	3.4.21.4
P35005	trypsin	IV	XI	XII	Drosophila melanogaster	3.4.21.4
P42276	trypsin	IV	XI	XII	Drosophila melanogaster	3.4.21.4
P42278	trypsin	IV	XI	XII	Drosophila melanogaster	3.4.21.4
P42279	trypsin	IV	XI	XII	Drosophila melanogaster	3.4.21.4
P42280	trypsin	IV	XI	XII	Drosophila melanogaster	3.4.21.4
P52905	trypsin	IV	XI	XII	Drosophila melanogaster	3.4.21.4
Q28XQ4	trypsin	IV	XI	XII	Drosophila pseudoobscura	3.4.21.-
Q28Y20	trypsin	IV	XI	XII	Drosophila pseudoobscura	3.4.21.-
Q28Y22	trypsin	IV	XI	XII	Drosophila pseudoobscura	3.4.21.-
Q28Y23	trypsin	IV	XI	XII	Drosophila pseudoobscura	3.4.21.-
Q28Y24	trypsin	IV	XI	XII	Drosophila pseudoobscura	3.4.21.-
Q28Y25	trypsin	IV	XI	XII	Drosophila pseudoobscura	3.4.21.-
Q28Y26	trypsin	IV	XI	XII	Drosophila pseudoobscura	3.4.21.-
Q28Y27	trypsin	IV	XI	XII	Drosophila pseudoobscura	3.4.21.-
Q28Y91	trypsin	IV	XI	XII	Drosophila pseudoobscura	3.4.21.-
Q28YJ3	trypsin	IV	XI	XII	Drosophila pseudoobscura	3.4.21.-
Q29C14	trypsin	IV	XI	XII	Drosophila pseudoobscura	3.4.21.-
Q29D77	trypsin	IV	XI	XII	Drosophila pseudoobscura	3.4.21.-
Q29DR0	trypsin	IV	XI	XII	Drosophila pseudoobscura	3.4.21.-
Q29EK0	trypsin	IV	XI	XII	Drosophila pseudoobscura	3.4.21.-
Q29H51	trypsin	IV	XI	XII	Drosophila pseudoobscura	3.4.21.-
Q29MC6	trypsin	IV	XI	XII	Drosophila pseudoobscura	3.4.21.-
Q2M0F5	trypsin	IV	XI	XII	Drosophila pseudoobscura	3.4.21.-
O18600	trypsin	IV	XI	XII	Drosophila virilis	
O18599	trypsin	IV	XI	XII	Drosophila virilis	3.4.21.-
Q27J28	trypsin	IV	XI	XII	Eupolyphaga sinensis	

Table S2.4. (continuation)

UniProt	Label [1]	Four	Eleven	Twelve	Species	EC Number
P15120	trypsin	IV	XI	XII	Gallus gallus	3.4.21.73
Q0Q605	trypsin	IV	XI	XII	Gryllus firmus	
Q0ZBW0	trypsin	IV	XI	XII	Gryllus firmus	
Q0ZBV9	trypsin	IV	XI	XII	Gryllus pennsylvanicus	
Q0ZBV8	trypsin	IV	XI	XII	Gryllus veletis	
Q6LAM0	trypsin	IV	XI	XII	Homo sapiens	
A1L453	trypsin	IV	XI	XII	Homo sapiens	3.4.21.-
Q16651	trypsin	IV	XI	XII	Homo sapiens	3.4.21.-
Q86T26	trypsin	IV	XI	XII	Homo sapiens	3.4.21.-
Q8IU80	trypsin	IV	XI	XII	Homo sapiens	3.4.21.-
Q8NF86	trypsin	IV	XI	XII	Homo sapiens	3.4.21.-
Q9NRR2	trypsin	IV	XI	XII	Homo sapiens	3.4.21.-
Q9Y6M0	trypsin	IV	XI	XII	Homo sapiens	3.4.21.-
P49862	trypsin	IV	XI	XII	Homo sapiens	3.4.21.117
P05156	trypsin	IV	XI	XII	Homo sapiens	3.4.21.45
Q25081	trypsin	IV	XI	XII	Hypoderma lineatum	
P35588	trypsin	IV	XI	XII	Hypoderma lineatum	3.4.21.-
Q6WGR1	trypsin	IV	XI	XII	Ictalurus punctatus	
Q7ZT70	trypsin	IV	XI	XII	Lampetra japonica	
A4UCN4	trypsin	IV	XI	XII	Lutzomyia longipalpis	
A8CW51	trypsin	IV	XI	XII	Lutzomyia longipalpis	
A8CW70	trypsin	IV	XI	XII	Lutzomyia longipalpis	
O44330	trypsin	IV	XI	XII	Manduca sexta	
A5CG75	trypsin	IV	XI	XII	Manduca sexta	3.4.21.1
Q5IS29	trypsin	IV	XI	XII	Mayetiola destructor	
Q5ISB8	trypsin	IV	XI	XII	Mayetiola destructor	
Q5IY42	trypsin	IV	XI	XII	Mayetiola destructor	
Q01136	trypsin	IV	XI	XII	Metarhizium anisopliae	
Q9Y842	trypsin	IV	XI	XII	Metarhizium anisopliae	
Q3V068	trypsin	IV	XI	XII	Mus musculus	
Q54AE4	trypsin	IV	XI	XII	Mus musculus	
Q80X17	trypsin	IV	XI	XII	Mus musculus	
Q80YD8	trypsin	IV	XI	XII	Mus musculus	
Q8BJV6	trypsin	IV	XI	XII	Mus musculus	
Q8R1A6	trypsin	IV	XI	XII	Mus musculus	
Q99L44	trypsin	IV	XI	XII	Mus musculus	
Q402U7	trypsin	IV	XI	XII	Mus musculus	3.4.21.-
Q80WM7	trypsin	IV	XI	XII	Mus musculus	3.4.21.-
Q8VHK8	trypsin	IV	XI	XII	Mus musculus	3.4.21.-
Q8VIF2	trypsin	IV	XI	XII	Mus musculus	3.4.21.-
Q9D9M0	trypsin	IV	XI	XII	Mus musculus	3.4.21.-
Q9DBI0	trypsin	IV	XI	XII	Mus musculus	3.4.21.-
Q9ESD1	trypsin	IV	XI	XII	Mus musculus	3.4.21.-
Q9JHJ7	trypsin	IV	XI	XII	Mus musculus	3.4.21.-
Q9QUL7	trypsin	IV	XI	XII	Mus musculus	3.4.21.-
Q61129	trypsin	IV	XI	XII	Mus musculus	3.4.21.45
A7SZ55	trypsin	IV	XI	XII	Nematostella vectensis	
A7SZI9	trypsin	IV	XI	XII	Nematostella vectensis	
Q0ZP54	trypsin	IV	XI	XII	Neodiprion abietis	
Q6JPG5	trypsin	IV	XI	XII	Neodiprion lecontei	
Q6JKF3	trypsin	IV	XI	XII	Neodiprion sertifer	
Q50LG7	trypsin	IV	XI	XII	Oryzias latipes	3.4.21.68
A4S4W9	trypsin	IV	XI	XII	Ostreococcus lucimarinus	
Q6R560	trypsin	IV	XI	XII	Ostrinia nubilalis	
Q4VSI1	trypsin	IV	XI	XII	Pediculus humanus	
Q4VSI2	trypsin	IV	XI	XII	Pediculus humanus	

Table S2.4. (continuation)

UniProt	Label [1]	Four	Eleven	Twelve	Species	EC Number
Q1M0X9	trypsin	IV	XI	XII	Periplaneta americana	
Q7Z0G1	trypsin	IV	XI	XII	Phlebotomus papatasi	
Q7Z0G2	trypsin	IV	XI	XII	Phlebotomus papatasi	
Q5RBI2	trypsin	IV	XI	XII	Pongo abelii	
Q5RCT3	trypsin	IV	XI	XII	Pongo abelii	
Q5R5A4	trypsin	IV	XI	XII	Pongo abelii	3.4.21.45
Q80XZ3	trypsin	IV	XI	XII	Rattus norvegicus	
Q80Z40	trypsin	IV	XI	XII	Rattus norvegicus	
Q9ES87	trypsin	IV	XI	XII	Rattus norvegicus	3.4.21.-
Q9WUW3	trypsin	IV	XI	XII	Rattus norvegicus	3.4.21.45
P32038	trypsin	IV	XI	XII	Rattus norvegicus	3.4.21.46
A4FM74	trypsin	IV	XI	XII	Saccharopolyspora erythraea	
A4FM78	trypsin	IV	XI	XII	Saccharopolyspora erythraea	
P51588	trypsin	IV	XI	XII	Sarcophaga bullata	3.4.21.4
Q4L1K1	trypsin	IV	XI	XII	Sesamia nonagrioides	3.4.21.4
Q4L1L0	trypsin	IV	XI	XII	Sesamia nonagrioides	3.4.21.4
Q4L1L1	trypsin	IV	XI	XII	Sesamia nonagrioides	3.4.21.4
Q4L1L3	trypsin	IV	XI	XII	Sesamia nonagrioides	3.4.21.4
O76520	trypsin	IV	XI	XII	Stomoxys calcitrans	
Q82KG0	trypsin	IV	XI	XII	Streptomyces avermitilis	
O69997	trypsin	IV	XI	XII	Streptomyces coelicolor	
Q54168	trypsin	IV	XI	XII	Streptomyces fradiae	
O97507	trypsin	IV	XI	XII	Sus scrofa	3.4.21.38
A1XG55	trypsin	IV	XI	XII	Tenebrio molitor	
A1XG56	trypsin	IV	XI	XII	Tenebrio molitor	
A1XG57	trypsin	IV	XI	XII	Tenebrio molitor	
A1XG58	trypsin	IV	XI	XII	Tenebrio molitor	
Q4RHT0	trypsin	IV	XI	XII	Tetraodon nigroviridis	
Q4S6B0	trypsin	IV	XI	XII	Tetraodon nigroviridis	
Q4TBY8	trypsin	IV	XI	XII	Tetraodon nigroviridis	
A1L3H8	trypsin	IV	XI	XII	Xenopus laevis	
O42272	trypsin	IV	XI	XII	Xenopus laevis	
Q2VPP0	trypsin	IV	XI	XII	Xenopus laevis	
Q5XK88	trypsin	IV	XI	XII	Xenopus laevis	
Q6IRA4	trypsin	IV	XI	XII	Xenopus laevis	
Q7SY86	trypsin	IV	XI	XII	Xenopus laevis	
Q7T0X2	trypsin	IV	XI	XII	Xenopus laevis	
Q9PVX7	trypsin	IV	XI	XII	Xenopus laevis	
Q63ZQ6	trypsin	IV	XI	XII	Xenopus laevis	3.4.21.109
Q6GR54	trypsin	IV	XI	XII	Xenopus laevis	3.4.21.109
Q9DGR1	trypsin	IV	XI	XII	Xenopus laevis	3.4.21.109
Q6P7I9	trypsin	IV	XI	XII	Xenopus laevis	3.4.21.68
Q28EB0	trypsin	IV	XI	XII	Xenopus tropicalis	
Q566K9	trypsin	IV	XI	XII	Xenopus tropicalis	
Q6NVR7	trypsin	IV	XI	XII	Xenopus tropicalis	
Q5BKN3	trypsin	IV	XI	XII	Xenopus tropicalis	3.4.21.68
Q1PAE8	trypsin	IV	XI	XII	Zabrotes subfasciatus	

Case study V: crotonases

After the filtering process, there remained 2,694 proteins in this SFLD [3] superfamily, all from the same subgroup and distributed among twelve families. Table S2.5 lists the protein set, along with the SFLD family labels and the clusters they were placed in by the proposed framework when considering twelve clusters, as well as the source organisms and existing EC number annotations according to UniProt.

Table S2.5. List of proteins in the crotonase superfamily.

UniProt	Family [3]	Cluster	Species	EC Number
Q6FBA0	1,2-epoxyphenylacetyl-CoA isomerase	VI	Acinetobacter baylyi	4.2.1.17
D0SWC2	1,2-epoxyphenylacetyl-CoA isomerase	VI	Acinetobacter lwoffii	
C6WKZ3	1,2-epoxyphenylacetyl-CoA isomerase	VI	Actinosynnema mirum	
I3YW92	1,2-epoxyphenylacetyl-CoA isomerase	VI	Aequorivita sublithincola	
F7UDG1	1,2-epoxyphenylacetyl-CoA isomerase	VI	Agrobacterium tumefaciens	
B7DMV5	1,2-epoxyphenylacetyl-CoA isomerase	VI	Alicyclobacillus acidocaldarius	
F8IEY7	1,2-epoxyphenylacetyl-CoA isomerase	VI	Alicyclobacillus acidocaldarius	
G7ZH09	1,2-epoxyphenylacetyl-CoA isomerase	VI	Azospirillum lipoferum	4.2.1.17
U5L9T9	1,2-epoxyphenylacetyl-CoA isomerase	VI	Bacillus infantis	
I8AJU6	1,2-epoxyphenylacetyl-CoA isomerase	VI	Bacillus macauensis	
D5DFP7	1,2-epoxyphenylacetyl-CoA isomerase	VI	Bacillus megaterium	4.2.1.17
D5DS24	1,2-epoxyphenylacetyl-CoA isomerase	VI	Bacillus megaterium	4.2.1.17
G9QMY0	1,2-epoxyphenylacetyl-CoA isomerase	VI	Bacillus smithii	
F7QNF8	1,2-epoxyphenylacetyl-CoA isomerase	VI	Bradyrhizobiaceae bacterium	4.2.1.17
C0Z572	1,2-epoxyphenylacetyl-CoA isomerase	VI	Brevibacillus brevis	4.2.1.17
J2G9L8	1,2-epoxyphenylacetyl-CoA isomerase	VI	Brevibacillus sp.	
J2HJN2	1,2-epoxyphenylacetyl-CoA isomerase	VI	Brevibacillus sp.	
F5L6I8	1,2-epoxyphenylacetyl-CoA isomerase	VI	Caldalkalibacillus thermarum	
F5L9G6	1,2-epoxyphenylacetyl-CoA isomerase	VI	Caldalkalibacillus thermarum	
Q3ABC8	1,2-epoxyphenylacetyl-CoA isomerase	VI	Carboxydothermus hydrog.	
D0D2A1	1,2-epoxyphenylacetyl-CoA isomerase	VI	Citreicella sp.	4.2.1.17
A3U7D4	1,2-epoxyphenylacetyl-CoA isomerase	VI	Croceibacter atlanticus	
H1S1R0	1,2-epoxyphenylacetyl-CoA isomerase	VI	Cupriavidus basilensis	
F8GWU3	1,2-epoxyphenylacetyl-CoA isomerase	VI	Cupriavidus necator	4.2.1.17
G0EZZ9	1,2-epoxyphenylacetyl-CoA isomerase	VI	Cupriavidus necator	4.2.1.17
B3R7H1	1,2-epoxyphenylacetyl-CoA isomerase	VI	Cupriavidus taiwanensis	4.2.1.17
Q9RRI1	1,2-epoxyphenylacetyl-CoA isomerase	VI	Deinococcus radiodurans	
F5SA74	1,2-epoxyphenylacetyl-CoA isomerase	VI	Desmospora sp.	4.2.1.55
F5SJL1	1,2-epoxyphenylacetyl-CoA isomerase	VI	Desmospora sp.	4.2.1.55
R4KEG6	1,2-epoxyphenylacetyl-CoA isomerase	VI	Desulfotomaculum gibsoniae	
M8JZ97	1,2-epoxyphenylacetyl-CoA isomerase	VI	Escherichia coli	4.2.1.17
P77467	1,2-epoxyphenylacetyl-CoA isomerase	VI	Escherichia coli	5.3.3.18
B1YKP5	1,2-epoxyphenylacetyl-CoA isomerase	VI	Exiguobacterium sibiricum	
Q26EY7	1,2-epoxyphenylacetyl-CoA isomerase	VI	Flavobacteria bacterium	
A8UE13	1,2-epoxyphenylacetyl-CoA isomerase	VI	Flavobacteriales bacterium	
F2IDT2	1,2-epoxyphenylacetyl-CoA isomerase	VI	Fluviicola taffensis	4.2.1.17
I2JMA4	1,2-epoxyphenylacetyl-CoA isomerase	VI	gamma proteobacterium	4.2.1.17
C1A789	1,2-epoxyphenylacetyl-CoA isomerase	VI	Gemmatimonas aurantiaca	4.2.1.17
F4AK31	1,2-epoxyphenylacetyl-CoA isomerase	VI	Glaciecola sp.	
F7SMV1	1,2-epoxyphenylacetyl-CoA isomerase	VI	Halomonas sp.	4.2.1.17
D8ITH1	1,2-epoxyphenylacetyl-CoA isomerase	VI	Herbaspirillum seropedicae	4.2.1.17
J3DFH0	1,2-epoxyphenylacetyl-CoA isomerase	VI	Herbaspirillum sp.	
Q28QE1	1,2-epoxyphenylacetyl-CoA isomerase	VI	Jannaschia sp.	4.2.1.17
A9DLU8	1,2-epoxyphenylacetyl-CoA isomerase	VI	Kordia algicida	
D6TJD3	1,2-epoxyphenylacetyl-CoA isomerase	VI	Ktedonobacter racemifer	
D5WUY1	1,2-epoxyphenylacetyl-CoA isomerase	VI	Kyrpidia tusciae	
D5WXP5	1,2-epoxyphenylacetyl-CoA isomerase	VI	Kyrpidia tusciae	
A0NQQ8	1,2-epoxyphenylacetyl-CoA isomerase	VI	Labrenzia aggregata	4.2.1.17
B9R4H9	1,2-epoxyphenylacetyl-CoA isomerase	VI	Labrenzia alexandrii	4.2.1.17
B8KT84	1,2-epoxyphenylacetyl-CoA isomerase	VI	Luminiphilus syltensis	4.2.1.17
A4TVC1	1,2-epoxyphenylacetyl-CoA isomerase	VI	Magnetospirillum gryph.	
F2NK49	1,2-epoxyphenylacetyl-CoA isomerase	VI	Marinithermus hydrothermalis	
E4PKI0	1,2-epoxyphenylacetyl-CoA isomerase	VI	Marinobacter adhaerens	
M1FG40	1,2-epoxyphenylacetyl-CoA isomerase	VI	Marinobacter sp.	
A6VZY1	1,2-epoxyphenylacetyl-CoA isomerase	VI	Marinomonas sp.	
A3YFA9	1,2-epoxyphenylacetyl-CoA isomerase	VI	Marinomonas sp.	4.2.1.17

Table S2.5. (continuation)

UniProt	Family [3]	Cluster	Species	EC Number
A3VLM6	1,2-epoxyphenylacetyl-CoA isomerase	VI	Maritimibacter alkaliphilus	
E4TP43	1,2-epoxyphenylacetyl-CoA isomerase	VI	Marivirga tractuosa	
D3PSX3	1,2-epoxyphenylacetyl-CoA isomerase	VI	Meiothermus ruber	
D7BFN7	1,2-epoxyphenylacetyl-CoA isomerase	VI	Meiothermus silvanus	
B8IP48	1,2-epoxyphenylacetyl-CoA isomerase	VI	Methylobacterium nodulans	
I4EQ52	1,2-epoxyphenylacetyl-CoA isomerase	VI	Modestobacter marinus	4.2.1.17
Q1QPQ2	1,2-epoxyphenylacetyl-CoA isomerase	VI	Nitrobacter hamburgensis	4.2.1.17
A4XEE6	1,2-epoxyphenylacetyl-CoA isomerase	VI	Novosphingobium aromat.	
A3U2W9	1,2-epoxyphenylacetyl-CoA isomerase	VI	Oceanicola batsensis	
Q2CG26	1,2-epoxyphenylacetyl-CoA isomerase	VI	Oceanicola granulosus	
E4U8E2	1,2-epoxyphenylacetyl-CoA isomerase	VI	Oceanithermus profundus	4.2.1.17
A1BBG6	1,2-epoxyphenylacetyl-CoA isomerase	VI	Paracoccus denitrificans	4.2.1.17
A5D469	1,2-epoxyphenylacetyl-CoA isomerase	VI	Pelotomaculum thermo.	
B4RH05	1,2-epoxyphenylacetyl-CoA isomerase	VI	Phenylobacterium zucineum	
F2IYW5	1,2-epoxyphenylacetyl-CoA isomerase	VI	Polymorphum gilvum	
J1FN53	1,2-epoxyphenylacetyl-CoA isomerase	VI	Pontibacter sp.	
B2PUH6	1,2-epoxyphenylacetyl-CoA isomerase	VI	Providencia stuartii	4.2.1.17
Q1I9V3	1,2-epoxyphenylacetyl-CoA isomerase	VI	Pseudomonas entomophila	4.2.1.17
Q4KBZ7	1,2-epoxyphenylacetyl-CoA isomerase	VI	Pseudomonas fluorescens	4.2.1.17
F4DZ63	1,2-epoxyphenylacetyl-CoA isomerase	VI	Pseudomonas mendocina	
B1J8Q5	1,2-epoxyphenylacetyl-CoA isomerase	VI	Pseudomonas putida	
A0A0A7Q134	1,2-epoxyphenylacetyl-CoA isomerase	VI	Pseudomonas putida	4.2.1.17
J2RLF7	1,2-epoxyphenylacetyl-CoA isomerase	VI	Pseudomonas sp.	
J2SN96	1,2-epoxyphenylacetyl-CoA isomerase	VI	Pseudomonas sp.	
J2UTV7	1,2-epoxyphenylacetyl-CoA isomerase	VI	Pseudomonas sp.	
J2Y0E4	1,2-epoxyphenylacetyl-CoA isomerase	VI	Pseudomonas sp.	
J2Y3K4	1,2-epoxyphenylacetyl-CoA isomerase	VI	Pseudomonas sp.	
Q70IM8	1,2-epoxyphenylacetyl-CoA isomerase	VI	Pseudomonas sp.	
I4MZX6	1,2-epoxyphenylacetyl-CoA isomerase	VI	Pseudomonas sp.	4.2.1.17
V7D5P6	1,2-epoxyphenylacetyl-CoA isomerase	VI	Pseudomonas taiwanensis	4.2.1.17
F4CJE7	1,2-epoxyphenylacetyl-CoA isomerase	VI	Pseudonocardia dioxanivorans	
B6R3F3	1,2-epoxyphenylacetyl-CoA isomerase	VI	Pseudovibrio sp.	
G8PJN5	1,2-epoxyphenylacetyl-CoA isomerase	VI	Pseudovibrio sp.	
B6R4H5	1,2-epoxyphenylacetyl-CoA isomerase	VI	Pseudovibrio sp.	4.2.1.17
K4IFC9	1,2-epoxyphenylacetyl-CoA isomerase	VI	Psychroflexus torquis	
H6Q8Z9	1,2-epoxyphenylacetyl-CoA isomerase	VI	Pyrobaculum oguniense	4.2.1.17
H6QDS6	1,2-epoxyphenylacetyl-CoA isomerase	VI	Pyrobaculum oguniense	4.2.1.17
R7XRI3	1,2-epoxyphenylacetyl-CoA isomerase	VI	Ralstonia sp.	
G9A9N4	1,2-epoxyphenylacetyl-CoA isomerase	VI	Rhizobium fredii	4.2.1.17
J2I554	1,2-epoxyphenylacetyl-CoA isomerase	VI	Rhizobium sp.	
A3JSB9	1,2-epoxyphenylacetyl-CoA isomerase	VI	Rhodobacteraceae bacterium	
B6AVE8	1,2-epoxyphenylacetyl-CoA isomerase	VI	Rhodobacteraceae bacterium	
B9NSA6	1,2-epoxyphenylacetyl-CoA isomerase	VI	Rhodobacteraceae bacterium	
B6BER4	1,2-epoxyphenylacetyl-CoA isomerase	VI	Rhodobacterales bacterium	
B3QC39	1,2-epoxyphenylacetyl-CoA isomerase	VI	Rhodopseudomonas palustris	
E6VQ53	1,2-epoxyphenylacetyl-CoA isomerase	VI	Rhodopseudomonas palustris	
Q132F9	1,2-epoxyphenylacetyl-CoA isomerase	VI	Rhodopseudomonas palustris	4.2.1.17
Q2ISP4	1,2-epoxyphenylacetyl-CoA isomerase	VI	Rhodopseudomonas palustris	4.2.1.17
G2SH89	1,2-epoxyphenylacetyl-CoA isomerase	VI	Rhodothermus marinus	
Q163X0	1,2-epoxyphenylacetyl-CoA isomerase	VI	Roseobacter denitrificans	4.2.1.-
A4EKF3	1,2-epoxyphenylacetyl-CoA isomerase	VI	Roseobacter sp.	
A4ERT6	1,2-epoxyphenylacetyl-CoA isomerase	VI	Roseobacter sp.	
A6FRH8	1,2-epoxyphenylacetyl-CoA isomerase	VI	Roseobacter sp.	
B7RPS2	1,2-epoxyphenylacetyl-CoA isomerase	VI	Roseobacter sp.	
A3VXF0	1,2-epoxyphenylacetyl-CoA isomerase	VI	Roseovarius sp.	
Q5LS86	1,2-epoxyphenylacetyl-CoA isomerase	VI	Ruegeria pomeroyi	

Table S2.5. (continuation)

UniProt	Family [3]	Cluster	Species	EC Number
B7QRR4	1,2-epoxyphenylacetyl-CoA isomerase	VI	Ruegeria sp.	
Q1GHK3	1,2-epoxyphenylacetyl-CoA isomerase	VI	Ruegeria sp.	4.2.1.17
A3K6J2	1,2-epoxyphenylacetyl-CoA isomerase	VI	Sagittula stellata	
J0Y102	1,2-epoxyphenylacetyl-CoA isomerase	VI	Saprospira grandis	
B1KMB2	1,2-epoxyphenylacetyl-CoA isomerase	VI	Shewanella woodyi	
A6UH29	1,2-epoxyphenylacetyl-CoA isomerase	VI	Sinorhizobium medicae	
D1C849	1,2-epoxyphenylacetyl-CoA isomerase	VI	Sphaerobacter thermophilus	
D4Z8D4	1,2-epoxyphenylacetyl-CoA isomerase	VI	Sphingobium japonicum	4.2.1.17
A0A0A1YCZ5	1,2-epoxyphenylacetyl-CoA isomerase	VI	Sphingomonas sp.	
Q1NAB7	1,2-epoxyphenylacetyl-CoA isomerase	VI	Sphingomonas sp.	
A5V7U3	1,2-epoxyphenylacetyl-CoA isomerase	VI	Sphingomonas wittichii	
B4V170	1,2-epoxyphenylacetyl-CoA isomerase	VI	Streptomyces sp.	
F3Z981	1,2-epoxyphenylacetyl-CoA isomerase	VI	Streptomyces sp.	
I2N5N3	1,2-epoxyphenylacetyl-CoA isomerase	VI	Streptomyces tsukubensis	
F2RIH4	1,2-epoxyphenylacetyl-CoA isomerase	VI	Streptomyces venezuelae	4.2.1.17
G2GCA9	1,2-epoxyphenylacetyl-CoA isomerase	VI	Streptomyces zinciresistens	
A0A085BVI8	1,2-epoxyphenylacetyl-CoA isomerase	VI	Sulfitobacter sp.	
A3SWQ9	1,2-epoxyphenylacetyl-CoA isomerase	VI	Sulfitobacter sp.	
F8I2X6	1,2-epoxyphenylacetyl-CoA isomerase	VI	Sulfobacillus acidophilus	
G8TUW8	1,2-epoxyphenylacetyl-CoA isomerase	VI	Sulfobacillus acidophilus	4.2.1.17
D7CKL0	1,2-epoxyphenylacetyl-CoA isomerase	VI	Syntrophothermus lipocalidus	
B7A929	1,2-epoxyphenylacetyl-CoA isomerase	VI	Thermus aquaticus	
E8PKD3	1,2-epoxyphenylacetyl-CoA isomerase	VI	Thermus scotoductus	
G8NBI4	1,2-epoxyphenylacetyl-CoA isomerase	VI	Thermus sp.	
F6DHT4	1,2-epoxyphenylacetyl-CoA isomerase	VI	Thermus thermophilus	
Q5SLK3	1,2-epoxyphenylacetyl-CoA isomerase	VI	Thermus thermophilus	
Q9F1Q4	1,2-epoxyphenylacetyl-CoA isomerase	VI	Thermus thermophilus	
E0XRE3	1,2-epoxyphenylacetyl-CoA isomerase	VI	uncultured Sphingobacteriales	
A6ERY9	1,2-epoxyphenylacetyl-CoA isomerase	VI	unidentified eubacterium	
A0A077QNI6	1,2-epoxyphenylacetyl-CoA isomerase	VI	Xenorhabdus bovienii	4.2.1.17
D3VFX8	1,2-epoxyphenylacetyl-CoA isomerase	VI	Xenorhabdus nematophila	4.2.1.17
D8FZI1	1,4-dihydroxy-2-napthoyl-CoA synthase	VIII	[Oscillatoria] sp.	4.1.3.36
A0A098BF11	1,4-dihydroxy-2-napthoyl-CoA synthase	VIII		
B0C9S2	1,4-dihydroxy-2-napthoyl-CoA synthase	VIII	Acaryochloris marina	4.1.3.36
C7RVD9	1,4-dihydroxy-2-napthoyl-CoA synthase	VIII	Accumulibacter phosphatis	4.1.3.36
C7M2I0	1,4-dihydroxy-2-napthoyl-CoA synthase	VIII	Acidimicrobium ferrooxidans	4.1.3.36
E8KKC9	1,4-dihydroxy-2-napthoyl-CoA synthase	VIII	Actinobacillus ureae	4.1.3.36
C6WND4	1,4-dihydroxy-2-napthoyl-CoA synthase	VIII	Actinosynnema mirum	4.1.3.36
A4SS24	1,4-dihydroxy-2-napthoyl-CoA synthase	VIII	Aeromonas salmonicida	4.1.3.36
B2UMY4	1,4-dihydroxy-2-napthoyl-CoA synthase	VIII	Akkermansia muciniphila	4.1.3.36
B7DN46	1,4-dihydroxy-2-napthoyl-CoA synthase	VIII	Alicyclobacillus acidocaldarius	4.1.3.36
B6EJ58	1,4-dihydroxy-2-napthoyl-CoA synthase	VIII	Aliivibrio salmonicida	4.1.3.36
D3RPJ2	1,4-dihydroxy-2-napthoyl-CoA synthase	VIII	Allochromatium vinosum	4.1.3.36
G0FP35	1,4-dihydroxy-2-napthoyl-CoA synthase	VIII	Amycolatopsis mediterranei	
Q8GYN9	1,4-dihydroxy-2-napthoyl-CoA synthase	VIII	Arabidopsis thaliana	4.1.3.36
D2U4H4	1,4-dihydroxy-2-napthoyl-CoA synthase	VIII	Arsenophonus nasoniae	4.1.3.36
D3EP98	1,4-dihydroxy-2-napthoyl-CoA synthase	VIII	Atelocyanobacterium thalassa	4.1.3.36
R8PZP1	1,4-dihydroxy-2-napthoyl-CoA synthase	VIII	Bacillus cereus	4.1.3.36
G2TN69	1,4-dihydroxy-2-napthoyl-CoA synthase	VIII	Bacillus coagulans	4.1.3.36
A6CM77	1,4-dihydroxy-2-napthoyl-CoA synthase	VIII	Bacillus sp.	4.1.3.36
L8AIK2	1,4-dihydroxy-2-napthoyl-CoA synthase	VIII	Bacillus subtilis	4.1.3.36
P23966	1,4-dihydroxy-2-napthoyl-CoA synthase	VIII	Bacillus subtilis	4.1.3.36
S0FA35	1,4-dihydroxy-2-napthoyl-CoA synthase	VIII	Bacteroides coprophilus	4.1.3.36
A0A081U6L2	1,4-dihydroxy-2-napthoyl-CoA synthase	VIII	Bacteroides fragilis	4.1.3.36
A0A069SUA8	1,4-dihydroxy-2-napthoyl-CoA synthase	VIII	Bacteroides vulgatus	4.1.3.36
Q6MHP8	1,4-dihydroxy-2-napthoyl-CoA synthase	VIII	Bdellovibrio bacteriovorus	4.1.3.36

Table S2.5. (continuation)

UniProt	Family [3]	Cluster	Species	EC Number
D0J9G2	1,4-dihydroxy-2-napthoyl-CoA synthase	VIII	Blattabacterium sp.	4.1.3.36
D0JAV0	1,4-dihydroxy-2-napthoyl-CoA synthase	VIII	Blattabacterium sp.	4.1.3.36
D4YM10	1,4-dihydroxy-2-napthoyl-CoA synthase	VIII	Brevibacterium mcbrellneri	4.1.3.36
D9Y4W2	1,4-dihydroxy-2-napthoyl-CoA synthase	VIII	Burkholderiales bacterium	4.1.3.36
C2M608	1,4-dihydroxy-2-napthoyl-CoA synthase	VIII	Capnocytophaga gingivalis	4.1.3.36
C7M712	1,4-dihydroxy-2-napthoyl-CoA synthase	VIII	Capnocytophaga ochracea	4.1.3.36
F0RC85	1,4-dihydroxy-2-napthoyl-CoA synthase	VIII	Cellulophaga lytica	4.1.3.36
A8I2W2	1,4-dihydroxy-2-napthoyl-CoA synthase	VIII	Chlamydomonas reinhardtii	4.1.3.36
B3QKZ3	1,4-dihydroxy-2-napthoyl-CoA synthase	VIII	Chlorobaculum parvum	4.1.3.36
Q3APV5	1,4-dihydroxy-2-napthoyl-CoA synthase	VIII	Chlorobium chlorochromatii	4.1.3.36
Q0YUC9	1,4-dihydroxy-2-napthoyl-CoA synthase	VIII	Chlorobium ferrooxidans	4.1.3.36
B3EG70	1,4-dihydroxy-2-napthoyl-CoA synthase	VIII	Chlorobium limicola	4.1.3.36
Q3B615	1,4-dihydroxy-2-napthoyl-CoA synthase	VIII	Chlorobium luteolum	4.1.3.36
A1BI76	1,4-dihydroxy-2-napthoyl-CoA synthase	VIII	Chlorobium phaeobacteroides	4.1.3.36
B3EMZ7	1,4-dihydroxy-2-napthoyl-CoA synthase	VIII	Chlorobium phaeobacteroides	4.1.3.36
A4SD57	1,4-dihydroxy-2-napthoyl-CoA synthase	VIII	Chlorobium phaeovibrioides	4.1.3.36
B8G7B5	1,4-dihydroxy-2-napthoyl-CoA synthase	VIII	Chloroflexus aggregans	4.1.3.36
B3QUU0	1,4-dihydroxy-2-napthoyl-CoA synthase	VIII	Chloroherpeton thalassium	4.1.3.36
A0A086F6A9	1,4-dihydroxy-2-napthoyl-CoA synthase	VIII	Chryseobacterium sp.	4.1.3.36
A5CNG4	1,4-dihydroxy-2-napthoyl-CoA synthase	VIII	Clavibacter michiganensis	4.1.3.36
D3F0C7	1,4-dihydroxy-2-napthoyl-CoA synthase	VIII	Conexibacter woesei	4.1.3.36
D5EQA7	1,4-dihydroxy-2-napthoyl-CoA synthase	VIII	Coraliomargarita akajimensis	4.1.3.36
A0A0F5DE12	1,4-dihydroxy-2-napthoyl-CoA synthase	VIII	Corynebacterium diphtheriae	4.1.3.36
D7WB20	1,4-dihydroxy-2-napthoyl-CoA synthase	VIII	Corynebacterium genitalium	4.1.3.36
C2GGE8	1,4-dihydroxy-2-napthoyl-CoA synthase	VIII	Corynebacterium glucuronolyticum	4.1.3.36
C0XUH3	1,4-dihydroxy-2-napthoyl-CoA synthase	VIII	Corynebacterium lipophiloflavum	4.1.3.36
C7MLC8	1,4-dihydroxy-2-napthoyl-CoA synthase	VIII	Cryptobacterium curtum	4.1.3.36
E5GBI7	1,4-dihydroxy-2-napthoyl-CoA synthase	VIII	Cucumis melo	
Q85G05	1,4-dihydroxy-2-napthoyl-CoA synthase	VIII	Cyanidioschyzon merolae	
Q9TM10	1,4-dihydroxy-2-napthoyl-CoA synthase	VIII	Cyanidium caldarium	4.1.3.36
B5IKY9	1,4-dihydroxy-2-napthoyl-CoA synthase	VIII	Cyanobium sp.	4.1.3.36
B7K9T2	1,4-dihydroxy-2-napthoyl-CoA synthase	VIII	Cyanothece sp.	4.1.3.36
B8HLB4	1,4-dihydroxy-2-napthoyl-CoA synthase	VIII	Cyanothece sp.	4.1.3.36
D4THD9	1,4-dihydroxy-2-napthoyl-CoA synthase	VIII	Cylindrospermopsis raciborskii	4.1.3.36
Q11TV1	1,4-dihydroxy-2-napthoyl-CoA synthase	VIII	Cytophaga hutchinsonii	4.1.3.36
Q47FL7	1,4-dihydroxy-2-napthoyl-CoA synthase	VIII	Dechloromonas aromatica	4.1.3.36
B8FA10	1,4-dihydroxy-2-napthoyl-CoA synthase	VIII	Desulfatibacillum alkenivorans	4.1.3.36
Q250I3	1,4-dihydroxy-2-napthoyl-CoA synthase	VIII	Desulfitobacterium hafniense	4.1.3.36
Q6ARP4	1,4-dihydroxy-2-napthoyl-CoA synthase	VIII	Desulfotalea psychrophila	4.1.3.36
C6W5A5	1,4-dihydroxy-2-napthoyl-CoA synthase	VIII	Dyadobacter fermentans	4.1.3.36
D4F7N9	1,4-dihydroxy-2-napthoyl-CoA synthase	VIII	Edwardsiella tarda	4.1.3.36
C7CWW0	1,4-dihydroxy-2-napthoyl-CoA synthase	VIII	Enterococcus faecalis	4.1.3.36
T2NLM6	1,4-dihydroxy-2-napthoyl-CoA synthase	VIII	Enterococcus faecium	4.1.3.36
D8MUS4	1,4-dihydroxy-2-napthoyl-CoA synthase	VIII	Erwinia billingiae	4.1.3.36
B1YK25	1,4-dihydroxy-2-napthoyl-CoA synthase	VIII	Exiguobacterium sibiricum	4.1.3.36
C4L319	1,4-dihydroxy-2-napthoyl-CoA synthase	VIII	Exiguobacterium sp.	4.1.3.36
C0BKA2	1,4-dihydroxy-2-napthoyl-CoA synthase	VIII	Flavobacteria bacterium	4.1.3.36
C0BNY0	1,4-dihydroxy-2-napthoyl-CoA synthase	VIII	Flavobacteria bacterium	4.1.3.36
Q26CL1	1,4-dihydroxy-2-napthoyl-CoA synthase	VIII	Flavobacteria bacterium	4.1.3.36
C6X650	1,4-dihydroxy-2-napthoyl-CoA synthase	VIII	Flavobacteriaceae bacterium	4.1.3.36
B2XXS5	1,4-dihydroxy-2-napthoyl-CoA synthase	VIII	Galdieria maxima	
A4IS12	1,4-dihydroxy-2-napthoyl-CoA synthase	VIII	Geobacillus thermodenitrificans	4.1.3.36
D2S516	1,4-dihydroxy-2-napthoyl-CoA synthase	VIII	Geodermatophilus obscurus	4.1.3.36
C6TE57	1,4-dihydroxy-2-napthoyl-CoA synthase	VIII	Glycine max	
A0M340	1,4-dihydroxy-2-napthoyl-CoA synthase	VIII	Gramella forsetii	4.1.3.36
D0IBX0	1,4-dihydroxy-2-napthoyl-CoA synthase	VIII	Grimontia hollisae	4.1.3.36

Table S2.5. (continuation)

UniProt	Family [3]	Cluster	Species	EC Number
E7QWV2	1,4-dihydroxy-2-napthoyl-CoA synthase	VIII	Haladaptatus paucihalophilus	4.1.3.36
D8J2U6	1,4-dihydroxy-2-napthoyl-CoA synthase	VIII	Halalkalicoccus jeotgali	4.1.3.36
D0LQC4	1,4-dihydroxy-2-napthoyl-CoA synthase	VIII	Haliangium ochraceum	4.1.3.36
E1X4E5	1,4-dihydroxy-2-napthoyl-CoA synthase	VIII	Halobacteriovorax marinus	4.1.3.36
B0R4U0	1,4-dihydroxy-2-napthoyl-CoA synthase	VIII	Halobacterium salinarum	4.1.3.36
Q18J10	1,4-dihydroxy-2-napthoyl-CoA synthase	VIII	Haloquadratum walsbyi	4.1.3.36
C7NRE5	1,4-dihydroxy-2-napthoyl-CoA synthase	VIII	Halorhabdus utahensis	4.1.3.36
A1WW43	1,4-dihydroxy-2-napthoyl-CoA synthase	VIII	Halorhodospira halophila	4.1.3.36
B9LR73	1,4-dihydroxy-2-napthoyl-CoA synthase	VIII	Halorubrum lacusprofundi	4.1.3.36
D2RS84	1,4-dihydroxy-2-napthoyl-CoA synthase	VIII	Haloterrigena turkmenica	4.1.3.36
D2PTD2	1,4-dihydroxy-2-napthoyl-CoA synthase	VIII	Kribbella flavida	4.1.3.36
D6TM00	1,4-dihydroxy-2-napthoyl-CoA synthase	VIII	Ktedonobacter racemifer	4.1.3.36
M5AAC0	1,4-dihydroxy-2-napthoyl-CoA synthase	VIII	Lactobacillus brevis	4.1.3.36
Q9CHK2	1,4-dihydroxy-2-napthoyl-CoA synthase	VIII	Lactococcus lactis	4.1.3.36
Q1MQ66	1,4-dihydroxy-2-napthoyl-CoA synthase	VIII	Lawsonia intracellularis	4.1.3.36
E4RRY1	1,4-dihydroxy-2-napthoyl-CoA synthase	VIII	Leadbetterella byssophila	4.1.3.36
A3XN30	1,4-dihydroxy-2-napthoyl-CoA synthase	VIII	Leeuwenhoekiella blandensis	4.1.3.36
Q6AHC7	1,4-dihydroxy-2-napthoyl-CoA synthase	VIII	Leifsonia xyli	4.1.3.36
Q040D4	1,4-dihydroxy-2-napthoyl-CoA synthase	VIII	Leuconostoc mesenteroides	4.1.3.36
A0A0E0UWG0	1,4-dihydroxy-2-napthoyl-CoA synthase	VIII	Listeria monocytogenes	4.1.3.36
B9E7P4	1,4-dihydroxy-2-napthoyl-CoA synthase	VIII	Macrococcus caseolyticus	4.1.3.36
E4TR92	1,4-dihydroxy-2-napthoyl-CoA synthase	VIII	Marivirga tractuosa	4.1.3.36
E8NG45	1,4-dihydroxy-2-napthoyl-CoA synthase	VIII	Microbacterium testaceum	4.1.3.36
L8NZ07	1,4-dihydroxy-2-napthoyl-CoA synthase	VIII	Microcystis aeruginosa	4.1.3.36
C4RCB5	1,4-dihydroxy-2-napthoyl-CoA synthase	VIII	Micromonospora sp.	4.1.3.36
A1ZNM5	1,4-dihydroxy-2-napthoyl-CoA synthase	VIII	Microscilla marina	4.1.3.36
A6FHU5	1,4-dihydroxy-2-napthoyl-CoA synthase	VIII	Moritella sp.	4.1.3.36
X8BBE0	1,4-dihydroxy-2-napthoyl-CoA synthase	VIII	Mycobacterium avium	4.1.3.36
E6TC08	1,4-dihydroxy-2-napthoyl-CoA synthase	VIII	Mycobacterium gilvum	4.1.3.36
A0QRD3	1,4-dihydroxy-2-napthoyl-CoA synthase	VIII	Mycobacterium smegmatis	4.1.3.36
A1UAV7	1,4-dihydroxy-2-napthoyl-CoA synthase	VIII	Mycobacterium sp.	4.1.3.36
P9WNP4	1,4-dihydroxy-2-napthoyl-CoA synthase	VIII	Mycobacterium tuberculosis	4.1.3.36
Q1D7L6	1,4-dihydroxy-2-napthoyl-CoA synthase	VIII	Myxococcus xanthus	4.1.3.36
Q3IQY8	1,4-dihydroxy-2-napthoyl-CoA synthase	VIII	Natronomonas pharaonis	4.1.3.36
Q5YPA1	1,4-dihydroxy-2-napthoyl-CoA synthase	VIII	Nocardia farcinica	4.1.3.36
A1SDB8	1,4-dihydroxy-2-napthoyl-CoA synthase	VIII	Nocardioides sp.	4.1.3.36
D7AXT2	1,4-dihydroxy-2-napthoyl-CoA synthase	VIII	Nocardiopsis dassonvillei	4.1.3.36
Q8ENZ6	1,4-dihydroxy-2-napthoyl-CoA synthase	VIII	Oceanobacillus iheyensis	4.1.3.36
R6F0F1	1,4-dihydroxy-2-napthoyl-CoA synthase	VIII	Odoribacter splanchnicus	4.1.3.36
A0NLC7	1,4-dihydroxy-2-napthoyl-CoA synthase	VIII	Oenococcus oeni	4.1.3.36
Q04H13	1,4-dihydroxy-2-napthoyl-CoA synthase	VIII	Oenococcus oeni	4.1.3.36
B1ZZJ3	1,4-dihydroxy-2-napthoyl-CoA synthase	VIII	Opitutus terrae	4.1.3.36
Q8LR33	1,4-dihydroxy-2-napthoyl-CoA synthase	VIII	Oryza sativa	
E1IFG5	1,4-dihydroxy-2-napthoyl-CoA synthase	VIII	Oscillochloris trichoides	4.1.3.36
A4RS73	1,4-dihydroxy-2-napthoyl-CoA synthase	VIII	Ostreococcus lucimarinus	
E4T4N4	1,4-dihydroxy-2-napthoyl-CoA synthase	VIII	Paludibacter propionicigenes	4.1.3.36
Q9CLV5	1,4-dihydroxy-2-napthoyl-CoA synthase	VIII	Pasteurella multocida	4.1.3.36
B1X586	1,4-dihydroxy-2-napthoyl-CoA synthase	VIII	Paulinella chromatophora	
B4SEU5	1,4-dihydroxy-2-napthoyl-CoA synthase	VIII	Pelodictyon phaeoclathratiforme	4.1.3.36
T4BPS0	1,4-dihydroxy-2-napthoyl-CoA synthase	VIII	Peptoclostridium difficile	4.1.3.36
L7NKA3	1,4-dihydroxy-2-napthoyl-CoA synthase	VIII	Photobacterium damsela	4.1.3.36
Q7N2K5	1,4-dihydroxy-2-napthoyl-CoA synthase	VIII	Photorhabdus luminescens	4.1.3.36
A9T6M9	1,4-dihydroxy-2-napthoyl-CoA synthase	VIII	Physcomitrella patens	
B8LPA3	1,4-dihydroxy-2-napthoyl-CoA synthase	VIII	Picea sitchensis	
E7RE08	1,4-dihydroxy-2-napthoyl-CoA synthase	VIII	Planococcus donghaensis	4.1.3.36
Q7MUJ0	1,4-dihydroxy-2-napthoyl-CoA synthase	VIII	Porphyromonas gingivalis	4.1.3.36

Table S2.5. (continuation)

UniProt	Family [3]	Cluster	Species	EC Number
A0A096B1B6	1,4-dihydroxy-2-napthoyl-CoA synthase	VIII	Prevotella amnii	4.1.3.36
D1PXD3	1,4-dihydroxy-2-napthoyl-CoA synthase	VIII	Prevotella bergensis	4.1.3.36
D8DW88	1,4-dihydroxy-2-napthoyl-CoA synthase	VIII	Prevotella bryantii	4.1.3.36
E1KRQ8	1,4-dihydroxy-2-napthoyl-CoA synthase	VIII	Prevotella disiens	4.1.3.36
E0NVS0	1,4-dihydroxy-2-napthoyl-CoA synthase	VIII	Prevotella marshii	4.1.3.36
F0F9H3	1,4-dihydroxy-2-napthoyl-CoA synthase	VIII	Prevotella multiformis	4.1.3.36
D5ETM5	1,4-dihydroxy-2-napthoyl-CoA synthase	VIII	Prevotella ruminicola	4.1.3.36
D3I9K4	1,4-dihydroxy-2-napthoyl-CoA synthase	VIII	Prevotella sp.	4.1.3.36
A2BQ89	1,4-dihydroxy-2-napthoyl-CoA synthase	VIII	Prochlorococcus marinus	4.1.3.36
A2C166	1,4-dihydroxy-2-napthoyl-CoA synthase	VIII	Prochlorococcus marinus	4.1.3.36
A2CAW3	1,4-dihydroxy-2-napthoyl-CoA synthase	VIII	Prochlorococcus marinus	4.1.3.36
A9BAW1	1,4-dihydroxy-2-napthoyl-CoA synthase	VIII	Prochlorococcus marinus	4.1.3.36
Q7V276	1,4-dihydroxy-2-napthoyl-CoA synthase	VIII	Prochlorococcus marinus	4.1.3.36
Q7VBN9	1,4-dihydroxy-2-napthoyl-CoA synthase	VIII	Prochlorococcus marinus	4.1.3.36
B4S4J6	1,4-dihydroxy-2-napthoyl-CoA synthase	VIII	Prosthecochloris aestuarii	4.1.3.36
C0AV89	1,4-dihydroxy-2-napthoyl-CoA synthase	VIII	Proteus penneri	4.1.3.36
Q6MCB1	1,4-dihydroxy-2-napthoyl-CoA synthase	VIII	Protochlamydia amoebophila	4.1.3.36
B2Q045	1,4-dihydroxy-2-napthoyl-CoA synthase	VIII	Providencia stuartii	4.1.3.36
K4IM85	1,4-dihydroxy-2-napthoyl-CoA synthase	VIII	Psychroflexus torquis	4.1.3.36
A1SRV0	1,4-dihydroxy-2-napthoyl-CoA synthase	VIII	Psychromonas ingrahamii	4.1.3.36
M4UAJ9	1,4-dihydroxy-2-napthoyl-CoA synthase	VIII	Psychromonas sp.	4.1.3.36
A9WRZ3	1,4-dihydroxy-2-napthoyl-CoA synthase	VIII	Renibacterium salmoninarum	4.1.3.36
Q2PQY7	1,4-dihydroxy-2-napthoyl-CoA synthase	VIII	Rhodococcus sp.	4.1.3.36
G2SJI3	1,4-dihydroxy-2-napthoyl-CoA synthase	VIII	Rhodothermus marinus	4.1.3.36
B9SH93	1,4-dihydroxy-2-napthoyl-CoA synthase	VIII	Ricinus communis	4.1.3.36
A4CL94	1,4-dihydroxy-2-napthoyl-CoA synthase	VIII	Robiginitalea biformata	4.1.3.36
A5UPB4	1,4-dihydroxy-2-napthoyl-CoA synthase	VIII	Roseiflexus sp.	4.1.3.36
Q1AS24	1,4-dihydroxy-2-napthoyl-CoA synthase	VIII	Rubrobacter xylanophilus	4.1.3.36
C7MTC1	1,4-dihydroxy-2-napthoyl-CoA synthase	VIII	Saccharomonospora viridis	4.1.3.36
A4FPV2	1,4-dihydroxy-2-napthoyl-CoA synthase	VIII	Saccharopolyspora erythraea	4.1.3.36
Q2RYX3	1,4-dihydroxy-2-napthoyl-CoA synthase	VIII	Salinibacter ruber	4.1.3.36
Q7CQ56	1,4-dihydroxy-2-napthoyl-CoA synthase	VIII	Salmonella typhimurium	4.1.3.36
D6ZD00	1,4-dihydroxy-2-napthoyl-CoA synthase	VIII	Segniliparus rotundus	4.1.3.36
D8RDJ4	1,4-dihydroxy-2-napthoyl-CoA synthase	VIII	Selaginella moellendorffii	
A9DFZ8	1,4-dihydroxy-2-napthoyl-CoA synthase	VIII	Shewanella benthica	4.1.3.36
B0TQD3	1,4-dihydroxy-2-napthoyl-CoA synthase	VIII	Shewanella halifaxensis	4.1.3.36
C7N2V4	1,4-dihydroxy-2-napthoyl-CoA synthase	VIII	Slackia heliotrinireducens	4.1.3.36
A9FX24	1,4-dihydroxy-2-napthoyl-CoA synthase	VIII	Sorangium cellulosum	4.1.3.36
D1C9C9	1,4-dihydroxy-2-napthoyl-CoA synthase	VIII	Sphaerobacter thermophilus	4.1.3.36
D2QQU6	1,4-dihydroxy-2-napthoyl-CoA synthase	VIII	Spirosoma linguale	4.1.3.36
Q5HQC3	1,4-dihydroxy-2-napthoyl-CoA synthase	VIII	Staphylococcus epidermidis	4.1.3.36
E8SET6	1,4-dihydroxy-2-napthoyl-CoA synthase	VIII	Staphylococcus pseudintermedius	4.1.3.36
D9VJ75	1,4-dihydroxy-2-napthoyl-CoA synthase	VIII	Streptomyces sp.	4.1.3.36
D2B2R3	1,4-dihydroxy-2-napthoyl-CoA synthase	VIII	Streptosporangium roseum	4.1.3.36
E8LJ74	1,4-dihydroxy-2-napthoyl-CoA synthase	VIII	Succinatimonas hippei	4.1.3.36
S3BG77	1,4-dihydroxy-2-napthoyl-CoA synthase	VIII	Sutterella wadsworthensis	4.1.3.36
Q31QP0	1,4-dihydroxy-2-napthoyl-CoA synthase	VIII	Synechococcus elongatus	4.1.3.36
A3Z1C0	1,4-dihydroxy-2-napthoyl-CoA synthase	VIII	Synechococcus sp.	4.1.3.36
A3Z7J0	1,4-dihydroxy-2-napthoyl-CoA synthase	VIII	Synechococcus sp.	4.1.3.36
A4CU15	1,4-dihydroxy-2-napthoyl-CoA synthase	VIII	Synechococcus sp.	4.1.3.36
A5GT35	1,4-dihydroxy-2-napthoyl-CoA synthase	VIII	Synechococcus sp.	4.1.3.36
B4WNU9	1,4-dihydroxy-2-napthoyl-CoA synthase	VIII	Synechococcus sp.	4.1.3.36
Q05TR5	1,4-dihydroxy-2-napthoyl-CoA synthase	VIII	Synechococcus sp.	4.1.3.36
Q0I9Z0	1,4-dihydroxy-2-napthoyl-CoA synthase	VIII	Synechococcus sp.	4.1.3.36
Q2JNU9	1,4-dihydroxy-2-napthoyl-CoA synthase	VIII	Synechococcus sp.	4.1.3.36
Q3AVF7	1,4-dihydroxy-2-napthoyl-CoA synthase	VIII	Synechococcus sp.	4.1.3.36

Table S2.5. (continuation)

UniProt	Family [3]	Cluster	Species	EC Number
Q6Q301	1,4-dihydroxy-2-napthoyl-CoA synthase	VIII	Synechococcus sp.	4.1.3.36
Q7U7I4	1,4-dihydroxy-2-napthoyl-CoA synthase	VIII	Synechococcus sp.	4.1.3.36
Q2LXE4	1,4-dihydroxy-2-napthoyl-CoA synthase	VIII	Syntrophus aciditrophicus	4.1.3.36
C5BMH5	1,4-dihydroxy-2-napthoyl-CoA synthase	VIII	Teredinibacter turnerae	4.1.3.36
E6SH67	1,4-dihydroxy-2-napthoyl-CoA synthase	VIII	Thermaerobacter marianensis	4.1.3.36
K6QF07	1,4-dihydroxy-2-napthoyl-CoA synthase	VIII	Thermaerobacter subterraneus	4.1.3.36
D1CBH4	1,4-dihydroxy-2-napthoyl-CoA synthase	VIII	Thermobaculum terrenum	4.1.3.36
Q47Q22	1,4-dihydroxy-2-napthoyl-CoA synthase	VIII	Thermobifida fusca	4.1.3.36
D6YB60	1,4-dihydroxy-2-napthoyl-CoA synthase	VIII	Thermobispora bispora	4.1.3.36
B9L3E0	1,4-dihydroxy-2-napthoyl-CoA synthase	VIII	Thermomicrobium roseum	4.1.3.36
D1A3R4	1,4-dihydroxy-2-napthoyl-CoA synthase	VIII	Thermomonospora curvata	4.1.3.36
Q8DG66	1,4-dihydroxy-2-napthoyl-CoA synthase	VIII	Thermosynechococcus elongatus	4.1.3.36
C4L9G1	1,4-dihydroxy-2-napthoyl-CoA synthase	VIII	Tolumonas auensis	4.1.3.36
Q10XC8	1,4-dihydroxy-2-napthoyl-CoA synthase	VIII	Trichodesmium erythraeum	4.1.3.36
Q83GX0	1,4-dihydroxy-2-napthoyl-CoA synthase	VIII	Tropheryma whipplei	4.1.3.36
D6KR42	1,4-dihydroxy-2-napthoyl-CoA synthase	VIII	Veillonella sp.	4.1.3.36
B5JFX6	1,4-dihydroxy-2-napthoyl-CoA synthase	VIII	Verrucomicrobiae bacterium	4.1.3.36
Q7MMF4	1,4-dihydroxy-2-napthoyl-CoA synthase	VIII	Vibrio vulnificus	4.1.3.36
A5B5A4	1,4-dihydroxy-2-napthoyl-CoA synthase	VIII	Vitis vinifera	
D8U3P9	1,4-dihydroxy-2-napthoyl-CoA synthase	VIII	Volvox carteri	
F8LDJ4	1,4-dihydroxy-2-napthoyl-CoA synthase	VIII	Waddlia chondrophila	4.1.3.36
F0P0X7	1,4-dihydroxy-2-napthoyl-CoA synthase	VIII	Weeksella virosa	4.1.3.36
C5R9D0	1,4-dihydroxy-2-napthoyl-CoA synthase	VIII	Weissella paramesenteroides	4.1.3.36
C0HEB1	1,4-dihydroxy-2-napthoyl-CoA synthase	VIII	Zea mays	
Q6FBZ0	crotonobetainyl-CoA hydratase	X	Acinetobacter baylyi	4.2.1.-
Q2KX73	crotonobetainyl-CoA hydratase	X	Bordetella avium	4.2.1.-
K0MFA7	crotonobetainyl-CoA hydratase	X	Bordetella parapertussis	4.2.1.-
A8ALR7	crotonobetainyl-CoA hydratase	X	Citrobacter koseri	4.2.1.149
C5B8Q7	crotonobetainyl-CoA hydratase	X	Edwardsiella ictaluri	4.2.1.-
D0ZCF6	crotonobetainyl-CoA hydratase	X	Edwardsiella tarda	
D4F8P2	crotonobetainyl-CoA hydratase	X	Edwardsiella tarda	
E3G9M6	crotonobetainyl-CoA hydratase	X	Enterobacter lignolyticus	4.2.1.149
E5YL73	crotonobetainyl-CoA hydratase	X	Enterobacteriaceae bacterium	
P31551	crotonobetainyl-CoA hydratase	X	Escherichia coli	4.2.1.149
U9YND2	crotonobetainyl-CoA hydratase	X	Escherichia coli	4.2.1.149
B7LWM7	crotonobetainyl-CoA hydratase	X	Escherichia fergusonii	4.2.1.149
A0NVW9	crotonobetainyl-CoA hydratase	X	Labrenzia aggregata	4.2.1.-
A3V8E8	crotonobetainyl-CoA hydratase	X	Loktanella vestfoldensis	4.2.1.-
A3JC02	crotonobetainyl-CoA hydratase	X	Marinobacter sp.	4.2.1.-
A3JC03	crotonobetainyl-CoA hydratase	X	Marinobacter sp.	4.2.1.-
V7K4P8	crotonobetainyl-CoA hydratase	X	Mycobacterium avium	4.2.1.17
V7L868	crotonobetainyl-CoA hydratase	X	Mycobacterium avium	4.2.1.17
V7P8P2	crotonobetainyl-CoA hydratase	X	Mycobacterium avium	4.2.1.17
X8CRT1	crotonobetainyl-CoA hydratase	X	Mycobacterium intracellulare	4.2.1.-
L7UYP3	crotonobetainyl-CoA hydratase	X	Mycobacterium liflandii	4.2.1.17
B2HMZ1	crotonobetainyl-CoA hydratase	X	Mycobacterium marinum	
Q73WL5	crotonobetainyl-CoA hydratase	X	Mycobacterium paratuberculosis	
A0A0D6HQM5	crotonobetainyl-CoA hydratase	X	Mycobacterium smegmatis	4.2.1.-; 4.2.1.17
A0A0D6IWJ0	crotonobetainyl-CoA hydratase	X	Mycobacterium smegmatis	4.2.1.-; 4.2.1.17
A0QRL1	crotonobetainyl-CoA hydratase	X	Mycobacterium smegmatis	4.2.1.-; 4.2.1.17
C2LLR4	crotonobetainyl-CoA hydratase	X	Proteus mirabilis	4.2.1.-
B4EY26	crotonobetainyl-CoA hydratase	X	Proteus mirabilis	4.2.1.149
D4C1C5	crotonobetainyl-CoA hydratase	X	Providencia rettgeri	4.2.1.-
Q98CR0	crotonobetainyl-CoA hydratase	X	Rhizobium loti	4.2.1.17
A3JUT7	crotonobetainyl-CoA hydratase	X	Rhodobacteraceae bacterium	4.2.1.-
Q0RW28	crotonobetainyl-CoA hydratase	X	Rhodococcus jostii	

Table S2.5. (continuation)

UniProt	Family [3]	Cluster	Species	EC Number
A3X727	crotonobetainyl-CoA hydratase	X	Roseobacter sp.	4.2.1.-
A4ET59	crotonobetainyl-CoA hydratase	X	Roseobacter sp.	4.2.1.-
A3SRC2	crotonobetainyl-CoA hydratase	X	Roseovarius nubinhibens	4.2.1.-
A3VZI4	crotonobetainyl-CoA hydratase	X	Roseovarius sp.	4.2.1.-
Q5LKU4	crotonobetainyl-CoA hydratase	X	Ruegeria pomeroyi	
Q5LPZ0	crotonobetainyl-CoA hydratase	X	Ruegeria pomeroyi	
A3K4X5	crotonobetainyl-CoA hydratase	X	Sagittula stellata	4.2.1.-
A9MR28	crotonobetainyl-CoA hydratase	X	Salmonella arizonae	4.2.1.149
B4TIG9	crotonobetainyl-CoA hydratase	X	Salmonella heidelberg	4.2.1.149
B4T6J5	crotonobetainyl-CoA hydratase	X	Salmonella newport	4.2.1.149
Q8Z9L5	crotonobetainyl-CoA hydratase	X	Salmonella typhi	4.2.1.149
B0TNP7	crotonobetainyl-CoA hydratase	X	Shewanella halifaxensis	
A3QGE0	crotonobetainyl-CoA hydratase	X	Shewanella loihica	4.2.1.17
A8H9V7	crotonobetainyl-CoA hydratase	X	Shewanella pealeana	
E2XD12	crotonobetainyl-CoA hydratase	X	Shigella dysenteriae	4.2.1.149
C7RQ76	delta(3,5)-delta(2,4)-dienoyl-CoA isomerase	VII	Accumulibacter phosphatis	
A1TS40	delta(3,5)-delta(2,4)-dienoyl-CoA isomerase	VII	Acidovorax citrulli	
O74188	delta(3,5)-delta(2,4)-dienoyl-CoA isomerase	VII	Agaricus bisporus	
A6RGX5	delta(3,5)-delta(2,4)-dienoyl-CoA isomerase	VII	Ajellomyces capsulatus	
C0NZL5	delta(3,5)-delta(2,4)-dienoyl-CoA isomerase	VII	Ajellomyces capsulatus	
C5GRI5	delta(3,5)-delta(2,4)-dienoyl-CoA isomerase	VII	Ajellomyces dermatitidis	
Q0VTC4	delta(3,5)-delta(2,4)-dienoyl-CoA isomerase	VII	Alcanivorax borkumensis	4.2.1.17
F2G639	delta(3,5)-delta(2,4)-dienoyl-CoA isomerase	VII	Alteromonas mediterranea	
D7MP94	delta(3,5)-delta(2,4)-dienoyl-CoA isomerase	VII	Arabidopsis lyrata	
D4AX01	delta(3,5)-delta(2,4)-dienoyl-CoA isomerase	VII	Arthroderma benhamiae	
F1L460	delta(3,5)-delta(2,4)-dienoyl-CoA isomerase	VII	Ascaris suum	
A1CLF2	delta(3,5)-delta(2,4)-dienoyl-CoA isomerase	VII	Aspergillus clavatus	
B8NXX1	delta(3,5)-delta(2,4)-dienoyl-CoA isomerase	VII	Aspergillus flavus	
A2QTG8	delta(3,5)-delta(2,4)-dienoyl-CoA isomerase	VII	Aspergillus niger	4.2.1.-
I8A5W7	delta(3,5)-delta(2,4)-dienoyl-CoA isomerase	VII	Aspergillus oryzae	
Q2TYE1	delta(3,5)-delta(2,4)-dienoyl-CoA isomerase	VII	Aspergillus oryzae	
Q0CS22	delta(3,5)-delta(2,4)-dienoyl-CoA isomerase	VII	Aspergillus terreus	
Q1N3H0	delta(3,5)-delta(2,4)-dienoyl-CoA isomerase	VII	Bermanella marisrubri	
Q3T172	delta(3,5)-delta(2,4)-dienoyl-CoA isomerase	VII	Bos taurus	
G2XUK2	delta(3,5)-delta(2,4)-dienoyl-CoA isomerase	VII	Botryotinia fuckeliana	
C3YGP8	delta(3,5)-delta(2,4)-dienoyl-CoA isomerase	VII	Branchiostoma floridae	
A8XW28	delta(3,5)-delta(2,4)-dienoyl-CoA isomerase	VII	Caenorhabditis briggsae	
Q20959	delta(3,5)-delta(2,4)-dienoyl-CoA isomerase	VII	Caenorhabditis elegans	
Q9TYL2	delta(3,5)-delta(2,4)-dienoyl-CoA isomerase	VII	Caenorhabditis elegans	
E3M0X1	delta(3,5)-delta(2,4)-dienoyl-CoA isomerase	VII	Caenorhabditis remanei	
E3M4F7	delta(3,5)-delta(2,4)-dienoyl-CoA isomerase	VII	Caenorhabditis remanei	
C1BNM4	delta(3,5)-delta(2,4)-dienoyl-CoA isomerase	VII	Caligus rogercresseyi	
D5VNY5	delta(3,5)-delta(2,4)-dienoyl-CoA isomerase	VII	Caulobacter segnis	
B0T486	delta(3,5)-delta(2,4)-dienoyl-CoA isomerase	VII	Caulobacter sp.	
Q2HGD6	delta(3,5)-delta(2,4)-dienoyl-CoA isomerase	VII	Chaetomium globosum	
E1ZQF9	delta(3,5)-delta(2,4)-dienoyl-CoA isomerase	VII	Chlorella variabilis	
Q7NV60	delta(3,5)-delta(2,4)-dienoyl-CoA isomerase	VII	Chromobacterium violaceum	4.2.1.17
A0A0E1RXB2	delta(3,5)-delta(2,4)-dienoyl-CoA isomerase	VII	Coccidioides immitis	
C5PE65	delta(3,5)-delta(2,4)-dienoyl-CoA isomerase	VII	Coccidioides posadasii	5.3.3.-
E3QDX9	delta(3,5)-delta(2,4)-dienoyl-CoA isomerase	VII	Colletotrichum graminicola	
B7X4H0	delta(3,5)-delta(2,4)-dienoyl-CoA isomerase	VII	Comamonas testosteroni	
A4A7R5	delta(3,5)-delta(2,4)-dienoyl-CoA isomerase	VII	Congregibacter litoralis	4.2.1.17
A8NLE6	delta(3,5)-delta(2,4)-dienoyl-CoA isomerase	VII	Coprinopsis cinerea	
B0W6D4	delta(3,5)-delta(2,4)-dienoyl-CoA isomerase	VII	Culex quinquefasciatus	
Q1LBV5	delta(3,5)-delta(2,4)-dienoyl-CoA isomerase	VII	Cupriavidus metallidurans	4.2.1.17
C9Y8M4	delta(3,5)-delta(2,4)-dienoyl-CoA isomerase	VII	Curvibacter putative	4.2.1.17

Table S2.5. (continuation)

UniProt	Family [3]	Cluster	Species	EC Number
A8KBX4	delta(3,5)-delta(2,4)-dienoyl-CoA isomerase	VII	Danio rerio	
Q5XJP4	delta(3,5)-delta(2,4)-dienoyl-CoA isomerase	VII	Danio rerio	
E9H131	delta(3,5)-delta(2,4)-dienoyl-CoA isomerase	VII	Daphnia pulex	
Q47AT2	delta(3,5)-delta(2,4)-dienoyl-CoA isomerase	VII	Dechloromonas aromatica	
E1QIP3	delta(3,5)-delta(2,4)-dienoyl-CoA isomerase	VII	Desulfarculus baarsii	
B8FEK9	delta(3,5)-delta(2,4)-dienoyl-CoA isomerase	VII	Desulfatibacillum alkenivorans	
B8FHR6	delta(3,5)-delta(2,4)-dienoyl-CoA isomerase	VII	Desulfatibacillum alkenivorans	
C0QD39	delta(3,5)-delta(2,4)-dienoyl-CoA isomerase	VII	Desulfobacterium autotrophicum	4.2.1.17
A9A0B6	delta(3,5)-delta(2,4)-dienoyl-CoA isomerase	VII	Desulfococcus oleovorans	
F0ZT90	delta(3,5)-delta(2,4)-dienoyl-CoA isomerase	VII	Dictyostelium purpureum	
B3MQI8	delta(3,5)-delta(2,4)-dienoyl-CoA isomerase	VII	Drosophila ananassae	
B4JK95	delta(3,5)-delta(2,4)-dienoyl-CoA isomerase	VII	Drosophila grimshawi	
B4L7X6	delta(3,5)-delta(2,4)-dienoyl-CoA isomerase	VII	Drosophila mojavensis	
B4GV21	delta(3,5)-delta(2,4)-dienoyl-CoA isomerase	VII	Drosophila persimilis	
B4M9X9	delta(3,5)-delta(2,4)-dienoyl-CoA isomerase	VII	Drosophila virilis	
B4NE83	delta(3,5)-delta(2,4)-dienoyl-CoA isomerase	VII	Drosophila willistoni	
D7FJQ4	delta(3,5)-delta(2,4)-dienoyl-CoA isomerase	VII	Ectocarpus siliculosus	
Q5BAA1	delta(3,5)-delta(2,4)-dienoyl-CoA isomerase	VII	Emericella nidulans	
F6S8M6	delta(3,5)-delta(2,4)-dienoyl-CoA isomerase	VII	Equus caballus	
Q0RGH4	delta(3,5)-delta(2,4)-dienoyl-CoA isomerase	VII	Frankia alni	4.2.1.17
A8KXJ0	delta(3,5)-delta(2,4)-dienoyl-CoA isomerase	VII	Frankia sp.	
B8KHC6	delta(3,5)-delta(2,4)-dienoyl-CoA isomerase	VII	gamma proteobacterium	
Q1YQ17	delta(3,5)-delta(2,4)-dienoyl-CoA isomerase	VII	gamma proteobacterium	4.2.1.17
A0A016PNJ1	delta(3,5)-delta(2,4)-dienoyl-CoA isomerase	VII	Gibberella zeae	
D3TS20	delta(3,5)-delta(2,4)-dienoyl-CoA isomerase	VII	Glossina morsitans	
C6TH01	delta(3,5)-delta(2,4)-dienoyl-CoA isomerase	VII	Glycine max	
D0L8W4	delta(3,5)-delta(2,4)-dienoyl-CoA isomerase	VII	Gordonia bronchialis	
F1YP26	delta(3,5)-delta(2,4)-dienoyl-CoA isomerase	VII	Gordonia neofelifaecis	4.2.1.17
F0XSM4	delta(3,5)-delta(2,4)-dienoyl-CoA isomerase	VII	Grosmannia clavigera	
Q2SLF3	delta(3,5)-delta(2,4)-dienoyl-CoA isomerase	VII	Hahella chejuensis	
E2C9X1	delta(3,5)-delta(2,4)-dienoyl-CoA isomerase	VII	Harpegnathos saltator	
Q13011	delta(3,5)-delta(2,4)-dienoyl-CoA isomerase	VII	Homo sapiens	5.3.3.-
F2DRP8	delta(3,5)-delta(2,4)-dienoyl-CoA isomerase	VII	Hordeum vulgare	
A0A059G039	delta(3,5)-delta(2,4)-dienoyl-CoA isomerase	VII	Hyphomonas hirschiana	
B7QNU0	delta(3,5)-delta(2,4)-dienoyl-CoA isomerase	VII	Ixodes scapularis	4.2.1.17
C1D8Q1	delta(3,5)-delta(2,4)-dienoyl-CoA isomerase	VII	Laribacter hongkongensis	4.2.1.17
A4H9G6	delta(3,5)-delta(2,4)-dienoyl-CoA isomerase	VII	Leishmania braziliensis	4.2.1.17
A4HXT3	delta(3,5)-delta(2,4)-dienoyl-CoA isomerase	VII	Leishmania infantum	4.2.1.17
C1BS93	delta(3,5)-delta(2,4)-dienoyl-CoA isomerase	VII	Lepeophtheirus salmonis	
E4ZQP3	delta(3,5)-delta(2,4)-dienoyl-CoA isomerase	VII	Leptosphaeria maculans	
B0SPS5	delta(3,5)-delta(2,4)-dienoyl-CoA isomerase	VII	Leptospira biflexa	4.2.1.17
M6C1Z2	delta(3,5)-delta(2,4)-dienoyl-CoA isomerase	VII	Leptospira borgpetersenii	
M3I948	delta(3,5)-delta(2,4)-dienoyl-CoA isomerase	VII	Leptospira interrogans	
Q72P47	delta(3,5)-delta(2,4)-dienoyl-CoA isomerase	VII	Leptospira interrogans	
E1G1M5	delta(3,5)-delta(2,4)-dienoyl-CoA isomerase	VII	Loa loa	
B8KXT1	delta(3,5)-delta(2,4)-dienoyl-CoA isomerase	VII	Luminiphilus syltensis	
G7PXH6	delta(3,5)-delta(2,4)-dienoyl-CoA isomerase	VII	Macaca fascicularis	
G7NNJ2	delta(3,5)-delta(2,4)-dienoyl-CoA isomerase	VII	Macaca mulatta	
A0YFK8	delta(3,5)-delta(2,4)-dienoyl-CoA isomerase	VII	marine gamma	
A0Z7Z7	delta(3,5)-delta(2,4)-dienoyl-CoA isomerase	VII	marine gamma	
A0Z644	delta(3,5)-delta(2,4)-dienoyl-CoA isomerase	VII	marine gamma	4.2.1.17
E4PR93	delta(3,5)-delta(2,4)-dienoyl-CoA isomerase	VII	Marinobacter adhaerens	
U7NWE2	delta(3,5)-delta(2,4)-dienoyl-CoA isomerase	VII	Marinobacter sp.	
A3Y7R6	delta(3,5)-delta(2,4)-dienoyl-CoA isomerase	VII	Marinomonas sp.	
E4TLL5	delta(3,5)-delta(2,4)-dienoyl-CoA isomerase	VII	Marivirga tractuosa	
A0A0A1UWK1	delta(3,5)-delta(2,4)-dienoyl-CoA isomerase	VII	Metarhizium robertsii	

Table S2.5. (continuation)

UniProt	Family [3]	Cluster	Species	EC Number
C1MR88	delta(3,5)-delta(2,4)-dienoyl-CoA isomerase	VII	Micromonas pusilla	
C1E0G4	delta(3,5)-delta(2,4)-dienoyl-CoA isomerase	VII	Micromonas sp.	
A1ZQ27	delta(3,5)-delta(2,4)-dienoyl-CoA isomerase	VII	Microscilla marina	5.3.3.-
A6F7E7	delta(3,5)-delta(2,4)-dienoyl-CoA isomerase	VII	Moritella sp.	4.2.1.17
V7K5W9	delta(3,5)-delta(2,4)-dienoyl-CoA isomerase	VII	Mycobacterium avium	4.2.1.17
X8E491	delta(3,5)-delta(2,4)-dienoyl-CoA isomerase	VII	Mycobacterium chelonae	
A4T6B6	delta(3,5)-delta(2,4)-dienoyl-CoA isomerase	VII	Mycobacterium gilvum	
E6TLI6	delta(3,5)-delta(2,4)-dienoyl-CoA isomerase	VII	Mycobacterium gilvum	
Q9CD94	delta(3,5)-delta(2,4)-dienoyl-CoA isomerase	VII	Mycobacterium leprae	
B2HLK1	delta(3,5)-delta(2,4)-dienoyl-CoA isomerase	VII	Mycobacterium marinum	
D5P984	delta(3,5)-delta(2,4)-dienoyl-CoA isomerase	VII	Mycobacterium parascrofulaceum	4.2.1.17
A0R5Y0	delta(3,5)-delta(2,4)-dienoyl-CoA isomerase	VII	Mycobacterium smegmatis	5.3.3.-; 4.2.1.17
A1UN55	delta(3,5)-delta(2,4)-dienoyl-CoA isomerase	VII	Mycobacterium sp.	
A0A0E8B8A5	delta(3,5)-delta(2,4)-dienoyl-CoA isomerase	VII	Mycobacterium tuberculosis	4.2.1.17
L7N577	delta(3,5)-delta(2,4)-dienoyl-CoA isomerase	VII	Mycobacterium tuberculosis	4.2.1.17
A1TGS8	delta(3,5)-delta(2,4)-dienoyl-CoA isomerase	VII	Mycobacterium vanbaalenii	
Q1D045	delta(3,5)-delta(2,4)-dienoyl-CoA isomerase	VII	Myxococcus xanthus	
D2VM20	delta(3,5)-delta(2,4)-dienoyl-CoA isomerase	VII	Naegleria gruberi	
C7YRY3	delta(3,5)-delta(2,4)-dienoyl-CoA isomerase	VII	Nectria haematococca	
A7SDI6	delta(3,5)-delta(2,4)-dienoyl-CoA isomerase	VII	Nematostella vectensis	
A1D5W9	delta(3,5)-delta(2,4)-dienoyl-CoA isomerase	VII	Neosartorya fischeri	
Q4WYW7	delta(3,5)-delta(2,4)-dienoyl-CoA isomerase	VII	Neosartorya fumigata	
Q2BQR2	delta(3,5)-delta(2,4)-dienoyl-CoA isomerase	VII	Neptuniibacter caesariensis	
Q5Z2W6	delta(3,5)-delta(2,4)-dienoyl-CoA isomerase	VII	Nocardia farcinica	
A3UJS8	delta(3,5)-delta(2,4)-dienoyl-CoA isomerase	VII	Oceanicaulis sp.	
E4Y2J1	delta(3,5)-delta(2,4)-dienoyl-CoA isomerase	VII	Oikopleura dioica	
Q8RUL6	delta(3,5)-delta(2,4)-dienoyl-CoA isomerase	VII	Oryza sativa	
C1BJW1	delta(3,5)-delta(2,4)-dienoyl-CoA isomerase	VII	Osmerus mordax	
A4S066	delta(3,5)-delta(2,4)-dienoyl-CoA isomerase	VII	Ostreococcus lucimarinus	
Q015E7	delta(3,5)-delta(2,4)-dienoyl-CoA isomerase	VII	Ostreococcus tauri	
C0SHD2	delta(3,5)-delta(2,4)-dienoyl-CoA isomerase	VII	Paracoccidioides brasiliensis	
C1GGI8	delta(3,5)-delta(2,4)-dienoyl-CoA isomerase	VII	Paracoccidioides brasiliensis	
C1GX33	delta(3,5)-delta(2,4)-dienoyl-CoA isomerase	VII	Paracoccidioides lutzii	
A0CCL1	delta(3,5)-delta(2,4)-dienoyl-CoA isomerase	VII	Paramecium tetraurelia	
A0DTH6	delta(3,5)-delta(2,4)-dienoyl-CoA isomerase	VII	Paramecium tetraurelia	
A0E1C1	delta(3,5)-delta(2,4)-dienoyl-CoA isomerase	VII	Paramecium tetraurelia	
E0VFP2	delta(3,5)-delta(2,4)-dienoyl-CoA isomerase	VII	Pediculus humanus	4.2.1.17
B6H316	delta(3,5)-delta(2,4)-dienoyl-CoA isomerase	VII	Penicillium rubens	
B7G2J4	delta(3,5)-delta(2,4)-dienoyl-CoA isomerase	VII	Phaeodactylum tricornutum	5.3.3.-
Q0UP31	delta(3,5)-delta(2,4)-dienoyl-CoA isomerase	VII	Phaeosphaeria nodorum	
B4RB92	delta(3,5)-delta(2,4)-dienoyl-CoA isomerase	VII	Phenylobacterium zucineum	
A9SW38	delta(3,5)-delta(2,4)-dienoyl-CoA isomerase	VII	Physcomitrella patens	
D0MQD0	delta(3,5)-delta(2,4)-dienoyl-CoA isomerase	VII	Phytophthora infestans	
A9NUY4	delta(3,5)-delta(2,4)-dienoyl-CoA isomerase	VII	Picea sitchensis	
B2B4P9	delta(3,5)-delta(2,4)-dienoyl-CoA isomerase	VII	Podospora anserina	
B9GMQ0	delta(3,5)-delta(2,4)-dienoyl-CoA isomerase	VII	Populus trichocarpa	
Q15W42	delta(3,5)-delta(2,4)-dienoyl-CoA isomerase	VII	Pseudoalteromonas atlantica	
B9Z0U2	delta(3,5)-delta(2,4)-dienoyl-CoA isomerase	VII	Pseudogulbenkiania ferrooxidans	
A0A086BY32	delta(3,5)-delta(2,4)-dienoyl-CoA isomerase	VII	Pseudomonas aeruginosa	
F3DED8	delta(3,5)-delta(2,4)-dienoyl-CoA isomerase	VII	Pseudomonas amygdali	4.2.1.17
F2ZMI3	delta(3,5)-delta(2,4)-dienoyl-CoA isomerase	VII	Pseudomonas coronafaciens	4.2.1.17
A0A0C2A5A8	delta(3,5)-delta(2,4)-dienoyl-CoA isomerase	VII	Pseudomonas fluorescens	
Q4KBL1	delta(3,5)-delta(2,4)-dienoyl-CoA isomerase	VII	Pseudomonas fluorescens	
C3KAY4	delta(3,5)-delta(2,4)-dienoyl-CoA isomerase	VII	Pseudomonas fluorescens	4.2.1.17
E2XRF2	delta(3,5)-delta(2,4)-dienoyl-CoA isomerase	VII	Pseudomonas fluorescens	4.2.1.17
A4XU14	delta(3,5)-delta(2,4)-dienoyl-CoA isomerase	VII	Pseudomonas mendocina	

Table S2.5. (continuation)

UniProt	Family [3]	Cluster	Species	EC Number
A0A0A7PPE3	delta(3,5)-delta(2,4)-dienoyl-CoA isomerase	VII	Pseudomonas putida	
B0KMR6	delta(3,5)-delta(2,4)-dienoyl-CoA isomerase	VII	Pseudomonas putida	
B1J6H5	delta(3,5)-delta(2,4)-dienoyl-CoA isomerase	VII	Pseudomonas putida	
S6L7R6	delta(3,5)-delta(2,4)-dienoyl-CoA isomerase	VII	Pseudomonas stutzeri	4.2.1.17
A0A099SYW3	delta(3,5)-delta(2,4)-dienoyl-CoA isomerase	VII	Pseudomonas syringae	
Q4ZTN1	delta(3,5)-delta(2,4)-dienoyl-CoA isomerase	VII	Pseudomonas syringae	
Q882B0	delta(3,5)-delta(2,4)-dienoyl-CoA isomerase	VII	Pseudomonas syringae	
E3RHH5	delta(3,5)-delta(2,4)-dienoyl-CoA isomerase	VII	Pyrenophora teres	
Q62651	delta(3,5)-delta(2,4)-dienoyl-CoA isomerase	VII	Rattus norvegicus	5.3.3.-
E4WCN7	delta(3,5)-delta(2,4)-dienoyl-CoA isomerase	VII	Rhodococcus equi	
E9T279	delta(3,5)-delta(2,4)-dienoyl-CoA isomerase	VII	Rhodococcus equi	
Q0S8F2	delta(3,5)-delta(2,4)-dienoyl-CoA isomerase	VII	Rhodococcus jostii	
C1BA52	delta(3,5)-delta(2,4)-dienoyl-CoA isomerase	VII	Rhodococcus opacus	5.3.3.-
A0A069JCS6	delta(3,5)-delta(2,4)-dienoyl-CoA isomerase	VII	Rhodococcus qingshengii	4.2.1.17
Q21X87	delta(3,5)-delta(2,4)-dienoyl-CoA isomerase	VII	Rhodoferax ferrireducens	
B9RM93	delta(3,5)-delta(2,4)-dienoyl-CoA isomerase	VII	Ricinus communis	4.2.1.17
C7MXJ2	delta(3,5)-delta(2,4)-dienoyl-CoA isomerase	VII	Saccharomonospora viridis	
D8QB02	delta(3,5)-delta(2,4)-dienoyl-CoA isomerase	VII	Schizophyllum commune	
A7E6I3	delta(3,5)-delta(2,4)-dienoyl-CoA isomerase	VII	Sclerotinia sclerotiorum	
D8S1K9	delta(3,5)-delta(2,4)-dienoyl-CoA isomerase	VII	Selaginella moellendorffii	
D8T948	delta(3,5)-delta(2,4)-dienoyl-CoA isomerase	VII	Selaginella moellendorffii	
Q082C3	delta(3,5)-delta(2,4)-dienoyl-CoA isomerase	VII	Shewanella frigidimarina	
F7W4P6	delta(3,5)-delta(2,4)-dienoyl-CoA isomerase	VII	Sordaria macrospora	
C5XGX9	delta(3,5)-delta(2,4)-dienoyl-CoA isomerase	VII	Sorghum bicolor	
D2QNV1	delta(3,5)-delta(2,4)-dienoyl-CoA isomerase	VII	Spirosoma linguale	
Q09E88	delta(3,5)-delta(2,4)-dienoyl-CoA isomerase	VII	Stigmatella aurantiaca	5.3.3.-
D9VGW1	delta(3,5)-delta(2,4)-dienoyl-CoA isomerase	VII	Streptomyces sp.	
A7YB26	delta(3,5)-delta(2,4)-dienoyl-CoA isomerase	VII	Sus scrofa	
Q1HL07	delta(3,5)-delta(2,4)-dienoyl-CoA isomerase	VII	Sus scrofa	
Q3Y5G5	delta(3,5)-delta(2,4)-dienoyl-CoA isomerase	VII	Sus scrofa	
B6QVV1	delta(3,5)-delta(2,4)-dienoyl-CoA isomerase	VII	Talaromyces marneffei	
B8MTE3	delta(3,5)-delta(2,4)-dienoyl-CoA isomerase	VII	Talaromyces stipitatus	
I7MCX3	delta(3,5)-delta(2,4)-dienoyl-CoA isomerase	VII	Tetrahymena thermophila	
D6WH44	delta(3,5)-delta(2,4)-dienoyl-CoA isomerase	VII	Tribolium castaneum	
D7EI39	delta(3,5)-delta(2,4)-dienoyl-CoA isomerase	VII	Tribolium castaneum	
E5S0Z8	delta(3,5)-delta(2,4)-dienoyl-CoA isomerase	VII	Trichinella spiralis	
F2RX04	delta(3,5)-delta(2,4)-dienoyl-CoA isomerase	VII	Trichophyton tonsurans	
B3RJF0	delta(3,5)-delta(2,4)-dienoyl-CoA isomerase	VII	Trichoplax adhaerens	
B3RL87	delta(3,5)-delta(2,4)-dienoyl-CoA isomerase	VII	Trichoplax adhaerens	
B3SER6	delta(3,5)-delta(2,4)-dienoyl-CoA isomerase	VII	Trichoplax adhaerens	
D5GPK1	delta(3,5)-delta(2,4)-dienoyl-CoA isomerase	VII	Tuber melanosporum	
E0XYA6	delta(3,5)-delta(2,4)-dienoyl-CoA isomerase	VII	uncultured Pseudomonadales	
D7U5Z9	delta(3,5)-delta(2,4)-dienoyl-CoA isomerase	VII	Vitis vinifera	
Q6DJP2	delta(3,5)-delta(2,4)-dienoyl-CoA isomerase	VII	Xenopus laevis	
Q28H32	delta(3,5)-delta(2,4)-dienoyl-CoA isomerase	VII	Xenopus tropicalis	
B4FD65	delta(3,5)-delta(2,4)-dienoyl-CoA isomerase	VII	Zea mays	
A8MRJ9	delta(3,5)-delta(2,4)-dienoyl-CoA isomerase	VII		
Q9FHR8	delta(3,5)-delta(2,4)-dienoyl-CoA isomerase	VII		
A0A0B6HTM9	Diffusible signal factor (DSF) synthase	II		
C4T5S8	Diffusible signal factor (DSF) synthase	II		
D4XBH3	Diffusible signal factor (DSF) synthase	II	Achromobacter piechaudii	4.2.1.17
E3HNE5	Diffusible signal factor (DSF) synthase	II	Achromobacter xylosoxidans	
E5U1C4	Diffusible signal factor (DSF) synthase	II	Achromobacter xylosoxidans	4.2.1.17
B1F8R6	Diffusible signal factor (DSF) synthase	II	Burkholderia ambifaria	
B1YZG5	Diffusible signal factor (DSF) synthase	II	Burkholderia ambifaria	
Q392D3	Diffusible signal factor (DSF) synthase	II	Burkholderia lata	

Table S2.5. (continuation)

UniProt	Family [3]	Cluster	Species	EC Number
B2JMW5	Diffusible signal factor (DSF) synthase	II	Burkholderia phymatum	
B2TE12	Diffusible signal factor (DSF) synthase	II	Burkholderia phytofirmans	
E8YR76	Diffusible signal factor (DSF) synthase	II	Burkholderia sp.	
I2IUA5	Diffusible signal factor (DSF) synthase	II	Burkholderia sp.	
Q13M84	Diffusible signal factor (DSF) synthase	II	Burkholderia xenovorans	
C9XTL6	Diffusible signal factor (DSF) synthase	II	Cronobacter turicensis	
D2ZCY5	Diffusible signal factor (DSF) synthase	II	Enterobacter cancerogenus	4.2.1.17
A4WAX7	Diffusible signal factor (DSF) synthase	II	Enterobacter sp.	
Q2NA37	Diffusible signal factor (DSF) synthase	II	Erythrobacter litoralis	
A3WBV4	Diffusible signal factor (DSF) synthase	II	Erythrobacter sp.	4.2.1.17
U4QVY1	Diffusible signal factor (DSF) synthase	II	Leptospirillum sp.	
Q0F1C3	Diffusible signal factor (DSF) synthase	II	Mariprofundus ferrooxydans	4.2.1.17
A2SN82	Diffusible signal factor (DSF) synthase	II	Methylibium petroleiphilum	
Q1GXW7	Diffusible signal factor (DSF) synthase	II	Methylobacillus flagellatus	
C5B5V9	Diffusible signal factor (DSF) synthase	II	Methylobacterium extorquens	
B1M8T5	Diffusible signal factor (DSF) synthase	II	Methylobacterium radiotolerans	
C0N6D5	Diffusible signal factor (DSF) synthase	II	Methylophaga thiooxydans	
D7DMQ5	Diffusible signal factor (DSF) synthase	II	Methylotenera versatilis	
E0LVL2	Diffusible signal factor (DSF) synthase	II	Pantoea sp.	
E6WDS2	Diffusible signal factor (DSF) synthase	II	Pantoea sp.	
E6WTP3	Diffusible signal factor (DSF) synthase	II	Pseudoxanthomonas suwonensis	
H8NPB6	Diffusible signal factor (DSF) synthase	II	Rahnella aquatilis	4.2.1.17
D5RRC0	Diffusible signal factor (DSF) synthase	II	Roseomonas cervicalis	4.2.1.17
D4DYW9	Diffusible signal factor (DSF) synthase	II	Serratia odorifera	4.2.1.17
S0ANM6	Diffusible signal factor (DSF) synthase	II	Serratia plymuthica	
A8GL46	Diffusible signal factor (DSF) synthase	II	Serratia proteamaculans	
D5CRD9	Diffusible signal factor (DSF) synthase	II	Sideroxydans lithotrophicus	
F6EVM0	Diffusible signal factor (DSF) synthase	II	Sphingobium chlorophenolicum	
Q1N7J5	Diffusible signal factor (DSF) synthase	II	Sphingomonas sp.	
D7A6D6	Diffusible signal factor (DSF) synthase	II	Starkeya novella	
A7K6P3	Diffusible signal factor (DSF) synthase	II	Stenotrophomonas maltophilia	
Q19VG8	Diffusible signal factor (DSF) synthase	II	Stenotrophomonas maltophilia	
A6Q7Q9	Diffusible signal factor (DSF) synthase	II	Sulfurovum sp.	
B8GTC7	Diffusible signal factor (DSF) synthase	II	Thioalkalivibrio sulfidiphilus	
Q3SFI5	Diffusible signal factor (DSF) synthase	II	Thiobacillus denitrificans	
D2UA80	Diffusible signal factor (DSF) synthase	II	Xanthomonas albilineans	4.2.1.17
G2LR94	Diffusible signal factor (DSF) synthase	II	Xanthomonas axonopodis	
A0A0C7E292	Diffusible signal factor (DSF) synthase	II	Xanthomonas campestris	
A0A0E2YV05	Diffusible signal factor (DSF) synthase	II	Xanthomonas campestris	
Q7CLS3	Diffusible signal factor (DSF) synthase	II	Xanthomonas campestris	
Q3BUB2	Diffusible signal factor (DSF) synthase	II	Xanthomonas campestris	4.2.1.17
R4N067	Diffusible signal factor (DSF) synthase	II	Xanthomonas citri	
Q5GYU8	Diffusible signal factor (DSF) synthase	II	Xanthomonas oryzae	
F0BFE0	Diffusible signal factor (DSF) synthase	II	Xanthomonas vesicatoria	
A0A060H9C8	Diffusible signal factor (DSF) synthase	II	Xylella fastidiosa	
A1JHW3	Diffusible signal factor (DSF) synthase	II	Yersinia enterocolitica	
C4SDQ4	Diffusible signal factor (DSF) synthase	II	Yersinia mollaretii	
E1ZF97	dodecenoyl-CoA delta-isomerase (mito)	XII	Chlorella variabilis	
B3S9B8	dodecenoyl-CoA delta-isomerase (mito)	XII	Trichoplax adhaerens	
C1DYG1	dodecenoyl-CoA delta-isomerase (mito)	X	Micromonas sp.	
J9K4B4	dodecenoyl-CoA delta-isomerase (mito)	V	Acyrthosiphon pisum	
Q17BZ3	dodecenoyl-CoA delta-isomerase (mito)	V	Aedes aegypti	
Q17BZ6	dodecenoyl-CoA delta-isomerase (mito)	V	Aedes aegypti	
D2HV72	dodecenoyl-CoA delta-isomerase (mito)	V	Ailuropoda melanoleuca	
F0WGM6	dodecenoyl-CoA delta-isomerase (mito)	V	Albugo laibachii	
Q5TNW4	dodecenoyl-CoA delta-isomerase (mito)	V	Anopheles gambiae	

Table S2.5. (continuation)

UniProt	Family [3]	Cluster	Species	EC Number
Q2NL38	dodecenoyl-CoA delta-isomerase (mito)	V	Bos taurus	
C3Z7P1	dodecenoyl-CoA delta-isomerase (mito)	V	Branchiostoma floridae	
C1C0F4	dodecenoyl-CoA delta-isomerase (mito)	V	Caligus clemensi	
F7H4L8	dodecenoyl-CoA delta-isomerase (mito)	V	Callithrix jacchus	
E2AID5	dodecenoyl-CoA delta-isomerase (mito)	V	Camponotus floridanus	
A8IRX0	dodecenoyl-CoA delta-isomerase (mito)	V	Chlamydomonas reinhardtii	
D1FPK3	dodecenoyl-CoA delta-isomerase (mito)	V	Cimex lectularius	
B0X2M2	dodecenoyl-CoA delta-isomerase (mito)	V	Culex quinquefasciatus	
B0X2M5	dodecenoyl-CoA delta-isomerase (mito)	V	Culex quinquefasciatus	
Q7SXW5	dodecenoyl-CoA delta-isomerase (mito)	V	Danio rerio	
E9FSH3	dodecenoyl-CoA delta-isomerase (mito)	V	Daphnia pulex	
B3MPA9	dodecenoyl-CoA delta-isomerase (mito)	V	Drosophila ananassae	
B3N8K0	dodecenoyl-CoA delta-isomerase (mito)	V	Drosophila erecta	
B3N8K1	dodecenoyl-CoA delta-isomerase (mito)	V	Drosophila erecta	
B4JE40	dodecenoyl-CoA delta-isomerase (mito)	V	Drosophila grimshawi	
Q9VL67	dodecenoyl-CoA delta-isomerase (mito)	V	Drosophila melanogaster	5.3.3.8
Q9VL68	dodecenoyl-CoA delta-isomerase (mito)	V	Drosophila melanogaster	5.3.3.8
B4KJE7	dodecenoyl-CoA delta-isomerase (mito)	V	Drosophila mojavensis	
B4KJE9	dodecenoyl-CoA delta-isomerase (mito)	V	Drosophila mojavensis	
B4G7V4	dodecenoyl-CoA delta-isomerase (mito)	V	Drosophila persimilis	
B4G7V5	dodecenoyl-CoA delta-isomerase (mito)	V	Drosophila persimilis	
B5DJ49	dodecenoyl-CoA delta-isomerase (mito)	V	Drosophila pseudoobscura	
Q29NE3	dodecenoyl-CoA delta-isomerase (mito)	V	Drosophila pseudoobscura	
B4HWC5	dodecenoyl-CoA delta-isomerase (mito)	V	Drosophila sechellia	
B4Q897	dodecenoyl-CoA delta-isomerase (mito)	V	Drosophila simulans	
B4LRD3	dodecenoyl-CoA delta-isomerase (mito)	V	Drosophila virilis	
B4MZ14	dodecenoyl-CoA delta-isomerase (mito)	V	Drosophila willistoni	
B4N061	dodecenoyl-CoA delta-isomerase (mito)	V	Drosophila willistoni	
B4NYP1	dodecenoyl-CoA delta-isomerase (mito)	V	Drosophila yakuba	
C1BWB8	dodecenoyl-CoA delta-isomerase (mito)	V	Esox lucius	
D3TPU1	dodecenoyl-CoA delta-isomerase (mito)	V	Glossina morsitans	
E2BKH1	dodecenoyl-CoA delta-isomerase (mito)	V	Harpegnathos saltator	
D2XML5	dodecenoyl-CoA delta-isomerase (mito)	V	Heliothis virescens	
Q96DC0	dodecenoyl-CoA delta-isomerase (mito)	V	Homo sapiens	
P42126	dodecenoyl-CoA delta-isomerase (mito)	V	Homo sapiens	5.3.3.8
A4HJK3	dodecenoyl-CoA delta-isomerase (mito)	V	Leishmania braziliensis	5.3.3.8
A4HJL5	dodecenoyl-CoA delta-isomerase (mito)	V	Leishmania braziliensis	5.3.3.8
E9BN03	dodecenoyl-CoA delta-isomerase (mito)	V	Leishmania donovani	
E9BN11	dodecenoyl-CoA delta-isomerase (mito)	V	Leishmania donovani	
A4I738	dodecenoyl-CoA delta-isomerase (mito)	V	Leishmania infantum	1.1.1.35; 5.3.3.8
Q4Q628	dodecenoyl-CoA delta-isomerase (mito)	V	Leishmania major	1.1.1.35; 5.3.3.8
Q4Q636	dodecenoyl-CoA delta-isomerase (mito)	V	Leishmania major	5.3.3.8
E9B233	dodecenoyl-CoA delta-isomerase (mito)	V	Leishmania mexicana	
E9B226	dodecenoyl-CoA delta-isomerase (mito)	V	Leishmania mexicana	5.3.3.8
D3PHA3	dodecenoyl-CoA delta-isomerase (mito)	V	Lepeophtheirus salmonis	
C1C4V3	dodecenoyl-CoA delta-isomerase (mito)	V	Lithobates catesbeiana	
C1MQK5	dodecenoyl-CoA delta-isomerase (mito)	V	Micromonas pusilla	
A9UPK7	dodecenoyl-CoA delta-isomerase (mito)	V	Monosiga brevicollis	
P42125	dodecenoyl-CoA delta-isomerase (mito)	V	Mus musculus	5.3.3.8
D2VEL7	dodecenoyl-CoA delta-isomerase (mito)	V	Naegleria gruberi	
D2VNQ9	dodecenoyl-CoA delta-isomerase (mito)	V	Naegleria gruberi	
K7ISH0	dodecenoyl-CoA delta-isomerase (mito)	V	Nasonia vitripennis	
A7RUH9	dodecenoyl-CoA delta-isomerase (mito)	V	Nematostella vectensis	
A7RUI0	dodecenoyl-CoA delta-isomerase (mito)	V	Nematostella vectensis	
G1RDM4	dodecenoyl-CoA delta-isomerase (mito)	V	Nomascus leucogenys	
E4XPU3	dodecenoyl-CoA delta-isomerase (mito)	V	Oikopleura dioica	

Table S2.5. (continuation)

UniProt	Family [3]	Cluster	Species	EC Number
C1BLR2	dodecenoyl-CoA delta-isomerase (mito)	V	Osmerus mordax	
A4RR99	dodecenoyl-CoA delta-isomerase (mito)	V	Ostreococcus lucimarinus	
E0VVX4	dodecenoyl-CoA delta-isomerase (mito)	V	Pediculus humanus	5.3.3.8
B7FRZ1	dodecenoyl-CoA delta-isomerase (mito)	V	Phaeodactylum tricornutum	
D0MXD7	dodecenoyl-CoA delta-isomerase (mito)	V	Phytophthora infestans	
Q68G41	dodecenoyl-CoA delta-isomerase (mito)	V	Rattus norvegicus	
P23965	dodecenoyl-CoA delta-isomerase (mito)	V	Rattus norvegicus	5.3.3.8
Q64592	dodecenoyl-CoA delta-isomerase (mito)	V	Rattus norvegicus	5.3.3.8
C0HAW6	dodecenoyl-CoA delta-isomerase (mito)	V	Salmo salar	
F2UL70	dodecenoyl-CoA delta-isomerase (mito)	V	Salpingoeca rosetta	
W4ZFH9	dodecenoyl-CoA delta-isomerase (mito)	V	Strongylocentrotus purpuratus	
B8C1Y7	dodecenoyl-CoA delta-isomerase (mito)	V	Thalassiosira pseudonana	5.3.3.8
B3S9B9	dodecenoyl-CoA delta-isomerase (mito)	V	Trichoplax adhaerens	
C9ZME0	dodecenoyl-CoA delta-isomerase (mito)	V	Trypanosoma brucei	5.3.3.8
Q57V74	dodecenoyl-CoA delta-isomerase (mito)	V	Trypanosoma brucei	5.3.3.8
Q584L4	dodecenoyl-CoA delta-isomerase (mito)	V	Trypanosoma brucei	5.3.3.8
K4E5W6	dodecenoyl-CoA delta-isomerase (mito)	V	Trypanosoma cruzi	
Q4CRI1	dodecenoyl-CoA delta-isomerase (mito)	V	Trypanosoma cruzi	5.3.3.8
Q4CTQ3	dodecenoyl-CoA delta-isomerase (mito)	V	Trypanosoma cruzi	5.3.3.8
D8UEX0	dodecenoyl-CoA delta-isomerase (mito)	V	Volvox carteri	
Q6IR83	dodecenoyl-CoA delta-isomerase (mito)	V	Xenopus laevis	
Q5M8Y6	dodecenoyl-CoA delta-isomerase (mito)	V	Xenopus tropicalis	
A7ETW4	dodecenoyl-CoA delta-isomerase (perox)	VI	Sclerotinia sclerotiorum	
Q5B9R4	dodecenoyl-CoA delta-isomerase (perox)	XII	Emericella nidulans	
E4ZTF3	dodecenoyl-CoA delta-isomerase (perox)	XII	Leptosphaeria maculans	
A0A014QSB5	dodecenoyl-CoA delta-isomerase (perox)	XII	Metarhizium robertsii	
Q0CKJ3	dodecenoyl-CoA delta-isomerase (perox)	X	Aspergillus terreus	
B0CSA7	dodecenoyl-CoA delta-isomerase (perox)	X	Laccaria bicolor	
Q0V5P8	dodecenoyl-CoA delta-isomerase (perox)	X	Phaeosphaeria nodorum	
A6R688	dodecenoyl-CoA delta-isomerase (perox)	III	Ajellomyces capsulatus	
Q74ZB6	dodecenoyl-CoA delta-isomerase (perox)	III	Ashbya gossypii	
A1CMB8	dodecenoyl-CoA delta-isomerase (perox)	III	Aspergillus clavatus	
A2R750	dodecenoyl-CoA delta-isomerase (perox)	III	Aspergillus niger	
A2R4R0	dodecenoyl-CoA delta-isomerase (perox)	III	Aspergillus niger	5.3.3.-
A2QLZ9	dodecenoyl-CoA delta-isomerase (perox)	III	Aspergillus niger	5.3.3.8
I8A2A8	dodecenoyl-CoA delta-isomerase (perox)	III	Aspergillus oryzae	
I8U401	dodecenoyl-CoA delta-isomerase (perox)	III	Aspergillus oryzae	
Q0CD23	dodecenoyl-CoA delta-isomerase (perox)	III	Aspergillus terreus	
Q0CH09	dodecenoyl-CoA delta-isomerase (perox)	III	Aspergillus terreus	
Q5A318	dodecenoyl-CoA delta-isomerase (perox)	III	Candida albicans	
B9WMQ0	dodecenoyl-CoA delta-isomerase (perox)	III	Candida dubliniensis	5.3.3.8
Q6FUA2	dodecenoyl-CoA delta-isomerase (perox)	III	Candida glabrata	
C5MAF1	dodecenoyl-CoA delta-isomerase (perox)	III	Candida tropicalis	
C5MAF3	dodecenoyl-CoA delta-isomerase (perox)	III	Candida tropicalis	
Q2H970	dodecenoyl-CoA delta-isomerase (perox)	III	Chaetomium globosum	
C4XYI9	dodecenoyl-CoA delta-isomerase (perox)	III	Clavispora lusitaniae	
C5NZU6	dodecenoyl-CoA delta-isomerase (perox)	III	Coccidioides posadasii	5.3.3.8
E3Q382	dodecenoyl-CoA delta-isomerase (perox)	III	Colletotrichum graminicola	
A8NFK4	dodecenoyl-CoA delta-isomerase (perox)	III	Coprinopsis cinerea	
Q6BQU9	dodecenoyl-CoA delta-isomerase (perox)	III	Debaryomyces hansenii	
A0A016PJY3	dodecenoyl-CoA delta-isomerase (perox)	III	Gibberella zeae	
F0XUH5	dodecenoyl-CoA delta-isomerase (perox)	III	Grosmannia clavigera	
Q6CUI1	dodecenoyl-CoA delta-isomerase (perox)	III	Kluyveromyces lactis	
F2QLP4	dodecenoyl-CoA delta-isomerase (perox)	III	Komagataella phaffii	
C5DN92	dodecenoyl-CoA delta-isomerase (perox)	III	Lachancea thermotolerans	
E5A0G2	dodecenoyl-CoA delta-isomerase (perox)	III	Leptosphaeria maculans	

Table S2.5. (continuation)

UniProt	Family [3]	Cluster	Species	EC Number
L7JKB4	dodecenoyl-CoA delta-isomerase (perox)	III	Magnaporthe oryzae	
E9E534	dodecenoyl-CoA delta-isomerase (perox)	III	Metarhizium acridum	
A5D9Y8	dodecenoyl-CoA delta-isomerase (perox)	III	Meyerozyma guilliermondii	
C7YYP5	dodecenoyl-CoA delta-isomerase (perox)	III	Nectria haematococca	
Q7RZR5	dodecenoyl-CoA delta-isomerase (perox)	III	Neurospora crassa	
D7PDN1	dodecenoyl-CoA delta-isomerase (perox)	III	Ophiocordyceps unilateralis	
C1GCQ2	dodecenoyl-CoA delta-isomerase (perox)	III	Paracoccidioides brasiliensis	
B6HDN3	dodecenoyl-CoA delta-isomerase (perox)	III	Penicillium rubens	
Q0V2T7	dodecenoyl-CoA delta-isomerase (perox)	III	Phaeosphaeria nodorum	
B2AVZ8	dodecenoyl-CoA delta-isomerase (perox)	III	Podospora anserina	
E3S7U7	dodecenoyl-CoA delta-isomerase (perox)	III	Pyrenophora teres	
B2WBD8	dodecenoyl-CoA delta-isomerase (perox)	III	Pyrenophora tritici-repentis	
A0A0D4THP4	dodecenoyl-CoA delta-isomerase (perox)	III	Saccharomyces cerevisiae	
E7KRY7	dodecenoyl-CoA delta-isomerase (perox)	III	Saccharomyces cerevisiae	
E7NKV3	dodecenoyl-CoA delta-isomerase (perox)	III	Saccharomyces cerevisiae	
E7Q745	dodecenoyl-CoA delta-isomerase (perox)	III	Saccharomyces cerevisiae	
Q08558	dodecenoyl-CoA delta-isomerase (perox)	III	Saccharomyces cerevisiae	5.3.3.-
Q05871	dodecenoyl-CoA delta-isomerase (perox)	III	Saccharomyces cerevisiae	5.3.3.8
A3LYV7	dodecenoyl-CoA delta-isomerase (perox)	III	Scheffersomyces stipitis	
D8PVE9	dodecenoyl-CoA delta-isomerase (perox)	III	Schizophyllum commune	
F7VYE5	dodecenoyl-CoA delta-isomerase (perox)	III	Sordaria macrospora	
B6Q2F0	dodecenoyl-CoA delta-isomerase (perox)	III	Talaromyces marneffei	
B8M6D4	dodecenoyl-CoA delta-isomerase (perox)	III	Talaromyces stipitatus	
D4DII1	dodecenoyl-CoA delta-isomerase (perox)	III	Trichophyton verrucosum	
D5GAD9	dodecenoyl-CoA delta-isomerase (perox)	III	Tuber melanosporum	
A0A0D1CVH5	dodecenoyl-CoA delta-isomerase (perox)	III	Ustilago maydis	
A7THM4	dodecenoyl-CoA delta-isomerase (perox)	III	Vanderwaltozyma polyspora	
A7TS99	dodecenoyl-CoA delta-isomerase (perox)	III	Vanderwaltozyma polyspora	
C9SPS2	dodecenoyl-CoA delta-isomerase (perox)	III	Verticillium alfalfae	
C5DWF6	dodecenoyl-CoA delta-isomerase (perox)	III	Zygosaccharomyces rouxii	
C5DWF9	dodecenoyl-CoA delta-isomerase (perox)	III	Zygosaccharomyces rouxii	
A0A0E1QX95	enoyl-CoA hydratase	VI		
K0MB48	enoyl-CoA hydratase	VI	Bordetella parapertussis	
K0MIR4	enoyl-CoA hydratase	VI	Bordetella parapertussis	
A0A0A0XGJ3	enoyl-CoA hydratase	VI	Bordetella pertussis	4.2.1.17
A0A0A0XPA8	enoyl-CoA hydratase	VI	Bordetella pertussis	4.2.1.17
C0GAD9	enoyl-CoA hydratase	VI	Brucella ceti	
A0A0A5L5G2	enoyl-CoA hydratase	VI	Burkholderia cepacia	4.2.1.17
Q39JY7	enoyl-CoA hydratase	VI	Burkholderia lata	4.2.1.17
A8EGC0	enoyl-CoA hydratase	VI	Burkholderia pseudomallei	
A0A0F5KIG8	enoyl-CoA hydratase	VI	Burkholderia pseudomallei	4.2.1.17
Q13TX4	enoyl-CoA hydratase	VI	Burkholderia xenovorans	4.2.1.17
D5VJS7	enoyl-CoA hydratase	VI	Caulobacter segnis	
D8DCV9	enoyl-CoA hydratase	VI	Comamonas testosteroni	
B3R6Z2	enoyl-CoA hydratase	VI	Cupriavidus taiwanensis	4.2.1.17
Q1IXE2	enoyl-CoA hydratase	VI	Deinococcus geothermalis	4.2.1.17
D2ZC02	enoyl-CoA hydratase	VI	Enterobacter cancerogenus	4.2.1.17
T0QCH6	enoyl-CoA hydratase	VI	Geobacillus sp.	
Q39TH3	enoyl-CoA hydratase	VI	Geobacter metallireducens	
D3LRN5	enoyl-CoA hydratase	VI	Micrococcus luteus	
Q3K7R8	enoyl-CoA hydratase	VI	Pseudomonas fluorescens	
D8N606	enoyl-CoA hydratase	VI	Ralstonia solanacearum	4.2.1.17
G2ZYW8	enoyl-CoA hydratase	VI	Ralstonia syzygii	4.2.1.17
Q2KBR9	enoyl-CoA hydratase	VI	Rhizobium etli	4.2.1.17
E4WFI6	enoyl-CoA hydratase	VI	Rhodococcus equi	
C0ZQZ0	enoyl-CoA hydratase	VI	Rhodococcus erythropolis	4.2.1.17

Table S2.5. (continuation)

UniProt	Family [3]	Cluster	Species	EC Number
E6VG60	enoyl-CoA hydratase	VI	Rhodopseudomonas palustris	
A3W6I2	enoyl-CoA hydratase	VI	Roseovarius sp.	
E7N231	enoyl-CoA hydratase	VI	Selenomonas artemidis	
C4V110	enoyl-CoA hydratase	VI	Selenomonas flueggei	4.2.1.55
S0AIY9	enoyl-CoA hydratase	VI	Serratia plymuthica	5.3.3.18
Q47SM8	enoyl-CoA hydratase	VI	Thermobifida fusca	4.2.1.17
Q47TV9	enoyl-CoA hydratase	VI	Thermobifida fusca	4.2.1.17
D5WZE4	enoyl-CoA hydratase	VI	Thiomonas intermedia	
E6V0E3	enoyl-CoA hydratase	VI	Variovorax paradoxus	
D6DIH8	enoyl-CoA hydratase	XII	[Clostridium] cf.	4.2.1.17
D6DM85	enoyl-CoA hydratase	XII	[Clostridium] cf.	4.2.1.55
E7GI75	enoyl-CoA hydratase	XII	[Clostridium] symbiosum	
E7GR51	enoyl-CoA hydratase	XII	[Clostridium] symbiosum	
U2C8F6	enoyl-CoA hydratase	XII	[Clostridium] symbiosum	
I5AR59	enoyl-CoA hydratase	XII	[Eubacterium] cellulosolvens	
C0ERR2	enoyl-CoA hydratase	XII	[Eubacterium] hallii	4.2.1.55
E0QIU2	enoyl-CoA hydratase	XII	[Eubacterium] yurii	4.2.1.55
A0A098HHW6	enoyl-CoA hydratase	XII		
A0A0E1QM62	enoyl-CoA hydratase	XII		
A0A0E1QZB5	enoyl-CoA hydratase	XII		
E5U479	enoyl-CoA hydratase	XII		
F3JJE7	enoyl-CoA hydratase	XII		
X7T2T4	enoyl-CoA hydratase	XII		
C7RRT3	enoyl-CoA hydratase	XII	Accumulibacter phosphatis	
D4XJ03	enoyl-CoA hydratase	XII	Achromobacter piechaudii	4.2.1.-
D4XGK5	enoyl-CoA hydratase	XII	Achromobacter piechaudii	4.2.1.55
E3HJ70	enoyl-CoA hydratase	XII	Achromobacter xylosoxidans	
E3HMI0	enoyl-CoA hydratase	XII	Achromobacter xylosoxidans	4.2.1.17
E5U1B7	enoyl-CoA hydratase	XII	Achromobacter xylosoxidans	4.2.1.17
D2RL82	enoyl-CoA hydratase	XII	Acidaminococcus fermentans	
U2VV69	enoyl-CoA hydratase	XII	Acidaminococcus sp.	4.2.1.55
D9PZU6	enoyl-CoA hydratase	XII	Acidilobus saccharovorans	4.2.1.17
C7M145	enoyl-CoA hydratase	XII	Acidimicrobium ferrooxidans	
A5FWB1	enoyl-CoA hydratase	XII	Acidiphilium cryptum	
C1FAD2	enoyl-CoA hydratase	XII	Acidobacterium capsulatum	4.2.1.55
A0LRW4	enoyl-CoA hydratase	XII	Acidothermus cellulolyticus	4.2.1.17
A0LSP5	enoyl-CoA hydratase	XII	Acidothermus cellulolyticus	4.2.1.17
Q0IEM7	enoyl-CoA hydratase	XII	Aedes aegypti	
E2SCU8	enoyl-CoA hydratase	XII	Aeromicrobium marinum	
Q9YEI7	enoyl-CoA hydratase	XII	Aeropyrum pernix	
Q9YG45	enoyl-CoA hydratase	XII	Aeropyrum pernix	
D6V445	enoyl-CoA hydratase	XII	Afipia sp.	
E0MTD1	enoyl-CoA hydratase	XII	Ahrensia sp.	
C5GJ49	enoyl-CoA hydratase	XII	Ajellomyces dermatitidis	
F2T809	enoyl-CoA hydratase	XII	Ajellomyces dermatitidis	
A3I2T8	enoyl-CoA hydratase	XII	Algoriphagus machipongonensis	
E8TQA1	enoyl-CoA hydratase	XII	Alicycliphilus denitrificans	
E8TW89	enoyl-CoA hydratase	XII	Alicycliphilus denitrificans	
E8U1Z0	enoyl-CoA hydratase	XII	Alicycliphilus denitrificans	
C8WUH5	enoyl-CoA hydratase	XII	Alicyclobacillus acidocaldarius	
A8MFU2	enoyl-CoA hydratase	XII	Alkaliphilus oremlandii	
A8TMQ5	enoyl-CoA hydratase	XII	alpha proteobacterium	
B3ERB0	enoyl-CoA hydratase	XII	Amoebophilus asiaticus	
G0FL34	enoyl-CoA hydratase	XII	Amycolatopsis mediterranei	
G0FIJ4	enoyl-CoA hydratase	XII	Amycolatopsis mediterranei	4.2.1.17
F0GYE4	enoyl-CoA hydratase	XII	Anaerococcus hydrogenalis	4.2.1.55

Table S2.5. (continuation)

UniProt	Family [3]	Cluster	Species	EC Number
A0A095Y8D2	enoyl-CoA hydratase	XII	Anaerococcus lactolyticus	
C2CKM2	enoyl-CoA hydratase	XII	Anaerococcus tetradius	4.2.1.55
B1CB74	enoyl-CoA hydratase	XII	Anaerofustis stercorihominis	
Q2IID6	enoyl-CoA hydratase	XII	Anaeromyxobacter dehalogenans	4.2.1.17
Q2IIF8	enoyl-CoA hydratase	XII	Anaeromyxobacter dehalogenans	4.2.1.17
A7HC92	enoyl-CoA hydratase	XII	Anaeromyxobacter sp.	
A7HCC1	enoyl-CoA hydratase	XII	Anaeromyxobacter sp.	
E5VWX4	enoyl-CoA hydratase	XII	Anaerostipes sp.	
B0PHX6	enoyl-CoA hydratase	XII	Anaerotruncus colihominis	
Q7QHU0	enoyl-CoA hydratase	XII	Anopheles gambiae	
B7GGZ2	enoyl-CoA hydratase	XII	Anoxybacillus flavithermus	
A0A088A241	enoyl-CoA hydratase	XII	Apis mellifera	
A0A075WI62	enoyl-CoA hydratase	XII	Archaeoglobus fulgidus	4.2.1.17; 4.2.1.5
A0A075WCV2	enoyl-CoA hydratase	XII	Archaeoglobus fulgidus	4.2.1.55
Q5P040	enoyl-CoA hydratase	XII	Aromatoleum aromaticum	
Q5P0L7	enoyl-CoA hydratase	XII	Aromatoleum aromaticum	
Q5P691	enoyl-CoA hydratase	XII	Aromatoleum aromaticum	
Q5P5S6	enoyl-CoA hydratase	XII	Aromatoleum aromaticum	4.2.1.17
E1VRN7	enoyl-CoA hydratase	XII	Arthrobacter arilaitensis	
A2QDJ5	enoyl-CoA hydratase	XII	Aspergillus niger	4.2.1.17
Q0CNQ0	enoyl-CoA hydratase	XII	Aspergillus terreus	
A1K8G0	enoyl-CoA hydratase	XII	Azoarcus sp.	4.2.1.17
Q70JY2	enoyl-CoA hydratase	XII	Bacillus amyloliquefaciens	
Q81YG6	enoyl-CoA hydratase	XII	Bacillus anthracis	
E6U0G2	enoyl-CoA hydratase	XII	Bacillus cellulosilyticus	
C2QDA0	enoyl-CoA hydratase	XII	Bacillus cereus	
C2S3W8	enoyl-CoA hydratase	XII	Bacillus cereus	
C2UX38	enoyl-CoA hydratase	XII	Bacillus cereus	
C2WFT3	enoyl-CoA hydratase	XII	Bacillus cereus	
J8G3Z4	enoyl-CoA hydratase	XII	Bacillus cereus	
Q737U4	enoyl-CoA hydratase	XII	Bacillus cereus	
Q63B38	enoyl-CoA hydratase	XII	Bacillus cereus	4.2.1.55
Q5WEK4	enoyl-CoA hydratase	XII	Bacillus clausii	4.2.1.17
Q5WHW1	enoyl-CoA hydratase	XII	Bacillus clausii	4.2.1.17
G2TJ17	enoyl-CoA hydratase	XII	Bacillus coagulans	
G2TK60	enoyl-CoA hydratase	XII	Bacillus coagulans	
G2TKQ7	enoyl-CoA hydratase	XII	Bacillus coagulans	
A7GPP7	enoyl-CoA hydratase	XII	Bacillus cytotoxicus	
A7GTI6	enoyl-CoA hydratase	XII	Bacillus cytotoxicus	
Q9K8A5	enoyl-CoA hydratase	XII	Bacillus halodurans	
Q9KBD2	enoyl-CoA hydratase	XII	Bacillus halodurans	
U5L9T5	enoyl-CoA hydratase	XII	Bacillus infantis	
Q65IX2	enoyl-CoA hydratase	XII	Bacillus licheniformis	
T5HPQ2	enoyl-CoA hydratase	XII	Bacillus licheniformis	
T5HG93	enoyl-CoA hydratase	XII	Bacillus licheniformis	4.2.1.17
A0A0B6ALV3	enoyl-CoA hydratase	XII	Bacillus megaterium	
D5DRC3	enoyl-CoA hydratase	XII	Bacillus megaterium	4.2.1.17
D5DS18	enoyl-CoA hydratase	XII	Bacillus megaterium	4.2.1.17
A7Z7E2	enoyl-CoA hydratase	XII	Bacillus methylotrophicus	4.2.1.17
D3FWG6	enoyl-CoA hydratase	XII	Bacillus pseudofirmus	
D3FY04	enoyl-CoA hydratase	XII	Bacillus pseudofirmus	
C3BGP9	enoyl-CoA hydratase	XII	Bacillus pseudomycoides	
A0A0C2T6Q4	enoyl-CoA hydratase	XII	Bacillus pumilus	4.2.1.17
D6XSX0	enoyl-CoA hydratase	XII	Bacillus selenitireducens	
E5WJ89	enoyl-CoA hydratase	XII	Bacillus sp.	
A3I803	enoyl-CoA hydratase	XII	Bacillus sp.	4.2.1.17

Table S2.5. (continuation)

UniProt	Family [3]	Cluster	Species	EC Number
Q2B6Q5	enoyl-CoA hydratase	XII	Bacillus sp.	4.2.1.17
A0A080UE48	enoyl-CoA hydratase	XII	Bacillus subtilis	
A0A080ULR1	enoyl-CoA hydratase	XII	Bacillus subtilis	4.2.1.116
P94549	enoyl-CoA hydratase	XII	Bacillus subtilis	4.2.1.17
C3G3A8	enoyl-CoA hydratase	XII	Bacillus thuringiensis	
C3GJ82	enoyl-CoA hydratase	XII	Bacillus thuringiensis	
C3HA95	enoyl-CoA hydratase	XII	Bacillus thuringiensis	
Q6MLZ9	enoyl-CoA hydratase	XII	Bdellovibrio bacteriovorus	4.2.1.17
E5Y1N3	enoyl-CoA hydratase	XII	Bilophila wadsworthia	
A0A063UQP6	enoyl-CoA hydratase	XII	Bordetella bronchiseptica	
Q7W0Z7	enoyl-CoA hydratase	XII	Bordetella parapertussis	
Q7W6S6	enoyl-CoA hydratase	XII	Bordetella parapertussis	
Q7WBJ9	enoyl-CoA hydratase	XII	Bordetella parapertussis	
K0MJU5	enoyl-CoA hydratase	XII	Bordetella parapertussis	4.2.1.17
A0A0A0XRN5	enoyl-CoA hydratase	XII	Bordetella pertussis	
A0A0A0XHI0	enoyl-CoA hydratase	XII	Bordetella pertussis	4.2.1.17
A0A0A0XLA3	enoyl-CoA hydratase	XII	Bordetella pertussis	4.2.1.55
A9HVK9	enoyl-CoA hydratase	XII	Bordetella petrii	4.2.1.-
A9IC00	enoyl-CoA hydratase	XII	Bordetella petrii	4.2.1.17
Q2TBT3	enoyl-CoA hydratase	XII	Bos taurus	
G2YES4	enoyl-CoA hydratase	XII	Botryotinia fuckeliana	
C7MHC6	enoyl-CoA hydratase	XII	Brachybacterium faecium	4.2.1.17
D5U563	enoyl-CoA hydratase	XII	Brachyspira murdochii	
D8IC34	enoyl-CoA hydratase	XII	Brachyspira pilosicoli	
Q89CF3	enoyl-CoA hydratase	XII	Bradyrhizobium diazoefficiens	
Q89GI0	enoyl-CoA hydratase	XII	Bradyrhizobium diazoefficiens	
Q89N92	enoyl-CoA hydratase	XII	Bradyrhizobium diazoefficiens	
Q89PE5	enoyl-CoA hydratase	XII	Bradyrhizobium diazoefficiens	
A5ELP7	enoyl-CoA hydratase	XII	Bradyrhizobium sp.	4.2.1.-
A4YSQ7	enoyl-CoA hydratase	XII	Bradyrhizobium sp.	4.2.1.17
A4Z0G8	enoyl-CoA hydratase	XII	Bradyrhizobium sp.	4.2.1.17
A5ECV2	enoyl-CoA hydratase	XII	Bradyrhizobium sp.	4.2.1.17
A5EF30	enoyl-CoA hydratase	XII	Bradyrhizobium sp.	4.2.1.17
A5EHM2	enoyl-CoA hydratase	XII	Bradyrhizobium sp.	4.2.1.17
A5ES32	enoyl-CoA hydratase	XII	Bradyrhizobium sp.	4.2.1.17
C0ZDH2	enoyl-CoA hydratase	XII	Brevibacillus brevis	
C0Z9H6	enoyl-CoA hydratase	XII	Brevibacillus brevis	4.2.1.17
C0ZD52	enoyl-CoA hydratase	XII	Brevibacillus brevis	4.2.1.55
D4YK28	enoyl-CoA hydratase	XII	Brevibacterium mcbrellneri	4.2.1.55
D0P9P2	enoyl-CoA hydratase	XII	Brucella suis	
B1T454	enoyl-CoA hydratase	XII	Burkholderia ambifaria	
B1FU95	enoyl-CoA hydratase	XII	Burkholderia graminis	
B1G492	enoyl-CoA hydratase	XII	Burkholderia graminis	
B1G4W8	enoyl-CoA hydratase	XII	Burkholderia graminis	
B1G6E6	enoyl-CoA hydratase	XII	Burkholderia graminis	
B1G9J2	enoyl-CoA hydratase	XII	Burkholderia graminis	
B1GB42	enoyl-CoA hydratase	XII	Burkholderia graminis	
Q396R1	enoyl-CoA hydratase	XII	Burkholderia lata	4.2.1.17
B9B5S5	enoyl-CoA hydratase	XII	Burkholderia multivorans	4.2.1.55
B2JSZ1	enoyl-CoA hydratase	XII	Burkholderia phymatum	
B2TCN9	enoyl-CoA hydratase	XII	Burkholderia phytofirmans	
B5WFJ7	enoyl-CoA hydratase	XII	Burkholderia sp.	
B5WH72	enoyl-CoA hydratase	XII	Burkholderia sp.	
B5WJ29	enoyl-CoA hydratase	XII	Burkholderia sp.	
D5WHX0	enoyl-CoA hydratase	XII	Burkholderia sp.	
E1T9N9	enoyl-CoA hydratase	XII	Burkholderia sp.	

Table S2.5. (continuation)

UniProt	Family [3]	Cluster	Species	EC Number
E1TKB8	enoyl-CoA hydratase	XII	Burkholderia sp.	
A4JMN3	enoyl-CoA hydratase	XII	Burkholderia vietnamiensis	4.2.1.17
Q13GJ1	enoyl-CoA hydratase	XII	Burkholderia xenovorans	
Q13G87	enoyl-CoA hydratase	XII	Burkholderia xenovorans	4.2.1.17
Q13GQ5	enoyl-CoA hydratase	XII	Burkholderia xenovorans	4.2.1.17
Q13H98	enoyl-CoA hydratase	XII	Burkholderia xenovorans	4.2.1.17
D7GUE1	enoyl-CoA hydratase	XII	butyrate-producing bacterium	4.2.1.17; 4.2.1.5
D7GUN0	enoyl-CoA hydratase	XII	butyrate-producing bacterium	4.2.1.55
D4RW87	enoyl-CoA hydratase	XII	Butyrivibrio crossotus	4.2.1.55
Q65Y12	enoyl-CoA hydratase	XII	Butyrivibrio fibrisolvens	
D4IYU6	enoyl-CoA hydratase	XII	Butyrivibrio fibrisolvens	4.2.1.55
E0RVR2	enoyl-CoA hydratase	XII	Butyrivibrio proteoclasticus	4.2.1.17
A8XEB9	enoyl-CoA hydratase	XII	Caenorhabditis briggsae	
Q8RC92	enoyl-CoA hydratase	XII	Caldanaerobacter subterraneus	
E4TK04	enoyl-CoA hydratase	XII	Calditerrivibrio nitroreducens	4.2.1.17; 4.2.1.5
E6N9F0	enoyl-CoA hydratase	XII	Candidatus Caldiarchaeum	4.2.1.17
Q1Q7B4	enoyl-CoA hydratase	XII	Candidatus Kuenenia	4.2.1.17
C7DGQ7	enoyl-CoA hydratase	XII	Candidatus Micrarchaeum	
C7DHK3	enoyl-CoA hydratase	XII	Candidatus Micrarchaeum	
Q3ABC5	enoyl-CoA hydratase	XII	Carboxydothermus hydrog.	
Q3ABQ5	enoyl-CoA hydratase	XII	Carboxydothermus hydrog.	4.2.1.55
Q3ACK7	enoyl-CoA hydratase	XII	Carboxydothermus hydrog.	4.2.1.55
A8U751	enoyl-CoA hydratase	XII	Carnobacterium sp.	4.2.1.17
C7Q3R1	enoyl-CoA hydratase	XII	Catenulispora acidiphila	
C7Q7B8	enoyl-CoA hydratase	XII	Catenulispora acidiphila	
D5VF88	enoyl-CoA hydratase	XII	Caulobacter segnis	
B0T5M5	enoyl-CoA hydratase	XII	Caulobacter sp.	
E6XCN0	enoyl-CoA hydratase	XII	Cellulophaga algicola	4.2.1.17; 4.2.1.5
W7R144	enoyl-CoA hydratase	XII	Cellulophaga geojensis	
A0RTZ4	enoyl-CoA hydratase	XII	Cenarchaeum symbiosum	4.2.1.55
Q11AS3	enoyl-CoA hydratase	XII	Chelativorans sp.	
Q11E52	enoyl-CoA hydratase	XII	Chelativorans sp.	4.2.1.17
C7PDB6	enoyl-CoA hydratase	XII	Chitinophaga pinensis	
E1ZT47	enoyl-CoA hydratase	XII	Chlorella variabilis	
Q7NXS3	enoyl-CoA hydratase	XII	Chromobacterium violaceum	4.2.1.17; 5.3.3.8
D7VWY8	enoyl-CoA hydratase	XII	Chryseobacterium gleum	
D0D9P4	enoyl-CoA hydratase	XII	Citreicella sp.	
D8WWH8	enoyl-CoA hydratase	XII	Clostridia bacterium	
D8WWI4	enoyl-CoA hydratase	XII	Clostridia bacterium	
D8WWT5	enoyl-CoA hydratase	XII	Clostridia bacterium	
C5EFU7	enoyl-CoA hydratase	XII	Clostridiales bacterium	
C5EKS1	enoyl-CoA hydratase	XII	Clostridiales bacterium	
P52046	enoyl-CoA hydratase	XII	Clostridium acetobutylicum	4.2.1.150
A6M223	enoyl-CoA hydratase	XII	Clostridium beijerinckii	
Q8RMI7	enoyl-CoA hydratase	XII	Clostridium beijerinckii	4.2.1.17
A8RJI0	enoyl-CoA hydratase	XII	Clostridium bolteae	
A0A0C2SHJ9	enoyl-CoA hydratase	XII	Clostridium botulinum	
A0A0E1KTK7	enoyl-CoA hydratase	XII	Clostridium botulinum	
B1B8W4	enoyl-CoA hydratase	XII	Clostridium botulinum	4.2.1.55
B1BB95	enoyl-CoA hydratase	XII	Clostridium botulinum	4.2.1.55
B2TM66	enoyl-CoA hydratase	XII	Clostridium botulinum	4.2.1.55
W1UD01	enoyl-CoA hydratase	XII	Clostridium butyricum	
C6PR29	enoyl-CoA hydratase	XII	Clostridium carboxidivorans	
D6M9A0	enoyl-CoA hydratase	XII	Clostridium carboxidivorans	4.2.1.55
D9ST06	enoyl-CoA hydratase	XII	Clostridium cellulovorans	
A0A099IDJ0	enoyl-CoA hydratase	XII	Clostridium innocuum	

Table S2.5. (continuation)

UniProt	Family [3]	Cluster	Species	EC Number
A5N093	enoyl-CoA hydratase	XII	Clostridium kluyveri	4.2.1.55
A5N5C7	enoyl-CoA hydratase	XII	Clostridium kluyveri	4.2.1.55
B1V4H9	enoyl-CoA hydratase	XII	Clostridium perfringens	4.2.1.55
H7CZ06	enoyl-CoA hydratase	XII	Clostridium perfringens	4.2.1.55
A0A072YG47	enoyl-CoA hydratase	XII	Clostridium sp.	
G5FCH1	enoyl-CoA hydratase	XII	Clostridium sp.	
V9HC04	enoyl-CoA hydratase	XII	Clostridium sp.	
E3PXT7	enoyl-CoA hydratase	XII	Clostridium sticklandii	4.2.1.17
Q891F2	enoyl-CoA hydratase	XII	Clostridium tetani	
Q898H3	enoyl-CoA hydratase	XII	Clostridium tetani	4.2.1.55
C5P7W8	enoyl-CoA hydratase	XII	Coccidioides posadasii	
E3Q1Y8	enoyl-CoA hydratase	XII	Colletotrichum graminicola	
B7WWW9	enoyl-CoA hydratase	XII	Comamonas testosteroni	
B7X0F6	enoyl-CoA hydratase	XII	Comamonas testosteroni	
D0J7F3	enoyl-CoA hydratase	XII	Comamonas testosteroni	
A0A096G738	enoyl-CoA hydratase	XII	Comamonas testosteroni	4.2.1.17
D3F114	enoyl-CoA hydratase	XII	Conexibacter woesei	
D3F5C0	enoyl-CoA hydratase	XII	Conexibacter woesei	
D3F6J4	enoyl-CoA hydratase	XII	Conexibacter woesei	
D4JAG4	enoyl-CoA hydratase	XII	Coprococcus catus	
D4J3Y3	enoyl-CoA hydratase	XII	Coprococcus catus	4.2.1.55
D4J7C3	enoyl-CoA hydratase	XII	Coprococcus catus	4.2.1.55
C0B8K1	enoyl-CoA hydratase	XII	Coprococcus comes	4.2.1.55
D5HGH8	enoyl-CoA hydratase	XII	Coprococcus sp.	4.2.1.55
D5ELS2	enoyl-CoA hydratase	XII	Coraliomargarita akajimensis	
D5NWR2	enoyl-CoA hydratase	XII	Corynebacterium ammoniagenes	
Q8FLT4	enoyl-CoA hydratase	XII	Corynebacterium efficiens	
Q8FSR0	enoyl-CoA hydratase	XII	Corynebacterium efficiens	
A3U6F9	enoyl-CoA hydratase	XII	Croceibacter atlanticus	
Q560C1	enoyl-CoA hydratase	XII	Cryptococcus neoformans	
Q5KPC4	enoyl-CoA hydratase	XII	Cryptococcus neoformans	
Q1LD10	enoyl-CoA hydratase	XII	Cupriavidus metallidurans	4.2.1.17
Q1LFI4	enoyl-CoA hydratase	XII	Cupriavidus metallidurans	4.2.1.17
Q1LK46	enoyl-CoA hydratase	XII	Cupriavidus metallidurans	4.2.1.17
Q1LKT3	enoyl-CoA hydratase	XII	Cupriavidus metallidurans	4.2.1.17
Q0K1I8	enoyl-CoA hydratase	XII	Cupriavidus necator	4.2.1.17
Q0KAX8	enoyl-CoA hydratase	XII	Cupriavidus necator	4.2.1.17
Q46NM5	enoyl-CoA hydratase	XII	Cupriavidus pinatubonensis	
Q46QF5	enoyl-CoA hydratase	XII	Cupriavidus pinatubonensis	
Q46RT8	enoyl-CoA hydratase	XII	Cupriavidus pinatubonensis	
Q46QJ3	enoyl-CoA hydratase	XII	Cupriavidus pinatubonensis	4.2.1.17
Q46V68	enoyl-CoA hydratase	XII	Cupriavidus pinatubonensis	4.2.1.17
B3RD65	enoyl-CoA hydratase	XII	Cupriavidus taiwanensis	4.2.1.17
C9YF21	enoyl-CoA hydratase	XII	Curvibacter putative	
Q11Z55	enoyl-CoA hydratase	XII	Cytophaga hutchinsonii	4.2.1.17
Q6DC25	enoyl-CoA hydratase	XII	Danio rerio	
Q6NWF7	enoyl-CoA hydratase	XII	Danio rerio	
Q7ZUR3	enoyl-CoA hydratase	XII	Danio rerio	
D3P9Y7	enoyl-CoA hydratase	XII	Deferribacter desulfuricans	
D3P9A0	enoyl-CoA hydratase	XII	Deferribacter desulfuricans	4.2.1.55
Q1IXZ4	enoyl-CoA hydratase	XII	Deinococcus geothermalis	
E8U8N4	enoyl-CoA hydratase	XII	Deinococcus maricopensis	4.2.1.55
F0RJL4	enoyl-CoA hydratase	XII	Deinococcus proteolyticus	4.2.1.55
Q9RV78	enoyl-CoA hydratase	XII	Deinococcus radiodurans	
A9BS38	enoyl-CoA hydratase	XII	Delftia acidovorans	
A0A031IN97	enoyl-CoA hydratase	XII	Delftia sp.	

Table S2.5. (continuation)

UniProt	Family [3]	Cluster	Species	EC Number
D8F747	enoyl-CoA hydratase	XII	delta proteobacterium	
D8F7G8	enoyl-CoA hydratase	XII	delta proteobacterium	
D8F7H9	enoyl-CoA hydratase	XII	delta proteobacterium	
D8F7I8	enoyl-CoA hydratase	XII	delta proteobacterium	
D8FBL3	enoyl-CoA hydratase	XII	delta proteobacterium	
D8F862	enoyl-CoA hydratase	XII	delta proteobacterium	4.2.1.55
D4H6K9	enoyl-CoA hydratase	XII	Denitrovibrio acetiphilus	
A0A075JER8	enoyl-CoA hydratase	XII	Dermacoccus nishinomiyaensis	
E1QJR6	enoyl-CoA hydratase	XII	Desulfarculus baarsii	
E1QGG7	enoyl-CoA hydratase	XII	Desulfarculus baarsii	4.2.1.55
B8FBX2	enoyl-CoA hydratase	XII	Desulfatibacillum alkenivorans	
B8FKF4	enoyl-CoA hydratase	XII	Desulfatibacillum alkenivorans	
B8FIU2	enoyl-CoA hydratase	XII	Desulfatibacillum alkenivorans	4.2.1.17
B8FJA9	enoyl-CoA hydratase	XII	Desulfatibacillum alkenivorans	4.2.1.17
B8FWP0	enoyl-CoA hydratase	XII	Desulfitobacterium hafniense	
Q24UL4	enoyl-CoA hydratase	XII	Desulfitobacterium hafniense	
A0A098AYS4	enoyl-CoA hydratase	XII	Desulfitobacterium hafniense	4.2.1.17
C0QCY8	enoyl-CoA hydratase	XII	Desulfobacterium autotrophicum	4.2.1.55
C0QFR9	enoyl-CoA hydratase	XII	Desulfobacterium autotrophicum	4.2.1.55
C0QHK2	enoyl-CoA hydratase	XII	Desulfobacterium autotrophicum	4.2.1.55
E8RDP9	enoyl-CoA hydratase	XII	Desulfobulbus propionicus	4.2.1.17
A8ZVI2	enoyl-CoA hydratase	XII	Desulfococcus oleovorans	
A8ZW96	enoyl-CoA hydratase	XII	Desulfococcus oleovorans	
A9A007	enoyl-CoA hydratase	XII	Desulfococcus oleovorans	
C8VWY4	enoyl-CoA hydratase	XII	Desulfotomaculum acetoxidans	
A4J5F3	enoyl-CoA hydratase	XII	Desulfotomaculum reducens	
A4J1J6	enoyl-CoA hydratase	XII	Desulfotomaculum reducens	4.2.1.17
A4J4L9	enoyl-CoA hydratase	XII	Desulfotomaculum reducens	4.2.1.17
E6W696	enoyl-CoA hydratase	XII	Desulfurispirillum indicum	
Q1K2C7	enoyl-CoA hydratase	XII	Desulfuromonas acetoxidans	
C0GEG5	enoyl-CoA hydratase	XII	Dethiobacter alkaliphilus	
C0GGI8	enoyl-CoA hydratase	XII	Dethiobacter alkaliphilus	
C0GKR8	enoyl-CoA hydratase	XII	Dethiobacter alkaliphilus	
A8LT87	enoyl-CoA hydratase	XII	Dinoroseobacter shibae	
F0HQ19	enoyl-CoA hydratase	XII	Eggerthella sp.	4.2.1.55
Q5B984	enoyl-CoA hydratase	XII	Emericella nidulans	
E4KQP5	enoyl-CoA hydratase	XII	Eremococcus coleocola	4.2.1.55
E4KQQ0	enoyl-CoA hydratase	XII	Eremococcus coleocola	4.2.1.55
E4KRB8	enoyl-CoA hydratase	XII	Eremococcus coleocola	4.2.1.55
B2VIL0	enoyl-CoA hydratase	XII	Erwinia tasmaniensis	4.2.1.17
U5F896	enoyl-CoA hydratase	XII	Erysipelotrichaceae bacterium	
T6H161	enoyl-CoA hydratase	XII	Escherichia coli	
E3GGC4	enoyl-CoA hydratase	XII	Eubacterium limosum	
E3GIR9	enoyl-CoA hydratase	XII	Eubacterium limosum	
C7GZN1	enoyl-CoA hydratase	XII	Eubacterium saphenum	4.2.1.55
A5Z9I6	enoyl-CoA hydratase	XII	Eubacterium ventriosum	4.2.1.55
B1YIJ9	enoyl-CoA hydratase	XII	Exiguobacterium sibiricum	
E2ZH38	enoyl-CoA hydratase	XII	Faecalibacterium cf.	4.2.1.55
S0AM24	enoyl-CoA hydratase	XII	Ferroplasma acidarmanus	
D6GPX4	enoyl-CoA hydratase	XII	Filifactor alocis	
A3J3F6	enoyl-CoA hydratase	XII	Flavobacteria bacterium	
C0BNL2	enoyl-CoA hydratase	XII	Flavobacteria bacterium	
Q26DR3	enoyl-CoA hydratase	XII	Flavobacteria bacterium	4.2.1.17
Q26GS7	enoyl-CoA hydratase	XII	Flavobacteria bacterium	4.2.1.17
C6WZV7	enoyl-CoA hydratase	XII	Flavobacteriaceae bacterium	4.2.1.17
A8UIF0	enoyl-CoA hydratase	XII	Flavobacteriales bacterium	

Table S2.5. (continuation)

UniProt	Family [3]	Cluster	Species	EC Number
A5FLF7	enoyl-CoA hydratase	XII	Flavobacterium johnsoniae	
A0A076NWA0	enoyl-CoA hydratase	XII	Flavobacterium psychrophilum	4.2.1.55
Q0RU73	enoyl-CoA hydratase	XII	Frankia alni	4.2.1.-
Q0RL25	enoyl-CoA hydratase	XII	Frankia alni	4.2.1.17
Q0RL52	enoyl-CoA hydratase	XII	Frankia alni	4.2.1.17
A8LEG9	enoyl-CoA hydratase	XII	Frankia sp.	
D3CYX8	enoyl-CoA hydratase	XII	Frankia sp.	
E3IYR8	enoyl-CoA hydratase	XII	Frankia sp.	
E3J3W8	enoyl-CoA hydratase	XII	Frankia sp.	
E3JDC7	enoyl-CoA hydratase	XII	Frankia sp.	
F8B4I6	enoyl-CoA hydratase	XII	Frankia symbiont	
E5BH60	enoyl-CoA hydratase	XII	Fusobacterium gonidiaformans	4.2.1.55
C3WBI0	enoyl-CoA hydratase	XII	Fusobacterium mortiferum	4.2.1.55
A0A064AJB0	enoyl-CoA hydratase	XII	Fusobacterium necrophorum	
A0A0D6G4R5	enoyl-CoA hydratase	XII	Fusobacterium nucleatum	4.2.1.17
D5RAC1	enoyl-CoA hydratase	XII	Fusobacterium nucleatum	4.2.1.55
D4CYJ9	enoyl-CoA hydratase	XII	Fusobacterium periodonticum	4.2.1.55
C6JKT4	enoyl-CoA hydratase	XII	Fusobacterium varium	4.2.1.55
B8KNS8	enoyl-CoA hydratase	XII	gamma proteobacterium	
Q1YP77	enoyl-CoA hydratase	XII	gamma proteobacterium	
C1A6J8	enoyl-CoA hydratase	XII	Gemmatimonas aurantiaca	4.2.1.17
Q5KZJ9	enoyl-CoA hydratase	XII	Geobacillus kaustophilus	
Q5QL51	enoyl-CoA hydratase	XII	Geobacillus kaustophilus	
Q5KYF9	enoyl-CoA hydratase	XII	Geobacillus kaustophilus	4.2.1.17
A0A0E0TBN5	enoyl-CoA hydratase	XII	Geobacillus sp.	
A0A0F6BNY1	enoyl-CoA hydratase	XII	Geobacillus sp.	
C5D5Q3	enoyl-CoA hydratase	XII	Geobacillus sp.	
T0NZ09	enoyl-CoA hydratase	XII	Geobacillus sp.	
V6VI07	enoyl-CoA hydratase	XII	Geobacillus sp.	
A4INB7	enoyl-CoA hydratase	XII	Geobacillus thermodenitrificans	
B5E7U4	enoyl-CoA hydratase	XII	Geobacter bemidjiensis	
B9LZZ6	enoyl-CoA hydratase	XII	Geobacter daltonii	
B9M432	enoyl-CoA hydratase	XII	Geobacter daltonii	
B9M8K1	enoyl-CoA hydratase	XII	Geobacter daltonii	
B9M959	enoyl-CoA hydratase	XII	Geobacter daltonii	
B3E839	enoyl-CoA hydratase	XII	Geobacter lovleyi	
Q39TI5	enoyl-CoA hydratase	XII	Geobacter metallireducens	
Q39TJ0	enoyl-CoA hydratase	XII	Geobacter metallireducens	
Q39TJ3	enoyl-CoA hydratase	XII	Geobacter metallireducens	
Q39TX4	enoyl-CoA hydratase	XII	Geobacter metallireducens	
Q39TY8	enoyl-CoA hydratase	XII	Geobacter metallireducens	
Q39UX7	enoyl-CoA hydratase	XII	Geobacter metallireducens	
Q39VC0	enoyl-CoA hydratase	XII	Geobacter metallireducens	
C6E212	enoyl-CoA hydratase	XII	Geobacter sp.	
C6E4E1	enoyl-CoA hydratase	XII	Geobacter sp.	
E8WNM3	enoyl-CoA hydratase	XII	Geobacter sp.	
E8WNW0	enoyl-CoA hydratase	XII	Geobacter sp.	
Q74DD9	enoyl-CoA hydratase	XII	Geobacter sulfurreducens	
A5G5Z5	enoyl-CoA hydratase	XII	Geobacter uraniireducens	4.2.1.17
D2S5B2	enoyl-CoA hydratase	XII	Geodermatophilus obscurus	
D2SEM2	enoyl-CoA hydratase	XII	Geodermatophilus obscurus	
A0A016PAS7	enoyl-CoA hydratase	XII	Gibberella zeae	
A0A016Q5X5	enoyl-CoA hydratase	XII	Gibberella zeae	
A9H4Y1	enoyl-CoA hydratase	XII	Gluconacetobacter diazotrophicus	
C6THP6	enoyl-CoA hydratase	XII	Glycine max	
A0A0F2GF91	enoyl-CoA hydratase	XII	Gordonia sp.	

Table S2.5. (continuation)

UniProt	Family [3]	Cluster	Species	EC Number
A0M6B3	enoyl-CoA hydratase	XII	Gramella forsetii	4.2.1.55
G8NQ56	enoyl-CoA hydratase	XII	Granulicella mallensis	4.2.1.17
E8WZL5	enoyl-CoA hydratase	XII	Granulicella tundricola	
Q2SC94	enoyl-CoA hydratase	XII	Hahella chejuensis	
E7QPV2	enoyl-CoA hydratase	XII	Haladaptatus paucihalophilus	
E7QT99	enoyl-CoA hydratase	XII	Haladaptatus paucihalophilus	
D8J8B4	enoyl-CoA hydratase	XII	Halalkalicoccus jeotgali	
D8JB26	enoyl-CoA hydratase	XII	Halalkalicoccus jeotgali	
D8JB43	enoyl-CoA hydratase	XII	Halalkalicoccus jeotgali	
D8JCV9	enoyl-CoA hydratase	XII	Halalkalicoccus jeotgali	
E4RP32	enoyl-CoA hydratase	XII	Halanaerobium hydrog.	
E3DM10	enoyl-CoA hydratase	XII	Halanaerobium praevalens	4.2.1.17
D0LYC9	enoyl-CoA hydratase	XII	Haliangium ochraceum	
M0JLL0	enoyl-CoA hydratase	XII	Haloarcula sinaiiensis	
E1WXZ2	enoyl-CoA hydratase	XII	Halobacteriovorax marinus	
B0R3C3	enoyl-CoA hydratase	XII	Halobacterium salinarum	4.2.1.17
L5NZ25	enoyl-CoA hydratase	XII	Haloferax sp.	
D4GXV2	enoyl-CoA hydratase	XII	Haloferax volcanii	4.2.1.17
E4NN03	enoyl-CoA hydratase	XII	Halogeometricum borinquense	4.2.1.17
E4NWD0	enoyl-CoA hydratase	XII	Halogeometricum borinquense	4.2.1.17
E1V567	enoyl-CoA hydratase	XII	Halomonas elongata	4.2.1.55
Q18HM3	enoyl-CoA hydratase	XII	Haloquadratum walsbyi	4.2.1.17
C7NQM2	enoyl-CoA hydratase	XII	Halorhabdus utahensis	
B9LMX3	enoyl-CoA hydratase	XII	Halorubrum lacusprofundi	
D2RUQ1	enoyl-CoA hydratase	XII	Haloterrigena turkmenica	
D2RZW8	enoyl-CoA hydratase	XII	Haloterrigena turkmenica	
D2S0U5	enoyl-CoA hydratase	XII	Haloterrigena turkmenica	
D2S0W2	enoyl-CoA hydratase	XII	Haloterrigena turkmenica	
D2S0W5	enoyl-CoA hydratase	XII	Haloterrigena turkmenica	
D2S385	enoyl-CoA hydratase	XII	Haloterrigena turkmenica	
B0TAM0	enoyl-CoA hydratase	XII	Heliobacterium modesticaldum	
D8ISR1	enoyl-CoA hydratase	XII	Herbaspirillum seropedicae	4.2.1.17
A9AYA5	enoyl-CoA hydratase	XII	Herpetosiphon aurantiacus	
A9B0Y0	enoyl-CoA hydratase	XII	Herpetosiphon aurantiacus	
C6XPV3	enoyl-CoA hydratase	XII	Hirschia baltica	
A9CZD8	enoyl-CoA hydratase	XII	Hoeflea phototrophica	4.2.1.17
B7CAJ3	enoyl-CoA hydratase	XII	Holdemanella biformis	4.2.1.55
Q86YB7	enoyl-CoA hydratase	XII	Homo sapiens	
E3HAR4	enoyl-CoA hydratase	XII	Ilyobacter polytropus	4.2.1.55
E3HE39	enoyl-CoA hydratase	XII	Ilyobacter polytropus	4.2.1.55
E6S821	enoyl-CoA hydratase	XII	Intrasporangium calvum	4.2.1.17
E6SFX3	enoyl-CoA hydratase	XII	Intrasporangium calvum	4.2.1.17
E8R5K7	enoyl-CoA hydratase	XII	Isosphaera pallida	
A3TR04	enoyl-CoA hydratase	XII	Janibacter sp.	
Q28UN0	enoyl-CoA hydratase	XII	Jannaschia sp.	
A6WB93	enoyl-CoA hydratase	XII	Kineococcus radiotolerans	
B2GHL8	enoyl-CoA hydratase	XII	Kocuria rhizophila	4.2.1.17
A9DN11	enoyl-CoA hydratase	XII	Kordia algicida	
Q1ISD5	enoyl-CoA hydratase	XII	Koribacter versatilis	4.2.1.17
D2Q1Z9	enoyl-CoA hydratase	XII	Kribbella flavida	
D5WUF6	enoyl-CoA hydratase	XII	Kyrpidia tusciae	
D5WXT4	enoyl-CoA hydratase	XII	Kyrpidia tusciae	
C7NFF6	enoyl-CoA hydratase	XII	Kytococcus sedentarius	
C7NG05	enoyl-CoA hydratase	XII	Kytococcus sedentarius	
A0NW20	enoyl-CoA hydratase	XII	Labrenzia aggregata	4.2.1.17
B9QU83	enoyl-CoA hydratase	XII	Labrenzia alexandrii	

Table S2.5. (continuation)

UniProt	Family [3]	Cluster	Species	EC Number
E6LL61	enoyl-CoA hydratase	XII	Lachnoanaerobaculum saburreum	4.2.1.55
I0RAN1	enoyl-CoA hydratase	XII	Lachnoanaerobaculum saburreum	4.2.1.55
R5YYQ6	enoyl-CoA hydratase	XII	Lachnospiraceae bacterium	
C2KD03	enoyl-CoA hydratase	XII	Lactobacillus crispatus	4.2.1.55
A3XH09	enoyl-CoA hydratase	XII	Leeuwenhoekiella blandensis	
G9EMG8	enoyl-CoA hydratase	XII	Legionella drancourtii	
D3HQR8	enoyl-CoA hydratase	XII	Legionella longbeachae	4.2.1.17
Q5WY38	enoyl-CoA hydratase	XII	Legionella pneumophila	
A4HM74	enoyl-CoA hydratase	XII	Leishmania braziliensis	
E9AEL3	enoyl-CoA hydratase	XII	Leishmania major	
B0SMA7	enoyl-CoA hydratase	XII	Leptospira biflexa	4.2.1.55
Q04WA5	enoyl-CoA hydratase	XII	Leptospira borgpetersenii	
M6GD87	enoyl-CoA hydratase	XII	Leptospira interrogans	
B1Y362	enoyl-CoA hydratase	XII	Leptothrix cholodnii	
D7WMI6	enoyl-CoA hydratase	XII	Lysinibacillus fusiformis	
D7WPZ0	enoyl-CoA hydratase	XII	Lysinibacillus fusiformis	4.2.1.17
B1HNI6	enoyl-CoA hydratase	XII	Lysinibacillus sphaericus	
G4MZ24	enoyl-CoA hydratase	XII	Magnaporthe oryzae	
A4ANR3	enoyl-CoA hydratase	XII	Maribacter sp.	
A4AJA9	enoyl-CoA hydratase	XII	marine actinobacterium	
A4ALU7	enoyl-CoA hydratase	XII	marine actinobacterium	
A0YEC0	enoyl-CoA hydratase	XII	marine gamma	
A0Z5F2	enoyl-CoA hydratase	XII	marine gamma	
B7RV29	enoyl-CoA hydratase	XII	marine gamma	
B7RWE9	enoyl-CoA hydratase	XII	marine gamma	
A0Z2J2	enoyl-CoA hydratase	XII	marine gamma	4.2.1.17
E4PI71	enoyl-CoA hydratase	XII	Marinobacter adhaerens	
A3J9M7	enoyl-CoA hydratase	XII	Marinobacter sp.	
A3Y533	enoyl-CoA hydratase	XII	Marinomonas sp.	
A3Y537	enoyl-CoA hydratase	XII	Marinomonas sp.	
A3Y686	enoyl-CoA hydratase	XII	Marinomonas sp.	
A3YAH8	enoyl-CoA hydratase	XII	Marinomonas sp.	
A3Y9Y9	enoyl-CoA hydratase	XII	Marinomonas sp.	4.2.1.17
A3VE87	enoyl-CoA hydratase	XII	Maritimibacter alkaliphilus	
A3VIJ7	enoyl-CoA hydratase	XII	Maritimibacter alkaliphilus	
A3VD01	enoyl-CoA hydratase	XII	Maritimibacter alkaliphilus	4.2.1.17
E4TL21	enoyl-CoA hydratase	XII	Marivirga tractuosa	4.2.1.17
D3LX36	enoyl-CoA hydratase	XII	Megasphaera genomosp.	4.2.1.55
E2Z9G7	enoyl-CoA hydratase	XII	Megasphaera micronuciformis	4.2.1.55
D3PLE5	enoyl-CoA hydratase	XII	Meiothermus ruber	
D3PSX2	enoyl-CoA hydratase	XII	Meiothermus ruber	
D7BH90	enoyl-CoA hydratase	XII	Meiothermus silvanus	4.2.1.55
E8TDX1	enoyl-CoA hydratase	XII	Mesorhizobium ciceri	
E8TJL2	enoyl-CoA hydratase	XII	Mesorhizobium ciceri	
F7Y098	enoyl-CoA hydratase	XII	Mesorhizobium opportunistum	
F7Y219	enoyl-CoA hydratase	XII	Mesorhizobium opportunistum	
A4YDL1	enoyl-CoA hydratase	XII	Metallosphaera sedula	
A4YI89	enoyl-CoA hydratase	XII	Metallosphaera sedula	4.2.1.116
Q6TN86	enoyl-CoA hydratase	XII	Methylobacterium extorquens	
B8IC52	enoyl-CoA hydratase	XII	Methylobacterium nodulans	
E8N9A0	enoyl-CoA hydratase	XII	Microbacterium testaceum	
D9T059	enoyl-CoA hydratase	XII	Micromonospora aurantiaca	
D9TE79	enoyl-CoA hydratase	XII	Micromonospora aurantiaca	
C4RB42	enoyl-CoA hydratase	XII	Micromonospora sp.	
A1ZQE7	enoyl-CoA hydratase	XII	Microscilla marina	4.2.1.55
H1Y2G5	enoyl-CoA hydratase	XII	Mucilaginibacter paludis	

Table S2.5. (continuation)

UniProt	Family [3]	Cluster	Species	EC Number
Q3TLP5	enoyl-CoA hydratase	XII	Mus musculus	
V7JJ13	enoyl-CoA hydratase	XII	Mycobacterium avium	4.2.1.17
V7JP66	enoyl-CoA hydratase	XII	Mycobacterium avium	4.2.1.17
X8E443	enoyl-CoA hydratase	XII	Mycobacterium chelonae	
X8ECP9	enoyl-CoA hydratase	XII	Mycobacterium chelonae	
E6TIF4	enoyl-CoA hydratase	XII	Mycobacterium gilvum	
E6TMD2	enoyl-CoA hydratase	XII	Mycobacterium gilvum	
X7ZAE6	enoyl-CoA hydratase	XII	Mycobacterium kansasii	
B2HJY7	enoyl-CoA hydratase	XII	Mycobacterium marinum	
B2HKK9	enoyl-CoA hydratase	XII	Mycobacterium marinum	
D5P5N3	enoyl-CoA hydratase	XII	Mycobacterium parascrofulaceum	4.2.1.17
Q73VC7	enoyl-CoA hydratase	XII	Mycobacterium paratuberculosis	4.2.1.17
A0R765	enoyl-CoA hydratase	XII	Mycobacterium smegmatis	
A0QZM2	enoyl-CoA hydratase	XII	Mycobacterium smegmatis	4.2.1.-
A0QPR5	enoyl-CoA hydratase	XII	Mycobacterium smegmatis	4.2.1.17
A0QXN1	enoyl-CoA hydratase	XII	Mycobacterium smegmatis	4.2.1.17
A0QZV6	enoyl-CoA hydratase	XII	Mycobacterium smegmatis	4.2.1.17
A1UD87	enoyl-CoA hydratase	XII	Mycobacterium sp.	
A2VNK5	enoyl-CoA hydratase	XII	Mycobacterium tuberculosis	
D6FKT4	enoyl-CoA hydratase	XII	Mycobacterium tuberculosis	
A0PLL1	enoyl-CoA hydratase	XII	Mycobacterium ulcerans	
Q1D5U2	enoyl-CoA hydratase	XII	Myxococcus xanthus	4.2.1.55
Q1D5Y4	enoyl-CoA hydratase	XII	Myxococcus xanthus	4.2.1.55
C8XAB9	enoyl-CoA hydratase	XII	Nakamurella multipartita	
A0A0B4J2T1	enoyl-CoA hydratase	XII	Nasonia vitripennis	
B2A632	enoyl-CoA hydratase	XII	Natranaerobius thermophilus	
D3T037	enoyl-CoA hydratase	XII	Natrialba magadii	
D3T0H1	enoyl-CoA hydratase	XII	Natrialba magadii	
Q3IPZ0	enoyl-CoA hydratase	XII	Natronomonas pharaonis	4.2.1.17
Q3IR37	enoyl-CoA hydratase	XII	Natronomonas pharaonis	4.2.1.17
C7ZB84	enoyl-CoA hydratase	XII	Nectria haematococca	
A7SS28	enoyl-CoA hydratase	XII	Nematostella vectensis	
A9A2G5	enoyl-CoA hydratase	XII	Nitrosopumilus maritimus	
D8PHR1	enoyl-CoA hydratase	XII	Nitrospira defluvii	4.2.1.17
Q5YXT7	enoyl-CoA hydratase	XII	Nocardia farcinica	
E9UN25	enoyl-CoA hydratase	XII	Nocardioidaceae bacterium	
E9V023	enoyl-CoA hydratase	XII	Nocardioidaceae bacterium	
A1SCQ9	enoyl-CoA hydratase	XII	Nocardioides sp.	4.2.1.17
A1SHP0	enoyl-CoA hydratase	XII	Nocardioides sp.	4.2.1.17
A1SPQ7	enoyl-CoA hydratase	XII	Nocardioides sp.	4.2.1.17
D7AUE5	enoyl-CoA hydratase	XII	Nocardiopsis dassonvillei	4.2.1.17
A4XEX0	enoyl-CoA hydratase	XII	Novosphingobium aromat.	4.2.1.17
Q2G8G2	enoyl-CoA hydratase	XII	Novosphingobium aromat.	4.2.1.17
A3U237	enoyl-CoA hydratase	XII	Oceanicola batsensis	
A3TUR4	enoyl-CoA hydratase	XII	Oceanicola batsensis	4.2.1.17
E4U7B4	enoyl-CoA hydratase	XII	Oceanithermus profundus	4.2.1.17
Q8EPI5	enoyl-CoA hydratase	XII	Oceanobacillus iheyensis	4.2.1.17
C4WKI8	enoyl-CoA hydratase	XII	Ochrobactrum intermedium	
F9Z8C8	enoyl-CoA hydratase	XII	Odoribacter splanchnicus	4.2.1.55
B6JI41	enoyl-CoA hydratase	XII	Oligotropha carboxidovorans	
Q6H7I3	enoyl-CoA hydratase	XII	Oryza sativa	
Q6H7I4	enoyl-CoA hydratase	XII	Oryza sativa	
E1IFB9	enoyl-CoA hydratase	XII	Oscillochloris trichoides	
C6J301	enoyl-CoA hydratase	XII	Paenibacillus sp.	
C6J3H5	enoyl-CoA hydratase	XII	Paenibacillus sp.	
C0SIY5	enoyl-CoA hydratase	XII	Paracoccidioides brasiliensis	

Table S2.5. (continuation)

UniProt	Family [3]	Cluster	Species	EC Number
A1B553	enoyl-CoA hydratase	XII	Paracoccus denitrificans	
A1BC08	enoyl-CoA hydratase	XII	Paracoccus denitrificans	
A0C5H1	enoyl-CoA hydratase	XII	Paramecium tetraurelia	
A0D1K8	enoyl-CoA hydratase	XII	Paramecium tetraurelia	
A7HU11	enoyl-CoA hydratase	XII	Parvibaculum lavamentivorans	
A7HU29	enoyl-CoA hydratase	XII	Parvibaculum lavamentivorans	
E0THJ0	enoyl-CoA hydratase	XII	Parvularcula bermudensis	
C6XXC4	enoyl-CoA hydratase	XII	Pedobacter heparinus	4.2.1.55
A6EDR1	enoyl-CoA hydratase	XII	Pedobacter sp.	
A5D4Y0	enoyl-CoA hydratase	XII	Pelotomaculum thermo.	
B6H8A6	enoyl-CoA hydratase	XII	Penicillium rubens	
A0A069ADE8	enoyl-CoA hydratase	XII	Peptoclostridium difficile	4.2.1.55; 4.2.1.1
A0A0A8U1E3	enoyl-CoA hydratase	XII	Peptoclostridium difficile	4.2.1.55
E4KZS7	enoyl-CoA hydratase	XII	Peptoniphilus harei	4.2.1.55
A0A096BDY2	enoyl-CoA hydratase	XII	Peptoniphilus lacrimalis	
D7N8J4	enoyl-CoA hydratase	XII	Peptoniphilus sp.	4.2.1.55
U7V1P8	enoyl-CoA hydratase	XII	Peptoniphilus sp.	4.2.1.55
I7EUX8	enoyl-CoA hydratase	XII	Phaeobacter gallaeciensis	
I7DWQ7	enoyl-CoA hydratase	XII	Phaeobacter inhibens	
I7E6J1	enoyl-CoA hydratase	XII	Phaeobacter inhibens	
Q0U7R0	enoyl-CoA hydratase	XII	Phaeosphaeria nodorum	
B4R9H1	enoyl-CoA hydratase	XII	Phenylobacterium zucineum	
Q1Z2S0	enoyl-CoA hydratase	XII	Photobacterium profundum	
E7RIF4	enoyl-CoA hydratase	XII	Planococcus donghaensis	4.2.1.17
A6GI53	enoyl-CoA hydratase	XII	Plesiocystis pacifica	
A6GIQ5	enoyl-CoA hydratase	XII	Plesiocystis pacifica	
A4BXH5	enoyl-CoA hydratase	XII	Polaribacter irgensii	
A2TXY1	enoyl-CoA hydratase	XII	Polaribacter sp.	
A1VJU6	enoyl-CoA hydratase	XII	Polaromonas naphthalenivorans	
Q126G4	enoyl-CoA hydratase	XII	Polaromonas sp.	
Q128L7	enoyl-CoA hydratase	XII	Polaromonas sp.	
Q12FT4	enoyl-CoA hydratase	XII	Polaromonas sp.	4.2.1.17
F2J6B8	enoyl-CoA hydratase	XII	Polymorphum gilvum	
B9HX29	enoyl-CoA hydratase	XII	Populus trichocarpa	
C3JBJ6	enoyl-CoA hydratase	XII	Porphyromonas endodontalis	4.2.1.55
Q7MVI2	enoyl-CoA hydratase	XII	Porphyromonas gingivalis	
C2M985	enoyl-CoA hydratase	XII	Porphyromonas uenonis	4.2.1.55
Q3IE67	enoyl-CoA hydratase	XII	Pseudoalteromonas haloplanktis	4.2.1.17
B9Z0D7	enoyl-CoA hydratase	XII	Pseudogulbenkiania ferrooxidans	
V6AE70	enoyl-CoA hydratase	XII	Pseudomonas aeruginosa	
A0A072ZTS3	enoyl-CoA hydratase	XII	Pseudomonas aeruginosa	4.2.1.150
C3K8M5	enoyl-CoA hydratase	XII	Pseudomonas fluorescens	
A0A0B5JWX3	enoyl-CoA hydratase	XII	Pseudomonas plecoglossicida	
B0KML9	enoyl-CoA hydratase	XII	Pseudomonas putida	
F0S7S0	enoyl-CoA hydratase	XII	Pseudopedobacter saltans	4.2.1.55
E6MHW1	enoyl-CoA hydratase	XII	Pseudoramibacter alactolyticus	4.2.1.55
B6QXY5	enoyl-CoA hydratase	XII	Pseudovibrio sp.	4.2.1.55
B6R3L1	enoyl-CoA hydratase	XII	Pseudovibrio sp.	4.2.1.55
E6WR65	enoyl-CoA hydratase	XII	Pseudoxanthomonas suwonensis	
D0U566	enoyl-CoA hydratase	XII	Psychrobacter sp.	
K4III3	enoyl-CoA hydratase	XII	Psychroflexus torquis	
A3MVR3	enoyl-CoA hydratase	XII	Pyrobaculum calidifontis	4.2.1.17
B2UA60	enoyl-CoA hydratase	XII	Ralstonia pickettii	
R7XFD4	enoyl-CoA hydratase	XII	Ralstonia sp.	
R7XKT3	enoyl-CoA hydratase	XII	Ralstonia sp.	4.2.1.17
D3ZIL6	enoyl-CoA hydratase	XII	Rattus norvegicus	

Table S2.5. (continuation)

UniProt	Family [3]	Cluster	Species	EC Number
B3PWW8	enoyl-CoA hydratase	XII	Rhizobium etli	4.2.1.17
C6B5V5	enoyl-CoA hydratase	XII	Rhizobium leguminosarum	
Q1M4T2	enoyl-CoA hydratase	XII	Rhizobium leguminosarum	
Q983W9	enoyl-CoA hydratase	XII	Rhizobium loti	
Q987P2	enoyl-CoA hydratase	XII	Rhizobium loti	
A0A083ZRX3	enoyl-CoA hydratase	XII	Rhizobium radiobacter	4.2.1.55
C3MAP0	enoyl-CoA hydratase	XII	Rhizobium sp.	
A4WSS6	enoyl-CoA hydratase	XII	Rhodobacter sphaeroides	
B9KMQ4	enoyl-CoA hydratase	XII	Rhodobacter sphaeroides	
B9KVT5	enoyl-CoA hydratase	XII	Rhodobacter sphaeroides	
A3PMV8	enoyl-CoA hydratase	XII	Rhodobacter sphaeroides	4.2.1.17
A4WSR8	enoyl-CoA hydratase	XII	Rhodobacter sphaeroides	4.2.1.17
Q3IZD9	enoyl-CoA hydratase	XII	Rhodobacter sphaeroides	4.2.1.17
B6AZF5	enoyl-CoA hydratase	XII	Rhodobacteraceae bacterium	
E4WA13	enoyl-CoA hydratase	XII	Rhodococcus equi	
E9SXB5	enoyl-CoA hydratase	XII	Rhodococcus equi	
E9T3S7	enoyl-CoA hydratase	XII	Rhodococcus equi	
T1VWI1	enoyl-CoA hydratase	XII	Rhodococcus erythropolis	
C0ZTJ5	enoyl-CoA hydratase	XII	Rhodococcus erythropolis	4.2.1.17
Q0S5T5	enoyl-CoA hydratase	XII	Rhodococcus jostii	
Q0SAM2	enoyl-CoA hydratase	XII	Rhodococcus jostii	
Q0S2K8	enoyl-CoA hydratase	XII	Rhodococcus jostii	4.2.1.17
Q0RVK4	enoyl-CoA hydratase	XII	Rhodococcus jostii	4.2.1.55
C1B647	enoyl-CoA hydratase	XII	Rhodococcus opacus	4.2.1.17
C1BDU6	enoyl-CoA hydratase	XII	Rhodococcus opacus	4.2.1.17
A0A069JHM0	enoyl-CoA hydratase	XII	Rhodococcus qingshengii	
M2X0F1	enoyl-CoA hydratase	XII	Rhodococcus qingshengii	
B8X9Y0	enoyl-CoA hydratase	XII	Rhodococcus sp.	
J2JG18	enoyl-CoA hydratase	XII	Rhodococcus sp.	
L2TJK1	enoyl-CoA hydratase	XII	Rhodococcus wratislaviensis	4.2.1.17
Q222H5	enoyl-CoA hydratase	XII	Rhodoferax ferrireducens	
Q222M2	enoyl-CoA hydratase	XII	Rhodoferax ferrireducens	
B3Q7V3	enoyl-CoA hydratase	XII	Rhodopseudomonas palustris	
B3QET5	enoyl-CoA hydratase	XII	Rhodopseudomonas palustris	
E6VNB4	enoyl-CoA hydratase	XII	Rhodopseudomonas palustris	
Q07K02	enoyl-CoA hydratase	XII	Rhodopseudomonas palustris	
Q139W8	enoyl-CoA hydratase	XII	Rhodopseudomonas palustris	
Q21B08	enoyl-CoA hydratase	XII	Rhodopseudomonas palustris	
Q21B14	enoyl-CoA hydratase	XII	Rhodopseudomonas palustris	
Q6N486	enoyl-CoA hydratase	XII	Rhodopseudomonas palustris	4.2.1.-
Q134G1	enoyl-CoA hydratase	XII	Rhodopseudomonas palustris	4.2.1.17
Q2IY89	enoyl-CoA hydratase	XII	Rhodopseudomonas palustris	4.2.1.17
D0MIX9	enoyl-CoA hydratase	XII	Rhodothermus marinus	
G2SE51	enoyl-CoA hydratase	XII	Rhodothermus marinus	4.2.1.17
E4TCS0	enoyl-CoA hydratase	XII	Riemerella anatipestifer	
A4CHJ4	enoyl-CoA hydratase	XII	Robiginitalea biformata	
C0FP89	enoyl-CoA hydratase	XII	Roseburia inulinivorans	
A7NNS3	enoyl-CoA hydratase	XII	Roseiflexus castenholzii	
A5UVM8	enoyl-CoA hydratase	XII	Roseiflexus sp.	4.2.1.17
Q16CS7	enoyl-CoA hydratase	XII	Roseobacter denitrificans	4.2.1.-
A4EUM9	enoyl-CoA hydratase	XII	Roseobacter sp.	
B7RHB4	enoyl-CoA hydratase	XII	Roseobacter sp.	
B7RQY0	enoyl-CoA hydratase	XII	Roseobacter sp.	
A3X6D0	enoyl-CoA hydratase	XII	Roseobacter sp.	4.2.1.17
A3VZZ6	enoyl-CoA hydratase	XII	Roseovarius sp.	
A3W202	enoyl-CoA hydratase	XII	Roseovarius sp.	

Table S2.5. (continuation)

UniProt	Family [3]	Cluster	Species	EC Number
A3W6G8	enoyl-CoA hydratase	XII	Roseovarius sp.	
A6E1A8	enoyl-CoA hydratase	XII	Roseovarius sp.	
A3W3C7	enoyl-CoA hydratase	XII	Roseovarius sp.	4.2.1.17
F0SS49	enoyl-CoA hydratase	XII	Rubinisphaera brasiliensis	
Q1ATI2	enoyl-CoA hydratase	XII	Rubrobacter xylanophilus	4.2.1.17
Q1AV57	enoyl-CoA hydratase	XII	Rubrobacter xylanophilus	4.2.1.17
Q1AV70	enoyl-CoA hydratase	XII	Rubrobacter xylanophilus	4.2.1.17
Q1AZ30	enoyl-CoA hydratase	XII	Rubrobacter xylanophilus	4.2.1.17
Q5LLW6	enoyl-CoA hydratase	XII	Ruegeria pomeroyi	4.2.1.155
F4XDI2	enoyl-CoA hydratase	XII	Ruminococcaceae bacterium	
C7MZQ2	enoyl-CoA hydratase	XII	Saccharomonospora viridis	
C7MW58	enoyl-CoA hydratase	XII	Saccharomonospora viridis	4.2.1.17
T2RVL6	enoyl-CoA hydratase	XII	Saccharopolyspora erythraea	
A3K472	enoyl-CoA hydratase	XII	Sagittula stellata	4.2.1.17
A4X425	enoyl-CoA hydratase	XII	Salinispora tropica	
A7F3S7	enoyl-CoA hydratase	XII	Sclerotinia sclerotiorum	
D8QYQ8	enoyl-CoA hydratase	XII	Selaginella moellendorffii	
B0TMT0	enoyl-CoA hydratase	XII	Shewanella halifaxensis	
A8H4G9	enoyl-CoA hydratase	XII	Shewanella pealeana	
A8FZE7	enoyl-CoA hydratase	XII	Shewanella sediminis	
B1KL79	enoyl-CoA hydratase	XII	Shewanella woodyi	
C4G9V3	enoyl-CoA hydratase	XII	Shuttleworthia satelles	
A6UBK8	enoyl-CoA hydratase	XII	Sinorhizobium medicae	
A6UEB6	enoyl-CoA hydratase	XII	Sinorhizobium medicae	
A6UJN3	enoyl-CoA hydratase	XII	Sinorhizobium medicae	
A6ULC8	enoyl-CoA hydratase	XII	Sinorhizobium medicae	
A0A0E0UJQ0	enoyl-CoA hydratase	XII	Sinorhizobium meliloti	
F7X1U7	enoyl-CoA hydratase	XII	Sinorhizobium meliloti	
F7X2P2	enoyl-CoA hydratase	XII	Sinorhizobium meliloti	
F7XEI0	enoyl-CoA hydratase	XII	Sinorhizobium meliloti	
Q1WL77	enoyl-CoA hydratase	XII	Sinorhizobium meliloti	
Q01T70	enoyl-CoA hydratase	XII	Solibacter usitatus	4.2.1.17
A9GBT5	enoyl-CoA hydratase	XII	Sorangium cellulosum	4.2.1.55
A9GQ91	enoyl-CoA hydratase	XII	Sorangium cellulosum	4.2.1.55
D1C1Z3	enoyl-CoA hydratase	XII	Sphaerobacter thermophilus	
D1C3U0	enoyl-CoA hydratase	XII	Sphaerobacter thermophilus	
F4CAQ9	enoyl-CoA hydratase	XII	Sphingobacterium sp.	4.2.1.17
C2FS94	enoyl-CoA hydratase	XII	Sphingobacterium spiritivorum	4.2.1.55
A0A0A1Y9Y5	enoyl-CoA hydratase	XII	Sphingomonas sp.	
A5V4A9	enoyl-CoA hydratase	XII	Sphingomonas wittichii	
Q1GUS6	enoyl-CoA hydratase	XII	Sphingopyxis alaskensis	
D2QP04	enoyl-CoA hydratase	XII	Spirosoma linguale	
D3Q007	enoyl-CoA hydratase	XII	Stackebrandtia nassauensis	
D3Q8P8	enoyl-CoA hydratase	XII	Stackebrandtia nassauensis	
B8L2Z1	enoyl-CoA hydratase	XII	Stenotrophomonas sp.	4.2.1.55
Q08YS1	enoyl-CoA hydratase	XII	Stigmatella aurantiaca	4.2.1.18; 4.2.1.5
Q08P39	enoyl-CoA hydratase	XII	Stigmatella aurantiaca	4.2.1.55
E8JTC1	enoyl-CoA hydratase	XII	Streptococcus cristatus	4.2.1.55
X8KHN1	enoyl-CoA hydratase	XII	Streptococcus sp.	
D6B1P4	enoyl-CoA hydratase	XII	Streptomyces albus	4.2.1.17
D6B7U8	enoyl-CoA hydratase	XII	Streptomyces albus	4.2.1.17
D7BXE3	enoyl-CoA hydratase	XII	Streptomyces bingchenggensis	
D7CE91	enoyl-CoA hydratase	XII	Streptomyces bingchenggensis	
B5GVC1	enoyl-CoA hydratase	XII	Streptomyces clavuligerus	
E2Q3X2	enoyl-CoA hydratase	XII	Streptomyces clavuligerus	4.2.1.17
Q9EWW0	enoyl-CoA hydratase	XII	Streptomyces coelicolor	

Table S2.5. (continuation)

UniProt	Family [3]	Cluster	Species	EC Number
Q9RJU6	enoyl-CoA hydratase	XII	Streptomyces coelicolor	
D5ZRR9	enoyl-CoA hydratase	XII	Streptomyces ghanaensis	
D6A1R7	enoyl-CoA hydratase	XII	Streptomyces ghanaensis	
D6A5N7	enoyl-CoA hydratase	XII	Streptomyces ghanaensis	
D9XTI1	enoyl-CoA hydratase	XII	Streptomyces griseoflavus	
G0PTP4	enoyl-CoA hydratase	XII	Streptomyces griseus	
Q9KHD9	enoyl-CoA hydratase	XII	Streptomyces griseus	
D9WCZ3	enoyl-CoA hydratase	XII	Streptomyces himastatinicus	
D6EG99	enoyl-CoA hydratase	XII	Streptomyces lividans	
D6EXV0	enoyl-CoA hydratase	XII	Streptomyces lividans	
E8VYZ1	enoyl-CoA hydratase	XII	Streptomyces pratensis	
E8WAL9	enoyl-CoA hydratase	XII	Streptomyces pratensis	
B5HK71	enoyl-CoA hydratase	XII	Streptomyces pristinaespiralis	
W9G1F8	enoyl-CoA hydratase	XII	Streptomyces roseosporus	
C9Z6U1	enoyl-CoA hydratase	XII	Streptomyces scabiei	
C9ZGV4	enoyl-CoA hydratase	XII	Streptomyces scabiei	
B4VAK1	enoyl-CoA hydratase	XII	Streptomyces sp.	
D6M4W6	enoyl-CoA hydratase	XII	Streptomyces sp.	
D6M5P5	enoyl-CoA hydratase	XII	Streptomyces sp.	
D6MSW7	enoyl-CoA hydratase	XII	Streptomyces sp.	
D9UPW7	enoyl-CoA hydratase	XII	Streptomyces sp.	
D9V1Q2	enoyl-CoA hydratase	XII	Streptomyces sp.	
D9V5R0	enoyl-CoA hydratase	XII	Streptomyces sp.	
D9VCR1	enoyl-CoA hydratase	XII	Streptomyces sp.	
D9VVQ3	enoyl-CoA hydratase	XII	Streptomyces sp.	
G2NLX7	enoyl-CoA hydratase	XII	Streptomyces sp.	
B5HMF5	enoyl-CoA hydratase	XII	Streptomyces sviceus	
B5HPC5	enoyl-CoA hydratase	XII	Streptomyces sviceus	
G2NXL2	enoyl-CoA hydratase	XII	Streptomyces violaceusniger	
G2NY41	enoyl-CoA hydratase	XII	Streptomyces violaceusniger	
G2P4V9	enoyl-CoA hydratase	XII	Streptomyces violaceusniger	
G2P562	enoyl-CoA hydratase	XII	Streptomyces violaceusniger	
G2PCU0	enoyl-CoA hydratase	XII	Streptomyces violaceusniger	
D9XDC9	enoyl-CoA hydratase	XII	Streptomyces viridochromogenes	
D9XHG7	enoyl-CoA hydratase	XII	Streptomyces viridochromogenes	
D2AX51	enoyl-CoA hydratase	XII	Streptosporangium roseum	
D1PJ73	enoyl-CoA hydratase	XII	Subdoligranulum variabile	4.2.1.55
M1IEP3	enoyl-CoA hydratase	XII	Sulfolobus acidocaldarius	
Q4J8D5	enoyl-CoA hydratase	XII	Sulfolobus acidocaldarius	4.2.1.55
C3N4B1	enoyl-CoA hydratase	XII	Sulfolobus islandicus	
C3NH34	enoyl-CoA hydratase	XII	Sulfolobus islandicus	
A0A0E3KB58	enoyl-CoA hydratase	XII	Sulfolobus solfataricus	
Q97YL2	enoyl-CoA hydratase	XII	Sulfolobus solfataricus	4.2.1.17
Q976U5	enoyl-CoA hydratase	XII	Sulfolobus tokodaii	4.2.1.17
Q67P31	enoyl-CoA hydratase	XII	Symbiobacterium thermophilum	
A0LPA2	enoyl-CoA hydratase	XII	Syntrophobacter fumaroxidans	4.2.1.17
Q0AVC9	enoyl-CoA hydratase	XII	Syntrophomonas wolfei	4.2.1.17
Q0AVM1	enoyl-CoA hydratase	XII	Syntrophomonas wolfei	4.2.1.17
Q0AWW6	enoyl-CoA hydratase	XII	Syntrophomonas wolfei	4.2.1.17
Q0AYU3	enoyl-CoA hydratase	XII	Syntrophomonas wolfei	4.2.1.17
D7CKN4	enoyl-CoA hydratase	XII	Syntrophothermus lipocalidus	4.2.1.17
D7CKX9	enoyl-CoA hydratase	XII	Syntrophothermus lipocalidus	4.2.1.17
D7CIV8	enoyl-CoA hydratase	XII	Syntrophothermus lipocalidus	4.2.1.55
D7CJI4	enoyl-CoA hydratase	XII	Syntrophothermus lipocalidus	4.2.1.55
D7CKN5	enoyl-CoA hydratase	XII	Syntrophothermus lipocalidus	4.2.1.55
D7CKQ1	enoyl-CoA hydratase	XII	Syntrophothermus lipocalidus	4.2.1.55

Table S2.5. (continuation)

UniProt	Family [3]	Cluster	Species	EC Number
D7CMS2	enoyl-CoA hydratase	XII	Syntrophothermus lipocalidus	4.2.1.55
D7CPW2	enoyl-CoA hydratase	XII	Syntrophothermus lipocalidus	4.2.1.55
Q2LUN3	enoyl-CoA hydratase	XII	Syntrophus aciditrophicus	
I7MCH4	enoyl-CoA hydratase	XII	Tetrahymena thermophila	
C4K9L8	enoyl-CoA hydratase	XII	Thauera sp.	
A0A0C3N2C6	enoyl-CoA hydratase	XII	Thauera sp.	4.2.1.116
E6SH14	enoyl-CoA hydratase	XII	Thermaerobacter marianensis	
I8QZG8	enoyl-CoA hydratase	XII	Thermoanaerobacter siderophilus	
D9TQ01	enoyl-CoA hydratase	XII	Thermoanaerobacterium thermosac.	
D1CDC3	enoyl-CoA hydratase	XII	Thermobaculum terrenum	
Q47SD9	enoyl-CoA hydratase	XII	Thermobifida fusca	4.2.1.17
D6Y6R2	enoyl-CoA hydratase	XII	Thermobispora bispora	
B9KXU8	enoyl-CoA hydratase	XII	Thermomicrobium roseum	
B9L5H2	enoyl-CoA hydratase	XII	Thermomicrobium roseum	4.2.1.17
B9L259	enoyl-CoA hydratase	XII	Thermomicrobium roseum	4.2.1.55
Q9HLV4	enoyl-CoA hydratase	XII	Thermoplasma acidophilum	
Q9HM10	enoyl-CoA hydratase	XII	Thermoplasma acidophilum	
Q97CA4	enoyl-CoA hydratase	XII	Thermoplasma volcanium	
Q97CT4	enoyl-CoA hydratase	XII	Thermoplasma volcanium	
A1HPI9	enoyl-CoA hydratase	XII	Thermosinus carboxydivorans	
A1HSH2	enoyl-CoA hydratase	XII	Thermosinus carboxydivorans	
B7AA35	enoyl-CoA hydratase	XII	Thermus aquaticus	4.2.1.17
Q5SIE0	enoyl-CoA hydratase	XII	Thermus thermophilus	
D6CMD6	enoyl-CoA hydratase	XII	Thiomonas arsenitoxydans	4.2.1.55
E7NY34	enoyl-CoA hydratase	XII	Treponema phagedenis	4.2.1.55
C8PR75	enoyl-CoA hydratase	XII	Treponema vincentii	4.2.1.55
D6WXJ1	enoyl-CoA hydratase	XII	Tribolium castaneum	
Q4CWM5	enoyl-CoA hydratase	XII	Trypanosoma cruzi	
D5UNT0	enoyl-CoA hydratase	XII	Tsukamurella paurometabola	
D5UU31	enoyl-CoA hydratase	XII	Tsukamurella paurometabola	
D6PBI7	enoyl-CoA hydratase	XII	uncultured archaeon	
Q64BG5	enoyl-CoA hydratase	XII	uncultured archaeon	
D1JFP4	enoyl-CoA hydratase	XII	uncultured archaeon	4.2.1.55
B1N6E0	enoyl-CoA hydratase	XII	uncultured bacterium	
B3F475	enoyl-CoA hydratase	XII	uncultured bacterium	
H5SLJ5	enoyl-CoA hydratase	XII	uncultured crenarchaeote	
E1YB19	enoyl-CoA hydratase	XII	uncultured Desulfobacterium	
E1YKI5	enoyl-CoA hydratase	XII	uncultured Desulfobacterium	
E1YKJ2	enoyl-CoA hydratase	XII	uncultured Desulfobacterium	
E1YKK5	enoyl-CoA hydratase	XII	uncultured Desulfobacterium	
B3T4G1	enoyl-CoA hydratase	XII	uncultured marine	
B3V6K5	enoyl-CoA hydratase	XII	uncultured marine	1.1.1.35
E0XZ52	enoyl-CoA hydratase	XII	uncultured Rhizobiales	
A6ELJ7	enoyl-CoA hydratase	XII	unidentified eubacterium	
A0A0D1E0P3	enoyl-CoA hydratase	XII	Ustilago maydis	
Q4PEN0	enoyl-CoA hydratase	XII	Ustilago maydis	
C5CYV7	enoyl-CoA hydratase	XII	Variovorax paradoxus	
E6V386	enoyl-CoA hydratase	XII	Variovorax paradoxus	
A1WQR5	enoyl-CoA hydratase	XII	Verminephrobacter eiseniae	
A1WRQ9	enoyl-CoA hydratase	XII	Verminephrobacter eiseniae	
A1WF10	enoyl-CoA hydratase	XII	Verminephrobacter eiseniae	4.2.1.17
A1WIF9	enoyl-CoA hydratase	XII	Verminephrobacter eiseniae	4.2.1.17
A1WIW1	enoyl-CoA hydratase	XII	Verminephrobacter eiseniae	4.2.1.17
A1WNT2	enoyl-CoA hydratase	XII	Verminephrobacter eiseniae	4.2.1.17
A1WPM2	enoyl-CoA hydratase	XII	Verminephrobacter eiseniae	4.2.1.17
F0V0X3	enoyl-CoA hydratase	XII	Vibrio nigripulchritudo	

Table S2.5. (continuation)

UniProt	Family [3]	Cluster	Species	EC Number
E8MC54	enoyl-CoA hydratase	XII	Vibrio sinaloensis	
A3UPT1	enoyl-CoA hydratase	XII	Vibrio splendidus	
A5AYE3	enoyl-CoA hydratase	XII	Vitis vinifera	
D6YRX7	enoyl-CoA hydratase	XII	Waddlia chondrophila	4.2.1.55
F0P1K9	enoyl-CoA hydratase	XII	Weeksella virosa	4.2.1.17; 4.2.1.5
A7IKN6	enoyl-CoA hydratase	XII	Xanthobacter autotrophicus	
D2U8T9	enoyl-CoA hydratase	XII	Xanthomonas albilineans	4.2.1.55
B0RV18	enoyl-CoA hydratase	XII	Xanthomonas campestris	4.2.1.17
Q5XG25	enoyl-CoA hydratase	XII	Xenopus laevis	
Q6C0S5	enoyl-CoA hydratase	XII	Yarrowia lipolytica	
D5BBP9	enoyl-CoA hydratase	XII	Zunongwangia profunda	
A1W287	enoyl-CoA hydratase	X	Acidovorax sp.	4.2.1.17
A0A0D5YIY0	enoyl-CoA hydratase	X	Acinetobacter baumannii	4.2.1.17
D8JM23	enoyl-CoA hydratase	X	Acinetobacter oleivorans	
K9CTB7	enoyl-CoA hydratase	X	Acinetobacter sp.	
Q6ZZH3	enoyl-CoA hydratase	X	Actinoplanes teichomyceticus	
H0H5T1	enoyl-CoA hydratase	X	Agrobacterium tumefaciens	4.2.1.17
A6QSI5	enoyl-CoA hydratase	X	Ajellomyces capsulatus	
F0UPB6	enoyl-CoA hydratase	X	Ajellomyces capsulatus	
C5GID8	enoyl-CoA hydratase	X	Ajellomyces dermatitidis	
C5GS56	enoyl-CoA hydratase	X	Ajellomyces dermatitidis	
B4X4L4	enoyl-CoA hydratase	X	Alcanivorax sp.	
E8TVV4	enoyl-CoA hydratase	X	Alicycliphilus denitrificans	
G0G164	enoyl-CoA hydratase	X	Amycolatopsis mediterranei	
Q2IFZ7	enoyl-CoA hydratase	X	Anaeromyxobacter dehalogenans	
Q7PY24	enoyl-CoA hydratase	X	Anopheles gambiae	
A1R3Q4	enoyl-CoA hydratase	X	Arthrobacter aurescens	
A0JTV3	enoyl-CoA hydratase	X	Arthrobacter sp.	4.2.1.17
C5FVG6	enoyl-CoA hydratase	X	Arthroderma otae	
A1C6B3	enoyl-CoA hydratase	X	Aspergillus clavatus	
A1CC84	enoyl-CoA hydratase	X	Aspergillus clavatus	
A0A0D9MVR4	enoyl-CoA hydratase	X	Aspergillus flavus	
A2QD50	enoyl-CoA hydratase	X	Aspergillus niger	4.2.1.17
A2QGJ8	enoyl-CoA hydratase	X	Aspergillus niger	5.-.-.-
I8TIC1	enoyl-CoA hydratase	X	Aspergillus oryzae	
I8TJI8	enoyl-CoA hydratase	X	Aspergillus oryzae	
Q2US93	enoyl-CoA hydratase	X	Aspergillus oryzae	
Q0CKD8	enoyl-CoA hydratase	X	Aspergillus terreus	
Q0CXS5	enoyl-CoA hydratase	X	Aspergillus terreus	
Q5XW69	enoyl-CoA hydratase	X	Azoarcus sp.	
Q7VUB1	enoyl-CoA hydratase	X	Bordetella pertussis	4.2.1.-
M7TWX8	enoyl-CoA hydratase	X	Botryotinia fuckeliana	
M7UUA6	enoyl-CoA hydratase	X	Botryotinia fuckeliana	
A4YR68	enoyl-CoA hydratase	X	Bradyrhizobium sp.	4.2.1.17
A5EEQ6	enoyl-CoA hydratase	X	Bradyrhizobium sp.	4.2.1.17
A5EFV4	enoyl-CoA hydratase	X	Bradyrhizobium sp.	4.2.1.17
Q57EV4	enoyl-CoA hydratase	X	Brucella abortus	
Q0B1C1	enoyl-CoA hydratase	X	Burkholderia ambifaria	
B1K3Z6	enoyl-CoA hydratase	X	Burkholderia cenocepacia	
A0A088TBM9	enoyl-CoA hydratase	X	Burkholderia cepacia	
A0A0A5LNY5	enoyl-CoA hydratase	X	Burkholderia cepacia	4.2.1.17
Q39N06	enoyl-CoA hydratase	X	Burkholderia lata	4.2.1.17
Q39P26	enoyl-CoA hydratase	X	Burkholderia lata	4.2.1.17
B9BTT8	enoyl-CoA hydratase	X	Burkholderia multivorans	
B2JSH2	enoyl-CoA hydratase	X	Burkholderia phymatum	
B2T0A3	enoyl-CoA hydratase	X	Burkholderia phytofirmans	

Table S2.5. (continuation)

UniProt	Family [3]	Cluster	Species	EC Number
A8E9L0	enoyl-CoA hydratase	X	Burkholderia pseudomallei	
A0A069AYF7	enoyl-CoA hydratase	X	Burkholderia pseudomallei	4.2.1.17
A0A0D5LAE0	enoyl-CoA hydratase	X	Burkholderia sp.	
B5WI60	enoyl-CoA hydratase	X	Burkholderia sp.	
I2IC82	enoyl-CoA hydratase	X	Burkholderia sp.	
A4JQ06	enoyl-CoA hydratase	X	Burkholderia vietnamiensis	4.2.1.17
Q13I51	enoyl-CoA hydratase	X	Burkholderia xenovorans	4.2.1.17
B0SX37	enoyl-CoA hydratase	X	Caulobacter sp.	
B0T9N1	enoyl-CoA hydratase	X	Caulobacter sp.	
Q2GRD2	enoyl-CoA hydratase	X	Chaetomium globosum	
J3K865	enoyl-CoA hydratase	X	Coccidioides immitis	
B7X550	enoyl-CoA hydratase	X	Comamonas testosteroni	
D3FBM3	enoyl-CoA hydratase	X	Conexibacter woesei	
A4AD92	enoyl-CoA hydratase	X	Congregibacter litoralis	4.2.1.17
A8N3H3	enoyl-CoA hydratase	X	Coprinopsis cinerea	
Q1LCW3	enoyl-CoA hydratase	X	Cupriavidus metallidurans	4.2.1.17
Q0KAI1	enoyl-CoA hydratase	X	Cupriavidus necator	4.2.1.17
Q46NA5	enoyl-CoA hydratase	X	Cupriavidus pinatubonensis	
Q471A0	enoyl-CoA hydratase	X	Cupriavidus pinatubonensis	4.2.1.17
B4GNY1	enoyl-CoA hydratase	X	Drosophila persimilis	
B4HGX6	enoyl-CoA hydratase	X	Drosophila sechellia	
B4IGU1	enoyl-CoA hydratase	X	Drosophila sechellia	
B4PRC1	enoyl-CoA hydratase	X	Drosophila yakuba	
Q5AZP5	enoyl-CoA hydratase	X	Emericella nidulans	
Q5B0S8	enoyl-CoA hydratase	X	Emericella nidulans	
Q2NDY9	enoyl-CoA hydratase	X	Erythrobacter litoralis	
A3WGH4	enoyl-CoA hydratase	X	Erythrobacter sp.	
A5PC80	enoyl-CoA hydratase	X	Erythrobacter sp.	
E9YX03	enoyl-CoA hydratase	X	Escherichia coli	
S0AQA3	enoyl-CoA hydratase	X	Ferroplasma acidarmanus	
Q0RJ70	enoyl-CoA hydratase	X	Frankia alni	4.2.1.17
Q0RN05	enoyl-CoA hydratase	X	Frankia alni	4.2.1.17
A8L5R0	enoyl-CoA hydratase	X	Frankia sp.	
A8L836	enoyl-CoA hydratase	X	Frankia sp.	
A8LBA2	enoyl-CoA hydratase	X	Frankia sp.	
A8LD66	enoyl-CoA hydratase	X	Frankia sp.	
A8LD72	enoyl-CoA hydratase	X	Frankia sp.	
Q2JA70	enoyl-CoA hydratase	X	Frankia sp.	4.2.1.17
A0A016Q4M0	enoyl-CoA hydratase	X	Gibberella zeae	
A0A016Q7G1	enoyl-CoA hydratase	X	Gibberella zeae	
D2RPG3	enoyl-CoA hydratase	X	Haloterrigena turkmenica	
A0A059FZB5	enoyl-CoA hydratase	X	Hyphomonas hirschiana	
A3TIG7	enoyl-CoA hydratase	X	Janibacter sp.	
Q28KM1	enoyl-CoA hydratase	X	Jannaschia sp.	
Q28KU5	enoyl-CoA hydratase	X	Jannaschia sp.	4.2.1.17
A0P146	enoyl-CoA hydratase	X	Labrenzia aggregata	
A0NZH1	enoyl-CoA hydratase	X	Labrenzia aggregata	4.2.1.-
A4ALU5	enoyl-CoA hydratase	X	marine actinobacterium	
A4AHS5	enoyl-CoA hydratase	X	marine actinobacterium	4.2.1.17
A0Y8B2	enoyl-CoA hydratase	X	marine gamma	
A0YHH7	enoyl-CoA hydratase	X	marine gamma	4.2.1.17
A0Z6G3	enoyl-CoA hydratase	X	marine gamma	4.2.1.17
A3Y683	enoyl-CoA hydratase	X	Marinomonas sp.	4.2.1.-
A3VK64	enoyl-CoA hydratase	X	Maritimibacter alkaliphilus	
E8T8Z6	enoyl-CoA hydratase	X	Mesorhizobium ciceri	
E9DRA5	enoyl-CoA hydratase	X	Metarhizium acridum	

Table S2.5. (continuation)

UniProt	Family [3]	Cluster	Species	EC Number
A0A014PN89	enoyl-CoA hydratase	X	Metarhizium robertsii	
A9VX95	enoyl-CoA hydratase	X	Methylobacterium extorquens	
B7KXW2	enoyl-CoA hydratase	X	Methylobacterium extorquens	
C7CGB0	enoyl-CoA hydratase	X	Methylobacterium extorquens	4.2.1.17
V7JT16	enoyl-CoA hydratase	X	Mycobacterium avium	
V7JUG8	enoyl-CoA hydratase	X	Mycobacterium avium	
V7K6H2	enoyl-CoA hydratase	X	Mycobacterium avium	
V7JBQ4	enoyl-CoA hydratase	X	Mycobacterium avium	4.2.1.17
V7JJQ3	enoyl-CoA hydratase	X	Mycobacterium avium	4.2.1.17
V7KAC9	enoyl-CoA hydratase	X	Mycobacterium avium	4.2.1.17
V7KZ97	enoyl-CoA hydratase	X	Mycobacterium avium	4.2.1.17
E6TL70	enoyl-CoA hydratase	X	Mycobacterium gilvum	
A4T7S8	enoyl-CoA hydratase	X	Mycobacterium gilvum	4.2.1.17
A4T948	enoyl-CoA hydratase	X	Mycobacterium gilvum	4.2.1.17
E6TFL4	enoyl-CoA hydratase	X	Mycobacterium gilvum	4.2.1.17
E6TJG3	enoyl-CoA hydratase	X	Mycobacterium gilvum	4.2.1.17
J9WGH7	enoyl-CoA hydratase	X	Mycobacterium indicus	
H8IM21	enoyl-CoA hydratase	X	Mycobacterium intracellulare	
U5WSM9	enoyl-CoA hydratase	X	Mycobacterium kansasii	4.2.1.17
A0A0D6J0J6	enoyl-CoA hydratase	X	Mycobacterium smegmatis	4.2.1.-
A1UDW1	enoyl-CoA hydratase	X	Mycobacterium sp.	
A1UGP0	enoyl-CoA hydratase	X	Mycobacterium sp.	
A1UM49	enoyl-CoA hydratase	X	Mycobacterium sp.	4.2.1.17
A0A040DM48	enoyl-CoA hydratase	X	Mycobacterium tuberculosis	
A0A042R8X5	enoyl-CoA hydratase	X	Mycobacterium tuberculosis	
A0A047RNB3	enoyl-CoA hydratase	X	Mycobacterium tuberculosis	
A0A051TU83	enoyl-CoA hydratase	X	Mycobacterium tuberculosis	
A0A045IFS9	enoyl-CoA hydratase	X	Mycobacterium tuberculosis	4.2.1.17
A0PMM1	enoyl-CoA hydratase	X	Mycobacterium ulcerans	
A1T7I4	enoyl-CoA hydratase	X	Mycobacterium vanbaalenii	
A1T6K9	enoyl-CoA hydratase	X	Mycobacterium vanbaalenii	4.2.1.17
A1TDW3	enoyl-CoA hydratase	X	Mycobacterium vanbaalenii	4.2.1.17
A1TFN7	enoyl-CoA hydratase	X	Mycobacterium vanbaalenii	4.2.1.17
C7YV42	enoyl-CoA hydratase	X	Nectria haematococca	
A1D7U8	enoyl-CoA hydratase	X	Neosartorya fischeri	
A1DGY8	enoyl-CoA hydratase	X	Neosartorya fischeri	
A1DKF6	enoyl-CoA hydratase	X	Neosartorya fischeri	
Q4WF54	enoyl-CoA hydratase	X	Neosartorya fumigata	4.2.1.-
Q4X1A5	enoyl-CoA hydratase	X	Neosartorya fumigata	4.2.1.-
Q5YNE5	enoyl-CoA hydratase	X	Nocardia farcinica	
Q5YVK2	enoyl-CoA hydratase	X	Nocardia farcinica	
Q5Z2G8	enoyl-CoA hydratase	X	Nocardia farcinica	
A1SEV1	enoyl-CoA hydratase	X	Nocardioides sp.	4.2.1.17
A1SK94	enoyl-CoA hydratase	X	Nocardioides sp.	4.2.1.17
Q2GB15	enoyl-CoA hydratase	X	Novosphingobium aromat.	
Q2G850	enoyl-CoA hydratase	X	Novosphingobium aromat.	4.2.1.17
Q2G8B7	enoyl-CoA hydratase	X	Novosphingobium aromat.	4.2.1.17
C1H930	enoyl-CoA hydratase	X	Paracoccidioides lutzii	
A7HWG5	enoyl-CoA hydratase	X	Parvibaculum lavamentivorans	
B6HRL6	enoyl-CoA hydratase	X	Penicillium rubens	
Q0V6R7	enoyl-CoA hydratase	X	Phaeosphaeria nodorum	
A0A086C1Q3	enoyl-CoA hydratase	X	Pseudomonas aeruginosa	4.2.1.17
I7C7A2	enoyl-CoA hydratase	X	Pseudomonas putida	
Q8ZV32	enoyl-CoA hydratase	X	Pyrobaculum aerophilum	
D8N807	enoyl-CoA hydratase	X	Ralstonia solanacearum	4.2.1.17
D8NHB5	enoyl-CoA hydratase	X	Ralstonia solanacearum	4.2.1.17

Table S2.5. (continuation)

UniProt	Family [3]	Cluster	Species	EC Number
Q2K1H3	enoyl-CoA hydratase	X	Rhizobium etli	
B3PS61	enoyl-CoA hydratase	X	Rhizobium etli	4.2.1.17
C6B3I4	enoyl-CoA hydratase	X	Rhizobium leguminosarum	
Q1M7C8	enoyl-CoA hydratase	X	Rhizobium leguminosarum	
Q1M712	enoyl-CoA hydratase	X	Rhizobium leguminosarum	4.2.1.-
A0A083ZQ07	enoyl-CoA hydratase	X	Rhizobium radiobacter	4.2.1.17
Q0FAC5	enoyl-CoA hydratase	X	Rhodobacterales bacterium	
Q0S5K4	enoyl-CoA hydratase	X	Rhodococcus jostii	
Q0S9N9	enoyl-CoA hydratase	X	Rhodococcus jostii	
Q0SEE4	enoyl-CoA hydratase	X	Rhodococcus jostii	
Q0S7L2	enoyl-CoA hydratase	X	Rhodococcus jostii	4.2.1.17
C1B8S1	enoyl-CoA hydratase	X	Rhodococcus opacus	
C1B9R2	enoyl-CoA hydratase	X	Rhodococcus opacus	
N1MG66	enoyl-CoA hydratase	X	Rhodococcus sp.	4.2.1.17
U0F8C4	enoyl-CoA hydratase	X	Rhodococcus sp.	4.2.1.17
Q138P8	enoyl-CoA hydratase	X	Rhodopseudomonas palustris	
Q6N8Z0	enoyl-CoA hydratase	X	Rhodopseudomonas palustris	
Q6N498	enoyl-CoA hydratase	X	Rhodopseudomonas palustris	4.2.1.-
Q07J31	enoyl-CoA hydratase	X	Rhodopseudomonas palustris	4.2.1.17
Q139Y9	enoyl-CoA hydratase	X	Rhodopseudomonas palustris	4.2.1.17
Q20XY4	enoyl-CoA hydratase	X	Rhodopseudomonas palustris	4.2.1.17
Q218F1	enoyl-CoA hydratase	X	Rhodopseudomonas palustris	4.2.1.17
Q2IU13	enoyl-CoA hydratase	X	Rhodopseudomonas palustris	4.2.1.17
Q6N7V9	enoyl-CoA hydratase	X	Rhodopseudomonas palustris	4.2.1.17
Q16CI0	enoyl-CoA hydratase	X	Roseobacter denitrificans	4.2.1.-
A4EN19	enoyl-CoA hydratase	X	Roseobacter sp.	
A6FRV4	enoyl-CoA hydratase	X	Roseobacter sp.	
A6FTU6	enoyl-CoA hydratase	X	Roseobacter sp.	
A6DWU4	enoyl-CoA hydratase	X	Roseovarius sp.	4.2.1.-
Q1GCU1	enoyl-CoA hydratase	X	Ruegeria sp.	
C7MYF0	enoyl-CoA hydratase	X	Saccharomonospora viridis	
T2RRH0	enoyl-CoA hydratase	X	Saccharopolyspora erythraea	
T2RUG2	enoyl-CoA hydratase	X	Saccharopolyspora erythraea	
T2RYS9	enoyl-CoA hydratase	X	Saccharopolyspora erythraea	4.2.1.17
A3JZE0	enoyl-CoA hydratase	X	Sagittula stellata	4.2.1.-
A8M6F2	enoyl-CoA hydratase	X	Salinispora arenicola	
A4X884	enoyl-CoA hydratase	X	Salinispora tropica	4.2.1.17
A7EG08	enoyl-CoA hydratase	X	Sclerotinia sclerotiorum	
S0ADZ3	enoyl-CoA hydratase	X	Serratia plymuthica	4.2.1.-
A8GCU1	enoyl-CoA hydratase	X	Serratia proteamaculans	
A1SA48	enoyl-CoA hydratase	X	Shewanella amazonensis	4.2.1.17
A0KT40	enoyl-CoA hydratase	X	Shewanella sp.	4.2.1.17
D4ZFI0	enoyl-CoA hydratase	X	Shewanella violacea	
A6U6R5	enoyl-CoA hydratase	X	Sinorhizobium medicae	
A6UBI0	enoyl-CoA hydratase	X	Sinorhizobium medicae	
C5WMF3	enoyl-CoA hydratase	X	Sorghum bicolor	
Q1N744	enoyl-CoA hydratase	X	Sphingomonas sp.	
A5V8M2	enoyl-CoA hydratase	X	Sphingomonas wittichii	
A5V7R2	enoyl-CoA hydratase	X	Sphingomonas wittichii	4.2.1.17
A5V7X5	enoyl-CoA hydratase	X	Sphingomonas wittichii	4.2.1.17
Q1GS52	enoyl-CoA hydratase	X	Sphingopyxis alaskensis	4.2.1.17
Q93GX5	enoyl-CoA hydratase	X	Streptomyces avermitilis	
D9WGX6	enoyl-CoA hydratase	X	Streptomyces himastatinicus	
Q9KHL0	enoyl-CoA hydratase	X	Streptomyces maritimus	
C9Z4T7	enoyl-CoA hydratase	X	Streptomyces scabiei	
D9US25	enoyl-CoA hydratase	X	Streptomyces sp.	

Table S2.5. (continuation)

UniProt	Family [3]	Cluster	Species	EC Number
A0A085BZ11	enoyl-CoA hydratase	X	Sulfitobacter sp.	4.2.1.17
M1J0Y1	enoyl-CoA hydratase	X	Sulfolobus acidocaldarius	
Q976Z3	enoyl-CoA hydratase	X	Sulfolobus tokodaii	4.2.1.17
Q9KJE7	enoyl-CoA hydratase	X	Thauera aromatica	
Q47QF1	enoyl-CoA hydratase	X	Thermobifida fusca	4.2.1.17
D5X623	enoyl-CoA hydratase	X	Thiomonas intermedia	
Q4PK70	enoyl-CoA hydratase	X	uncultured bacterium	
E1YFR5	enoyl-CoA hydratase	X	uncultured Desulfobacterium	
A7U0V0	enoyl-CoA hydratase	X	uncultured haloarchaeon	
C5CMH2	enoyl-CoA hydratase	X	Variovorax paradoxus	
A1WNL6	enoyl-CoA hydratase	X	Verminephrobacter eiseniae	4.2.1.17
A1WPK5	enoyl-CoA hydratase	X	Verminephrobacter eiseniae	4.2.1.17
E1QTQ7	enoyl-CoA hydratase	X	Vulcanisaeta distributa	
F0QYV3	enoyl-CoA hydratase	X	Vulcanisaeta moutnovskia	
A0A0C7CXM0	enoyl-CoA hydratase	XI		
A0A0F1CA53	enoyl-CoA hydratase	XI		
D7HZ69	enoyl-CoA hydratase	XI		
E7P4E0	enoyl-CoA hydratase	XI		
C7RVJ1	enoyl-CoA hydratase	XI	Accumulibacter phosphatis	
F7S335	enoyl-CoA hydratase	XI	Acidiphilium sp.	4.2.1.17
F0Q8T7	enoyl-CoA hydratase	XI	Acidovorax avenae	
A0A009I2K8	enoyl-CoA hydratase	XI	Acinetobacter baumannii	
A0A009MXP8	enoyl-CoA hydratase	XI	Acinetobacter baumannii	
A0A009MY24	enoyl-CoA hydratase	XI	Acinetobacter baumannii	
Q6FBV4	enoyl-CoA hydratase	XI	Acinetobacter baylyi	4.2.1.17
Q937T3	enoyl-CoA hydratase	XI	Acinetobacter baylyi	4.2.1.17
F0KJC4	enoyl-CoA hydratase	XI	Acinetobacter calcoaceticus	
F0KK99	enoyl-CoA hydratase	XI	Acinetobacter calcoaceticus	
D0SU20	enoyl-CoA hydratase	XI	Acinetobacter lwoffii	
D0SWC8	enoyl-CoA hydratase	XI	Acinetobacter lwoffii	
D8JM16	enoyl-CoA hydratase	XI	Acinetobacter oleivorans	4.2.1.17
N9Q1Y6	enoyl-CoA hydratase	XI	Acinetobacter sp.	
J9JM53	enoyl-CoA hydratase	XI	Acyrthosiphon pisum	
Q17E10	enoyl-CoA hydratase	XI	Aedes aegypti	
D6V8R5	enoyl-CoA hydratase	XI	Afipia sp.	
Q7D1P4	enoyl-CoA hydratase	XI	Agrobacterium fabrum	
B9JIE7	enoyl-CoA hydratase	XI	Agrobacterium radiobacter	
E0MMC4	enoyl-CoA hydratase	XI	Ahrensia sp.	
D2I0P4	enoyl-CoA hydratase	XI	Ailuropoda melanoleuca	
A6REJ3	enoyl-CoA hydratase	XI	Ajellomyces capsulatus	
C6HHD7	enoyl-CoA hydratase	XI	Ajellomyces capsulatus	
F0WL51	enoyl-CoA hydratase	XI	Albugo laibachii	
E7DDH9	enoyl-CoA hydratase	XI	Alcaligenes faecalis	
A0A0A5L5T4	enoyl-CoA hydratase	XI	Alcaligenes xylosoxydans	4.2.1.17
E8TR17	enoyl-CoA hydratase	XI	Alicycliphilus denitrificans	
C8WUA7	enoyl-CoA hydratase	XI	Alicyclobacillus acidocaldarius	
A8U121	enoyl-CoA hydratase	XI	alpha proteobacterium	
W5JKK3	enoyl-CoA hydratase	XI	Anopheles darlingi	
Q5P6B0	enoyl-CoA hydratase	XI	Aromatoleum aromaticum	
Q5NZY4	enoyl-CoA hydratase	XI	Aromatoleum aromaticum	4.2.1.17
Q5P654	enoyl-CoA hydratase	XI	Aromatoleum aromaticum	4.2.1.17
Q5P772	enoyl-CoA hydratase	XI	Aromatoleum aromaticum	4.2.1.17
A1R248	enoyl-CoA hydratase	XI	Arthrobacter aurescens	
B8HBE6	enoyl-CoA hydratase	XI	Arthrobacter chlorophenolicus	
A0JS04	enoyl-CoA hydratase	XI	Arthrobacter sp.	4.2.1.17
E4UYE5	enoyl-CoA hydratase	XI	Arthroderma gypseum	

Table S2.5. (continuation)

UniProt	Family [3]	Cluster	Species	EC Number
F1L9N2	enoyl-CoA hydratase	XI	Ascaris suum	
F1LCC6	enoyl-CoA hydratase	XI	Ascaris suum	
A1C8U5	enoyl-CoA hydratase	XI	Aspergillus clavatus	
A0A0D9N109	enoyl-CoA hydratase	XI	Aspergillus flavus	
A2QCA2	enoyl-CoA hydratase	XI	Aspergillus niger	4.2.1.17
Q1YED6	enoyl-CoA hydratase	XI	Aurantimonas manganoxydans	
F0YAZ6	enoyl-CoA hydratase	XI	Aureococcus anophagefferens	
A1K3K2	enoyl-CoA hydratase	XI	Azoarcus sp.	4.2.1.17
A1K6T9	enoyl-CoA hydratase	XI	Azoarcus sp.	4.2.1.17
A8IQN3	enoyl-CoA hydratase	XI	Azorhizobium caulinodans	
D3NY30	enoyl-CoA hydratase	XI	Azospirillum sp.	4.2.1.17
C1DNT6	enoyl-CoA hydratase	XI	Azotobacter vinelandii	
Q9Z9V2	enoyl-CoA hydratase	XI	Bacillus halodurans	
A6CP11	enoyl-CoA hydratase	XI	Bacillus sp.	
E5WMZ5	enoyl-CoA hydratase	XI	Bacillus sp.	
B2IFQ3	enoyl-CoA hydratase	XI	Beijerinckia indica	
E3UKK2	enoyl-CoA hydratase	XI	Biston betularia	
Q2F6B0	enoyl-CoA hydratase	XI	Bombyx mori	
Q2KU28	enoyl-CoA hydratase	XI	Bordetella avium	
K0MNY1	enoyl-CoA hydratase	XI	Bordetella parapertussis	
Q58DM8	enoyl-CoA hydratase	XI	Bos taurus	4.2.1.17
M7UCD1	enoyl-CoA hydratase	XI	Botryotinia fuckeliana	
A5EG33	enoyl-CoA hydratase	XI	Bradyrhizobium sp.	4.2.1.17
C3YPS2	enoyl-CoA hydratase	XI	Branchiostoma floridae	
C0Z573	enoyl-CoA hydratase	XI	Brevibacillus brevis	4.2.1.17
D4YLZ6	enoyl-CoA hydratase	XI	Brevibacterium mcbrellneri	4.2.1.55
B4W765	enoyl-CoA hydratase	XI	Brevundimonas sp.	
D9QGA0	enoyl-CoA hydratase	XI	Brevundimonas subvibrioides	
E0DJT0	enoyl-CoA hydratase	XI	Brucella inopinata	4.2.1.17
F2HXA4	enoyl-CoA hydratase	XI	Brucella melitensis	
D1CZX6	enoyl-CoA hydratase	XI	Brucella sp.	
B1KCQ8	enoyl-CoA hydratase	XI	Burkholderia cenocepacia	
E5AQ28	enoyl-CoA hydratase	XI	Burkholderia rhizoxinica	4.2.1.17
D5WKB4	enoyl-CoA hydratase	XI	Burkholderia sp.	
I2IJ54	enoyl-CoA hydratase	XI	Burkholderia sp.	
I2IML8	enoyl-CoA hydratase	XI	Burkholderia sp.	
Q2SUJ3	enoyl-CoA hydratase	XI	Burkholderia thailandensis	
A8X7A3	enoyl-CoA hydratase	XI	Caenorhabditis briggsae	
Q9NEZ8	enoyl-CoA hydratase	XI	Caenorhabditis elegans	
P34559	enoyl-CoA hydratase	XI	Caenorhabditis elegans	4.2.1.17
E3LS83	enoyl-CoA hydratase	XI	Caenorhabditis remanei	
E3MFB1	enoyl-CoA hydratase	XI	Caenorhabditis remanei	
C1C0A7	enoyl-CoA hydratase	XI	Caligus clemensi	
C1BNS6	enoyl-CoA hydratase	XI	Caligus rogercresseyi	
C1BNZ6	enoyl-CoA hydratase	XI	Caligus rogercresseyi	
F7H7J1	enoyl-CoA hydratase	XI	Callithrix jacchus	
E1ZZT3	enoyl-CoA hydratase	XI	Camponotus floridanus	
A0A0D2WU30	enoyl-CoA hydratase	XI	Capsaspora owczarzaki	
Q9AC55	enoyl-CoA hydratase	XI	Caulobacter crescentus	
B0T7D4	enoyl-CoA hydratase	XI	Caulobacter sp.	
Q2H6M6	enoyl-CoA hydratase	XI	Chaetomium globosum	
Q11AV7	enoyl-CoA hydratase	XI	Chelativorans sp.	4.2.1.17
C7PIE7	enoyl-CoA hydratase	XI	Chitinophaga pinensis	
B8G7Q4	enoyl-CoA hydratase	XI	Chloroflexus aggregans	
A9WBE8	enoyl-CoA hydratase	XI	Chloroflexus aurantiacus	
F6WAG8	enoyl-CoA hydratase	XI	Ciona intestinalis	

Table S2.5. (continuation)

UniProt	Family [3]	Cluster	Species	EC Number
D0DAB3	enoyl-CoA hydratase	XI	Citreicella sp.	4.2.1.17
J3KD95	enoyl-CoA hydratase	XI	Coccidioides immitis	
E3Q8C6	enoyl-CoA hydratase	XI	Colletotrichum graminicola	
Q485U2	enoyl-CoA hydratase	XI	Colwellia psychrerythraea	
D8DDW5	enoyl-CoA hydratase	XI	Comamonas testosteroni	4.2.1.17
D3F5E5	enoyl-CoA hydratase	XI	Conexibacter woesei	
A8N6E7	enoyl-CoA hydratase	XI	Coprinopsis cinerea	
D5P0B3	enoyl-CoA hydratase	XI	Corynebacterium ammoniagenes	
E2MYS6	enoyl-CoA hydratase	XI	Corynebacterium amycolatum	4.2.1.17
C3PF63	enoyl-CoA hydratase	XI	Corynebacterium aurimucosum	4.2.1.17
Q4JXA7	enoyl-CoA hydratase	XI	Corynebacterium jeikeium	4.2.1.17
C4LH94	enoyl-CoA hydratase	XI	Corynebacterium kroppenstedtii	4.2.1.17
C2CLY6	enoyl-CoA hydratase	XI	Corynebacterium striatum	4.2.1.17
B1VIG7	enoyl-CoA hydratase	XI	Corynebacterium urealyticum	4.2.1.17
G0HCY0	enoyl-CoA hydratase	XI	Corynebacterium variabile	
E6RAB9	enoyl-CoA hydratase	XI	Cryptococcus gattii	
Q6UP88	enoyl-CoA hydratase	XI	Cupriavidus pinatubonensis	
Q46WW3	enoyl-CoA hydratase	XI	Cupriavidus pinatubonensis	4.2.1.17
C9Y7M4	enoyl-CoA hydratase	XI	Curvibacter putative	4.2.1.17
Q7T1D9	enoyl-CoA hydratase	XI	Danio rerio	
E9GVE9	enoyl-CoA hydratase	XI	Daphnia pulex	
Q47JP6	enoyl-CoA hydratase	XI	Dechloromonas aromatica	4.2.1.17
E8UB20	enoyl-CoA hydratase	XI	Deinococcus maricopensis	4.2.1.17
Q54BX7	enoyl-CoA hydratase	XI	Dictyostelium discoideum	
Q1ZXF1	enoyl-CoA hydratase	XI	Dictyostelium discoideum	4.2.1.17
F0ZH22	enoyl-CoA hydratase	XI	Dictyostelium purpureum	
E6JBY1	enoyl-CoA hydratase	XI	Dietzia cinnamea	
A8LNK9	enoyl-CoA hydratase	XI	Dinoroseobacter shibae	4.2.1.17
B4KPW3	enoyl-CoA hydratase	XI	Drosophila mojavensis	
B4N5T9	enoyl-CoA hydratase	XI	Drosophila willistoni	
D8LKM6	enoyl-CoA hydratase	XI	Ectocarpus siliculosus	4.2.1.17
Q5B0L4	enoyl-CoA hydratase	XI	Emericella nidulans	
D2ZC01	enoyl-CoA hydratase	XI	Enterobacter cancerogenus	
E3G8U7	enoyl-CoA hydratase	XI	Enterobacter lignolyticus	
E5YBW1	enoyl-CoA hydratase	XI	Enterobacteriaceae bacterium	
Q2N892	enoyl-CoA hydratase	XI	Erythrobacter litoralis	
A3WEF3	enoyl-CoA hydratase	XI	Erythrobacter sp.	
A5PAX2	enoyl-CoA hydratase	XI	Erythrobacter sp.	
C1BW26	enoyl-CoA hydratase	XI	Esox lucius	
B1YIJ5	enoyl-CoA hydratase	XI	Exiguobacterium sibiricum	
Q0G5Y2	enoyl-CoA hydratase	XI	Fulvimarina pelagi	4.2.1.17
A0A0F6BLH9	enoyl-CoA hydratase	XI	Geobacillus sp.	
V6VGG9	enoyl-CoA hydratase	XI	Geobacillus sp.	
A0A098DGE7	enoyl-CoA hydratase	XI	Gibberella zeae	
D3TLZ0	enoyl-CoA hydratase	XI	Glossina morsitans	
D0L572	enoyl-CoA hydratase	XI	Gordonia bronchialis	
F1YIM4	enoyl-CoA hydratase	XI	Gordonia neofelifaecis	
F0XUE1	enoyl-CoA hydratase	XI	Grosmannia clavigera	
E1X2Y3	enoyl-CoA hydratase	XI	Halobacteriovorax marinus	
C8YX87	enoyl-CoA hydratase	XI	Halomonas sp.	
E2C9L5	enoyl-CoA hydratase	XI	Harpegnathos saltator	
C9S271	enoyl-CoA hydratase	XI	Heliconius melpomene	
D8ITH6	enoyl-CoA hydratase	XI	Herbaspirillum seropedicae	
D8IS16	enoyl-CoA hydratase	XI	Herbaspirillum seropedicae	4.2.1.17
C6XPW1	enoyl-CoA hydratase	XI	Hirschia baltica	
C6XRU8	enoyl-CoA hydratase	XI	Hirschia baltica	

Table S2.5. (continuation)

UniProt	Family [3]	Cluster	Species	EC Number
A9CWQ5	enoyl-CoA hydratase	XI	Hoeflea phototrophica	4.2.1.17
P30084	enoyl-CoA hydratase	XI	Homo sapiens	4.2.1.17
A0A059FX58	enoyl-CoA hydratase	XI	Hyphomonas hirschiana	
A0A059G0H5	enoyl-CoA hydratase	XI	Hyphomonas hirschiana	
E3TGN9	enoyl-CoA hydratase	XI	Ictalurus punctatus	4.2.1.17; 4.2.1.7
B7QBM8	enoyl-CoA hydratase	XI	Ixodes scapularis	4.2.1.17
Q28JI4	enoyl-CoA hydratase	XI	Jannaschia sp.	4.2.1.17
A6SUI3	enoyl-CoA hydratase	XI	Janthinobacterium sp.	4.2.1.17
A3E3X9	enoyl-CoA hydratase	XI	Karlodinium veneficum	
E4N1A7	enoyl-CoA hydratase	XI	Kitasatospora setae	4.2.1.17
Q4LEY5	enoyl-CoA hydratase	XI	Klebsiella sp.	
B2GKY0	enoyl-CoA hydratase	XI	Kocuria rhizophila	4.2.1.17
D6TH89	enoyl-CoA hydratase	XI	Ktedonobacter racemifer	
A0NUG3	enoyl-CoA hydratase	XI	Labrenzia aggregata	4.2.1.17
B0D8U0	enoyl-CoA hydratase	XI	Laccaria bicolor	
C1D5V1	enoyl-CoA hydratase	XI	Laribacter hongkongensis	
E7S0V3	enoyl-CoA hydratase	XI	Lautropia mirabilis	
A4HHN0	enoyl-CoA hydratase	XI	Leishmania braziliensis	
E9ALJ3	enoyl-CoA hydratase	XI	Leishmania mexicana	
C1BUV5	enoyl-CoA hydratase	XI	Lepeophtheirus salmonis	
E4ZI22	enoyl-CoA hydratase	XI	Leptosphaeria maculans	
B1Y4M0	enoyl-CoA hydratase	XI	Leptothrix cholodnii	
B1Y7U3	enoyl-CoA hydratase	XI	Leptothrix cholodnii	
A3V1Q7	enoyl-CoA hydratase	XI	Loktanella vestfoldensis	
G7N1G5	enoyl-CoA hydratase	XI	Macaca mulatta	
G4N8F1	enoyl-CoA hydratase	XI	Magnaporthe oryzae	
A4TVC2	enoyl-CoA hydratase	XI	Magnetospirillum gryph.	
A4TXE9	enoyl-CoA hydratase	XI	Magnetospirillum gryph.	
A4TXR8	enoyl-CoA hydratase	XI	Magnetospirillum gryph.	
A0A0C2U721	enoyl-CoA hydratase	XI	Magnetospirillum magnetot.	
A0A0C2YH38	enoyl-CoA hydratase	XI	Magnetospirillum magnetot.	
A8PTF1	enoyl-CoA hydratase	XI	Malassezia globosa	
Q0AK36	enoyl-CoA hydratase	XI	Maricaulis maris	4.2.1.17
A6F421	enoyl-CoA hydratase	XI	Marinobacter algicola	
A3JBF2	enoyl-CoA hydratase	XI	Marinobacter sp.	
A3YFB0	enoyl-CoA hydratase	XI	Marinomonas sp.	
A3YH16	enoyl-CoA hydratase	XI	Marinomonas sp.	
A6VZY2	enoyl-CoA hydratase	XI	Marinomonas sp.	
A3VCV1	enoyl-CoA hydratase	XI	Maritimibacter alkaliphilus	
A3VLN4	enoyl-CoA hydratase	XI	Maritimibacter alkaliphilus	4.2.1.17
E4TQJ5	enoyl-CoA hydratase	XI	Marivirga tractuosa	4.2.1.17
D7BFN9	enoyl-CoA hydratase	XI	Meiothermus silvanus	
A0A088E3K7	enoyl-CoA hydratase	XI	Metallosphaera sedula	4.2.1.17
A0A0B4IDU9	enoyl-CoA hydratase	XI	Metarhizium majus	
A2SDC0	enoyl-CoA hydratase	XI	Methylibium petroleiphilum	4.2.1.17
B8IUF4	enoyl-CoA hydratase	XI	Methylobacterium nodulans	
B1ZCT5	enoyl-CoA hydratase	XI	Methylobacterium populi	
B1LX51	enoyl-CoA hydratase	XI	Methylobacterium radiotolerans	
E8NCN0	enoyl-CoA hydratase	XI	Microbacterium testaceum	
D3LPP5	enoyl-CoA hydratase	XI	Micrococcus luteus	
C4RG31	enoyl-CoA hydratase	XI	Micromonospora sp.	
E2LNX1	enoyl-CoA hydratase	XI	Moniliophthora perniciosa	
A9UU77	enoyl-CoA hydratase	XI	Monosiga brevicollis	
Q8BH95	enoyl-CoA hydratase	XI	Mus musculus	4.2.1.17
A0A089T6W4	enoyl-CoA hydratase	XI	Mycobacterium abscessus	4.2.1.17
X8A4C7	enoyl-CoA hydratase	XI	Mycobacterium avium	

Table S2.5. (continuation)

UniProt	Family [3]	Cluster	Species	EC Number
P64017	enoyl-CoA hydratase	XI	Mycobacterium bovis	4.2.1.17
O07137	enoyl-CoA hydratase	XI	Mycobacterium leprae	4.2.1.17
A0A0D6IYN8	enoyl-CoA hydratase	XI	Mycobacterium smegmatis	4.2.1.17
A0R747	enoyl-CoA hydratase	XI	Mycobacterium smegmatis	4.2.1.17
A1UKR5	enoyl-CoA hydratase	XI	Mycobacterium sp.	4.2.1.17
A1TE56	enoyl-CoA hydratase	XI	Mycobacterium vanbaalenii	4.2.1.17
D2V1H4	enoyl-CoA hydratase	XI	Naegleria gruberi	
C7YR16	enoyl-CoA hydratase	XI	Nectria haematococca	
C7Z2N1	enoyl-CoA hydratase	XI	Nectria haematococca	
A7S886	enoyl-CoA hydratase	XI	Nematostella vectensis	
A1DAF7	enoyl-CoA hydratase	XI	Neosartorya fischeri	
A1DH12	enoyl-CoA hydratase	XI	Neosartorya fischeri	
Q2BHS2	enoyl-CoA hydratase	XI	Neptuniibacter caesariensis	
Q7RYW9	enoyl-CoA hydratase	XI	Neurospora crassa	
Q1QK06	enoyl-CoA hydratase	XI	Nitrobacter hamburgensis	4.2.1.17
A3X021	enoyl-CoA hydratase	XI	Nitrobacter sp.	
Q3SQD5	enoyl-CoA hydratase	XI	Nitrobacter winogradskyi	4.2.1.17
A4BNW9	enoyl-CoA hydratase	XI	Nitrococcus mobilis	4.2.1.17
Q5Z105	enoyl-CoA hydratase	XI	Nocardia farcinica	
Q2GA18	enoyl-CoA hydratase	XI	Novosphingobium aromat.	4.2.1.17
F1Z4G9	enoyl-CoA hydratase	XI	Novosphingobium nitrogenifigens	
F1ZD15	enoyl-CoA hydratase	XI	Novosphingobium nitrogenifigens	
A9DY61	enoyl-CoA hydratase	XI	Oceanibulbus indolifex	
A3UI34	enoyl-CoA hydratase	XI	Oceanicaulis sp.	4.2.1.17
A3TY09	enoyl-CoA hydratase	XI	Oceanicola batsensis	
Q2CDF9	enoyl-CoA hydratase	XI	Oceanicola granulosus	
M9REP0	enoyl-CoA hydratase	XI	Octadecabacter antarcticus	
E4XDY4	enoyl-CoA hydratase	XI	Oikopleura dioica	
E1IGB5	enoyl-CoA hydratase	XI	Oscillochloris trichoides	
W2EEY9	enoyl-CoA hydratase	XI	Paenibacillus larvae	4.2.1.17
C1G2P3	enoyl-CoA hydratase	XI	Paracoccidioides brasiliensis	
A1B645	enoyl-CoA hydratase	XI	Paracoccus denitrificans	4.2.1.17
A1BAN3	enoyl-CoA hydratase	XI	Paracoccus denitrificans	4.2.1.17
A0BCQ4	enoyl-CoA hydratase	XI	Paramecium tetraurelia	
A0CEE4	enoyl-CoA hydratase	XI	Paramecium tetraurelia	
A7HZC6	enoyl-CoA hydratase	XI	Parvibaculum lavamentivorans	
E0TEI9	enoyl-CoA hydratase	XI	Parvularcula bermudensis	
E0VN39	enoyl-CoA hydratase	XI	Pediculus humanus	4.2.1.17
Q0FVF4	enoyl-CoA hydratase	XI	Pelagibaca bermudensis	
B6HSG3	enoyl-CoA hydratase	XI	Penicillium rubens	
C5KCT6	enoyl-CoA hydratase	XI	Perkinsus marinus	
C5KK63	enoyl-CoA hydratase	XI	Perkinsus marinus	
C5L2Z5	enoyl-CoA hydratase	XI	Perkinsus marinus	
I7DW67	enoyl-CoA hydratase	XI	Phaeobacter inhibens	
B7GE26	enoyl-CoA hydratase	XI	Phaeodactylum tricornutum	4.2.1.17
Q0UJE7	enoyl-CoA hydratase	XI	Phaeosphaeria nodorum	
Q0UWB3	enoyl-CoA hydratase	XI	Phaeosphaeria nodorum	
B4RC45	enoyl-CoA hydratase	XI	Phenylobacterium zucineum	
A9TBR6	enoyl-CoA hydratase	XI	Physcomitrella patens	
D0NW02	enoyl-CoA hydratase	XI	Phytophthora infestans	
B2AXD7	enoyl-CoA hydratase	XI	Podospora anserina	
A1VP59	enoyl-CoA hydratase	XI	Polaromonas naphthalenivorans	4.2.1.17
A1VSN8	enoyl-CoA hydratase	XI	Polaromonas naphthalenivorans	4.2.1.17
Q12EG8	enoyl-CoA hydratase	XI	Polaromonas sp.	4.2.1.17
F2IVN1	enoyl-CoA hydratase	XI	Polymorphum gilvum	
F2J4G8	enoyl-CoA hydratase	XI	Polymorphum gilvum	4.2.1.17

Table S2.5. (continuation)

UniProt	Family [3]	Cluster	Species	EC Number
B1XSZ8	enoyl-CoA hydratase	XI	Polynucleobacter necessarius	
A4SZ58	enoyl-CoA hydratase	XI	Polynucleobacter necessarius	4.2.1.17
B8PH17	enoyl-CoA hydratase	XI	Postia placenta	
B6XHB6	enoyl-CoA hydratase	XI	Providencia alcalifaciens	
D4C2I4	enoyl-CoA hydratase	XI	Providencia rettgeri	
D1P0Z2	enoyl-CoA hydratase	XI	Providencia rustigianii	
B2PUH7	enoyl-CoA hydratase	XI	Providencia stuartii	
A6V1K0	enoyl-CoA hydratase	XI	Pseudomonas aeruginosa	
Q9HY35	enoyl-CoA hydratase	XI	Pseudomonas aeruginosa	
Q1I9V4	enoyl-CoA hydratase	XI	Pseudomonas entomophila	4.2.1.17
E9M090	enoyl-CoA hydratase	XI	Pseudomonas fluorescens	
E2XRW5	enoyl-CoA hydratase	XI	Pseudomonas fluorescens	4.2.1.17
Q3KC98	enoyl-CoA hydratase	XI	Pseudomonas fluorescens	4.2.1.17
B1J8Q4	enoyl-CoA hydratase	XI	Pseudomonas putida	
B1JBL8	enoyl-CoA hydratase	XI	Pseudomonas putida	
A5W3A0	enoyl-CoA hydratase	XI	Pseudomonas putida	4.2.1.17
Q48JK6	enoyl-CoA hydratase	XI	Pseudomonas savastanoi	4.2.1.17
Q70IM9	enoyl-CoA hydratase	XI	Pseudomonas sp.	
Q845K2	enoyl-CoA hydratase	XI	Pseudomonas sp.	
A4VKU8	enoyl-CoA hydratase	XI	Pseudomonas stutzeri	
Q880J4	enoyl-CoA hydratase	XI	Pseudomonas syringae	
G8PK98	enoyl-CoA hydratase	XI	Pseudovibrio sp.	
B6YYV3	enoyl-CoA hydratase	XI	Pseudovibrio sp.	4.2.1.17
Q1QBD8	enoyl-CoA hydratase	XI	Psychrobacter cryohalolentis	4.2.1.17
A1SSP3	enoyl-CoA hydratase	XI	Psychromonas ingrahamii	
E3KGL2	enoyl-CoA hydratase	XI	Puccinia graminis	
E3RKE9	enoyl-CoA hydratase	XI	Pyrenophora teres	
B2VX94	enoyl-CoA hydratase	XI	Pyrenophora tritici-repentis	
B2WEG2	enoyl-CoA hydratase	XI	Pyrenophora tritici-repentis	
H8NQG2	enoyl-CoA hydratase	XI	Rahnella aquatilis	
U3G9E1	enoyl-CoA hydratase	XI	Ralstonia sp.	
A9WQX5	enoyl-CoA hydratase	XI	Renibacterium salmoninarum	4.2.1.17
B5ZYL0	enoyl-CoA hydratase	XI	Rhizobium leguminosarum	
B6A4Z1	enoyl-CoA hydratase	XI	Rhizobium leguminosarum	
Q98BG7	enoyl-CoA hydratase	XI	Rhizobium loti	
Q98LI4	enoyl-CoA hydratase	XI	Rhizobium loti	
Q52995	enoyl-CoA hydratase	XI	Rhizobium meliloti	4.2.1.17
A0A061MQ98	enoyl-CoA hydratase	XI	Rhizobium rhizogenes	
D5ATE6	enoyl-CoA hydratase	XI	Rhodobacter capsulatus	4.2.1.17
C8RX84	enoyl-CoA hydratase	XI	Rhodobacter sp.	
A4WVR5	enoyl-CoA hydratase	XI	Rhodobacter sphaeroides	4.2.1.17
A3JM08	enoyl-CoA hydratase	XI	Rhodobacteraceae bacterium	
B6AV33	enoyl-CoA hydratase	XI	Rhodobacteraceae bacterium	4.2.1.17
B9NUJ5	enoyl-CoA hydratase	XI	Rhodobacteraceae bacterium	4.2.1.17
B9NVR2	enoyl-CoA hydratase	XI	Rhodobacteraceae bacterium	4.2.1.17
Q0FCH8	enoyl-CoA hydratase	XI	Rhodobacterales bacterium	
E9SYN0	enoyl-CoA hydratase	XI	Rhodococcus equi	
Q0SGI5	enoyl-CoA hydratase	XI	Rhodococcus jostii	
C1B644	enoyl-CoA hydratase	XI	Rhodococcus opacus	4.2.1.17
A0A069JLN8	enoyl-CoA hydratase	XI	Rhodococcus qingshengii	4.2.1.17
J2J201	enoyl-CoA hydratase	XI	Rhodococcus sp.	
Q21Y48	enoyl-CoA hydratase	XI	Rhodoferax ferrireducens	4.2.1.17
E3I397	enoyl-CoA hydratase	XI	Rhodomicrobium vannielii	
E3I3E9	enoyl-CoA hydratase	XI	Rhodomicrobium vannielii	
E6VJ27	enoyl-CoA hydratase	XI	Rhodopseudomonas palustris	
E6VL87	enoyl-CoA hydratase	XI	Rhodopseudomonas palustris	

Table S2.5. (continuation)

UniProt	Family [3]	Cluster	Species	EC Number
Q07U11	enoyl-CoA hydratase	XI	Rhodopseudomonas palustris	4.2.1.-
Q13AV7	enoyl-CoA hydratase	XI	Rhodopseudomonas palustris	4.2.1.-
Q6N399	enoyl-CoA hydratase	XI	Rhodopseudomonas palustris	4.2.1.-
Q217Y9	enoyl-CoA hydratase	XI	Rhodopseudomonas palustris	4.2.1.17
Q2IU58	enoyl-CoA hydratase	XI	Rhodopseudomonas palustris	4.2.1.17
B6IVU6	enoyl-CoA hydratase	XI	Rhodospirillum centenum	4.2.1.17
Q2RMQ0	enoyl-CoA hydratase	XI	Rhodospirillum rubrum	4.2.1.17
E2CDH8	enoyl-CoA hydratase	XI	Roseibium sp.	
E2CE74	enoyl-CoA hydratase	XI	Roseibium sp.	
Q16DK9	enoyl-CoA hydratase	XI	Roseobacter denitrificans	4.2.1.17
A4EFZ5	enoyl-CoA hydratase	XI	Roseobacter sp.	
A6FKX8	enoyl-CoA hydratase	XI	Roseobacter sp.	
D5RLF9	enoyl-CoA hydratase	XI	Roseomonas cervicalis	4.2.1.17
A3SKA6	enoyl-CoA hydratase	XI	Roseovarius nubinhibens	
A6E1I1	enoyl-CoA hydratase	XI	Roseovarius sp.	
D0CR34	enoyl-CoA hydratase	XI	Ruegeria lacuscaerulensis	4.2.1.17
Q5LWT8	enoyl-CoA hydratase	XI	Ruegeria pomeroyi	4.2.1.17
A3K328	enoyl-CoA hydratase	XI	Sagittula stellata	
A3K7Y3	enoyl-CoA hydratase	XI	Sagittula stellata	4.2.1.17
A8M212	enoyl-CoA hydratase	XI	Salinispora arenicola	
A4X301	enoyl-CoA hydratase	XI	Salinispora tropica	
F2U5J0	enoyl-CoA hydratase	XI	Salpingoeca rosetta	
D8QBU2	enoyl-CoA hydratase	XI	Schizophyllum commune	
D8S6B8	enoyl-CoA hydratase	XI	Selaginella moellendorffii	
D4E4J4	enoyl-CoA hydratase	XI	Serratia odorifera	4.2.1.17
S0AF73	enoyl-CoA hydratase	XI	Serratia plymuthica	4.2.1.17
A8GGD8	enoyl-CoA hydratase	XI	Serratia proteamaculans	
E9IFF6	enoyl-CoA hydratase	XI	Solenopsis invicta	
D1C848	enoyl-CoA hydratase	XI	Sphaerobacter thermophilus	
F6EUP7	enoyl-CoA hydratase	XI	Sphingobium chlorophenolicum	
D4Z2S5	enoyl-CoA hydratase	XI	Sphingobium japonicum	4.2.1.17
Q1NB15	enoyl-CoA hydratase	XI	Sphingomonas sp.	
A5V400	enoyl-CoA hydratase	XI	Sphingomonas wittichii	4.2.1.17
Q1GNL3	enoyl-CoA hydratase	XI	Sphingopyxis alaskensis	4.2.1.17
D7A493	enoyl-CoA hydratase	XI	Starkeya novella	
D6B1W9	enoyl-CoA hydratase	XI	Streptomyces albus	
D7CFQ8	enoyl-CoA hydratase	XI	Streptomyces bingchenggensis	
B5GMB2	enoyl-CoA hydratase	XI	Streptomyces clavuligerus	4.2.1.17
B1VV64	enoyl-CoA hydratase	XI	Streptomyces griseus	
D9WH62	enoyl-CoA hydratase	XI	Streptomyces himastatinicus	
C9ZCA1	enoyl-CoA hydratase	XI	Streptomyces scabiei	
D6KDQ8	enoyl-CoA hydratase	XI	Streptomyces sp.	
D9UN16	enoyl-CoA hydratase	XI	Streptomyces sp.	
B4V5S3	enoyl-CoA hydratase	XI	Streptomyces sp.	4.2.1.17
D9X7U2	enoyl-CoA hydratase	XI	Streptomyces viridochromogenes	
A0A0B4J2W3	enoyl-CoA hydratase	XI	Strongylocentrotus purpuratus	
Q4J6K9	enoyl-CoA hydratase	XI	Sulfolobus acidocaldarius	
C3N211	enoyl-CoA hydratase	XI	Sulfolobus islandicus	
M9UCV4	enoyl-CoA hydratase	XI	Sulfolobus islandicus	
Q67SZ6	enoyl-CoA hydratase	XI	Symbiobacterium thermophilum	
B5FXI3	enoyl-CoA hydratase	XI	Taeniopygia guttata	
A0A093VN83	enoyl-CoA hydratase	XI	Talaromyces marneffei	
I7M0C0	enoyl-CoA hydratase	XI	Tetrahymena thermophila	
B8C2B7	enoyl-CoA hydratase	XI	Thalassiosira pseudonana	
C7DF67	enoyl-CoA hydratase	XI	Thalassobium sp.	4.2.1.17
Q9AJS7	enoyl-CoA hydratase	XI	Thauera aromatica	

Table S2.5. (continuation)

UniProt	Family [3]	Cluster	Species	EC Number
A0A0C3IX24	enoyl-CoA hydratase	XI	Thauera sp.	
C4K935	enoyl-CoA hydratase	XI	Thauera sp.	
K6QFD5	enoyl-CoA hydratase	XI	Thermaerobacter subterraneus	
D6CR47	enoyl-CoA hydratase	XI	Thiomonas arsenitoxydans	4.2.1.17
S7UFF2	enoyl-CoA hydratase	XI	Toxoplasma gondii	4.2.1.17
D6WVI1	enoyl-CoA hydratase	XI	Tribolium castaneum	
E5SFS7	enoyl-CoA hydratase	XI	Trichinella spiralis	
B3RM37	enoyl-CoA hydratase	XI	Trichoplax adhaerens	
Q582Q0	enoyl-CoA hydratase	XI	Trypanosoma brucei	4.2.1.17
Q4E679	enoyl-CoA hydratase	XI	Trypanosoma cruzi	4.2.1.17
D5G6G8	enoyl-CoA hydratase	XI	Tuber melanosporum	
E0XUZ1	enoyl-CoA hydratase	XI	uncultured gamma	
E7C432	enoyl-CoA hydratase	XI	uncultured gamma	
E7C7K5	enoyl-CoA hydratase	XI	uncultured gamma	
E7C834	enoyl-CoA hydratase	XI	uncultured gamma	
E7C8P6	enoyl-CoA hydratase	XI	uncultured gamma	
C5CLZ2	enoyl-CoA hydratase	XI	Variovorax paradoxus	
A1WF39	enoyl-CoA hydratase	XI	Verminephrobacter eiseniae	4.2.1.17
C9SAG0	enoyl-CoA hydratase	XI	Verticillium alfalfae	
E3BPI3	enoyl-CoA hydratase	XI	Vibrio caribbeanicus	
A7IDR6	enoyl-CoA hydratase	XI	Xanthobacter autotrophicus	
A7IKJ4	enoyl-CoA hydratase	XI	Xanthobacter autotrophicus	
Q6GLG0	enoyl-CoA hydratase	XI	Xenopus tropicalis	
D3UY95	enoyl-CoA hydratase	XI	Xenorhabdus bovienii	4.2.1.17
N1NUG1	enoyl-CoA hydratase	XI	Xenorhabdus nematophila	4.2.1.17
Q6CF43	enoyl-CoA hydratase	XI	Yarrowia lipolytica	
A0A0A8XIG1	feruloyl-CoA hydratase/lyase	IV	Acinetobacter calcoaceticus	
B9JVE2	feruloyl-CoA hydratase/lyase	IV	Agrobacterium vitis	
Q9EY87	feruloyl-CoA hydratase/lyase	IV	Amycolatopsis sp.	
D3P2L1	feruloyl-CoA hydratase/lyase	IV	Azospirillum sp.	4.2.1.17
C1DQW6	feruloyl-CoA hydratase/lyase	IV	Azotobacter vinelandii	
D9QKS0	feruloyl-CoA hydratase/lyase	IV	Brevundimonas subvibrioides	
B2TE87	feruloyl-CoA hydratase/lyase	IV	Burkholderia phytofirmans	
A0A0F5KB95	feruloyl-CoA hydratase/lyase	IV	Burkholderia pseudomallei	
A0A0D0HF72	feruloyl-CoA hydratase/lyase	IV	Burkholderia sp.	
B5WN65	feruloyl-CoA hydratase/lyase	IV	Burkholderia sp.	
E8YS32	feruloyl-CoA hydratase/lyase	IV	Burkholderia sp.	
D5VDG3	feruloyl-CoA hydratase/lyase	IV	Caulobacter segnis	
D5VLZ1	feruloyl-CoA hydratase/lyase	IV	Caulobacter segnis	
B0SXT9	feruloyl-CoA hydratase/lyase	IV	Caulobacter sp.	
Q1R0W7	feruloyl-CoA hydratase/lyase	IV	Chromohalobacter salexigens	
A9BTY2	feruloyl-CoA hydratase/lyase	IV	Delftia acidovorans	
B0U9L1	feruloyl-CoA hydratase/lyase	IV	Methylobacterium sp.	
Q2G7R4	feruloyl-CoA hydratase/lyase	IV	Novosphingobium aromat.	
O69762	feruloyl-CoA hydratase/lyase	IV	Pseudomonas fluorescens	4.1.2.41; 4.2.1.1
C3VA24	feruloyl-CoA hydratase/lyase	IV	Pseudomonas nitroreducens	
Q70V08	feruloyl-CoA hydratase/lyase	IV	Pseudomonas putida	
O05618	feruloyl-CoA hydratase/lyase	IV	Pseudomonas sp.	
Q8XT90	feruloyl-CoA hydratase/lyase	IV	Ralstonia solanacearum	
B6A0K2	feruloyl-CoA hydratase/lyase	IV	Rhizobium leguminosarum	
Q98JP8	feruloyl-CoA hydratase/lyase	IV	Rhizobium loti	
C7MUI1	feruloyl-CoA hydratase/lyase	IV	Saccharomonospora viridis	
T2RXF8	feruloyl-CoA hydratase/lyase	IV	Saccharopolyspora erythraea	4.2.1.17
Q02CE9	feruloyl-CoA hydratase/lyase	IV	Solibacter usitatus	
F6F1R8	feruloyl-CoA hydratase/lyase	IV	Sphingobium chlorophenolicum	
Q8RR26	feruloyl-CoA hydratase/lyase	IV	Sphingomonas paucimobilis	

Table S2.5. (continuation)

UniProt	Family [3]	Cluster	Species	EC Number
Q8RR28	feruloyl-CoA hydratase/lyase	IV	Sphingomonas paucimobilis	
D2UGL1	feruloyl-CoA hydratase/lyase	IV	Xanthomonas albilineans	4.2.1.17
Z4WJ71	feruloyl-CoA hydratase/lyase	IV	Xanthomonas axonopodis	
C7LZ14	methylglutaconyl-CoA hydratase 2	XII	Acidimicrobium ferrooxidans	
C6WRC4	methylglutaconyl-CoA hydratase 2	XII	Actinosynnema mirum	
A3HYH6	methylglutaconyl-CoA hydratase 2	XII	Algoriphagus machipongonensis	
A8TM08	methylglutaconyl-CoA hydratase 2	XII	alpha proteobacterium	
D3PR97	methylglutaconyl-CoA hydratase 2	XII	Meiothermus ruber	
D7BDZ3	methylglutaconyl-CoA hydratase 2	XII	Meiothermus silvanus	
V7MY98	methylglutaconyl-CoA hydratase 2	XII	Mycobacterium avium	4.2.1.17
E4U535	methylglutaconyl-CoA hydratase 2	XII	Oceanithermus profundus	
B4RIG7	methylglutaconyl-CoA hydratase 2	XII	Phenylobacterium zucineum	
D0MI48	methylglutaconyl-CoA hydratase 2	XII	Rhodothermus marinus	
B7A9W0	methylglutaconyl-CoA hydratase 2	XII	Thermus aquaticus	4.2.1.17
E8PNH8	methylglutaconyl-CoA hydratase 2	XII	Thermus scotoductus	
H9ZSU3	methylglutaconyl-CoA hydratase 2	XII	Thermus thermophilus	
D7CY55	methylglutaconyl-CoA hydratase 2	XII	Truepera radiovictrix	
C0G3F0	methylglutaconyl-CoA hydratase 2	X	Brucella ceti	
E8UBK7	methylglutaconyl-CoA hydratase 2	X	Deinococcus maricopensis	
E1VL69	methylglutaconyl-CoA hydratase 2	X	gamma proteobacterium	
Q27Q49	methylglutaconyl-CoA hydratase 2	IX	Acanthamoeba castellanii	
C7RQF6	methylglutaconyl-CoA hydratase 2	IX	Accumulibacter phosphatis	
D4XBC2	methylglutaconyl-CoA hydratase 2	IX	Achromobacter piechaudii	4.2.1.17
E3HM25	methylglutaconyl-CoA hydratase 2	IX	Achromobacter xylosoxidans	
E5U1N7	methylglutaconyl-CoA hydratase 2	IX	Achromobacter xylosoxidans	4.2.1.17
A0A066PX63	methylglutaconyl-CoA hydratase 2	IX	Acidiphilium sp.	
C1F7A5	methylglutaconyl-CoA hydratase 2	IX	Acidobacterium capsulatum	
A1TVA5	methylglutaconyl-CoA hydratase 2	IX	Acidovorax citrulli	
C5T2N5	methylglutaconyl-CoA hydratase 2	IX	Acidovorax delafieldii	
A1WC95	methylglutaconyl-CoA hydratase 2	IX	Acidovorax sp.	
A0A010PU54	methylglutaconyl-CoA hydratase 2	IX	Acinetobacter baumannii	
F0KKW4	methylglutaconyl-CoA hydratase 2	IX	Acinetobacter calcoaceticus	
D0SC71	methylglutaconyl-CoA hydratase 2	IX	Acinetobacter johnsonii	
D8JM02	methylglutaconyl-CoA hydratase 2	IX	Acinetobacter oleivorans	
N9Q1L7	methylglutaconyl-CoA hydratase 2	IX	Acinetobacter sp.	
Q3HW12	methylglutaconyl-CoA hydratase 2	IX	Acinetobacter sp.	4.2.1.18
A0A0A5LA58	methylglutaconyl-CoA hydratase 2	IX	Aeromonas hydrophila	
A4SM62	methylglutaconyl-CoA hydratase 2	IX	Aeromonas salmonicida	
B9K5X0	methylglutaconyl-CoA hydratase 2	IX	Agrobacterium vitis	
Q0VQ62	methylglutaconyl-CoA hydratase 2	IX	Alcanivorax borkumensis	4.2.1.17
B4X0S6	methylglutaconyl-CoA hydratase 2	IX	Alcanivorax sp.	
E8TS32	methylglutaconyl-CoA hydratase 2	IX	Alicycliphilus denitrificans	
E8TV79	methylglutaconyl-CoA hydratase 2	IX	Alicycliphilus denitrificans	
A8TZE3	methylglutaconyl-CoA hydratase 2	IX	alpha proteobacterium	
A0A0E0YAK9	methylglutaconyl-CoA hydratase 2	IX	Alteromonas macleodii	
Q5P3D1	methylglutaconyl-CoA hydratase 2	IX	Aromatoleum aromaticum	4.2.1.17
E8RLE7	methylglutaconyl-CoA hydratase 2	IX	Asticcacaulis excentricus	
Q1YN40	methylglutaconyl-CoA hydratase 2	IX	Aurantimonas manganoxydans	
A1KA30	methylglutaconyl-CoA hydratase 2	IX	Azoarcus sp.	4.2.1.17
A8HZK2	methylglutaconyl-CoA hydratase 2	IX	Azorhizobium caulinodans	
D3NZC9	methylglutaconyl-CoA hydratase 2	IX	Azospirillum sp.	4.2.1.17
C1DL55	methylglutaconyl-CoA hydratase 2	IX	Azotobacter vinelandii	
Q6MHG6	methylglutaconyl-CoA hydratase 2	IX	Bdellovibrio bacteriovorus	4.2.1.17
Q1MXN1	methylglutaconyl-CoA hydratase 2	IX	Bermanella marisrubri	
A0A0A0XIA0	methylglutaconyl-CoA hydratase 2	IX	Bordetella pertussis	4.2.1.17
A9IP53	methylglutaconyl-CoA hydratase 2	IX	Bordetella petrii	4.2.1.17

Table S2.5. (continuation)

UniProt	Family [3]	Cluster	Species	EC Number
Q89Y39	methylglutaconyl-CoA hydratase 2	IX	Bradyrhizobium diazoefficiens	
B4WE95	methylglutaconyl-CoA hydratase 2	IX	Brevundimonas sp.	
D9QGS7	methylglutaconyl-CoA hydratase 2	IX	Brevundimonas subvibrioides	
A2WF94	methylglutaconyl-CoA hydratase 2	IX	Burkholderia dolosa	
A2WGG4	methylglutaconyl-CoA hydratase 2	IX	Burkholderia dolosa	
C5AHY9	methylglutaconyl-CoA hydratase 2	IX	Burkholderia glumae	
B9B8Z1	methylglutaconyl-CoA hydratase 2	IX	Burkholderia multivorans	
A0A088X8H7	methylglutaconyl-CoA hydratase 2	IX	Burkholderia oklahomensis	
B2JM09	methylglutaconyl-CoA hydratase 2	IX	Burkholderia phymatum	
E5AVD2	methylglutaconyl-CoA hydratase 2	IX	Burkholderia rhizoxinica	4.2.1.18
D5WIJ1	methylglutaconyl-CoA hydratase 2	IX	Burkholderia sp.	
E1TES3	methylglutaconyl-CoA hydratase 2	IX	Burkholderia sp.	
F0G2F0	methylglutaconyl-CoA hydratase 2	IX	Burkholderia sp.	
Q13GM5	methylglutaconyl-CoA hydratase 2	IX	Burkholderia xenovorans	4.2.1.17
Q13LP7	methylglutaconyl-CoA hydratase 2	IX	Burkholderia xenovorans	4.2.1.17
Q9A6C5	methylglutaconyl-CoA hydratase 2	IX	Caulobacter crescentus	
D5VL94	methylglutaconyl-CoA hydratase 2	IX	Caulobacter segnis	
B0T2M9	methylglutaconyl-CoA hydratase 2	IX	Caulobacter sp.	
C7PUC2	methylglutaconyl-CoA hydratase 2	IX	Chitinophaga pinensis	
B8GAW0	methylglutaconyl-CoA hydratase 2	IX	Chloroflexus aggregans	
A9WB03	methylglutaconyl-CoA hydratase 2	IX	Chloroflexus aurantiacus	
A0A0C1CEJ6	methylglutaconyl-CoA hydratase 2	IX	Chromobacterium piscinae	4.2.1.17
D0D471	methylglutaconyl-CoA hydratase 2	IX	Citreicella sp.	
Q485C2	methylglutaconyl-CoA hydratase 2	IX	Colwellia psychrerythraea	
B7WX38	methylglutaconyl-CoA hydratase 2	IX	Comamonas testosteroni	
A4A6C0	methylglutaconyl-CoA hydratase 2	IX	Congregibacter litoralis	4.2.1.18
Q83CX5	methylglutaconyl-CoA hydratase 2	IX	Coxiella burnetii	4.2.1.18
Q1LS77	methylglutaconyl-CoA hydratase 2	IX	Cupriavidus metallidurans	4.2.1.17
Q0KF90	methylglutaconyl-CoA hydratase 2	IX	Cupriavidus necator	4.2.1.17
Q471U9	methylglutaconyl-CoA hydratase 2	IX	Cupriavidus pinatubonensis	
Q11QU9	methylglutaconyl-CoA hydratase 2	IX	Cytophaga hutchinsonii	4.2.1.18
Q47J39	methylglutaconyl-CoA hydratase 2	IX	Dechloromonas aromatica	
Q47JY2	methylglutaconyl-CoA hydratase 2	IX	Dechloromonas aromatica	
A0A031IBV2	methylglutaconyl-CoA hydratase 2	IX	Delftia sp.	
A8LIT2	methylglutaconyl-CoA hydratase 2	IX	Dinoroseobacter shibae	4.2.1.18
C8PXY2	methylglutaconyl-CoA hydratase 2	IX	Enhydrobacter aerosaccus	
E1SU54	methylglutaconyl-CoA hydratase 2	IX	Ferrimonas balearica	4.2.1.18
B8KNS0	methylglutaconyl-CoA hydratase 2	IX	gamma proteobacterium	
Q1YSE9	methylglutaconyl-CoA hydratase 2	IX	gamma proteobacterium	
Q39QH0	methylglutaconyl-CoA hydratase 2	IX	Geobacter metallireducens	
G8P1A5	methylglutaconyl-CoA hydratase 2	IX	Granulicella mallensis	4.2.1.17
E8X3I1	methylglutaconyl-CoA hydratase 2	IX	Granulicella tundricola	
Q2SFA9	methylglutaconyl-CoA hydratase 2	IX	Hahella chejuensis	
E1X2N9	methylglutaconyl-CoA hydratase 2	IX	Halobacteriovorax marinus	
E1V3Y8	methylglutaconyl-CoA hydratase 2	IX	Halomonas elongata	4.2.1.17
D8J0K8	methylglutaconyl-CoA hydratase 2	IX	Herbaspirillum seropedicae	4.2.1.17
A9AYB9	methylglutaconyl-CoA hydratase 2	IX	Herpetosiphon aurantiacus	
C6XL99	methylglutaconyl-CoA hydratase 2	IX	Hirschia baltica	
A0A059FZ76	methylglutaconyl-CoA hydratase 2	IX	Hyphomonas hirschiana	
A3WM36	methylglutaconyl-CoA hydratase 2	IX	Idiomarina baltica	
Q5QW26	methylglutaconyl-CoA hydratase 2	IX	Idiomarina loihiensis	
Q28T95	methylglutaconyl-CoA hydratase 2	IX	Jannaschia sp.	4.2.1.18
C7RB34	methylglutaconyl-CoA hydratase 2	IX	Kangiella koreensis	
Q1IRS2	methylglutaconyl-CoA hydratase 2	IX	Koribacter versatilis	
D6TPI3	methylglutaconyl-CoA hydratase 2	IX	Ktedonobacter racemifer	
A0NYT7	methylglutaconyl-CoA hydratase 2	IX	Labrenzia aggregata	4.2.1.17

Table S2.5. (continuation)

UniProt	Family [3]	Cluster	Species	EC Number
B9QSS1	methylglutaconyl-CoA hydratase 2	IX	Labrenzia alexandrii	
C1D4I8	methylglutaconyl-CoA hydratase 2	IX	Laribacter hongkongensis	4.2.1.17
D3HRA0	methylglutaconyl-CoA hydratase 2	IX	Legionella longbeachae	4.2.1.17
D5TE84	methylglutaconyl-CoA hydratase 2	IX	Legionella pneumophila	4.2.1.17
B1Y2P2	methylglutaconyl-CoA hydratase 2	IX	Leptothrix cholodnii	
B1Y4M6	methylglutaconyl-CoA hydratase 2	IX	Leptothrix cholodnii	
B1Y520	methylglutaconyl-CoA hydratase 2	IX	Leptothrix cholodnii	
A6GLN9	methylglutaconyl-CoA hydratase 2	IX	Limnobacter sp.	4.2.1.17
A3V7M1	methylglutaconyl-CoA hydratase 2	IX	Loktanella vestfoldensis	4.2.1.17
B8KXY4	methylglutaconyl-CoA hydratase 2	IX	Luminiphilus syltensis	
A4TZG6	methylglutaconyl-CoA hydratase 2	IX	Magnetospirillum gryph.	
A4U4P0	methylglutaconyl-CoA hydratase 2	IX	Magnetospirillum gryph.	
Q2W9G1	methylglutaconyl-CoA hydratase 2	IX	Magnetospirillum magneticum	
A0A0C2UCB2	methylglutaconyl-CoA hydratase 2	IX	Magnetospirillum magnetot.	
A0A0C2YBW3	methylglutaconyl-CoA hydratase 2	IX	Magnetospirillum magnetot.	
Q0ANW6	methylglutaconyl-CoA hydratase 2	IX	Maricaulis maris	
A0YEY7	methylglutaconyl-CoA hydratase 2	IX	marine gamma	
B7S163	methylglutaconyl-CoA hydratase 2	IX	marine gamma	
E4PFL8	methylglutaconyl-CoA hydratase 2	IX	Marinobacter adhaerens	
E4PMN6	methylglutaconyl-CoA hydratase 2	IX	Marinobacter adhaerens	
A6F2K8	methylglutaconyl-CoA hydratase 2	IX	Marinobacter algicola	
A1U2I4	methylglutaconyl-CoA hydratase 2	IX	Marinobacter hydrocarbonoclasticus	
A3JHM5	methylglutaconyl-CoA hydratase 2	IX	Marinobacter sp.	
F2K4Q9	methylglutaconyl-CoA hydratase 2	IX	Marinomonas mediterranea	
A3Y9P6	methylglutaconyl-CoA hydratase 2	IX	Marinomonas sp.	
A3VI85	methylglutaconyl-CoA hydratase 2	IX	Maritimibacter alkaliphilus	4.2.1.17
E4TM80	methylglutaconyl-CoA hydratase 2	IX	Marivirga tractuosa	4.2.1.18
A2SE54	methylglutaconyl-CoA hydratase 2	IX	Methylibium petroleiphilum	4.2.1.17
A2SL72	methylglutaconyl-CoA hydratase 2	IX	Methylibium petroleiphilum	4.2.1.18
B8IXH8	methylglutaconyl-CoA hydratase 2	IX	Methylobacterium nodulans	
A9V763	methylglutaconyl-CoA hydratase 2	IX	Monosiga brevicollis	
A6FGF2	methylglutaconyl-CoA hydratase 2	IX	Moritella sp.	
Q2BQ71	methylglutaconyl-CoA hydratase 2	IX	Neptuniibacter caesariensis	
A4BVQ0	methylglutaconyl-CoA hydratase 2	IX	Nitrococcus mobilis	
D5BW06	methylglutaconyl-CoA hydratase 2	IX	Nitrosococcus halophilus	
A0A0E2Z0N1	methylglutaconyl-CoA hydratase 2	IX	Nitrosococcus oceani	4.2.1.17
D8PHS9	methylglutaconyl-CoA hydratase 2	IX	Nitrospira defluvii	4.2.1.17
A9E3E4	methylglutaconyl-CoA hydratase 2	IX	Oceanibulbus indolifex	4.2.1.17
A3UJT4	methylglutaconyl-CoA hydratase 2	IX	Oceanicaulis sp.	
A3TZJ0	methylglutaconyl-CoA hydratase 2	IX	Oceanicola batsensis	4.2.1.17
A3U1L5	methylglutaconyl-CoA hydratase 2	IX	Oceanicola batsensis	4.2.1.17
Q2CES7	methylglutaconyl-CoA hydratase 2	IX	Oceanicola granulosus	4.2.1.17
A6WUW8	methylglutaconyl-CoA hydratase 2	IX	Ochrobactrum anthropi	
M9RBD5	methylglutaconyl-CoA hydratase 2	IX	Octadecabacter antarcticus	
M9RMD2	methylglutaconyl-CoA hydratase 2	IX	Octadecabacter arcticus	
A1B865	methylglutaconyl-CoA hydratase 2	IX	Paracoccus denitrificans	4.2.1.18
C6Y038	methylglutaconyl-CoA hydratase 2	IX	Pedobacter heparinus	
A6ECC8	methylglutaconyl-CoA hydratase 2	IX	Pedobacter sp.	
Q0FNW6	methylglutaconyl-CoA hydratase 2	IX	Pelagibaca bermudensis	4.2.1.17
I7DNH6	methylglutaconyl-CoA hydratase 2	IX	Phaeobacter gallaeciensis	
B4R933	methylglutaconyl-CoA hydratase 2	IX	Phenylobacterium zucineum	
Q1Z6G7	methylglutaconyl-CoA hydratase 2	IX	Photobacterium profundum	
Q2C752	methylglutaconyl-CoA hydratase 2	IX	Photobacterium sp.	
A1VJG7	methylglutaconyl-CoA hydratase 2	IX	Polaromonas naphthalenivorans	
A1VRG2	methylglutaconyl-CoA hydratase 2	IX	Polaromonas naphthalenivorans	
Q12OB6	methylglutaconyl-CoA hydratase 2	IX	Polaromonas sp.	

Table S2.5. (continuation)

UniProt	Family [3]	Cluster	Species	EC Number
Q124J1	methylglutaconyl-CoA hydratase 2	IX	Polaromonas sp.	
Q12D25	methylglutaconyl-CoA hydratase 2	IX	Polaromonas sp.	
F2J257	methylglutaconyl-CoA hydratase 2	IX	Polymorphum gilvum	
Q15RQ3	methylglutaconyl-CoA hydratase 2	IX	Pseudoalteromonas atlantica	
Q3IGB0	methylglutaconyl-CoA hydratase 2	IX	Pseudoalteromonas haloplanktis	4.2.1.17
A4C9N0	methylglutaconyl-CoA hydratase 2	IX	Pseudoalteromonas tunicata	
B9Z368	methylglutaconyl-CoA hydratase 2	IX	Pseudogulbenkiania ferrooxidans	
B9Z4P0	methylglutaconyl-CoA hydratase 2	IX	Pseudogulbenkiania ferrooxidans	
W1MKV0	methylglutaconyl-CoA hydratase 2	IX	Pseudomonas aeruginosa	
Q1I890	methylglutaconyl-CoA hydratase 2	IX	Pseudomonas entomophila	4.2.1.17
C3JYF3	methylglutaconyl-CoA hydratase 2	IX	Pseudomonas fluorescens	
Q3KA08	methylglutaconyl-CoA hydratase 2	IX	Pseudomonas fluorescens	
Q4K9P5	methylglutaconyl-CoA hydratase 2	IX	Pseudomonas fluorescens	4.2.1.8
A4XTX6	methylglutaconyl-CoA hydratase 2	IX	Pseudomonas mendocina	
Q48IG3	methylglutaconyl-CoA hydratase 2	IX	Pseudomonas savastanoi	4.2.1.17
F0ED22	methylglutaconyl-CoA hydratase 2	IX	Pseudomonas sp.	
A4VPF0	methylglutaconyl-CoA hydratase 2	IX	Pseudomonas stutzeri	
A0A099SVS0	methylglutaconyl-CoA hydratase 2	IX	Pseudomonas syringae	
Q4ZTL5	methylglutaconyl-CoA hydratase 2	IX	Pseudomonas syringae	
B6R9C2	methylglutaconyl-CoA hydratase 2	IX	Pseudovibrio sp.	
E6WTI9	methylglutaconyl-CoA hydratase 2	IX	Pseudoxanthomonas suwonensis	
Q4FUX8	methylglutaconyl-CoA hydratase 2	IX	Psychrobacter arcticus	4.2.1.18
A5WH65	methylglutaconyl-CoA hydratase 2	IX	Psychrobacter sp.	
B2UDA2	methylglutaconyl-CoA hydratase 2	IX	Ralstonia pickettii	
D8NQS3	methylglutaconyl-CoA hydratase 2	IX	Ralstonia solanacearum	4.2.1.17
A4B8T8	methylglutaconyl-CoA hydratase 2	IX	Reinekea blandensis	
A4BDR7	methylglutaconyl-CoA hydratase 2	IX	Reinekea blandensis	
Q92VJ6	methylglutaconyl-CoA hydratase 2	IX	Rhizobium meliloti	4.2.1.17
A0A061MM15	methylglutaconyl-CoA hydratase 2	IX	Rhizobium rhizogenes	
C3KRB0	methylglutaconyl-CoA hydratase 2	IX	Rhizobium sp.	
D5ATH2	methylglutaconyl-CoA hydratase 2	IX	Rhodobacter capsulatus	4.1.3.36
A3PIW7	methylglutaconyl-CoA hydratase 2	IX	Rhodobacter sphaeroides	4.2.1.18
A4WU35	methylglutaconyl-CoA hydratase 2	IX	Rhodobacter sphaeroides	4.2.1.18
B6B465	methylglutaconyl-CoA hydratase 2	IX	Rhodobacteraceae bacterium	
B9NNG9	methylglutaconyl-CoA hydratase 2	IX	Rhodobacteraceae bacterium	
A3JUI9	methylglutaconyl-CoA hydratase 2	IX	Rhodobacteraceae bacterium	4.2.1.17
B6BB90	methylglutaconyl-CoA hydratase 2	IX	Rhodobacterales bacterium	
Q21RS3	methylglutaconyl-CoA hydratase 2	IX	Rhodoferax ferrireducens	
Q222S3	methylglutaconyl-CoA hydratase 2	IX	Rhodoferax ferrireducens	
B6IQT9	methylglutaconyl-CoA hydratase 2	IX	Rhodospirillum centenum	
Q2RT03	methylglutaconyl-CoA hydratase 2	IX	Rhodospirillum rubrum	4.2.1.18
E2CDS1	methylglutaconyl-CoA hydratase 2	IX	Roseibium sp.	
A7NHZ2	methylglutaconyl-CoA hydratase 2	IX	Roseiflexus castenholzii	
A5UZX6	methylglutaconyl-CoA hydratase 2	IX	Roseiflexus sp.	
F7ZCQ5	methylglutaconyl-CoA hydratase 2	IX	Roseobacter litoralis	
A4ELN1	methylglutaconyl-CoA hydratase 2	IX	Roseobacter sp.	
B7RPH6	methylglutaconyl-CoA hydratase 2	IX	Roseobacter sp.	
A3X7S0	methylglutaconyl-CoA hydratase 2	IX	Roseobacter sp.	4.2.1.17
A4EYS4	methylglutaconyl-CoA hydratase 2	IX	Roseobacter sp.	4.2.1.17
A6FQY6	methylglutaconyl-CoA hydratase 2	IX	Roseobacter sp.	4.2.1.17
D5RIV7	methylglutaconyl-CoA hydratase 2	IX	Roseomonas cervicalis	4.1.3.36
A3SNJ8	methylglutaconyl-CoA hydratase 2	IX	Roseovarius nubinhibens	4.2.1.17
A3VX19	methylglutaconyl-CoA hydratase 2	IX	Roseovarius sp.	4.2.1.17
A3W6H5	methylglutaconyl-CoA hydratase 2	IX	Roseovarius sp.	4.2.1.17
A6E068	methylglutaconyl-CoA hydratase 2	IX	Roseovarius sp.	4.2.1.17
D0CTK8	methylglutaconyl-CoA hydratase 2	IX	Ruegeria lacuscaerulensis	

Table S2.5. (continuation)

UniProt	Family [3]	Cluster	Species	EC Number
Q5LPR2	methylglutaconyl-CoA hydratase 2	IX	Ruegeria pomeroyi	
A3K1C0	methylglutaconyl-CoA hydratase 2	IX	Sagittula stellata	4.2.1.17
D5H985	methylglutaconyl-CoA hydratase 2	IX	Salinibacter ruber	4.2.1.17
A1S5B1	methylglutaconyl-CoA hydratase 2	IX	Shewanella amazonensis	
A9KVP2	methylglutaconyl-CoA hydratase 2	IX	Shewanella baltica	
Q12LZ9	methylglutaconyl-CoA hydratase 2	IX	Shewanella denitrificans	4.2.1.18
Q07ZJ5	methylglutaconyl-CoA hydratase 2	IX	Shewanella frigidimarina	4.2.1.18
B0TMU3	methylglutaconyl-CoA hydratase 2	IX	Shewanella halifaxensis	
A3QG40	methylglutaconyl-CoA hydratase 2	IX	Shewanella loihica	4.2.1.18
A8H6D2	methylglutaconyl-CoA hydratase 2	IX	Shewanella pealeana	
B8CRY5	methylglutaconyl-CoA hydratase 2	IX	Shewanella piezotolerans	4.2.1.17
E6XI96	methylglutaconyl-CoA hydratase 2	IX	Shewanella putrefaciens	
A8FT94	methylglutaconyl-CoA hydratase 2	IX	Shewanella sediminis	
D4ZHV3	methylglutaconyl-CoA hydratase 2	IX	Shewanella violacea	
B1KNN7	methylglutaconyl-CoA hydratase 2	IX	Shewanella woodyi	
C9D2P9	methylglutaconyl-CoA hydratase 2	IX	Silicibacter sp.	
A6UI57	methylglutaconyl-CoA hydratase 2	IX	Sinorhizobium medicae	
D1CAY0	methylglutaconyl-CoA hydratase 2	IX	Sphaerobacter thermophilus	
J7SX47	methylglutaconyl-CoA hydratase 2	IX	Stenotrophomonas maltophilia	
A0A0C3MLR2	methylglutaconyl-CoA hydratase 2	IX	Thauera sp.	
C4ZP69	methylglutaconyl-CoA hydratase 2	IX	Thauera sp.	
D6CMT8	methylglutaconyl-CoA hydratase 2	IX	Thiomonas arsenitoxydans	4.2.1.17
D6CRG8	methylglutaconyl-CoA hydratase 2	IX	Thiomonas arsenitoxydans	4.2.1.17
D5WZE6	methylglutaconyl-CoA hydratase 2	IX	Thiomonas intermedia	
C5JAS5	methylglutaconyl-CoA hydratase 2	IX	uncultured bacterium	
Q1EHZ7	methylglutaconyl-CoA hydratase 2	IX	uncultured organism	
E6V7E4	methylglutaconyl-CoA hydratase 2	IX	Variovorax paradoxus	
A1WHK6	methylglutaconyl-CoA hydratase 2	IX	Verminephrobacter eiseniae	
D0X396	methylglutaconyl-CoA hydratase 2	IX	Vibrio alginolyticus	
A7JYA8	methylglutaconyl-CoA hydratase 2	IX	Vibrio antiquarius	4.2.1.18
E8LZZ5	methylglutaconyl-CoA hydratase 2	IX	Vibrio brasiliensis	
A0A0A3ESQ2	methylglutaconyl-CoA hydratase 2	IX	Vibrio campbellii	
E3BMF7	methylglutaconyl-CoA hydratase 2	IX	Vibrio caribbeanicus	
F0M0J0	methylglutaconyl-CoA hydratase 2	IX	Vibrio furnissii	
C9QFR7	methylglutaconyl-CoA hydratase 2	IX	Vibrio orientalis	
A6AZ73	methylglutaconyl-CoA hydratase 2	IX	Vibrio parahaemolyticus	
A6BAJ7	methylglutaconyl-CoA hydratase 2	IX	Vibrio parahaemolyticus	
A6D641	methylglutaconyl-CoA hydratase 2	IX	Vibrio shilonii	
E8M593	methylglutaconyl-CoA hydratase 2	IX	Vibrio sinaloensis	
A8T041	methylglutaconyl-CoA hydratase 2	IX	Vibrio sp.	
B8K6R4	methylglutaconyl-CoA hydratase 2	IX	Vibrio sp.	
Q7MDI8	methylglutaconyl-CoA hydratase 2	IX	Vibrio vulnificus	
A7IGF8	methylglutaconyl-CoA hydratase 2	IX	Xanthobacter autotrophicus	
D2UAC3	methylglutaconyl-CoA hydratase 2	IX	Xanthomonas albilineans	4.2.1.17
B0RSN8	methylglutaconyl-CoA hydratase 2	IX	Xanthomonas campestris	4.2.1.17
Q3BUD3	methylglutaconyl-CoA hydratase 2	IX	Xanthomonas campestris	4.2.1.17
F0C8H6	methylglutaconyl-CoA hydratase 2	IX	Xanthomonas gardneri	
F0BF16	methylglutaconyl-CoA hydratase 2	IX	Xanthomonas vesicatoria	
A0A0B6HV44	methylmalonyl-CoA decarboxylase	I		
C7RN30	methylmalonyl-CoA decarboxylase	I	Accumulibacter phosphatis	
A2VS95	methylmalonyl-CoA decarboxylase	I	Burkholderia cenocepacia	
Q13SD9	methylmalonyl-CoA decarboxylase	I	Burkholderia xenovorans	
A0A064DQ91	methylmalonyl-CoA decarboxylase	I	Citrobacter freundii	
A0A0A1RU22	methylmalonyl-CoA decarboxylase	I	Citrobacter pasteurii	4.1.1.41
D8EVY3	methylmalonyl-CoA decarboxylase	I	delta proteobacterium	
E3G494	methylmalonyl-CoA decarboxylase	I	Enterobacter lignolyticus	

Table S2.5. (continuation)

UniProt	Family [3]	Cluster	Species	EC Number
B1EFY3	methylmalonyl-CoA decarboxylase	I	Escherichia albertii	4.1.1.41
A0A024KPQ1	methylmalonyl-CoA decarboxylase	I	Escherichia coli	
A0A0E2A4R1	methylmalonyl-CoA decarboxylase	I	Escherichia coli	
B3HGR3	methylmalonyl-CoA decarboxylase	I	Escherichia coli	
B6I751	methylmalonyl-CoA decarboxylase	I	Escherichia coli	
E9TJU6	methylmalonyl-CoA decarboxylase	I	Escherichia coli	
N2JG47	methylmalonyl-CoA decarboxylase	I	Escherichia coli	
P52045	methylmalonyl-CoA decarboxylase	I	Escherichia coli	4.1.1.41
B3E862	methylmalonyl-CoA decarboxylase	I	Geobacter lovleyi	
E8WPY7	methylmalonyl-CoA decarboxylase	I	Geobacter sp.	
A5G7C1	methylmalonyl-CoA decarboxylase	I	Geobacter uraniireducens	
B1Y5L4	methylmalonyl-CoA decarboxylase	I	Leptothrix cholodnii	
Q2W529	methylmalonyl-CoA decarboxylase	I	Magnetospirillum magneticum	
A0A0C2UB39	methylmalonyl-CoA decarboxylase	I	Magnetospirillum magnetot.	
A4SZB1	methylmalonyl-CoA decarboxylase	I	Polynucleobacter necessarius	
E3I3S5	methylmalonyl-CoA decarboxylase	I	Rhodomicrobium vannielii	
A9MRF8	methylmalonyl-CoA decarboxylase	I	Salmonella arizonae	
H7EBW7	methylmalonyl-CoA decarboxylase	I	Salmonella enterica	
B3WX57	methylmalonyl-CoA decarboxylase	I	Shigella dysenteriae	4.1.1.41
I6GT65	methylmalonyl-CoA decarboxylase	I	Shigella flexneri	
A0A0E1NFX4	methylmalonyl-CoA decarboxylase	I	Yersinia enterocolitica	4.1.1.41
D3F027	methylmalonyl-CoA decarboxylase	X	Conexibacter woesei	
F1TD52	polyketide biosynth. enoyl-CoA hydratase	IV	[Clostridium] papyrosolvens	
E1UUM8	polyketide biosynth. enoyl-CoA hydratase	IV	Bacillus amyloliquefaciens	
Q1RS75	polyketide biosynth. enoyl-CoA hydratase	IV	Bacillus amyloliquefaciens	4.2.1.17
A0A080UJX7	polyketide biosynth. enoyl-CoA hydratase	IV	Bacillus subtilis	
P40805	polyketide biosynth. enoyl-CoA hydratase	IV	Bacillus subtilis	4.2.1.-
A7A5I1	polyketide biosynth. enoyl-CoA hydratase	IV	Bifidobacterium adolescentis	
F2LA04	polyketide biosynth. enoyl-CoA hydratase	IV	Burkholderia gladioli	
F2LK75	polyketide biosynth. enoyl-CoA hydratase	IV	Burkholderia gladioli	
A0A069B8Y4	polyketide biosynth. enoyl-CoA hydratase	IV	Burkholderia pseudomallei	
A0A0F5K5U9	polyketide biosynth. enoyl-CoA hydratase	IV	Burkholderia pseudomallei	
A3NJE5	polyketide biosynth. enoyl-CoA hydratase	IV	Burkholderia pseudomallei	
Q3JF91	polyketide biosynth. enoyl-CoA hydratase	IV	Burkholderia pseudomallei	4.2.1.17
A0A075E6J0	polyketide biosynth. enoyl-CoA hydratase	IV	Burkholderia sp.	
Q2T4N6	polyketide biosynth. enoyl-CoA hydratase	IV	Burkholderia thailandensis	
C7PSG1	polyketide biosynth. enoyl-CoA hydratase	IV	Chitinophaga pinensis	
F5B9D3	polyketide biosynth. enoyl-CoA hydratase	IV	Chitinophaga sancti	
B8HKZ4	polyketide biosynth. enoyl-CoA hydratase	IV	Cyanothece sp.	
C6CDV2	polyketide biosynth. enoyl-CoA hydratase	IV	Dickeya dadantii	
A9EAC8	polyketide biosynth. enoyl-CoA hydratase	IV	Kordia algicida	
Q6DNE8	polyketide biosynth. enoyl-CoA hydratase	IV	Lyngbya majuscula	
A4TWX5	polyketide biosynth. enoyl-CoA hydratase	IV	Magnetospirillum gryph.	
Q1D5F3	polyketide biosynth. enoyl-CoA hydratase	IV	Myxococcus xanthus	
D2JNW4	polyketide biosynth. enoyl-CoA hydratase	IV	Nostoc sp.	
E0RFQ7	polyketide biosynth. enoyl-CoA hydratase	IV	Paenibacillus polymyxa	
E3E978	polyketide biosynth. enoyl-CoA hydratase	IV	Paenibacillus polymyxa	
I8TTA6	polyketide biosynth. enoyl-CoA hydratase	IV	Pelosinus fermentans	
I6Y3T6	polyketide biosynth. enoyl-CoA hydratase	IV	Propionibacterium propionicum	
F8J3G4	polyketide biosynth. enoyl-CoA hydratase	IV	Pseudoalteromonas sp.	
D4NZD9	polyketide biosynth. enoyl-CoA hydratase	IV	Pseudomonas fluorescens	
Q8RL62	polyketide biosynth. enoyl-CoA hydratase	IV	Pseudomonas fluorescens	
A9GJ07	polyketide biosynth. enoyl-CoA hydratase	IV	Sorangium cellulosum	4.2.1.17
D9XQB6	polyketide biosynth. enoyl-CoA hydratase	IV	Streptomyces griseoflavus	
Q5I691	polyketide biosynth. enoyl-CoA hydratase	IV	symbiont bacterium	
Q49HJ6	polyketide biosynth. enoyl-CoA hydratase	IV	uncultured bacterial	

Table S2.5. (continuation)

UniProt	Family [3]	Cluster	Species	EC Number
D2SUE7	polyketide biosynth. enoyl-CoA hydratase	IV	uncultured bacterium	
C7PXR1	polyketide biosynth. enoyl-CoA hydratase	X	Catenulispora acidiphila	
D7RK28	polyketide biosynth. enoyl-CoA hydratase	X	Corallococcus coralloides	
D9T195	polyketide biosynth. enoyl-CoA hydratase	X	Micromonospora aurantiaca	
B4VFF4	polyketide biosynth. enoyl-CoA hydratase	X	Streptomyces sp.	
B5GAC2	polyketide biosynth. enoyl-CoA hydratase	IX	Streptomyces sp.	

Case study VI: enolases

After the filtering process, 4,791 proteins remained in this SFLD [3] superfamily, distributed among six subgroups and twelve families. The protein set, along with the SFLD family labels and the clusters they were placed in by the proposed framework when considering twelve clusters, as well as the source organisms and existing EC number annotations according to UniProt, are presented in Table S2.6.

Table S2.6. List of proteins in the enolase superfamily.

UniProt	Family [3]	Cluster	Species	EC Number
D7HYG3	D-galactonate dehydratase	XI		
V4PI73	D-galactonate dehydratase	XI	Asticcacaulis sp.	
Q0BC48	D-galactonate dehydratase	XI	Burkholderia ambifaria	4.2.1.6
Q6J674	D-galactonate dehydratase	XI	Collimonas fungivorans	
A8A6F2	D-galactonate dehydratase	XI	Escherichia coli	4.2.1.6
B1LPS5	D-galactonate dehydratase	XI	Escherichia coli	4.2.1.6
Q1R4P4	D-galactonate dehydratase	XI	Escherichia coli	4.2.1.6
M5ECV1	D-galactonate dehydratase	XI	Halanaerobium sacc.	4.2.1.39
A0A0B7G3I6	D-galactonate dehydratase	XI	Klebsiella variicola	4.2.1.6
F7Y0E3	D-galactonate dehydratase	XI	Mesorhizobium opportunistum	
A1D478	D-galactonate dehydratase	XI	Neosartorya fischeri	
M4S1M0	D-galactonate dehydratase	XI	Sphingomonas sp.	
Q47NZ8	D-galactonate dehydratase	XI	Thermobifida fusca	4.2.1.6
D4DHR6	D-galactonate dehydratase	XI	Trichophyton verrucosum	
A0A077Z5W4	D-galactonate dehydratase	XI	Trichuris trichiura	
V4J1P4	D-galactonate dehydratase	XI	uncultured Desulfofustis	
A6CZS0	D-galactonate dehydratase	XI	Vibrio shilonii	
Q123J3	D-galactonate dehydratase	XII	Polaromonas sp.	4.2.1.6
C0CXB1	D-galactonate dehydratase	X	[Clostridium asparagiforme]	
C0D3V1	D-galactonate dehydratase	X	[Clostridium asparagiforme]	
C0C5R8	D-galactonate dehydratase	X	[Clostridium] hylemonae	
A7VPV9	D-galactonate dehydratase	X	[Clostridium] leptum	
A0A052IM78	D-galactonate dehydratase	X		
A0A069HQ59	D-galactonate dehydratase	X		
A0A096Z5Z0	D-galactonate dehydratase	X		
E7P3W5	D-galactonate dehydratase	X		
J4Z413	D-galactonate dehydratase	X	Achromobacter piechaudii	
A1TPD4	D-galactonate dehydratase	X	Acidovorax citrulli	4.2.1.6
J0U2A8	D-galactonate dehydratase	X	Acidovorax sp.	
A0A086T984	D-galactonate dehydratase	X	Acremonium chrysogenum	
A0A099D2S4	D-galactonate dehydratase	X	Actinopolyspora erythraea	
K5X2M6	D-galactonate dehydratase	X	Agaricus bisporus	
K9I346	D-galactonate dehydratase	X	Agaricus bisporus	
A0A067TZ45	D-galactonate dehydratase	X	Agrobacterium tumefaciens	
M8AQK7	D-galactonate dehydratase	X	Agrobacterium tumefaciens	
F0U9A6	D-galactonate dehydratase	X	Ajellomyces capsulatus	
A0A0A5JAM4	D-galactonate dehydratase	X	Alcaligenes xylosoxydans	
J9HBB0	D-galactonate dehydratase	X	Alicyclobacillus hesperidum	
A0A0C2TBL2	D-galactonate dehydratase	X	Amanita muscaria	
D5EGV4	D-galactonate dehydratase	X	Aminobacterium colombiense	

Table S2.6. (continuation)

UniProt	Family [3]	Cluster	Species	EC Number
A0A066U8L4	D-galactonate dehydratase	X	Amycolatopsis rifamycinica	
A0A094LUX7	D-galactonate dehydratase	X	Amycolatopsis sp.	
E1W0G9	D-galactonate dehydratase	X	Arthrobacter arilaitensis	4.2.1.6
G1XMJ8	D-galactonate dehydratase	X	Arthrobotrys oligospora	
D4AN19	D-galactonate dehydratase	X	Arthroderma benhamiae	
A1CRB6	D-galactonate dehydratase	X	Aspergillus clavatus	
A0A084BS49	D-galactonate dehydratase	X	Aspergillus fumigatus	
G7X7H3	D-galactonate dehydratase	X	Aspergillus kawachii	
G7X955	D-galactonate dehydratase	X	Aspergillus kawachii	
A2QI98	D-galactonate dehydratase	X	Aspergillus niger	
G3Y8T6	D-galactonate dehydratase	X	Aspergillus niger	
A2RAU0	D-galactonate dehydratase	X	Aspergillus niger	5.5.-.-
I7ZN59	D-galactonate dehydratase	X	Aspergillus oryzae	
A0A017SCI8	D-galactonate dehydratase	X	Aspergillus ruber	
Q0CTA6	D-galactonate dehydratase	X	Aspergillus terreus	
A0A0C1BWH6	D-galactonate dehydratase	X	Aspergillus ustus	
V4P2C1	D-galactonate dehydratase	X	Asticcacaulis benevestitus	
V4PFB6	D-galactonate dehydratase	X	Asticcacaulis benevestitus	
E8RVF8	D-galactonate dehydratase	X	Asticcacaulis excentricus	
V4NKR2	D-galactonate dehydratase	X	Asticcacaulis sp.	
V4P1N6	D-galactonate dehydratase	X	Asticcacaulis sp.	
V4P7M4	D-galactonate dehydratase	X	Asticcacaulis sp.	
V4PNC9	D-galactonate dehydratase	X	Asticcacaulis sp.	
V4QCD8	D-galactonate dehydratase	X	Asticcacaulis sp.	
A0A074VH64	D-galactonate dehydratase	X	Aureobasidium melanogenum	
A0A074VZL9	D-galactonate dehydratase	X	Aureobasidium melanogenum	
A0A074WN72	D-galactonate dehydratase	X	Aureobasidium namibiae	
A0A074WV59	D-galactonate dehydratase	X	Aureobasidium namibiae	
A0A074XFJ4	D-galactonate dehydratase	X	Aureobasidium pullulans	
A0A074YGM7	D-galactonate dehydratase	X	Aureobasidium subglaciale	
A0A074YJI6	D-galactonate dehydratase	X	Aureobasidium subglaciale	
A0A0C4WU69	D-galactonate dehydratase	X	Azotobacter chroococcum	
C1DMD4	D-galactonate dehydratase	X	Azotobacter vinelandii	
W4QP97	D-galactonate dehydratase	X	Bacillus akibai	
K6DL86	D-galactonate dehydratase	X	Bacillus bataviensis	
Q5WKP1	D-galactonate dehydratase	X	Bacillus clausii	4.2.1.6
A0A0A6VAR3	D-galactonate dehydratase	X	Bacillus ginsengihumi	
A0A060M179	D-galactonate dehydratase	X	Bacillus lehensis	
A0A0B0ICW4	D-galactonate dehydratase	X	Bacillus okhensis	
A0A0B0IIS2	D-galactonate dehydratase	X	Bacillus okhensis	
M5PC86	D-galactonate dehydratase	X	Bacillus sonorensis	
E5WFX7	D-galactonate dehydratase	X	Bacillus sp.	
W7YYQ3	D-galactonate dehydratase	X	Bacillus sp.	
M2NN60	D-galactonate dehydratase	X	Baudoinia compniacensis	
W6YFF6	D-galactonate dehydratase	X	Bipolaris zeicola	
A0A067MUY6	D-galactonate dehydratase	X	Botryobasidium botryosum	
R1GFP1	D-galactonate dehydratase	X	Botryosphaeria parva	
M7TQB9	D-galactonate dehydratase	X	Botryotinia fuckeliana	
A0A0B9AXE8	D-galactonate dehydratase	X	Brevibacterium linens	4.2.1.6
A0A081UZS1	D-galactonate dehydratase	X	Burkholderia cepacia	
A2W7N5	D-galactonate dehydratase	X	Burkholderia dolosa	
A0A095W8Q4	D-galactonate dehydratase	X	Burkholderia gladioli	
A0A069PTJ9	D-galactonate dehydratase	X	Burkholderia glathei	4.2.1.39
Q39NI3	D-galactonate dehydratase	X	Burkholderia lata	4.2.1.6
K0DUT9	D-galactonate dehydratase	X	Burkholderia phenoliruptrix	
A0A069B558	D-galactonate dehydratase	X	Burkholderia pseudomallei	

Table S2.6. (continuation)

UniProt	Family [3]	Cluster	Species	EC Number
A0A0A3JN17	D-galactonate dehydratase	X	Burkholderia pseudomallei	
B1HFK9	D-galactonate dehydratase	X	Burkholderia pseudomallei	
A0A0A6Q7J4	D-galactonate dehydratase	X	Burkholderia sacchari	
A0A084D638	D-galactonate dehydratase	X	Burkholderia sp.	
A0A0D0HDZ2	D-galactonate dehydratase	X	Burkholderia sp.	
A0A0C1Z020	D-galactonate dehydratase	X	Burkholderia sp.	4.2.1.6
A0A096YJI3	D-galactonate dehydratase	X	Burkholderia thailandensis	
A0A085FZJ0	D-galactonate dehydratase	X	Buttiauxella agrestis	4.2.1.6
K9GV45	D-galactonate dehydratase	X	Caenispirillum salinarum	
V4HAK1	D-galactonate dehydratase	X	Candidatus Halobonum	
W9XIV3	D-galactonate dehydratase	X	Capronia coronata	
W9YJA1	D-galactonate dehydratase	X	Capronia epimyces	
A0A0D2GBG0	D-galactonate dehydratase	X	Capronia semiimmersa	
M2PHP9	D-galactonate dehydratase	X	Ceriporiopsis subvermispora	
V9DEK6	D-galactonate dehydratase	X	Cladophialophora carrionii	
A0A0D2AT46	D-galactonate dehydratase	X	Cladophialophora immunda	
W9X8J6	D-galactonate dehydratase	X	Cladophialophora psammophila	
W9W4G6	D-galactonate dehydratase	X	Cladophialophora yegresii	
W9WLI3	D-galactonate dehydratase	X	Cladophialophora yegresii	
U2CMQ3	D-galactonate dehydratase	X	Clostridiales bacterium	
C5EHK3	D-galactonate dehydratase	X	Clostridiales bacterium	4.2.1.6
R4K1U3	D-galactonate dehydratase	X	Clostridium pasteurianum	
R9BUD2	D-galactonate dehydratase	X	Clostridium sartagoforme	
U2CWE9	D-galactonate dehydratase	X	Clostridium sp.	
A0A0F0CM90	D-galactonate dehydratase	X	Clostridium sp.	4.2.1.6
G7M728	D-galactonate dehydratase	X	Clostridium sp.	4.2.1.6
M2T3D3	D-galactonate dehydratase	X	Cochliobolus sativus	
A0A010QW09	D-galactonate dehydratase	X	Colletotrichum fioriniae	
A0A010R963	D-galactonate dehydratase	X	Colletotrichum fioriniae	
L2FWC3	D-galactonate dehydratase	X	Colletotrichum gloeosporioides	
T0LX62	D-galactonate dehydratase	X	Colletotrichum gloeosporioides	
E3QUP8	D-galactonate dehydratase	X	Colletotrichum graminicola	
N4UKG1	D-galactonate dehydratase	X	Colletotrichum orbiculare	
N4VDJ0	D-galactonate dehydratase	X	Colletotrichum orbiculare	
A0A066X4E0	D-galactonate dehydratase	X	Colletotrichum sublineola	
A0A066XKC1	D-galactonate dehydratase	X	Colletotrichum sublineola	
A0A066XR36	D-galactonate dehydratase	X	Colletotrichum sublineola	
A0A0A1FJ19	D-galactonate dehydratase	X	Collimonas arenae	4.2.1.6
R7YS03	D-galactonate dehydratase	X	Coniosporium apollinis	
R7Z5D8	D-galactonate dehydratase	X	Coniosporium apollinis	
G3JFA4	D-galactonate dehydratase	X	Cordyceps militaris	
A7MN07	D-galactonate dehydratase	X	Cronobacter sakazakii	4.2.1.6
A0A0D2JGK0	D-galactonate dehydratase	X	Cryptococcus gattii	
F5H8S2	D-galactonate dehydratase	X	Cryptococcus neoformans	
W2RIR1	D-galactonate dehydratase	X	Cyphellophora europaea	
M5GAA5	D-galactonate dehydratase	X	Dacryopinax sp.	
S8AEG3	D-galactonate dehydratase	X	Dactylellina haptotyla	
C1D2H9	D-galactonate dehydratase	X	Deinococcus deserti	
Q1J2P2	D-galactonate dehydratase	X	Deinococcus geothermalis	
L0A8T0	D-galactonate dehydratase	X	Deinococcus peraridilitoris	
A0A0A7KIX3	D-galactonate dehydratase	X	Deinococcus swuensis	
R7SZZ1	D-galactonate dehydratase	X	Dichomitus squalens	
N1PX21	D-galactonate dehydratase	X	Dothistroma septosporum	
C6W0B6	D-galactonate dehydratase	X	Dyadobacter fermentans	
A0A075JWZ2	D-galactonate dehydratase	X	Dyella japonica	
A0A023NWF3	D-galactonate dehydratase	X	Dyella jiangningensis	

Table S2.6. (continuation)

UniProt	Family [3]	Cluster	Species	EC Number
L0G4P1	D-galactonate dehydratase	X	Echinicola vietnamensis	
W8I6T5	D-galactonate dehydratase	X	Ensifer adhaerens	
A0A0C5W5B8	D-galactonate dehydratase	X	Enterobacteriaceae bacterium	4.2.1.6
S1RL54	D-galactonate dehydratase	X	Enterococcus cecorum	
R2VES3	D-galactonate dehydratase	X	Enterococcus gilvus	
R2NMB1	D-galactonate dehydratase	X	Enterococcus malodoratus	
R2P088	D-galactonate dehydratase	X	Enterococcus malodoratus	
A0A070K8S6	D-galactonate dehydratase	X	Escherichia coli	4.2.1.6
B1LL17	D-galactonate dehydratase	X	Escherichia coli	4.2.1.6
B7MGB3	D-galactonate dehydratase	X	Escherichia coli	4.2.1.6
E1HSA1	D-galactonate dehydratase	X	Escherichia coli	4.2.1.6
E9XS92	D-galactonate dehydratase	X	Escherichia coli	4.2.1.6
S1HPS9	D-galactonate dehydratase	X	Escherichia coli	4.2.1.6
V6FPK7	D-galactonate dehydratase	X	Escherichia coli	4.2.1.6
M7TWV2	D-galactonate dehydratase	X	Eutypa lata	
A0A085G4L3	D-galactonate dehydratase	X	Ewingella americana	4.2.1.6
A0A072PSZ7	D-galactonate dehydratase	X	Exophiala aquamarina	
H6C429	D-galactonate dehydratase	X	Exophiala dermatitidis	
A0A0D1ZPY8	D-galactonate dehydratase	X	Exophiala mesophila	
A0A0D1X2R7	D-galactonate dehydratase	X	Exophiala sideris	
A0A0D1YJ43	D-galactonate dehydratase	X	Exophiala spinifera	
A0A0D2EZN4	D-galactonate dehydratase	X	Exophiala xenobiotica	
S8EAV9	D-galactonate dehydratase	X	Fomitopsis pinicola	
A0A0D2EKK0	D-galactonate dehydratase	X	Fonsecaea pedrosoi	
W9MPV8	D-galactonate dehydratase	X	Fusarium oxysporum	
W9N6V8	D-galactonate dehydratase	X	Fusarium oxysporum	
X0JMA5	D-galactonate dehydratase	X	Fusarium oxysporum	
K3VJE9	D-galactonate dehydratase	X	Fusarium pseudograminearum	
V9HQS8	D-galactonate dehydratase	X	Fusobacterium ulcerans	
J3NWJ1	D-galactonate dehydratase	X	Gaeumannomyces graminis	
A0A067TCY2	D-galactonate dehydratase	X	Galerina marginata	
A0A0A2YKT4	D-galactonate dehydratase	X	Gallibacterium anatis	
A0A0A2XX35	D-galactonate dehydratase	X	Gallibacterium genomosp.	
Q1YSG0	D-galactonate dehydratase	X	gamma proteobacterium	
A0A086D1J3	D-galactonate dehydratase	X	Gammaproteobacteria bacterium	
W0RNE5	D-galactonate dehydratase	X	Gemmatirosa kalamazoonesis	
Q5KYJ6	D-galactonate dehydratase	X	Geobacillus kaustophilus	4.2.1.6
S5ZP91	D-galactonate dehydratase	X	Geobacillus sp.	
A0A061N048	D-galactonate dehydratase	X	Geomicrobium sp.	
W7MTJ0	D-galactonate dehydratase	X	Gibberella moniliformis	
A0A016Q220	D-galactonate dehydratase	X	Gibberella zeae	
S3DWS2	D-galactonate dehydratase	X	Glarea lozoyensis	
S7PY50	D-galactonate dehydratase	X	Gloeophyllum trabeum	
A9HL32	D-galactonate dehydratase	X	Gluconacetobacter diazotrophicus	
B5ZCC2	D-galactonate dehydratase	X	Gluconacetobacter diazotrophicus	
F0XT56	D-galactonate dehydratase	X	Grosmannia clavigera	
A0A085H083	D-galactonate dehydratase	X	Hafnia alvei	4.2.1.6
A0A097QX73	D-galactonate dehydratase	X	Hafnia alvei	4.2.1.6
A0A0B9AH36	D-galactonate dehydratase	X	Hafnia paralvei	4.2.1.6
E7QVR7	D-galactonate dehydratase	X	Haladaptatus paucihalophilus	
D8J3L2	D-galactonate dehydratase	X	Halalkalicoccus jeotgali	
U2YW61	D-galactonate dehydratase	X	Halarchaeum acidiphilum	
G0HSC4	D-galactonate dehydratase	X	Haloarcula hispanica	4.2.1.6
Q5V6U9	D-galactonate dehydratase	X	Haloarcula marismortui	
M0JS81	D-galactonate dehydratase	X	Haloarcula sinaiiensis	
A0A0B5GZU1	D-galactonate dehydratase	X	Haloarcula sp.	

Table S2.6. (continuation)

UniProt	Family [3]	Cluster	Species	EC Number
M0IUZ9	D-galactonate dehydratase	X	Haloarcula vallismortis	
I0JIA3	D-galactonate dehydratase	X	Halobacillus halophilus	
L5N836	D-galactonate dehydratase	X	Halobacillus sp.	
L0KEA8	D-galactonate dehydratase	X	Halobacteroides halobius	
M0M6X8	D-galactonate dehydratase	X	Halococcus hamelinensis	
M0MI90	D-galactonate dehydratase	X	Halococcus morrhuae	
M0ML52	D-galactonate dehydratase	X	Halococcus saccharolyticus	
M0MY57	D-galactonate dehydratase	X	Halococcus salifodinae	
M0MVB4	D-galactonate dehydratase	X	Halococcus thailandensis	
M0JJT4	D-galactonate dehydratase	X	Haloferax denitrificans	
M0FM41	D-galactonate dehydratase	X	Haloferax sp.	
D4GR08	D-galactonate dehydratase	X	Haloferax volcanii	
M0CZ78	D-galactonate dehydratase	X	Halogeometricum pallidum	
J2Z8U3	D-galactonate dehydratase	X	Halogranum salarium	
J3EUG5	D-galactonate dehydratase	X	Halogranum salarium	
C7NWB9	D-galactonate dehydratase	X	Halomicrobium mukohataei	
W1N2D4	D-galactonate dehydratase	X	Halomonas huangheensis	
A0A098RE81	D-galactonate dehydratase	X	Halomonas salina	
A0A0C3II46	D-galactonate dehydratase	X	Halomonas sp.	
A0A0F4QQN5	D-galactonate dehydratase	X	Halomonas sp.	
F8DAX6	D-galactonate dehydratase	X	Halopiger xanaduensis	4.2.1.6
M0PL86	D-galactonate dehydratase	X	Halorubrum aidingense	
M0PNA2	D-galactonate dehydratase	X	Halorubrum arcis	
M0EM15	D-galactonate dehydratase	X	Halorubrum californiensis	
M0FJ97	D-galactonate dehydratase	X	Halorubrum hochstenium	
M0P451	D-galactonate dehydratase	X	Halorubrum kocurii	
M0NLI8	D-galactonate dehydratase	X	Halorubrum lipolyticum	
M0NNT0	D-galactonate dehydratase	X	Halorubrum lipolyticum	
M0DCX9	D-galactonate dehydratase	X	Halorubrum tebenquichense	
W0JUF8	D-galactonate dehydratase	X	Halostagnicola larsenii	
W0JWE1	D-galactonate dehydratase	X	Halostagnicola larsenii	
D2S241	D-galactonate dehydratase	X	Haloterrigena turkmenica	
R0EB91	D-galactonate dehydratase	X	Herbaspirillum frisingense	
I3CXL2	D-galactonate dehydratase	X	Herbaspirillum sp.	
J2W582	D-galactonate dehydratase	X	Herbaspirillum sp.	
J3DIN3	D-galactonate dehydratase	X	Herbaspirillum sp.	
W4JWH0	D-galactonate dehydratase	X	Heterobasidion irregulare	
D3AH14	D-galactonate dehydratase	X	Hungatella hathewayi	
A0A016XFM0	D-galactonate dehydratase	X	Hylemonella gracilis	
F3KW25	D-galactonate dehydratase	X	Hylemonella gracilis	
A0A0A7LSP0	D-galactonate dehydratase	X	Hymenobacter sp.	
G9NPQ5	D-galactonate dehydratase	X	Hypocrea atroviridis	
G0RQ45	D-galactonate dehydratase	X	Hypocrea jecorina	
G9N822	D-galactonate dehydratase	X	Hypocrea virens	
W9GM18	D-galactonate dehydratase	X	Intrasporangium chromatireducens	
L9PHY5	D-galactonate dehydratase	X	Janthinobacterium sp.	4.2.1.6
A0A0D5X0H9	D-galactonate dehydratase	X	Klebsiella michiganensis	4.2.1.6
A6TFZ4	D-galactonate dehydratase	X	Klebsiella pneumoniae	4.2.1.6
B5XW18	D-galactonate dehydratase	X	Klebsiella pneumoniae	4.2.1.6
Z5DJU6	D-galactonate dehydratase	X	Klebsiella pneumoniae	4.2.1.6
W7SQF2	D-galactonate dehydratase	X	Kutzneria sp.	
A0NSC3	D-galactonate dehydratase	X	Labrenzia aggregata	
U7GEI5	D-galactonate dehydratase	X	Labrenzia sp.	
F7K741	D-galactonate dehydratase	X	Lachnospiraceae bacterium	
F0THM8	D-galactonate dehydratase	X	Lactobacillus acidophilus	
S6BVE5	D-galactonate dehydratase	X	Lactobacillus casei	

Table S2.6. (continuation)

UniProt	Family [3]	Cluster	Species	EC Number
A0A0F4LJA4	D-galactonate dehydratase	X	Lactobacillus kimbladii	
A0A0F4LHU1	D-galactonate dehydratase	X	Lactobacillus melliventris	
A0A066P5F2	D-galactonate dehydratase	X	Lelliottia amnigena	4.2.1.6
E4ZWL5	D-galactonate dehydratase	X	Leptosphaeria maculans	
A0A0F3KT63	D-galactonate dehydratase	X	Luteibacter yeojuensis	4.2.1.6
A0A0A3J262	D-galactonate dehydratase	X	Lysinibacillus massiliensis	
A0A0A3HZC4	D-galactonate dehydratase	X	Lysinibacillus sinduriensis	
A0A021W7L4	D-galactonate dehydratase	X	Lysobacter capsici	
K2SKG8	D-galactonate dehydratase	X	Macrophomina phaseolina	
G4N2H1	D-galactonate dehydratase	X	Magnaporthe oryzae	
F4A3D4	D-galactonate dehydratase	X	Mahella australiensis	4.2.1.6
A4AM04	D-galactonate dehydratase	X	Maribacter sp.	
K1Y1H6	D-galactonate dehydratase	X	Marssonina brunnea	
A0A0D5LLR6	D-galactonate dehydratase	X	Martelella endophytica	
A0A0D5LMJ9	D-galactonate dehydratase	X	Martelella endophytica	
A0A098UF91	D-galactonate dehydratase	X	Massilia sp.	4.2.1.6
E8T9A6	D-galactonate dehydratase	X	Mesorhizobium ciceri	
V7G4L8	D-galactonate dehydratase	X	Mesorhizobium sp.	
X6AVP7	D-galactonate dehydratase	X	Mesorhizobium sp.	
X6GXE9	D-galactonate dehydratase	X	Mesorhizobium sp.	
X6ICB2	D-galactonate dehydratase	X	Mesorhizobium sp.	
A0A090FGU7	D-galactonate dehydratase	X	Mesorhizobium sp.	4.2.1.6
A0A0A1UVN2	D-galactonate dehydratase	X	Metarhizium robertsii	
W2EZ52	D-galactonate dehydratase	X	Microbispora sp.	
V2X817	D-galactonate dehydratase	X	Moniliophthora roreri	
A0R5F9	D-galactonate dehydratase	X	Mycobacterium smegmatis	4.2.1.6
L8F4G4	D-galactonate dehydratase	X	Mycobacterium smegmatis	4.2.1.6
L9XNX4	D-galactonate dehydratase	X	Natrinema versiforme	
L9X4V3	D-galactonate dehydratase	X	Natronococcus amylolyticus	
L9X2I2	D-galactonate dehydratase	X	Natronococcus jeotgali	
L0JZX0	D-galactonate dehydratase	X	Natronococcus occultus	4.2.1.6
C7Z2W1	D-galactonate dehydratase	X	Nectria haematococca	
Q4WJL9	D-galactonate dehydratase	X	Neosartorya fumigata	4.2.1.6
I5BYT5	D-galactonate dehydratase	X	Nitratireductor aquibiodomus	
A0A034UKP4	D-galactonate dehydratase	X	Nocardia brasiliensis	
Q8EP98	D-galactonate dehydratase	X	Oceanobacillus iheyensis	
Q6A202	D-galactonate dehydratase	X	Oenococcus phage	
A0A0C3CFG3	D-galactonate dehydratase	X	Oidiodendron maius	
A0A0C3HKL0	D-galactonate dehydratase	X	Oidiodendron maius	
A0A099D105	D-galactonate dehydratase	X	Oleiagrimonas soli	
F8FD55	D-galactonate dehydratase	X	Paenibacillus mucilaginosus	
A0A089I4T5	D-galactonate dehydratase	X	Paenibacillus sp.	
A0A0B2FE97	D-galactonate dehydratase	X	Paenibacillus sp.	
A0A0C2RDC9	D-galactonate dehydratase	X	Paenibacillus sp.	
C6D090	D-galactonate dehydratase	X	Paenibacillus sp.	
W4DN08	D-galactonate dehydratase	X	Paenibacillus sp.	
C6J2Z8	D-galactonate dehydratase	X	Paenibacillus sp.	4.2.1.6
C6J3Q7	D-galactonate dehydratase	X	Paenibacillus sp.	4.2.1.6
F3MCK4	D-galactonate dehydratase	X	Paenibacillus sp.	4.2.1.6
A0A089M2C4	D-galactonate dehydratase	X	Paenibacillus stellifer	
A0A0F5XVY9	D-galactonate dehydratase	X	Pantoea sp.	4.2.1.6
J3CZW9	D-galactonate dehydratase	X	Pantoea sp.	4.2.1.6
Q6CYT9	D-galactonate dehydratase	X	Pectobacterium atrosepticum	4.2.1.6
A0A0B8YFW5	D-galactonate dehydratase	X	Pedobacter glucosidilyticus	4.2.1.6
W6TNU6	D-galactonate dehydratase	X	Pedobacter sp.	
K9FV33	D-galactonate dehydratase	X	Penicillium digitatum	

Table S2.6. (continuation)

UniProt	Family [3]	Cluster	Species	EC Number
A0A0A2KMG4	D-galactonate dehydratase	X	Penicillium expansum	
W6QCX6	D-galactonate dehydratase	X	Penicillium roqueforti	
W3WQZ3	D-galactonate dehydratase	X	Pestalotiopsis fici	
W3WXV3	D-galactonate dehydratase	X	Pestalotiopsis fici	
A0A0C3RXV9	D-galactonate dehydratase	X	Phlebiopsis gigantea	
L8J356	D-galactonate dehydratase	X	Photobacterium marinum	
J2VQC2	D-galactonate dehydratase	X	Phyllobacterium sp.	
G8YMK4	D-galactonate dehydratase	X	Pichia sorbitophila	
I4X5Q4	D-galactonate dehydratase	X	Planococcus antarcticus	
M3F9W3	D-galactonate dehydratase	X	Planococcus halocryophilus	
A0A0B4REV5	D-galactonate dehydratase	X	Planococcus sp.	
A0A0A2TEK0	D-galactonate dehydratase	X	Pontibacillus yanchengensis	
A0A0B3RU98	D-galactonate dehydratase	X	Ponticoccus sp.	4.2.1.39
A0A0B2BVQ3	D-galactonate dehydratase	X	Porphyrobacter mercurialis	
A0A0C1L4Q3	D-galactonate dehydratase	X	Prauserella sp.	4.2.1.6
A0A0C1LEZ4	D-galactonate dehydratase	X	Prauserella sp.	4.2.1.6
K8W854	D-galactonate dehydratase	X	Providencia burhodogranariea	
M2Z2K0	D-galactonate dehydratase	X	Pseudocercospora fijiensis	
A0A094A750	D-galactonate dehydratase	X	Pseudogymnoascus sp.	
A0A094DSZ4	D-galactonate dehydratase	X	Pseudogymnoascus sp.	
A0A094EKK3	D-galactonate dehydratase	X	Pseudogymnoascus sp.	
A0A094EVV6	D-galactonate dehydratase	X	Pseudogymnoascus sp.	
A0A094FIJ4	D-galactonate dehydratase	X	Pseudogymnoascus sp.	
A0A094HSE1	D-galactonate dehydratase	X	Pseudogymnoascus sp.	
A0A094KCP3	D-galactonate dehydratase	X	Pseudogymnoascus sp.	
A0A0C2I9F1	D-galactonate dehydratase	X	Pseudomonas batumici	
A0A0E1E7C5	D-galactonate dehydratase	X	Pseudomonas chlororaphis	
W0H719	D-galactonate dehydratase	X	Pseudomonas cichorii	
A0A075PKU4	D-galactonate dehydratase	X	Pseudomonas fluorescens	
A0A0D0TP03	D-galactonate dehydratase	X	Pseudomonas fluorescens	4.2.1.6
A0A098SYM1	D-galactonate dehydratase	X	Pseudomonas lutea	
A0A0A4GQ22	D-galactonate dehydratase	X	Pseudomonas mediterranea	
A0A0D7FCC9	D-galactonate dehydratase	X	Pseudomonas oryzihabitans	
A0A077LQU4	D-galactonate dehydratase	X	Pseudomonas sp.	
A0A0A1GAY8	D-galactonate dehydratase	X	Pseudomonas sp.	
J0P6I4	D-galactonate dehydratase	X	Pseudomonas sp.	
J2WZ75	D-galactonate dehydratase	X	Pseudomonas sp.	
M5QW94	D-galactonate dehydratase	X	Pseudomonas sp.	
N2J5S8	D-galactonate dehydratase	X	Pseudomonas sp.	
S6IKE7	D-galactonate dehydratase	X	Pseudomonas sp.	
S6JEQ7	D-galactonate dehydratase	X	Pseudomonas sp.	
U3HTV8	D-galactonate dehydratase	X	Pseudomonas stutzeri	
I4L5B0	D-galactonate dehydratase	X	Pseudomonas synxantha	4.2.1.6
A0A085VHB9	D-galactonate dehydratase	X	Pseudomonas syringae	
A0A0E3Z2T1	D-galactonate dehydratase	X	Pseudoxanthomonas suwonensis	4.2.1.6
E6WRT9	D-galactonate dehydratase	X	Pseudoxanthomonas suwonensis	4.2.1.6
E3RYL6	D-galactonate dehydratase	X	Pyrenophora teres	
U4KUK6	D-galactonate dehydratase	X	Pyronema omphalodes	
H8NV05	D-galactonate dehydratase	X	Rahnella aquatilis	4.2.1.6
B2UCA8	D-galactonate dehydratase	X	Ralstonia pickettii	4.2.1.6
D8NY59	D-galactonate dehydratase	X	Ralstonia solanacearum	4.2.1.6
Q8XVS8	D-galactonate dehydratase	X	Ralstonia solanacearum	4.2.1.6
A0A0B1Y2S0	D-galactonate dehydratase	X	Ralstonia sp.	
S9RW45	D-galactonate dehydratase	X	Ralstonia sp.	4.2.1.6
U3GJ88	D-galactonate dehydratase	X	Ralstonia sp.	4.2.1.6
G2ZYU5	D-galactonate dehydratase	X	Ralstonia syzygii	4.2.1.6

Table S2.6. (continuation)

UniProt	Family [3]	Cluster	Species	EC Number
A0A0F4YEQ1	D-galactonate dehydratase	X	Rasamsonia emersonii	4.2.1.6
A0A0F4YZV0	D-galactonate dehydratase	X	Rasamsonia emersonii	4.2.1.6
A0A0D2I5Q0	D-galactonate dehydratase	X	Rhinocladiella mackenziei	
A0A060I5H4	D-galactonate dehydratase	X	Rhizobium etli	4.2.1.6
B3Q0W7	D-galactonate dehydratase	X	Rhizobium etli	4.2.1.6
S5SN52	D-galactonate dehydratase	X	Rhizobium etli	4.2.1.6
A0A0B4X459	D-galactonate dehydratase	X	Rhizobium gallicum	4.2.1.6
A0A0B4XCQ8	D-galactonate dehydratase	X	Rhizobium gallicum	4.2.1.6
S3I3G7	D-galactonate dehydratase	X	Rhizobium grahamii	
A0A072C4C1	D-galactonate dehydratase	X	Rhizobium leguminosarum	
Q1MBG1	D-galactonate dehydratase	X	Rhizobium leguminosarum	
A0A061N0X3	D-galactonate dehydratase	X	Rhizobium rhizogenes	
J2L2S0	D-galactonate dehydratase	X	Rhizobium sp.	
J6DPT9	D-galactonate dehydratase	X	Rhizobium sp.	
W6WPM6	D-galactonate dehydratase	X	Rhizobium sp.	4.2.1.6
L0LME8	D-galactonate dehydratase	X	Rhizobium tropici	
A0A074SYN8	D-galactonate dehydratase	X	Rhizoctonia solani	
I4VQ11	D-galactonate dehydratase	X	Rhodanobacter fulvus	
I4WEL8	D-galactonate dehydratase	X	Rhodanobacter thiooxydans	
L1KC33	D-galactonate dehydratase	X	Rhodobacter sp.	
A3PQU8	D-galactonate dehydratase	X	Rhodobacter sphaeroides	4.2.1.6
I0WW71	D-galactonate dehydratase	X	Rhodococcus imtechensis	
C1AVY0	D-galactonate dehydratase	X	Rhodococcus opacus	4.2.1.6
Q21ZV7	D-galactonate dehydratase	X	Rhodoferax ferrireducens	4.2.1.6
A0A0F2REK8	D-galactonate dehydratase	X	Rhodospirillaceae bacterium	
H5XGM4	D-galactonate dehydratase	X	Saccharomonospora cyanea	
R4W279	D-galactonate dehydratase	X	Salinarchaeum sp.	
A0A0C2DKX6	D-galactonate dehydratase	X	Salinicoccus roseus	
A0A084IH94	D-galactonate dehydratase	X	Salinisphaera hydrothermalis	
V7UB31	D-galactonate dehydratase	X	Salmonella enterica	4.2.1.6
B5RFZ8	D-galactonate dehydratase	X	Salmonella gallinarum	4.2.1.6
W9CHQ7	D-galactonate dehydratase	X	Sclerotinia borealis	
A7EXK2	D-galactonate dehydratase	X	Sclerotinia sclerotiorum	
F8PKT1	D-galactonate dehydratase	X	Serpula lacrymans	
R0K4K2	D-galactonate dehydratase	X	Setosphaeria turcica	
E7SZE3	D-galactonate dehydratase	X	Shigella boydii	4.2.1.6
I6DRX7	D-galactonate dehydratase	X	Shigella boydii	4.2.1.6
A0A0B2AQK7	D-galactonate dehydratase	X	Sinomonas humi	
A6UFI2	D-galactonate dehydratase	X	Sinorhizobium medicae	
F7XHC4	D-galactonate dehydratase	X	Sinorhizobium meliloti	
H0FV32	D-galactonate dehydratase	X	Sinorhizobium meliloti	
W0HST0	D-galactonate dehydratase	X	Sodalis praecaptivus	4.2.1.6
A0A0C9V2F3	D-galactonate dehydratase	X	Sphaerobolus stellatus	
M3DBN2	D-galactonate dehydratase	X	Sphaerulina musiva	
A0A0E9MJR2	D-galactonate dehydratase	X	Sphingomonas changbaiensis	
W0A8L8	D-galactonate dehydratase	X	Sphingomonas sanxanigenens	
A0A0F5P847	D-galactonate dehydratase	X	Sphingomonas sp.	
A0A0C2IZ29	D-galactonate dehydratase	X	Sporothrix brasiliensis	
A0A084AMG5	D-galactonate dehydratase	X	Stachybotrys chartarum	
A0A033V089	D-galactonate dehydratase	X	Staphylococcus aureus	
A0A0D0I3J0	D-galactonate dehydratase	X	Stenotrophomonas maltophilia	4.2.1.6
D7BPU0	D-galactonate dehydratase	X	Streptomyces bingchenggensis	
D7CBV2	D-galactonate dehydratase	X	Streptomyces bingchenggensis	
Q9RKG2	D-galactonate dehydratase	X	Streptomyces coelicolor	
L1KWN1	D-galactonate dehydratase	X	Streptomyces ipomoeae	
A0A066YAA3	D-galactonate dehydratase	X	Streptomyces olindensis	

Table S2.6. (continuation)

UniProt	Family [3]	Cluster	Species	EC Number
A0A0A0NMF1	D-galactonate dehydratase	X	Streptomyces rapamycinicus	
A0A014M7L1	D-galactonate dehydratase	X	Streptomyces sp.	
B4V8E9	D-galactonate dehydratase	X	Streptomyces sp.	
S2YDR5	D-galactonate dehydratase	X	Streptomyces sp.	
L7F0X3	D-galactonate dehydratase	X	Streptomyces turgidiscabies	
E8LMQ2	D-galactonate dehydratase	X	Succinatimonas hippei	
A0A0D0BJP5	D-galactonate dehydratase	X	Suillus luteus	
A0A061SLU8	D-galactonate dehydratase	X	Sulfitobacter mediterraneus	
A0A0B8N2H4	D-galactonate dehydratase	X	Talaromyces cellulolyticus	
B6QUQ8	D-galactonate dehydratase	X	Talaromyces marneffei	
B8MRZ5	D-galactonate dehydratase	X	Talaromyces stipitatus	4.2.1.6
A0A095VQ84	D-galactonate dehydratase	X	Tatumella morbirosei	
F1ZS13	D-galactonate dehydratase	X	Thermoanaerobacter ethanolicus	
M8DH50	D-galactonate dehydratase	X	Thermoanaerobacter thermohydrosulfuricus	4.2.1.6
L0IL05	D-galactonate dehydratase	X	Thermoanaerobacterium thermosac.	
F6BJT6	D-galactonate dehydratase	X	Thermoanaerobacterium	4.2.1.6
G2R5R1	D-galactonate dehydratase	X	Thielavia terrestris	
A0A074TK90	D-galactonate dehydratase	X	Thioclava dalianensis	
A0A074JFM6	D-galactonate dehydratase	X	Thioclava sp.	
R8BVK3	D-galactonate dehydratase	X	Togninia minima	
F5YNJ8	D-galactonate dehydratase	X	Treponema primitia	
F2PKL1	D-galactonate dehydratase	X	Trichophyton equinum	
A0A022U7S8	D-galactonate dehydratase	X	Trichophyton interdigitale	
A0A023A3S9	D-galactonate dehydratase	X	Trichophyton rubrum	
A0A022XHA1	D-galactonate dehydratase	X	Trichophyton soudanense	
D5G4N6	D-galactonate dehydratase	X	Tuber melanosporum	
C5CZC3	D-galactonate dehydratase	X	Variovorax paradoxus	4.2.1.6
A1WPC7	D-galactonate dehydratase	X	Verminephrobacter eiseniae	4.2.1.6
A0A0D2ACH6	D-galactonate dehydratase	X	Verruconis gallopava	
C9SSB2	D-galactonate dehydratase	X	Verticillium alfalfae	
B5ESA8	D-galactonate dehydratase	X	Vibrio fischeri	4.2.1.6
U4E4V2	D-galactonate dehydratase	X	Vibrio nigripulchritudo	4.2.1.6
U4KBW3	D-galactonate dehydratase	X	Vibrio nigripulchritudo	4.2.1.6
F9RV61	D-galactonate dehydratase	X	Vibrio scophthalmi	
Q7MC07	D-galactonate dehydratase	X	Vibrio vulnificus	
A0A024QI82	D-galactonate dehydratase	X	Virgibacillus sp.	
Q3BUN6	D-galactonate dehydratase	X	Xanthomonas campestris	4.2.1.6
K8Z085	D-galactonate dehydratase	X	Xanthomonas translucens	4.2.1.6
A0A068QS56	D-galactonate dehydratase	X	Xenorhabdus doucetiae	4.2.1.6
A0A068R3R4	D-galactonate dehydratase	X	Xenorhabdus poinarii	4.2.1.6
G0L9L1	D-galactonate dehydratase	X	Zobellia galactanivorans	4.2.1.6
A0A0F4GEX9	D-galactonate dehydratase	X	Zymoseptoria brevis	
H0FG41	D-tartrate dehydratase	III	Achromobacter arsenitoxydans	
A1TM00	D-tartrate dehydratase	III	Acidovorax citrulli	
A6VKU6	D-tartrate dehydratase	III	Actinobacillus succinogenes	
V8QM71	D-tartrate dehydratase	III	Advenella kashmirensis	
W0PCS1	D-tartrate dehydratase	III	Advenella mimigardefordensis	
D6V6U3	D-tartrate dehydratase	III	Afipia sp.	
B9JIP3	D-tartrate dehydratase	III	Agrobacterium radiobacter	
H0HGR9	D-tartrate dehydratase	III	Agrobacterium tumefaciens	
E0MTW3	D-tartrate dehydratase	III	Ahrensia sp.	
A0A0D6IK15	D-tartrate dehydratase	III	Alcaligenes xylosoxydans	5.1.2.2
A8TSK2	D-tartrate dehydratase	III	alpha proteobacterium	
A1K4V8	D-tartrate dehydratase	III	Azoarcus sp.	5.1.2.2
A9IDH2	D-tartrate dehydratase	III	Bordetella petrii	
A0A085F3N2	D-tartrate dehydratase	III	Bosea sp.	

Table S2.6. (continuation)

UniProt	Family [3]	Cluster	Species	EC Number
F7QGV7	D-tartrate dehydratase	III	Bradyrhizobiaceae bacterium	
Q89FH0	D-tartrate dehydratase	III	Bradyrhizobium diazoefficiens	4.2.1.81
A0A0D1NNW7	D-tartrate dehydratase	III	Bradyrhizobium elkanii	
A0A023XP17	D-tartrate dehydratase	III	Bradyrhizobium japonicum	
A5EB76	D-tartrate dehydratase	III	Bradyrhizobium sp.	
H0S7T3	D-tartrate dehydratase	III	Bradyrhizobium sp.	
H0S9A1	D-tartrate dehydratase	III	Bradyrhizobium sp.	
H5YA29	D-tartrate dehydratase	III	Bradyrhizobium sp.	
I0GG43	D-tartrate dehydratase	III	Bradyrhizobium sp.	
J3CZX5	D-tartrate dehydratase	III	Bradyrhizobium sp.	
U1GUA6	D-tartrate dehydratase	III	Bradyrhizobium sp.	
B1Z001	D-tartrate dehydratase	III	Burkholderia ambifaria	
Q0B4I4	D-tartrate dehydratase	III	Burkholderia ambifaria	
B1JWY1	D-tartrate dehydratase	III	Burkholderia cenocepacia	
A0A095YBM6	D-tartrate dehydratase	III	Burkholderia gladioli	
F2LDA0	D-tartrate dehydratase	III	Burkholderia gladioli	
B1G865	D-tartrate dehydratase	III	Burkholderia graminis	
A0A038GM30	D-tartrate dehydratase	III	Burkholderia jiangsuensis	
A0A038GTM5	D-tartrate dehydratase	III	Burkholderia jiangsuensis	
A0A095H768	D-tartrate dehydratase	III	Burkholderia mallei	
A0A088WZB8	D-tartrate dehydratase	III	Burkholderia oklahomensis	
B2JWU2	D-tartrate dehydratase	III	Burkholderia phymatum	
G8MGT9	D-tartrate dehydratase	III	Burkholderia sp.	
K8RD27	D-tartrate dehydratase	III	Burkholderia sp.	
Q142W1	D-tartrate dehydratase	III	Burkholderia xenovorans	
Q11AL1	D-tartrate dehydratase	III	Chelativorans sp.	
A0A0A3AKX0	D-tartrate dehydratase	III	Chelonobacter oris	
A0A023V4E0	D-tartrate dehydratase	III	Citrobacter freundii	
D4BIC8	D-tartrate dehydratase	III	Citrobacter youngae	
A0A069I6T3	D-tartrate dehydratase	III	Cupriavidus sp.	
A9BZR9	D-tartrate dehydratase	III	Delftia acidovorans	
W7WM03	D-tartrate dehydratase	III	Hydrogenophaga sp.	5.1.2.2
A0A0A0D5Z3	D-tartrate dehydratase	III	Inquilinus limosus	
W0V4W8	D-tartrate dehydratase	III	Janthinobacterium agaricidamnosum	
A0A0A6DY06	D-tartrate dehydratase	III	Janthinobacterium lividum	
A0A031GV27	D-tartrate dehydratase	III	Janthinobacterium lividum	5.1.2.2
A6SWV0	D-tartrate dehydratase	III	Janthinobacterium sp.	
L9PDE5	D-tartrate dehydratase	III	Janthinobacterium sp.	5.1.2.2
B9R305	D-tartrate dehydratase	III	Labrenzia alexandrii	
A0A085HPN1	D-tartrate dehydratase	III	Leminorella grimontii	5.3.3.4
A6GLG6	D-tartrate dehydratase	III	Limnobacter sp.	
V4RD70	D-tartrate dehydratase	III	Lutibaculum baratangense	5.3.3.4
A3JI80	D-tartrate dehydratase	III	Marinobacter sp.	
A0A098U8A6	D-tartrate dehydratase	III	Massilia sp.	
A9VZX8	D-tartrate dehydratase	III	Methylobacterium extorquens	
B8IQ93	D-tartrate dehydratase	III	Methylobacterium nodulans	
B1LTV7	D-tartrate dehydratase	III	Methylobacterium radiotolerans	
B0U9L4	D-tartrate dehydratase	III	Methylobacterium sp.	
F8BT98	D-tartrate dehydratase	III	Oligotropha carboxidovorans	
F1VWA1	D-tartrate dehydratase	III	Oxalobacteraceae bacterium	
A0A059IA93	D-tartrate dehydratase	III	Pantoea agglomerans	
A0A0F5XWG5	D-tartrate dehydratase	III	Pantoea sp.	
A1VSV9	D-tartrate dehydratase	III	Polaromonas naphthalenivorans	
Q128N0	D-tartrate dehydratase	III	Polaromonas sp.	
I4KJZ0	D-tartrate dehydratase	III	Pseudomonas fluorescens	
B1JFP6	D-tartrate dehydratase	III	Pseudomonas putida	

Table S2.6. (continuation)

UniProt	Family [3]	Cluster	Species	EC Number
A0A0A1HTQ3	D-tartrate dehydratase	III	Pseudomonas sp.	
U3QYF2	D-tartrate dehydratase	III	Ralstonia pickettii	
D8NSU3	D-tartrate dehydratase	III	Ralstonia solanacearum	5.1.2.2
G3A4Z7	D-tartrate dehydratase	III	Ralstonia syzygii	5.1.2.2
A0A068SPE3	D-tartrate dehydratase	III	Rhizobium galegae	
C6B5Y3	D-tartrate dehydratase	III	Rhizobium leguminosarum	
J2AYY5	D-tartrate dehydratase	III	Rhizobium sp.	
L0NCA9	D-tartrate dehydratase	III	Rhizobium sp.	
A3PP64	D-tartrate dehydratase	III	Rhodobacter sphaeroides	
Q3IWU6	D-tartrate dehydratase	III	Rhodobacter sphaeroides	5.1.2.2
Q220G1	D-tartrate dehydratase	III	Rhodoferax ferrireducens	
W8S1G1	D-tartrate dehydratase	III	Roseibacterium elongatum	5.3.3.4
A3SNI2	D-tartrate dehydratase	III	Roseovarius nubinhibens	
U2NH33	D-tartrate dehydratase	III	Serratia fonticola	
D4E6I6	D-tartrate dehydratase	III	Serratia odorifera	
W9GTX7	D-tartrate dehydratase	III	Skermanella stibiiresistens	
A0A074JQX5	D-tartrate dehydratase	III	Thioclava sp.	
Q6SEZ0	D-tartrate dehydratase	III	uncultured marine	
C5CTU5	D-tartrate dehydratase	III	Variovorax paradoxus	
C5CZG6	D-tartrate dehydratase	III	Variovorax paradoxus	
T1XK27	D-tartrate dehydratase	III	Variovorax paradoxus	
A0A081W9C4	D-tartrate dehydratase	III	Xanthomonas arboricola	
E5U9F5	D-tartrate dehydratase	XI	Achromobacter xylosoxidans	
H1SGW5	D-tartrate dehydratase	XI	Cupriavidus basilensis	
G8QHC1	D-tartrate dehydratase	XI	Dechlorosoma suillum	
I4Z0J0	D-tartrate dehydratase	XI	Microvirga lotononidis	
F4GRA0	D-tartrate dehydratase	XI	Pusillimonas sp.	
F0C1X9	D-tartrate dehydratase	XI	Xanthomonas gardneri	
A0A084JLZ8	dipeptide epimerase	IX	[Clostridium] celerecrescens	
A0A084JR08	dipeptide epimerase	IX	[Clostridium] celerecrescens	
M1Z6Y7	dipeptide epimerase	IX	[Clostridium] ultunense	
I4VAS8	dipeptide epimerase	IX		
F7NQB8	dipeptide epimerase	IX	Acetonema longum	
C0WAE1	dipeptide epimerase	IX	Acidaminococcus sp.	
R7LYD3	dipeptide epimerase	IX	Acidaminococcus sp.	
U2VT31	dipeptide epimerase	IX	Acidaminococcus sp.	
A0A090W511	dipeptide epimerase	IX	Algibacter lectus	
J2II24	dipeptide epimerase	IX	Alishewanella aestuarii	
I8U6N7	dipeptide epimerase	IX	Alishewanella agri	
H3ZDI6	dipeptide epimerase	IX	Alishewanella jeotgali	
A6TKI4	dipeptide epimerase	IX	Alkaliphilus metalliredigens	
A8MEZ5	dipeptide epimerase	IX	Alkaliphilus oremlandii	
U5DA53	dipeptide epimerase	IX	Amborella trichopoda	
D5EEU9	dipeptide epimerase	IX	Aminobacterium colombiense	
A0A0D1XZS9	dipeptide epimerase	IX	Aneurinibacillus migulanus	
Q9LJQ4	dipeptide epimerase	IX	Arabidopsis thaliana	
D2REB1	dipeptide epimerase	IX	Archaeoglobus profundus	
R9GM51	dipeptide epimerase	IX	Arcticibacter svalbardensis	
A0A078MQS7	dipeptide epimerase	IX	Arthrobacter sp.	
E1USC4	dipeptide epimerase	IX	Bacillus amyloliquefaciens	5.1.1.-
I2C418	dipeptide epimerase	IX	Bacillus amyloliquefaciens	5.5.1.7
A0A0C2YBL7	dipeptide epimerase	IX	Bacillus badius	
W4RM74	dipeptide epimerase	IX	Bacillus boroniphilus	
A0A084H2G3	dipeptide epimerase	IX	Bacillus cibi	
A0A0B5X5V8	dipeptide epimerase	IX	Bacillus coagulans	
F7YZM8	dipeptide epimerase	IX	Bacillus coagulans	

Table S2.6. (continuation)

UniProt	Family [3]	Cluster	Species	EC Number
G2TIP6	dipeptide epimerase	IX	Bacillus coagulans	
A0A084GZ01	dipeptide epimerase	IX	Bacillus indicus	
U5L7W8	dipeptide epimerase	IX	Bacillus infantis	
K1KNP7	dipeptide epimerase	IX	Bacillus isronensis	5.1.1.-
W7RD90	dipeptide epimerase	IX	Bacillus licheniformis	
A0A0B6ARC0	dipeptide epimerase	IX	Bacillus megaterium	
G2RTV8	dipeptide epimerase	IX	Bacillus megaterium	
D5DA58	dipeptide epimerase	IX	Bacillus megaterium	5.5.1.1
I3E8S6	dipeptide epimerase	IX	Bacillus methanolicus	
S6G1J5	dipeptide epimerase	IX	Bacillus methylotrophicus	5.1.1.-
R9C9T0	dipeptide epimerase	IX	Bacillus nealsonii	
A0A0B0IDJ3	dipeptide epimerase	IX	Bacillus okhensis	
A0A063Z287	dipeptide epimerase	IX	Bacillus pumilus	
A8FCA7	dipeptide epimerase	IX	Bacillus pumilus	
W8QYR3	dipeptide epimerase	IX	Bacillus pumilus	
M5P5C2	dipeptide epimerase	IX	Bacillus sonorensis	
A0A077JAJ7	dipeptide epimerase	IX	Bacillus sp.	
A0A081LA70	dipeptide epimerase	IX	Bacillus sp.	
A0A0A8JFU0	dipeptide epimerase	IX	Bacillus sp.	
A0A0B4S6I7	dipeptide epimerase	IX	Bacillus sp.	
A0A0F5KNU7	dipeptide epimerase	IX	Bacillus sp.	
A0A0F5MEA6	dipeptide epimerase	IX	Bacillus sp.	
E5WSI0	dipeptide epimerase	IX	Bacillus sp.	
I2HQ32	dipeptide epimerase	IX	Bacillus sp.	
M5R2Q3	dipeptide epimerase	IX	Bacillus stratosphericus	
A0A0C2U172	dipeptide epimerase	IX	Bacillus subtilis	
L8PXW3	dipeptide epimerase	IX	Bacillus subtilis	
O34508	dipeptide epimerase	IX	Bacillus subtilis	5.1.1.20
A0A080UJR7	dipeptide epimerase	IX	Bacillus subtilis	5.1.1.n1
A0A090J2Z1	dipeptide epimerase	IX	Bacillus thermoamylovorans	
W1SJ57	dipeptide epimerase	IX	Bacillus vireti	
K2NJ74	dipeptide epimerase	IX	Bacillus xiamenensis	
A0A081C487	dipeptide epimerase	IX	bacterium UASB270	
A0A081C7I2	dipeptide epimerase	IX	bacterium UASB270	
R6G2Z5	dipeptide epimerase	IX	Blautia sp.	
C7MGS5	dipeptide epimerase	IX	Brachybacterium faecium	
A0A022KZD5	dipeptide epimerase	IX	Brachybacterium muris	
Z9JUD1	dipeptide epimerase	IX	Brachybacterium phenoliresistens	
I1HBT7	dipeptide epimerase	IX	Brachypodium distachyon	
A0A078DY77	dipeptide epimerase	IX	Brassica napus	
A0A078GGZ7	dipeptide epimerase	IX	Brassica napus	
C0ZIC3	dipeptide epimerase	IX	Brevibacillus brevis	
A0A075R5E9	dipeptide epimerase	IX	Brevibacillus laterosporus	5.1.1.-
V6LYY7	dipeptide epimerase	IX	Brevibacillus panacihumi	
A0A0F5JUI6	dipeptide epimerase	IX	Burkholderia andropogonis	
C7DGW9	dipeptide epimerase	IX	Candidatus Micrarchaeum	
C7DGX5	dipeptide epimerase	IX	Candidatus Micrarchaeum	
F5RPF9	dipeptide epimerase	IX	Centipeda periodontii	4.2.1.6
A0A081SHV6	dipeptide epimerase	IX	Chlorobium sp.	
B8G3Y7	dipeptide epimerase	IX	Chloroflexus aggregans	
A9WD47	dipeptide epimerase	IX	Chloroflexus aurantiacus	
A0A017T6B2	dipeptide epimerase	IX	Chondromyces apiculatus	
V4VRY4	dipeptide epimerase	IX	Citrus clementina	
A0A067E7G1	dipeptide epimerase	IX	Citrus sinensis	
U2DC79	dipeptide epimerase	IX	Clostridiales bacterium	
R5T5Y1	dipeptide epimerase	IX	Clostridium hathewayi	

Table S2.6. (continuation)

UniProt	Family [3]	Cluster	Species	EC Number
D9R4S8	dipeptide epimerase	IX	Clostridium saccharolyticum	
M1N0H6	dipeptide epimerase	IX	Clostridium saccharoperbutylacetonicum	5.1.1.n1
A0A011ANF1	dipeptide epimerase	IX	Clostridium sp.	
A0A011ATP9	dipeptide epimerase	IX	Clostridium sp.	
T0N5R8	dipeptide epimerase	IX	Clostridium sp.	
A0A084JBV5	dipeptide epimerase	IX	Clostridium sulfidigenes	
W6N6I2	dipeptide epimerase	IX	Clostridium tyrobutyricum	
A0A068TQU1	dipeptide epimerase	IX	Coffea canephora	
A0A0C2UI58	dipeptide epimerase	IX	Cohnella sp.	
L2FPB5	dipeptide epimerase	IX	Colletotrichum gloeosporioides	
H8MT79	dipeptide epimerase	IX	Corallococcus coralloides	
A0A086E0L3	dipeptide epimerase	IX	Cryobacterium sp.	
S9QJ63	dipeptide epimerase	IX	Cystobacter fuscus	
A0A084SFQ8	dipeptide epimerase	IX	Cystobacter violaceus	
A0A0C7NLP7	dipeptide epimerase	IX	Defluviitoga tunisiensis	5.1.1.-
D8FAG0	dipeptide epimerase	IX	delta proteobacterium	
C0QM06	dipeptide epimerase	IX	Desulfobacterium autotrophicum	5.1.1.-
D2Z6Z6	dipeptide epimerase	IX	Dethiosulfovibrio peptidovorans	
X5DJU8	dipeptide epimerase	IX	Draconibacterium orientale	
A0A075K3M1	dipeptide epimerase	IX	Dyella japonica	
A0A023NXZ6	dipeptide epimerase	IX	Dyella jiangningensis	
I2F0E0	dipeptide epimerase	IX	Emticicia oligotrophica	
R3TSQ2	dipeptide epimerase	IX	Enterococcus caccae	
F0ENU6	dipeptide epimerase	IX	Enterococcus casseliflavus	
S4C9L7	dipeptide epimerase	IX	Enterococcus casseliflavus	
D4EQ88	dipeptide epimerase	IX	Enterococcus faecalis	
E6LFV9	dipeptide epimerase	IX	Enterococcus italicus	5.1.2.2
R2SZK9	dipeptide epimerase	IX	Enterococcus moraviensis	
G5IQU4	dipeptide epimerase	IX	Enterococcus saccharolyticus	
T0VJM2	dipeptide epimerase	IX	Enterococcus sp.	
J1HX67	dipeptide epimerase	IX	Enterococcus sp.	4.2.1.6
A0A022PXR9	dipeptide epimerase	IX	Erythranthe guttata	
E3GKX2	dipeptide epimerase	IX	Eubacterium limosum	
A0A059D0H4	dipeptide epimerase	IX	Eucalyptus grandis	
D4K2E7	dipeptide epimerase	IX	Faecalibacterium prausnitzii	5.5.1.1
R6Q5C0	dipeptide epimerase	IX	Faecalibacterium sp.	
T0M9I4	dipeptide epimerase	IX	Ferroplasma sp.	
T0NII3	dipeptide epimerase	IX	Ferroplasma sp.	
A0A017RSK1	dipeptide epimerase	IX	Fervidicella metallireducens	
I0K6L5	dipeptide epimerase	IX	Fibrella aestuarina	
I2GB30	dipeptide epimerase	IX	Fibrisoma limi	
R5EZD7	dipeptide epimerase	IX	Firmicutes bacterium	
A0A0C1KZN6	dipeptide epimerase	IX	Flavihumibacter solisilvae	
A0A0C1KP48	dipeptide epimerase	IX	Flavihumibacter sp.	
F4BK52	dipeptide epimerase	IX	Francisella cf.	5.5.1.1
A0A0B6D3A1	dipeptide epimerase	IX	Francisella philomiragia	
A0A0E2ZF79	dipeptide epimerase	IX	Francisella philomiragia	
C6YVF4	dipeptide epimerase	IX	Francisella philomiragia	
B0TZW0	dipeptide epimerase	IX	Francisella philomiragia	5.1.1.-
A0A097EMR0	dipeptide epimerase	IX	Francisella sp.	
A0Q5S7	dipeptide epimerase	IX	Francisella tularensis	5.1.1.20
L8JKW3	dipeptide epimerase	IX	Fulvivirga imtechensis	
K2KJT5	dipeptide epimerase	IX	Gallaecimonas xiamenensis	
W2UFN2	dipeptide epimerase	IX	Gammaproteobacteria bacterium	5.1.1.-
A0A0F6BI08	dipeptide epimerase	IX	Geobacillus sp.	
C5D8X6	dipeptide epimerase	IX	Geobacillus sp.	

Table S2.6. (continuation)

UniProt	Family [3]	Cluster	Species	EC Number
A4IT72	dipeptide epimerase	IX	Geobacillus thermodenitrificans	
B5EFW2	dipeptide epimerase	IX	Geobacter bemidjiensis	5.1.1.-
C6E6L3	dipeptide epimerase	IX	Geobacter sp.	
E8WIZ2	dipeptide epimerase	IX	Geobacter sp.	
Q7NC80	dipeptide epimerase	IX	Gloeobacter violaceus	
I1KRS2	dipeptide epimerase	IX	Glycine max	
F4L0J9	dipeptide epimerase	IX	Haliscomenobacter hydrossis	5.5.1.7
M0KKJ6	dipeptide epimerase	IX	Haloarcula amylolytica	
M0KQJ4	dipeptide epimerase	IX	Haloarcula argentinensis	
M0KKU0	dipeptide epimerase	IX	Haloarcula californiae	
G0HWQ5	dipeptide epimerase	IX	Haloarcula hispanica	
M0L9L4	dipeptide epimerase	IX	Haloarcula japonica	
Q5V5B0	dipeptide epimerase	IX	Haloarcula marismortui	
M0JXI3	dipeptide epimerase	IX	Haloarcula sinaiiensis	
M0JF73	dipeptide epimerase	IX	Haloarcula vallismortis	
A0A024P481	dipeptide epimerase	IX	Halobacillus karajensis	
A0A0B0D4X0	dipeptide epimerase	IX	Halobacillus sp.	
L5N601	dipeptide epimerase	IX	Halobacillus sp.	
M0J9L5	dipeptide epimerase	IX	Haloferax denitrificans	
M0H5U5	dipeptide epimerase	IX	Haloferax larsenii	
M0ITK0	dipeptide epimerase	IX	Haloferax sulfurifontis	
D4GR94	dipeptide epimerase	IX	Haloferax volcanii	
G2MN08	dipeptide epimerase	IX	halophilic archaeon	5.5.1.1
A0A0C1XMY1	dipeptide epimerase	IX	Hassallia byssoidea	
A9B055	dipeptide epimerase	IX	Herpetosiphon aurantiacus	5.1.1.-
D3A9C3	dipeptide epimerase	IX	Hungatella hathewayi	
G5IEY3	dipeptide epimerase	IX	Hungatella hathewayi	
N9XP57	dipeptide epimerase	IX	Hungatella hathewayi	
A0A085WKR4	dipeptide epimerase	IX	Hyalangium minutum	
A2BKM9	dipeptide epimerase	IX	Hyperthermus butylicus	5.5.1.1
A0A094J5T3	dipeptide epimerase	IX	Idiomarina sp.	
A0A067K9Q8	dipeptide epimerase	IX	Jatropha curcas	
A0A067KSD4	dipeptide epimerase	IX	Jatropha curcas	
A0A0C2RNZ9	dipeptide epimerase	IX	Jeotgalibacillus alimentarius	4.2.1.113
A0A0C2W8F6	dipeptide epimerase	IX	Jeotgalibacillus campisalis	4.2.1.113
A0A0C2S668	dipeptide epimerase	IX	Jeotgalibacillus soli	4.2.1.113
A0A0B5AVJ1	dipeptide epimerase	IX	Jeotgalibacillus sp.	4.2.1.113
C9M6L4	dipeptide epimerase	IX	Jonquetella anthropi	
D6TF52	dipeptide epimerase	IX	Ktedonobacter racemifer	
D6TQE1	dipeptide epimerase	IX	Ktedonobacter racemifer	
R9JZ19	dipeptide epimerase	IX	Lachnospiraceae bacterium	
F4FWD5	dipeptide epimerase	IX	Lactobacillus buchneri	5.5.1.1
K6S834	dipeptide epimerase	IX	Lactobacillus casei	4.2.1.-
A0A0A6NV92	dipeptide epimerase	IX	Lactobacillus curieae	
X0P928	dipeptide epimerase	IX	Lactobacillus farraginis	
A0A0A1GXW0	dipeptide epimerase	IX	Lactobacillus hokkaidonensis	
I7LAQ0	dipeptide epimerase	IX	Lactobacillus hominis	5.5.1.7
A0A081BGQ1	dipeptide epimerase	IX	Lactobacillus oryzae	
A0A081BKA3	dipeptide epimerase	IX	Lactobacillus oryzae	
A0A0C9QNK2	dipeptide epimerase	IX	Lactobacillus paracasei	
S2N5R8	dipeptide epimerase	IX	Lactobacillus paracasei	
S2QR68	dipeptide epimerase	IX	Lactobacillus paracasei	
C7T9Y2	dipeptide epimerase	IX	Lactobacillus rhamnosus	
K8QQA1	dipeptide epimerase	IX	Lactobacillus rhamnosus	5.5.1.1
A0A098G8P2	dipeptide epimerase	IX	Legionella fallonii	
A0A078KTS3	dipeptide epimerase	IX	Legionella massiliensis	

Table S2.6. (continuation)

UniProt	Family [3]	Cluster	Species	EC Number
A0A078KZD7	dipeptide epimerase	IX	Legionella massiliensis	
A0A0A2T5K5	dipeptide epimerase	IX	Legionella norrlandica	
D5TEM8	dipeptide epimerase	IX	Legionella pneumophila	5.5.1.1
V9VRJ8	dipeptide epimerase	IX	Leisingera methylohalidivorans	
A0A0B4BMP9	dipeptide epimerase	IX	Leisingera sp.	
A0A0B4CPP5	dipeptide epimerase	IX	Leisingera sp.	
A0A0B4EE62	dipeptide epimerase	IX	Leisingera sp.	
A0A0C1EUE8	dipeptide epimerase	IX	Leisingera sp.	
A0A0C1G786	dipeptide epimerase	IX	Leisingera sp.	
A0A0C1GZW7	dipeptide epimerase	IX	Leisingera sp.	
A0A0C1IVK8	dipeptide epimerase	IX	Leisingera sp.	
A0A062XEM1	dipeptide epimerase	IX	Leuconostoc pseudomesenteroides	
A0A097B1S9	dipeptide epimerase	IX	Listeria ivanovii	
G2Z9E7	dipeptide epimerase	IX	Listeria ivanovii	
A0A0F5Z701	dipeptide epimerase	IX	Listeria seeligeri	
E3ZLG1	dipeptide epimerase	IX	Listeria seeligeri	
A0A0F3KG52	dipeptide epimerase	IX	Luteibacter yeojuensis	
A0A0A3I5A4	dipeptide epimerase	IX	Lysinibacillus manganicus	
A0A0A3J5M8	dipeptide epimerase	IX	Lysinibacillus massiliensis	
A0A0A3ILK5	dipeptide epimerase	IX	Lysinibacillus odysseyi	
A0A0A3HPC6	dipeptide epimerase	IX	Lysinibacillus sinduriensis	
R7Z9M6	dipeptide epimerase	IX	Lysinibacillus sphaericus	
X2GKR7	dipeptide epimerase	IX	Lysinibacillus varians	
A0A0A0EXI4	dipeptide epimerase	IX	Lysobacter arseniciresistens	
A0A021VXZ2	dipeptide epimerase	IX	Lysobacter capsici	
A0A0A0EXB8	dipeptide epimerase	IX	Lysobacter daejeonensis	
A0A0A0MAG9	dipeptide epimerase	IX	Lysobacter defluvii	
A0A0A2WP89	dipeptide epimerase	IX	Lysobacter dokdonensis	
A0A0D0R380	dipeptide epimerase	IX	Lysobacter sp.	
H3K4G8	dipeptide epimerase	IX	Megamonas funiformis	
D4KF80	dipeptide epimerase	IX	Megamonas hypermegale	5.5.1.1
G0VL67	dipeptide epimerase	IX	Megasphaera elsdenii	
S7HSX5	dipeptide epimerase	IX	Megasphaera sp.	
U7UIP5	dipeptide epimerase	IX	Megasphaera sp.	
V7F970	dipeptide epimerase	IX	Mesorhizobium sp.	
X6HCT9	dipeptide epimerase	IX	Mesorhizobium sp.	
D3S616	dipeptide epimerase	IX	Methanocaldococcus sp.	
H1L1P6	dipeptide epimerase	IX	Methanotorris formicicus	
Q607C7	dipeptide epimerase	IX	Methylococcus capsulatus	5.1.1.-
A0A022LCI8	dipeptide epimerase	IX	Microbacterium sp.	
A0A0D0WPL8	dipeptide epimerase	IX	Micromonospora carbonacea	
W7VMU0	dipeptide epimerase	IX	Micromonospora sp.	
H5UU59	dipeptide epimerase	IX	Mobilicoccus pelagius	
M0RRW9	dipeptide epimerase	IX	Musa acuminata	
F8CBC6	dipeptide epimerase	IX	Myxococcus fulvus	
L7U586	dipeptide epimerase	IX	Myxococcus stipitatus	
Q1DFI0	dipeptide epimerase	IX	Myxococcus xanthus	
M0A355	dipeptide epimerase	IX	Natrialba hulunbeirensis	
G8TMC8	dipeptide epimerase	IX	Niastella koreensis	5.5.1.1
D8PCE1	dipeptide epimerase	IX	Nitrospira defluvii	5.5.1.1
E9V0D8	dipeptide epimerase	IX	Nocardioidaceae bacterium	
A0A0F2G6B3	dipeptide epimerase	IX	Nocardioides luteus	
Q8EM93	dipeptide epimerase	IX	Oceanobacillus iheyensis	
A0A0A1M6B2	dipeptide epimerase	IX	Oceanobacillus oncorhynchi	
W9AH65	dipeptide epimerase	IX	Oceanobacillus picturae	
D4CR10	dipeptide epimerase	IX	Oribacterium sp.	

Table S2.6. (continuation)

UniProt	Family [3]	Cluster	Species	EC Number
U2W0W8	dipeptide epimerase	IX	Oribacterium sp.	
A2WKG1	dipeptide epimerase	IX	Oryza sativa	
Q5ZC51	dipeptide epimerase	IX	Oryza sativa	
K9TLD6	dipeptide epimerase	IX	Oscillatoria acuminata	
G4KSR9	dipeptide epimerase	IX	Oscillibacter valericigenes	5.5.1.-
E1ICB1	dipeptide epimerase	IX	Oscillochloris trichoides	
A0A0D5NNN9	dipeptide epimerase	IX	Paenibacillus beijingensis	
A0A090ZA22	dipeptide epimerase	IX	Paenibacillus macerans	5.1.1.n1
A0A081P1E7	dipeptide epimerase	IX	Paenibacillus sp.	
A0A081P3T3	dipeptide epimerase	IX	Paenibacillus sp.	
A0A0C2V4D0	dipeptide epimerase	IX	Paenibacillus sp.	
A0A0F5R5U7	dipeptide epimerase	IX	Paenibacillus sp.	
A0A089LPR7	dipeptide epimerase	IX	Paenibacillus stellifer	
S2Y106	dipeptide epimerase	IX	Paenisporosarcina sp.	
K6Y4R0	dipeptide epimerase	IX	Paraglaciecola arctica	
K7A7Q8	dipeptide epimerase	IX	Paraglaciecola psychrophila	
A0A0C1FUF7	dipeptide epimerase	IX	Pedobacter kyungheensis	
A6EGX4	dipeptide epimerase	IX	Pedobacter sp.	
W6TN55	dipeptide epimerase	IX	Pedobacter sp.	
A0A075K8D5	dipeptide epimerase	IX	Pelosinus sp.	5.5.1.1
T4HW59	dipeptide epimerase	IX	Peptoclostridium difficile	
A0A0A8U254	dipeptide epimerase	IX	Peptoclostridium difficile	5.1.1.-
A0A090I239	dipeptide epimerase	IX	Peptoniphilus sp.	
A0A090I3V0	dipeptide epimerase	IX	Peptoniphilus sp.	
J4WDC4	dipeptide epimerase	IX	Peptostreptococcaceae bacterium	
W3WLS4	dipeptide epimerase	IX	Pestalotiopsis fici	
V7CKT4	dipeptide epimerase	IX	Phaseolus vulgaris	
A9SGD4	dipeptide epimerase	IX	Physcomitrella patens	
Q6L345	dipeptide epimerase	IX	Picrophilus torridus	
A0A0A2VCR4	dipeptide epimerase	IX	Pontibacillus chungwhensis	
A0A0A2TZA9	dipeptide epimerase	IX	Pontibacillus yanchengensis	
B9I2J6	dipeptide epimerase	IX	Populus trichocarpa	5.1.1.-
A0A0C1L673	dipeptide epimerase	IX	Prauserella sp.	
Z5XR81	dipeptide epimerase	IX	Pseudoalteromonas lipolytica	
A0A0F4PPX0	dipeptide epimerase	IX	Pseudoalteromonas ruthenica	
F0SE71	dipeptide epimerase	IX	Pseudopedobacter saltans	
G7UWS4	dipeptide epimerase	IX	Pseudoxanthomonas spadix	
A0A0E3Z233	dipeptide epimerase	IX	Pseudoxanthomonas suwonensis	
E6WVW4	dipeptide epimerase	IX	Pseudoxanthomonas suwonensis	
G0ECY5	dipeptide epimerase	IX	Pyrolobus fumarii	
I1DUH7	dipeptide epimerase	IX	Rheinheimera nanhaiensis	
M4NHS2	dipeptide epimerase	IX	Rhodanobacter denitrificans	
I4VRP9	dipeptide epimerase	IX	Rhodanobacter fulvus	
I4WA29	dipeptide epimerase	IX	Rhodanobacter sp.	
I4W1W1	dipeptide epimerase	IX	Rhodanobacter spathiphylli	
I4WN72	dipeptide epimerase	IX	Rhodanobacter thiooxydans	
B9SQ11	dipeptide epimerase	IX	Ricinus communis	5.5.1.1
A7NKH0	dipeptide epimerase	IX	Roseiflexus castenholzii	
A5UXJ3	dipeptide epimerase	IX	Roseiflexus sp.	
A0A0C1GRT1	dipeptide epimerase	IX	Ruegeria sp.	
A0A0C1JEE9	dipeptide epimerase	IX	Ruegeria sp.	
F8EC20	dipeptide epimerase	IX	Runella slithyformis	5.5.1.7
F8ENB3	dipeptide epimerase	IX	Runella slithyformis	5.5.1.7
G5GPH5	dipeptide epimerase	IX	Selenomonas infelix	
I0GQA2	dipeptide epimerase	IX	Selenomonas ruminantium	
J4XIB1	dipeptide epimerase	IX	Selenomonas sp.	

Table S2.6. (continuation)

UniProt	Family [3]	Cluster	Species	EC Number
L1N8V8	dipeptide epimerase	IX	Selenomonas sp.	
M1A4Q6	dipeptide epimerase	IX	Solanum tuberosum	
F2F4K7	dipeptide epimerase	IX	Solibacillus silvestris	
A9GEI3	dipeptide epimerase	IX	Sorangium cellulosum	
S4XIG9	dipeptide epimerase	IX	Sorangium cellulosum	
C5XQP6	dipeptide epimerase	IX	Sorghum bicolor	
U2HZI7	dipeptide epimerase	IX	Sphingobacterium paucimobilis	
A0A077XLU3	dipeptide epimerase	IX	Sphingobacterium sp.	
A0A088EUM1	dipeptide epimerase	IX	Sphingobacterium sp.	
A0A0B8T972	dipeptide epimerase	IX	Sphingobacterium sp.	
F4C491	dipeptide epimerase	IX	Sphingobacterium sp.	5.5.1.1
E1R131	dipeptide epimerase	IX	Spirochaeta smaragdinae	
E1R8W1	dipeptide epimerase	IX	Spirochaeta smaragdinae	
E1R995	dipeptide epimerase	IX	Spirochaeta smaragdinae	
D2QBA5	dipeptide epimerase	IX	Spirosoma linguale	
A0A098LJ33	dipeptide epimerase	IX	Sporocytophaga myxococcoides	
V6IZ72	dipeptide epimerase	IX	Sporolactobacillus laevolacticus	
F9DUF6	dipeptide epimerase	IX	Sporosarcina newyorkensis	4.2.1.6
A0A033U9T5	dipeptide epimerase	IX	Staphylococcus aureus	
A0A0D0JMV5	dipeptide epimerase	IX	Stenotrophomonas maltophilia	
B2FK18	dipeptide epimerase	IX	Stenotrophomonas maltophilia	
J7UPX8	dipeptide epimerase	IX	Stenotrophomonas maltophilia	
M5TRF0	dipeptide epimerase	IX	Stenotrophomonas maltophilia	
G0JUL9	dipeptide epimerase	IX	Stenotrophomonas maltophilia	5.5.1.7
A0A023Y818	dipeptide epimerase	IX	Stenotrophomonas rhizophila	
A0A031HCL3	dipeptide epimerase	IX	Stenotrophomonas sp.	
Q08S37	dipeptide epimerase	IX	Stigmatella aurantiaca	
A6Q9H5	dipeptide epimerase	IX	Sulfurovum sp.	5.5.1.7
A0A075LP27	dipeptide epimerase	IX	Terribacillus aidingensis	
D5X9W8	dipeptide epimerase	IX	Thermincola potens	
A1S1A2	dipeptide epimerase	IX	Thermofilum pendens	
S5ZUD1	dipeptide epimerase	IX	Thermofilum sp.	
T0LPI2	dipeptide epimerase	IX	Thermoplasmatales archaeon	
A1HLW3	dipeptide epimerase	IX	Thermosinus carboxydivorans	
F5YQR9	dipeptide epimerase	IX	Treponema primitia	
U2KI70	dipeptide epimerase	IX	Treponema socranskii	
V4XU93	dipeptide epimerase	IX	uncultured archaeon	
V4IZ61	dipeptide epimerase	IX	uncultured Desulfofustis	
A0A075JST4	dipeptide epimerase	IX	Virgibacillus sp.	
W4EPF4	dipeptide epimerase	IX	Viridibacillus arenosi	
D7SJ31	dipeptide epimerase	IX	Vitis vinifera	
A0A0D0P9E4	dipeptide epimerase	IX	Wenxinia marina	
D2UBF5	dipeptide epimerase	IX	Xanthomonas albilineans	5.5.1.7
A0A061YI44	dipeptide epimerase	IX	Xanthomonas arboricola	
A0A0A4F251	dipeptide epimerase	IX	Xanthomonas axonopodis	
A0A077SDW6	dipeptide epimerase	IX	Xanthomonas campestris	5.1.1.1
R0FE41	dipeptide epimerase	IX	Xanthomonas fragariae	
V7ZCX9	dipeptide epimerase	IX	Xanthomonas hortorum	
G7TAK7	dipeptide epimerase	IX	Xanthomonas oryzae	
A0A0A8DZR8	dipeptide epimerase	IX	Xanthomonas sacchari	
K8Z2L1	dipeptide epimerase	IX	Xanthomonas translucens	5.5.1.7
A0A0B2NBV0	dipeptide epimerase	IX	Xanthomonas vesicatoria	
C4IZH9	dipeptide epimerase	IX	Zea mays	
K7VPX9	dipeptide epimerase	IX	Zea mays	
G0LAY9	dipeptide epimerase	IX	Zobellia galactanivorans	5.5.1.1
B6G1W7	dipeptide epimerase	XI	[Clostridium] hiranonis	

Table S2.6. (continuation)

UniProt	Family [3]	Cluster	Species	EC Number
X0PGX5	dipeptide epimerase	XI		
F5JDH4	dipeptide epimerase	XI	Agrobacterium sp.	
Q94K39	dipeptide epimerase	XI	Arabidopsis thaliana	
A0A087HAL3	dipeptide epimerase	XI	Arabis alpina	
A7Z3S0	dipeptide epimerase	XI	Bacillus methylotrophicus	
G9QIK4	dipeptide epimerase	XI	Bacillus smithii	
D2EG65	dipeptide epimerase	XI	Candidatus Parvarchaeum	
R0HLX1	dipeptide epimerase	XI	Capsella rubella	
C5ENH0	dipeptide epimerase	XI	Clostridiales bacterium	
R6Z0D8	dipeptide epimerase	XI	Clostridium sp.	
E3PU99	dipeptide epimerase	XI	Clostridium sticklandii	
D4JBB1	dipeptide epimerase	XI	Coprococcus catus	
A0A099J1S6	dipeptide epimerase	XI	Cryobacterium roopkundense	
A0A081GNV5	dipeptide epimerase	XI	Cyanobium sp.	
Q11T61	dipeptide epimerase	XI	Cytophaga hutchinsonii	5.1.1.-
S7TRI6	dipeptide epimerase	XI	Desulfococcus multivorans	
P0CC06	dipeptide epimerase	XI	Dictyostelium discoideum	5.-.-.-
F4AXY5	dipeptide epimerase	XI	Dokdonia sp.	
B0G1K3	dipeptide epimerase	XI	Dorea formicigenerans	
C9ACM8	dipeptide epimerase	XI	Enterococcus casseliflavus	
Q834W6	dipeptide epimerase	XI	Enterococcus faecalis	5.1.1.-
R5C8K0	dipeptide epimerase	XI	Firmicutes bacterium	
X0M461	dipeptide epimerase	XI	Fusarium oxysporum	
B3E4C1	dipeptide epimerase	XI	Geobacter lovleyi	
U5QEX5	dipeptide epimerase	XI	Gloeobacter kilaueensis	
M0HHI3	dipeptide epimerase	XI	Haloferax elongans	
J3JDL6	dipeptide epimerase	XI	Halogranum salarium	
U1QRY8	dipeptide epimerase	XI	halophilic archaeon	
W0JX68	dipeptide epimerase	XI	Halostagnicola larsenii	
K2KAL2	dipeptide epimerase	XI	Idiomarina xiamenensis	
W9GB89	dipeptide epimerase	XI	Intrasporangium oryzae	
A0A068HC71	dipeptide epimerase	XI	Klebsiella oxytoca	
B1L5F2	dipeptide epimerase	XI	Korarchaeum cryptofilum	
D2Q355	dipeptide epimerase	XI	Kribbella flavida	
C2D5B0	dipeptide epimerase	XI	Lactobacillus brevis	
C0WPJ1	dipeptide epimerase	XI	Lactobacillus buchneri	5.5.1.1
H1LKW8	dipeptide epimerase	XI	Lactobacillus kisonensis	
S4PN30	dipeptide epimerase	XI	Lactobacillus otakiensis	
G9ZN92	dipeptide epimerase	XI	Lactobacillus parafarraginis	
C2K0Z4	dipeptide epimerase	XI	Lactobacillus rhamnosus	5.5.1.1
Q5WV92	dipeptide epimerase	XI	Legionella pneumophila	
H1GEX8	dipeptide epimerase	XI	Listeria innocua	
Q92FA9	dipeptide epimerase	XI	Listeria innocua	
A0AEX1	dipeptide epimerase	XI	Listeria welshimeri	
T5KQX7	dipeptide epimerase	XI	Microbacterium maritypicum	
D9T567	dipeptide epimerase	XI	Micromonospora aurantiaca	
I0L8G5	dipeptide epimerase	XI	Micromonospora lupini	
C4RKI3	dipeptide epimerase	XI	Micromonospora sp.	
F4XVU6	dipeptide epimerase	XI	Moorea producens	
H1Y0V2	dipeptide epimerase	XI	Mucilaginibacter paludis	
L9VP64	dipeptide epimerase	XI	Natronorubrum tibetense	
R6T3Q5	dipeptide epimerase	XI	Oscillibacter sp.	
C6XT95	dipeptide epimerase	XI	Pedobacter heparinus	
U3V702	dipeptide epimerase	XI	Peptoclostridium difficile	
U3XS51	dipeptide epimerase	XI	Peptoclostridium difficile	
V9HK71	dipeptide epimerase	XI	Peptostreptococcaceae bacterium	

Table S2.6. (continuation)

UniProt	Family [3]	Cluster	Species	EC Number
A9BJU2	dipeptide epimerase	XI	Petrotoga mobilis	
M5WUB5	dipeptide epimerase	XI	Prunus persica	
A0A0A1YFW6	dipeptide epimerase	XI	Pseudomonas taeanensis	
D1Y221	dipeptide epimerase	XI	Pyramidobacter piscolens	
A0A0A0HLU8	dipeptide epimerase	XI	Roseovarius mucosus	
K2GG54	dipeptide epimerase	XI	Salimicrobium jeotgali	
C4V5F8	dipeptide epimerase	XI	Selenomonas flueggei	5.5.1.1
E0P2T3	dipeptide epimerase	XI	Selenomonas sp.	5.1.2.2
K4KHY6	dipeptide epimerase	XI	Simiduia agarivorans	
D7VGU0	dipeptide epimerase	XI	Sphingobacterium spiritivorum	
D3PXT5	dipeptide epimerase	XI	Stackebrandtia nassauensis	
H1HV81	dipeptide epimerase	XI	Stomatobaculum longum	
D2SNE4	dipeptide epimerase	XI	Streptomyces fradiae	
G9RQ05	dipeptide epimerase	XI	Subdoligranulum sp.	
G9RYM6	dipeptide epimerase	XI	Subdoligranulum sp.	
A3Z0E6	dipeptide epimerase	XI	Synechococcus sp.	
A3ZA87	dipeptide epimerase	XI	Synechococcus sp.	
A4CW89	dipeptide epimerase	XI	Synechococcus sp.	
A5GML5	dipeptide epimerase	XI	Synechococcus sp.	
Q05X17	dipeptide epimerase	XI	Synechococcus sp.	
R7RQC0	dipeptide epimerase	XI	Thermobrachium celere	
V4X7P9	dipeptide epimerase	XI	uncultured archaeon	
K2AJL7	dipeptide epimerase	XI	uncultured bacterium	
K2D5W1	dipeptide epimerase	XI	uncultured bacterium	
L7HIC9	dipeptide epimerase	XI	Xanthomonas translucens	
V9VZQ5	dipeptide epimerase	XII	Leisingera methylohalidivorans	
A0A0B4DXG2	dipeptide epimerase	XII	Leisingera sp.	
W8HII9	dipeptide epimerase	XII	Rhodococcus opacus	
C0C4C9	enolase	XI	[Clostridium] hylemonae	4.2.1.11
B1C0G0	enolase	XI	[Clostridium] spiroforme	4.2.1.11
A0A0B8V700	enolase	XI		
B4N7J1	enolase	XI		
D3L489	enolase	XI		
W1Q478	enolase	XI	Abiotrophia defectiva	4.2.1.11
L7R2S3	enolase	XI	Acanthopteroctetes unifascia	
C7RSQ0	enolase	XI	Accumulibacter phosphatis	4.2.1.11
U2UPE2	enolase	XI	Acidaminococcus sp.	4.2.1.11
F4B4A6	enolase	XI	Acidianus hospitalis	4.2.1.11
D9Q1Q0	enolase	XI	Acidilobus saccharovorans	4.2.1.11
A1W4R1	enolase	XI	Acidovorax sp.	4.2.1.11
A0A086T0E7	enolase	XI	Acremonium chrysogenum	
S2WNT0	enolase	XI	Actinomyces europaeus	4.2.1.11
D4YHL6	enolase	XI	Aerococcus viridans	4.2.1.11
E0MK70	enolase	XI	Ahrensia sp.	4.2.1.11
B2URC4	enolase	XI	Akkermansia muciniphila	4.2.1.11
F0VZ95	enolase	XI	Albugo laibachii	
A7XZI0	enolase	XI	Aleuroglyphus ovatus	
B7DUE0	enolase	XI	Alicyclobacillus acidocaldarius	4.2.1.11
Q0A7K4	enolase	XI	Alkalilimnicola ehrlichii	4.2.1.11
Q43321	enolase	XI	Alnus glutinosa	4.2.1.11
A8TL63	enolase	XI	alpha proteobacterium	4.2.1.11
A8U2W2	enolase	XI	alpha proteobacterium	4.2.1.11
Q9HDT3	enolase	XI	Alternaria alternata	4.2.1.11
G3MM94	enolase	XI	Amblyomma maculatum	
W1PE11	enolase	XI	Amborella trichopoda	
Q9DDH5	enolase	XI	Amia calva	

Table S2.6. (continuation)

UniProt	Family [3]	Cluster	Species	EC Number
Q9DDH6	enolase	XI	Amia calva	
D5EDU2	enolase	XI	Aminobacterium colombiense	4.2.1.11
B3EUH8	enolase	XI	Amoebophilus asiaticus	4.2.1.11
G0FRR8	enolase	XI	Amycolatopsis mediterranei	
B1C826	enolase	XI	Anaerofustis stercorihominis	4.2.1.11
A7HCC4	enolase	XI	Anaeromyxobacter sp.	4.2.1.11
R5XWQ7	enolase	XI	Anaerotruncus sp.	4.2.1.11
C9YSH7	enolase	XI	Aphidius ervi	
Q5WQM3	enolase	XI	Apodachlya brachynema	
Q5WQM4	enolase	XI	Apodachlya brachynema	
O66778	enolase	XI	Aquifex aeolicus	4.2.1.11
D7BKN5	enolase	XI	Arcanobacterium haemolyticum	4.2.1.11
D2RG40	enolase	XI	Archaeoglobus profundus	4.2.1.11
D5V7I4	enolase	XI	Arcobacter nitrofigilis	4.2.1.11
Q6QWP8	enolase	XI	Artemia sp.	
M7MWX0	enolase	XI	Arthrobacter gangotriensis	4.2.1.11
G1X7I2	enolase	XI	Arthrobotrys oligospora	
B5W4A4	enolase	XI	Arthrospira maxima	4.2.1.11
Q756H2	enolase	XI	Ashbya gossypii	4.2.1.11
A8DU75	enolase	XI	Asterias rubens	
A0A074VF06	enolase	XI	Aureobasidium melanogenum	
F0XW81	enolase	XI	Aureococcus anophagefferens	
F0Y5L6	enolase	XI	Aureococcus anophagefferens	
L7R1F6	enolase	XI	Azaleodes sp.	
A8IBF3	enolase	XI	Azorhizobium caulinodans	4.2.1.11
A7AP71	enolase	XI	Babesia bovis	4.2.1.11
I7IS74	enolase	XI	Babesia microti	
Q89Z05	enolase	XI	Bacteroides thetaiotaomicron	4.2.1.11
D7JGP7	enolase	XI	Bacteroidetes oral	4.2.1.11
A1US94	enolase	XI	Bartonella bacilliformis	4.2.1.11
A9IS50	enolase	XI	Bartonella tribocorum	4.2.1.11
A0A0Y4	enolase	XI	Baryscapus sp.	
F4P088	enolase	XI	Batrachochytrium dendrobatidis	
B8DTI9	enolase	XI	Bifidobacterium animalis	4.2.1.11
K4ILT1	enolase	XI	Bifidobacterium asteroides	4.2.1.11
A0N0G8	enolase	XI	Blastocladiella emersonii	4.2.1.11
D8LY73	enolase	XI	Blastocystis hominis	
A3ZQA8	enolase	XI	Blastopirellula marina	4.2.1.11
Q493N5	enolase	XI	Blochmannia pennsylvanicus	4.2.1.11
B5RLS2	enolase	XI	Borrelia duttonii	4.2.1.11
A0A067MVI4	enolase	XI	Botryobasidium botryosum	
C8KI27	enolase	XI	Brachionus plicatilis	
C7MC78	enolase	XI	Brachybacterium faecium	4.2.1.11
J9URW4	enolase	XI	Brachyspira pilosicoli	4.2.1.11
Q89KV6	enolase	XI	Bradyrhizobium diazoefficiens	4.2.1.11
C3XQ23	enolase	XI	Branchiostoma floridae	
C0Z6L3	enolase	XI	Brevibacillus brevis	4.2.1.11
D4YQX1	enolase	XI	Brevibacterium mcbrellneri	4.2.1.11
Q8K9E0	enolase	XI	Buchnera aphidicola	4.2.1.11
D9Y752	enolase	XI	Burkholderiales bacterium	4.2.1.11
F5L4L8	enolase	XI	Caldalkalibacillus thermarum	4.2.1.11
A8MAB4	enolase	XI	Caldivirga maquilingensis	4.2.1.11
Q6QWP6	enolase	XI	Callinectes sapidus	
A7ZFN1	enolase	XI	Campylobacter concisus	4.2.1.11
A0RMH2	enolase	XI	Campylobacter fetus	4.2.1.11
A7I455	enolase	XI	Campylobacter hominis	4.2.1.11

Table S2.6. (continuation)

UniProt	Family [3]	Cluster	Species	EC Number
C6RCM4	enolase	XI	Campylobacter showae	4.2.1.11
E6N505	enolase	XI	Candidatus Caldiarchaeum	4.2.1.11
U6B7J7	enolase	XI	Candidatus Liberibacter	4.2.1.11
D5MHU5	enolase	XI	Candidatus Methylomirabilis	4.2.1.11
B6BPU2	enolase	XI	Candidatus Pelagibacter	4.2.1.11
E0WT39	enolase	XI	Candidatus Regiella	4.2.1.11
A0A0D2WTH4	enolase	XI	Capsaspora owczarzaki	
B0SYX8	enolase	XI	Caulobacter sp.	4.2.1.11
Q6QWP3	enolase	XI	Centruroides sp.	
A8DU78	enolase	XI	Cerebratulus sp.	
Q9DDG8	enolase	XI	Chiloscyllium punctatum	
Q9DDG9	enolase	XI	Chiloscyllium punctatum	
C7PNW3	enolase	XI	Chitinophaga pinensis	4.2.1.11
Q8KB35	enolase	XI	Chlorobium tepidum	4.2.1.11
A9WCM4	enolase	XI	Chloroflexus aurantiacus	4.2.1.11
R7Q6Y6	enolase	XI	Chondrus crispus	
V4UV38	enolase	XI	Citrus clementina	4.2.1.11
A0A067FQX5	enolase	XI	Citrus sinensis	4.2.1.11
Q97L52	enolase	XI	Clostridium acetobutylicum	4.2.1.11
A6LR09	enolase	XI	Clostridium beijerinckii	4.2.1.11
B1KTJ8	enolase	XI	Clostridium botulinum	4.2.1.11
B2TPW4	enolase	XI	Clostridium botulinum	4.2.1.11
G8LYV2	enolase	XI	Clostridium clariflavum	4.2.1.11
A0PYP4	enolase	XI	Clostridium novyi	4.2.1.11
R9BWK9	enolase	XI	Clostridium sartagoforme	4.2.1.11
R5IZA4	enolase	XI	Clostridium sp.	4.2.1.11
R5NSD2	enolase	XI	Clostridium sp.	4.2.1.11
R5T6P6	enolase	XI	Clostridium sp.	4.2.1.11
R5TKN3	enolase	XI	Clostridium sp.	4.2.1.11
R6APL4	enolase	XI	Clostridium sp.	4.2.1.11
R6D9D7	enolase	XI	Clostridium sp.	4.2.1.11
R6P9W3	enolase	XI	Clostridium sp.	4.2.1.11
R6Q714	enolase	XI	Clostridium sp.	4.2.1.11
R6QVU2	enolase	XI	Clostridium sp.	4.2.1.11
R6RSH0	enolase	XI	Clostridium sp.	4.2.1.11
R6UK57	enolase	XI	Clostridium sp.	4.2.1.11
R7L6V6	enolase	XI	Clostridium sp.	4.2.1.11
R7M3V7	enolase	XI	Clostridium sp.	4.2.1.11
R7MHC5	enolase	XI	Clostridium sp.	4.2.1.11
E3PWF7	enolase	XI	Clostridium sticklandii	4.2.1.11
Q96VP4	enolase	XI	Cochliobolus lunatus	4.2.1.11
Q95WB0	enolase	XI	Colpidium aqueous	
A4AAB8	enolase	XI	Congregibacter litoralis	4.2.1.11
E7GDB0	enolase	XI	Coprobacillus sp.	4.2.1.11
H1AK31	enolase	XI	Coprobacillus sp.	4.2.1.11
R5VG82	enolase	XI	Coprobacillus sp.	4.2.1.11
R6LBI1	enolase	XI	Coprococcus comes	4.2.1.11
B5Y9E0	enolase	XI	Coprothermobacter proteolyticus	4.2.1.11
R4V1A4	enolase	XI	Coptotermes formosanus	
L7R758	enolase	XI	Coptotriche malifoliella	
D5ENP3	enolase	XI	Coraliomargarita akajimensis	4.2.1.11
F2N705	enolase	XI	Coriobacterium glomerans	4.2.1.11
A4QCV0	enolase	XI	Corynebacterium glutamicum	4.2.1.11
M1NR44	enolase	XI	Corynebacterium halotolerans	4.2.1.11
Q4JU51	enolase	XI	Corynebacterium jeikeium	4.2.1.11
C4LHL7	enolase	XI	Corynebacterium kroppenstedtii	4.2.1.11

Table S2.6. (continuation)

UniProt	Family [3]	Cluster	Species	EC Number
C0XRQ4	enolase	XI	Corynebacterium lipophiloflavum	4.2.1.11
B6J4X4	enolase	XI	Coxiella burnetii	4.2.1.11
T2IH19	enolase	XI	Crocosphaera watsonii	4.2.1.11
B6A9M6	enolase	XI	Cryptosporidium muris	4.2.1.11
O74286	enolase	XI	Cunninghamella elegans	4.2.1.11
Q1LP64	enolase	XI	Cupriavidus metallidurans	4.2.1.11
B5IQP2	enolase	XI	Cyanobium sp.	4.2.1.11
B1X314	enolase	XI	Cyanothece sp.	4.2.1.11
Q11QE1	enolase	XI	Cytophaga hutchinsonii	4.2.1.11
M5GDM4	enolase	XI	Dacryopinax sp.	
G6D980	enolase	XI	Danaus plexippus	
Q3B7R7	enolase	XI	Danio rerio	
Q6QWP7	enolase	XI	Daphnia magna	
E9GAM4	enolase	XI	Daphnia pulex	4.2.1.11
Q47DI1	enolase	XI	Dechloromonas aromatica	4.2.1.11
D8K0N5	enolase	XI	Dehalogenimonas lykanthroporepellens	4.2.1.11
Q1J2H6	enolase	XI	Deinococcus geothermalis	4.2.1.11
Q9RR60	enolase	XI	Deinococcus radiodurans	4.2.1.11
N6UJY0	enolase	XI	Dendroctonus ponderosae	
E3BCW4	enolase	XI	Dermacoccus sp.	4.2.1.11
S7TZR4	enolase	XI	Desulfococcus multivorans	4.2.1.11
D6SPG5	enolase	XI	Desulfonatronospira thiodismutans	4.2.1.11
E8RA16	enolase	XI	Desulfurococcus mucosus	4.2.1.11
C0GFB6	enolase	XI	Dethiobacter alkaliphilus	4.2.1.11
D2Z8X7	enolase	XI	Dethiosulfovibrio peptidovorans	4.2.1.11
Q54RK5	enolase	XI	Dictyostelium discoideum	4.2.1.11
Q55F83	enolase	XI	Dictyostelium discoideum	4.2.1.11
F4PJ27	enolase	XI	Dictyostelium fasciculatum	
Q6QWQ1	enolase	XI	Diplopoda sp.	
R9NDQ1	enolase	XI	Dorea sp.	4.2.1.11
Q56RP1	enolase	XI	Drosophila affinis	
Q967M9	enolase	XI	Dryocoetoides cristatus	
C6W200	enolase	XI	Dyadobacter fermentans	4.2.1.11
A8DU76	enolase	XI	Echiurus echiurus	
D7FYQ9	enolase	XI	Ectocarpus siliculosus	4.2.1.11
L7R6I3	enolase	XI	Ectoedemia hypericella	
J9D056	enolase	XI	Edhazardia aedis	
U6GQB8	enolase	XI	Eimeria acervulina	
Q967Y8	enolase	XI	Eimeria tenella	4.2.1.11
L7R352	enolase	XI	Elhamma australasiae	
Q8SUA4	enolase	XI	Encephalitozoon cuniculi	
F8V7K3	enolase	XI	Encephalitozoon hellem	
F8V7I6	enolase	XI	Encephalitozoon romaleae	
A0A075MLH4	enolase	XI	endosymbiont of	4.2.1.11
C8PXN0	enolase	XI	Enhydrobacter aerosaccus	4.2.1.11
R2Q666	enolase	XI	Enterococcus asini	4.2.1.11
R2SQK6	enolase	XI	Enterococcus pallens	4.2.1.11
L7R371	enolase	XI	Enteucha acetosae	
Q9U5F7	enolase	XI	Eptatretus burgeri	
L7R3P7	enolase	XI	Eriocraniidae gen.	
F5WUF5	enolase	XI	Erysipelothrix rhusiopathiae	4.2.1.11
Q2NAQ1	enolase	XI	Erythrobacter litoralis	4.2.1.11
V1D7M6	enolase	XI	Eubacterium brachy	4.2.1.11
R5LHN4	enolase	XI	Eubacterium sp.	4.2.1.11
Q9LEK7	enolase	XI	Euglena gracilis	4.2.1.11
H9ZZG8	enolase	XI	Fervidicoccus fontis	4.2.1.11

Table S2.6. (continuation)

UniProt	Family [3]	Cluster	Species	EC Number
D6GSK6	enolase	XI	Filifactor alocis	4.2.1.11
B0S1G7	enolase	XI	Finegoldia magna	4.2.1.11
R5HDI1	enolase	XI	Firmicutes bacterium	4.2.1.11
C0BGE7	enolase	XI	Flavobacteria bacterium	4.2.1.11
C6X1X1	enolase	XI	Flavobacteriaceae bacterium	4.2.1.11
Q8RI55	enolase	XI	Fusobacterium nucleatum	4.2.1.11
K2JMJ2	enolase	XI	Gallaecimonas xiamenensis	4.2.1.11
D9SIP6	enolase	XI	Gallionella capsiferriformans	4.2.1.11
Q7NIR1	enolase	XI	Gloeobacter violaceus	4.2.1.11
Q0BSX3	enolase	XI	Granulibacter bethesdensis	4.2.1.11
D0BNZ7	enolase	XI	Granulicatella elegans	4.2.1.11
F0X9D1	enolase	XI	Grosmannia clavigera	
L1I6R1	enolase	XI	Guillardia theta	
L1JIB0	enolase	XI	Guillardia theta	
Q2SKX0	enolase	XI	Hahella chejuensis	4.2.1.11
D8J320	enolase	XI	Halalkalicoccus jeotgali	4.2.1.11
U2YU84	enolase	XI	Halarchaeum acidiphilum	4.2.1.11
P29201	enolase	XI	Haloarcula marismortui	4.2.1.11
A0A0B5GPH0	enolase	XI	Haloarcula sp.	4.2.1.11
B0R4Y8	enolase	XI	Halobacterium salinarum	4.2.1.11
M0MRT4	enolase	XI	Halococcus saccharolyticus	4.2.1.11
Q18G62	enolase	XI	Haloquadratum walsbyi	4.2.1.11
H0AC70	enolase	XI	Haloredivivus sp.	4.2.1.11
C7NMY1	enolase	XI	Halorhabdus utahensis	4.2.1.11
D0KYJ3	enolase	XI	Halothiobacillus neapolitanus	4.2.1.11
D0KZD5	enolase	XI	Halothiobacillus neapolitanus	4.2.1.11
C4K4K1	enolase	XI	Hamiltonella defensa	4.2.1.11
E2B9R0	enolase	XI	Harpegnathos saltator	
A0A0Y7	enolase	XI	Heimbra opaca	
L7R2Z8	enolase	XI	Heterobathmia pseuderiocrania	
Q9LEJ0	enolase	XI	Hevea brasiliensis	4.2.1.11
F2LVJ4	enolase	XI	Hippea maritima	4.2.1.11
A8USW8	enolase	XI	Hydrogenivirga sp.	4.2.1.11
D3DHY4	enolase	XI	Hydrogenobacter thermophilus	
A2BL98	enolase	XI	Hyperthermus butylicus	4.2.1.11
D8JYW9	enolase	XI	Hyphomicrobium denitrificans	4.2.1.11
Q0C0R3	enolase	XI	Hyphomonas neptunium	4.2.1.11
Q967N8	enolase	XI	Hypocryphalus mangiferae	
E3TF25	enolase	XI	Ictalurus punctatus	3.4.21.68; 3.4.21.
A3WM52	enolase	XI	Idiomarina baltica	4.2.1.11
A8AB84	enolase	XI	Ignicoccus hospitalis	4.2.1.11
M5A5I6	enolase	XI	Ilumatobacter coccineus	4.2.1.11
L7R317	enolase	XI	Imma tetrascia	
Q5WQM1	enolase	XI	Isochrysis galbana	
C7RAH9	enolase	XI	Kangiella koreensis	4.2.1.11
Q4H4A8	enolase	XI	Karenia brevis	
Q4H4B0	enolase	XI	Karenia brevis	
Q4H4B1	enolase	XI	Karenia brevis	
Q4H4B2	enolase	XI	Karenia brevis	
Q4H4A7	enolase	XI	Karenia brevis	4.2.1.11
Q4H4A9	enolase	XI	Karenia brevis	4.2.1.11
Q4H4A2	enolase	XI	Karenia mikimotoi	
Q4H4A5	enolase	XI	Karenia mikimotoi	
Q4H4A3	enolase	XI	Karenia mikimotoi	4.2.1.11
Q4H4A6	enolase	XI	Karenia mikimotoi	4.2.1.11
D5QFY1	enolase	XI	Komagataeibacter hansenii	4.2.1.11

Table S2.6. (continuation)

UniProt	Family [3]	Cluster	Species	EC Number
F2QXG9	enolase	XI	Komagataella phaffii	4.2.1.11
C5CFP3	enolase	XI	Kosmotoga olearia	4.2.1.11
D2PPA6	enolase	XI	Kribbella flavida	4.2.1.11
R9MP87	enolase	XI	Lachnospiraceae bacterium	4.2.1.11
Q5FKM6	enolase	XI	Lactobacillus acidophilus	4.2.1.11
Q03SL5	enolase	XI	Lactobacillus brevis	4.2.1.11
Q03AK4	enolase	XI	Lactobacillus casei	4.2.1.11
Q1G9S9	enolase	XI	Lactobacillus delbrueckii	4.2.1.11
D1YL58	enolase	XI	Lactobacillus gasseri	4.2.1.11
Q88YH3	enolase	XI	Lactobacillus plantarum	4.2.1.11
Q38Y18	enolase	XI	Lactobacillus sakei	4.2.1.11
Q9CHS7	enolase	XI	Lactococcus lactis	4.2.1.11
Q9CIT0	enolase	XI	Lactococcus lactis	4.2.1.11
L7R324	enolase	XI	Lampronia aenescens	
Q9DDH4	enolase	XI	Latimeria chalumnae	
G9EIV8	enolase	XI	Legionella drancourtii	4.2.1.11
A6DPS4	enolase	XI	Lentisphaera araneosa	4.2.1.11
A8DU73	enolase	XI	Leptochiton sp.	
U4QUW1	enolase	XI	Leptospirillum sp.	4.2.1.11
D0GPD9	enolase	XI	Leptotrichia goodfellowii	4.2.1.11
Q9PTX5	enolase	XI	Lethenteron reissneri	
Q9PTX6	enolase	XI	Lethenteron reissneri	
C6XFJ0	enolase	XI	Liberibacter asiaticus	4.2.1.11
Q6QWP9	enolase	XI	Limulus polyphemus	
Q6QWQ2	enolase	XI	Lithobius sp.	
V4A8P8	enolase	XI	Lottia gigantea	
B1HVS4	enolase	XI	Lysinibacillus sphaericus	4.2.1.11
A4AJC1	enolase	XI	marine actinobacterium	4.2.1.11
E4PF40	enolase	XI	Marinobacter adhaerens	4.2.1.11
A1TZ48	enolase	XI	Marinobacter hydrocarbonoclasticus	4.2.1.11
A3JL09	enolase	XI	Marinobacter sp.	4.2.1.11
C6LIZ0	enolase	XI	Marvinbryantia formatexigens	4.2.1.11
Q9U615	enolase	XI	Mastigamoeba balamuthi	4.2.1.11
Q947A2	enolase	XI	Mastocarpus papillatus	
Q947A3	enolase	XI	Mastocarpus papillatus	
D3LU65	enolase	XI	Megasphaera genomosp.	4.2.1.11
U7UH43	enolase	XI	Megasphaera sp.	4.2.1.11
D7BG30	enolase	XI	Meiothermus silvanus	4.2.1.11
Q43130	enolase	XI	Mesembryanthemum crystallinum	4.2.1.11
H0HWC0	enolase	XI	Mesorhizobium alhagi	4.2.1.11
K2RCG3	enolase	XI	Methanobacterium formicicum	4.2.1.11
F0TBB4	enolase	XI	Methanobacterium lacus	4.2.1.11
D5VRS0	enolase	XI	Methanocaldococcus infernus	4.2.1.11
N6V044	enolase	XI	Methanocaldococcus villosus	4.2.1.11
Q0W8C9	enolase	XI	Methanocella arvoryzae	4.2.1.11
D1YZ42	enolase	XI	Methanocella paludicola	4.2.1.11
D1Z1G7	enolase	XI	Methanocella paludicola	4.2.1.11
Q12VE5	enolase	XI	Methanococcoides burtonii	4.2.1.11
A3CWI5	enolase	XI	Methanoculleus marisnigri	4.2.1.11
E1RI52	enolase	XI	Methanolacinia petrolearia	4.2.1.11
G7WPS3	enolase	XI	Methanosaeta harundinacea	4.2.1.11
A0B7E8	enolase	XI	Methanosaeta thermophila	4.2.1.11
Q468E2	enolase	XI	Methanosarcina barkeri	4.2.1.11
Q2NG02	enolase	XI	Methanosphaera stadtmanae	4.2.1.11
O26149	enolase	XI	Methanothermobacter thermautotrophicus	4.2.1.11
F8ANY6	enolase	XI	Methanothermococcus okinawensis	4.2.1.11

Table S2.6. (continuation)

UniProt	Family [3]	Cluster	Species	EC Number
A9QPK3	enolase	XI	Methylacidiphilum infernorum	4.2.1.11
Q1H011	enolase	XI	Methylobacillus flagellatus	4.2.1.11
F5R8L5	enolase	XI	Methyloversatilis universalis	4.2.1.11
F5XTP5	enolase	XI	Microlunatus phosphovorus	4.2.1.11
L7R218	enolase	XI	Micropterix calthella	
A1ZJD6	enolase	XI	Microscilla marina	4.2.1.11
C9KLZ8	enolase	XI	Mitsuokella multacida	4.2.1.11
L7R6S5	enolase	XI	Mnesarchaea acuta	
E0N639	enolase	XI	Mobiluncus curtisii	4.2.1.11
D0YPE6	enolase	XI	Mobiluncus mulieris	4.2.1.11
A1BQS2	enolase	XI	Monocercomonoides sp.	
H1Y6V2	enolase	XI	Mucilaginibacter paludis	4.2.1.11
P47647	enolase	XI	Mycoplasma genitalium	4.2.1.11
R5MWV6	enolase	XI	Mycoplasma sp.	4.2.1.11
R7HE02	enolase	XI	Mycoplasma sp.	4.2.1.11
L7UBJ0	enolase	XI	Myxococcus stipitatus	4.2.1.11
Q3IQT0	enolase	XI	Natronomonas pharaonis	4.2.1.11
C7YJG0	enolase	XI	Nectria haematococca	
L7R6V1	enolase	XI	Nematopogon magna	
A7S5Z0	enolase	XI	Nematostella vectensis	
L7R3M1	enolase	XI	Nemophora sp.	
P42894	enolase	XI	Neocallimastix frontalis	4.2.1.11
Q9DDH0	enolase	XI	Neoceratodus forsteri	
Q2GD37	enolase	XI	Neorickettsia sennetsu	4.2.1.11
Q2BQW7	enolase	XI	Neptuniibacter caesariensis	4.2.1.11
Q6QWP5	enolase	XI	Nereis macrydi	
A4BS42	enolase	XI	Nitrococcus mobilis	4.2.1.11
D5BX72	enolase	XI	Nitrosococcus halophilus	4.2.1.11
Q3JCT1	enolase	XI	Nitrosococcus oceani	4.2.1.11
F9ZDS3	enolase	XI	Nitrosomonas sp.	4.2.1.11
M5DGB2	enolase	XI	Nitrosospira lacus	4.2.1.11
R7XUE5	enolase	XI	Nocardioides sp.	4.2.1.11
C4VAP2	enolase	XI	Nosema ceranae	
Q2CG63	enolase	XI	Oceanicola granulosus	4.2.1.11
Q6YQT9	enolase	XI	Onion yellows	4.2.1.11
D4P967	enolase	XI	Ornithodoros moubata	
K9TCC1	enolase	XI	Oscillatoria acuminata	4.2.1.11
Q6QWP4	enolase	XI	Ostracoda sp.	
E6WL82	enolase	XI	Pantoea sp.	4.2.1.11
A8DU80	enolase	XI	Paragordius varius	
Q95WB1	enolase	XI	Paramecium tetraurelia	
A0SNX4	enolase	XI	Paratrimastix pyriformis	
A7HXW7	enolase	XI	Parvibaculum lavamentivorans	4.2.1.11
P57975	enolase	XI	Pasteurella multocida	4.2.1.11
B1X4D4	enolase	XI	Paulinella chromatophora	
L7R394	enolase	XI	Pectinivalva sp.	
D2EHH5	enolase	XI	Pediococcus acidilactici	4.2.1.11
Q4FM37	enolase	XI	Pelagibacter ubique	4.2.1.11
A1APJ8	enolase	XI	Pelobacter propionicus	4.2.1.11
U7URH1	enolase	XI	Peptoniphilus sp.	4.2.1.11
R5IYU3	enolase	XI	Peptostreptococcus anaerobius	4.2.1.11
C5LEZ5	enolase	XI	Perkinsus marinus	
C5LK37	enolase	XI	Perkinsus marinus	
C5LGP8	enolase	XI	Perkinsus marinus	4.2.1.11
C0QRV6	enolase	XI	Persephonella marina	4.2.1.11
R6I4P6	enolase	XI	Phascolarctobacterium sp.	4.2.1.11

Table S2.6. (continuation)

UniProt	Family [3]	Cluster	Species	EC Number
A8DU77	enolase	XI	Phascolion strombus	
B4RBW1	enolase	XI	Phenylobacterium zucineum	4.2.1.11
Q6QWQ0	enolase	XI	Phormictopus sp.	
J3C442	enolase	XI	Phyllobacterium sp.	4.2.1.11
A9SGH3	enolase	XI	Physcomitrella patens	
A9SIQ6	enolase	XI	Physcomitrella patens	
W6L3B1	enolase	XI	Phytomonas sp.	
W2VML9	enolase	XI	Phytophthora parasitica	
B8LKJ8	enolase	XI	Picea sitchensis	
D5SQ52	enolase	XI	Planctopirus limnophila	4.2.1.11
A0A100	enolase	XI	Platygaster sp.	
D2WJB1	enolase	XI	Pollanisus sp.	
D3BP87	enolase	XI	Polysphondylium pallidum	
Q9NG69	enolase	XI	Polyxenus fasciculatus	
B9I2N0	enolase	XI	Populus trichocarpa	
D8E0G3	enolase	XI	Prevotella bryantii	4.2.1.11
D3IBM9	enolase	XI	Prevotella sp.	4.2.1.11
R5FPM2	enolase	XI	Prevotella sp.	4.2.1.11
A8DU79	enolase	XI	Priapulus caudatus	
Q947A0	enolase	XI	Prionitis lanceolata	
Q947A1	enolase	XI	Prionitis lanceolata	
L8N2W8	enolase	XI	Pseudanabaena biceps	4.2.1.11
U1LAT2	enolase	XI	Pseudoalteromonas spongiae	4.2.1.11
F7YXU7	enolase	XI	Pseudothermotoga thermarum	4.2.1.11
A3KBG5	enolase	XI	Pseudotrichonympha grassii	
Q1Q9K6	enolase	XI	Psychrobacter cryohalolentis	4.2.1.11
A1SSQ7	enolase	XI	Psychromonas ingrahamii	4.2.1.11
L7R3C5	enolase	XI	Ptyssoptera sp.	
E3KXK0	enolase	XI	Puccinia graminis	
A4WH06	enolase	XI	Pyrobaculum arsenaticum	4.2.1.11
A3MS45	enolase	XI	Pyrobaculum calidifontis	4.2.1.11
A1RTI8	enolase	XI	Pyrobaculum islandicum	4.2.1.11
B1YA37	enolase	XI	Pyrobaculum neutrophilum	4.2.1.11
Q98MZ3	enolase	XI	Rhizobium loti	4.2.1.11
C8S2U7	enolase	XI	Rhodobacter sp.	4.2.1.11
A3JPG7	enolase	XI	Rhodobacteraceae bacterium	4.2.1.11
Q7UIR2	enolase	XI	Rhodopirellula baltica	4.2.1.11
Q07ND9	enolase	XI	Rhodopseudomonas palustris	4.2.1.11
Q215A2	enolase	XI	Rhodopseudomonas palustris	4.2.1.11
Q2RT60	enolase	XI	Rhodospirillum rubrum	4.2.1.11
D0MJB6	enolase	XI	Rhodothermus marinus	4.2.1.11
B9S376	enolase	XI	Ricinus communis	4.2.1.11
R4WCU2	enolase	XI	Riptortus pedestris	
R7R898	enolase	XI	Roseburia sp.	4.2.1.11
F0SF81	enolase	XI	Rubinisphaera brasiliensis	4.2.1.11
A1AW20	enolase	XI	Ruthia magnifica	4.2.1.11
L7R3Y5	enolase	XI	Sabatinca zonodoxa	
B5VJJ6	enolase	XI	Saccharomyces cerevisiae	
Q21LC2	enolase	XI	Saccharophagus degradans	4.2.1.11
A4F804	enolase	XI	Saccharopolyspora erythraea	4.2.1.11
A8DU81	enolase	XI	Saccoglossus bromophenolosus	
A4X3B7	enolase	XI	Salinispora tropica	4.2.1.11
Q9DDG6	enolase	XI	Salmo trutta	
Q9DDG7	enolase	XI	Salmo trutta	
F2U843	enolase	XI	Salpingoeca rosetta	
A0A104	enolase	XI	Scelio sp.	

Table S2.6. (continuation)

UniProt	Family [3]	Cluster	Species	EC Number
A3LQD6	enolase	XI	Scheffersomyces stipitis	
G9C5D8	enolase	XI	Schistocerca gregaria	
Q27877	enolase	XI	Schistosoma mansoni	4.2.1.11
D8PM07	enolase	XI	Schizophyllum commune	
Q9NG68	enolase	XI	Scolopendra polymorpha	
E5XTF2	enolase	XI	Segniliparus rugosus	4.2.1.11
A0A106	enolase	XI	Semiotellus sp.	
D5CT27	enolase	XI	Sideroxydans lithotrophicus	4.2.1.11
E9IYB3	enolase	XI	Solenopsis invicta	
Q01YD1	enolase	XI	Solibacter usitatus	4.2.1.11
E7MNE8	enolase	XI	Solobacterium moorei	4.2.1.11
A9FA52	enolase	XI	Sorangium cellulosum	4.2.1.11
F7VSL8	enolase	XI	Sordaria macrospora	
Q1GVS8	enolase	XI	Sphingopyxis alaskensis	4.2.1.11
L7R1G0	enolase	XI	Spinulata maruga	
R4V4U6	enolase	XI	Spiribacter salinus	4.2.1.11
D2QD03	enolase	XI	Spirosoma linguale	4.2.1.11
D2QVS9	enolase	XI	Spirosoma linguale	4.2.1.11
A3DP05	enolase	XI	Staphylothermus marinus	4.2.1.11
B2FK88	enolase	XI	Stenotrophomonas maltophilia	4.2.1.11
H1HW25	enolase	XI	Stomatobaculum longum	4.2.1.11
D1AYQ7	enolase	XI	Streptobacillus moniliformis	4.2.1.11
Q04KG2	enolase	XI	Streptococcus pneumoniae	4.2.1.11
E8W3C3	enolase	XI	Streptomyces pratensis	4.2.1.11
B5H858	enolase	XI	Streptomyces pristinaespiralis	4.2.1.11
D6KEM4	enolase	XI	Streptomyces sp.	4.2.1.11
Q5IW34	enolase	XI	Streptomyces viridochromogenes	
D2B3Y9	enolase	XI	Streptosporangium roseum	4.2.1.11
Q4J920	enolase	XI	Sulfolobus acidocaldarius	4.2.1.11
Q972B6	enolase	XI	Sulfolobus tokodaii	4.2.1.11
B2V9N8	enolase	XI	Sulfurihydrogenibium sp.	4.2.1.11
A8DU74	enolase	XI	Sycon sp.	
B1XLD0	enolase	XI	Synechococcus sp.	4.2.1.11
P77972	enolase	XI	Synechocystis sp.	4.2.1.11
C5BMT5	enolase	XI	Teredinibacter turnerae	4.2.1.11
E8V1P1	enolase	XI	Terriglobus saanensis	4.2.1.11
Q95WA8	enolase	XI	Tetrahymena bergeri	
Q95WA9	enolase	XI	Tetrahymena thermophila	
I2H256	enolase	XI	Tetrapisispora blattae	
C7D732	enolase	XI	Thalassobium sp.	4.2.1.11
E9NJD0	enolase	XI	Theileria annulata	4.2.1.11
L0B147	enolase	XI	Theileria equi	4.2.1.11
D1B8F5	enolase	XI	Thermanaerovibrio acidaminovorans	4.2.1.11
D9TN99	enolase	XI	Thermoanaerobacterium thermosac.	4.2.1.11
D1CDH2	enolase	XI	Thermobaculum terrenum	4.2.1.11
B6YUB8	enolase	XI	Thermococcus onnurineus	4.2.1.11
D3SPX2	enolase	XI	Thermocrinis albus	4.2.1.11
F8ADR0	enolase	XI	Thermodesulfatator indicus	4.2.1.11
F8C2F4	enolase	XI	Thermodesulfobacterium geofontis	4.2.1.11
B5YKR8	enolase	XI	Thermodesulfovibrio yellowstonii	4.2.1.11
Q703Y8	enolase	XI	Thermoproteus tenax	4.2.1.11
D9RZ26	enolase	XI	Thermosediminibacter oceani	4.2.1.11
Q8DL40	enolase	XI	Thermosynechococcus elongatus	4.2.1.11
Q3SL43	enolase	XI	Thiobacillus denitrificans	4.2.1.11
L0GTN7	enolase	XI	Thioflavicoccus mobilis	4.2.1.11
D2WJC5	enolase	XI	Tineola bisselliella	4.2.1.11

Table S2.6. (continuation)

UniProt	Family [3]	Cluster	Species	EC Number
Q9NG67	enolase	XI	Tomocerus sp.	
A0A0B2UQW4	enolase	XI	Toxocara canis	4.2.1.11
Q9BPL7	enolase	XI	Toxoplasma gondii	4.2.1.11
Q9W6D2	enolase	XI	Trachemys scripta	
D6X009	enolase	XI	Tribolium castaneum	
D6X018	enolase	XI	Tribolium castaneum	
Q967U0	enolase	XI	Trichinella spiralis	
Q9NDF5	enolase	XI	Trypanosoma brucei	4.2.1.11
G0UVS4	enolase	XI	Trypanosoma congolense	
Q6W3C0	enolase	XI	Tuber borchii	4.2.1.11
D4W748	enolase	XI	Turicibacter sanguinis	4.2.1.11
D1JI24	enolase	XI	uncultured archaeon	4.2.1.11
K1XCI5	enolase	XI	uncultured bacterium	
K2C1I4	enolase	XI	uncultured bacterium	
D9I7J6	enolase	XI	uncultured bacterium	4.2.1.11
E3T6P2	enolase	XI	uncultured bacterium	4.2.1.11
K1XTZ0	enolase	XI	uncultured bacterium	4.2.1.11
K1YS92	enolase	XI	uncultured bacterium	4.2.1.11
K1YT30	enolase	XI	uncultured bacterium	4.2.1.11
K1ZKC4	enolase	XI	uncultured bacterium	4.2.1.11
K1ZLB3	enolase	XI	uncultured bacterium	4.2.1.11
K2BXX1	enolase	XI	uncultured bacterium	4.2.1.11
K2CCI2	enolase	XI	uncultured bacterium	4.2.1.11
K2DE11	enolase	XI	uncultured bacterium	4.2.1.11
D1KE84	enolase	XI	uncultured SUP05	4.2.1.11
A6ERK4	enolase	XI	unidentified eubacterium	4.2.1.11
L2GSH5	enolase	XI	Vavraia culicis	
B5JG80	enolase	XI	Verrucomicrobiae bacterium	4.2.1.11
H2IFF9	enolase	XI	Vibrio sp.	4.2.1.11
F8I082	enolase	XI	Weissella koreensis	4.2.1.11
L7R420	enolase	XI	Wormaldia moesta	
Q4UTP2	enolase	XI	Xanthomonas campestris	4.2.1.11
A8DU82	enolase	XI	Xenoturbella bocki	
Q6JKT2	enolase	XI	Zootermopsis parabasalian	
Q6JKT6	enolase	XI	Zootermopsis parabasalian	
S6EF90	enolase	XI	Zygosaccharomyces bailii	
C5DTW4	enolase	XI	Zygosaccharomyces rouxii	
C0D4Q2	enolase	XII	[Clostridium asparagiforme]	4.2.1.11
A0A084JMU0	enolase	XII	[Clostridium] celerecrescens	4.2.1.11
A0A078KTD7	enolase	XII	[Clostridium] cellulosi	4.2.1.11
G5HE86	enolase	XII	[Clostridium] citroniae	4.2.1.11
A0A069REU8	enolase	XII	[Clostridium] litorale	4.2.1.11
C0E907	enolase	XII	[Clostridium] methylpentosum	4.2.1.11
B0NKH1	enolase	XII	[Clostridium] scindens	4.2.1.11
B1C199	enolase	XII	[Clostridium] spiroforme	4.2.1.11
E7GKU8	enolase	XII	[Clostridium] symbiosum	4.2.1.11
M1YXF4	enolase	XII	[Clostridium] ultunense	4.2.1.11
I5AQF9	enolase	XII	[Eubacterium] cellulosolvens	4.2.1.11
C0EVL2	enolase	XII	[Eubacterium] hallii	4.2.1.11
D8FTK9	enolase	XII	[Oscillatoria] sp.	4.2.1.11
A0A052IRU4	enolase	XII		
A0A068VT36	enolase	XII		
A0A068XIY8	enolase	XII		
A0A089PLB1	enolase	XII		
A0A094RD92	enolase	XII		
A0A0A5IX75	enolase	XII		

Table S2.6. (continuation)

UniProt	Family [3]	Cluster	Species	EC Number
A0A0B5FKL4	enolase	XII		
A0A0B7HZ84	enolase	XII		
A0A0D1K5Q7	enolase	XII		
T0IKB5	enolase	XII		
U4TLC4	enolase	XII		
X7HZA7	enolase	XII		
L8GRZ9	enolase	XII	Acanthamoeba castellanii	
B0C9F0	enolase	XII	Acaryochloris marina	4.2.1.11
A0A0D6MWD1	enolase	XII	Acetobacter aceti	4.2.1.11
H1UEA1	enolase	XII	Acetobacter pasteurianus	4.2.1.11
R5QP51	enolase	XII	Acetobacter sp.	4.2.1.11
A0A095AX04	enolase	XII	Acetobacter tropicalis	4.2.1.11
H6LJN1	enolase	XII	Acetobacterium woodii	4.2.1.11
D9QU82	enolase	XII	Acetohalobium arabaticum	4.2.1.11
F7NJQ7	enolase	XII	Acetonema longum	4.2.1.11
U4KP04	enolase	XII	Acholeplasma brassicae	4.2.1.11
A9NF93	enolase	XII	Acholeplasma laidlawii	4.2.1.11
A0A061AB87	enolase	XII	Acholeplasma oculi	4.2.1.11
U4KL30	enolase	XII	Acholeplasma palmae	4.2.1.11
R5HVY1	enolase	XII	Acholeplasma sp.	4.2.1.11
H0F840	enolase	XII	Achromobacter arsenitoxydans	4.2.1.11
J4PCH8	enolase	XII	Achromobacter piechaudii	4.2.1.11
R7M3I6	enolase	XII	Acidaminococcus sp.	4.2.1.11
A0A094MTV9	enolase	XII	Acidihalobacter prosperus	4.2.1.11
C7M0K3	enolase	XII	Acidimicrobium ferrooxidans	4.2.1.11
A5FUW3	enolase	XII	Acidiphilium cryptum	4.2.1.11
A0A060A020	enolase	XII	Acidithiobacillus caldus	4.2.1.11
A0A060USC7	enolase	XII	Acidithiobacillus ferrivorans	4.2.1.11
B7J6R4	enolase	XII	Acidithiobacillus ferrooxidans	4.2.1.11
C1F9E6	enolase	XII	Acidobacterium capsulatum	4.2.1.11
K5ZL57	enolase	XII	Acidocella sp.	4.2.1.11
A0LW71	enolase	XII	Acidothermus cellulolyticus	4.2.1.11
J0UAX2	enolase	XII	Acidovorax sp.	4.2.1.11
B5ICN2	enolase	XII	Aciduliprofundum boonei	4.2.1.11
L0HK45	enolase	XII	Aciduliprofundum sp.	4.2.1.11
B7H227	enolase	XII	Acinetobacter baumannii	4.2.1.11
R5SJL1	enolase	XII	Acinetobacter sp.	4.2.1.11
A0A058ZGM0	enolase	XII	Actibacterium atlanticum	4.2.1.11
A0A037ZKR6	enolase	XII	Actibacterium mucosum	4.2.1.11
A0A094PNB3	enolase	XII	actinobacterium acAcidi	4.2.1.11
A0A094PMH9	enolase	XII	actinobacterium acAMD-2	4.2.1.11
A0A094Q450	enolase	XII	actinobacterium acAMD-5	4.2.1.11
A0A094P7Y7	enolase	XII	actinobacterium acIB-AMD-7	4.2.1.11
A0A094P8Q6	enolase	XII	actinobacterium acIB-AMD-7	4.2.1.11
A0A094RNT1	enolase	XII	actinobacterium acMicro-1	4.2.1.11
A0A094P1B1	enolase	XII	actinobacterium acMicro-4	4.2.1.11
J9H7E6	enolase	XII	actinobacterium SCGC	4.2.1.11
J9HN07	enolase	XII	actinobacterium SCGC	4.2.1.11
K9ECK4	enolase	XII	Actinobaculum massiliense	4.2.1.11
W7J7W4	enolase	XII	Actinokineospora spheciospongiae	
G9PHR8	enolase	XII	Actinomyces graevenitzii	4.2.1.11
K0YR93	enolase	XII	Actinomyces neuii	4.2.1.11
D0WQ13	enolase	XII	Actinomyces sp.	4.2.1.11
L1PI65	enolase	XII	Actinomyces sp.	4.2.1.11
S2ZIU5	enolase	XII	Actinomyces sp.	4.2.1.11
U5W1C5	enolase	XII	Actinoplanes friuliensis	4.2.1.11

Table S2.6. (continuation)

UniProt	Family [3]	Cluster	Species	EC Number
G8SDZ3	enolase	XII	Actinoplanes sp.	4.2.1.11
A0A0A6ULF3	enolase	XII	Actinoplanes utahensis	4.2.1.11
A0A0A6UVQ2	enolase	XII	Actinoplanes utahensis	4.2.1.11
A0A099D848	enolase	XII	Actinopolyspora erythraea	4.2.1.11
C6WP03	enolase	XII	Actinosynnema mirum	4.2.1.11
A0A021VPW6	enolase	XII	Actinotalea ferrariae	4.2.1.11
S2VHX4	enolase	XII	Actinotignum schaalii	4.2.1.11
S6C122	enolase	XII	Adlercreutzia equolifaciens	4.2.1.11
I3YWP2	enolase	XII	Aequorivita sublithincola	4.2.1.11
F2I5C0	enolase	XII	Aerococcus urinae	4.2.1.11
E2SBK6	enolase	XII	Aeromicrobium marinum	4.2.1.11
U3TBU6	enolase	XII	Aeropyrum camini	4.2.1.11
Q9Y927	enolase	XII	Aeropyrum pernix	4.2.1.11
W3REU8	enolase	XII	Afipia sp.	4.2.1.11
W3RLL4	enolase	XII	Afipia sp.	4.2.1.11
K5Y7K9	enolase	XII	Agaricus bisporus	
L7R1D5	enolase	XII	Agathiphaga queenslandensis	
U1LR64	enolase	XII	Agrococcus pavilionensis	4.2.1.11
R6JXD9	enolase	XII	Akkermansia muciniphila	4.2.1.11
F0WLM9	enolase	XII	Albugo laibachii	
A0A0A2MZY5	enolase	XII	Alcaligenes faecalis	4.2.1.11
A0A0D6HTT8	enolase	XII	Alcaligenes xylosoxydans	4.2.1.11
Q0VQD6	enolase	XII	Alcanivorax borkumensis	4.2.1.11
A0A0B4XPW9	enolase	XII	Alcanivorax pacificus	4.2.1.11
U7HXA2	enolase	XII	Alcanivorax sp.	4.2.1.11
A0A090WZH5	enolase	XII	Algibacter lectus	4.2.1.11
A3HXC6	enolase	XII	Algoriphagus machipongonensis	4.2.1.11
J9H8Y5	enolase	XII	Alicyclobacillus hesperidum	4.2.1.11
I8U7N2	enolase	XII	Alishewanella agri	4.2.1.11
G5H574	enolase	XII	Alistipes indistinctus	4.2.1.11
A0A0B0BVD3	enolase	XII	Alistipes inops	4.2.1.11
R7JNG2	enolase	XII	Alistipes putredinis	4.2.1.11
R6VTP9	enolase	XII	Alistipes sp.	4.2.1.11
R6X5L6	enolase	XII	Alistipes sp.	4.2.1.11
A0A011Q5P0	enolase	XII	Alkalibacterium sp.	4.2.1.11
A6TU30	enolase	XII	Alkaliphilus metalliredigens	4.2.1.11
A8MFY2	enolase	XII	Alkaliphilus oremlandii	4.2.1.11
D3RN66	enolase	XII	Allochromatium vinosum	4.2.1.11
K9E7J2	enolase	XII	Alloiococcus otitis	4.2.1.11
G5GAZ8	enolase	XII	Alloprevotella rava	4.2.1.11
C9LG18	enolase	XII	Alloprevotella tannerae	4.2.1.11
A0A061QF35	enolase	XII	alpha proteobacterium	4.2.1.11
D0RP36	enolase	XII	alpha proteobacterium	4.2.1.11
J9DJT1	enolase	XII	alpha proteobacterium	4.2.1.11
J9YV18	enolase	XII	alpha proteobacterium	4.2.1.11
J9Z1U8	enolase	XII	alpha proteobacterium	4.2.1.11
M2T804	enolase	XII	alpha proteobacterium	4.2.1.11
B4RVU5	enolase	XII	Alteromonas mediterranea	4.2.1.11
A0A0C2XP91	enolase	XII	Amanita muscaria	
E3CXK1	enolase	XII	Aminomonas paucivorans	4.2.1.11
C9R8S9	enolase	XII	Ammonifex degensii	4.2.1.11
K0J525	enolase	XII	Amphibacillus xylanus	4.2.1.11
I1GHA4	enolase	XII	Amphimedon queenslandica	
R1I219	enolase	XII	Amycolatopsis vancoresmycina	4.2.1.11
F6EQN8	enolase	XII	Amycolicicoccus subflavus	4.2.1.11
I4BUY4	enolase	XII	Anaerobaculum mobile	4.2.1.11

Table S2.6. (continuation)

UniProt	Family [3]	Cluster	Species	EC Number
C7RH35	enolase	XII	Anaerococcus prevotii	4.2.1.11
E8MZM0	enolase	XII	Anaerolinea thermophila	4.2.1.11
B4UDM8	enolase	XII	Anaeromyxobacter sp.	4.2.1.11
B0MCZ0	enolase	XII	Anaerostipes caccae	4.2.1.11
E5VQN4	enolase	XII	Anaerostipes sp.	4.2.1.11
E5VYZ4	enolase	XII	Anaerostipes sp.	4.2.1.11
B0P761	enolase	XII	Anaerotruncus colihominis	4.2.1.11
R7EMX6	enolase	XII	Anaerotruncus sp.	4.2.1.11
D1AUQ0	enolase	XII	Anaplasma centrale	4.2.1.11
Q5PAS6	enolase	XII	Anaplasma marginale	4.2.1.11
Q2GK24	enolase	XII	Anaplasma phagocytophilum	4.2.1.11
A0A074L5A1	enolase	XII	Anditalea andensis	4.2.1.11
A0A0D1VUV8	enolase	XII	Aneurinibacillus migulanus	4.2.1.11
S9UQY9	enolase	XII	Angomonas deanei	
Q8MU59	enolase	XII	Anisakis simplex	4.2.1.11
A0A059F2B4	enolase	XII	Anncaliia algerae	
A0A084VBB8	enolase	XII	Anopheles sinensis	
A0A061H0M9	enolase	XII	Anthracocystis flocculosa	
W4G9J6	enolase	XII	Aphanomyces astaci	
W4GNV5	enolase	XII	Aphanomyces astaci	
A0A0C5QH05	enolase	XII	Aphis gossypii	
A0A088A4S1	enolase	XII	Apis mellifera	
A0A011U786	enolase	XII	Aquamicrobium defluvii	4.2.1.11
A0A095X718	enolase	XII	Arcanobacterium sp.	4.2.1.11
O29133	enolase	XII	Archaeoglobus fulgidus	4.2.1.11
N0BJJ5	enolase	XII	Archaeoglobus sulfaticallidus	4.2.1.11
F2KPR7	enolase	XII	Archaeoglobus veneficus	4.2.1.11
A0A0B5HVJ7	enolase	XII	archaeon GW2011 AR10	4.2.1.11
A0A0B5HNI5	enolase	XII	archaeon GW2011 AR15	4.2.1.11
A0A0B3AA17	enolase	XII	archaeon GW2011 AR3	4.2.1.11
A0A0B3AD65	enolase	XII	archaeon GW2011 AR4	4.2.1.11
A0A0B3AGC8	enolase	XII	archaeon GW2011 AR9	4.2.1.11
A8EWY0	enolase	XII	Arcobacter butzleri	4.2.1.11
R9GSL1	enolase	XII	Arcticibacter svalbardensis	4.2.1.11
A0A091BEX6	enolase	XII	Arenimonas composti	4.2.1.11
A0A091BEF0	enolase	XII	Arenimonas malthae	4.2.1.11
A0A091AZE0	enolase	XII	Arenimonas oryziterrae	4.2.1.11
A0A095YF18	enolase	XII	Arthrobacter albus	4.2.1.11
E1VTK8	enolase	XII	Arthrobacter arilaitensis	4.2.1.11
A1R485	enolase	XII	Arthrobacter aurescens	4.2.1.11
N1UX89	enolase	XII	Arthrobacter crystallopoietes	4.2.1.11
A0A078MPS4	enolase	XII	Arthrobacter sp.	4.2.1.11
A0A0A1D221	enolase	XII	Arthrobacter sp.	4.2.1.11
A0A060QFF5	enolase	XII	Asaia platycodi	4.2.1.11
E5D7U6	enolase	XII	Ascaris suum	
F1KZV7	enolase	XII	Ascaris suum	
A0A017SKH9	enolase	XII	Aspergillus ruber	
V4Q0S0	enolase	XII	Asticcacaulis benevestitus	4.2.1.11
V4NT00	enolase	XII	Asticcacaulis sp.	4.2.1.11
L7R619	enolase	XII	Astrotischeria sp.	
D3EPI1	enolase	XII	Atelocyanobacterium thalassa	4.2.1.11
N2BSQ1	enolase	XII	Atopobium minutum	4.2.1.11
U1WU47	enolase	XII	Atopobium sp.	4.2.1.11
Z4WQ88	enolase	XII	Atopobium sp.	4.2.1.11
F1T678	enolase	XII	Atopobium vaginae	4.2.1.11
Q1YI20	enolase	XII	Aurantimonas manganoxydans	4.2.1.11

Table S2.6. (continuation)

UniProt	Family [3]	Cluster	Species	EC Number
A0A0B1Q696	enolase	XII	Aureimonas altamirensis	4.2.1.11
A0A074XUU4	enolase	XII	Aureobasidium pullulans	
A0A074Y2Z9	enolase	XII	Aureobasidium subglaciale	
K6VMU6	enolase	XII	Austwickia chelonae	4.2.1.11
A1K7F6	enolase	XII	Azoarcus sp.	4.2.1.11
H0PST5	enolase	XII	Azoarcus sp.	4.2.1.11
B6YQN5	enolase	XII	Azobacteroides pseudotrichonymphae	4.2.1.11
G7ZBA7	enolase	XII	Azospirillum lipoferum	4.2.1.11
R6I0U5	enolase	XII	Azospirillum sp.	4.2.1.11
A0A0F2KLX9	enolase	XII	Azospirillum thiophilum	4.2.1.11
A0A0C4WI98	enolase	XII	Azotobacter chroococcum	4.2.1.11
A0A061D7V7	enolase	XII	Babesia bigemina	
W4QLY1	enolase	XII	Bacillus akibai	4.2.1.11
A0A072NSP6	enolase	XII	Bacillus azotoformans	4.2.1.11
A0A0C2YCL4	enolase	XII	Bacillus badius	4.2.1.11
E6TRC7	enolase	XII	Bacillus cellulosilyticus	4.2.1.11
R8SPP2	enolase	XII	Bacillus cereus	4.2.1.11
F7Z5V9	enolase	XII	Bacillus coagulans	4.2.1.11
A7GUR7	enolase	XII	Bacillus cytotoxicus	4.2.1.11
A0A0A6Y2H5	enolase	XII	Bacillus ginsengihumi	4.2.1.11
Q9K717	enolase	XII	Bacillus halodurans	4.2.1.11
A0A084GMW6	enolase	XII	Bacillus indicus	4.2.1.11
Q65EN2	enolase	XII	Bacillus licheniformis	4.2.1.11
I8UJJ8	enolase	XII	Bacillus macauensis	4.2.1.11
R9C685	enolase	XII	Bacillus nealsonii	4.2.1.11
D6Y131	enolase	XII	Bacillus selenitireducens	4.2.1.11
A0A0A8JDW5	enolase	XII	Bacillus sp.	4.2.1.11
A6CTF9	enolase	XII	Bacillus sp.	4.2.1.11
R7F3G1	enolase	XII	Bacillus sp.	4.2.1.11
U1YXI7	enolase	XII	Bacillus sp.	4.2.1.11
A0A0D0FSY0	enolase	XII	Bacillus thermoamylovorans	4.2.1.11
T0C3C0	enolase	XII	Bacteriovorax sp.	4.2.1.11
T0SFR9	enolase	XII	Bacteriovorax sp.	4.2.1.11
T0SW82	enolase	XII	Bacteriovorax sp.	4.2.1.11
A0A081C9G1	enolase	XII	bacterium UASB270	4.2.1.11
A0A0A1PWK2	enolase	XII	bacterium YEK0313	4.2.1.11
A0A0A1PXY9	enolase	XII	bacterium YEK0313	4.2.1.11
U5Q7Q1	enolase	XII	Bacteroidales bacterium	4.2.1.11
F3ZNJ2	enolase	XII	Bacteroides coprosuis	4.2.1.11
A0A015URF2	enolase	XII	Bacteroides fragilis	4.2.1.11
A0A017PKJ1	enolase	XII	Bacteroides fragilis	4.2.1.11
R5AXN1	enolase	XII	Bacteroides sp.	4.2.1.11
R5C628	enolase	XII	Bacteroides sp.	4.2.1.11
R5F4N9	enolase	XII	Bacteroides sp.	4.2.1.11
R5RUU6	enolase	XII	Bacteroides sp.	4.2.1.11
R5YMZ3	enolase	XII	Bacteroides sp.	4.2.1.11
A6L3M9	enolase	XII	Bacteroides vulgatus	4.2.1.11
S8FEI4	enolase	XII	Bacteroidetes bacterium	4.2.1.11
W0EUB2	enolase	XII	Barnesiella viscericola	4.2.1.11
M1P3G5	enolase	XII	Bartonella australis	4.2.1.11
J1JCT8	enolase	XII	Bartonella doshiae	4.2.1.11
E6YV45	enolase	XII	Bartonella sp.	4.2.1.11
J1JZZ9	enolase	XII	Bartonella tamiae	4.2.1.11
A0A077DG27	enolase	XII	Basilea psittacipulmonis	4.2.1.11
Q1LTN8	enolase	XII	Baumannia cicadellinicola	4.2.1.11
Q6MPQ2	enolase	XII	Bdellovibrio bacteriovorus	4.2.1.11

Table S2.6. (continuation)

UniProt	Family [3]	Cluster	Species	EC Number
W5WWX2	enolase	XII	Bdellovibrio bacteriovorus	4.2.1.11
M4VA81	enolase	XII	Bdellovibrio exovorus	4.2.1.11
I3CGF1	enolase	XII	Beggiatoa alba	4.2.1.11
B2IKR4	enolase	XII	Beijerinckia indica	4.2.1.11
I3Z523	enolase	XII	Belliella baltica	4.2.1.11
Q1N1K9	enolase	XII	Bermanella marisrubri	4.2.1.11
B6BUG8	enolase	XII	beta proteobacterium	4.2.1.11
C5C093	enolase	XII	Beutenbergia cavernae	4.2.1.11
C5C344	enolase	XII	Beutenbergia cavernae	4.2.1.11
M7NVD4	enolase	XII	Bhargavaea cecembensis	4.2.1.11
B7GTK2	enolase	XII	Bifidobacterium longum	4.2.1.11
A0A087BVH7	enolase	XII	Bifidobacterium mongoliense	4.2.1.11
R6PZZ7	enolase	XII	Bifidobacterium pseudocat.	4.2.1.11
A0A087CCT8	enolase	XII	Bifidobacterium psychraerophilum	4.2.1.11
A0A087CDH6	enolase	XII	Bifidobacterium psychraerophilum	4.2.1.11
A0A087E7E1	enolase	XII	Bifidobacterium subtile	4.2.1.11
E5Y3S6	enolase	XII	Bilophila wadsworthia	4.2.1.11
G2EBH4	enolase	XII	Bizionia argentinensis	4.2.1.11
H6RSI7	enolase	XII	Blastococcus saxobsidens	4.2.1.11
W9BW68	enolase	XII	Blastomonas sp.	4.2.1.11
D0J9G5	enolase	XII	Blattabacterium sp.	4.2.1.11
D0JAU7	enolase	XII	Blattabacterium sp.	4.2.1.11
G7SQ43	enolase	XII	Blattabacterium sp.	4.2.1.11
G8LRT6	enolase	XII	Blattabacterium sp.	4.2.1.11
I3QGR6	enolase	XII	Blattabacterium sp.	4.2.1.11
M4ZSN7	enolase	XII	Blattabacterium sp.	4.2.1.11
U3Q9K8	enolase	XII	Blattabacterium sp.	4.2.1.11
Q2HXW9	enolase	XII	Blattella germanica	
R5BYA2	enolase	XII	Blautia hydrogenotrophica	4.2.1.11
D4LTZ5	enolase	XII	Blautia obeum	4.2.1.11
R5WR54	enolase	XII	Blautia sp.	4.2.1.11
R6GIK6	enolase	XII	Blautia sp.	4.2.1.11
R6KMY2	enolase	XII	Blautia sp.	4.2.1.11
R6KQX6	enolase	XII	Blautia sp.	4.2.1.11
R7JUY7	enolase	XII	Blautia sp.	4.2.1.11
A0A0E3U3U0	enolase	XII	Blochmannia endosymbiont	4.2.1.11
A0A0E3U3V9	enolase	XII	Blochmannia endosymbiont	4.2.1.11
Q7VQH3	enolase	XII	Blochmannia floridanus	4.2.1.11
E8Q5R8	enolase	XII	Blochmannia vafer	4.2.1.11
A9IIP8	enolase	XII	Bordetella petrii	4.2.1.11
A0A085FC53	enolase	XII	Bosea sp.	4.2.1.11
A0A022KRJ8	enolase	XII	Brachybacterium muris	4.2.1.11
Z9JRZ5	enolase	XII	Brachybacterium phenoliresistens	4.2.1.11
R5HIM5	enolase	XII	Brachyspira sp.	4.2.1.11
A0A0D1L9C0	enolase	XII	Bradyrhizobium elkanii	4.2.1.11
I2JUE0	enolase	XII	Brettanomyces bruxellensis	
H0U8M2	enolase	XII	Brevibacillus laterosporus	4.2.1.11
K9AP50	enolase	XII	Brevibacterium casei	4.2.1.11
F4R2M3	enolase	XII	Brevundimonas diminuta	4.2.1.11
W7CMC8	enolase	XII	Brochothrix thermosphacta	4.2.1.11
A5VQQ7	enolase	XII	Brucella ovis	4.2.1.11
W5XWA0	enolase	XII	Brugia malayi	
F7WZI6	enolase	XII	Buchnera aphidicola	4.2.1.11
G2LNB7	enolase	XII	Buchnera aphidicola	4.2.1.11
G2LPR0	enolase	XII	Buchnera aphidicola	4.2.1.11
P57492	enolase	XII	Buchnera aphidicola	4.2.1.11

Table S2.6. (continuation)

UniProt	Family [3]	Cluster	Species	EC Number
P59566	enolase	XII	Buchnera aphidicola	4.2.1.11
Q057H3	enolase	XII	Buchnera aphidicola	4.2.1.11
W0P4H1	enolase	XII	Buchnera aphidicola	4.2.1.11
D2MMA0	enolase	XII	Bulleidia extructa	4.2.1.11
A0A088UH93	enolase	XII	Burkholderia cenocepacia	4.2.1.11
A0A038GYN1	enolase	XII	Burkholderia jiangsuensis	4.2.1.11
A0A063BLY4	enolase	XII	Burkholderia sp.	4.2.1.11
G8MLM1	enolase	XII	Burkholderia sp.	4.2.1.11
I5D2M8	enolase	XII	Burkholderia terrae	4.2.1.11
A0A0A1H932	enolase	XII	Burkholderiales bacterium	4.2.1.11
H5WU84	enolase	XII	Burkholderiales bacterium	4.2.1.11
H5WV27	enolase	XII	Burkholderiales bacterium	4.2.1.11
R8VSD1	enolase	XII	Butyricicoccus pullicaecorum	4.2.1.11
R6QNB4	enolase	XII	Butyrivibrio sp.	4.2.1.11
K9HID7	enolase	XII	Caenispirillum salinarum	4.2.1.11
Q27527	enolase	XII	Caenorhabditis elegans	4.2.1.11
B9MS47	enolase	XII	Caldicellulosiruptor bescii	4.2.1.11
I0HYU7	enolase	XII	Caldilinea aerophila	4.2.1.11
R1CVU6	enolase	XII	Caldisalinibacter kiritimatiensis	4.2.1.11
I0GJN1	enolase	XII	Caldisericum exile	4.2.1.11
L0A874	enolase	XII	Caldisphaera lagunensis	4.2.1.11
E4TII5	enolase	XII	Calditerrivibrio nitroreducens	4.2.1.11
H1XXR5	enolase	XII	Caldithrix abyssi	4.2.1.11
V9KT03	enolase	XII	Callorhinchus milii	
A0A096CUQ6	enolase	XII	Caloranaerobacter azorensis	4.2.1.11
K9UVM7	enolase	XII	Calothrix sp.	4.2.1.11
A6DBB7	enolase	XII	Caminibacter mediatlanticus	4.2.1.11
E2A4J2	enolase	XII	Camponotus floridanus	
A0A071L7T1	enolase	XII	Campylobacter hyointestinalis	4.2.1.11
A0A076FE33	enolase	XII	Campylobacter iguaniorum	4.2.1.11
A8FNY3	enolase	XII	Campylobacter jejuni	4.2.1.11
B9KEN0	enolase	XII	Campylobacter lari	4.2.1.11
E6LBL3	enolase	XII	Campylobacter upsaliensis	4.2.1.11
S3XYQ3	enolase	XII	Campylobacter ureolyticus	4.2.1.11
P30575	enolase	XII	Candida albicans	4.2.1.11
Q6FQY4	enolase	XII	Candida glabrata	4.2.1.11
Q6FTW6	enolase	XII	Candida glabrata	4.2.1.11
M3HQP4	enolase	XII	Candida maltosa	
H8WWN3	enolase	XII	Candida orthopsilosis	
G3B384	enolase	XII	Candida tenuis	
C5MD83	enolase	XII	Candida tropicalis	
T0L8N5	enolase	XII	candidate division	4.2.1.11
V5RSC4	enolase	XII	candidate division	4.2.1.11
A0A011R2D7	enolase	XII	Candidatus Accumulibacter	4.2.1.11
A0A011R6R8	enolase	XII	Candidatus Accumulibacter	4.2.1.11
A0A031LP76	enolase	XII	Candidatus Acidianus	4.2.1.11
S5DYE0	enolase	XII	Candidatus Actinomarina	4.2.1.11
I0R1E0	enolase	XII	Candidatus Aquiluna	4.2.1.11
A0A0F5MMT9	enolase	XII	Candidatus Arcanobacter	4.2.1.11
G2IFW8	enolase	XII	Candidatus Arthromitus	4.2.1.11
A0A086CFU7	enolase	XII	Candidatus Atelocyanobacterium	4.2.1.11
V6DGH4	enolase	XII	Candidatus Babela	4.2.1.11
A0A088NAG7	enolase	XII	Candidatus Baumannia	4.2.1.11
A0A077BZY2	enolase	XII	Candidatus Caedibacter	4.2.1.11
E6P9C1	enolase	XII	Candidatus Caldiarchaeum	4.2.1.11
W6LXS8	enolase	XII	Candidatus Contendobacter	4.2.1.11

Table S2.6. (continuation)

UniProt	Family [3]	Cluster	Species	EC Number
K7YMT3	enolase	XII	Candidatus Endolissoclinum	4.2.1.11
V9TV54	enolase	XII	Candidatus Endolissoclinum	4.2.1.11
W4LQ41	enolase	XII	Candidatus Entotheonella	4.2.1.11
G2J9E7	enolase	XII	Candidatus Glomeribacter	4.2.1.11
V4HI73	enolase	XII	Candidatus Halobonum	4.2.1.11
W8GT69	enolase	XII	Candidatus Hepatoplasma	4.2.1.11
C5WCD6	enolase	XII	Candidatus Ishikawaella	4.2.1.11
I3IQ47	enolase	XII	Candidatus Jettenia	4.2.1.11
M1L2L6	enolase	XII	Candidatus Kinetoplastibacterium	4.2.1.11
M1LQ27	enolase	XII	Candidatus Kinetoplastibacterium	4.2.1.11
M1MBA5	enolase	XII	Candidatus Kinetoplastibacterium	4.2.1.11
Q1PYT0	enolase	XII	Candidatus Kuenenia	4.2.1.11
A0A0F3GUI8	enolase	XII	Candidatus Magnetobacterium	4.2.1.11
R9T650	enolase	XII	Candidatus Methanomassiliicoccus	4.2.1.11
A0A062UZ33	enolase	XII	Candidatus Methanoperedens	4.2.1.11
A0A0A7LBA2	enolase	XII	Candidatus Methanoplasma	4.2.1.11
R4Z728	enolase	XII	Candidatus Microthrix	4.2.1.11
A0A0F3NM79	enolase	XII	Candidatus Neoehrlichia	4.2.1.11
F9CU46	enolase	XII	Candidatus Nitrosoarchaeum	4.2.1.11
I3D0N0	enolase	XII	Candidatus Nitrosopumilus	4.2.1.11
K0B7H8	enolase	XII	Candidatus Nitrosopumilus	4.2.1.11
U3U5M0	enolase	XII	Candidatus Pantoea	4.2.1.11
A0A077AVM8	enolase	XII	Candidatus Paracaedibacter	4.2.1.11
X5MEN1	enolase	XII	Candidatus Phaeomarinobacter	4.2.1.11
A0A084CNU9	enolase	XII	Candidatus Photodesmus	4.2.1.11
S3DZI4	enolase	XII	Candidatus Photodesmus	4.2.1.11
U6E0B2	enolase	XII	Candidatus Phytoplasma	
G2GXN7	enolase	XII	Candidatus Regiella	4.2.1.11
A0A0C1V7Q8	enolase	XII	Candidatus Riesia	4.2.1.11
A0A0A7PB42	enolase	XII	Candidatus Saccharibacteria	4.2.1.11
A0A0B0EJN5	enolase	XII	Candidatus Scalindua	4.2.1.11
U5N4K5	enolase	XII	Candidatus Symbiobacter	4.2.1.11
A0A090AQC0	enolase	XII	Candidatus Tachikawaea	4.2.1.11
A0A0A6P389	enolase	XII	Candidatus Thiomargarita	4.2.1.11
L7VN06	enolase	XII	Candidatus Uzinura	4.2.1.11
R7VFP9	enolase	XII	Capitella teleta	
R7VKI5	enolase	XII	Capitella teleta	
S2VKL4	enolase	XII	Capnocytophaga granulosa	4.2.1.11
J1GSM3	enolase	XII	Capnocytophaga sp.	4.2.1.11
Q3AFC8	enolase	XII	Carboxydothermus hydrog.	4.2.1.11
X5J8W8	enolase	XII	Cardinium endosymbiont	4.2.1.11
G9ZHP1	enolase	XII	Cardiobacterium valvarum	4.2.1.11
U5S754	enolase	XII	Carnobacterium inhibens	4.2.1.11
K8E5Q7	enolase	XII	Carnobacterium maltaromaticum	4.2.1.11
W8X369	enolase	XII	Castellaniella defragrans	4.2.1.11
K8ZC71	enolase	XII	Catellicoccus marimammalium	4.2.1.11
R7GLE2	enolase	XII	Catenibacterium sp.	4.2.1.11
W7QKR7	enolase	XII	Catenovulum agarivorans	4.2.1.11
C7Q4T1	enolase	XII	Catenulispora acidiphila	4.2.1.11
C7QJ14	enolase	XII	Catenulispora acidiphila	4.2.1.11
V2Z4X0	enolase	XII	Catonella morbi	4.2.1.11
B8GW68	enolase	XII	Caulobacter crescentus	4.2.1.11
A0A0B5DSB8	enolase	XII	Celeribacter indicus	4.2.1.11
A0A0A0B6K5	enolase	XII	Cellulomonas cellasea	4.2.1.11
F4GYM5	enolase	XII	Cellulomonas fimi	4.2.1.11
E6XEG1	enolase	XII	Cellulophaga algicola	4.2.1.11

Table S2.6. (continuation)

UniProt	Family [3]	Cluster	Species	EC Number
F2JKY7	enolase	XII	Cellulosilyticum lentocellum	4.2.1.11
B3PJB3	enolase	XII	Cellvibrio japonicus	4.2.1.11
A0A026W628	enolase	XII	Cerapachys biroi	
M2R5Y1	enolase	XII	Ceriporiopsis subvermispora	
M7N9S4	enolase	XII	Cesiribacter andamanensis	4.2.1.11
U7V8T5	enolase	XII	Cetobacterium somerae	4.2.1.11
K9UM30	enolase	XII	Chamaesiphon minutus	4.2.1.11
Q11HU8	enolase	XII	Chelativorans sp.	4.2.1.11
M7AU47	enolase	XII	Chelonia mydas	
U7DBA8	enolase	XII	Chitinivibrio alkaliphilus	4.2.1.11
Q9PJF3	enolase	XII	Chlamydia muridarum	4.2.1.11
V8TSC2	enolase	XII	Chlamydia pecorum	4.2.1.11
Q9Z7A6	enolase	XII	Chlamydia pneumoniae	4.2.1.11
S7J3E1	enolase	XII	Chlamydia psittaci	4.2.1.11
S7J7B3	enolase	XII	Chlamydia psittaci	4.2.1.11
Q821H7	enolase	XII	Chlamydophila caviae	4.2.1.11
G2LGQ2	enolase	XII	Chloracidobacterium thermophilum	4.2.1.11
B3EL51	enolase	XII	Chlorobium phaeobacteroides	4.2.1.11
A4SGL6	enolase	XII	Chlorobium phaeovibrioides	4.2.1.11
Q8KG25	enolase	XII	Chlorobium tepidum	4.2.1.11
B3QXY4	enolase	XII	Chloroherpeton thalassium	4.2.1.11
A0A017T7X0	enolase	XII	Chondromyces apiculatus	4.2.1.11
R7QG79	enolase	XII	Chondrus crispus	
A0A0C1CM03	enolase	XII	Chromobacterium piscinae	4.2.1.11
Q1QZX7	enolase	XII	Chromohalobacter salexigens	4.2.1.11
D7W0W2	enolase	XII	Chryseobacterium gleum	4.2.1.11
A0A0C1FD52	enolase	XII	Chryseobacterium jeonii	4.2.1.11
B4D3Y9	enolase	XII	Chthoniobacter flavus	4.2.1.11
B4D7J4	enolase	XII	Chthoniobacter flavus	4.2.1.11
S0EX45	enolase	XII	Chthonomonas calidirosea	4.2.1.11
F6XZ93	enolase	XII	Ciona intestinalis	
R8WMY5	enolase	XII	Citrobacter sp.	4.2.1.11
A0A0D2G558	enolase	XII	Cladophialophora bantiana	
A0A0D2DE61	enolase	XII	Cladophialophora immunda	
B0RH60	enolase	XII	Clavibacter michiganensis	4.2.1.11
B0VFQ0	enolase	XII	Cloacimonas acidaminovorans	4.2.1.11
G7YF68	enolase	XII	Clonorchis sinensis	
A0A0F2PY17	enolase	XII	Clostridiaceae bacterium	4.2.1.11
C5EGE1	enolase	XII	Clostridiales bacterium	4.2.1.11
U2CZA6	enolase	XII	Clostridiales bacterium	4.2.1.11
K0B0D4	enolase	XII	Clostridium acidurici	4.2.1.11
A0A0C1R7E7	enolase	XII	Clostridium argentinense	4.2.1.11
A0A0A7FSA5	enolase	XII	Clostridium baratii	4.2.1.11
A8RRX2	enolase	XII	Clostridium bolteae	4.2.1.11
W6RWZ7	enolase	XII	Clostridium bornimense	4.2.1.11
W6RZM7	enolase	XII	Clostridium bornimense	4.2.1.11
B8I4U1	enolase	XII	Clostridium cellulolyticum	4.2.1.11
D9SRY4	enolase	XII	Clostridium cellulovorans	4.2.1.11
S6ET97	enolase	XII	Clostridium chauvoei	4.2.1.11
R7PLU1	enolase	XII	Clostridium clostridioforme	4.2.1.11
N9WAS7	enolase	XII	Clostridium colicanis	4.2.1.11
U2NLK5	enolase	XII	Clostridium intestinale	4.2.1.11
B9E6B1	enolase	XII	Clostridium kluyveri	4.2.1.11
D8GUQ9	enolase	XII	Clostridium ljungdahlii	4.2.1.11
R6PCS5	enolase	XII	Clostridium nexile	4.2.1.11
R4KCY6	enolase	XII	Clostridium pasteurianum	4.2.1.11

Table S2.6. (continuation)

UniProt	Family [3]	Cluster	Species	EC Number
Q0TQZ2	enolase	XII	Clostridium perfringens	4.2.1.11
A9KQ46	enolase	XII	Clostridium phytofermentans	4.2.1.11
A0A0E3K339	enolase	XII	Clostridium scatologenes	4.2.1.11
A0A099SCX5	enolase	XII	Clostridium sp.	4.2.1.11
D4CEB5	enolase	XII	Clostridium sp.	4.2.1.11
F0YV98	enolase	XII	Clostridium sp.	4.2.1.11
F0Z038	enolase	XII	Clostridium sp.	4.2.1.11
F7V8H4	enolase	XII	Clostridium sp.	4.2.1.11
J0MXT4	enolase	XII	Clostridium sp.	4.2.1.11
J1HAK4	enolase	XII	Clostridium sp.	4.2.1.11
K6U295	enolase	XII	Clostridium sp.	4.2.1.11
N2AUK7	enolase	XII	Clostridium sp.	4.2.1.11
R5A8T9	enolase	XII	Clostridium sp.	4.2.1.11
R5J3B2	enolase	XII	Clostridium sp.	4.2.1.11
R5JWR0	enolase	XII	Clostridium sp.	4.2.1.11
R5KKF9	enolase	XII	Clostridium sp.	4.2.1.11
R5KZC0	enolase	XII	Clostridium sp.	4.2.1.11
R5P359	enolase	XII	Clostridium sp.	4.2.1.11
R5QCC7	enolase	XII	Clostridium sp.	4.2.1.11
R5SXG5	enolase	XII	Clostridium sp.	4.2.1.11
R5T9N8	enolase	XII	Clostridium sp.	4.2.1.11
R5YTN6	enolase	XII	Clostridium sp.	4.2.1.11
R6BE23	enolase	XII	Clostridium sp.	4.2.1.11
R6C434	enolase	XII	Clostridium sp.	4.2.1.11
R6CAR5	enolase	XII	Clostridium sp.	4.2.1.11
R6CWK7	enolase	XII	Clostridium sp.	4.2.1.11
R6DDR6	enolase	XII	Clostridium sp.	4.2.1.11
R6GR49	enolase	XII	Clostridium sp.	4.2.1.11
R6M9W9	enolase	XII	Clostridium sp.	4.2.1.11
R6NC83	enolase	XII	Clostridium sp.	4.2.1.11
R6TUU5	enolase	XII	Clostridium sp.	4.2.1.11
R6X8W2	enolase	XII	Clostridium sp.	4.2.1.11
R6Y2J9	enolase	XII	Clostridium sp.	4.2.1.11
R6Y9W2	enolase	XII	Clostridium sp.	4.2.1.11
R7A1X2	enolase	XII	Clostridium sp.	4.2.1.11
R7ARZ3	enolase	XII	Clostridium sp.	4.2.1.11
R7C9K1	enolase	XII	Clostridium sp.	4.2.1.11
R7FM99	enolase	XII	Clostridium sp.	4.2.1.11
R7GFJ1	enolase	XII	Clostridium sp.	4.2.1.11
R7GKB7	enolase	XII	Clostridium sp.	4.2.1.11
R7JZY1	enolase	XII	Clostridium sp.	4.2.1.11
R7KAI6	enolase	XII	Clostridium sp.	4.2.1.11
R7LMH9	enolase	XII	Clostridium sp.	4.2.1.11
R7LT33	enolase	XII	Clostridium sp.	4.2.1.11
R7MIY7	enolase	XII	Clostridium sp.	4.2.1.11
R7P9U4	enolase	XII	Clostridium sp.	4.2.1.11
U2BJ93	enolase	XII	Clostridium sp.	4.2.1.11
U2DVZ7	enolase	XII	Clostridium sp.	4.2.1.11
L7VLP4	enolase	XII	Clostridium stercorarium	4.2.1.11
A0A084JC85	enolase	XII	Clostridium sulfidigenes	4.2.1.11
Q898R0	enolase	XII	Clostridium tetani	4.2.1.11
A3DBQ5	enolase	XII	Clostridium thermocellum	4.2.1.11
W6N463	enolase	XII	Clostridium tyrobutyricum	4.2.1.11
M2S4T4	enolase	XII	Cochliobolus sativus	
A0A0C2QHS7	enolase	XII	Cohnella sp.	4.2.1.11
B4W4X0	enolase	XII	Coleofasciculus chthonoplastes	4.2.1.11

Table S2.6. (continuation)

UniProt	Family [3]	Cluster	Species	EC Number
R6ZBN4	enolase	XII	Collinsella sp.	4.2.1.11
G1WI20	enolase	XII	Collinsella tanakaei	4.2.1.11
A0A099L184	enolase	XII	Colwellia psychrerythraea	4.2.1.11
Q47WR1	enolase	XII	Colwellia psychrerythraea	4.2.1.11
A0A0E3B9K5	enolase	XII	Comamonas testosteroni	4.2.1.11
G6EZF6	enolase	XII	Commensalibacter intestini	4.2.1.11
D3F4F0	enolase	XII	Conexibacter woesei	4.2.1.11
D3F9W2	enolase	XII	Conexibacter woesei	4.2.1.11
D3FEF2	enolase	XII	Conexibacter woesei	4.2.1.11
E7GDZ0	enolase	XII	Coprobacillus sp.	4.2.1.11
H1AJH8	enolase	XII	Coprobacillus sp.	4.2.1.11
R5G4X5	enolase	XII	Coprobacillus sp.	4.2.1.11
R7DT71	enolase	XII	Coprobacillus sp.	4.2.1.11
D4JC66	enolase	XII	Coprococcus catus	4.2.1.11
R5JG55	enolase	XII	Coprococcus sp.	4.2.1.11
R7L502	enolase	XII	Coraliomargarita sp.	4.2.1.11
H8MHW0	enolase	XII	Corallococcus coralloides	4.2.1.11
A0A0A8B4X1	enolase	XII	Coriobacteriaceae bacterium	4.2.1.11
D5NVU0	enolase	XII	Corynebacterium ammoniagenes	4.2.1.11
U3GWH2	enolase	XII	Corynebacterium argentoratense	4.2.1.11
A0A075TUK9	enolase	XII	Corynebacterium atypicum	4.2.1.11
C3PFA7	enolase	XII	Corynebacterium aurimucosum	4.2.1.11
A0A097IET2	enolase	XII	Corynebacterium doosanense	4.2.1.11
L1MN98	enolase	XII	Corynebacterium durum	4.2.1.11
A0A095Y0T2	enolase	XII	Corynebacterium freneyi	4.2.1.11
C2GIP5	enolase	XII	Corynebacterium glucuronolyticum	4.2.1.11
X5DS44	enolase	XII	Corynebacterium glyciniphilum	4.2.1.11
A0A076NQL1	enolase	XII	Corynebacterium imitans	4.2.1.11
E0DIN6	enolase	XII	Corynebacterium matruchotii	4.2.1.11
F8E0I0	enolase	XII	Corynebacterium resistens	4.2.1.11
S2Y239	enolase	XII	Corynebacterium sp.	4.2.1.11
U7MKX9	enolase	XII	Corynebacterium sp.	4.2.1.11
S4XFA0	enolase	XII	Corynebacterium terpenotabidum	4.2.1.11
A0A095YGV8	enolase	XII	Corynebacterium tuscaniense	4.2.1.11
B1VFL0	enolase	XII	Corynebacterium urealyticum	4.2.1.11
A0A077HM10	enolase	XII	Corynebacterium ureicelerivorans	4.2.1.11
A0A0A8E8C8	enolase	XII	Coxiella endosymbiont	4.2.1.11
A0A090CZI2	enolase	XII	Criblamydia sequanensis	4.2.1.11
K9VY78	enolase	XII	Crinalium epipsammum	4.2.1.11
A3U7P5	enolase	XII	Croceibacter atlanticus	4.2.1.11
A0A086E107	enolase	XII	Cryobacterium sp.	4.2.1.11
Q6RG04	enolase	XII	Cryphonectria parasitica	4.2.1.11
C7MP99	enolase	XII	Cryptobacterium curtum	4.2.1.11
Q5KG11	enolase	XII	Cryptococcus neoformans	
Q5KLA7	enolase	XII	Cryptococcus neoformans	
A0A010ZMQ2	enolase	XII	Cryptosporangium arvum	4.2.1.11
F0X3K6	enolase	XII	Cryptosporidium parvum	
A0A0C4YUL7	enolase	XII	Cupriavidus basilensis	4.2.1.11
G0EYL2	enolase	XII	Cupriavidus necator	4.2.1.11
A0A069IDF6	enolase	XII	Cupriavidus sp.	4.2.1.11
A0A022L8J1	enolase	XII	Curtobacterium flaccumfaciens	4.2.1.11
A0A022LJX3	enolase	XII	Curtobacterium flaccumfaciens	4.2.1.11
M1V8C2	enolase	XII	Cyanidioschyzon merolae	
K9Z2Q2	enolase	XII	Cyanobacterium aponinum	4.2.1.11
K9YPY5	enolase	XII	Cyanobacterium stanieri	4.2.1.11
K9P9R2	enolase	XII	Cyanobium gracile	4.2.1.11

Table S2.6. (continuation)

UniProt	Family [3]	Cluster	Species	EC Number
B1WNS7	enolase	XII	Cyanothece sp.	4.2.1.11
B7JW48	enolase	XII	Cyanothece sp.	4.2.1.11
B7KL24	enolase	XII	Cyanothece sp.	4.2.1.11
B8HQY9	enolase	XII	Cyanothece sp.	4.2.1.11
A0A061B1C1	enolase	XII	Cyberlindnera fabianii	
S7VFJ1	enolase	XII	Cyclobacterium qasimii	4.2.1.11
K0C3E4	enolase	XII	Cycloclasticus sp.	4.2.1.11
S9P1Z2	enolase	XII	Cystobacter fuscus	4.2.1.11
M5G9J7	enolase	XII	Dacryopinax sp.	
S7ZZF7	enolase	XII	Dactylellina haptotyla	
K9YU61	enolase	XII	Dactylococcopsis salina	4.2.1.11
P42040	enolase	XII	Davidiella tassiana	4.2.1.11
Q6BI20	enolase	XII	Debaryomyces hansenii	4.2.1.11
Q6BTB1	enolase	XII	Debaryomyces hansenii	4.2.1.11
D3PAT9	enolase	XII	Deferribacter desulfuricans	4.2.1.11
A0A059ITG3	enolase	XII	Defluviimonas sp.	4.2.1.11
A0A0C7P350	enolase	XII	Defluviitoga tunisiensis	4.2.1.11
W0EJI7	enolase	XII	Dehalobacter restrictus	4.2.1.11
Q3ZX11	enolase	XII	Dehalococcoides mccartyi	4.2.1.11
C1CXJ3	enolase	XII	Deinococcus deserti	4.2.1.11
H8GS35	enolase	XII	Deinococcus gobiensis	4.2.1.11
E8U3X3	enolase	XII	Deinococcus maricopensis	4.2.1.11
L0A356	enolase	XII	Deinococcus peraridilitoris	4.2.1.11
F0RIS3	enolase	XII	Deinococcus proteolyticus	4.2.1.11
A9BZT6	enolase	XII	Delftia acidovorans	4.2.1.11
D8F8Y9	enolase	XII	delta proteobacterium	4.2.1.11
Q1NIN6	enolase	XII	delta proteobacterium	4.2.1.11
J3JWT4	enolase	XII	Dendroctonus ponderosae	
J3JXB7	enolase	XII	Dendroctonus ponderosae	
D4H5B2	enolase	XII	Denitrovibrio acetiphilus	4.2.1.11
S3Y237	enolase	XII	Dermabacter sp.	4.2.1.11
X4ZE83	enolase	XII	Dermatophagoides farinae	
F5SGJ0	enolase	XII	Desmospora sp.	4.2.1.11
E1QL98	enolase	XII	Desulfarculus baarsii	4.2.1.11
B8FKT4	enolase	XII	Desulfatibacillum alkenivorans	4.2.1.11
A0A0F2R1S2	enolase	XII	Desulfatitalea sp.	4.2.1.11
Q24MW5	enolase	XII	Desulfitobacterium hafniense	4.2.1.11
Q24YW4	enolase	XII	Desulfitobacterium hafniense	4.2.1.11
W0EGN3	enolase	XII	Desulfitobacterium metallireducens	4.2.1.11
F2NDZ5	enolase	XII	Desulfobacca acetoxidans	4.2.1.11
I5B5S6	enolase	XII	Desulfobacter postgatei	4.2.1.11
C0QI43	enolase	XII	Desulfobacterium autotrophicum	4.2.1.11
K0N7R0	enolase	XII	Desulfobacula toluolica	4.2.1.11
A0A0F2NR66	enolase	XII	Desulfobulbaceae bacterium	4.2.1.11
E8RGZ7	enolase	XII	Desulfobulbus propionicus	4.2.1.11
A0A0A2HYU8	enolase	XII	Desulfobulbus sp.	4.2.1.11
M1P7S7	enolase	XII	Desulfocapsa sulfexigens	4.2.1.11
A8ZSY5	enolase	XII	Desulfococcus oleovorans	4.2.1.11
C8X0C3	enolase	XII	Desulfohalobium retbaense	4.2.1.11
C7LXR1	enolase	XII	Desulfomicrobium baculatum	4.2.1.11
I4C776	enolase	XII	Desulfomonile tiedjei	4.2.1.11
A0A068JQN2	enolase	XII	Desulfonatronum thiodismutans	4.2.1.11
B1I0Y0	enolase	XII	Desulforudis audaxviator	4.2.1.11
I4DC84	enolase	XII	Desulfosporosinus acidiphilus	4.2.1.11
G2FZ17	enolase	XII	Desulfosporosinus sp.	4.2.1.11
Q6AM97	enolase	XII	Desulfotalea psychrophila	4.2.1.11

Table S2.6. (continuation)

UniProt	Family [3]	Cluster	Species	EC Number
S0FWX2	enolase	XII	Desulfotignum phosphitoxidans	4.2.1.11
C8VXZ7	enolase	XII	Desulfotomaculum acetoxidans	4.2.1.11
C8VZN6	enolase	XII	Desulfotomaculum acetoxidans	4.2.1.11
R4KLK7	enolase	XII	Desulfotomaculum gibsoniae	4.2.1.11
F6CN81	enolase	XII	Desulfotomaculum kuznetsovii	4.2.1.11
F6B4L0	enolase	XII	Desulfotomaculum nigrificans	4.2.1.11
F6DNA6	enolase	XII	Desulfotomaculum ruminis	4.2.1.11
E6VXR4	enolase	XII	Desulfovibrio aespoeensis	4.2.1.11
F3YUE2	enolase	XII	Desulfovibrio africanus	4.2.1.11
F3YYQ2	enolase	XII	Desulfovibrio africanus	4.2.1.11
Q316Q0	enolase	XII	Desulfovibrio alaskensis	4.2.1.11
S7UMG9	enolase	XII	Desulfovibrio alkalitolerans	4.2.1.11
B8J467	enolase	XII	Desulfovibrio desulfuricans	4.2.1.11
F0JC83	enolase	XII	Desulfovibrio desulfuricans	4.2.1.11
E1K175	enolase	XII	Desulfovibrio fructosivorans	4.2.1.11
T2G9P3	enolase	XII	Desulfovibrio gigas	4.2.1.11
C4XLR9	enolase	XII	Desulfovibrio magneticus	4.2.1.11
M1WQM5	enolase	XII	Desulfovibrio piezophilus	4.2.1.11
B6WVI9	enolase	XII	Desulfovibrio piger	4.2.1.11
C6BSL8	enolase	XII	Desulfovibrio salexigens	4.2.1.11
G1UU17	enolase	XII	Desulfovibrio sp.	4.2.1.11
S7VHH5	enolase	XII	Desulfovibrio sp.	4.2.1.11
O32513	enolase	XII	Desulfovibrio vulgaris	4.2.1.11
W0JF24	enolase	XII	Desulfurella acetivorans	4.2.1.11
E6W384	enolase	XII	Desulfurispirillum indicum	4.2.1.11
D6Z1Z2	enolase	XII	Desulfurivibrio alkaliphilus	4.2.1.11
F0S3I7	enolase	XII	Desulfurobacterium thermolithotrophum	4.2.1.11
I3XPV2	enolase	XII	Desulfurococcus fermentans	4.2.1.11
Q1K122	enolase	XII	Desulfuromonas acetoxidans	4.2.1.11
A0A0D2JA49	enolase	XII	Dethiosulfatarculus sandiegensis	4.2.1.11
A0A0F5QA03	enolase	XII	Devosia epidermidihirudinis	4.2.1.11
A0A0F5FRC7	enolase	XII	Devosia geojensis	4.2.1.11
A0A0F5LMS0	enolase	XII	Devosia limi	4.2.1.11
A0A087LN06	enolase	XII	Devosia sp.	4.2.1.11
C9LLT5	enolase	XII	Dialister invisus	4.2.1.11
E4L7U8	enolase	XII	Dialister microaerophilus	4.2.1.11
R6AQE1	enolase	XII	Dialister sp.	4.2.1.11
R7CKE7	enolase	XII	Dialister sp.	4.2.1.11
A5EW24	enolase	XII	Dichelobacter nodosus	4.2.1.11
B5YF30	enolase	XII	Dictyoglomus thermophilum	4.2.1.11
F0ZJC2	enolase	XII	Dictyostelium purpureum	
A0A022L2L3	enolase	XII	Dietzia sp.	4.2.1.11
A8LQL4	enolase	XII	Dinoroseobacter shibae	4.2.1.11
A0A0B6VRV3	enolase	XII	Diplonema papillatum	
A0A0A2GY83	enolase	XII	Dokdonia donghaensis	4.2.1.11
H3NBS1	enolase	XII	Dolosigranulum pigrum	4.2.1.11
R6S333	enolase	XII	Dorea formicigenerans	4.2.1.11
A6BJ74	enolase	XII	Dorea longicatena	4.2.1.11
R7FQ69	enolase	XII	Dorea longicatena	4.2.1.11
R6WUT3	enolase	XII	Dorea sp.	4.2.1.11
O02654	enolase	XII	Doryteuthis pealeii	4.2.1.11
X5DIB1	enolase	XII	Draconibacterium orientale	4.2.1.11
W7HVG5	enolase	XII	Drechslerella stenobrocha	
P15007	enolase	XII	Drosophila melanogaster	4.2.1.11
A0A023NSI6	enolase	XII	Dyella jiangningensis	4.2.1.11
F5IZU2	enolase	XII	Dysgonomonas gadei	4.2.1.11

Table S2.6. (continuation)

UniProt	Family [3]	Cluster	Species	EC Number
F8WXH5	enolase	XII	Dysgonomonas mossii	4.2.1.11
F8X0M1	enolase	XII	Dysgonomonas mossii	4.2.1.11
U6JBE4	enolase	XII	Echinococcus granulosus	
A0A068Y4F6	enolase	XII	Echinococcus multilocularis	
H1G6M6	enolase	XII	Ectothiorhodospira sp.	4.2.1.11
D0ZAE2	enolase	XII	Edwardsiella tarda	4.2.1.11
D4F7J4	enolase	XII	Edwardsiella tarda	4.2.1.11
M0Q9U2	enolase	XII	Edwardsiella tarda	4.2.1.11
F7UWQ0	enolase	XII	Eggerthella sp.	4.2.1.11
R5FKV2	enolase	XII	Eggerthella sp.	4.2.1.11
M2NG10	enolase	XII	Eggerthia catenaformis	4.2.1.11
Q3YRX9	enolase	XII	Ehrlichia canis	4.2.1.11
Q5HB46	enolase	XII	Ehrlichia ruminantium	4.2.1.11
R9CPP3	enolase	XII	Elizabethkingia meningoseptica	4.2.1.11
B2KBA5	enolase	XII	Elusimicrobium minutum	4.2.1.11
R1DQ51	enolase	XII	Emiliania huxleyi	
I2EY78	enolase	XII	Emticicia oligotrophica	4.2.1.11
E0S9V5	enolase	XII	Encephalitozoon intestinalis	
A0A0C1QK29	enolase	XII	endosymbiont of	4.2.1.11
G2DDK8	enolase	XII	endosymbiont of	4.2.1.11
S6BJC3	enolase	XII	endosymbiont of	4.2.1.11
A0A081KD28	enolase	XII	Endozoicomonas elysicola	4.2.1.11
A0A081N5B6	enolase	XII	Endozoicomonas montiporae	4.2.1.11
A0A081NBP6	enolase	XII	Endozoicomonas montiporae	4.2.1.11
A0A081NK39	enolase	XII	Endozoicomonas numazuensis	4.2.1.11
W8IFY6	enolase	XII	Ensifer adhaerens	4.2.1.11
P51555	enolase	XII	Entamoeba histolytica	4.2.1.11
S0B1X8	enolase	XII	Entamoeba invadens	
R2S4S4	enolase	XII	Enterococcus asini	4.2.1.11
P0DM31	enolase	XII	Enterococcus faecalis	4.2.1.11
S0JCK0	enolase	XII	Enterococcus saccharolyticus	4.2.1.11
W8GMW5	enolase	XII	Epicauta chinensis	
A0A085BNE5	enolase	XII	Epilithonimonas sp.	4.2.1.11
E4KPA7	enolase	XII	Eremococcus coleocola	4.2.1.11
I1VYS7	enolase	XII	Erwinia amylovora	4.2.1.11
E2SP01	enolase	XII	Erysipelotrichaceae bacterium	4.2.1.11
A0A074N3X7	enolase	XII	Erythrobacter litoralis	4.2.1.11
A0A074M4W7	enolase	XII	Erythrobacter sp.	4.2.1.11
A5P7B1	enolase	XII	Erythrobacter sp.	4.2.1.11
A7ZQM2	enolase	XII	Escherichia coli	4.2.1.11
E6U7N2	enolase	XII	Ethanoligenens harbinense	4.2.1.11
W8T533	enolase	XII	Eubacterium acidaminophilum	4.2.1.11
C4Z1M9	enolase	XII	Eubacterium eligens	4.2.1.11
R6G8L4	enolase	XII	Eubacterium hallii	4.2.1.11
E3GQN7	enolase	XII	Eubacterium limosum	4.2.1.11
C7H0M7	enolase	XII	Eubacterium saphenum	4.2.1.11
R5DWY2	enolase	XII	Eubacterium sp.	4.2.1.11
R5L8K6	enolase	XII	Eubacterium sp.	4.2.1.11
R5LBK0	enolase	XII	Eubacterium sp.	4.2.1.11
R5ZM53	enolase	XII	Eubacterium sp.	4.2.1.11
R6FX37	enolase	XII	Eubacterium sp.	4.2.1.11
R6N256	enolase	XII	Eubacterium sp.	4.2.1.11
R6Q9G2	enolase	XII	Eubacterium sp.	4.2.1.11
X8K3Z7	enolase	XII	Eubacterium sulci	4.2.1.11
A5Z9K9	enolase	XII	Eubacterium ventriosum	4.2.1.11
M7SUV3	enolase	XII	Eutypa lata	

Table S2.6. (continuation)

UniProt	Family [3]	Cluster	Species	EC Number
C4L5H4	enolase	XII	Exiguobacterium sp.	4.2.1.11
H6C452	enolase	XII	Exophiala dermatitidis	
A0A0D1ZP97	enolase	XII	Exophiala mesophila	
A0A0D2DTF2	enolase	XII	Exophiala oligosperma	
A0A0D1X1A0	enolase	XII	Exophiala sideris	
A0A0D2EVD2	enolase	XII	Exophiala xenobiotica	
K1LIT8	enolase	XII	Facklamia hominis	4.2.1.11
K1LNU3	enolase	XII	Facklamia ignava	4.2.1.11
H3NHW3	enolase	XII	Facklamia languida	4.2.1.11
D4K9R5	enolase	XII	Faecalibacterium prausnitzii	4.2.1.11
U2PDE8	enolase	XII	Faecalitalea cylindroides	4.2.1.11
Q27655	enolase	XII	Fasciola hepatica	4.2.1.11
A0A098C263	enolase	XII	Fermentimonas caenicola	4.2.1.11
E1SSZ8	enolase	XII	Ferrimonas balearica	4.2.1.11
D3S034	enolase	XII	Ferroglobus placidus	4.2.1.11
S0AQW2	enolase	XII	Ferroplasma acidarmanus	4.2.1.11
A0A017RWF6	enolase	XII	Fervidicella metallireducens	4.2.1.11
A7HNS8	enolase	XII	Fervidobacterium nodosum	4.2.1.11
I0K517	enolase	XII	Fibrella aestuarina	4.2.1.11
I2GTI7	enolase	XII	Fibrisoma limi	4.2.1.11
A0A068NUK1	enolase	XII	Fimbriimonas ginsengisoli	4.2.1.11
R5ATB4	enolase	XII	Firmicutes bacterium	4.2.1.11
R5HC41	enolase	XII	Firmicutes bacterium	4.2.1.11
R5JC54	enolase	XII	Firmicutes bacterium	4.2.1.11
R5MWK4	enolase	XII	Firmicutes bacterium	4.2.1.11
R5QVH5	enolase	XII	Firmicutes bacterium	4.2.1.11
R5VDN6	enolase	XII	Firmicutes bacterium	4.2.1.11
R5XFF4	enolase	XII	Firmicutes bacterium	4.2.1.11
R6BTC5	enolase	XII	Firmicutes bacterium	4.2.1.11
R6ECB8	enolase	XII	Firmicutes bacterium	4.2.1.11
R6EU80	enolase	XII	Firmicutes bacterium	4.2.1.11
R6FJY7	enolase	XII	Firmicutes bacterium	4.2.1.11
R6I2H5	enolase	XII	Firmicutes bacterium	4.2.1.11
R6MHC3	enolase	XII	Firmicutes bacterium	4.2.1.11
R6QBM0	enolase	XII	Firmicutes bacterium	4.2.1.11
R6RE25	enolase	XII	Firmicutes bacterium	4.2.1.11
R6RTY5	enolase	XII	Firmicutes bacterium	4.2.1.11
R6V2Z8	enolase	XII	Firmicutes bacterium	4.2.1.11
R6XCB5	enolase	XII	Firmicutes bacterium	4.2.1.11
R6XJS3	enolase	XII	Firmicutes bacterium	4.2.1.11
R6ZM70	enolase	XII	Firmicutes bacterium	4.2.1.11
R6ZNA6	enolase	XII	Firmicutes bacterium	4.2.1.11
R7BVJ3	enolase	XII	Firmicutes bacterium	4.2.1.11
R7HIP5	enolase	XII	Firmicutes bacterium	4.2.1.11
R7IFC1	enolase	XII	Firmicutes bacterium	4.2.1.11
R7MZG8	enolase	XII	Firmicutes bacterium	4.2.1.11
R9LST9	enolase	XII	Firmicutes bacterium	4.2.1.11
G6FMZ6	enolase	XII	Fischerella sp.	4.2.1.11
A0A0C1IP37	enolase	XII	Flavihumibacter sp.	4.2.1.11
C0BMC4	enolase	XII	Flavobacteria bacterium	4.2.1.11
A0A0F2NWJ0	enolase	XII	Flavobacteriales bacterium	4.2.1.11
G8X6E9	enolase	XII	Flavobacterium columnare	4.2.1.11
A5FN12	enolase	XII	Flavobacterium johnsoniae	4.2.1.11
A6GZ69	enolase	XII	Flavobacterium psychrophilum	4.2.1.11
A0A0A2M026	enolase	XII	Flavobacterium rivuli	4.2.1.11
I4AMT3	enolase	XII	Flexibacter litoralis	4.2.1.11

Table S2.6. (continuation)

UniProt	Family [3]	Cluster	Species	EC Number
F8E6J0	enolase	XII	Flexistipes sinusarabici	4.2.1.11
F2II54	enolase	XII	Fluviicola taffensis	4.2.1.11
S8F7G9	enolase	XII	Fomitopsis pinicola	
A0A0D2IUS4	enolase	XII	Fonsecaea multimorphosa	
A0A0D2F1F6	enolase	XII	Fonsecaea pedrosoi	
A0A058ZFZ4	enolase	XII	Fonticula alba	
A0A0C9Q7U7	enolase	XII	Fopius arisanus	
T2KHI7	enolase	XII	Formosa agariphila	4.2.1.11
A0A0B6D2B9	enolase	XII	Francisella philomiragia	4.2.1.11
B0U1A7	enolase	XII	Francisella philomiragia	4.2.1.11
A0Q5J9	enolase	XII	Francisella tularensis	4.2.1.11
Q0RCG8	enolase	XII	Frankia alni	4.2.1.11
E3JCN4	enolase	XII	Frankia sp.	4.2.1.11
Q2J619	enolase	XII	Frankia sp.	4.2.1.11
F8AVT3	enolase	XII	Frankia symbiont	4.2.1.11
H8L060	enolase	XII	Frateuria aurantia	4.2.1.11
D4M8A9	enolase	XII	Fretibacterium fastidiosum	4.2.1.11
Q0FZ58	enolase	XII	Fulvimarina pelagi	4.2.1.11
L8JZQ8	enolase	XII	Fulvivirga imtechensis	4.2.1.11
A0A0C9MB86	enolase	XII	fungal sp.	
E5BDT5	enolase	XII	Fusobacterium gonidiaformans	4.2.1.11
C3WGA8	enolase	XII	Fusobacterium mortiferum	4.2.1.11
R7LVR3	enolase	XII	Fusobacterium sp.	4.2.1.11
M2X275	enolase	XII	Galdieria sulphuraria	4.2.1.11
P07322	enolase	XII	Gallus gallus	4.2.1.11
P51913	enolase	XII	Gallus gallus	4.2.1.11
B5JV92	enolase	XII	gamma proteobacterium	4.2.1.11
E1VNQ0	enolase	XII	gamma proteobacterium	4.2.1.11
F3L387	enolase	XII	gamma proteobacterium	4.2.1.11
F3LF82	enolase	XII	gamma proteobacterium	4.2.1.11
H3NSK4	enolase	XII	gamma proteobacterium	4.2.1.11
I2JN34	enolase	XII	gamma proteobacterium	4.2.1.11
A0A086D121	enolase	XII	Gammaproteobacteria bacterium	4.2.1.11
A0A0F2P456	enolase	XII	Gammaproteobacteria bacterium	4.2.1.11
W2UI02	enolase	XII	Gammaproteobacteria bacterium	4.2.1.11
K9SBS7	enolase	XII	Geitlerinema sp.	4.2.1.11
E5V3S4	enolase	XII	Gemella morbillorum	4.2.1.11
C1A7Y3	enolase	XII	Gemmatimonas aurantiaca	4.2.1.11
W0RM83	enolase	XII	Gemmatirosa kalamazoonesis	4.2.1.11
A0A0B5FRX3	enolase	XII	Geoalkalibacter subterraneus	4.2.1.11
C5D7M1	enolase	XII	Geobacillus sp.	4.2.1.11
B5EGF2	enolase	XII	Geobacter bemidjiensis	4.2.1.11
B3E2S8	enolase	XII	Geobacter lovleyi	4.2.1.11
Q39T27	enolase	XII	Geobacter metallireducens	4.2.1.11
Q74AR6	enolase	XII	Geobacter sulfurreducens	4.2.1.11
D2S939	enolase	XII	Geodermatophilus obscurus	4.2.1.11
A0A0A7GI52	enolase	XII	Geoglobus acetivorans	4.2.1.11
A0A061N4W6	enolase	XII	Geomicrobium sp.	4.2.1.11
A0A074Q3Q4	enolase	XII	Georgenia sp.	4.2.1.11
A0A066T8T3	enolase	XII	Gilliamella apicola	4.2.1.11
C1J0I5	enolase	XII	Gillichthys mirabilis	
H2BYB5	enolase	XII	Gillisia limnaea	4.2.1.11
A6C446	enolase	XII	Gimesia maris	4.2.1.11
G4QM37	enolase	XII	Glaciecola nitratireducens	4.2.1.11
H5TB25	enolase	XII	Glaciecola punicea	4.2.1.11
U5QN03	enolase	XII	Gloeobacter kilaueensis	4.2.1.11

Table S2.6. (continuation)

UniProt	Family [3]	Cluster	Species	EC Number
K9XFG9	enolase	XII	Gloeocapsa sp.	4.2.1.11
L8LHN6	enolase	XII	Gloeocapsa sp.	4.2.1.11
L8LQQ3	enolase	XII	Gloeocapsa sp.	4.2.1.11
S7S302	enolase	XII	Gloeophyllum trabeum	
D3TNV2	enolase	XII	Glossina morsitans	
A9HJ75	enolase	XII	Gluconacetobacter diazotrophicus	4.2.1.11
K7SJ92	enolase	XII	Gluconobacter oxydans	4.2.1.11
Q5FNN5	enolase	XII	Gluconobacter oxydans	4.2.1.11
G7GNZ4	enolase	XII	Gordonia amarae	4.2.1.11
G7H610	enolase	XII	Gordonia araii	4.2.1.11
L7LEA2	enolase	XII	Gordonia hirsuta	4.2.1.11
H5TJV0	enolase	XII	Gordonia otitidis	4.2.1.11
K6W8F1	enolase	XII	Gordonia rhizosphera	4.2.1.11
M0QJC8	enolase	XII	Gordonia soli	4.2.1.11
A0A0F2GDW3	enolase	XII	Gordonia sp.	4.2.1.11
W4VHZ2	enolase	XII	Gracilibacillus boraciitolerans	4.2.1.11
A0M568	enolase	XII	Gramella forsetii	4.2.1.11
A0A023B3P4	enolase	XII	Gregarina niphandrodes	4.2.1.11
A0A0C5VMY8	enolase	XII	Gynuella sunshinyii	4.2.1.11
A0A097CK68	enolase	XII	Haemaphysalis flava	
A0A086YH16	enolase	XII	Haematobacter missouriensis	4.2.1.11
E7QSE0	enolase	XII	Haladaptatus paucihalophilus	4.2.1.11
M5E3S6	enolase	XII	Halanaerobium sacc.	4.2.1.11
D0LHW3	enolase	XII	Haliangium ochraceum	4.2.1.11
F4KXV2	enolase	XII	Haliscomenobacter hydrossis	4.2.1.11
M0KMY1	enolase	XII	Haloarcula amylolytica	4.2.1.11
L5ND55	enolase	XII	Halobacillus sp.	4.2.1.11
E1X196	enolase	XII	Halobacteriovorax marinus	4.2.1.11
W0JZU1	enolase	XII	Halobacterium sp.	4.2.1.11
L0KBR5	enolase	XII	Halobacteroides halobius	4.2.1.11
M0LWX8	enolase	XII	Halococcus hamelinensis	4.2.1.11
M0MZC4	enolase	XII	Halococcus thailandensis	4.2.1.11
I3R8A0	enolase	XII	Haloferax mediterranei	4.2.1.11
M0G3Q0	enolase	XII	Haloferax prahovense	4.2.1.11
E4NRP9	enolase	XII	Halogeometricum borinquense	4.2.1.11
J2ZEH9	enolase	XII	Halogranum salarium	4.2.1.11
S2LDH8	enolase	XII	Halomonas anticariensis	4.2.1.11
W1N2Y2	enolase	XII	Halomonas huangheensis	4.2.1.11
A0A0B1PU71	enolase	XII	Halomonas hydrothermalis	4.2.1.11
H0J4Q7	enolase	XII	Halomonas sp.	4.2.1.11
U7NEP9	enolase	XII	Halomonas sp.	4.2.1.11
U1QJV7	enolase	XII	Halonotius sp.	4.2.1.11
G2MJ49	enolase	XII	halophilic archaeon	4.2.1.11
U1MYP6	enolase	XII	halophilic archaeon	4.2.1.11
U1PHL0	enolase	XII	halophilic archaeon	4.2.1.11
U1R597	enolase	XII	halophilic archaeon	4.2.1.11
U2EF66	enolase	XII	Haloplasma contractile	4.2.1.11
U1PWP7	enolase	XII	Haloquadratum sp.	4.2.1.11
F7PFF2	enolase	XII	Halorhabdus tiamatea	4.2.1.11
W8KT39	enolase	XII	Halorhodospira halochloris	4.2.1.11
A1WWZ2	enolase	XII	Halorhodospira halophila	4.2.1.11
M0PFH9	enolase	XII	Halorubrum aidingense	4.2.1.11
M0P402	enolase	XII	Halorubrum kocurii	4.2.1.11
B9LPW6	enolase	XII	Halorubrum lacusprofundi	4.2.1.11
M0NHD3	enolase	XII	Halorubrum lipolyticum	4.2.1.11
M0CQR5	enolase	XII	Halosimplex carlsbadense	4.2.1.11

Table S2.6. (continuation)

UniProt	Family [3]	Cluster	Species	EC Number
A0A063ZRU7	enolase	XII	Halostagnicola sp.	4.2.1.11
M0C3K2	enolase	XII	Haloterrigena salina	4.2.1.11
K9YDQ3	enolase	XII	Halothece sp.	4.2.1.11
B8CYF9	enolase	XII	Halothermothrix orenii	4.2.1.11
M0B9V9	enolase	XII	Halovivax asiaticus	4.2.1.11
A0A0F4XBK8	enolase	XII	Hanseniaspora uvarum	
A0A0C1XL44	enolase	XII	Hassallia byssoidea	4.2.1.11
H3NMX9	enolase	XII	Helcococcus kunzii	4.2.1.11
A0A099UKW9	enolase	XII	Helicobacter apodemus	4.2.1.11
C3XDL4	enolase	XII	Helicobacter bilis	4.2.1.11
F8KP10	enolase	XII	Helicobacter bizzozeronii	4.2.1.11
V8CLP3	enolase	XII	Helicobacter canis	4.2.1.11
I0ELQ0	enolase	XII	Helicobacter cetorum	4.2.1.11
E7ACM7	enolase	XII	Helicobacter felis	4.2.1.11
T1D3B9	enolase	XII	Helicobacter fennelliae	4.2.1.11
Q7VIH4	enolase	XII	Helicobacter hepaticus	4.2.1.11
V8C574	enolase	XII	Helicobacter macacae	4.2.1.11
A0A099TYB5	enolase	XII	Helicobacter muridarum	4.2.1.11
D3UG27	enolase	XII	Helicobacter mustelae	4.2.1.11
C5F0S2	enolase	XII	Helicobacter pullorum	4.2.1.11
Q9ZMS6	enolase	XII	Helicobacter pylori	4.2.1.11
A0A099B4X4	enolase	XII	Helicobacter sanguini	4.2.1.11
A0A099TJ78	enolase	XII	Helicobacter sp.	4.2.1.11
E7G345	enolase	XII	Helicobacter suis	4.2.1.11
C3XKI2	enolase	XII	Helicobacter winghamensis	4.2.1.11
A0A059LFH8	enolase	XII	Helicosporidium sp.	
B0TDD4	enolase	XII	Heliobacterium modesticaldum	4.2.1.11
B0TGK0	enolase	XII	Heliobacterium modesticaldum	4.2.1.11
T1FM52	enolase	XII	Helobdella robusta	
A9B5H3	enolase	XII	Herpetosiphon aurantiacus	4.2.1.11
Q5UU97	enolase	XII	Heterocapsa triquetra	
Q5WQL5	enolase	XII	Heterocapsa triquetra	
Q5WQM2	enolase	XII	Heterosigma akashiwo	
C6XJT3	enolase	XII	Hirschia baltica	4.2.1.11
A0A0F2Q4K2	enolase	XII	Hoeflea sp.	4.2.1.11
B7CCY3	enolase	XII	Holdemanella biformis	4.2.1.11
A3KBG7	enolase	XII	Holomastigotoides mirabile	
P56252	enolase	XII	Homarus gammarus	4.2.1.11
P09104	enolase	XII	Homo sapiens	4.2.1.11
P13929	enolase	XII	Homo sapiens	4.2.1.11
D3AIZ7	enolase	XII	Hungatella hathewayi	4.2.1.11
G5II31	enolase	XII	Hungatella hathewayi	4.2.1.11
A0A085WNR8	enolase	XII	Hyalangium minutum	4.2.1.11
I7Z928	enolase	XII	Hydrocarboniphaga effusa	4.2.1.11
B4U9X7	enolase	XII	Hydrogenobaculum sp.	4.2.1.11
A0A076HSP7	enolase	XII	Hymenobacter sp.	4.2.1.11
A0A0A7LNC9	enolase	XII	Hymenobacter sp.	4.2.1.11
V5SE62	enolase	XII	Hyphomicrobium nitrativorans	4.2.1.11
F8JCK9	enolase	XII	Hyphomicrobium sp.	4.2.1.11
A0A059DPK6	enolase	XII	Hyphomonas sp.	4.2.1.11
G9MUI4	enolase	XII	Hypocrea virens	
Q5R143	enolase	XII	Idiomarina loihiensis	4.2.1.11
A0A094J118	enolase	XII	Idiomarina salinarum	4.2.1.11
A0A094JAM1	enolase	XII	Idiomarina sp.	4.2.1.11
F7RWL2	enolase	XII	Idiomarina sp.	4.2.1.11
K2KDJ9	enolase	XII	Idiomarina xiamenensis	4.2.1.11

Table S2.6. (continuation)

UniProt	Family [3]	Cluster	Species	EC Number
I0ANE7	enolase	XII	Ignavibacterium album	4.2.1.11
E0SP60	enolase	XII	Ignisphaera aggregans	4.2.1.11
E3H8X7	enolase	XII	Ilyobacter polytropus	4.2.1.11
I0WDU7	enolase	XII	Imtechella halotolerans	4.2.1.11
A0A0A0D9L3	enolase	XII	Inquilinus limosus	4.2.1.11
A0A0A0DAM3	enolase	XII	Inquilinus limosus	4.2.1.11
E6SCQ2	enolase	XII	Intrasporangium calvum	4.2.1.11
W9GKF2	enolase	XII	Intrasporangium chromatireducens	4.2.1.11
W9G6H4	enolase	XII	Intrasporangium oryzae	4.2.1.11
F6FSP5	enolase	XII	Isoptericola variabilis	4.2.1.11
E8R0W0	enolase	XII	Isosphaera pallida	4.2.1.11
A0A067Q7E3	enolase	XII	Jaapia argillacea	
A0A067QHI3	enolase	XII	Jaapia argillacea	
A0A0D1EJA3	enolase	XII	Jannaschia aquimarina	4.2.1.11
Q28RE8	enolase	XII	Jannaschia sp.	4.2.1.11
W0V3S9	enolase	XII	Janthinobacterium agaricidamnosum	4.2.1.11
A6SXG3	enolase	XII	Janthinobacterium sp.	4.2.1.11
A0A0C2W1M4	enolase	XII	Jeotgalibacillus soli	4.2.1.11
A0A078M125	enolase	XII	Jeotgalicoccus sp.	4.2.1.11
G5GGW8	enolase	XII	Johnsonella ignava	4.2.1.11
C7R008	enolase	XII	Jonesia denitrificans	4.2.1.11
C9MA33	enolase	XII	Jonquetella anthropi	4.2.1.11
I3C6R3	enolase	XII	Joostella marina	4.2.1.11
Q4H4A4	enolase	XII	Karenia mikimotoi	
F9YAG3	enolase	XII	Ketogulonicigenium vulgare	4.2.1.11
A6W6X2	enolase	XII	Kineococcus radiotolerans	4.2.1.11
K6VCX4	enolase	XII	Kineosphaera limosa	4.2.1.11
A0A0D5WV27	enolase	XII	Klebsiella michiganensis	4.2.1.11
Q70CP7	enolase	XII	Kluyveromyces lactis	4.2.1.11
A0A0A0JI91	enolase	XII	Knoellia subterranea	4.2.1.11
M2XST0	enolase	XII	Kocuria palustris	4.2.1.11
A0A0A6VU11	enolase	XII	Kocuria polaris	4.2.1.11
B2GM13	enolase	XII	Kocuria rhizophila	4.2.1.11
B1L7C2	enolase	XII	Korarchaeum cryptofilum	4.2.1.11
Q1ISS7	enolase	XII	Koribacter versatilis	4.2.1.11
D2PRA8	enolase	XII	Kribbella flavida	4.2.1.11
D6TQI9	enolase	XII	Ktedonobacter racemifer	4.2.1.11
W6MN56	enolase	XII	Kuraishia capsulata	
W5VZ51	enolase	XII	Kutzneria albida	4.2.1.11
D5WR87	enolase	XII	Kyrpidia tusciae	4.2.1.11
C7NEU0	enolase	XII	Kytococcus sedentarius	4.2.1.11
C5DD59	enolase	XII	Lachancea thermotolerans	
W2VKI8	enolase	XII	Lachnoanaerobaculum sp.	4.2.1.11
E5XJ88	enolase	XII	Lachnospiraceae bacterium	4.2.1.11
F3AB78	enolase	XII	Lachnospiraceae bacterium	4.2.1.11
F3APT5	enolase	XII	Lachnospiraceae bacterium	4.2.1.11
F3BB65	enolase	XII	Lachnospiraceae bacterium	4.2.1.11
F7JP28	enolase	XII	Lachnospiraceae bacterium	4.2.1.11
F7K5N9	enolase	XII	Lachnospiraceae bacterium	4.2.1.11
F7KAW7	enolase	XII	Lachnospiraceae bacterium	4.2.1.11
R9JE99	enolase	XII	Lachnospiraceae bacterium	4.2.1.11
R9K6G9	enolase	XII	Lachnospiraceae bacterium	4.2.1.11
R9ML02	enolase	XII	Lachnospiraceae bacterium	4.2.1.11
W3ASK4	enolase	XII	Lachnospiraceae bacterium	4.2.1.11
F6GJX5	enolase	XII	Lacinutrix sp.	4.2.1.11
U2PBL5	enolase	XII	Lactobacillus brevis	4.2.1.11

Table S2.6. (continuation)

UniProt	Family [3]	Cluster	Species	EC Number
K6PNJ9	enolase	XII	Lactobacillus casei	4.2.1.11
K6S2Q1	enolase	XII	Lactobacillus casei	4.2.1.11
X0PQX3	enolase	XII	Lactobacillus composti	
X0QPT2	enolase	XII	Lactobacillus composti	
J3JBY7	enolase	XII	Lactobacillus coryniformis	4.2.1.11
V7HZN1	enolase	XII	Lactobacillus equi	4.2.1.11
W6T9X1	enolase	XII	Lactobacillus fabifermentans	4.2.1.11
B2GAM0	enolase	XII	Lactobacillus fermentum	4.2.1.11
W9EE11	enolase	XII	Lactobacillus florum	4.2.1.11
A0A0C1M5Z4	enolase	XII	Lactobacillus fructivorans	4.2.1.11
A0A087QB07	enolase	XII	Lactobacillus gasseri	4.2.1.11
Q042F4	enolase	XII	Lactobacillus gasseri	4.2.1.11
U4QN65	enolase	XII	Lactobacillus helveticus	4.2.1.11
A0A0A1GUM6	enolase	XII	Lactobacillus hokkaidonensis	4.2.1.11
A0A0A1GYU6	enolase	XII	Lactobacillus hokkaidonensis	4.2.1.11
Q74J64	enolase	XII	Lactobacillus johnsonii	4.2.1.11
A0A0F4LUJ6	enolase	XII	Lactobacillus mellifer	4.2.1.11
Q88VW2	enolase	XII	Lactobacillus plantarum	4.2.1.11
U2W411	enolase	XII	Lactobacillus plantarum	4.2.1.11
Q1WSY0	enolase	XII	Lactobacillus salivarius	4.2.1.11
G2KVF4	enolase	XII	Lactobacillus sanfranciscensis	4.2.1.11
U4TUU8	enolase	XII	Lactobacillus shenzhenensis	4.2.1.11
A0A023CVT0	enolase	XII	Lactobacillus sucicola	
V8AM72	enolase	XII	Lactococcus garvieae	
I7LQU9	enolase	XII	Lactococcus raffinolactis	4.2.1.11
C1DCC3	enolase	XII	Laribacter hongkongensis	4.2.1.11
H3B528	enolase	XII	Latimeria chalumnae	
E7RWL4	enolase	XII	Lautropia mirabilis	4.2.1.11
Q1MS78	enolase	XII	Lawsonia intracellularis	4.2.1.11
E4RY47	enolase	XII	Leadbetterella byssophila	4.2.1.11
A0A098G560	enolase	XII	Legionella fallonii	4.2.1.11
A0A0A8US63	enolase	XII	Legionella hackeliae	4.2.1.11
D3HKH5	enolase	XII	Legionella longbeachae	4.2.1.11
A0A078L1C8	enolase	XII	Legionella massiliensis	
A0A0A2SQN8	enolase	XII	Legionella norrlandica	4.2.1.11
W2V1Z2	enolase	XII	Legionella oakridgensis	4.2.1.11
Q5WV02	enolase	XII	Legionella pneumophila	4.2.1.11
Q6ADR6	enolase	XII	Leifsonia xyli	4.2.1.11
E9BC06	enolase	XII	Leishmania donovani	
U9VHX8	enolase	XII	Leptolyngbya sp.	4.2.1.11
H2CCC1	enolase	XII	Leptonema illini	4.2.1.11
B0S8S8	enolase	XII	Leptospira biflexa	4.2.1.11
Q72QZ8	enolase	XII	Leptospira interrogans	4.2.1.11
S3UKW8	enolase	XII	Leptospira wolffii	4.2.1.11
C6HUW6	enolase	XII	Leptospirillum ferrodiazotrophum	4.2.1.11
I0IQK8	enolase	XII	Leptospirillum ferrooxidans	4.2.1.11
T0Z6W3	enolase	XII	Leptospirillum sp.	4.2.1.11
A0A0D0ILK4	enolase	XII	Leucobacter komagatae	4.2.1.11
A0A061LV15	enolase	XII	Leucobacter sp.	4.2.1.11
B1MVW3	enolase	XII	Leuconostoc citreum	4.2.1.11
L0EXJ6	enolase	XII	Liberibacter crescens	4.2.1.11
E4UCY0	enolase	XII	Liberibacter solanacearum	4.2.1.11
A0A077WIU9	enolase	XII	Lichtheimia ramosa	
A0A077X407	enolase	XII	Lichtheimia ramosa	
A6GUX2	enolase	XII	Limnobacter sp.	4.2.1.11
C1KY94	enolase	XII	Listeria monocytogenes	4.2.1.11

Table S2.6. (continuation)

UniProt	Family [3]	Cluster	Species	EC Number
S9QHU9	enolase	XII	Litoreibacter arenae	4.2.1.11
E1FJ38	enolase	XII	Loa loa	
U2Z533	enolase	XII	Loktanella cinnabarina	4.2.1.11
A3V4E1	enolase	XII	Loktanella vestfoldensis	4.2.1.11
B8KSZ0	enolase	XII	Luminiphilus syltensis	4.2.1.11
A0A0F3L0S1	enolase	XII	Luteibacter yeojuensis	4.2.1.11
V4QUT5	enolase	XII	Lutibaculum baratangense	4.2.1.11
A8CWB5	enolase	XII	Lutzomyia longipalpis	
U7QCP1	enolase	XII	Lyngbya aestuarii	4.2.1.11
A0A0C1UVE5	enolase	XII	Lyngbya confervoides	4.2.1.11
A0A0C1Y4Y6	enolase	XII	Lyngbya confervoides	4.2.1.11
A0A087N0E0	enolase	XII	Lysinibacillus sp.	4.2.1.11
A0A021VXS0	enolase	XII	Lysobacter capsici	4.2.1.11
A0A0A0ETP2	enolase	XII	Lysobacter concretionis	4.2.1.11
A0A0A0EYR2	enolase	XII	Lysobacter daejeonensis	4.2.1.11
A0A0A0M6T4	enolase	XII	Lysobacter defluvii	4.2.1.11
A0A0A2WHH3	enolase	XII	Lysobacter dokdonensis	4.2.1.11
B9EAH1	enolase	XII	Macrococcus caseolyticus	4.2.1.11
D3QZG1	enolase	XII	Mageeibacillus indolicus	4.2.1.11
A0L7X8	enolase	XII	Magnetococcus marinus	4.2.1.11
W6KAM2	enolase	XII	Magnetospira sp.	4.2.1.11
A4TXZ6	enolase	XII	Magnetospirillum gryph.	4.2.1.11
Q2W698	enolase	XII	Magnetospirillum magneticum	4.2.1.11
F3ZXZ8	enolase	XII	Mahella australiensis	4.2.1.11
M5EJ19	enolase	XII	Malassezia sympodialis	
A0A093RU69	enolase	XII	Manacus vitellinus	
A0A071LNL4	enolase	XII	Mangrovibacter sp.	4.2.1.11
A0A084TIF9	enolase	XII	Mangrovimonas yunxiaonensis	4.2.1.11
A4AM70	enolase	XII	Maribacter sp.	4.2.1.11
Q0APT2	enolase	XII	Maricaulis maris	4.2.1.11
W0E1A3	enolase	XII	Marichromatium purpuratum	4.2.1.11
A0A0C1RDX7	enolase	XII	marine actinobacterium	4.2.1.11
A0A0C1RHC1	enolase	XII	marine actinobacterium	4.2.1.11
A0A0C1UAZ3	enolase	XII	marine actinobacterium	4.2.1.11
A0YEP0	enolase	XII	marine gamma	4.2.1.11
A0Z6I9	enolase	XII	marine gamma	4.2.1.11
B7RY52	enolase	XII	marine gamma	4.2.1.11
A0A087S4E8	enolase	XII	Marine Group	4.2.1.11
F2NNF8	enolase	XII	Marinithermus hydrothermalis	4.2.1.11
H2J683	enolase	XII	Marinitoga piezophila	4.2.1.11
A6F5H3	enolase	XII	Marinobacter algicola	4.2.1.11
N6W2Z6	enolase	XII	Marinobacter nanhaiticus	4.2.1.11
A0A081G0Q4	enolase	XII	Marinobacterium sp.	4.2.1.11
A6VUU9	enolase	XII	Marinomonas sp.	4.2.1.11
Q0F060	enolase	XII	Mariprofundus ferrooxydans	4.2.1.11
A3VDS5	enolase	XII	Maritimibacter alkaliphilus	4.2.1.11
E4TU15	enolase	XII	Marivirga tractuosa	4.2.1.11
A0A0D5LT59	enolase	XII	Martelella endophytica	4.2.1.11
A0A098U1T5	enolase	XII	Massilia sp.	4.2.1.11
V5SHY9	enolase	XII	Mayetiola destructor	
H3K631	enolase	XII	Megamonas funiformis	4.2.1.11
R7N0U2	enolase	XII	Megasphaera elsdenii	4.2.1.11
E2Z9F0	enolase	XII	Megasphaera micronuciformis	4.2.1.11
F9MME4	enolase	XII	Megasphaera sp.	4.2.1.11
D3PKA4	enolase	XII	Meiothermus ruber	4.2.1.11
F4S6B5	enolase	XII	Melampsora larici-populina	

Table S2.6. (continuation)

UniProt	Family [3]	Cluster	Species	EC Number
A0A077R5F2	enolase	XII	Melanopsichium pennsylvanicum	
I6ZX08	enolase	XII	Melioribacter roseus	4.2.1.11
A0A0D1PWH0	enolase	XII	Mesorhizobium sp.	4.2.1.11
N1JQX1	enolase	XII	Mesotoga infera	4.2.1.11
F4G0P8	enolase	XII	Metallosphaera cuprina	4.2.1.11
A4YHC1	enolase	XII	Metallosphaera sedula	4.2.1.11
H2C8A7	enolase	XII	Metallosphaera yellowstonensis	4.2.1.11
A0A089ZG82	enolase	XII	Methanobacterium formicicum	4.2.1.11
K2RT51	enolase	XII	Methanobacterium formicicum	4.2.1.11
D3E2K4	enolase	XII	Methanobrevibacter ruminantium	4.2.1.11
R7PWD5	enolase	XII	Methanobrevibacter smithii	4.2.1.11
R9SK87	enolase	XII	Methanobrevibacter sp.	4.2.1.11
Q60173	enolase	XII	Methanocaldococcus jannaschii	4.2.1.11
H8I5H5	enolase	XII	Methanocella conradii	4.2.1.11
H8IB05	enolase	XII	Methanocella conradii	4.2.1.11
A0A0E3SSN9	enolase	XII	Methanococcoides methylutens	4.2.1.11
A6UUM2	enolase	XII	Methanococcus aeolicus	4.2.1.11
Q6M075	enolase	XII	Methanococcus maripaludis	4.2.1.11
A6US08	enolase	XII	Methanococcus vannielii	4.2.1.11
D7DR37	enolase	XII	Methanococcus voltae	4.2.1.11
A2SSV1	enolase	XII	Methanocorpusculum labreanum	4.2.1.11
I7LJ04	enolase	XII	Methanoculleus bourgensis	4.2.1.11
A0A063Z9M4	enolase	XII	Methanoculleus sp.	4.2.1.11
A0A063ZCG2	enolase	XII	Methanoculleus sp.	4.2.1.11
R7Q107	enolase	XII	Methanoculleus sp.	4.2.1.11
J1L1N3	enolase	XII	Methanofollis liminatans	4.2.1.11
D7E7F1	enolase	XII	Methanohalobium evestigatum	4.2.1.11
D5E8J9	enolase	XII	Methanohalophilus mahii	4.2.1.11
K4MDI4	enolase	XII	Methanolobus psychrophilus	4.2.1.11
W9DQ37	enolase	XII	Methanolobus tindarius	4.2.1.11
L0KT74	enolase	XII	Methanomethylovorans hollandica	4.2.1.11
H1YWY1	enolase	XII	Methanoplanus limicola	4.2.1.11
Q8TUV6	enolase	XII	Methanopyrus kandleri	4.2.1.11
A7IAG9	enolase	XII	Methanoregula boonei	4.2.1.11
L0H9Y6	enolase	XII	Methanoregula formicica	4.2.1.11
F4BUI5	enolase	XII	Methanosaeta concilii	4.2.1.11
F7XPE7	enolase	XII	Methanosalsum zhilinae	4.2.1.11
B8GEF9	enolase	XII	Methanosphaerula palustris	4.2.1.11
Q2FLB5	enolase	XII	Methanospirillum hungatei	4.2.1.11
Q2FQL9	enolase	XII	Methanospirillum hungatei	4.2.1.11
Q2FTN3	enolase	XII	Methanospirillum hungatei	4.2.1.11
E3GZF8	enolase	XII	Methanothermus fervidus	4.2.1.11
F6BD08	enolase	XII	Methanotorris igneus	4.2.1.11
A0A0C1URV1	enolase	XII	Methylacidiphilum kamchatkense	4.2.1.11
G3J1L7	enolase	XII	Methylobacter tundripaludum	4.2.1.11
A9W6G9	enolase	XII	Methylobacterium extorquens	4.2.1.11
M7YB91	enolase	XII	Methylobacterium mesophilicum	4.2.1.11
B8IXI8	enolase	XII	Methylobacterium nodulans	4.2.1.11
B1LZU6	enolase	XII	Methylobacterium radiotolerans	4.2.1.11
B0UHJ8	enolase	XII	Methylobacterium sp.	4.2.1.11
B0UPU6	enolase	XII	Methylobacterium sp.	4.2.1.11
A0A0A8K3P2	enolase	XII	Methyloceanibacter caenitepidi	4.2.1.11
B8EJT3	enolase	XII	Methylocella silvestris	4.2.1.11
Q604M4	enolase	XII	Methylococcus capsulatus	4.2.1.11
Q606T2	enolase	XII	Methylococcus capsulatus	4.2.1.11
J7Q535	enolase	XII	Methylocystis sp.	4.2.1.11

Table S2.6. (continuation)

UniProt	Family [3]	Cluster	Species	EC Number
V5BSB2	enolase	XII	Methyloglobulus morosus	4.2.1.11
H8GK88	enolase	XII	Methylomicrobium album	4.2.1.11
G4SXV6	enolase	XII	Methylomicrobium alcaliphilum	4.2.1.11
G0A6Y6	enolase	XII	Methylomonas methanica	4.2.1.11
F5SVN4	enolase	XII	Methylophaga aminisulfidivorans	4.2.1.11
I1YEX5	enolase	XII	Methylophaga frappieri	4.2.1.11
M7PPS2	enolase	XII	Methylophaga lonarensis	4.2.1.11
I1XIX9	enolase	XII	Methylophaga nitratireducenticrescens	4.2.1.11
A0P7D6	enolase	XII	Methylophilales bacterium	4.2.1.11
C6WX05	enolase	XII	Methylotenera mobilis	4.2.1.11
A5DM90	enolase	XII	Meyerozyma guilliermondii	
G2KSN0	enolase	XII	Micavibrio aeruginosavorus	4.2.1.11
A0A0B2AA15	enolase	XII	Microbacterium mangrovi	4.2.1.11
A0A031FQS7	enolase	XII	Microbacterium oleivorans	4.2.1.11
A0A062VQ89	enolase	XII	Microbacterium sp.	4.2.1.11
W0ZCH2	enolase	XII	Microbacterium sp.	4.2.1.11
E8NAU3	enolase	XII	Microbacterium testaceum	4.2.1.11
W2EJ86	enolase	XII	Microbispora sp.	4.2.1.11
U5HBY9	enolase	XII	Microbotryum lychnidis-dioicae	
C5C987	enolase	XII	Micrococcus luteus	4.2.1.11
D3LL73	enolase	XII	Micrococcus luteus	4.2.1.11
K9WE79	enolase	XII	Microcoleus sp.	4.2.1.11
B0JMP6	enolase	XII	Microcystis aeruginosa	4.2.1.11
I4G0A3	enolase	XII	Microcystis aeruginosa	4.2.1.11
I0LD61	enolase	XII	Micromonospora lupini	4.2.1.11
I4Z0G7	enolase	XII	Microvirga lotononidis	4.2.1.11
F7XUS2	enolase	XII	Midichloria mitochondrii	4.2.1.11
U2TB12	enolase	XII	Mitsuokella sp.	4.2.1.11
H5USC7	enolase	XII	Mobilicoccus pelagius	4.2.1.11
A0A098Y1Y9	enolase	XII	Modestobacter sp.	4.2.1.11
X8IR53	enolase	XII	Mogibacterium timidum	4.2.1.11
A0A088T1K0	enolase	XII	Mollicutes bacterium	4.2.1.11
A0A094ILP7	enolase	XII	Mollicutes bacterium	4.2.1.11
F8UGT4	enolase	XII	Moniezia expansa	4.2.1.11
A0A0D2MAX0	enolase	XII	Monoraphidium neglectum	4.2.1.11
F4XZP8	enolase	XII	Moorea producens	4.2.1.11
Q2RLT8	enolase	XII	Moorella thermoacetica	4.2.1.11
F7XXM6	enolase	XII	Moranella endobia	4.2.1.11
A0A066UKL8	enolase	XII	Moraxella bovoculi	4.2.1.11
A0A076U0T1	enolase	XII	Moraxella catarrhalis	4.2.1.11
L2FBF4	enolase	XII	Moraxella macacae	4.2.1.11
W9R689	enolase	XII	Morus notabilis	
A0A060R640	enolase	XII	Mucinivorans hirudinis	4.2.1.11
V2RHN6	enolase	XII	Mucispirillum schaedleri	4.2.1.11
S2K9S5	enolase	XII	Mucor circinelloides	
A0A0B2AWS9	enolase	XII	Mumia flava	4.2.1.11
A0A0B2BGD2	enolase	XII	Mumia flava	4.2.1.11
A0A089T2F0	enolase	XII	Mycobacterium abscessus	4.2.1.11
A0A064C9N0	enolase	XII	Mycobacterium aromaticivorans	4.2.1.11
A0A0E3XMK3	enolase	XII	Mycobacterium chelonae	4.2.1.11
X7YWJ8	enolase	XII	Mycobacterium kansasii	4.2.1.11
Q9CD42	enolase	XII	Mycobacterium leprae	4.2.1.11
L0J537	enolase	XII	Mycobacterium smegmatis	4.2.1.11
A0A081I4Y0	enolase	XII	Mycobacterium sp.	4.2.1.11
A3Q5F7	enolase	XII	Mycobacterium sp.	4.2.1.11
A1TEE4	enolase	XII	Mycobacterium vanbaalenii	4.2.1.11

Table S2.6. (continuation)

UniProt	Family [3]	Cluster	Species	EC Number
I0S024	enolase	XII	Mycobacterium xenopi	4.2.1.11
E4QSS3	enolase	XII	Mycoplasma hyopneumoniae	4.2.1.11
A0A014M301	enolase	XII	Mycoplasma ovipneumoniae	4.2.1.11
R5M356	enolase	XII	Mycoplasma sp.	4.2.1.11
R5YE77	enolase	XII	Mycoplasma sp.	4.2.1.11
R7NG06	enolase	XII	Mycoplasma sp.	4.2.1.11
S7NI64	enolase	XII	Myotis brandtii	
H1ZB37	enolase	XII	Myroides odoratus	4.2.1.11
Q1D401	enolase	XII	Myxococcus xanthus	4.2.1.11
C8XKR0	enolase	XII	Nakamurella multipartita	4.2.1.11
W7TR29	enolase	XII	Nannochloropsis gaditana	
B2A6Z1	enolase	XII	Natranaerobius thermophilus	4.2.1.11
M0AUF8	enolase	XII	Natrialba asiatica	4.2.1.11
M0AAF6	enolase	XII	Natrialba taiwanensis	4.2.1.11
M1XRK5	enolase	XII	Natronomonas moolapensis	4.2.1.11
L9WT60	enolase	XII	Natronorubrum bangense	4.2.1.11
G0W891	enolase	XII	Naumovozyma dairenensis	
B9L7U3	enolase	XII	Nautilia profundicola	4.2.1.11
A1KUB6	enolase	XII	Neisseria meningitidis	4.2.1.11
G4CJW3	enolase	XII	Neisseria shayeganii	4.2.1.11
L1NT61	enolase	XII	Neisseria sp.	4.2.1.11
A0A0C1KAS2	enolase	XII	Neochlamydia sp.	4.2.1.11
X5GX54	enolase	XII	Neorickettsia helminthoeca	4.2.1.11
A0A0B0DPS1	enolase	XII	Neurospora crassa	
Q7RV85	enolase	XII	Neurospora crassa	4.2.1.11
W0F2Q2	enolase	XII	Niabella soli	4.2.1.11
G8TIM5	enolase	XII	Niastella koreensis	4.2.1.11
V5RDY5	enolase	XII	Nilaparvata lugens	4.2.1.11
E6WZ20	enolase	XII	Nitratifractor salsuginis	4.2.1.11
E6X3F5	enolase	XII	Nitratifractor salsuginis	4.2.1.11
A0A084UAJ0	enolase	XII	Nitratireductor basaltis	4.2.1.11
K2MC11	enolase	XII	Nitratireductor pacificus	4.2.1.11
A6Q5K4	enolase	XII	Nitratiruptor sp.	4.2.1.11
A0A063Y7Z5	enolase	XII	Nitrincola lacisaponensis	4.2.1.11
I5CA02	enolase	XII	Nitritalea halalkaliphila	4.2.1.11
Q1QMI9	enolase	XII	Nitrobacter hamburgensis	4.2.1.11
Q0AD93	enolase	XII	Nitrosomonas eutropha	4.2.1.11
F8GLQ9	enolase	XII	Nitrosomonas sp.	4.2.1.11
K0IE56	enolase	XII	Nitrososphaera gargensis	4.2.1.11
A0A060HIS3	enolase	XII	Nitrososphaera viennensis	4.2.1.11
Q2Y9P0	enolase	XII	Nitrosospira multiformis	4.2.1.11
D8PH76	enolase	XII	Nitrospira defluvii	4.2.1.11
Q5YQ30	enolase	XII	Nocardia farcinica	4.2.1.11
W5TJA6	enolase	XII	Nocardia nova	4.2.1.11
W5TK55	enolase	XII	Nocardia nova	4.2.1.11
A0A0F2GCC7	enolase	XII	Nocardioides luteus	4.2.1.11
A0A0A1DJT2	enolase	XII	Nocardioides simplex	4.2.1.11
A1SF66	enolase	XII	Nocardioides sp.	4.2.1.11
D7B6W7	enolase	XII	Nocardiopsis dassonvillei	4.2.1.11
W8VVW2	enolase	XII	Nonlabens marinus	4.2.1.11
A0A090Q573	enolase	XII	Nonlabens sediminis	4.2.1.11
A0A081DCG1	enolase	XII	Nonlabens ulvanivorans	4.2.1.11
T0L7Z5	enolase	XII	Nosema apis	
B2J1R2	enolase	XII	Nostoc punctiforme	4.2.1.11
Q2G662	enolase	XII	Novosphingobium aromat.	4.2.1.11
G6EFB7	enolase	XII	Novosphingobium pentarom.	4.2.1.11

Table S2.6. (continuation)

UniProt	Family [3]	Cluster	Species	EC Number
K2KK29	enolase	XII	Oceanibaculum indicum	4.2.1.11
A3UCP8	enolase	XII	Oceanicaulis sp.	4.2.1.11
H2FUW2	enolase	XII	Oceanimonas sp.	4.2.1.11
K2HMW8	enolase	XII	Oceaniovalibus guishaninsula	4.2.1.11
E4U5G6	enolase	XII	Oceanithermus profundus	4.2.1.11
Q8ENP5	enolase	XII	Oceanobacillus iheyensis	4.2.1.11
A6X7S5	enolase	XII	Ochrobactrum anthropi	4.2.1.11
M9R5E5	enolase	XII	Octadecabacter antarcticus	4.2.1.11
R5P1E2	enolase	XII	Odoribacter sp.	4.2.1.11
R6FFC2	enolase	XII	Odoribacter splanchnicus	4.2.1.11
A0A0A0M417	enolase	XII	Oenococcus alcoholitolerans	4.2.1.11
Q04DH2	enolase	XII	Oenococcus oeni	4.2.1.11
W1QIU6	enolase	XII	Ogataea parapolymorpha	4.2.1.11
E4XB96	enolase	XII	Oikopleura dioica	
A0A099CUD4	enolase	XII	Oleiagrimonas soli	4.2.1.11
R4YQ33	enolase	XII	Oleispira antarctica	4.2.1.11
A0A095ZDE3	enolase	XII	Oligella urethralis	4.2.1.11
B6JFY2	enolase	XII	Oligotropha carboxidovorans	4.2.1.11
U2V4P1	enolase	XII	Olsenella profusa	4.2.1.11
G5F186	enolase	XII	Olsenella sp.	4.2.1.11
E1QYM1	enolase	XII	Olsenella uli	4.2.1.11
Q5XXS5	enolase	XII	Oncometopia nigricans	
A0A074ZWN8	enolase	XII	Opisthorchis viverrini	
W0IZK2	enolase	XII	Opitutaceae bacterium	4.2.1.11
B1ZV17	enolase	XII	Opitutus terrae	4.2.1.11
A0A0B2UFE8	enolase	XII	Ordospora colligata	
I3JIW3	enolase	XII	Oreochromis niloticus	
I3K5B6	enolase	XII	Oreochromis niloticus	
F5T6L3	enolase	XII	Oribacterium sp.	4.2.1.11
I3ZY69	enolase	XII	Ornithobacterium rhinotracheale	4.2.1.11
B9F777	enolase	XII	Oryza sativa	
B9G3A0	enolase	XII	Oryza sativa	
K9VLP7	enolase	XII	Oscillatoria nigro-viridis	4.2.1.11
K8GDL8	enolase	XII	Oscillatoriales cyanobacterium	4.2.1.11
E1IHR1	enolase	XII	Oscillochloris trichoides	4.2.1.11
S6H7L9	enolase	XII	Osedax symbiont	4.2.1.11
G8R2N1	enolase	XII	Owenweeksia hongkongensis	4.2.1.11
C3X9M3	enolase	XII	Oxalobacter formigenes	4.2.1.11
W2EEN7	enolase	XII	Paenibacillus larvae	4.2.1.11
A0A090Y4U5	enolase	XII	Paenibacillus macerans	4.2.1.11
C6J3Q6	enolase	XII	Paenibacillus sp.	4.2.1.11
A0A086XZW6	enolase	XII	Paenirhodobacter enshiensis	4.2.1.11
S2XEK7	enolase	XII	Paenisporosarcina sp.	4.2.1.11
A0A075LXL2	enolase	XII	Palaeococcus pacificus	4.2.1.11
E4T3A2	enolase	XII	Paludibacter propionicigenes	4.2.1.11
I4DJA8	enolase	XII	Papilio xuthus	
A0A0F5IJZ5	enolase	XII	Parabacteroides sp.	4.2.1.11
F8L277	enolase	XII	Parachlamydia acanthamoebae	4.2.1.11
A1B9D2	enolase	XII	Paracoccus denitrificans	4.2.1.11
K7AKD0	enolase	XII	Paraglaciecola psychrophila	4.2.1.11
A0BCX6	enolase	XII	Paramecium tetraurelia	
H6SPX4	enolase	XII	Pararhodospirillum photometricum	4.2.1.11
V7PX37	enolase	XII	Parcubacteria bacterium	4.2.1.11
F5TAP9	enolase	XII	Parvimonas sp.	4.2.1.11
E0TEH6	enolase	XII	Parvularcula bermudensis	4.2.1.11
H0EAP6	enolase	XII	Patulibacter medicamentivorans	4.2.1.11

Table S2.6. (continuation)

UniProt	Family [3]	Cluster	Species	EC Number
G8PAE8	enolase	XII	Pediococcus claussenii	4.2.1.11
A0A0F3G0K0	enolase	XII	Pediococcus damnosus	4.2.1.11
Q03GW5	enolase	XII	Pediococcus pentosaceus	4.2.1.11
A0A081PH15	enolase	XII	Pedobacter antarcticus	4.2.1.11
A0A0B8XTS5	enolase	XII	Pedobacter glucosidilyticus	4.2.1.11
A0A0C1D6Y7	enolase	XII	Pedobacter kyungheensis	4.2.1.11
A0A0D0FXK1	enolase	XII	Pedobacter sp.	4.2.1.11
A6EJ08	enolase	XII	Pedobacter sp.	4.2.1.11
W6TLG9	enolase	XII	Pedobacter sp.	4.2.1.11
B9XJE7	enolase	XII	Pedosphaera parvula	4.2.1.11
Q0FHW9	enolase	XII	Pelagibaca bermudensis	4.2.1.11
F2I123	enolase	XII	Pelagibacter sp.	4.2.1.11
G4R8Y6	enolase	XII	Pelagibacterium halotolerans	4.2.1.11
V8G3B6	enolase	XII	Pelistega indica	4.2.1.11
Q3A578	enolase	XII	Pelobacter carbinolicus	4.2.1.11
B4SGB2	enolase	XII	Pelodictyon phaeoclathratiforme	4.2.1.11
A0A075KIR3	enolase	XII	Pelosinus sp.	4.2.1.11
Q76KF9	enolase	XII	Penicillium chrysogenum	4.2.1.11
A0A0A2KMQ1	enolase	XII	Penicillium italicum	
W6PV89	enolase	XII	Penicillium roqueforti	
Q181T5	enolase	XII	Peptoclostridium difficile	4.2.1.11
A0A086YPT0	enolase	XII	Peptococcaceae bacterium	4.2.1.11
A0A0F2NHE9	enolase	XII	Peptococcaceae bacterium	4.2.1.11
A0A0F2PM27	enolase	XII	Peptococcaceae bacterium	4.2.1.11
E4KXJ8	enolase	XII	Peptoniphilus harei	4.2.1.11
G4D131	enolase	XII	Peptoniphilus indolicus	4.2.1.11
A0A095ZHX1	enolase	XII	Peptoniphilus lacrimalis	4.2.1.11
A0A090HYJ5	enolase	XII	Peptoniphilus sp.	4.2.1.11
D7N5D9	enolase	XII	Peptoniphilus sp.	4.2.1.11
U7UTH3	enolase	XII	Peptoniphilus sp.	4.2.1.11
J5H1N5	enolase	XII	Peptostreptococcaceae bacterium	4.2.1.11
U2J0D0	enolase	XII	Peptostreptococcaceae bacterium	4.2.1.11
V9HNB6	enolase	XII	Peptostreptococcaceae bacterium	4.2.1.11
C5KJ81	enolase	XII	Perkinsus marinus	
C5LEZ8	enolase	XII	Perkinsus marinus	
A9BIS7	enolase	XII	Petrotoga mobilis	4.2.1.11
A0A098S5M9	enolase	XII	Phaeodactylibacter xiamenensis	4.2.1.11
B7S3N7	enolase	XII	Phaeodactylum tricornutum	
Q5WQL9	enolase	XII	Phaeodactylum tricornutum	
S9S8X7	enolase	XII	Phaeospirillum fulvum	4.2.1.11
R6HRU5	enolase	XII	Phascolarctobacterium sp.	4.2.1.11
E8LF30	enolase	XII	Phascolarctobacterium succinatutens	4.2.1.11
A0A0B9G1G9	enolase	XII	Photobacterium gaetbulicola	4.2.1.11
A0A0B9H1N4	enolase	XII	Photobacterium gaetbulicola	4.2.1.11
A0A0B7JHU5	enolase	XII	Photobacterium phosphoreum	4.2.1.11
Q6LMT1	enolase	XII	Photobacterium profundum	4.2.1.11
I0IGS0	enolase	XII	Phycisphaera mikurensis	4.2.1.11
A9RBK0	enolase	XII	Physcomitrella patens	
W6KT45	enolase	XII	Phytomonas sp.	
G5AIQ2	enolase	XII	Phytophthora sojae	
B1V8R6	enolase	XII	Phytoplasma australiense	4.2.1.11
B8LQR0	enolase	XII	Picea sitchensis	
A0A099NYL7	enolase	XII	Pichia kudriavzevii	
A9YTT1	enolase	XII	Pichia kudriavzevii	4.2.1.11
G8YLG8	enolase	XII	Pichia sorbitophila	
Q6KZN3	enolase	XII	Picrophilus torridus	4.2.1.11

Table S2.6. (continuation)

UniProt	Family [3]	Cluster	Species	EC Number
D2R8I8	enolase	XII	Pirellula staleyi	4.2.1.11
A0A076K4Y8	enolase	XII	Planktomarina temperata	4.2.1.11
A0A073CLH7	enolase	XII	Planktothrix agardhii	4.2.1.11
E7REW5	enolase	XII	Planococcus donghaensis	4.2.1.11
Q8IJN7	enolase	XII	Plasmodium falciparum	4.2.1.11
I2FKC3	enolase	XII	Plasmodium gallinaceum	
Q7RA60	enolase	XII	Plasmodium yoelii	4.2.1.11
A6FXX9	enolase	XII	Plesiocystis pacifica	4.2.1.11
K9SZT4	enolase	XII	Pleurocapsa sp.	4.2.1.11
M7NNH8	enolase	XII	Pneumocystis murina	
A0A099Y2S4	enolase	XII	Polaribacter sp.	4.2.1.11
F2IZT1	enolase	XII	Polymorphum gilvum	4.2.1.11
A4SXE9	enolase	XII	Polynucleobacter necessarius	4.2.1.11
A0A0A5HRG8	enolase	XII	Pontibacillus litoralis	4.2.1.11
J0LN27	enolase	XII	Pontibacter sp.	4.2.1.11
A0A0B2BWH6	enolase	XII	Porphyrobacter mercurialis	4.2.1.11
A0A0A2EJY2	enolase	XII	Porphyromonadaceae bacterium	4.2.1.11
F4KMN9	enolase	XII	Porphyromonas asaccharolytica	4.2.1.11
A0A0A2EWC3	enolase	XII	Porphyromonas cangingivalis	4.2.1.11
T1CQX7	enolase	XII	Porphyromonas crevioricanis	4.2.1.11
B2RLL7	enolase	XII	Porphyromonas gingivalis	4.2.1.11
A0A0A2GCM0	enolase	XII	Porphyromonas gingivicanis	4.2.1.11
A0A0A2G8L6	enolase	XII	Porphyromonas macacae	4.2.1.11
A0A0A2DUR9	enolase	XII	Porphyromonas sp.	4.2.1.11
A0A0A2F9N7	enolase	XII	Porphyromonas sp.	4.2.1.11
R5GHJ0	enolase	XII	Porphyromonas sp.	4.2.1.11
U2IY81	enolase	XII	Porphyromonas sp.	4.2.1.11
F8N9A5	enolase	XII	Prevotella multisaccharivorax	4.2.1.11
L1MIJ4	enolase	XII	Prevotella sp.	4.2.1.11
R5AIE4	enolase	XII	Prevotella sp.	4.2.1.11
R5GH82	enolase	XII	Prevotella sp.	4.2.1.11
R5M8U9	enolase	XII	Prevotella sp.	4.2.1.11
R7F6F1	enolase	XII	Prevotella sp.	4.2.1.11
A0A0F5C9C9	enolase	XII	Pristionchus pacificus	
A2C038	enolase	XII	Prochlorococcus marinus	4.2.1.11
A9BDH2	enolase	XII	Prochlorococcus marinus	4.2.1.11
Q7V377	enolase	XII	Prochlorococcus marinus	4.2.1.11
Q7V483	enolase	XII	Prochlorococcus marinus	4.2.1.11
Q7VDY0	enolase	XII	Prochlorococcus marinus	4.2.1.11
A0A0A2BJ39	enolase	XII	Prochlorococcus sp.	4.2.1.11
A0A0A2BU21	enolase	XII	Prochlorococcus sp.	4.2.1.11
U2QBN0	enolase	XII	Propionibacterium acidifaciens	4.2.1.11
K7RLP8	enolase	XII	Propionibacterium acidipropionici	4.2.1.11
K7RNU4	enolase	XII	Propionibacterium acidipropionici	4.2.1.11
Q6AAB8	enolase	XII	Propionibacterium acnes	4.2.1.11
Q3LFH6	enolase	XII	Propionibacterium freudenreichii	4.2.1.11
I6X6A1	enolase	XII	Propionibacterium propionicum	4.2.1.11
S3XDK5	enolase	XII	Propionibacterium sp.	4.2.1.11
U7JLC4	enolase	XII	Propionibacterium sp.	4.2.1.11
S2W2A4	enolase	XII	Propionimicrobium lymphophilum	4.2.1.11
B4S487	enolase	XII	Prosthecochloris aestuarii	4.2.1.11
Q6MEY2	enolase	XII	Protochlamydia amoebophila	4.2.1.11
K9SGH3	enolase	XII	Pseudanabaena sp.	4.2.1.11
Q15QR6	enolase	XII	Pseudoalteromonas atlantica	4.2.1.11
Q3IDM2	enolase	XII	Pseudoalteromonas haloplanktis	4.2.1.11
A0A0F4Q2W7	enolase	XII	Pseudoalteromonas piscicida	4.2.1.11

Table S2.6. (continuation)

UniProt	Family [3]	Cluster	Species	EC Number
A4C6K0	enolase	XII	Pseudoalteromonas tunicata	4.2.1.11
G2IZ10	enolase	XII	Pseudogulbenkiania sp.	4.2.1.11
A0A094I6L2	enolase	XII	Pseudogymnoascus sp.	
A0A095VUJ7	enolase	XII	Pseudohaliea rubra	4.2.1.11
A6V1F3	enolase	XII	Pseudomonas aeruginosa	4.2.1.11
I3V1B9	enolase	XII	Pseudomonas putida	4.2.1.11
A0A081YD49	enolase	XII	Pseudomonas sp.	4.2.1.11
J2X2Y1	enolase	XII	Pseudomonas sp.	4.2.1.11
J2Y2D8	enolase	XII	Pseudomonas sp.	4.2.1.11
Q4ZWE0	enolase	XII	Pseudomonas syringae	4.2.1.11
A0A0A1YH48	enolase	XII	Pseudomonas taeanensis	4.2.1.11
F4CTX5	enolase	XII	Pseudonocardia dioxanivorans	4.2.1.11
A0A0A0EAA0	enolase	XII	Pseudooceanicola atlanticus	4.2.1.11
F0SEU5	enolase	XII	Pseudopedobacter saltans	4.2.1.11
E6MHG6	enolase	XII	Pseudoramibacter alactolyticus	4.2.1.11
A8F5E9	enolase	XII	Pseudothermotoga lettingae	4.2.1.11
G8PH24	enolase	XII	Pseudovibrio sp.	4.2.1.11
G7UP87	enolase	XII	Pseudoxanthomonas spadix	4.2.1.11
A5WG13	enolase	XII	Psychrobacter sp.	4.2.1.11
N1WT30	enolase	XII	Psychroflexus gondwanensis	4.2.1.11
D5BPG4	enolase	XII	Puniceispirillum marinum	4.2.1.11
D1Y6M3	enolase	XII	Pyramidobacter piscolens	4.2.1.11
A0A0B6WXK7	enolase	XII	Pyrinomonas methylaliphatogenes	4.2.1.11
Q8ZYE7	enolase	XII	Pyrobaculum aerophilum	4.2.1.11
G7VBQ8	enolase	XII	Pyrobaculum ferrireducens	4.2.1.11
Q9UXZ0	enolase	XII	Pyrococcus abyssi	4.2.1.11
F8AFK3	enolase	XII	Pyrococcus yayanosii	4.2.1.11
G0EDB5	enolase	XII	Pyrolobus fumarii	4.2.1.11
B2U9C3	enolase	XII	Ralstonia pickettii	4.2.1.11
U3QRY0	enolase	XII	Ralstonia pickettii	4.2.1.11
A0A0C5BGG6	enolase	XII	Rathayibacter toxicus	4.2.1.11
A4BCH7	enolase	XII	Reinekea blandensis	4.2.1.11
A0A0D2HDC8	enolase	XII	Rhinocladiella mackenziei	
A0A060HWW6	enolase	XII	Rhizobium etli	4.2.1.11
Q2K8W9	enolase	XII	Rhizobium etli	4.2.1.11
J3BM75	enolase	XII	Rhizobium sp.	4.2.1.11
X8J7Y6	enolase	XII	Rhizoctonia solani	
A0A015JTL5	enolase	XII	Rhizophagus irregularis	
I1BX31	enolase	XII	Rhizopus delemar	
I1CFP8	enolase	XII	Rhizopus delemar	
I4VJZ5	enolase	XII	Rhodanobacter fulvus	4.2.1.11
V7EHR6	enolase	XII	Rhodobacter sp.	4.2.1.11
A3PIV3	enolase	XII	Rhodobacter sphaeroides	4.2.1.11
B6AXV1	enolase	XII	Rhodobacteraceae bacterium	4.2.1.11
U4V089	enolase	XII	Rhodobacteraceae bacterium	4.2.1.11
X6L1V8	enolase	XII	Rhodobacteraceae bacterium	4.2.1.11
Q0FF02	enolase	XII	Rhodobacterales bacterium	4.2.1.11
C1A306	enolase	XII	Rhodococcus erythropolis	4.2.1.11
A0A069J7Z7	enolase	XII	Rhodococcus qingshengii	4.2.1.11
R7WIF3	enolase	XII	Rhodococcus rhodnii	4.2.1.11
R7WMM0	enolase	XII	Rhodococcus rhodnii	4.2.1.11
L8DKU4	enolase	XII	Rhodococcus sp.	4.2.1.11
M2W7C9	enolase	XII	Rhodococcus triatomae	4.2.1.11
L2TL30	enolase	XII	Rhodococcus wratislaviensis	4.2.1.11
A0A060JGY6	enolase	XII	Rhodoluna lacicola	4.2.1.11
A0A037V2F1	enolase	XII	Rhodomicrobium udaipurense	4.2.1.11

Table S2.6. (continuation)

UniProt	Family [3]	Cluster	Species	EC Number
M5RZ09	enolase	XII	Rhodopirellula maiorica	4.2.1.11
M5ULG4	enolase	XII	Rhodopirellula sallentina	4.2.1.11
M5T6P0	enolase	XII	Rhodopirellula sp.	4.2.1.11
M5TMM0	enolase	XII	Rhodopirellula sp.	4.2.1.11
Q136E5	enolase	XII	Rhodopseudomonas palustris	4.2.1.11
B6IQ30	enolase	XII	Rhodospirillum centenum	4.2.1.11
M7X749	enolase	XII	Rhodosporidium toruloides	
Q870B9	enolase	XII	Rhodotorula mucilaginosa	4.2.1.11
A0A095CUT1	enolase	XII	Rhodovulum sp.	4.2.1.11
J5PP59	enolase	XII	Rhodovulum sp.	4.2.1.11
A0A077FJ51	enolase	XII	Rickettsiales bacterium	4.2.1.11
A8PLE6	enolase	XII	Rickettsiella grylli	4.2.1.11
E4TBY8	enolase	XII	Riemerella anatipestifer	4.2.1.11
D4G821	enolase	XII	Riesia pediculicola	4.2.1.11
A4CJX1	enolase	XII	Robiginitalea biformata	4.2.1.11
G2SXV1	enolase	XII	Roseburia hominis	4.2.1.11
G2T3U0	enolase	XII	Roseburia hominis	4.2.1.11
C7GDS1	enolase	XII	Roseburia intestinalis	4.2.1.11
R6PRJ9	enolase	XII	Roseburia sp.	4.2.1.11
R7ECA7	enolase	XII	Roseburia sp.	4.2.1.11
R7INN0	enolase	XII	Roseburia sp.	4.2.1.11
W8RUJ6	enolase	XII	Roseibacterium elongatum	4.2.1.11
E2CRB5	enolase	XII	Roseibium sp.	4.2.1.11
A5V1W0	enolase	XII	Roseiflexus sp.	4.2.1.11
W4HQ93	enolase	XII	Roseivivax atlanticus	4.2.1.11
X7FAH6	enolase	XII	Roseivivax isoporae	4.2.1.11
A6FR37	enolase	XII	Roseobacter sp.	4.2.1.11
B7RQ35	enolase	XII	Roseobacter sp.	4.2.1.11
D5RIX8	enolase	XII	Roseomonas cervicalis	4.2.1.11
A0A0A0HGU3	enolase	XII	Roseovarius mucosus	4.2.1.11
D8USJ0	enolase	XII	Rothia dentocariosa	4.2.1.11
A0A075ATR4	enolase	XII	Rozella allomycis	4.2.1.11
A0A017HQZ8	enolase	XII	Rubellimicrobium mesophilum	4.2.1.11
S9SJB2	enolase	XII	Rubellimicrobium thermophilum	4.2.1.11
U5DN29	enolase	XII	Rubidibacter lacunae	4.2.1.11
I0HPA3	enolase	XII	Rubrivivax gelatinosus	4.2.1.11
A0A023X0W3	enolase	XII	Rubrobacter radiotolerans	4.2.1.11
Q1AXJ9	enolase	XII	Rubrobacter xylanophilus	4.2.1.11
Q5LQL4	enolase	XII	Ruegeria pomeroyi	4.2.1.11
F4XCZ6	enolase	XII	Ruminococcaceae bacterium	4.2.1.11
U2M0H4	enolase	XII	Ruminococcus callidus	4.2.1.11
D4LDC4	enolase	XII	Ruminococcus champanellensis	4.2.1.11
R5TXT4	enolase	XII	Ruminococcus gnavus	4.2.1.11
V8BQX0	enolase	XII	Ruminococcus gnavus	4.2.1.11
R7DBG2	enolase	XII	Ruminococcus obeum	4.2.1.11
R5E4I1	enolase	XII	Ruminococcus sp.	4.2.1.11
R5NQ97	enolase	XII	Ruminococcus sp.	4.2.1.11
R7GSQ8	enolase	XII	Ruminococcus sp.	4.2.1.11
R7H1E8	enolase	XII	Ruminococcus sp.	4.2.1.11
R7KM64	enolase	XII	Ruminococcus sp.	4.2.1.11
R7MSK6	enolase	XII	Ruminococcus sp.	4.2.1.11
F8EKQ7	enolase	XII	Runella slithyformis	4.2.1.11
A0A060Q9D6	enolase	XII	Saccharibacter sp.	4.2.1.11
I1D680	enolase	XII	Saccharomonospora glauca	4.2.1.11
P0CX10	enolase	XII	Saccharomyces cerevisiae	4.2.1.11
A3JYS8	enolase	XII	Sagittula stellata	4.2.1.11

Table S2.6. (continuation)

UniProt	Family [3]	Cluster	Species	EC Number
R4VUT1	enolase	XII	Salinarchaeum sp.	4.2.1.11
Q2S4F8	enolase	XII	Salinibacter ruber	4.2.1.11
A0A0C2E8G8	enolase	XII	Salinicoccus roseus	4.2.1.11
A0A084IJZ7	enolase	XII	Salinisphaera hydrothermalis	4.2.1.11
U2G0Z6	enolase	XII	Salinisphaera shabanensis	4.2.1.11
V5WCT9	enolase	XII	Salinispira pacifica	4.2.1.11
S9RWN8	enolase	XII	Salipiger mucosus	4.2.1.11
B5DGQ7	enolase	XII	Salmo salar	4.2.1.11
V2J5A2	enolase	XII	Salmonella enterica	4.2.1.11
D1BBH2	enolase	XII	Sanguibacter keddieii	4.2.1.11
A0A0C3N9U2	enolase	XII	Sanguibacteroides justesenii	4.2.1.11
T0QUI1	enolase	XII	Saprolegnia diclina	
A0A067D0S2	enolase	XII	Saprolegnia parasitica	
H6L0D9	enolase	XII	Saprospira grandis	4.2.1.11
G5ZVY1	enolase	XII	SAR116 cluster	4.2.1.11
G6A0D0	enolase	XII	SAR116 cluster	4.2.1.11
J4UZ59	enolase	XII	SAR86 cluster	4.2.1.11
J5KFF8	enolase	XII	SAR86 cluster	4.2.1.11
K6G5P2	enolase	XII	SAR86 cluster	4.2.1.11
A0A084G9L1	enolase	XII	Scedosporium apiospermum	
A0A084GAU3	enolase	XII	Scedosporium apiospermum	
P33676	enolase	XII	Schistosoma japonicum	4.2.1.11
S9VTN8	enolase	XII	Schizosaccharomyces cryophilus	
B6JZR2	enolase	XII	Schizosaccharomyces japonicus	
S9Q6W7	enolase	XII	Schizosaccharomyces octosporus	
P40370	enolase	XII	Schizosaccharomyces pombe	4.2.1.11
Q8NKC2	enolase	XII	Schizosaccharomyces pombe	4.2.1.11
A0A085L2Y7	enolase	XII	Schleiferia thermophila	4.2.1.11
A0A0C3AYF0	enolase	XII	Scleroderma citrinum	
A0A0C1WSV2	enolase	XII	Scytonema millei	4.2.1.11
A0A0C1X897	enolase	XII	Scytonema millei	4.2.1.11
J3TF02	enolase	XII	secondary endosymbiont	4.2.1.11
J3Z5S5	enolase	XII	secondary endosymbiont	4.2.1.11
D6Z7N6	enolase	XII	Segniliparus rotundus	4.2.1.11
D8RNF8	enolase	XII	Selaginella moellendorffii	
D8SMH9	enolase	XII	Selaginella moellendorffii	
I0GVM4	enolase	XII	Selenomonas ruminantium	4.2.1.11
J4WL21	enolase	XII	Selenomonas sp.	4.2.1.11
J4XG03	enolase	XII	Selenomonas sp.	4.2.1.11
A0A0C3B9L0	enolase	XII	Serendipita vermifera	
U2M450	enolase	XII	Serratia fonticola	4.2.1.11
A0A0A5LUA8	enolase	XII	Serratia marcescens	4.2.1.11
V3T1C3	enolase	XII	Serratia sp.	4.2.1.11
R4I0K6	enolase	XII	Serratia symbiotica	4.2.1.11
A1S4D7	enolase	XII	Shewanella amazonensis	4.2.1.11
Q086B0	enolase	XII	Shewanella frigidimarina	4.2.1.11
X8GSE3	enolase	XII	Shuttleworthia sp.	4.2.1.11
K4KJ44	enolase	XII	Simiduia agarivorans	4.2.1.11
K4KQH1	enolase	XII	Simiduia agarivorans	4.2.1.11
F8L481	enolase	XII	Simkania negevensis	4.2.1.11
L0DPP1	enolase	XII	Singulisphaera acidiphila	4.2.1.11
L0DR95	enolase	XII	Singulisphaera acidiphila	4.2.1.11
R4ILK5	enolase	XII	Sinorhizobium sp.	4.2.1.11
A0A0D2VWZ4	enolase	XII	Skermanella aerolata	4.2.1.11
D0WJ88	enolase	XII	Slackia exigua	4.2.1.11
C7N7U6	enolase	XII	Slackia heliotrinireducens	4.2.1.11

Table S2.6. (continuation)

UniProt	Family [3]	Cluster	Species	EC Number
K0YMK6	enolase	XII	Slackia piriformis	4.2.1.11
A0A091FA27	enolase	XII	Smithella sp.	4.2.1.11
A0A0E3ZBR0	enolase	XII	Sneathia amnii	4.2.1.11
A0A080JZE9	enolase	XII	Snodgrassella alvi	4.2.1.11
W0HZT9	enolase	XII	Sodalis praecaptivus	4.2.1.11
P26300	enolase	XII	Solanum lycopersicum	4.2.1.11
A0A0B0H746	enolase	XII	Solemya velum	4.2.1.11
H8KSX9	enolase	XII	Solitalea canadensis	4.2.1.11
G3ATE4	enolase	XII	Spathaspora passalidarum	
D1C4D4	enolase	XII	Sphaerobacter thermophilus	4.2.1.11
F4GL60	enolase	XII	Sphaerochaeta coccoides	4.2.1.11
G8QU67	enolase	XII	Sphaerochaeta pleomorpha	4.2.1.11
U2JAT9	enolase	XII	Sphingobacterium paucimobilis	4.2.1.11
F4C365	enolase	XII	Sphingobacterium sp.	4.2.1.11
G2IIF4	enolase	XII	Sphingobium sp.	4.2.1.11
A0A0E9MM98	enolase	XII	Sphingomonas changbaiensis	4.2.1.11
A0A0C5LEG8	enolase	XII	Sphingomonas hengshuiensis	4.2.1.11
A0A031JE98	enolase	XII	Sphingomonas paucimobilis	4.2.1.11
A0A031JH44	enolase	XII	Sphingomonas paucimobilis	4.2.1.11
W0ACE7	enolase	XII	Sphingomonas sanxanigenens	4.2.1.11
A0A0A6CTV2	enolase	XII	Sphingomonas sp.	4.2.1.11
J8SMB5	enolase	XII	Sphingomonas sp.	4.2.1.11
M4S3H9	enolase	XII	Sphingomonas sp.	4.2.1.11
A0A097EEU7	enolase	XII	Sphingomonas taxi	4.2.1.11
A0A0D1MGT0	enolase	XII	Sphingomonas taxi	4.2.1.11
A5V3E8	enolase	XII	Sphingomonas wittichii	4.2.1.11
N9UPW1	enolase	XII	Sphingopyxis sp.	4.2.1.11
U5T337	enolase	XII	Spiribacter sp.	4.2.1.11
H9UGX7	enolase	XII	Spirochaeta africana	4.2.1.11
E1R7X8	enolase	XII	Spirochaeta smaragdinae	4.2.1.11
A0A098QUU3	enolase	XII	Spirochaeta sp.	4.2.1.11
A0A0A0DM42	enolase	XII	Spirochaeta sp.	4.2.1.11
G0GEX1	enolase	XII	Spirochaeta thermophila	4.2.1.11
V6LEX0	enolase	XII	Spironucleus salmonicida	
S5G1I9	enolase	XII	Spodoptera litura	
A0A098LCE6	enolase	XII	Sporocytophaga myxococcoides	4.2.1.11
V6IWE5	enolase	XII	Sporolactobacillus laevolacticus	4.2.1.11
F9DXN4	enolase	XII	Sporosarcina newyorkensis	4.2.1.11
D3PZ17	enolase	XII	Stackebrandtia nassauensis	4.2.1.11
K9XRB3	enolase	XII	Stanieria cyanosphaera	4.2.1.11
A7WZT2	enolase	XII	Staphylococcus aureus	4.2.1.11
A0A0A8HRJ3	enolase	XII	Staphylococcus hyicus	4.2.1.11
K9B0Z4	enolase	XII	Staphylococcus massiliensis	4.2.1.11
R6TTE3	enolase	XII	Staphylococcus sp.	4.2.1.11
D6ZYJ2	enolase	XII	Starkeya novella	4.2.1.11
A0A087SYD4	enolase	XII	Stegodyphus mimosarum	
D7RIF2	enolase	XII	Steinernema carpocapsae	
A3KBH1	enolase	XII	Stephanonympha sp.	
W1VVN9	enolase	XII	Streptococcus parasanguinis	4.2.1.11
A0A0C2JG30	enolase	XII	Streptomonospora alba	4.2.1.11
S4MHW8	enolase	XII	Streptomyces afghaniensis	4.2.1.11
X0MSW1	enolase	XII	Streptomyces albulus	4.2.1.11
Q9F2Q3	enolase	XII	Streptomyces coelicolor	4.2.1.11
Q9F3P9	enolase	XII	Streptomyces coelicolor	4.2.1.11
N0CVQ0	enolase	XII	Streptomyces fulvissimus	4.2.1.11
L1KLF8	enolase	XII	Streptomyces ipomoeae	4.2.1.11

Table S2.6. (continuation)

UniProt	Family [3]	Cluster	Species	EC Number
V6KHM4	enolase	XII	Streptomyces niveus	4.2.1.11
A0A0B5DFA9	enolase	XII	Streptomyces nodosus	4.2.1.11
A0A066YDE2	enolase	XII	Streptomyces olindensis	4.2.1.11
A0A0C1TIN6	enolase	XII	Streptomyces pluripotens	4.2.1.11
A0A022MJU4	enolase	XII	Streptomyces sp.	4.2.1.11
A0A069K0Y6	enolase	XII	Streptomyces sp.	4.2.1.11
A0A087K0P9	enolase	XII	Streptomyces sp.	4.2.1.11
A0A0C2B5G7	enolase	XII	Streptomyces sp.	4.2.1.11
A0A0F5A8E7	enolase	XII	Streptomyces sp.	4.2.1.11
A0A0F5ADJ4	enolase	XII	Streptomyces sp.	4.2.1.11
D9V441	enolase	XII	Streptomyces sp.	4.2.1.11
F3ZAK7	enolase	XII	Streptomyces sp.	4.2.1.11
K1W4P5	enolase	XII	Streptomyces sp.	4.2.1.11
S2YXS5	enolase	XII	Streptomyces sp.	4.2.1.11
V9Z6M5	enolase	XII	Streptomyces sp.	4.2.1.11
L7FDR5	enolase	XII	Streptomyces turgidiscabies	4.2.1.11
F2RC75	enolase	XII	Streptomyces venezuelae	4.2.1.11
A0A090MVT2	enolase	XII	Strongyloides ratti	
G9RYE5	enolase	XII	Subdoligranulum sp.	4.2.1.11
E8LMX6	enolase	XII	Succinatimonas hippei	4.2.1.11
R5ELH7	enolase	XII	Succinatimonas sp.	4.2.1.11
A0A073IIJ5	enolase	XII	Sulfitobacter donghicola	4.2.1.11
A0A061SWC0	enolase	XII	Sulfitobacter mediterraneus	4.2.1.11
G8U0N0	enolase	XII	Sulfobacillus acidophilus	4.2.1.11
W7KLB8	enolase	XII	Sulfolobales archaeon	4.2.1.11
C3MPU0	enolase	XII	Sulfolobus islandicus	4.2.1.11
A0A0E9MD30	enolase	XII	Sulfuricella sp.	4.2.1.11
E4TW98	enolase	XII	Sulfuricurvum kujiense	4.2.1.11
A0A0C2ZTL1	enolase	XII	Sulfuricurvum sp.	4.2.1.11
C1DWH5	enolase	XII	Sulfurihydrogenibium azorense	4.2.1.11
E0USY0	enolase	XII	Sulfurimonas autotrophica	4.2.1.11
Q30P06	enolase	XII	Sulfurimonas denitrificans	4.2.1.11
W0SGS2	enolase	XII	Sulfuritalea hydrogenivorans	4.2.1.11
D1B577	enolase	XII	Sulfurospirillum deleyianum	4.2.1.11
A0A0B0Q2R1	enolase	XII	Sulfurospirillum sp.	4.2.1.11
A0A0C2YY49	enolase	XII	Sulfurovum sp.	4.2.1.11
A0A0C2ZKA7	enolase	XII	Sulfurovum sp.	4.2.1.11
A0A0C2ZL52	enolase	XII	Sulfurovum sp.	4.2.1.11
A6Q9K9	enolase	XII	Sulfurovum sp.	4.2.1.11
I2K4F8	enolase	XII	Sulfurovum sp.	4.2.1.11
H3KF04	enolase	XII	Sutterella parvirubra	4.2.1.11
R7C5H4	enolase	XII	Sutterella sp.	4.2.1.11
R7I4U7	enolase	XII	Sutterella sp.	4.2.1.11
R7KG50	enolase	XII	Sutterella sp.	4.2.1.11
K1JT11	enolase	XII	Sutterella wadsworthensis	4.2.1.11
S3BBF6	enolase	XII	Sutterella wadsworthensis	4.2.1.11
Q67SV9	enolase	XII	Symbiobacterium thermophilum	4.2.1.11
Q31QJ8	enolase	XII	Synechococcus elongatus	4.2.1.11
A3YU92	enolase	XII	Synechococcus sp.	4.2.1.11
A5GPE0	enolase	XII	Synechococcus sp.	4.2.1.11
A5GWK7	enolase	XII	Synechococcus sp.	4.2.1.11
B4WR07	enolase	XII	Synechococcus sp.	4.2.1.11
K9RVM4	enolase	XII	Synechococcus sp.	4.2.1.11
K9SU56	enolase	XII	Synechococcus sp.	4.2.1.11
Q2JIT3	enolase	XII	Synechococcus sp.	4.2.1.11
G9PYB7	enolase	XII	Synergistes sp.	4.2.1.11

Table S2.6. (continuation)

UniProt	Family [3]	Cluster	Species	EC Number
A0LEC9	enolase	XII	Syntrophobacter fumaroxidans	4.2.1.11
F0T1Q1	enolase	XII	Syntrophobotulus glycolicus	4.2.1.11
Q0B080	enolase	XII	Syntrophomonas wolfei	4.2.1.11
D7CPG7	enolase	XII	Syntrophothermus lipocalidus	4.2.1.11
Q2LR33	enolase	XII	Syntrophus aciditrophicus	4.2.1.11
I2B2U1	enolase	XII	Taenia multiceps	
G9S5N8	enolase	XII	Tannerella sp.	4.2.1.11
R5IDI6	enolase	XII	Tannerella sp.	4.2.1.11
W2CB38	enolase	XII	Tannerella sp.	4.2.1.11
A0A098GHQ8	enolase	XII	Tatlockia micdadei	4.2.1.11
A0A099NP12	enolase	XII	Taylorella equigenitalis	4.2.1.11
F4LQU7	enolase	XII	Tepidanaerobacter acetatoxydans	4.2.1.11
A0A081B8Y1	enolase	XII	Tepidicaulis marinus	4.2.1.11
A0A075LP56	enolase	XII	Terribacillus aidingensis	4.2.1.11
G4L975	enolase	XII	Tetragenococcus halophilus	4.2.1.11
I2H4A9	enolase	XII	Tetrapisispora blattae	
N0E5K3	enolase	XII	Tetrasphaera elongata	4.2.1.11
B8BZT5	enolase	XII	Thalassiosira pseudonana	4.2.1.11
B8C355	enolase	XII	Thalassiosira pseudonana	4.2.1.11
A0A099TFH3	enolase	XII	Thalassobacter sp.	4.2.1.11
W8FV27	enolase	XII	Thalassolituus oleivorans	4.2.1.11
A0A0B4Y0H0	enolase	XII	Thalassospira xiamenensis	4.2.1.11
A0A099L6F2	enolase	XII	Thalassotalea sp.	4.2.1.11
A0A099LF63	enolase	XII	Thalassotalea sp.	4.2.1.11
N6YGF0	enolase	XII	Thauera sp.	4.2.1.11
J4DAA3	enolase	XII	Theileria orientalis	
A0A0C2IVW0	enolase	XII	Thelohanellus kitauei	
A0A061G0N4	enolase	XII	Theobroma cacao	
A0A061G7D5	enolase	XII	Theobroma cacao	
K4LFQ3	enolase	XII	Thermacetogenium phaeum	4.2.1.11
E6SKU4	enolase	XII	Thermaerobacter marianensis	4.2.1.11
H0UPF8	enolase	XII	Thermanaerovibrio velox	4.2.1.11
D5XC90	enolase	XII	Thermincola potens	4.2.1.11
A0A094JQ51	enolase	XII	Thermoactinomyces sp.	4.2.1.11
B0K6X6	enolase	XII	Thermoanaerobacter sp.	4.2.1.11
F6BHX9	enolase	XII	Thermoanaerobacterium	4.2.1.11
A0A062XTD3	enolase	XII	Thermoanaerobaculum aquaticum	4.2.1.11
Q47SV1	enolase	XII	Thermobifida fusca	4.2.1.11
D6Y890	enolase	XII	Thermobispora bispora	4.2.1.11
R7RR33	enolase	XII	Thermobrachium celere	4.2.1.11
W0DG57	enolase	XII	Thermocrinis ruber	4.2.1.11
A0A075WVB6	enolase	XII	Thermodesulfobacterium commune	4.2.1.11
M1E586	enolase	XII	Thermodesulfobium narugense	4.2.1.11
A1RWE2	enolase	XII	Thermofilum pendens	4.2.1.11
S6A544	enolase	XII	Thermofilum sp.	4.2.1.11
I3TCH5	enolase	XII	Thermogladius cellulolyticus	4.2.1.11
B9KYT8	enolase	XII	Thermomicrobium roseum	4.2.1.11
D1A7N3	enolase	XII	Thermomonospora curvata	4.2.1.11
Q9HJT1	enolase	XII	Thermoplasma acidophilum	4.2.1.11
Q979Z9	enolase	XII	Thermoplasma volcanium	4.2.1.11
M4YP09	enolase	XII	Thermoplasmatales archaeon	4.2.1.11
M7T9L7	enolase	XII	Thermoplasmatales archaeon	4.2.1.11
M7TBS3	enolase	XII	Thermoplasmatales archaeon	4.2.1.11
T0LZW7	enolase	XII	Thermoplasmatales archaeon	4.2.1.11
T0MSR9	enolase	XII	Thermoplasmatales archaeon	4.2.1.11
T0MVB2	enolase	XII	Thermoplasmatales archaeon	4.2.1.11

Table S2.6. (continuation)

UniProt	Family [3]	Cluster	Species	EC Number
T0NC23	enolase	XII	Thermoplasmatales archaeon	4.2.1.11
F2L2M4	enolase	XII	Thermoproteus uzoniensis	4.2.1.11
A1HSQ9	enolase	XII	Thermosinus carboxydivorans	4.2.1.11
B7IFN4	enolase	XII	Thermosipho africanus	4.2.1.11
A6LJF0	enolase	XII	Thermosipho melanesiensis	4.2.1.11
D5U004	enolase	XII	Thermosphaera aggregans	4.2.1.11
P42848	enolase	XII	Thermotoga maritima	4.2.1.11
G7V8Q3	enolase	XII	Thermovirga lienii	4.2.1.11
A0A0A2WUJ2	enolase	XII	Thermus filiformis	4.2.1.11
K7QTC7	enolase	XII	Thermus oshimai	4.2.1.11
Q72H85	enolase	XII	Thermus thermophilus	4.2.1.11
A0A0F4ZLQ7	enolase	XII	Thielaviopsis punctulata	
W0DWR9	enolase	XII	Thioalkalimicrobium aerophilum	4.2.1.11
L0DVB7	enolase	XII	Thioalkalivibrio nitratireducens	4.2.1.11
D3SAB9	enolase	XII	Thioalkalivibrio sp.	4.2.1.11
B8GQ75	enolase	XII	Thioalkalivibrio sulfidiphilus	4.2.1.11
F9UBK8	enolase	XII	Thiocapsa marina	4.2.1.11
A0A085TV03	enolase	XII	Thioclava sp.	4.2.1.11
I3YFP2	enolase	XII	Thiocystis violascens	4.2.1.11
W0TQY1	enolase	XII	Thiolapillus brandeum	4.2.1.11
Q31G68	enolase	XII	Thiomicrospira crunogena	4.2.1.11
D6CRM6	enolase	XII	Thiomonas arsenitoxydans	4.2.1.11
D6CVF4	enolase	XII	Thiomonas arsenitoxydans	4.2.1.11
D5X1T4	enolase	XII	Thiomonas intermedia	4.2.1.11
A0A090ANZ4	enolase	XII	Thioploca ingrica	4.2.1.11
G2DXX5	enolase	XII	Thiorhodococcus drewsii	4.2.1.11
G4E483	enolase	XII	Thiorhodospira sibirica	4.2.1.11
H8Z3C8	enolase	XII	Thiorhodovibrio sp.	4.2.1.11
I3BZ66	enolase	XII	Thiothrix nivea	4.2.1.11
J0LEB9	enolase	XII	Thiovulum sp.	4.2.1.11
Q5WQL6	enolase	XII	Thraustotheca clavata	
A0A095XDD5	enolase	XII	Tissierellia bacterium	4.2.1.11
A0A095YTT2	enolase	XII	Tissierellia bacterium	4.2.1.11
A0A095ZRZ5	enolase	XII	Tissierellia bacterium	4.2.1.11
I3TLE7	enolase	XII	Tistrella mobilis	4.2.1.11
Q9UAE6	enolase	XII	Toxoplasma gondii	4.2.1.11
F5YCD8	enolase	XII	Treponema azotonutricium	4.2.1.11
F4LP46	enolase	XII	Treponema brennaborense	4.2.1.11
F8F056	enolase	XII	Treponema caldarium	4.2.1.11
Q73P50	enolase	XII	Treponema denticola	4.2.1.11
S3K493	enolase	XII	Treponema maltophilum	4.2.1.11
P74934	enolase	XII	Treponema pallidum	4.2.1.11
A0A0B7GTE7	enolase	XII	Treponema phagedenis	4.2.1.11
F5YJB7	enolase	XII	Treponema primitia	4.2.1.11
U2LJC5	enolase	XII	Treponema socranskii	4.2.1.11
A0A0A0X143	enolase	XII	Treponema sp.	4.2.1.11
I0XAN4	enolase	XII	Treponema sp.	4.2.1.11
F2NWL0	enolase	XII	Treponema succinifaciens	4.2.1.11
D6X017	enolase	XII	Tribolium castaneum	
Q110V4	enolase	XII	Trichodesmium erythraeum	4.2.1.11
B3RZY5	enolase	XII	Trichoplax adhaerens	
K1VJ96	enolase	XII	Trichosporon asahii	
A0A077YX57	enolase	XII	Trichuris trichiura	
M8ABT2	enolase	XII	Triticum urartu	
Q83H73	enolase	XII	Tropheryma whipplei	4.2.1.11
D7CWQ5	enolase	XII	Truepera radiovictrix	4.2.1.11

Table S2.6. (continuation)

UniProt	Family [3]	Cluster	Species	EC Number
A0A0A8FK94	enolase	XII	Trueperella pyogenes	4.2.1.11
D0A1Y2	enolase	XII	Trypanosoma brucei	
S5NFX0	enolase	XII	Trypanosoma cruzi	4.2.1.11
A0A061IS86	enolase	XII	Trypanosoma rangeli	
G0U5T7	enolase	XII	Trypanosoma vivax	4.2.1.11
D5UVH0	enolase	XII	Tsukamurella paurometabola	4.2.1.11
A0A0C3L2H7	enolase	XII	Tulasnella calospora	
A0A074LPM6	enolase	XII	Tumebacillus flagellatus	4.2.1.11
I7KIG0	enolase	XII	Turicella otitidis	4.2.1.11
H5SI69	enolase	XII	uncultured Acetothermia	4.2.1.11
V4JZD2	enolase	XII	uncultured Acidilobus	4.2.1.11
F2YWW0	enolase	XII	uncultured Acidobacteria	4.2.1.11
H5SFW5	enolase	XII	uncultured Acidobacteria	4.2.1.11
V4XPJ9	enolase	XII	uncultured archaeon	4.2.1.11
V4Y4R8	enolase	XII	uncultured archaeon	4.2.1.11
V5A5C4	enolase	XII	uncultured archaeon	4.2.1.11
K2BQ86	enolase	XII	uncultured bacterium	
K2CCT3	enolase	XII	uncultured bacterium	
K2FSM3	enolase	XII	uncultured bacterium	
A0A059WW83	enolase	XII	uncultured bacterium	4.2.1.11
K1XJE2	enolase	XII	uncultured bacterium	4.2.1.11
K1Z6G8	enolase	XII	uncultured bacterium	4.2.1.11
K1ZHR7	enolase	XII	uncultured bacterium	4.2.1.11
K1ZU32	enolase	XII	uncultured bacterium	4.2.1.11
K2A1D1	enolase	XII	uncultured bacterium	4.2.1.11
K2AB67	enolase	XII	uncultured bacterium	4.2.1.11
K2AHD7	enolase	XII	uncultured bacterium	4.2.1.11
K2ANN7	enolase	XII	uncultured bacterium	4.2.1.11
K2AQP7	enolase	XII	uncultured bacterium	4.2.1.11
K2B776	enolase	XII	uncultured bacterium	4.2.1.11
K2BG27	enolase	XII	uncultured bacterium	4.2.1.11
K2BQR0	enolase	XII	uncultured bacterium	4.2.1.11
K2BY99	enolase	XII	uncultured bacterium	4.2.1.11
K2D2D4	enolase	XII	uncultured bacterium	4.2.1.11
K2DMJ6	enolase	XII	uncultured bacterium	4.2.1.11
K2E245	enolase	XII	uncultured bacterium	4.2.1.11
K2E3J9	enolase	XII	uncultured bacterium	4.2.1.11
K2E752	enolase	XII	uncultured bacterium	4.2.1.11
K2EJF2	enolase	XII	uncultured bacterium	4.2.1.11
K2ELG6	enolase	XII	uncultured bacterium	4.2.1.11
K2ELX2	enolase	XII	uncultured bacterium	4.2.1.11
K2EM90	enolase	XII	uncultured bacterium	4.2.1.11
K2F2H8	enolase	XII	uncultured bacterium	4.2.1.11
K2FFY8	enolase	XII	uncultured bacterium	4.2.1.11
K2FP41	enolase	XII	uncultured bacterium	4.2.1.11
K2GNX9	enolase	XII	uncultured bacterium	4.2.1.11
K2GQ36	enolase	XII	uncultured bacterium	4.2.1.11
M1L0F5	enolase	XII	uncultured bacterium	4.2.1.11
G3BMR0	enolase	XII	uncultured candidate	4.2.1.11
E1YE22	enolase	XII	uncultured Desulfobacterium	4.2.1.11
V4J7G9	enolase	XII	uncultured Desulfofustis	4.2.1.11
H6RE44	enolase	XII	uncultured Flavobacteriia	4.2.1.11
A0A075FL09	enolase	XII	uncultured marine	4.2.1.11
A0A075GC87	enolase	XII	uncultured marine	4.2.1.11
A0A075GG62	enolase	XII	uncultured marine	4.2.1.11
A0A075GGT2	enolase	XII	uncultured marine	4.2.1.11

Table S2.6. (continuation)

UniProt	Family [3]	Cluster	Species	EC Number
A0A075GIU6	enolase	XII	uncultured marine	4.2.1.11
A0A075GPK2	enolase	XII	uncultured marine	4.2.1.11
Q6SGG1	enolase	XII	uncultured marine	4.2.1.11
E7C683	enolase	XII	uncultured nuHF2	4.2.1.11
B1GYL7	enolase	XII	Uncultured termite	4.2.1.11
V4LED9	enolase	XII	uncultured Thiohalocapsa	4.2.1.11
A0A0C5RNW5	enolase	XII	Ureaplasma diversum	4.2.1.11
B1AIH2	enolase	XII	Ureaplasma parvum	4.2.1.11
B5ZAZ0	enolase	XII	Ureaplasma urealyticum	4.2.1.11
V6Q788	enolase	XII	Vagococcus lutrae	4.2.1.11
A7TEY1	enolase	XII	Vanderwaltozyma polyspora	
W1TUA1	enolase	XII	Varibaculum cambriense	4.2.1.11
J3CQ44	enolase	XII	Variovorax sp.	4.2.1.11
A0A096C045	enolase	XII	Veillonella montpellierensis	4.2.1.11
E4LDD0	enolase	XII	Veillonella sp.	4.2.1.11
F9N718	enolase	XII	Veillonella sp.	4.2.1.11
R5BRJ8	enolase	XII	Veillonella sp.	4.2.1.11
A1WLU9	enolase	XII	Verminephrobacter eiseniae	4.2.1.11
A0A0E3YTQ9	enolase	XII	Verrucomicrobia bacterium	4.2.1.11
A0A0D2AVQ5	enolase	XII	Verruconis gallopava	
A5CX71	enolase	XII	Vesicomyosocius okutanii	4.2.1.11
A6ALC2	enolase	XII	Vibrio campbellii	4.2.1.11
U4EH51	enolase	XII	Vibrio nigripulchritudo	4.2.1.11
W4EN79	enolase	XII	Viridibacillus arenosi	4.2.1.11
E1QNF7	enolase	XII	Vulcanisaeta distributa	4.2.1.11
F8LAZ8	enolase	XII	Waddlia chondrophila	4.2.1.11
R9AID0	enolase	XII	Wallemia ichthyophaga	
F0NYT2	enolase	XII	Weeksella virosa	4.2.1.11
A0A075TXS5	enolase	XII	Weissella ceti	
A0A069CUH7	enolase	XII	Weissella oryzae	4.2.1.11
A0A0D0NKD8	enolase	XII	Wenxinia marina	4.2.1.11
E9M0G1	enolase	XII	Wickerhamomyces ciferrii	
H6Q4W3	enolase	XII	Wigglesworthia glossinidia	4.2.1.11
Q8D2K1	enolase	XII	Wigglesworthia glossinidia	4.2.1.11
V8CTE5	enolase	XII	Williamsia sp.	4.2.1.11
S7VS02	enolase	XII	Winogradskyella psychrotolerans	4.2.1.11
L8XUP5	enolase	XII	Wohlfahrtiimonas chitiniclastica	4.2.1.11
A0A060PXH5	enolase	XII	Wolbachia endosymbiont	4.2.1.11
I7JEI8	enolase	XII	Wolbachia endosymbiont	4.2.1.11
B3CNP3	enolase	XII	Wolbachia pipientis	4.2.1.11
C0R5N4	enolase	XII	Wolbachia sp.	4.2.1.11
Q5GTG4	enolase	XII	Wolbachia sp.	4.2.1.11
Q7M8Q0	enolase	XII	Wolinella succinogenes	4.2.1.11
A7INB6	enolase	XII	Xanthobacter autotrophicus	4.2.1.11
L8M6E4	enolase	XII	Xenococcus sp.	4.2.1.11
D1BXQ5	enolase	XII	Xylanimonas cellulosilytica	4.2.1.11
B2I937	enolase	XII	Xylella fastidiosa	4.2.1.11
Q6C1F3	enolase	XII	Yarrowia lipolytica	4.2.1.11
G9Z8B8	enolase	XII	Yokenella regensburgei	4.2.1.11
V7I5Y2	enolase	XII	Youngiibacter fragilis	4.2.1.11
P26301	enolase	XII	Zea mays	4.2.1.11
P42895	enolase	XII	Zea mays	4.2.1.11
Q6JKT3	enolase	XII	Zootermopsis parabasalian	
Q6JKT4	enolase	XII	Zootermopsis parabasalian	
Q6JKT5	enolase	XII	Zootermopsis parabasalian	
P33675	enolase	XII	Zymomonas mobilis	4.2.1.11

Table S2.6. (continuation)

UniProt	Family [3]	Cluster	Species	EC Number
A0A038GE84	glucarate dehydratase	VIII		
A0A0B5FBX3	glucarate dehydratase	VIII		
S6MRM5	glucarate dehydratase	VIII		
W8FCK7	glucarate dehydratase	VIII	Agrobacterium tumefaciens	
A0A066U9S9	glucarate dehydratase	VIII	Amycolatopsis rifamycinica	
R1I3Z1	glucarate dehydratase	VIII	Amycolatopsis vancoresmycina	
A0A086ZGW6	glucarate dehydratase	VIII	Bifidobacterium bohemicum	4.2.1.40
K5DVM6	glucarate dehydratase	VIII	Bradyrhizobium lupini	
A0A0F5JY99	glucarate dehydratase	VIII	Burkholderia andropogonis	
A0A095WC85	glucarate dehydratase	VIII	Burkholderia gladioli	
A0A0B6RY10	glucarate dehydratase	VIII	Burkholderia glumae	4.2.1.40
A0A069P6W0	glucarate dehydratase	VIII	Burkholderia grimmiae	
A0A038H5T4	glucarate dehydratase	VIII	Burkholderia jiangsuensis	
A0A095EXD5	glucarate dehydratase	VIII	Burkholderia mallei	
B2TB15	glucarate dehydratase	VIII	Burkholderia phytofirmans	
A0A060PB25	glucarate dehydratase	VIII	Burkholderia sp.	
A0A084DN05	glucarate dehydratase	VIII	Burkholderia sp.	
R4X037	glucarate dehydratase	VIII	Burkholderia sp.	
A0A0C2CF73	glucarate dehydratase	VIII	Burkholderia sp.	4.2.1.40
W8WUM2	glucarate dehydratase	VIII	Castellaniella defragrans	4.2.1.40
Q1QUM5	glucarate dehydratase	VIII	Chromohalobacter salexigens	
W9WX56	glucarate dehydratase	VIII	Cladophialophora psammophila	
A0A067LS14	glucarate dehydratase	VIII	Clavibacter cf.	
A0A010RRJ3	glucarate dehydratase	VIII	Colletotrichum fioriniae	
A0A014MC02	glucarate dehydratase	VIII	Comamonas aquatica	
A0A096F927	glucarate dehydratase	VIII	Comamonas testosteroni	
A0A0E3BX71	glucarate dehydratase	VIII	Comamonas testosteroni	
R7ZW00	glucarate dehydratase	VIII	Cyclobacteriaceae bacterium	
S7VDN0	glucarate dehydratase	VIII	Cyclobacterium qasimii	4.2.1.40
A0A0F5FR79	glucarate dehydratase	VIII	Devosia geojensis	
I2EUQ2	glucarate dehydratase	VIII	Emticicia oligotrophica	
H4V537	glucarate dehydratase	VIII	Escherichia coli	
S1IKY2	glucarate dehydratase	VIII	Escherichia coli	
A0A070T4Y9	glucarate dehydratase	VIII	Escherichia coli	4.2.1.40
M7SYJ6	glucarate dehydratase	VIII	Eutypa lata	
W9ZLB5	glucarate dehydratase	VIII	Fusarium oxysporum	
X0BTH7	glucarate dehydratase	VIII	Fusarium oxysporum	
K3VRU5	glucarate dehydratase	VIII	Fusarium pseudograminearum	
W7ME34	glucarate dehydratase	VIII	Gibberella moniliformis	
A0A016Q4W2	glucarate dehydratase	VIII	Gibberella zeae	
D0LDF9	glucarate dehydratase	VIII	Gordonia bronchialis	4.2.1.40
K6WJ67	glucarate dehydratase	VIII	Gordonia rhizosphera	
J9SIC3	glucarate dehydratase	VIII	Gordonia sp.	
R7YAA3	glucarate dehydratase	VIII	Gordonia terrae	
A0A0C5VI02	glucarate dehydratase	VIII	Gynuella sunshinyii	4.2.1.40
I3CY13	glucarate dehydratase	VIII	Herbaspirillum sp.	
J3D023	glucarate dehydratase	VIII	Herbaspirillum sp.	
A0A016XLM2	glucarate dehydratase	VIII	Hylemonella gracilis	
W5WGB0	glucarate dehydratase	VIII	Kutzneria albida	
W7T1B9	glucarate dehydratase	VIII	Kutzneria sp.	
A0A081G4H7	glucarate dehydratase	VIII	Marinobacterium sp.	
A0A0D0JZQ6	glucarate dehydratase	VIII	Microbacterium sp.	
W2EVN8	glucarate dehydratase	VIII	Microbispora sp.	
W9APD4	glucarate dehydratase	VIII	Mycobacterium cosmeticum	
A0A024M426	glucarate dehydratase	VIII	Mycobacterium farcinogenes	
X5LDJ9	glucarate dehydratase	VIII	Mycobacterium mageritense	

Table S2.6. (continuation)

UniProt	Family [3]	Cluster	Species	EC Number
V5XI18	glucarate dehydratase	VIII	Mycobacterium neoaurum	
A0A099CE11	glucarate dehydratase	VIII	Mycobacterium rufum	
A0A0B2Y1D3	glucarate dehydratase	VIII	Mycobacterium setense	
A0A0B2Y884	glucarate dehydratase	VIII	Mycobacterium setense	
L8F4C2	glucarate dehydratase	VIII	Mycobacterium smegmatis	
X5LT29	glucarate dehydratase	VIII	Mycobacterium vulneris	
A0A034ULJ2	glucarate dehydratase	VIII	Nocardia brasiliensis	
A0A0C1DBX6	glucarate dehydratase	VIII	Nocardia vulneris	
A0A097N7F3	glucarate dehydratase	VIII	Pandoraea pnomenusa	
A0A0B3TCP1	glucarate dehydratase	VIII	Pandoraea pnomenusa	
A0A0A8BST8	glucarate dehydratase	VIII	Pandoraea pulmonicola	
R7WVG9	glucarate dehydratase	VIII	Pandoraea sp.	
V5UDH5	glucarate dehydratase	VIII	Pandoraea sp.	
A0A0A8BLJ9	glucarate dehydratase	VIII	Pandoraea sputorum	
A0A0C5JTW1	glucarate dehydratase	VIII	Pandoraea vervacti	
W3XJL6	glucarate dehydratase	VIII	Pestalotiopsis fici	
J3DDF1	glucarate dehydratase	VIII	Polaromonas sp.	
A0A0B3S866	glucarate dehydratase	VIII	Ponticoccus sp.	4.2.1.40
A0A0C1NPC3	glucarate dehydratase	VIII	Prauserella sp.	4.2.1.40
K2RX67	glucarate dehydratase	VIII	Pseudomonas avellanae	
W8PJ38	glucarate dehydratase	VIII	Pseudomonas brassicacearum	
A0A0A6D7S7	glucarate dehydratase	VIII	Pseudomonas chlororaphis	
W0HGW4	glucarate dehydratase	VIII	Pseudomonas cichorii	
A0A0C1W273	glucarate dehydratase	VIII	Pseudomonas fluorescens	
A0A0C1WH07	glucarate dehydratase	VIII	Pseudomonas fluorescens	
A0A0D0KJL3	glucarate dehydratase	VIII	Pseudomonas fluorescens	
A0A0D0NDL4	glucarate dehydratase	VIII	Pseudomonas fluorescens	
A0A0F4VHX1	glucarate dehydratase	VIII	Pseudomonas fluorescens	
J2ENS8	glucarate dehydratase	VIII	Pseudomonas fluorescens	
L7HMU5	glucarate dehydratase	VIII	Pseudomonas fluorescens	
A0A0D0T5Y3	glucarate dehydratase	VIII	Pseudomonas fluorescens	4.2.1.40
A0A0E3KHT0	glucarate dehydratase	VIII	Pseudomonas fluorescens	4.2.1.40
A0A0B1ZAM2	glucarate dehydratase	VIII	Pseudomonas frederiksbergensis	
A0A0D0JEH2	glucarate dehydratase	VIII	Pseudomonas fulva	
A0A0F4XEE3	glucarate dehydratase	VIII	Pseudomonas kilonensis	
A0A098SYH1	glucarate dehydratase	VIII	Pseudomonas lutea	
A0A059KXC6	glucarate dehydratase	VIII	Pseudomonas mandelii	
A0A0A4GE09	glucarate dehydratase	VIII	Pseudomonas mediterranea	
V8R6W6	glucarate dehydratase	VIII	Pseudomonas moraviensis	
M4JZQ7	glucarate dehydratase	VIII	Pseudomonas poae	
S6BBC4	glucarate dehydratase	VIII	Pseudomonas resinovorans	4.2.1.40
A0A089YP19	glucarate dehydratase	VIII	Pseudomonas rhizosphaerae	
A0A031J1V0	glucarate dehydratase	VIII	Pseudomonas sp.	
A0A077LER5	glucarate dehydratase	VIII	Pseudomonas sp.	
A0A0E9ZU31	glucarate dehydratase	VIII	Pseudomonas sp.	
I4N3V6	glucarate dehydratase	VIII	Pseudomonas sp.	
J2MP87	glucarate dehydratase	VIII	Pseudomonas sp.	
J2PMB2	glucarate dehydratase	VIII	Pseudomonas sp.	
J2TR08	glucarate dehydratase	VIII	Pseudomonas sp.	
J2XDY8	glucarate dehydratase	VIII	Pseudomonas sp.	
J3E662	glucarate dehydratase	VIII	Pseudomonas sp.	
J3GEJ2	glucarate dehydratase	VIII	Pseudomonas sp.	
J3GQP2	glucarate dehydratase	VIII	Pseudomonas sp.	
M5QRS1	glucarate dehydratase	VIII	Pseudomonas sp.	
N2J4B0	glucarate dehydratase	VIII	Pseudomonas sp.	
S6H523	glucarate dehydratase	VIII	Pseudomonas sp.	

Table S2.6. (continuation)

UniProt	Family [3]	Cluster	Species	EC Number
S6HH47	glucarate dehydratase	VIII	Pseudomonas sp.	
S6JPK8	glucarate dehydratase	VIII	Pseudomonas sp.	
A0A0A1HUE2	glucarate dehydratase	VIII	Pseudomonas sp.	4.2.1.40
W6VAQ0	glucarate dehydratase	VIII	Pseudomonas sp.	4.2.1.40
A0A0E2J832	glucarate dehydratase	VIII	Pseudomonas syringae	
S3MYD0	glucarate dehydratase	VIII	Pseudomonas syringae	
A0A0D0LE05	glucarate dehydratase	VIII	Pseudomonas viridiflava	
F4GNS5	glucarate dehydratase	VIII	Pusillimonas sp.	
S9S3I1	glucarate dehydratase	VIII	Ralstonia sp.	
F5XX60	glucarate dehydratase	VIII	Ramlibacter tataouinensis	
A0A068T3L5	glucarate dehydratase	VIII	Rhizobium galegae	
A0A024IXQ6	glucarate dehydratase	VIII	Rhizobium radiobacter	
A0A037XTQ3	glucarate dehydratase	VIII	Rhizobium radiobacter	
A0A098RNB3	glucarate dehydratase	VIII	Rhizobium sp.	
U4Q057	glucarate dehydratase	VIII	Rhizobium sp.	4.2.1.40
W6WQP2	glucarate dehydratase	VIII	Rhizobium sp.	4.2.1.40
A0A076EQ94	glucarate dehydratase	VIII	Rhodococcus opacus	
K8XWR0	glucarate dehydratase	VIII	Rhodococcus opacus	
A0A0D0JTH4	glucarate dehydratase	VIII	Rhodococcus sp.	
J2JBT3	glucarate dehydratase	VIII	Rhodococcus sp.	
A0A0F3I9U1	glucarate dehydratase	VIII	Rhodococcus sp.	4.2.1.40
M2V9X8	glucarate dehydratase	VIII	Rhodococcus triatomae	
L2TKS1	glucarate dehydratase	VIII	Rhodococcus wratislaviensis	
X0Q9E7	glucarate dehydratase	VIII	Rhodococcus wratislaviensis	
G5PS54	glucarate dehydratase	VIII	Salmonella enterica	
G5SG79	glucarate dehydratase	VIII	Salmonella enterica	
S4MJ65	glucarate dehydratase	VIII	Streptomyces afghaniensis	
S4MWR8	glucarate dehydratase	VIII	Streptomyces afghaniensis	
A0A059VW47	glucarate dehydratase	VIII	Streptomyces albulus	
X0MKG1	glucarate dehydratase	VIII	Streptomyces albulus	
A0A0B5EY95	glucarate dehydratase	VIII	Streptomyces albus	
M3FSU8	glucarate dehydratase	VIII	Streptomyces bottropensis	
S5UUG0	glucarate dehydratase	VIII	Streptomyces collinus	
A0A0C5FPS0	glucarate dehydratase	VIII	Streptomyces cyaneogriseus	
K4RBD5	glucarate dehydratase	VIII	Streptomyces davawensis	4.2.1.40
A0A081EM66	glucarate dehydratase	VIII	Streptomyces fradiae	
M3DPZ1	glucarate dehydratase	VIII	Streptomyces gancidicus	
A0A072SGS1	glucarate dehydratase	VIII	Streptomyces griseorubens	
G0Q6B3	glucarate dehydratase	VIII	Streptomyces griseus	4.2.1.40
L1KRG6	glucarate dehydratase	VIII	Streptomyces ipomoeae	
L1L4T4	glucarate dehydratase	VIII	Streptomyces ipomoeae	
A0A061A914	glucarate dehydratase	VIII	Streptomyces iranensis	
A0A086N371	glucarate dehydratase	VIII	Streptomyces mutabilis	
V6KKF6	glucarate dehydratase	VIII	Streptomyces niveus	
A0A0B5DHH0	glucarate dehydratase	VIII	Streptomyces nodosus	
A0A066Y7K4	glucarate dehydratase	VIII	Streptomyces olindensis	
A0A066Y7M7	glucarate dehydratase	VIII	Streptomyces olindensis	
A0A0A0NWU7	glucarate dehydratase	VIII	Streptomyces rapamycinicus	
L8EPI3	glucarate dehydratase	VIII	Streptomyces rimosus	
V6JX56	glucarate dehydratase	VIII	Streptomyces roseochromogenus	
A0A086GRF5	glucarate dehydratase	VIII	Streptomyces scabiei	
A0A086H1S0	glucarate dehydratase	VIII	Streptomyces scabiei	
A0A014N684	glucarate dehydratase	VIII	Streptomyces sp.	
A0A022MJY2	glucarate dehydratase	VIII	Streptomyces sp.	
A0A069JQN2	glucarate dehydratase	VIII	Streptomyces sp.	
A0A087K2U5	glucarate dehydratase	VIII	Streptomyces sp.	

Table S2.6. (continuation)

UniProt	Family [3]	Cluster	Species	EC Number
A0A0C1XV54	glucarate dehydratase	VIII	Streptomyces sp.	
A0A0C2AXU5	glucarate dehydratase	VIII	Streptomyces sp.	
A0A0C2AXW0	glucarate dehydratase	VIII	Streptomyces sp.	
A0A0F5AHU5	glucarate dehydratase	VIII	Streptomyces sp.	
A0A0F5VQ93	glucarate dehydratase	VIII	Streptomyces sp.	
S2Y675	glucarate dehydratase	VIII	Streptomyces sp.	
L7F0T0	glucarate dehydratase	VIII	Streptomyces turgidiscabies	
L8P6G4	glucarate dehydratase	VIII	Streptomyces viridochromogenes	
V8CRW5	glucarate dehydratase	VIII	Williamsia sp.	
S1H7Y5	glucarate dehydratase	XI		
U5W655	glucarate dehydratase	XI	Actinoplanes friuliensis	
J5AX65	glucarate dehydratase	XI	Burkholderia multivorans	
L2QC19	glucarate dehydratase	XI	Enterococcus faecium	
A0A029HLH8	glucarate dehydratase	XI	Escherichia coli	
A0A029IKS2	glucarate dehydratase	XI	Escherichia coli	
N2JIF6	glucarate dehydratase	XI	Escherichia coli	
N4NGB3	glucarate dehydratase	XI	Escherichia coli	
N4NK41	glucarate dehydratase	XI	Escherichia coli	
E3XIF3	glucarate dehydratase	XI	Escherichia coli	4.2.1.40
W7WD05	glucarate dehydratase	XI	Methylibium sp.	4.2.1.40
M7S6Q4	glucarate dehydratase	XI	Salmonella enterica	
I6GVI4	glucarate dehydratase	XI	Shigella flexneri	4.2.1.40
I6GVP1	glucarate dehydratase	XI	Shigella flexneri	4.2.1.40
K0X8W5	glucarate dehydratase	XI	Shigella flexneri	4.2.1.40
A0A0C1SRP5	glucarate dehydratase	XI	Streptomyces pluripotens	
A0A069HVU6	L-fuconate dehydratase	VII		
C9VVZ0	L-fuconate dehydratase	VII		
W8GAP6	L-fuconate dehydratase	VII		
S3MX33	L-fuconate dehydratase	VII	Acinetobacter rudis	
L9M6T1	L-fuconate dehydratase	VII	Acinetobacter sp.	
N9QFP6	L-fuconate dehydratase	VII	Acinetobacter sp.	
I0H598	L-fuconate dehydratase	VII	Actinoplanes missouriensis	
W0P8J6	L-fuconate dehydratase	VII	Advenella mimigardefordensis	
Q7CW73	L-fuconate dehydratase	VII	Agrobacterium fabrum	
F7U3B0	L-fuconate dehydratase	VII	Agrobacterium tumefaciens	
G6XU00	L-fuconate dehydratase	VII	Agrobacterium tumefaciens	
B9K0A9	L-fuconate dehydratase	VII	Agrobacterium vitis	
A0A076MYB0	L-fuconate dehydratase	VII	Amycolatopsis methanolica	
J1JVE1	L-fuconate dehydratase	VII	Bartonella tamiae	
U2CSK2	L-fuconate dehydratase	VII	Bifidobacterium breve	
A0A0A8NFK8	L-fuconate dehydratase	VII	Bifidobacterium longum	
A0A0A8NKH7	L-fuconate dehydratase	VII	Bifidobacterium longum	
D6ZWK3	L-fuconate dehydratase	VII	Bifidobacterium longum	
D6ZY08	L-fuconate dehydratase	VII	Bifidobacterium longum	
C0BRF9	L-fuconate dehydratase	VII	Bifidobacterium pseudocat.	
C4INM5	L-fuconate dehydratase	VII	Brucella abortus	
C0G410	L-fuconate dehydratase	VII	Brucella ceti	
A0A083V3G0	L-fuconate dehydratase	VII	Brucella inopinata	
E0DNF3	L-fuconate dehydratase	VII	Brucella inopinata	5.1.2.2
D1CVH9	L-fuconate dehydratase	VII	Brucella sp.	
B1F8C7	L-fuconate dehydratase	VII	Burkholderia ambifaria	
B1SWZ0	L-fuconate dehydratase	VII	Burkholderia ambifaria	
Q0B773	L-fuconate dehydratase	VII	Burkholderia ambifaria	
A0A071MJS2	L-fuconate dehydratase	VII	Burkholderia cenocepacia	
A0A088U0H2	L-fuconate dehydratase	VII	Burkholderia cenocepacia	
A0A088UQW3	L-fuconate dehydratase	VII	Burkholderia cenocepacia	

Table S2.6. (continuation)

UniProt	Family [3]	Cluster	Species	EC Number
A2W3I3	L-fuconate dehydratase	VII	Burkholderia cenocepacia	
A0A081VGB9	L-fuconate dehydratase	VII	Burkholderia cepacia	
A0A088TZQ4	L-fuconate dehydratase	VII	Burkholderia cepacia	
A0A095DJU3	L-fuconate dehydratase	VII	Burkholderia cepacia	
A0A095F7T9	L-fuconate dehydratase	VII	Burkholderia cepacia	
J7JBU0	L-fuconate dehydratase	VII	Burkholderia cepacia	
A2WG49	L-fuconate dehydratase	VII	Burkholderia dolosa	
A0A095XMB3	L-fuconate dehydratase	VII	Burkholderia gladioli	
F2LJB8	L-fuconate dehydratase	VII	Burkholderia gladioli	
A0A095FCX1	L-fuconate dehydratase	VII	Burkholderia mallei	
A0A0B8ZV53	L-fuconate dehydratase	VII	Burkholderia multivorans	
B9AYJ0	L-fuconate dehydratase	VII	Burkholderia multivorans	
B9BRU5	L-fuconate dehydratase	VII	Burkholderia multivorans	
K0DSX9	L-fuconate dehydratase	VII	Burkholderia phenoliruptrix	
A0A087NYB4	L-fuconate dehydratase	VII	Burkholderia pyrrocinia	
A0A084DEJ6	L-fuconate dehydratase	VII	Burkholderia sp.	
B5WPF5	L-fuconate dehydratase	VII	Burkholderia sp.	
D5WDH8	L-fuconate dehydratase	VII	Burkholderia sp.	
F0GA25	L-fuconate dehydratase	VII	Burkholderia sp.	
U2FGT8	L-fuconate dehydratase	VII	Burkholderia sp.	
A4JJB9	L-fuconate dehydratase	VII	Burkholderia vietnamiensis	
W8WU78	L-fuconate dehydratase	VII	Castellaniella defragrans	4.2.1.68
J2H037	L-fuconate dehydratase	VII	Caulobacter sp.	
A0A0A1FAJ5	L-fuconate dehydratase	VII	Collimonas arenae	4.2.1.68
D5NZS3	L-fuconate dehydratase	VII	Corynebacterium ammoniagenes	
K8AE55	L-fuconate dehydratase	VII	Cronobacter condimenti	4.2.1.68
A0A011A0C2	L-fuconate dehydratase	VII	Cryptosporangium arvum	
C9YB86	L-fuconate dehydratase	VII	Curvibacter putative	5.1.2.2
A0A072T5Y7	L-fuconate dehydratase	VII	Delftia tsuruhatensis	
A0A081J3M9	L-fuconate dehydratase	VII	Delftia tsuruhatensis	
A0A087M5A2	L-fuconate dehydratase	VII	Devosia riboflavina	
A0A087LMH8	L-fuconate dehydratase	VII	Devosia sp.	
W8I5M3	L-fuconate dehydratase	VII	Ensifer adhaerens	
D8MW22	L-fuconate dehydratase	VII	Erwinia billingiae	
E3D9N3	L-fuconate dehydratase	VII	Gardnerella vaginalis	
W0RR05	L-fuconate dehydratase	VII	Gemmatirosa kalamazoonesis	
A9HPM6	L-fuconate dehydratase	VII	Gluconacetobacter diazotrophicus	
B5ZFB9	L-fuconate dehydratase	VII	Gluconacetobacter diazotrophicus	
D8J108	L-fuconate dehydratase	VII	Herbaspirillum seropedicae	
I3CXN1	L-fuconate dehydratase	VII	Herbaspirillum sp.	
J2TXM3	L-fuconate dehydratase	VII	Herbaspirillum sp.	
J3HWI1	L-fuconate dehydratase	VII	Herbaspirillum sp.	
Q28QJ1	L-fuconate dehydratase	VII	Jannaschia sp.	
A0A090G1V9	L-fuconate dehydratase	VII	Mesorhizobium plurifarium	4.2.1.68
A0A090DDL7	L-fuconate dehydratase	VII	Mesorhizobium sp.	4.2.1.68
A0A022L1F8	L-fuconate dehydratase	VII	Microbacterium sp.	
A0A022LFJ0	L-fuconate dehydratase	VII	Microbacterium sp.	
A0A062VT92	L-fuconate dehydratase	VII	Microbacterium sp.	
A0A0D0JR50	L-fuconate dehydratase	VII	Microbacterium sp.	
W2EM32	L-fuconate dehydratase	VII	Microbispora sp.	
W2F1G5	L-fuconate dehydratase	VII	Microbispora sp.	
A0A0B2BDS5	L-fuconate dehydratase	VII	Mumia flava	
I5C6L5	L-fuconate dehydratase	VII	Nitratireductor aquibiodomus	
A0A084U5E3	L-fuconate dehydratase	VII	Nitratireductor basaltis	
T0I2U5	L-fuconate dehydratase	VII	Novosphingobium lindaniclasticum	
Q2CHH7	L-fuconate dehydratase	VII	Oceanicola granulosus	

Table S2.6. (continuation)

UniProt	Family [3]	Cluster	Species	EC Number
A6WVP6	L-fuconate dehydratase	VII	Ochrobactrum anthropi	
U4VEX1	L-fuconate dehydratase	VII	Ochrobactrum intermedium	
M3JAA8	L-fuconate dehydratase	VII	Ochrobactrum sp.	
U1YW51	L-fuconate dehydratase	VII	Ochrobactrum sp.	
A0A0F5XW32	L-fuconate dehydratase	VII	Pantoea sp.	
J2ULU9	L-fuconate dehydratase	VII	Pantoea sp.	
A0A099G2Q6	L-fuconate dehydratase	VII	Paracoccus sanguinis	
J3CF78	L-fuconate dehydratase	VII	Phyllobacterium sp.	
D5SU39	L-fuconate dehydratase	VII	Planctopirus limnophila	
A0A076K6Y6	L-fuconate dehydratase	VII	Planktomarina temperata	
A0A076JWA4	L-fuconate dehydratase	VII	Planktomarina temperata	5.1.2.2
A0A089PL92	L-fuconate dehydratase	VII	Pluralibacter gergoviae	
J3DD58	L-fuconate dehydratase	VII	Polaromonas sp.	
M4WSG7	L-fuconate dehydratase	VII	Pseudomonas denitrificans	
A0A098SWP5	L-fuconate dehydratase	VII	Pseudomonas lutea	
Q88J18	L-fuconate dehydratase	VII	Pseudomonas putida	
J1INB8	L-fuconate dehydratase	VII	Pseudomonas sp.	
A0A060IES7	L-fuconate dehydratase	VII	Rhizobium etli	
Q2JZW8	L-fuconate dehydratase	VII	Rhizobium etli	
S5SSX1	L-fuconate dehydratase	VII	Rhizobium etli	
B6A481	L-fuconate dehydratase	VII	Rhizobium leguminosarum	
C6B7Z9	L-fuconate dehydratase	VII	Rhizobium leguminosarum	
I9N4G0	L-fuconate dehydratase	VII	Rhizobium leguminosarum	
J0K1Y9	L-fuconate dehydratase	VII	Rhizobium leguminosarum	
J0KLA7	L-fuconate dehydratase	VII	Rhizobium leguminosarum	
J0L5M6	L-fuconate dehydratase	VII	Rhizobium leguminosarum	
Q1M5D8	L-fuconate dehydratase	VII	Rhizobium leguminosarum	
W0INX3	L-fuconate dehydratase	VII	Rhizobium leguminosarum	
Q92VK9	L-fuconate dehydratase	VII	Rhizobium meliloti	5.1.2.2
A0A037Y0B6	L-fuconate dehydratase	VII	Rhizobium radiobacter	
A0A083ZHL0	L-fuconate dehydratase	VII	Rhizobium radiobacter	
A0A061MQD4	L-fuconate dehydratase	VII	Rhizobium rhizogenes	
A0A081MHL3	L-fuconate dehydratase	VII	Rhizobium sp.	
A0A095VJ22	L-fuconate dehydratase	VII	Rhizobium sp.	
C3KMJ8	L-fuconate dehydratase	VII	Rhizobium sp.	
J1TI24	L-fuconate dehydratase	VII	Rhizobium sp.	
J2W715	L-fuconate dehydratase	VII	Rhizobium sp.	
J6DSB7	L-fuconate dehydratase	VII	Rhizobium sp.	
L0NAS3	L-fuconate dehydratase	VII	Rhizobium sp.	
W6RG88	L-fuconate dehydratase	VII	Rhizobium sp.	5.1.2.2
V7EQS1	L-fuconate dehydratase	VII	Rhodobacter sp.	
Q21TR0	L-fuconate dehydratase	VII	Rhodoferax ferrireducens	
A4EPQ5	L-fuconate dehydratase	VII	Roseobacter sp.	
Q1GLV3	L-fuconate dehydratase	VII	Ruegeria sp.	
H5XDI0	L-fuconate dehydratase	VII	Saccharomonospora cyanea	
I1CZT1	L-fuconate dehydratase	VII	Saccharomonospora glauca	
H5X2I2	L-fuconate dehydratase	VII	Saccharomonospora marina	
T2RV12	L-fuconate dehydratase	VII	Saccharopolyspora erythraea	
A0A084IHZ8	L-fuconate dehydratase	VII	Salinisphaera hydrothermalis	
A0A084YQ11	L-fuconate dehydratase	VII	Serratia grimesii	
A0A069CNQ1	L-fuconate dehydratase	VII	Serratia liquefaciens	
A0A0A5MLT0	L-fuconate dehydratase	VII	Serratia marcescens	
L0MCE9	L-fuconate dehydratase	VII	Serratia marcescens	
W0T196	L-fuconate dehydratase	VII	Serratia marcescens	
A0A086G7V5	L-fuconate dehydratase	VII	Serratia nematodiphila	
D4DXJ3	L-fuconate dehydratase	VII	Serratia odorifera	

Table S2.6. (continuation)

UniProt	Family [3]	Cluster	Species	EC Number
S0A8X1	L-fuconate dehydratase	VII	Serratia plymuthica	4.2.1.68
A8G9F6	L-fuconate dehydratase	VII	Serratia proteamaculans	
A0A021WZY7	L-fuconate dehydratase	VII	Shinella sp.	4.2.1.68
A0A072CFH4	L-fuconate dehydratase	VII	Sinorhizobium americanum	
I3X2W5	L-fuconate dehydratase	VII	Sinorhizobium fredii	
A6UJC0	L-fuconate dehydratase	VII	Sinorhizobium medicae	
W0A9H6	L-fuconate dehydratase	VII	Sphingomonas sanxanigenens	
D3QAP7	L-fuconate dehydratase	VII	Stackebrandtia nassauensis	
X0MJN8	L-fuconate dehydratase	VII	Streptomyces albulus	
A0A0A8EVS5	L-fuconate dehydratase	VII	Streptomyces sp.	
H0B9K2	L-fuconate dehydratase	VII	Streptomyces sp.	
D2AYS8	L-fuconate dehydratase	VII	Streptosporangium roseum	
A0A095TJH0	L-fuconate dehydratase	VII	Tatumella morbirosei	
A0A074JW81	L-fuconate dehydratase	VII	Thioclava sp.	
T1W5N6	L-fuconate dehydratase	VII	uncultured organism	
C5CZB5	L-fuconate dehydratase	VII	Variovorax paradoxus	
T1XL07	L-fuconate dehydratase	VII	Variovorax paradoxus	
I3PCI6	L-fuconate dehydratase	VII	Variovorax sp.	
J3CTV8	L-fuconate dehydratase	VII	Variovorax sp.	
A1WGV3	L-fuconate dehydratase	VII	Verminephrobacter eiseniae	
Q8PF00	L-fuconate dehydratase	VII	Xanthomonas axonopodis	
Q66C33	L-fuconate dehydratase	VII	Yersinia pseudotuberculosis	
N8YF24	L-fuconate dehydratase	XI	Acinetobacter guillouiae	
F5J705	L-fuconate dehydratase	XI	Agrobacterium sp.	
H0HD24	L-fuconate dehydratase	XI	Agrobacterium tumefaciens	
S2WHX9	L-fuconate dehydratase	XI	Delftia acidovorans	
K2P3Z7	L-fuconate dehydratase	XI	Galbibacter marinus	
U7FXT1	L-fuconate dehydratase	XI	Labrenzia sp.	
F5XLP1	L-fuconate dehydratase	XI	Microlunatus phosphovorus	
H0JDC3	L-fuconate dehydratase	XI	Pseudomonas psychrotolerans	
N2J7H6	L-fuconate dehydratase	XI	Pseudomonas sp.	
F2AJQ0	L-fuconate dehydratase	XI	Rhizobium etli	
G9AEN7	L-fuconate dehydratase	XI	Rhizobium fredii	
F0SPZ4	L-fuconate dehydratase	XI	Rubinisphaera brasiliensis	
S9RP95	L-fuconate dehydratase	XI	Salipiger mucosus	4.2.1.68
N1MQP4	L-fuconate dehydratase	XI	Sphingobium japonicum	4.2.1.68
F3ND86	L-fuconate dehydratase	XI	Streptomyces griseoaurantiacus	
L8EU13	L-fuconate dehydratase	XI	Streptomyces rimosus	
K2AZV9	L-fuconate dehydratase	XI	uncultured bacterium	
E6V1J7	L-fuconate dehydratase	XI	Variovorax paradoxus	
V0G6W7	L-talarate/galactarate dehydratase	IV		
X7IBV6	L-talarate/galactarate dehydratase	IV		
J0UFI5	L-talarate/galactarate dehydratase	IV	Acidovorax sp.	
W8FLW8	L-talarate/galactarate dehydratase	IV	Agrobacterium tumefaciens	4.2.1.42
A1R0V5	L-talarate/galactarate dehydratase	IV	Arthrobacter aurescens	5.1.2.2
B8H867	L-talarate/galactarate dehydratase	IV	Arthrobacter chlorophenolicus	
N1UTC7	L-talarate/galactarate dehydratase	IV	Arthrobacter crystallopoietes	
L8TNR0	L-talarate/galactarate dehydratase	IV	Arthrobacter nitrophenolicus	
A0A0B4ETS6	L-talarate/galactarate dehydratase	IV	Arthrobacter phenanthrenivorans	
F0M4Q6	L-talarate/galactarate dehydratase	IV	Arthrobacter phenanthrenivorans	4.2.1.-; 4.2.1.42
A0A0C1C2S3	L-talarate/galactarate dehydratase	IV	Arthrobacter sp.	
U1Y614	L-talarate/galactarate dehydratase	IV	Arthrobacter sp.	
A0JQW3	L-talarate/galactarate dehydratase	IV	Arthrobacter sp.	4.2.1.-; 4.2.1.42
J7JBW5	L-talarate/galactarate dehydratase	IV	Burkholderia cepacia	4.2.1.42
A0A0B6S138	L-talarate/galactarate dehydratase	IV	Burkholderia glumae	5.1.2.2
A0A095H7Q5	L-talarate/galactarate dehydratase	IV	Burkholderia mallei	

Table S2.6. (continuation)

UniProt	Family [3]	Cluster	Species	EC Number
A0A0B1Y2J2	L-talarate/galactarate dehydratase	IV	Burkholderia sp.	
K8RIQ2	L-talarate/galactarate dehydratase	IV	Burkholderia sp.	
A0A0C2BZ07	L-talarate/galactarate dehydratase	IV	Burkholderia sp.	5.1.2.2
A0A0F5SEH2	L-talarate/galactarate dehydratase	IV	Citrobacter amalonaticus	
M1K6Q3	L-talarate/galactarate dehydratase	IV	Citrobacter amalonaticus	
A0A0A1RT97	L-talarate/galactarate dehydratase	IV	Citrobacter pasteurii	5.1.2.2
R8WWB6	L-talarate/galactarate dehydratase	IV	Citrobacter sp.	
H1SHW0	L-talarate/galactarate dehydratase	IV	Cupriavidus basilensis	
A0A0C4YGA5	L-talarate/galactarate dehydratase	IV	Cupriavidus basilensis	4.2.1.42
Q46S91	L-talarate/galactarate dehydratase	IV	Cupriavidus pinatubonensis	4.2.1.-; 4.2.1.42
L2E811	L-talarate/galactarate dehydratase	IV	Cupriavidus sp.	
V2IP29	L-talarate/galactarate dehydratase	IV	Cupriavidus sp.	
A9BLY2	L-talarate/galactarate dehydratase	IV	Delftia acidovorans	
A0A031I2E2	L-talarate/galactarate dehydratase	IV	Delftia sp.	
F6B1U4	L-talarate/galactarate dehydratase	IV	Delftia sp.	5.1.2.2
A0A072T891	L-talarate/galactarate dehydratase	IV	Delftia tsuruhatensis	
A0A0A3ZQS1	L-talarate/galactarate dehydratase	IV	Dickeya chrysanthemi	
C6C955	L-talarate/galactarate dehydratase	IV	Dickeya dadantii	
D2BRK3	L-talarate/galactarate dehydratase	IV	Dickeya dadantii	
E0SJZ7	L-talarate/galactarate dehydratase	IV	Dickeya dadantii	
U6ZQB6	L-talarate/galactarate dehydratase	IV	Dickeya solani	
A0A0A8F8E1	L-talarate/galactarate dehydratase	IV	Dickeya zeae	
C6CKK6	L-talarate/galactarate dehydratase	IV	Dickeya zeae	
A0A0A3YKA8	L-talarate/galactarate dehydratase	IV	Enterobacter cancerogenus	
R9VJ16	L-talarate/galactarate dehydratase	IV	Enterobacter sp.	
A0A0A3Z4D2	L-talarate/galactarate dehydratase	IV	Erwinia typographi	
F0JQ37	L-talarate/galactarate dehydratase	IV	Escherichia fergusonii	
B7LQU9	L-talarate/galactarate dehydratase	IV	Escherichia fergusonii	5.1.2.2
A0A086D652	L-talarate/galactarate dehydratase	IV	Gammaproteobacteria bacterium	
D2S478	L-talarate/galactarate dehydratase	IV	Geodermatophilus obscurus	
A0A068HCA1	L-talarate/galactarate dehydratase	IV	Klebsiella oxytoca	
A0A0E0X119	L-talarate/galactarate dehydratase	IV	Klebsiella oxytoca	
J5Y2R0	L-talarate/galactarate dehydratase	IV	Klebsiella sp.	
A0A0C7KUF3	L-talarate/galactarate dehydratase	IV	Klebsiella variicola	
R5XF46	L-talarate/galactarate dehydratase	IV	Klebsiella variicola	
A0A063XXA3	L-talarate/galactarate dehydratase	IV	Kosakonia radicincitans	
W6IYA6	L-talarate/galactarate dehydratase	IV	Kosakonia sacchari	
A0A071LW10	L-talarate/galactarate dehydratase	IV	Mangrovibacter sp.	
A0A081FZJ1	L-talarate/galactarate dehydratase	IV	Marinobacterium sp.	4.2.1.113
A0A098U8R2	L-talarate/galactarate dehydratase	IV	Massilia sp.	
B8IEI0	L-talarate/galactarate dehydratase	IV	Methylobacterium nodulans	
B0UQI2	L-talarate/galactarate dehydratase	IV	Methylobacterium sp.	
A0A0E9BI78	L-talarate/galactarate dehydratase	IV	Pantoea ananas	4.2.1.-; 4.2.1.42
A0A0B1RBJ6	L-talarate/galactarate dehydratase	IV	Pantoea rodasii	
A0A0F3LN40	L-talarate/galactarate dehydratase	IV	Pantoea sp.	
E6WJY7	L-talarate/galactarate dehydratase	IV	Pantoea sp.	
Q6D8H6	L-talarate/galactarate dehydratase	IV	Pectobacterium atrosepticum	
A0A093SIS0	L-talarate/galactarate dehydratase	IV	Pectobacterium carotovorum	
A0A0B2WC64	L-talarate/galactarate dehydratase	IV	Pectobacterium carotovorum	
A0A0B3YB60	L-talarate/galactarate dehydratase	IV	Pectobacterium carotovorum	
A0A0B9CSP0	L-talarate/galactarate dehydratase	IV	Pectobacterium carotovorum	
C6DAG0	L-talarate/galactarate dehydratase	IV	Pectobacterium carotovorum	
D0KK81	L-talarate/galactarate dehydratase	IV	Pectobacterium wasabiae	
Q12GE3	L-talarate/galactarate dehydratase	IV	Polaromonas sp.	4.2.1.-; 4.2.1.42
A0A0C2E9C3	L-talarate/galactarate dehydratase	IV	Pseudomonas batumici	
A0A0B1Z0I1	L-talarate/galactarate dehydratase	IV	Pseudomonas frederiksbergensis	

Table S2.6. (continuation)

UniProt	Family [3]	Cluster	Species	EC Number
A0A0D0KAZ5	L-talarate/galactarate dehydratase	IV	Pseudomonas fulva	
F6AHR9	L-talarate/galactarate dehydratase	IV	Pseudomonas fulva	5.1.2.2
A0A0F4XQ41	L-talarate/galactarate dehydratase	IV	Pseudomonas kilonensis	
F4DUI4	L-talarate/galactarate dehydratase	IV	Pseudomonas mendocina	
A0A028VA29	L-talarate/galactarate dehydratase	IV	Pseudomonas pseudoalcaligenes	
A0A077LIG7	L-talarate/galactarate dehydratase	IV	Pseudomonas sp.	
S6I772	L-talarate/galactarate dehydratase	IV	Pseudomonas sp.	
S6IQI7	L-talarate/galactarate dehydratase	IV	Pseudomonas sp.	
W8R7R6	L-talarate/galactarate dehydratase	IV	Pseudomonas stutzeri	
A0A085VH12	L-talarate/galactarate dehydratase	IV	Pseudomonas syringae	
A0A085VKG1	L-talarate/galactarate dehydratase	IV	Pseudomonas syringae	
K2T3N5	L-talarate/galactarate dehydratase	IV	Pseudomonas syringae	
U3QRF0	L-talarate/galactarate dehydratase	IV	Ralstonia pickettii	
A0A0D0K9V1	L-talarate/galactarate dehydratase	IV	Rhizobium radiobacter	
A9MKR9	L-talarate/galactarate dehydratase	IV	Salmonella arizonae	
E8XG12	L-talarate/galactarate dehydratase	IV	Salmonella typhimurium	
Q8ZL58	L-talarate/galactarate dehydratase	IV	Salmonella typhimurium	4.2.1.156; 4.2.1.4
D7C3X1	L-talarate/galactarate dehydratase	IV	Streptomyces bingchenggensis	
A0A061A354	L-talarate/galactarate dehydratase	IV	Streptomyces iranensis	
A0A0A0NX13	L-talarate/galactarate dehydratase	IV	Streptomyces rapamycinicus	
A0A014M5C7	L-talarate/galactarate dehydratase	IV	Streptomyces sp.	
G2P402	L-talarate/galactarate dehydratase	IV	Streptomyces violaceusniger	
H0F6W4	L-talarate/galactarate dehydratase	XI	Achromobacter arsenitoxydans	
H0QKP2	L-talarate/galactarate dehydratase	XI	Arthrobacter globiformis	
G8Q889	L-talarate/galactarate dehydratase	XI	Pseudomonas fluorescens	5.1.2.2
H2JV15	L-talarate/galactarate dehydratase	XI	Streptomyces hygroscopicus	
H0FAQ8	mannonate dehydratase	I	Achromobacter arsenitoxydans	
F7T116	mannonate dehydratase	I	Achromobacter insuavis	
J4Y6C9	mannonate dehydratase	I	Achromobacter piechaudii	
D4X6T3	mannonate dehydratase	I	Achromobacter piechaudii	5.1.2.2
A0A095UMT3	mannonate dehydratase	I	Achromobacter sp.	
E3HXJ3	mannonate dehydratase	I	Achromobacter xylosoxidans	
A0A0A5J9Z3	mannonate dehydratase	I	Alcaligenes xylosoxydans	
A0A0D6FQ08	mannonate dehydratase	I	Alcaligenes xylosoxydans	
A0A075P439	mannonate dehydratase	I	Alteromonas australica	
V4RBL5	mannonate dehydratase	I	Asticcacaulis benevestitus	
F4QMQ4	mannonate dehydratase	I	Asticcacaulis biprosthecum	
F4QU87	mannonate dehydratase	I	Asticcacaulis biprosthecum	
E8RU01	mannonate dehydratase	I	Asticcacaulis excentricus	
V4NI00	mannonate dehydratase	I	Asticcacaulis sp.	
V4P143	mannonate dehydratase	I	Asticcacaulis sp.	
V4PIC3	mannonate dehydratase	I	Asticcacaulis sp.	
V4PVX3	mannonate dehydratase	I	Asticcacaulis sp.	
V4R7I3	mannonate dehydratase	I	Asticcacaulis sp.	
V4YRX5	mannonate dehydratase	I	Betaproteobacteria bacterium	
W9BWV7	mannonate dehydratase	I	Blastomonas sp.	
A9I6B4	mannonate dehydratase	I	Bordetella petrii	
U2Z8M6	mannonate dehydratase	I	Brevundimonas abyssalis	
H5WQ02	mannonate dehydratase	I	Burkholderiales bacterium	
Q9A4L8	mannonate dehydratase	I	Caulobacter crescentus	4.2.1.8
Q9AAR4	mannonate dehydratase	I	Caulobacter crescentus	4.2.1.8
R0EHE5	mannonate dehydratase	I	Caulobacter crescentus	4.2.1.8
D5VMM7	mannonate dehydratase	I	Caulobacter segnis	
J2GJ67	mannonate dehydratase	I	Caulobacter sp.	
J2GPJ8	mannonate dehydratase	I	Caulobacter sp.	
B0T0B1	mannonate dehydratase	I	Caulobacter sp.	4.2.1.8

Table S2.6. (continuation)

UniProt	Family [3]	Cluster	Species	EC Number
B0T4L2	mannonate dehydratase	I	Caulobacter sp.	4.2.1.8
A0A0C4YFH8	mannonate dehydratase	I	Cupriavidus basilensis	
C9YAX2	mannonate dehydratase	I	Curvibacter putative	4.2.1.6
J2UKC4	mannonate dehydratase	I	Herbaspirillum sp.	
C6XQG0	mannonate dehydratase	I	Hirschia baltica	
A0A059FRT6	mannonate dehydratase	I	Hyphomonas johnsonii	
G1Y3L2	mannonate dehydratase	I	Nitrospirillum amazonense	
A4XF23	mannonate dehydratase	I	Novosphingobium aromat.	4.2.1.8
T0H4U1	mannonate dehydratase	I	Novosphingobium lindaniclasticum	
A0A031JH68	mannonate dehydratase	I	Novosphingobium resinovorum	
A0A031JTM9	mannonate dehydratase	I	Novosphingobium resinovorum	
A0A0D1CEP0	mannonate dehydratase	I	Novosphingobium sp.	
F6IDM5	mannonate dehydratase	I	Novosphingobium sp.	
A0A0B8ZFC8	mannonate dehydratase	I	Novosphingobium subterraneum	
A0A0B8ZFH2	mannonate dehydratase	I	Novosphingobium subterraneum	
B4RGH9	mannonate dehydratase	I	Phenylobacterium zucineum	
Q125C0	mannonate dehydratase	I	Polaromonas sp.	4.2.1.8
A0A0B2BT97	mannonate dehydratase	I	Porphyrobacter mercurialis	
W1KQH7	mannonate dehydratase	I	Sphingobium chinhatense	
T0HEJ9	mannonate dehydratase	I	Sphingobium lactosutens	
T0HV29	mannonate dehydratase	I	Sphingobium lactosutens	
A0A013VKJ9	mannonate dehydratase	I	Sphingobium sp.	
A0A013WWB4	mannonate dehydratase	I	Sphingobium sp.	
A0A087N933	mannonate dehydratase	I	Sphingobium sp.	
A0A087NFW2	mannonate dehydratase	I	Sphingobium sp.	
J2CPU4	mannonate dehydratase	I	Sphingobium sp.	
W1S5M2	mannonate dehydratase	I	Sphingobium sp.	
T0JBK1	mannonate dehydratase	I	Sphingobium ummariense	
A0A084EGM9	mannonate dehydratase	I	Sphingobium yanoikuyae	
A0A0C5L1K3	mannonate dehydratase	I	Sphingomonas hengshuiensis	
W0A6S3	mannonate dehydratase	I	Sphingomonas sanxanigenens	
W0A866	mannonate dehydratase	I	Sphingomonas sanxanigenens	
A0A031HZI8	mannonate dehydratase	I	Sphingomonas sp.	
A0A0A1YAF5	mannonate dehydratase	I	Sphingomonas sp.	
J8VM37	mannonate dehydratase	I	Sphingomonas sp.	
M4S302	mannonate dehydratase	I	Sphingomonas sp.	
M4S4D7	mannonate dehydratase	I	Sphingomonas sp.	
Q1NAJ2	mannonate dehydratase	I	Sphingomonas sp.	4.2.1.8
A0A097EFX3	mannonate dehydratase	I	Sphingomonas taxi	
A5V7I3	mannonate dehydratase	I	Sphingomonas wittichii	
A5V6Z0	mannonate dehydratase	I	Sphingomonas wittichii	4.2.1.8
A0A0A7PLG2	mannonate dehydratase	I	Sphingopyxis fribergensis	
A0A095ATF6	mannonate dehydratase	I	Sphingopyxis sp.	
A0A095B6E7	mannonate dehydratase	I	Sphingopyxis sp.	
A0A095C0I5	mannonate dehydratase	I	Sphingopyxis sp.	
T1WB97	mannonate dehydratase	I	uncultured organism	
C5CMZ1	mannonate dehydratase	I	Variovorax paradoxus	
T1XA18	mannonate dehydratase	I	Variovorax paradoxus	
J3CQN2	mannonate dehydratase	I	Variovorax sp.	
A1WNS2	mannonate dehydratase	I	Verminephrobacter eiseniae	
V4NNA5	mannonate dehydratase	XI	Asticcacaulis sp.	
G1XZX5	mannonate dehydratase	XI	Nitrospirillum amazonense	
I9C4A3	mannonate dehydratase	XI	Novosphingobium sp.	
J3DGV2	mannonate dehydratase	XI	Polaromonas sp.	
A0A027YWU5	methylaspartate ammonia-lyase	XI		
A0A0A5IKW1	methylaspartate ammonia-lyase	XI		

Table S2.6. (continuation)

UniProt	Family [3]	Cluster	Species	EC Number
T0JWX2	methylaspartate ammonia-lyase	XI		
D9QUW7	methylaspartate ammonia-lyase	XI	Acetohalobium arabaticum	4.3.1.2
G5GA80	methylaspartate ammonia-lyase	XI	Alloprevotella rava	
Q7W411	methylaspartate ammonia-lyase	XI	Bordetella parapertussis	4.3.1.2
Q7W579	methylaspartate ammonia-lyase	XI	Bordetella parapertussis	4.3.1.2
A0A0A0XJJ5	methylaspartate ammonia-lyase	XI	Bordetella pertussis	4.3.1.2
A0A0A0XNM5	methylaspartate ammonia-lyase	XI	Bordetella pertussis	4.3.1.2
I7J5P0	methylaspartate ammonia-lyase	XI	Caloramator australicus	4.3.1.2
U7VCU6	methylaspartate ammonia-lyase	XI	Cetobacterium somerae	
O66145	methylaspartate ammonia-lyase	XI	Citrobacter amalonaticus	4.3.1.2
U2DG57	methylaspartate ammonia-lyase	XI	Clostridiales bacterium	
C6PXL1	methylaspartate ammonia-lyase	XI	Clostridium carboxidivorans	4.3.1.2
C6PZ59	methylaspartate ammonia-lyase	XI	Clostridium carboxidivorans	4.3.1.2
D8GIL1	methylaspartate ammonia-lyase	XI	Clostridium ljungdahlii	4.3.1.2
R6AL32	methylaspartate ammonia-lyase	XI	Clostridium sp.	
R6BP01	methylaspartate ammonia-lyase	XI	Clostridium sp.	
T0N769	methylaspartate ammonia-lyase	XI	Clostridium sp.	
U2DNR7	methylaspartate ammonia-lyase	XI	Clostridium sp.	
Q890S3	methylaspartate ammonia-lyase	XI	Clostridium tetani	4.3.1.2
Q05514	methylaspartate ammonia-lyase	XI	Clostridium tetanomorphum	4.3.1.2
I4ADL3	methylaspartate ammonia-lyase	XI	Desulfitobacterium dehalogenans	4.3.1.2
G9XVP7	methylaspartate ammonia-lyase	XI	Desulfitobacterium hafniense	
Q24SH0	methylaspartate ammonia-lyase	XI	Desulfitobacterium hafniense	
A0A098B3F3	methylaspartate ammonia-lyase	XI	Desulfitobacterium hafniense	4.3.1.2
W0EB38	methylaspartate ammonia-lyase	XI	Desulfitobacterium metallireducens	
I4DBP3	methylaspartate ammonia-lyase	XI	Desulfosporosinus acidiphilus	4.3.1.2
J7IWG8	methylaspartate ammonia-lyase	XI	Desulfosporosinus meridiei	4.3.1.2
G7W9T9	methylaspartate ammonia-lyase	XI	Desulfosporosinus orientis	
A0A099SFX4	methylaspartate ammonia-lyase	XI	Desulfosporosinus sp.	
G2FLZ0	methylaspartate ammonia-lyase	XI	Desulfosporosinus sp.	4.3.1.2
H5Y029	methylaspartate ammonia-lyase	XI	Desulfosporosinus youngiae	
E3G175	methylaspartate ammonia-lyase	XI	Enterobacter lignolyticus	4.3.1.2
V0SFM8	methylaspartate ammonia-lyase	XI	Escherichia coli	
B7LKN2	methylaspartate ammonia-lyase	XI	Escherichia fergusonii	4.3.1.2
H1PN72	methylaspartate ammonia-lyase	XI	Eubacterium infirmum	
R5J397	methylaspartate ammonia-lyase	XI	Firmicutes bacterium	
R6HBC9	methylaspartate ammonia-lyase	XI	Firmicutes bacterium	
R6R2W7	methylaspartate ammonia-lyase	XI	Firmicutes bacterium	
E3DQR4	methylaspartate ammonia-lyase	XI	Halanaerobium praevalens	4.3.1.2
Q5V465	methylaspartate ammonia-lyase	XI	Haloarcula marismortui	4.3.1.2
B0R7I5	methylaspartate ammonia-lyase	XI	Halobacterium salinarum	4.3.1.2
U2EGW8	methylaspartate ammonia-lyase	XI	Haloplasma contractile	4.3.1.2
E3H6D1	methylaspartate ammonia-lyase	XI	Ilyobacter polytropus	4.3.1.2
A0A0A2R940	methylaspartate ammonia-lyase	XI	Morganella morganii	
I9B6S7	methylaspartate ammonia-lyase	XI	Pelosinus fermentans	
E0NU52	methylaspartate ammonia-lyase	XI	Prevotella marshii	4.3.1.2
V8CPQ9	methylaspartate ammonia-lyase	XI	Prevotella nigrescens	
Q98A98	methylaspartate ammonia-lyase	XI	Rhizobium loti	
Q8KJF8	methylaspartate ammonia-lyase	XI	Rhizobium loti	4.3.1.2
A9MJL0	methylaspartate ammonia-lyase	XI	Salmonella arizonae	
R7RTR7	methylaspartate ammonia-lyase	XI	Thermobrachium celere	4.3.1.2
E9S201	methylaspartate ammonia-lyase	XI	Treponema denticola	
F5YRD1	methylaspartate ammonia-lyase	XI	Treponema primitia	4.3.1.2
A0A0E1NIT1	methylaspartate ammonia-lyase	XI	Yersinia enterocolitica	4.3.1.2
G9YZ23	methylaspartate ammonia-lyase	XI	Yokenella regensburgei	
A0A0D1YIW7	N-succinylamino acid racemase 2	IX	Aneurinibacillus migulanus	

Table S2.6. (continuation)

UniProt	Family [3]	Cluster	Species	EC Number
M5RC02	N-succinylamino acid racemase 2	IX	Anoxybacillus sp.	
A0A0C2UFA4	N-succinylamino acid racemase 2	IX	Bacillus badius	
A0A0C2XVL2	N-succinylamino acid racemase 2	IX	Bacillus badius	
A0A0C2XYY1	N-succinylamino acid racemase 2	IX	Bacillus badius	
A0A0C2Y619	N-succinylamino acid racemase 2	IX	Bacillus badius	
C2MUG2	N-succinylamino acid racemase 2	IX	Bacillus cereus	
C2TAZ2	N-succinylamino acid racemase 2	IX	Bacillus cereus	
C2UQ68	N-succinylamino acid racemase 2	IX	Bacillus cereus	
C2VNB0	N-succinylamino acid racemase 2	IX	Bacillus cereus	
C2YLC4	N-succinylamino acid racemase 2	IX	Bacillus cereus	
D7WK27	N-succinylamino acid racemase 2	IX	Bacillus cereus	
J7XYI6	N-succinylamino acid racemase 2	IX	Bacillus cereus	
J8GZ12	N-succinylamino acid racemase 2	IX	Bacillus cereus	
J8MRV8	N-succinylamino acid racemase 2	IX	Bacillus cereus	
M4H9M3	N-succinylamino acid racemase 2	IX	Bacillus cereus	
R8C152	N-succinylamino acid racemase 2	IX	Bacillus cereus	
R8GS06	N-succinylamino acid racemase 2	IX	Bacillus cereus	
R8H067	N-succinylamino acid racemase 2	IX	Bacillus cereus	
R8I794	N-succinylamino acid racemase 2	IX	Bacillus cereus	
R8MAP9	N-succinylamino acid racemase 2	IX	Bacillus cereus	
R8PRZ7	N-succinylamino acid racemase 2	IX	Bacillus cereus	
R8R0E3	N-succinylamino acid racemase 2	IX	Bacillus cereus	
R8U971	N-succinylamino acid racemase 2	IX	Bacillus cereus	
R8UHV0	N-succinylamino acid racemase 2	IX	Bacillus cereus	
S3IPR5	N-succinylamino acid racemase 2	IX	Bacillus cereus	
Q81IL5	N-succinylamino acid racemase 2	IX	Bacillus cereus	5.1.1.-
A0A073KAX9	N-succinylamino acid racemase 2	IX	Bacillus gaemokensis	
K1KR47	N-succinylamino acid racemase 2	IX	Bacillus isronensis	5.1.1.-
A0A073JYA4	N-succinylamino acid racemase 2	IX	Bacillus manliponensis	
A0A0A0EAZ6	N-succinylamino acid racemase 2	IX	Bacillus niacini	
A0A0A8JNE0	N-succinylamino acid racemase 2	IX	Bacillus sp.	
A0A0F5RII5	N-succinylamino acid racemase 2	IX	Bacillus sp.	
A0A090IYM4	N-succinylamino acid racemase 2	IX	Bacillus thermoamylovorans	
A0A0D0G5Q9	N-succinylamino acid racemase 2	IX	Bacillus thermoamylovorans	
C3FXT1	N-succinylamino acid racemase 2	IX	Bacillus thuringiensis	
K0FWT6	N-succinylamino acid racemase 2	IX	Bacillus thuringiensis	
A0A0F6J3M5	N-succinylamino acid racemase 2	IX	Bacillus thuringiensis	5.1.1.-
A0A084IXX4	N-succinylamino acid racemase 2	IX	Bacillus weihenstephanensis	
M7PBF2	N-succinylamino acid racemase 2	IX	Bhargavaea cecembensis	5.1.1.-
M8DER0	N-succinylamino acid racemase 2	IX	Brevibacillus borstelensis	
C0Z5K7	N-succinylamino acid racemase 2	IX	Brevibacillus brevis	
V6M482	N-succinylamino acid racemase 2	IX	Brevibacillus panacihumi	
J3A5R9	N-succinylamino acid racemase 2	IX	Brevibacillus sp.	
A0A099DGP9	N-succinylamino acid racemase 2	IX	Exiguobacterium mexicanum	
A0A069EYI7	N-succinylamino acid racemase 2	IX	Exiguobacterium sp.	
A0A0C2VQA1	N-succinylamino acid racemase 2	IX	Jeotgalibacillus alimentarius	
A0A0B5AVV5	N-succinylamino acid racemase 2	IX	Jeotgalibacillus sp.	4.2.1.113
A0A0A3IX08	N-succinylamino acid racemase 2	IX	Lysinibacillus manganicus	
A0A0A3JRG7	N-succinylamino acid racemase 2	IX	Lysinibacillus massiliensis	
A0A0A3IMC2	N-succinylamino acid racemase 2	IX	Lysinibacillus odysseyi	
A0A0A3I076	N-succinylamino acid racemase 2	IX	Lysinibacillus sinduriensis	
A0A078MF35	N-succinylamino acid racemase 2	IX	Lysinibacillus sp.	
A0A087N303	N-succinylamino acid racemase 2	IX	Lysinibacillus sp.	
I4X6U2	N-succinylamino acid racemase 2	IX	Planococcus antarcticus	
E7RGL1	N-succinylamino acid racemase 2	IX	Planococcus donghaensis	
W3ACV6	N-succinylamino acid racemase 2	IX	Planomicrobium glaciei	

Table S2.6. (continuation)

UniProt	Family [3]	Cluster	Species	EC Number
A0A0A5G1S4	N-succinylamino acid racemase 2	IX	Pontibacillus litoralis	
F2F431	N-succinylamino acid racemase 2	IX	Solibacillus silvestris	
A0A075JRY6	N-succinylamino acid racemase 2	IX	Virgibacillus sp.	
W4F4J1	N-succinylamino acid racemase 2	IX	Viridibacillus arenosi	
B3ZAI2	N-succinylamino acid racemase 2	XI	Bacillus cereus	
C2W3E6	N-succinylamino acid racemase 2	XI	Bacillus cereus	
R8XQ22	N-succinylamino acid racemase 2	XI	Bacillus cereus	
G2TPI5	N-succinylamino acid racemase 2	XI	Bacillus coagulans	
D3FWL0	N-succinylamino acid racemase 2	XI	Bacillus pseudofirmus	
A3I4A2	N-succinylamino acid racemase 2	XI	Bacillus sp.	
A3I872	N-succinylamino acid racemase 2	XI	Bacillus sp.	
C4L1A8	N-succinylamino acid racemase 2	XI	Exiguobacterium sp.	
S3GFU4	N-succinylamino acid racemase 2	XI	Exiguobacterium sp.	
C3IQZ7	o-succinylbenzoate synthase	VI		
C7LY01	o-succinylbenzoate synthase	VI	Acidimicrobium ferrooxidans	4.2.1.113
C1F8P4	o-succinylbenzoate synthase	VI	Acidobacterium capsulatum	4.2.1.113
C8WS29	o-succinylbenzoate synthase	VI	Alicyclobacillus acidocaldarius	4.2.1.113
Q8VT69	o-succinylbenzoate synthase	VI	Amycolatopsis azurea	4.2.1.113
Q9FD06	o-succinylbenzoate synthase	VI	Amycolatopsis lurida	4.2.1.113
Q44244	o-succinylbenzoate synthase	VI	Amycolatopsis sp.	4.2.1.113
B7GJL3	o-succinylbenzoate synthase	VI	Anoxybacillus flavithermus	4.2.1.113
A0A023PBT5	o-succinylbenzoate synthase	VI	Bacillus bombysepticus	4.2.1.113
B3ZEB0	o-succinylbenzoate synthase	VI	Bacillus cereus	4.2.1.113
B5UP55	o-succinylbenzoate synthase	VI	Bacillus cereus	4.2.1.113
B9J2F1	o-succinylbenzoate synthase	VI	Bacillus cereus	4.2.1.113
C2MLM5	o-succinylbenzoate synthase	VI	Bacillus cereus	4.2.1.113
C2N887	o-succinylbenzoate synthase	VI	Bacillus cereus	4.2.1.113
C2PLZ8	o-succinylbenzoate synthase	VI	Bacillus cereus	4.2.1.113
C2QIY6	o-succinylbenzoate synthase	VI	Bacillus cereus	4.2.1.113
C2R000	o-succinylbenzoate synthase	VI	Bacillus cereus	4.2.1.113
C2REZ2	o-succinylbenzoate synthase	VI	Bacillus cereus	4.2.1.113
C2SAN0	o-succinylbenzoate synthase	VI	Bacillus cereus	4.2.1.113
C2T7P9	o-succinylbenzoate synthase	VI	Bacillus cereus	4.2.1.113
C2TNF6	o-succinylbenzoate synthase	VI	Bacillus cereus	4.2.1.113
C2V291	o-succinylbenzoate synthase	VI	Bacillus cereus	4.2.1.113
C2W0P6	o-succinylbenzoate synthase	VI	Bacillus cereus	4.2.1.113
C2WDM0	o-succinylbenzoate synthase	VI	Bacillus cereus	4.2.1.113
C2Y0X0	o-succinylbenzoate synthase	VI	Bacillus cereus	4.2.1.113
C2YY84	o-succinylbenzoate synthase	VI	Bacillus cereus	4.2.1.113
C2ZEH3	o-succinylbenzoate synthase	VI	Bacillus cereus	4.2.1.113
J8DSQ7	o-succinylbenzoate synthase	VI	Bacillus cereus	4.2.1.113
J9CYS8	o-succinylbenzoate synthase	VI	Bacillus cereus	4.2.1.113
Q632I6	o-succinylbenzoate synthase	VI	Bacillus cereus	4.2.1.113
Q72YL0	o-succinylbenzoate synthase	VI	Bacillus cereus	4.2.1.113
G2TN71	o-succinylbenzoate synthase	VI	Bacillus coagulans	4.2.1.113
A7GU87	o-succinylbenzoate synthase	VI	Bacillus cytotoxicus	4.2.1.113
U5LH01	o-succinylbenzoate synthase	VI	Bacillus infantis	4.2.1.113
T5HLU8	o-succinylbenzoate synthase	VI	Bacillus licheniformis	4.2.1.113
A7Z808	o-succinylbenzoate synthase	VI	Bacillus methylotrophicus	4.2.1.113
A0A0B5SB45	o-succinylbenzoate synthase	VI	Bacillus mycoides	4.2.1.113
C3AS80	o-succinylbenzoate synthase	VI	Bacillus mycoides	4.2.1.113
A8FGK5	o-succinylbenzoate synthase	VI	Bacillus pumilus	4.2.1.113
B4ANK1	o-succinylbenzoate synthase	VI	Bacillus pumilus	4.2.1.113
A3I6J7	o-succinylbenzoate synthase	VI	Bacillus sp.	4.2.1.113
A3IB71	o-succinylbenzoate synthase	VI	Bacillus sp.	4.2.1.113
A6CM80	o-succinylbenzoate synthase	VI	Bacillus sp.	4.2.1.113

Table S2.6. (continuation)

UniProt	Family [3]	Cluster	Species	EC Number
O34514	o-succinylbenzoate synthase	VI	Bacillus subtilis	4.2.1.113
A0A0D1NZ10	o-succinylbenzoate synthase	VI	Bacillus thuringiensis	4.2.1.113
A0A0F6JDV4	o-succinylbenzoate synthase	VI	Bacillus thuringiensis	4.2.1.113
C3C9M0	o-succinylbenzoate synthase	VI	Bacillus thuringiensis	4.2.1.113
C3DRZ4	o-succinylbenzoate synthase	VI	Bacillus thuringiensis	4.2.1.113
C3G9T9	o-succinylbenzoate synthase	VI	Bacillus thuringiensis	4.2.1.113
C3HQG0	o-succinylbenzoate synthase	VI	Bacillus thuringiensis	4.2.1.113
C3I7W3	o-succinylbenzoate synthase	VI	Bacillus thuringiensis	4.2.1.113
Q6HC30	o-succinylbenzoate synthase	VI	Bacillus thuringiensis	4.2.1.113
A9VM73	o-succinylbenzoate synthase	VI	Bacillus weihenstephanensis	4.2.1.113
W4DWF8	o-succinylbenzoate synthase	VI	Bacillus weihenstephanensis	4.2.1.113
C0ZHI1	o-succinylbenzoate synthase	VI	Brevibacillus brevis	4.2.1.113
C0ZIW6	o-succinylbenzoate synthase	VI	Brevibacillus brevis	4.2.1.113
C7Q4V3	o-succinylbenzoate synthase	VI	Catenulispora acidiphila	4.2.1.113
C7QI75	o-succinylbenzoate synthase	VI	Catenulispora acidiphila	4.2.1.113
B8G4N8	o-succinylbenzoate synthase	VI	Chloroflexus aggregans	4.2.1.113
A9WGZ9	o-succinylbenzoate synthase	VI	Chloroflexus aurantiacus	4.2.1.113
T2J1E8	o-succinylbenzoate synthase	VI	Crocosphaera watsonii	4.2.1.113
A3IXI5	o-succinylbenzoate synthase	VI	Cyanothece sp.	4.2.1.113
B1WTP2	o-succinylbenzoate synthase	VI	Cyanothece sp.	4.2.1.113
C1CZ82	o-succinylbenzoate synthase	VI	Deinococcus deserti	4.2.1.113
A0A098AY27	o-succinylbenzoate synthase	VI	Desulfitobacterium hafniense	4.2.1.113
B8FTZ2	o-succinylbenzoate synthase	VI	Desulfitobacterium hafniense	4.2.1.113
C6CDC6	o-succinylbenzoate synthase	VI	Dickeya dadantii	4.2.1.113
C9AC63	o-succinylbenzoate synthase	VI	Enterococcus casseliflavus	4.2.1.113
C9CNG8	o-succinylbenzoate synthase	VI	Enterococcus casseliflavus	4.2.1.113
C7CWW5	o-succinylbenzoate synthase	VI	Enterococcus faecalis	4.2.1.113
Q838J7	o-succinylbenzoate synthase	VI	Enterococcus faecalis	4.2.1.113
V7ZND7	o-succinylbenzoate synthase	VI	Enterococcus faecalis	4.2.1.113
T0VIJ0	o-succinylbenzoate synthase	VI	Enterococcus sp.	4.2.1.113
B1YK23	o-succinylbenzoate synthase	VI	Exiguobacterium sibiricum	4.2.1.113
C4L317	o-succinylbenzoate synthase	VI	Exiguobacterium sp.	4.2.1.113
C1A995	o-succinylbenzoate synthase	VI	Gemmatimonas aurantiaca	4.2.1.113
Q5L1G9	o-succinylbenzoate synthase	VI	Geobacillus kaustophilus	4.2.1.113
A0A0F6BQC9	o-succinylbenzoate synthase	VI	Geobacillus sp.	4.2.1.113
C5D7F0	o-succinylbenzoate synthase	VI	Geobacillus sp.	4.2.1.113
A4ILI9	o-succinylbenzoate synthase	VI	Geobacillus thermodenitrificans	4.2.1.113
A6CBP1	o-succinylbenzoate synthase	VI	Gimesia maris	4.2.1.113
D0L512	o-succinylbenzoate synthase	VI	Gordonia bronchialis	4.2.1.113
A3TM74	o-succinylbenzoate synthase	VI	Janibacter sp.	4.2.1.113
B1L4D6	o-succinylbenzoate synthase	VI	Korarchaeum cryptofilum	4.2.1.113
Q1IJW6	o-succinylbenzoate synthase	VI	Koribacter versatilis	4.2.1.113
Q1IPL2	o-succinylbenzoate synthase	VI	Koribacter versatilis	4.2.1.113
C5CFI0	o-succinylbenzoate synthase	VI	Kosmotoga olearia	4.2.1.113
D2Q1Y9	o-succinylbenzoate synthase	VI	Kribbella flavida	4.2.1.113
C8P8M1	o-succinylbenzoate synthase	VI	Lactobacillus antri	4.2.1.113
D0DRT1	o-succinylbenzoate synthase	VI	Lactobacillus fermentum	4.2.1.113
S5NRE7	o-succinylbenzoate synthase	VI	Lactobacillus reuteri	4.2.1.113
C7T9N5	o-succinylbenzoate synthase	VI	Lactobacillus rhamnosus	4.2.1.113
C2ETA2	o-succinylbenzoate synthase	VI	Lactobacillus vaginalis	4.2.1.113
A2RM77	o-succinylbenzoate synthase	VI	Lactococcus lactis	4.2.1.113
Q9CHK4	o-succinylbenzoate synthase	VI	Lactococcus lactis	4.2.1.113
T0RY68	o-succinylbenzoate synthase	VI	Lactococcus lactis	4.2.1.113
B1MZ25	o-succinylbenzoate synthase	VI	Leuconostoc citreum	4.2.1.113
C2KKL6	o-succinylbenzoate synthase	VI	Leuconostoc mesenteroides	4.2.1.113
Q03Y59	o-succinylbenzoate synthase	VI	Leuconostoc mesenteroides	4.2.1.113

Table S2.6. (continuation)

UniProt	Family [3]	Cluster	Species	EC Number
D7UVX9	o-succinylbenzoate synthase	VI	Listeria grayi	4.2.1.113
Q927X3	o-succinylbenzoate synthase	VI	Listeria innocua	4.2.1.113
A0A0E0UYM6	o-succinylbenzoate synthase	VI	Listeria monocytogenes	4.2.1.113
A0A0E0ZWW4	o-succinylbenzoate synthase	VI	Listeria monocytogenes	4.2.1.113
A0A0E1R8N8	o-succinylbenzoate synthase	VI	Listeria monocytogenes	4.2.1.113
Q8Y4D0	o-succinylbenzoate synthase	VI	Listeria monocytogenes	4.2.1.113
A0ALK4	o-succinylbenzoate synthase	VI	Listeria welshimeri	4.2.1.113
B1HYU9	o-succinylbenzoate synthase	VI	Lysinibacillus sphaericus	4.2.1.113
D3PSR8	o-succinylbenzoate synthase	VI	Meiothermus ruber	4.2.1.113
D7BG01	o-succinylbenzoate synthase	VI	Meiothermus silvanus	4.2.1.113
C8XAI1	o-succinylbenzoate synthase	VI	Nakamurella multipartita	4.2.1.113
A9EFP3	o-succinylbenzoate synthase	VI	Oceanibulbus indolifex	4.2.1.113
Q8EMX7	o-succinylbenzoate synthase	VI	Oceanobacillus iheyensis	4.2.1.113
C4WQW7	o-succinylbenzoate synthase	VI	Ochrobactrum intermedium	4.2.1.113
C6DIY8	o-succinylbenzoate synthase	VI	Pectobacterium carotovorum	4.2.1.113
D0KF53	o-succinylbenzoate synthase	VI	Pectobacterium wasabiae	4.2.1.113
A9WN93	o-succinylbenzoate synthase	VI	Renibacterium salmoninarum	4.2.1.113
C8S199	o-succinylbenzoate synthase	VI	Rhodobacter sp.	4.2.1.113
B6B5Q1	o-succinylbenzoate synthase	VI	Rhodobacterales bacterium	4.2.1.113
C0ZPK2	o-succinylbenzoate synthase	VI	Rhodococcus erythropolis	4.2.1.113
C0ZRF4	o-succinylbenzoate synthase	VI	Rhodococcus erythropolis	4.2.1.113
C3JF10	o-succinylbenzoate synthase	VI	Rhodococcus erythropolis	4.2.1.113
C3JVB8	o-succinylbenzoate synthase	VI	Rhodococcus erythropolis	4.2.1.113
A7NLX0	o-succinylbenzoate synthase	VI	Roseiflexus castenholzii	4.2.1.113
A5UX74	o-succinylbenzoate synthase	VI	Roseiflexus sp.	4.2.1.113
Q027H6	o-succinylbenzoate synthase	VI	Solibacter usitatus	4.2.1.113
D3Q2N8	o-succinylbenzoate synthase	VI	Stackebrandtia nassauensis	4.2.1.113
A3DMP1	o-succinylbenzoate synthase	VI	Staphylothermus marinus	4.2.1.113
D9WUW7	o-succinylbenzoate synthase	VI	Streptomyces himastatinicus	4.2.1.113
D9UYN8	o-succinylbenzoate synthase	VI	Streptomyces sp.	4.2.1.113
D9VDR7	o-succinylbenzoate synthase	VI	Streptomyces sp.	4.2.1.113
D2ARB2	o-succinylbenzoate synthase	VI	Streptosporangium roseum	4.2.1.113
D2B5M4	o-succinylbenzoate synthase	VI	Streptosporangium roseum	4.2.1.113
D2BC18	o-succinylbenzoate synthase	VI	Streptosporangium roseum	4.2.1.113
D1CFN4	o-succinylbenzoate synthase	VI	Thermobaculum terrenum	4.2.1.113
A1RWU3	o-succinylbenzoate synthase	VI	Thermofilum pendens	4.2.1.113
B7A7E3	o-succinylbenzoate synthase	VI	Thermus aquaticus	4.2.1.113
C4FSN4	o-succinylbenzoate synthase	VI	Veillonella dispar	4.2.1.113
R5BIZ3	o-succinylbenzoate synthase	VI	Veillonella sp.	4.2.1.113
W1UPJ7	o-succinylbenzoate synthase	VI	Veillonella sp.	4.2.1.113
C5R8G9	o-succinylbenzoate synthase	VI	Weissella paramesenteroides	4.2.1.113
A0A011PTB3	o-succinylbenzoate synthase	IX	Candidatus Accumulibacter	4.2.1.113
A0A011MYG4	o-succinylbenzoate synthase	IX	Candidatus Accumulibacter	5.1.1.-
D0D090	o-succinylbenzoate synthase	IX	Citreicella sp.	
D2SB85	o-succinylbenzoate synthase	IX	Geodermatophilus obscurus	
D0LZJ1	o-succinylbenzoate synthase	IX	Haliangium ochraceum	
M0DZP8	o-succinylbenzoate synthase	IX	Halorubrum tebenquichense	4.2.1.113
B9E714	o-succinylbenzoate synthase	IX	Macrococcus caseolyticus	
F7YD12	o-succinylbenzoate synthase	IX	Mesorhizobium opportunistum	
M0BLH1	o-succinylbenzoate synthase	IX	Natrialba aegyptia	4.2.1.113
L9Y8N7	o-succinylbenzoate synthase	IX	Natrinema versiforme	4.2.1.113
L0AE97	o-succinylbenzoate synthase	IX	Natronobacterium gregoryi	4.2.1.113
L9W3V1	o-succinylbenzoate synthase	IX	Natronorubrum bangense	4.2.1.113
L9VZB1	o-succinylbenzoate synthase	IX	Natronorubrum sulfidifaciens	4.2.1.113
E6WN49	o-succinylbenzoate synthase	IX	Pantoea sp.	
B6A5G0	o-succinylbenzoate synthase	IX	Rhizobium leguminosarum	

Table S2.6. (continuation)

UniProt	Family [3]	Cluster	Species	EC Number
S9QKQ4	o-succinylbenzoate synthase	IX	Salipiger mucosus	
D2QBV0	o-succinylbenzoate synthase	IX	Spirosoma linguale	
L0H1T4	o-succinylbenzoate synthase	IX	Thioflavicoccus mobilis	4.2.1.113
W9VYU8	o-succinylbenzoate synthase	IX	Thiorhodococcus sp.	4.2.1.113
C7RVE0	o-succinylbenzoate synthase	XI	Accumulibacter phosphatis	
A3I1S9	o-succinylbenzoate synthase	XI	Algoriphagus machipongonensis	4.2.1.113
D3RPJ0	o-succinylbenzoate synthase	XI	Allochromatium vinosum	4.2.1.113
A0A074L2V7	o-succinylbenzoate synthase	XI	Anditalea andensis	4.2.1.113
R6T6P2	o-succinylbenzoate synthase	XI	Bacteroides coprophilus	4.2.1.113
A0A076IL77	o-succinylbenzoate synthase	XI	Bacteroides dorei	4.2.1.113
B6W3I7	o-succinylbenzoate synthase	XI	Bacteroides dorei	4.2.1.113
C3R6B8	o-succinylbenzoate synthase	XI	Bacteroides dorei	4.2.1.113
I9RDU8	o-succinylbenzoate synthase	XI	Bacteroides dorei	4.2.1.113
F3PWC9	o-succinylbenzoate synthase	XI	Bacteroides fluxus	4.2.1.113
B5CZ30	o-succinylbenzoate synthase	XI	Bacteroides plebeius	4.2.1.113
R5N7H5	o-succinylbenzoate synthase	XI	Bacteroides sp.	4.2.1.113
R5U0C3	o-succinylbenzoate synthase	XI	Bacteroides sp.	4.2.1.113
R6YQE5	o-succinylbenzoate synthase	XI	Bacteroides sp.	4.2.1.113
R7CZK4	o-succinylbenzoate synthase	XI	Bacteroides sp.	4.2.1.113
R7NQF2	o-succinylbenzoate synthase	XI	Bacteroides sp.	4.2.1.113
A0A069SLL0	o-succinylbenzoate synthase	XI	Bacteroides vulgatus	4.2.1.113
I3Z8Q5	o-succinylbenzoate synthase	XI	Belliella baltica	4.2.1.113
D0J9F9	o-succinylbenzoate synthase	XI	Blattabacterium sp.	
G7SQ37	o-succinylbenzoate synthase	XI	Blattabacterium sp.	
L7YCT5	o-succinylbenzoate synthase	XI	Blattabacterium sp.	
U3QD04	o-succinylbenzoate synthase	XI	Blattabacterium sp.	
D0JAV3	o-succinylbenzoate synthase	XI	Blattabacterium sp.	5.5.1.7
K9ADV1	o-succinylbenzoate synthase	XI	Brevibacterium casei	4.2.1.113
B4EGJ5	o-succinylbenzoate synthase	XI	Burkholderia cenocepacia	
I0I4Y5	o-succinylbenzoate synthase	XI	Caldilinea aerophila	4.2.1.113
A0A011RFS2	o-succinylbenzoate synthase	XI	Candidatus Accumulibacter	5.1.1.-
A0A0A6PHP3	o-succinylbenzoate synthase	XI	Candidatus Thiomargarita	4.2.1.113
F9YVB0	o-succinylbenzoate synthase	XI	Capnocytophaga canimorsus	4.2.1.113
B3QKZ2	o-succinylbenzoate synthase	XI	Chlorobaculum parvum	4.2.1.113
Q3APV6	o-succinylbenzoate synthase	XI	Chlorobium chlorochromatii	4.2.1.113
Q0YUC8	o-succinylbenzoate synthase	XI	Chlorobium ferrooxidans	4.2.1.113
B3EG71	o-succinylbenzoate synthase	XI	Chlorobium limicola	4.2.1.113
Q3B616	o-succinylbenzoate synthase	XI	Chlorobium luteolum	4.2.1.113
A1BI77	o-succinylbenzoate synthase	XI	Chlorobium phaeobacteroides	4.2.1.113
B3EMZ8	o-succinylbenzoate synthase	XI	Chlorobium phaeobacteroides	4.2.1.113
A4SD56	o-succinylbenzoate synthase	XI	Chlorobium phaeovibrioides	4.2.1.113
Q8KBE1	o-succinylbenzoate synthase	XI	Chlorobium tepidum	4.2.1.113
B3QUT9	o-succinylbenzoate synthase	XI	Chloroherpeton thalassium	4.2.1.113
A0A017TFA9	o-succinylbenzoate synthase	XI	Chondromyces apiculatus	4.2.1.113
H8MGN5	o-succinylbenzoate synthase	XI	Corallococcus coralloides	4.2.1.113
W5WTP6	o-succinylbenzoate synthase	XI	Corynebacterium falsenii	4.2.1.113
Q4JT08	o-succinylbenzoate synthase	XI	Corynebacterium jeikeium	4.2.1.113
F8E2Q2	o-succinylbenzoate synthase	XI	Corynebacterium resistens	4.2.1.113
B1VEM6	o-succinylbenzoate synthase	XI	Corynebacterium urealyticum	4.2.1.113
Q9TM12	o-succinylbenzoate synthase	XI	Cyanidium caldarium	
R7ZR53	o-succinylbenzoate synthase	XI	Cyclobacteriaceae bacterium	4.2.1.113
G0J7D0	o-succinylbenzoate synthase	XI	Cyclobacterium marinum	4.2.1.113
S7VCW5	o-succinylbenzoate synthase	XI	Cyclobacterium qasimii	4.2.1.113
S9Q4K2	o-succinylbenzoate synthase	XI	Cystobacter fuscus	
Q11SE3	o-succinylbenzoate synthase	XI	Cytophaga hutchinsonii	4.2.1.113
G8QGD3	o-succinylbenzoate synthase	XI	Dechlorosoma suillum	4.2.1.113

Table S2.6. (continuation)

UniProt	Family [3]	Cluster	Species	EC Number
C1D1D5	o-succinylbenzoate synthase	XI	Deinococcus deserti	
B8FA12	o-succinylbenzoate synthase	XI	Desulfatibacillum alkenivorans	4.2.1.113
Q6ARP5	o-succinylbenzoate synthase	XI	Desulfotalea psychrophila	4.2.1.113
C6W0I2	o-succinylbenzoate synthase	XI	Dyadobacter fermentans	4.2.1.113
F5IT38	o-succinylbenzoate synthase	XI	Dysgonomonas gadei	4.2.1.113
L0G161	o-succinylbenzoate synthase	XI	Echinicola vietnamensis	4.2.1.113
I2EWP8	o-succinylbenzoate synthase	XI	Emticicia oligotrophica	4.2.1.113
G2DA36	o-succinylbenzoate synthase	XI	endosymbiont of	4.2.1.113
G2FC95	o-succinylbenzoate synthase	XI	endosymbiont of	4.2.1.113
I0KB13	o-succinylbenzoate synthase	XI	Fibrella aestuarina	4.2.1.113
I2GJ52	o-succinylbenzoate synthase	XI	Fibrisoma limi	4.2.1.113
C0BNQ8	o-succinylbenzoate synthase	XI	Flavobacteria bacterium	4.2.1.113
G2Z7D2	o-succinylbenzoate synthase	XI	Flavobacterium branchiophilum	4.2.1.113
H7FWE2	o-succinylbenzoate synthase	XI	Flavobacterium frigoris	4.2.1.113
A5FG57	o-succinylbenzoate synthase	XI	Flavobacterium johnsoniae	4.2.1.113
A0A085EGL6	o-succinylbenzoate synthase	XI	Flavobacterium sp.	4.2.1.113
L8JUE9	o-succinylbenzoate synthase	XI	Fulvivirga imtechensis	4.2.1.113
A6BZA8	o-succinylbenzoate synthase	XI	Gimesia maris	
D0L557	o-succinylbenzoate synthase	XI	Gordonia bronchialis	
D0LH51	o-succinylbenzoate synthase	XI	Haliangium ochraceum	
M0LCL8	o-succinylbenzoate synthase	XI	Halobiforma lacisalsi	4.2.1.113
M0LZ40	o-succinylbenzoate synthase	XI	Halobiforma nitratireducens	4.2.1.113
M0ICW5	o-succinylbenzoate synthase	XI	Haloferax alexandrinus	4.2.1.113
M0GQW4	o-succinylbenzoate synthase	XI	Haloferax lucentense	4.2.1.113
M0FNR1	o-succinylbenzoate synthase	XI	Haloferax sp.	4.2.1.113
M0G065	o-succinylbenzoate synthase	XI	Haloferax sp.	4.2.1.113
M0IQN9	o-succinylbenzoate synthase	XI	Haloferax sulfurifontis	4.2.1.113
D4GY84	o-succinylbenzoate synthase	XI	Haloferax volcanii	4.2.1.113
L9UU79	o-succinylbenzoate synthase	XI	Haloferax volcanii	4.2.1.113
J3JHV2	o-succinylbenzoate synthase	XI	Halogranum salarium	4.2.1.113
U1PT83	o-succinylbenzoate synthase	XI	halophilic archaeon	4.2.1.113
G0LKG8	o-succinylbenzoate synthase	XI	Haloquadratum walsbyi	4.2.1.113
Q18J08	o-succinylbenzoate synthase	XI	Haloquadratum walsbyi	4.2.1.113
U1NID4	o-succinylbenzoate synthase	XI	Haloquadratum walsbyi	4.2.1.113
M0PIU9	o-succinylbenzoate synthase	XI	Halorubrum aidingense	4.2.1.113
M0PPU5	o-succinylbenzoate synthase	XI	Halorubrum arcis	4.2.1.113
M0E0Y1	o-succinylbenzoate synthase	XI	Halorubrum californiensis	4.2.1.113
M0EJL5	o-succinylbenzoate synthase	XI	Halorubrum coriense	4.2.1.113
M0F2C6	o-succinylbenzoate synthase	XI	Halorubrum hochstenium	4.2.1.113
M0NG44	o-succinylbenzoate synthase	XI	Halorubrum kocurii	4.2.1.113
B9LN30	o-succinylbenzoate synthase	XI	Halorubrum lacusprofundi	4.2.1.113
M0P1D5	o-succinylbenzoate synthase	XI	Halorubrum lipolyticum	4.2.1.113
M0P3U4	o-succinylbenzoate synthase	XI	Halorubrum litoreum	4.2.1.113
M0DNG3	o-succinylbenzoate synthase	XI	Halorubrum saccharovorum	4.2.1.113
U1QFM9	o-succinylbenzoate synthase	XI	Halorubrum sp.	4.2.1.113
M0DMF5	o-succinylbenzoate synthase	XI	Halorubrum terrestre	4.2.1.113
M0BZN2	o-succinylbenzoate synthase	XI	Haloterrigena salina	4.2.1.113
M0BZZ8	o-succinylbenzoate synthase	XI	Haloterrigena thermotolerans	4.2.1.113
D2RS79	o-succinylbenzoate synthase	XI	Haloterrigena turkmenica	4.2.1.113
A0A085WRP0	o-succinylbenzoate synthase	XI	Hyalangium minutum	
A0A076HXB7	o-succinylbenzoate synthase	XI	Hymenobacter sp.	4.2.1.113
W8F0Q5	o-succinylbenzoate synthase	XI	Hymenobacter swuensis	4.2.1.113
A8ABR4	o-succinylbenzoate synthase	XI	Ignicoccus hospitalis	
S2DMN1	o-succinylbenzoate synthase	XI	Indibacter alkaliphilus	4.2.1.113
A9DU98	o-succinylbenzoate synthase	XI	Kordia algicida	4.2.1.113
D6TM01	o-succinylbenzoate synthase	XI	Ktedonobacter racemifer	4.2.1.113

Table S2.6. (continuation)

UniProt	Family [3]	Cluster	Species	EC Number
E4RQ11	o-succinylbenzoate synthase	XI	Leadbetterella byssophila	4.2.1.113
W0E0M5	o-succinylbenzoate synthase	XI	Marichromatium purpuratum	4.2.1.113
M7XK74	o-succinylbenzoate synthase	XI	Mariniradius saccharolyticus	4.2.1.113
E4TRG7	o-succinylbenzoate synthase	XI	Marivirga tractuosa	4.2.1.113
A1ZMA5	o-succinylbenzoate synthase	XI	Microscilla marina	4.2.1.113
A6FHU6	o-succinylbenzoate synthase	XI	Moritella sp.	4.2.1.113
A0A090IF12	o-succinylbenzoate synthase	XI	Moritella viscosa	4.2.1.113
F8C9B8	o-succinylbenzoate synthase	XI	Myxococcus fulvus	4.2.1.113
L7UDQ1	o-succinylbenzoate synthase	XI	Myxococcus stipitatus	4.2.1.113
Q1D6J8	o-succinylbenzoate synthase	XI	Myxococcus xanthus	4.2.1.113
B2A420	o-succinylbenzoate synthase	XI	Natranaerobius thermophilus	4.2.1.113
M0ARI5	o-succinylbenzoate synthase	XI	Natrialba asiatica	4.2.1.113
M0AKM0	o-succinylbenzoate synthase	XI	Natrialba chahannaoensis	4.2.1.113
L9ZJ92	o-succinylbenzoate synthase	XI	Natrialba hulunbeirensis	4.2.1.113
D3SU17	o-succinylbenzoate synthase	XI	Natrialba magadii	4.2.1.113
L9ZIX3	o-succinylbenzoate synthase	XI	Natrialba taiwanensis	4.2.1.113
L9ZQA8	o-succinylbenzoate synthase	XI	Natrinema altunense	4.2.1.113
L9YPV0	o-succinylbenzoate synthase	XI	Natrinema gari	4.2.1.113
L9YZB8	o-succinylbenzoate synthase	XI	Natrinema pallidum	4.2.1.113
L0JNJ5	o-succinylbenzoate synthase	XI	Natrinema pellirubrum	4.2.1.113
I7BZ55	o-succinylbenzoate synthase	XI	Natrinema sp.	4.2.1.113
L9XNP8	o-succinylbenzoate synthase	XI	Natronolimnobius innermongolicus	4.2.1.113
L9VMH5	o-succinylbenzoate synthase	XI	Natronorubrum tibetense	4.2.1.113
I5C9Q6	o-succinylbenzoate synthase	XI	Nitritalea halalkaliphila	4.2.1.113
B1ZW04	o-succinylbenzoate synthase	XI	Opitutus terrae	4.2.1.113
K9VQ98	o-succinylbenzoate synthase	XI	Oscillatoria nigro-viridis	4.2.1.113
B4SEU6	o-succinylbenzoate synthase	XI	Pelodictyon phaeoclathratiforme	4.2.1.113
J1FGN2	o-succinylbenzoate synthase	XI	Pontibacter sp.	4.2.1.113
F4KKE4	o-succinylbenzoate synthase	XI	Porphyromonas asaccharolytica	4.2.1.113
F5X9G1	o-succinylbenzoate synthase	XI	Porphyromonas gingivalis	4.2.1.113
Q7MUJ1	o-succinylbenzoate synthase	XI	Porphyromonas gingivalis	4.2.1.113
U2K8T4	o-succinylbenzoate synthase	XI	Porphyromonas gingivalis	4.2.1.113
W1R628	o-succinylbenzoate synthase	XI	Porphyromonas gingivalis	4.2.1.113
A0A0A2FXY0	o-succinylbenzoate synthase	XI	Porphyromonas gulae	4.2.1.113
C2MBE7	o-succinylbenzoate synthase	XI	Porphyromonas uenonis	4.2.1.113
A0A095ZEH0	o-succinylbenzoate synthase	XI	Prevotella buccalis	4.2.1.113
D1W650	o-succinylbenzoate synthase	XI	Prevotella buccalis	4.2.1.113
D1PBJ6	o-succinylbenzoate synthase	XI	Prevotella copri	4.2.1.113
R6C433	o-succinylbenzoate synthase	XI	Prevotella copri	4.2.1.113
A0A096AT54	o-succinylbenzoate synthase	XI	Prevotella disiens	4.2.1.113
E1KRQ7	o-succinylbenzoate synthase	XI	Prevotella disiens	4.2.1.113
U2J4N4	o-succinylbenzoate synthase	XI	Prevotella disiens	4.2.1.113
F0F9P7	o-succinylbenzoate synthase	XI	Prevotella multiformis	4.2.1.113
R5CJ55	o-succinylbenzoate synthase	XI	Prevotella sp.	4.2.1.113
R5FND5	o-succinylbenzoate synthase	XI	Prevotella sp.	4.2.1.113
R6BXE4	o-succinylbenzoate synthase	XI	Prevotella sp.	4.2.1.113
R6Q0P7	o-succinylbenzoate synthase	XI	Prevotella sp.	4.2.1.113
R6YGX2	o-succinylbenzoate synthase	XI	Prevotella sp.	4.2.1.113
R7EYU7	o-succinylbenzoate synthase	XI	Prevotella sp.	4.2.1.113
R7J094	o-succinylbenzoate synthase	XI	Prevotella sp.	4.2.1.113
D1W1B5	o-succinylbenzoate synthase	XI	Prevotella timonensis	4.2.1.113
B4S4J7	o-succinylbenzoate synthase	XI	Prosthecochloris aestuarii	4.2.1.113
K4IKC4	o-succinylbenzoate synthase	XI	Psychroflexus torquis	4.2.1.113
U5BV71	o-succinylbenzoate synthase	XI	Rhodonellum psychrophilum	4.2.1.113
D0MFG0	o-succinylbenzoate synthase	XI	Rhodothermus marinus	4.2.1.113
G2SEP3	o-succinylbenzoate synthase	XI	Rhodothermus marinus	4.2.1.113

Table S2.6. (continuation)

UniProt	Family [3]	Cluster	Species	EC Number
A3SNF7	o-succinylbenzoate synthase	XI	Roseovarius nubinhibens	
F8EHR6	o-succinylbenzoate synthase	XI	Runella slithyformis	4.2.1.113
D5H8W4	o-succinylbenzoate synthase	XI	Salinibacter ruber	4.2.1.113
Q2S2V3	o-succinylbenzoate synthase	XI	Salinibacter ruber	4.2.1.113
A1SBA8	o-succinylbenzoate synthase	XI	Shewanella amazonensis	4.2.1.113
A3CYZ7	o-succinylbenzoate synthase	XI	Shewanella baltica	4.2.1.113
A9KU67	o-succinylbenzoate synthase	XI	Shewanella baltica	4.2.1.113
B8E3U9	o-succinylbenzoate synthase	XI	Shewanella baltica	4.2.1.113
G0AUK2	o-succinylbenzoate synthase	XI	Shewanella baltica	4.2.1.113
A9CXZ2	o-succinylbenzoate synthase	XI	Shewanella benthica	4.2.1.113
Q089C2	o-succinylbenzoate synthase	XI	Shewanella frigidimarina	4.2.1.113
A3QJ19	o-succinylbenzoate synthase	XI	Shewanella loihica	4.2.1.113
Q8E8T2	o-succinylbenzoate synthase	XI	Shewanella oneidensis	4.2.1.113
A4Y257	o-succinylbenzoate synthase	XI	Shewanella putrefaciens	4.2.1.113
E6XIL4	o-succinylbenzoate synthase	XI	Shewanella putrefaciens	4.2.1.113
D4ZEF9	o-succinylbenzoate synthase	XI	Shewanella violacea	4.2.1.113
D1C9C7	o-succinylbenzoate synthase	XI	Sphaerobacter thermophilus	4.2.1.113
D2QCR0	o-succinylbenzoate synthase	XI	Spirosoma linguale	4.2.1.113
E3FMQ3	o-succinylbenzoate synthase	XI	Stigmatella aurantiaca	
D6A8F1	o-succinylbenzoate synthase	XI	Streptomyces ghanaensis	
D9WC22	o-succinylbenzoate synthase	XI	Streptomyces himastatinicus	
D9WS40	o-succinylbenzoate synthase	XI	Streptomyces himastatinicus	
A3YUG2	o-succinylbenzoate synthase	XI	Synechococcus sp.	
K9SY90	o-succinylbenzoate synthase	XI	Synechococcus sp.	
Q2LX98	o-succinylbenzoate synthase	XI	Syntrophus aciditrophicus	4.2.1.113
G8UI36	o-succinylbenzoate synthase	XI	Tannerella forsythia	4.2.1.113
R5IPQ1	o-succinylbenzoate synthase	XI	Tannerella sp.	4.2.1.113
C5BIZ3	o-succinylbenzoate synthase	XI	Teredinibacter turnerae	
D1CGG4	o-succinylbenzoate synthase	XI	Thermobaculum terrenum	
B9L3E2	o-succinylbenzoate synthase	XI	Thermomicrobium roseum	4.2.1.113
L0DWC3	o-succinylbenzoate synthase	XI	Thioalkalivibrio nitratireducens	4.2.1.113
F9U6L3	o-succinylbenzoate synthase	XI	Thiocapsa marina	4.2.1.113
I3YBK8	o-succinylbenzoate synthase	XI	Thiocystis violascens	4.2.1.113
W0TL64	o-succinylbenzoate synthase	XI	Thiolapillus brandeum	4.2.1.113
G4E517	o-succinylbenzoate synthase	XI	Thiorhodospira sibirica	4.2.1.113
H8YWU8	o-succinylbenzoate synthase	XI	Thiorhodovibrio sp.	4.2.1.113
V4YCM3	o-succinylbenzoate synthase	XI	uncultured archaeon	4.2.1.113
C5D0C9	o-succinylbenzoate synthase	XI	Variovorax paradoxus	
F0P1Q4	o-succinylbenzoate synthase	XI	Weeksella virosa	4.2.1.113
L8LXT9	o-succinylbenzoate synthase	XI	Xenococcus sp.	4.2.1.113
U2I1E6	o-succinylbenzoate synthase	XII	Corynebacterium pseudodiphtheriticum	4.2.1.113
Q28SI7	o-succinylbenzoate synthase	XII	Jannaschia sp.	5.5.1.1
A6W5P3	o-succinylbenzoate synthase	XII	Kineococcus radiotolerans	4.2.1.113
A0A010Q5I0	o-succinylbenzoate synthase	XII	Nesterenkonia sp.	4.2.1.113
B9NVY5	o-succinylbenzoate synthase	XII	Rhodobacteraceae bacterium	5.5.1.1
Q5LM96	o-succinylbenzoate synthase	XII	Ruegeria pomeroyi	5.5.1.1
C5D0Z8	o-succinylbenzoate synthase	XII	Variovorax paradoxus	
A0A027YIL9	rhamnonate dehydratase	V		
A0A094NSZ0	rhamnonate dehydratase	V		
W4NBU9	rhamnonate dehydratase	V		
L8HKA8	rhamnonate dehydratase	V	Acanthamoeba castellanii	
N8X8V5	rhamnonate dehydratase	V	Acinetobacter bereziniae	4.2.1.90
M2TRK1	rhamnonate dehydratase	V	alpha proteobacterium	4.2.1.90
A0A0F3KDW9	rhamnonate dehydratase	V	Aquitalea magnusonii	4.2.1.90
D5V3H1	rhamnonate dehydratase	V	Arcobacter nitrofigilis	4.2.1.90
H0QTN6	rhamnonate dehydratase	V	Arthrobacter globiformis	4.2.1.90

Table S2.6. (continuation)

UniProt	Family [3]	Cluster	Species	EC Number
E8RLX5	rhamnonate dehydratase	V	Asticcacaulis excentricus	4.2.1.90
A8I5F1	rhamnonate dehydratase	V	Azorhizobium caulinodans	4.2.1.90
C1DMY1	rhamnonate dehydratase	V	Azotobacter vinelandii	4.2.1.90
A0A086ZN78	rhamnonate dehydratase	V	Bifidobacterium biavatii	4.2.1.90
A5ZUR5	rhamnonate dehydratase	V	Blautia obeum	4.2.1.90
A0A069ITA3	rhamnonate dehydratase	V	Brevundimonas sp.	4.2.1.90
B4WBZ7	rhamnonate dehydratase	V	Brevundimonas sp.	4.2.1.90
D9QN20	rhamnonate dehydratase	V	Brevundimonas subvibrioides	4.2.1.90
B1FK31	rhamnonate dehydratase	V	Burkholderia ambifaria	4.2.1.90
B1YMY4	rhamnonate dehydratase	V	Burkholderia ambifaria	4.2.1.90
Q0BA70	rhamnonate dehydratase	V	Burkholderia ambifaria	4.2.1.90
A0A071M197	rhamnonate dehydratase	V	Burkholderia cenocepacia	4.2.1.90
B1KAV6	rhamnonate dehydratase	V	Burkholderia cenocepacia	4.2.1.90
A0A095GZL2	rhamnonate dehydratase	V	Burkholderia cepacia	4.2.1.90
A0A069PB87	rhamnonate dehydratase	V	Burkholderia glathei	4.2.1.90
B1FSL3	rhamnonate dehydratase	V	Burkholderia graminis	4.2.1.90
A0A069NHV2	rhamnonate dehydratase	V	Burkholderia grimmiae	4.2.1.90
A0A038GQL9	rhamnonate dehydratase	V	Burkholderia jiangsuensis	4.2.1.90
B2JLI8	rhamnonate dehydratase	V	Burkholderia phymatum	4.2.1.90
B2TFM7	rhamnonate dehydratase	V	Burkholderia phytofirmans	4.2.1.90
A0A087NLJ2	rhamnonate dehydratase	V	Burkholderia pyrrocinia	4.2.1.90
A0A060P4N5	rhamnonate dehydratase	V	Burkholderia sp.	4.2.1.90
B5WBA9	rhamnonate dehydratase	V	Burkholderia sp.	4.2.1.90
D5WID4	rhamnonate dehydratase	V	Burkholderia sp.	4.2.1.90
E1TFD0	rhamnonate dehydratase	V	Burkholderia sp.	4.2.1.90
E8YRQ6	rhamnonate dehydratase	V	Burkholderia sp.	4.2.1.90
I2IUM5	rhamnonate dehydratase	V	Burkholderia sp.	4.2.1.90
K8RA45	rhamnonate dehydratase	V	Burkholderia sp.	4.2.1.90
W6X884	rhamnonate dehydratase	V	Burkholderia sp.	4.2.1.90
Q13LW5	rhamnonate dehydratase	V	Burkholderia xenovorans	4.2.1.90
A8MA91	rhamnonate dehydratase	V	Caldivirga maquilingensis	4.2.1.90
R7TCS0	rhamnonate dehydratase	V	Capitella teleta	
D5VGC7	rhamnonate dehydratase	V	Caulobacter segnis	4.2.1.90
D5VL18	rhamnonate dehydratase	V	Caulobacter segnis	4.2.1.90
A0A083Z6B4	rhamnonate dehydratase	V	Citrobacter amalonaticus	4.2.1.90
D2TGZ8	rhamnonate dehydratase	V	Citrobacter rodentium	4.2.1.90
A0A064D0X6	rhamnonate dehydratase	V	Citrobacter sp.	4.2.1.90
F0YV43	rhamnonate dehydratase	V	Clostridium sp.	
A0A080P0S3	rhamnonate dehydratase	V	Delftia acidovorans	4.2.1.90
A9BQY2	rhamnonate dehydratase	V	Delftia acidovorans	4.2.1.90
A0A072T2W9	rhamnonate dehydratase	V	Delftia tsuruhatensis	4.2.1.90
B1EJN5	rhamnonate dehydratase	V	Escherichia albertii	4.2.1.90
W0AVH9	rhamnonate dehydratase	V	Escherichia albertii	4.2.1.90
A0A079YC28	rhamnonate dehydratase	V	Escherichia coli	4.2.1.90
A0A0B1G4Z0	rhamnonate dehydratase	V	Escherichia coli	4.2.1.90
A8A2B3	rhamnonate dehydratase	V	Escherichia coli	4.2.1.90
B1LLK1	rhamnonate dehydratase	V	Escherichia coli	4.2.1.90
B7MG14	rhamnonate dehydratase	V	Escherichia coli	4.2.1.90
B7MXS8	rhamnonate dehydratase	V	Escherichia coli	4.2.1.90
B7UFQ9	rhamnonate dehydratase	V	Escherichia coli	4.2.1.90
E9YBF6	rhamnonate dehydratase	V	Escherichia coli	4.2.1.90
P77215	rhamnonate dehydratase	V	Escherichia coli	4.2.1.90
Q0TFJ5	rhamnonate dehydratase	V	Escherichia coli	4.2.1.90
Q8XE07	rhamnonate dehydratase	V	Escherichia coli	4.2.1.90
T5P868	rhamnonate dehydratase	V	Escherichia coli	4.2.1.90
V0ZV42	rhamnonate dehydratase	V	Escherichia coli	4.2.1.90

Table S2.6. (continuation)

UniProt	Family [3]	Cluster	Species	EC Number
X7P5N7	rhamnonate dehydratase	V	Escherichia coli	4.2.1.90
D5RB60	rhamnonate dehydratase	V	Fusobacterium nucleatum	4.2.1.90
A0A080KIC0	rhamnonate dehydratase	V	Gilliamella apicola	4.2.1.90
W1N5R3	rhamnonate dehydratase	V	Halomonas huangheensis	4.2.1.90
C6XQD0	rhamnonate dehydratase	V	Hirschia baltica	4.2.1.90
A0A068HFG8	rhamnonate dehydratase	V	Klebsiella oxytoca	4.2.1.90
A0A0E0X2Y5	rhamnonate dehydratase	V	Klebsiella oxytoca	4.2.1.90
H3N0B6	rhamnonate dehydratase	V	Klebsiella oxytoca	4.2.1.90
A0A0C7K8C6	rhamnonate dehydratase	V	Klebsiella pneumoniae	4.2.1.90
A6TBU8	rhamnonate dehydratase	V	Klebsiella pneumoniae	4.2.1.90
B5XNY1	rhamnonate dehydratase	V	Klebsiella pneumoniae	4.2.1.90
J2LQ38	rhamnonate dehydratase	V	Klebsiella pneumoniae	4.2.1.90
W8XR55	rhamnonate dehydratase	V	Klebsiella quasipneumoniae	4.2.1.90
J5UTJ9	rhamnonate dehydratase	V	Klebsiella sp.	4.2.1.90
A0A085HJ16	rhamnonate dehydratase	V	Leminorella grimontii	4.2.1.90
A0A081G3C5	rhamnonate dehydratase	V	Marinobacterium sp.	4.2.1.90
B8IEQ0	rhamnonate dehydratase	V	Methylobacterium nodulans	4.2.1.90
T5KB16	rhamnonate dehydratase	V	Microbacterium maritypicum	4.2.1.90
F5XJ21	rhamnonate dehydratase	V	Microlunatus phosphovorus	4.2.1.90
J3DBV1	rhamnonate dehydratase	V	Pantoea sp.	4.2.1.90
Q12DF1	rhamnonate dehydratase	V	Polaromonas sp.	4.2.1.90
W8PSP6	rhamnonate dehydratase	V	Pseudomonas brassicacearum	4.2.1.90
A0A075P3C9	rhamnonate dehydratase	V	Pseudomonas fluorescens	4.2.1.90
A0A0A1Z3C6	rhamnonate dehydratase	V	Pseudomonas fluorescens	4.2.1.90
H0J728	rhamnonate dehydratase	V	Pseudomonas psychrotolerans	4.2.1.90
A0A031IQ69	rhamnonate dehydratase	V	Pseudomonas sp.	4.2.1.90
A0A077LL20	rhamnonate dehydratase	V	Pseudomonas sp.	4.2.1.90
I4N592	rhamnonate dehydratase	V	Pseudomonas sp.	4.2.1.90
J0PM02	rhamnonate dehydratase	V	Pseudomonas sp.	4.2.1.90
J3IKT8	rhamnonate dehydratase	V	Pseudomonas sp.	4.2.1.90
W2DEV5	rhamnonate dehydratase	V	Pseudomonas sp.	4.2.1.90
W2DG94	rhamnonate dehydratase	V	Pseudomonas sp.	4.2.1.90
A4FLW9	rhamnonate dehydratase	V	Saccharopolyspora erythraea	4.2.1.90
A0A084IK24	rhamnonate dehydratase	V	Salinisphaera hydrothermalis	4.2.1.90
Q57M62	rhamnonate dehydratase	V	Salmonella choleraesuis	4.2.1.90
G5LRB2	rhamnonate dehydratase	V	Salmonella enterica	4.2.1.90
B4TBF8	rhamnonate dehydratase	V	Salmonella heidelberg	4.2.1.90
C0Q077	rhamnonate dehydratase	V	Salmonella paratyphi	4.2.1.90
A0A0D6FCA4	rhamnonate dehydratase	V	Salmonella typhimurium	4.2.1.90
V3TSQ7	rhamnonate dehydratase	V	Serratia sp.	4.2.1.90
Q31Z80	rhamnonate dehydratase	V	Shigella boydii	4.2.1.90
Q3YZV9	rhamnonate dehydratase	V	Shigella sonnei	4.2.1.90
A0A087NC78	rhamnonate dehydratase	V	Sphingobium sp.	4.2.1.90
J2WPE2	rhamnonate dehydratase	V	Sphingobium sp.	4.2.1.90
W1S8C1	rhamnonate dehydratase	V	Sphingobium sp.	4.2.1.90
A0A085K6T7	rhamnonate dehydratase	V	Sphingobium yanoikuyae	4.2.1.90
K9DAW4	rhamnonate dehydratase	V	Sphingobium yanoikuyae	4.2.1.90
W0AIP8	rhamnonate dehydratase	V	Sphingomonas sanxanigenens	4.2.1.90
A0A031HNS3	rhamnonate dehydratase	V	Sphingomonas sp.	4.2.1.90
Q1NEI8	rhamnonate dehydratase	V	Sphingomonas sp.	4.2.1.90
A0A097EF78	rhamnonate dehydratase	V	Sphingomonas taxi	4.2.1.90
A0A097EKH1	rhamnonate dehydratase	V	Sphingomonas taxi	4.2.1.90
C4LDK0	rhamnonate dehydratase	V	Tolumonas auensis	4.2.1.90
A0A085A9R5	rhamnonate dehydratase	V	Trabulsiella guamensis	4.2.1.90
T1XDA0	rhamnonate dehydratase	V	Variovorax paradoxus	4.2.1.90
T1XJ81	rhamnonate dehydratase	V	Variovorax paradoxus	4.2.1.90

Table S2.6. (continuation)

UniProt	Family [3]	Cluster	Species	EC Number
A1WFL2	rhamnonate dehydratase	V	Verminephrobacter eiseniae	4.2.1.90
E1QSX7	rhamnonate dehydratase	V	Vulcanisaeta distributa	4.2.1.90
S2VK30	rhamnonate dehydratase	XI	Actinotignum schaalii	4.2.1.90
G8MB95	rhamnonate dehydratase	XI	Burkholderia sp.	4.2.1.90
K1PQQ4	rhamnonate dehydratase	XI	Crassostrea gigas	
F8GV68	rhamnonate dehydratase	XI	Cupriavidus necator	4.2.1.90
M5GEV7	rhamnonate dehydratase	XI	Dacryopinax sp.	
R1CGL0	rhamnonate dehydratase	XI	Emiliania huxleyi	
F0XHP9	rhamnonate dehydratase	XI	Grosmannia clavigera	
E1VAZ5	rhamnonate dehydratase	XI	Halomonas elongata	4.2.1.90
V4B4V5	rhamnonate dehydratase	XI	Lottia gigantea	
G1XX26	rhamnonate dehydratase	XI	Nitrospirillum amazonense	4.2.1.90
F8PMR8	rhamnonate dehydratase	XI	Serpula lacrymans	
K0RDA3	rhamnonate dehydratase	XI	Thalassiosira oceanica	
F0YC34	rhamnonate dehydratase	XII	Aureococcus anophagefferens	
F2UQQ3	rhamnonate dehydratase	XII	Salpingoeca rosetta	
A0A086TBU1	rhamnonate dehydratase	II	Acremonium chrysogenum	
G1XMZ4	rhamnonate dehydratase	II	Arthrobotrys oligospora	
E4UX24	rhamnonate dehydratase	II	Arthroderma gypseum	
C5FYA8	rhamnonate dehydratase	II	Arthroderma otae	
A1CDC2	rhamnonate dehydratase	II	Aspergillus clavatus	
G7XRY0	rhamnonate dehydratase	II	Aspergillus kawachii	
A2R1E3	rhamnonate dehydratase	II	Aspergillus niger	5.1.2.2
A0A017S8F5	rhamnonate dehydratase	II	Aspergillus ruber	
Q0CPR8	rhamnonate dehydratase	II	Aspergillus terreus	
A0A074VLK5	rhamnonate dehydratase	II	Aureobasidium melanogenum	
A0A074WMC6	rhamnonate dehydratase	II	Aureobasidium namibiae	
A0A074X8G3	rhamnonate dehydratase	II	Aureobasidium pullulans	
A0A074YDT8	rhamnonate dehydratase	II	Aureobasidium subglaciale	
M2NH86	rhamnonate dehydratase	II	Baudoinia compniacensis	
A0A0A2VBM1	rhamnonate dehydratase	II	Beauveria bassiana	
J4VQG6	rhamnonate dehydratase	II	Beauveria bassiana	
G2YE24	rhamnonate dehydratase	II	Botryotinia fuckeliana	
G3B403	rhamnonate dehydratase	II	Candida tenuis	
W9ZH00	rhamnonate dehydratase	II	Capronia coronata	
W9YHN1	rhamnonate dehydratase	II	Capronia epimyces	
Q2GUB7	rhamnonate dehydratase	II	Chaetomium globosum	
G0S9F2	rhamnonate dehydratase	II	Chaetomium thermophilum	
V9D318	rhamnonate dehydratase	II	Cladophialophora carrionii	
W9WL80	rhamnonate dehydratase	II	Cladophialophora psammophila	
W9W9A8	rhamnonate dehydratase	II	Cladophialophora yegresii	
M2TKK9	rhamnonate dehydratase	II	Cochliobolus heterostrophus	
L2F9W2	rhamnonate dehydratase	II	Colletotrichum gloeosporioides	
T0LBN3	rhamnonate dehydratase	II	Colletotrichum gloeosporioides	
E3QT50	rhamnonate dehydratase	II	Colletotrichum graminicola	
A0A0D0TTP2	rhamnonate dehydratase	II	Cryptococcus gattii	
E6QYV5	rhamnonate dehydratase	II	Cryptococcus gattii	
F5HDR2	rhamnonate dehydratase	II	Cryptococcus neoformans	
W2S262	rhamnonate dehydratase	II	Cyphellophora europaea	
Q6BQZ6	rhamnonate dehydratase	II	Debaryomyces hansenii	
N1PUI0	rhamnonate dehydratase	II	Dothistroma septosporum	
W7HII3	rhamnonate dehydratase	II	Drechslerella stenobrocha	
Q5B1A8	rhamnonate dehydratase	II	Emericella nidulans	
A0A072PIR0	rhamnonate dehydratase	II	Exophiala aquamarina	
H6BTG1	rhamnonate dehydratase	II	Exophiala dermatitidis	
F9FRZ6	rhamnonate dehydratase	II	Fusarium oxysporum	

Table S2.6. (continuation)

UniProt	Family [3]	Cluster	Species	EC Number
X0B3R7	rhamnonate dehydratase	II	Fusarium oxysporum	
X0GBJ4	rhamnonate dehydratase	II	Fusarium oxysporum	
J3P8C7	rhamnonate dehydratase	II	Gaeumannomyces graminis	
A0A016P7T5	rhamnonate dehydratase	II	Gibberella zeae	
D0VX14	rhamnonate dehydratase	II	Gibberella zeae	
H0EMK3	rhamnonate dehydratase	II	Glarea lozoyensis	
W4JXR4	rhamnonate dehydratase	II	Heterobasidion irregulare	
G9P2W6	rhamnonate dehydratase	II	Hypocrea atroviridis	
G0RPW6	rhamnonate dehydratase	II	Hypocrea jecorina	
F2QN28	rhamnonate dehydratase	II	Komagataella phaffii	4.2.1.90
W6MQW5	rhamnonate dehydratase	II	Kuraishia capsulata	
K2RUY0	rhamnonate dehydratase	II	Macrophomina phaseolina	
L7JIR5	rhamnonate dehydratase	II	Magnaporthe oryzae	
A5DJY7	rhamnonate dehydratase	II	Meyerozyma guilliermondii	
A9VCD0	rhamnonate dehydratase	II	Monosiga brevicollis	
G2QGB5	rhamnonate dehydratase	II	Myceliophthora thermophila	
C7YT92	rhamnonate dehydratase	II	Nectria haematococca	
A1DCA0	rhamnonate dehydratase	II	Neosartorya fischeri	
F8MSA4	rhamnonate dehydratase	II	Neurospora tetrasperma	
W1QBZ6	rhamnonate dehydratase	II	Ogataea parapolymorpha	4.2.1.90
K9FC04	rhamnonate dehydratase	II	Penicillium digitatum	
A0A0A2K1X3	rhamnonate dehydratase	II	Penicillium expansum	
S7ZGY0	rhamnonate dehydratase	II	Penicillium oxalicum	
W6Q3Q3	rhamnonate dehydratase	II	Penicillium roqueforti	
B6HMB3	rhamnonate dehydratase	II	Penicillium rubens	
Q0U9G2	rhamnonate dehydratase	II	Phaeosphaeria nodorum	
A0A093ZW12	rhamnonate dehydratase	II	Pseudogymnoascus sp.	
E3S0H5	rhamnonate dehydratase	II	Pyrenophora teres	
B2W4T0	rhamnonate dehydratase	II	Pyrenophora tritici-repentis	
A0A074S8P4	rhamnonate dehydratase	II	Rhizoctonia solani	
X8JUQ8	rhamnonate dehydratase	II	Rhizoctonia solani	
A3LZU6	rhamnonate dehydratase	II	Scheffersomyces stipitis	
W9CBP7	rhamnonate dehydratase	II	Sclerotinia borealis	
A7EYB0	rhamnonate dehydratase	II	Sclerotinia sclerotiorum	
F7W3B1	rhamnonate dehydratase	II	Sordaria macrospora	
M3C318	rhamnonate dehydratase	II	Sphaerulina musiva	
A0A084RE54	rhamnonate dehydratase	II	Stachybotrys chartarum	
A0A084Q9W9	rhamnonate dehydratase	II	Stachybotrys chlorohalonata	
A0A093XGF0	rhamnonate dehydratase	II	Talaromyces marneffei	
B8LZE6	rhamnonate dehydratase	II	Talaromyces stipitatus	
A0A066VRY4	rhamnonate dehydratase	II	Tilletiaria anomala	
A0A059J771	rhamnonate dehydratase	II	Trichophyton interdigitale	
A0A023A8K4	rhamnonate dehydratase	II	Trichophyton rubrum	
J5RIB2	rhamnonate dehydratase	II	Trichosporon asahii	
D5G8M5	rhamnonate dehydratase	II	Tuber melanosporum	
A0A063C3Z9	rhamnonate dehydratase	II	Ustilaginoidea virens	
F9WZP3	rhamnonate dehydratase	II	Zymoseptoria tritici	

References

1. Melo-Minardi RC, Bastard K, Artiguenave F. Identification of subfamily-specific sites based on active sites modeling and clustering. Bioinformatics. 2010 Dec;26(24):3075–3082.

2. Bastard K, Smith AAT, Vergne-Vaxelaire C, Perret A, Zaparucha A, Melo-Minardi RC, et al. Revealing the hidden functional diversity of an enzyme family. Nat Chem Biol. 2014;10:42–49.

3. Akiva E, Brown S, Almonacid DE, Barber 2nd AE, Custer AF, Hicks MA, et al. The Structure-Function Linkage Database. Nucl Acids Res. 2014 Jan;42(D1):D521–30.
