# Supplementary material for: Isofunctional Protein Subfamily Detection Using Data Integration and Spectral Clustering
Source: PLoS Comput Biol. 2016 Jun 27;12(6):e1005001. doi: 10.1371/journal.pcbi.1005001 (PMC4922564; doi:10.1371/journal.pcbi.1005001)
Supplement: S3 Text — (PDF) [file pcbi.1005001.s003.pdf]

Isofunctional Protein Subfamily Detection using Data Integration and Spectral Clustering

Elisa Boari de Lima[1,2,*], Wagner Meira Júnior[2], Raquel Cardoso de Melo-Minardi[2]

1 Department of Biochemistry and Immunology, Federal University of Minas Gerais, Belo Horizonte, MG, Brazil
2 Department of Computer Science, Federal University of Minas Gerais, Belo Horizonte, MG, Brazil

*** eblima@dcc.ufmg.br**

S3 Text: MI values for the best results produced by the GP system

This supplementary text presents the mutual information (MI) values for the best individuals found in each run of the genetic programming (GP) system, calculated as presented in the paper. Results correspond to those obtained using the updated protein sets, i.e., proteins used in [1] which have since become obsolete in UniProt were removed. Tables have the following columns:

1. **Parameters:** indicates the crossover, reproduction, and mutation rates used by the GP system, in *cc_rr_mm* format. Value 70_10_20, for example, indicates the rates used were 70% for crossover, 10% for reproduction, and 20% for mutation.

2. **Run:** seed value used for the random number generator, which also marks the number of the repetition. The seed value must be fixed to allow experiment reproduction.

3. **Positive Graph:** indicates results obtained using the similarity graph built from only the positive values in the similarity matrix calculated by the GP system.

4. **Complete Graph:** indicates results obtained using the similarity graph built from all values in the similarity matrix calculated by the GP system, rescaled to the [0, 1] interval.

5. **Individual:** identifier of the individual which lead to the best clustering.

6. **MI:** mutual information value for the clustering.

7. **Difference:** corresponds to the difference in MI values obtained using each graph construction method. The smaller the difference, the smaller the effect of choosing one method over the other to build the similarity graph from the similarity matrix.

Case study I: nucleotidyl cyclases

Table S3.1. MI values for the best results produced by the GP system for dividing the nucleotidyl cyclases into two clusters.

Parameters	Run	Positive Graph		Complete Graph		Difference
		Individual	MI	Individual	MI	
70_10_20	1	134	25.7782	122	25.7782	0.0000
70_10_20	2	92	25.7541	94	25.7541	0.0000
70_10_20	3	154	25.7782	138	25.7541	0.0241
70_10_20	4	23	25.7782	183	25.7782	0.0000
70_10_20	5	69	25.7782	24	25.7541	0.0241
70_20_10	1	81	25.7782	182	25.7782	0.0000
70_20_10	2	98	25.7782	98	25.7782	0.0000
70_20_10	3	97	25.7541	176	25.7782	-0.0242
70_20_10	4	23	25.7782	182	25.7782	0.0000
70_20_10	5	269	25.7782	24	25.7541	0.0241
80_05_15	1	112	25.7782	144	25.7782	0.0000
80_05_15	2	192	25.7782	191	25.7782	0.0000
80_05_15	3	78	25.7782	81	25.7541	0.0241
80_05_15	4	23	25.7782	65	25.7782	0.0000
80_05_15	5	132	25.7541	24	25.7541	0.0000
80_15_05	1	84	25.7782	167	25.7782	0.0000
80_15_05	2	180	25.7782	99	25.7782	0.0000
80_15_05	3	78	25.7782	203	25.7782	0.0000
80_15_05	4	23	25.7782	90	25.7782	0.0000
80_15_05	5	236	25.7782	270	25.7782	0.0000
80_20_00	1	63	25.7782	135	25.7782	0.0000
80_20_00	2	113	25.7541	268	25.7782	-0.0241
80_20_00	3	78	25.7782	81	25.7541	0.0241
80_20_00	4	23	25.7782	139	25.7782	0.0000
80_20_00	5	116	25.7541	24	25.7541	0.0000
85_05_10	1	73	25.7782	94	25.7782	0.0000
85_05_10	2	61	25.7782	124	25.7541	0.0241
85_05_10	3	278	25.7782	81	25.7541	0.0241
85_05_10	4	23	25.7782	90	25.7782	0.0000
85_05_10	5	293	25.7782	24	25.7541	0.0241
85_10_05	1	84	25.7782	109	25.7782	0.0000
85_10_05	2	134	25.7782	233	25.7782	0.0000
85_10_05	3	269	25.7782	274	25.7782	0.0000
85_10_05	4	23	25.7782	90	25.7782	0.0000
85_10_05	5	137	25.7541	170	25.7782	-0.0241
90_05_05	1	139	25.7782	109	25.7782	0.0000
90_05_05	2	134	25.7782	141	25.7541	0.0241
90_05_05	3	178	25.7782	167	25.7782	0.0000
90_05_05	4	23	25.7782	90	25.7782	0.0000
90_05_05	5	130	25.7541	24	25.7541	0.0000
90_10_00	1	86	25.7782	142	25.7782	0.0000
90_10_00	2	94	25.7541	110	25.7782	-0.0241
90_10_00	3	67	25.7541	192	25.7782	-0.0242
90_10_00	4	23	25.7782	104	25.7782	0.0000
90_10_00	5	263	25.7782	24	25.7541	0.0241

Table S3.2. MI values for the best results produced by the GP system for dividing the nucleotidyl cyclases into three clusters.

Parameters	Run	Positive Graph		Complete Graph		Difference
		Individual	MI	Individual	MI	
70_10_20	1	210	22.4108	10	22.2276	0.1832
70_10_20	2	215	22.2177	29	22.0781	0.1396
70_10_20	3	51	22.2161	40	22.2276	-0.0115
70_10_20	4	166	22.2161	8	22.2276	-0.0115
70_10_20	5	95	22.0829	95	22.2276	-0.1447
70_20_10	1	119	22.4232	10	22.2276	0.1956
70_20_10	2	211	22.2177	29	22.0781	0.1396
70_20_10	3	190	22.3641	40	22.2276	0.1365
70_20_10	4	94	22.2276	8	22.2276	0.0000
70_20_10	5	95	22.0829	102	22.2276	-0.1447
80_05_15	1	63	22.3483	10	22.2276	0.1207
80_05_15	2	158	22.2177	157	22.2276	-0.0100
80_05_15	3	116	22.2276	40	22.2276	0.0000
80_05_15	4	154	22.2276	8	22.2276	0.0000
80_05_15	5	117	22.2177	65	22.2276	-0.0100
80_15_05	1	109	22.2161	10	22.2276	-0.0115
80_15_05	2	87	22.2177	83	22.2276	-0.0100
80_15_05	3	51	22.2161	40	22.2276	-0.0115
80_15_05	4	247	22.1253	8	22.2276	-0.1023
80_15_05	5	270	22.3641	135	22.2276	0.1365
80_20_00	1	109	22.2161	10	22.2276	-0.0115
80_20_00	2	87	22.2177	83	22.2276	-0.0100
80_20_00	3	51	22.2161	40	22.2276	-0.0115
80_20_00	4	227	22.2161	8	22.2276	-0.0115
80_20_00	5	256	22.3641	136	22.2276	0.1365
85_05_10	1	193	22.2161	10	22.2276	-0.0115
85_05_10	2	127	22.2177	236	22.2276	-0.0100
85_05_10	3	124	22.2276	40	22.2276	0.0000
85_05_10	4	221	22.2276	8	22.2276	0.0000
85_05_10	5	246	22.0829	214	22.2276	-0.1447
85_10_05	1	224	22.1420	10	22.2276	-0.0856
85_10_05	2	248	22.1253	94	22.2276	-0.1023
85_10_05	3	124	22.2276	40	22.2276	0.0000
85_10_05	4	110	22.0951	8	22.2276	-0.1326
85_10_05	5	273	22.3641	126	22.2276	0.1365
90_05_05	1	190	22.4232	10	22.2276	0.1956
90_05_05	2	74	22.2161	29	22.0781	0.1380
90_05_05	3	298	22.2276	40	22.2276	0.0000
90_05_05	4	71	22.2276	8	22.2276	0.0000
90_05_05	5	125	22.2177	104	22.2276	-0.0100
90_10_00	1	280	22.1420	10	22.2276	-0.0856
90_10_00	2	87	22.2177	83	22.2276	-0.0100
90_10_00	3	51	22.2161	40	22.2276	-0.0115
90_10_00	4	242	22.3641	8	22.2276	0.1365
90_10_00	5	125	22.2177	15	22.0951	0.1226

Table S3.3. MI values for the best results produced by the GP system for dividing the nucleotidyl cyclases into six clusters.

Parameters	Run	Positive Graph		Complete Graph		Difference
		Individual	MI	Individual	MI	
70_10_20	1	211	16.1195	264	16.1080	0.0114
70_10_20	2	205	16.0963	243	16.1124	-0.0161
70_10_20	3	267	16.1316	126	16.0963	0.0353
70_10_20	4	169	16.1204	236	15.5422	0.5782
70_10_20	5	242	15.9896	220	16.0963	-0.1067
70_20_10	1	230	16.1316	183	16.1080	0.0236
70_20_10	2	191	16.1138	218	16.0963	0.0175
70_20_10	3	203	16.1341	266	16.1273	0.0067
70_20_10	4	267	16.1014	192	16.1204	-0.0191
70_20_10	5	178	15.2173	220	16.0963	-0.8790
80_05_15	1	286	16.1273	108	16.1080	0.0193
80_05_15	2	263	16.1138	228	16.0963	0.0175
80_05_15	3	293	16.1341	98	16.0963	0.0377
80_05_15	4	212	16.1273	151	16.1028	0.0245
80_05_15	5	275	16.1273	264	16.1205	0.0068
80_15_05	1	255	16.0963	216	16.1180	-0.0217
80_15_05	2	245	16.1148	213	16.1243	-0.0095
80_15_05	3	238	16.1341	232	16.1014	0.0327
80_15_05	4	179	15.7321	232	16.1243	-0.3922
80_15_05	5	249	16.0276	252	16.0584	-0.0308
80_20_00	1	255	16.1541	102	16.1080	0.0461
80_20_00	2	251	16.1273	277	16.1243	0.0031
80_20_00	3	264	16.1316	267	16.1014	0.0303
80_20_00	4	217	16.1273	227	16.1204	0.0068
80_20_00	5	192	15.9350	221	16.0963	-0.1614
85_05_10	1	256	16.1341	215	16.1036	0.0305
85_05_10	2	260	16.0963	219	16.1138	-0.0175
85_05_10	3	245	16.1316	285	16.1014	0.0303
85_05_10	4	141	16.1273	122	16.1028	0.0245
85_05_10	5	232	16.1036	262	16.1316	-0.0281
85_10_05	1	162	16.1316	133	16.1080	0.0236
85_10_05	2	289	16.1273	148	16.1138	0.0135
85_10_05	3	277	16.1316	203	16.1014	0.0303
85_10_05	4	141	16.1273	158	16.1204	0.0068
85_10_05	5	256	15.9810	235	16.1080	-0.1270
90_05_05	1	226	16.1273	192	16.1036	0.0238
90_05_05	2	265	16.1138	239	16.0963	0.0175
90_05_05	3	286	16.1341	191	16.0963	0.0377
90_05_05	4	180	16.0802	168	16.1028	-0.0226
90_05_05	5	251	16.1273	274	16.1273	0.0000
90_10_00	1	248	16.1273	215	16.1080	0.0193
90_10_00	2	141	16.0963	265	16.1138	-0.0175
90_10_00	3	263	16.1316	190	16.0963	0.0353
90_10_00	4	177	15.8968	214	15.8600	0.0369
90_10_00	5	288	15.9721	263	16.0963	-0.1242

Case study II: DUF189

This protein family of unknown function was studied in [2], whose main results contained seven clusters obtained by manipulating ASMC's [1] output. Hence, the GP system was run to divide the family into seven clusters. It was also run to divide it into nine clusters, as described in the paper. MI values for the best clusterings found in each run are presented in Tables S3.4 and S3.5.

Table S3.4. MI values for the best results produced by the GP system for dividing the DUF849 family into seven clusters.

Parameters	Run	Positive Graph		Complete Graph		Difference
		Individual	MI	Individual	MI	
70_10_20	1	280	36.4800	268	36.4700	0.0100
70_10_20	2	255	36.3378	254	34.7473	1.5905
70_10_20	3	250	36.1348	274	36.5913	-0.4565
70_10_20	4	92	34.3354	55	33.7960	0.5394
70_10_20	5	256	35.4881	277	35.7840	-0.2959
70_20_10	1	229	36.3417	212	36.3562	-0.0145
70_20_10	2	247	36.3582	262	33.7253	2.6329
70_20_10	3	260	35.8160	56	36.2143	-0.3983
70_20_10	4	92	34.3354	55	33.7960	0.5394
70_20_10	5	169	35.3704	235	35.7334	-0.3631
80_05_15	1	265	36.5131	287	36.5434	-0.0303
80_05_15	2	258	36.2111	293	33.2584	2.9527
80_05_15	3	198	36.1348	150	36.2936	-0.1588
80_05_15	4	55	34.3354	55	33.7960	0.5394
80_05_15	5	121	35.3862	216	36.2514	-0.8652
80_15_05	1	160	36.5131	254	36.5656	-0.0525
80_15_05	2	217	36.3207	243	33.5775	2.7432
80_15_05	3	192	36.1527	96	36.5913	-0.4386
80_15_05	4	55	34.3354	55	33.7960	0.5394
80_15_05	5	250	35.3427	224	35.7914	-0.4488
80_20_00	1	180	35.4427	238	36.4443	-1.0016
80_20_00	2	250	36.3378	270	34.5569	1.7809
80_20_00	3	240	36.1473	117	36.5913	-0.4440
80_20_00	4	169	34.5757	55	33.7960	0.7797
80_20_00	5	194	35.3744	271	35.7888	-0.4144
85_05_10	1	288	36.1612	290	36.3505	-0.1893
85_05_10	2	279	36.4962	278	34.7172	1.7790
85_05_10	3	295	35.3143	251	36.2945	-0.9802
85_05_10	4	55	34.3354	55	33.7960	0.5394
85_05_10	5	290	35.3704	220	35.7888	-0.4184
85_10_05	1	160	36.5131	274	36.5633	-0.0502
85_10_05	2	217	36.3207	209	34.6441	1.6766
85_10_05	3	282	35.9846	252	36.5973	-0.6127
85_10_05	4	55	34.3354	55	33.7960	0.5394
85_10_05	5	256	35.4091	270	35.7686	-0.3595
90_05_05	1	118	36.4291	273	36.5084	-0.0793
90_05_05	2	281	36.4634	289	34.4250	2.0384
90_05_05	3	233	36.1167	257	36.6185	-0.5017
90_05_05	4	55	34.3354	55	33.7960	0.5394
90_05_05	5	174	35.3704	247	35.7045	-0.3342

Table S3.4. (continuation)

Parameters	Run	Positive Graph Individual	MI	Complete Graph Individual	MI	Difference
90_10_00	1	282	35.9436	270	36.5154	-0.5718
90_10_00	2	223	36.4774	239	34.2418	2.2356
90_10_00	3	243	36.1672	274	36.2945	-0.1273
90_10_00	4	55	34.3354	55	33.7960	0.5394
90_10_00	5	207	35.3704	253	35.7840	-0.4136

Table S3.5. MI values for the best results produced by the GP system for dividing the DUF849 family into nine clusters.

Parameters	Run	Positive Graph Individual	MI	Complete Graph Individual	MI	Difference
70_10_20	1	180	30.0869	290	30.3911	-0.3042
70_10_20	2	157	29.7184	284	29.9506	-0.2322
70_10_20	3	27	31.1308	86	30.2573	0.8735
70_10_20	4	156	29.5388	270	28.0474	1.4914
70_10_20	5	78	30.0013	248	28.9187	1.0826
70_20_10	1	223	30.8106	194	30.2873	0.5233
70_20_10	2	171	29.8363	249	29.9506	-0.1143
70_20_10	3	163	31.5659	271	30.3571	1.2088
70_20_10	4	165	27.2626	70	27.3979	-0.1353
70_20_10	5	48	28.0877	99	28.4798	-0.3920
80_05_15	1	266	30.7279	58	30.2873	0.4406
80_05_15	2	145	29.5013	232	30.4943	-0.9930
80_05_15	3	27	31.1308	235	30.0818	1.0490
80_05_15	4	235	30.6274	285	27.5383	3.0891
80_05_15	5	192	28.9631	282	29.7710	-0.8079
80_15_05	1	237	30.2783	58	30.2873	-0.0089
80_15_05	2	134	29.5013	215	29.7786	-0.2773
80_15_05	3	27	31.1308	156	29.8974	1.2334
80_15_05	4	119	27.2038	248	27.5383	-0.3346
80_15_05	5	205	30.0013	249	28.8662	1.1351
80_20_00	1	207	30.9503	228	30.3788	0.5715
80_20_00	2	222	29.8995	258	30.0367	-0.1372
80_20_00	3	27	31.1308	156	29.5412	1.5896
80_20_00	4	190	27.9067	204	28.5153	-0.6086
80_20_00	5	227	30.1823	154	28.9544	1.2279
85_05_10	1	287	30.9924	232	30.3340	0.6584
85_05_10	2	254	29.7116	143	30.4802	-0.7686
85_05_10	3	27	31.1308	173	29.6171	1.5137
85_05_10	4	290	27.9067	176	29.6163	-1.7096
85_05_10	5	178	28.5947	244	27.8711	0.7236
85_10_05	1	242	30.6657	228	30.3184	0.3473
85_10_05	2	138	29.7067	124	29.7373	-0.0306
85_10_05	3	27	31.1308	194	30.6098	0.5210
85_10_05	4	119	27.2038	265	28.5153	-1.3116
85_10_05	5	102	29.1743	199	28.4836	0.6906

Table S3.5. (continuation)

Parameters	Run	Positive Graph		Complete Graph		Difference
		Individual	MI	Individual	MI	
90_05_05	1	285	30.3175	184	30.4663	-0.1488
90_05_05	2	130	29.5013	156	30.3518	-0.8504
90_05_05	3	27	31.1308	158	29.6372	1.4936
90_05_05	4	119	27.2038	70	27.3979	-0.1942
90_05_05	5	79	30.0013	253	28.7216	1.2797
90_10_00	1	228	30.9658	90	30.2873	0.6786
90_10_00	2	206	30.3169	275	30.5261	-0.2092
90_10_00	3	27	31.1308	269	30.1420	0.9888
90_10_00	4	171	28.1251	220	27.5588	0.5663
90_10_00	5	79	30.0013	175	27.6613	2.3400

Case study III: protein kinases

As mentioned in the paper, this protein family contains two main subfamilies: Ser/Thr kinases and Tyr kinases, besides a Tyr kinase subgroup reported in [1] as being Epidermal Growth Factor Receptors (EGFRs). For the updated protein set, ASMC produced, with the same parameter values used in [1] for the original protein set, a hierarchical clustering that divides the family into three and seven clusters, respectively, in its first two levels. Hence, the GP system was run to divide this family into two, three, and seven clusters. MI values for the best clusterings found in each run are presented in Tables S3.6 through S3.8.

Table S3.6. MI values for the best results produced by the GP system for dividing the protein kinases into two clusters.

Parameters	Run	Positive Graph		Complete Graph		Difference
		Individual	MI	Individual	MI	
70_10_20	1	243	109.7282	245	109.5696	0.1587
70_10_20	2	4	109.2974	4	109.2974	0.0000
70_10_20	3	222	109.3256	247	109.0210	0.3046
70_10_20	4	15	109.2974	186	109.6239	-0.3265
70_10_20	5	135	109.8928	84	109.6227	0.2701
70_20_10	1	244	109.7282	87	109.2974	0.4308
70_20_10	2	4	109.2974	4	109.2974	0.0000
70_20_10	3	268	109.4410	270	109.8944	-0.4534
70_20_10	4	263	109.7164	15	109.2974	0.4190
70_20_10	5	135	109.8928	84	109.6227	0.2701
80_05_15	1	178	109.8944	270	109.8944	0.0000
80_05_15	2	4	109.2974	4	109.2974	0.0000
80_05_15	3	267	109.5824	245	109.3815	0.2009
80_05_15	4	239	109.5572	117	109.6614	-0.1041
80_05_15	5	284	109.8928	84	109.6227	0.2701
80_15_05	1	246	109.8944	253	109.9902	-0.0958
80_15_05	2	4	109.2974	4	109.2974	0.0000
80_15_05	3	252	109.6010	281	109.1808	0.4202
80_15_05	4	260	109.7164	15	109.2974	0.4190
80_15_05	5	158	109.8928	84	109.6227	0.2701

Table S3.6. (continuation)

Parameters	Run	Positive Graph		Complete Graph		Difference
		Individual	MI	Individual	MI	
80_20_00	1	273	109.7282	243	109.3703	0.3579
80_20_00	2	4	109.2974	103	109.6227	-0.3253
80_20_00	3	244	109.8928	213	109.0198	0.8730
80_20_00	4	264	109.8928	177	109.6614	0.2314
80_20_00	5	191	109.8928	229	109.6614	0.2314
85_05_10	1	252	109.8944	229	109.8944	0.0000
85_05_10	2	4	109.2974	143	109.6227	-0.3253
85_05_10	3	296	109.7021	220	109.6614	0.0407
85_05_10	4	171	109.7282	15	109.2974	0.4308
85_05_10	5	175	109.8928	84	109.6227	0.2701
85_10_05	1	260	109.9003	205	109.8944	0.0059
85_10_05	2	101	109.8944	140	109.5574	0.3370
85_10_05	3	241	109.3930	230	109.6614	-0.2684
85_10_05	4	157	109.7282	184	109.6614	0.0668
85_10_05	5	112	109.7282	246	109.7282	0.0000
90_05_05	1	242	109.7649	101	109.8944	-0.1295
90_05_05	2	4	109.2974	4	109.2974	0.0000
90_05_05	3	255	109.8928	294	109.0719	0.8209
90_05_05	4	295	109.7282	229	109.6614	0.0668
90_05_05	5	237	109.8928	97	109.6227	0.2701
90_10_00	1	204	109.7282	101	109.8944	-0.1662
90_10_00	2	4	109.2974	145	109.5574	-0.2600
90_10_00	3	287	109.7282	200	109.4864	0.2418
90_10_00	4	284	109.8928	266	109.7282	0.1646
90_10_00	5	234	109.8928	132	109.6227	0.2701

Table S3.7. MI values for the best results produced by the GP system for dividing the protein kinases into three clusters.

Parameters	Run	Positive Graph		Complete Graph		Difference
		Individual	MI	Individual	MI	
70_10_20	1	218	102.3506	274	102.7412	-0.3906
70_10_20	2	159	102.9445	172	102.8691	0.0753
70_10_20	3	287	103.1394	192	102.8009	0.3385
70_10_20	4	105	102.9408	237	102.9373	0.0036
70_10_20	5	46	101.9447	251	102.6676	-0.7229
70_20_10	1	227	102.5444	227	102.7196	-0.1752
70_20_10	2	118	102.9408	267	102.8691	0.0717
70_20_10	3	253	102.8804	215	103.1629	-0.2825
70_20_10	4	250	102.9816	274	102.8691	0.1125
70_20_10	5	245	102.5468	75	102.7477	-0.2009
80_05_15	1	265	102.2702	276	102.7175	-0.4473
80_05_15	2	166	102.9408	206	102.9184	0.0225
80_05_15	3	226	102.8787	277	102.8833	-0.0046
80_05_15	4	167	102.9408	270	102.9184	0.0225
80_05_15	5	251	102.6222	266	102.7477	-0.1255

Table S3.7. (continuation)

Parameters	Run	Positive Graph		Complete Graph		Difference
		Individual	MI	Individual	MI	
80_15_05	1	268	102.6539	263	102.7142	-0.0603
80_15_05	2	18	101.9447	260	102.9184	-0.9737
80_15_05	3	173	102.8804	267	102.8691	0.0112
80_15_05	4	247	102.9816	3	101.6886	1.2930
80_15_05	5	276	102.7329	75	102.7477	-0.0149
80_20_00	1	254	102.6832	259	102.4953	0.1879
80_20_00	2	227	102.9445	207	102.8691	0.0753
80_20_00	3	256	102.8804	192	102.8009	0.0794
80_20_00	4	118	102.9408	267	101.9039	1.0370
80_20_00	5	272	102.5438	75	102.7477	-0.2040
85_05_10	1	255	102.4351	288	102.4728	-0.0377
85_05_10	2	143	102.9408	276	102.9184	0.0225
85_05_10	3	256	102.9445	242	102.9219	0.0225
85_05_10	4	125	102.9408	250	102.9184	0.0225
85_05_10	5	269	102.8263	75	102.7477	0.0786
85_10_05	1	276	102.2992	247	102.6505	-0.3513
85_10_05	2	271	102.7230	188	102.0206	0.7024
85_10_05	3	209	102.8787	267	102.9214	-0.0427
85_10_05	4	191	102.9408	215	102.8691	0.0717
85_10_05	5	249	102.5708	243	102.7865	-0.2157
90_05_05	1	246	102.3885	291	102.7366	-0.3481
90_05_05	2	203	102.9408	231	102.8691	0.0717
90_05_05	3	112	102.8787	285	103.0458	-0.1671
90_05_05	4	237	103.1297	268	102.9837	0.1460
90_05_05	5	247	102.7531	264	102.9888	-0.2358
90_10_00	1	261	102.4485	216	102.7531	-0.3046
90_10_00	2	18	101.9447	222	102.8691	-0.9244
90_10_00	3	265	102.9162	146	103.1629	-0.2466
90_10_00	4	141	102.9408	259	102.9371	0.0037
90_10_00	5	46	101.9447	279	102.8836	-0.9389

Table S3.8. MI values for the best results produced by the GP system for dividing the protein kinases into seven clusters.

Parameters	Run	Positive Graph		Complete Graph		Difference
		Individual	MI	Individual	MI	
70_10_20	1	256	49.9020	176	50.4225	-0.5206
70_10_20	2	261	48.8298	18	48.3159	0.5138
70_10_20	3	236	51.9059	226	50.4563	1.4496
70_10_20	4	17	47.7466	17	47.4240	0.3226
70_10_20	5	149	50.4456	96	51.6303	-1.1848
70_20_10	1	137	49.2668	213	48.9539	0.3129
70_20_10	2	148	48.5169	18	48.3159	0.2009
70_20_10	3	63	49.7334	111	50.0601	-0.3267
70_20_10	4	120	48.2904	17	47.4240	0.8665
70_20_10	5	266	50.2809	169	51.4415	-1.1607

Table S3.8. (continuation)

Parameters	Run	Positive Graph		Complete Graph		Difference
		Individual	MI	Individual	MI	
80_05_15	1	228	49.0882	212	49.0906	-0.0024
80_05_15	2	285	48.5075	18	48.3159	0.1916
80_05_15	3	282	50.6998	207	51.5856	-0.8858
80_05_15	4	245	48.9012	17	47.4240	1.4773
80_05_15	5	236	50.2063	246	50.3479	-0.1416
80_15_05	1	131	49.5268	236	50.6654	-1.1386
80_15_05	2	124	48.1744	175	48.5792	-0.4048
80_15_05	3	113	51.6869	276	50.1051	1.5818
80_15_05	4	157	48.2904	79	48.2885	0.0019
80_15_05	5	249	50.2828	231	51.6303	-1.3475
80_20_00	1	95	49.5268	141	48.9539	0.5730
80_20_00	2	205	48.8258	175	48.5792	0.2467
80_20_00	3	255	52.0297	215	51.5242	0.5055
80_20_00	4	171	48.0079	17	47.4240	0.5840
80_20_00	5	267	50.4456	205	51.6754	-1.2299
85_05_10	1	290	49.2668	281	48.8809	0.3859
85_05_10	2	29	47.7825	18	48.3159	-0.5335
85_05_10	3	262	52.2069	156	49.8595	2.3475
85_05_10	4	286	48.9638	17	47.4240	1.5398
85_05_10	5	281	50.5978	214	50.1263	0.4715
85_10_05	1	95	49.5268	86	48.2885	1.2383
85_10_05	2	178	48.1413	18	48.3159	-0.1747
85_10_05	3	160	51.6869	281	52.4204	-0.7335
85_10_05	4	17	47.7466	79	48.2885	-0.5419
85_10_05	5	267	50.2226	178	51.6303	-1.4077
90_05_05	1	95	49.5268	294	50.4225	-0.8957
90_05_05	2	286	49.2495	18	48.3159	0.9336
90_05_05	3	281	51.6869	192	52.4204	-0.7335
90_05_05	4	137	48.0428	265	48.2885	-0.2457
90_05_05	5	202	50.4582	141	51.6303	-1.1721
90_10_00	1	207	50.0807	160	50.4537	-0.3730
90_10_00	2	212	48.8258	18	48.3159	0.5099
90_10_00	3	222	51.6869	211	52.4204	-0.7335
90_10_00	4	17	47.7466	17	47.4240	0.3226
90_10_00	5	276	50.2226	286	51.4415	-1.2189

Case study IV: serine proteases

This protein family contains three main subfamilies: trypsins, chymotrypsins, and elastases, besides a trypsin subgroup found in [1] which are kallikreins. After removing from the protein set used in [1] those proteins which have since become obsolete in UniProt, ASMC, when applied with the same Cobweb parameter values (-A 1.0 and -C 0.25), does not divide this family. Thus, the main parameter (-C) was reduced in 0.05 decrements until a value was found which divided the family: 0.15, which produced a hierarchical clustering with four and eleven clusters, respectively, in its first two levels. Hence, the GP system was run to divide the serine proteases into four and eleven clusters. It was additionally run to divide the family into twelve clusters, as discussed in the paper. The MI values for the best clusterings found in each run are presented in Tables S3.9 through S3.11.

Table S3.9. MI values for the best results produced by the GP system for dividing the serine proteases into four clusters.

Parameters	Run	Positive Graph		Complete Graph		Difference
		Individual	MI	Individual	MI	
70_10_20	1	222	17.8037	17	16.0867	1.7170
70_10_20	2	241	17.8533	29	16.0867	1.7666
70_10_20	3	184	17.5642	199	17.5364	0.0278
70_10_20	4	17	16.1090	17	16.0867	0.0223
70_10_20	5	102	13.8851	15	16.0691	-2.1840
70_20_10	1	106	17.7094	17	16.0867	1.6227
70_20_10	2	141	17.6544	187	16.2248	1.4295
70_20_10	3	118	17.5642	121	17.5364	0.0278
70_20_10	4	181	17.7156	17	16.0867	1.6289
70_20_10	5	269	13.8507	15	16.0691	-2.2184
80_05_15	1	111	17.7094	17	16.0867	1.6227
80_05_15	2	148	17.6544	237	16.2248	1.4295
80_05_15	3	32	16.1063	32	16.0691	0.0372
80_05_15	4	120	17.5701	237	17.5446	0.0255
80_05_15	5	291	13.8851	15	16.0691	-2.1840
80_15_05	1	211	17.5701	17	16.0867	1.4834
80_15_05	2	135	17.7184	196	17.6072	0.1112
80_15_05	3	234	17.7660	32	16.0691	1.6969
80_15_05	4	17	16.1090	17	16.0867	0.0223
80_15_05	5	167	13.4801	15	16.0691	-2.5889
80_20_00	1	225	17.5701	17	16.0867	1.4834
80_20_00	2	200	17.8351	277	17.6072	0.2279
80_20_00	3	202	17.7660	32	16.0691	1.6969
80_20_00	4	219	17.7156	17	16.0867	1.6289
80_20_00	5	278	13.7223	15	16.0691	-2.3468
85_05_10	1	121	17.7094	17	16.0867	1.6227
85_05_10	2	242	17.5701	29	16.0867	1.4834
85_05_10	3	257	17.8351	32	16.0691	1.7660
85_05_10	4	188	17.7156	17	16.0867	1.6289
85_05_10	5	285	13.6395	15	16.0691	-2.4295
85_10_05	1	148	17.2979	17	16.0867	1.2111
85_10_05	2	242	17.8533	277	16.2248	1.6285
85_10_05	3	286	17.8351	32	16.0691	1.7660
85_10_05	4	17	16.1090	17	16.0867	0.0223
85_10_05	5	276	13.7072	15	16.0691	-2.3619
90_05_05	1	289	17.6843	17	16.0867	1.5976
90_05_05	2	110	17.6544	295	16.2248	1.4295
90_05_05	3	245	17.7660	32	16.0691	1.6969
90_05_05	4	17	16.1090	130	17.6072	-1.4982
90_05_05	5	229	13.8851	15	16.0691	-2.1840
90_10_00	1	146	17.6065	224	16.2214	1.3851
90_10_00	2	110	17.6544	192	16.2248	1.4295
90_10_00	3	272	17.8343	32	16.0691	1.7652
90_10_00	4	238	17.7094	17	16.0867	1.6227
90_10_00	5	209	13.6937	15	16.0691	-2.3754

Table S3.10. MI values for the best results produced by the GP system for dividing the serine proteases into eleven clusters.

Parameters	Run	Positive Graph		Complete Graph		Difference
		Individual	MI	Individual	MI	
70_10_20	1	163	11.8605	138	11.2046	0.6559
70_10_20	2	233	11.5305	29	10.6556	0.8749
70_10_20	3	210	10.9983	40	10.9025	0.0958
70_10_20	4	197	11.8265	17	11.2644	0.5621
70_10_20	5	135	11.8437	252	11.8432	0.0005
70_20_10	1	10	11.5110	140	11.2046	0.3064
70_20_10	2	265	11.5305	29	10.6556	0.8749
70_20_10	3	110	11.1309	40	10.9025	0.2284
70_20_10	4	252	11.8265	17	11.2644	0.5621
70_20_10	5	248	11.9697	192	11.7837	0.1859
80_05_15	1	243	12.0891	133	11.2046	0.8845
80_05_15	2	268	10.8520	29	10.6556	0.1963
80_05_15	3	125	11.1309	40	10.9025	0.2284
80_05_15	4	17	11.3230	17	11.2644	0.0586
80_05_15	5	148	11.9727	270	11.8234	0.1493
80_15_05	1	10	11.5110	103	11.2046	0.3064
80_15_05	2	87	11.0820	125	11.7242	-0.6422
80_15_05	3	40	10.2737	40	10.9025	-0.6288
80_15_05	4	17	11.3230	17	11.2644	0.0586
80_15_05	5	241	11.7153	140	11.4526	0.2627
80_20_00	1	10	11.5110	17	11.0393	0.4717
80_20_00	2	87	11.0820	249	11.7524	-0.6704
80_20_00	3	128	11.1309	40	10.9025	0.2284
80_20_00	4	17	11.3230	17	11.2644	0.0586
80_20_00	5	251	11.3166	231	11.5857	-0.2691
85_05_10	1	10	11.5110	138	11.2046	0.3064
85_05_10	2	271	11.8600	29	10.6556	1.2043
85_05_10	3	223	11.4356	40	10.9025	0.5331
85_05_10	4	17	11.3230	17	11.2644	0.0586
85_05_10	5	265	11.8761	280	11.7637	0.1123
85_10_05	1	10	11.5110	128	11.4406	0.0705
85_10_05	2	87	10.6629	29	10.6556	0.0073
85_10_05	3	110	11.1507	40	10.9025	0.2482
85_10_05	4	17	11.3230	17	11.2644	0.0586
85_10_05	5	279	11.6609	176	11.7421	-0.0812
90_05_05	1	10	11.5110	132	11.2046	0.3064
90_05_05	2	87	10.6629	29	10.6556	0.0073
90_05_05	3	110	11.1507	40	10.9025	0.2482
90_05_05	4	247	11.8361	17	11.2644	0.5718
90_05_05	5	206	11.8761	151	11.6679	0.2082
90_10_00	1	10	11.5110	17	11.0393	0.4717
90_10_00	2	87	10.6629	141	10.8721	-0.2092
90_10_00	3	40	10.2737	40	10.9025	-0.6288
90_10_00	4	17	11.3230	17	11.2644	0.0586
90_10_00	5	209	11.8555	206	11.7837	0.0718

Table S3.11. MI values for the best results produced by the GP system for dividing the serine proteases into twelve clusters.

Parameters	Run	Positive Graph		Complete Graph		Difference
		Individual	MI	Individual	MI	
70_10_20	1	280	11.3348	202	11.1580	0.1768
70_10_20	2	171	10.5592	29	10.4847	0.0745
70_10_20	3	40	10.7440	180	10.9399	-0.1959
70_10_20	4	91	11.0090	91	10.9940	0.0150
70_10_20	5	261	11.6845	108	10.4934	1.1911
70_20_10	1	17	10.6734	147	11.0149	-0.3415
70_20_10	2	173	11.2264	212	11.3942	-0.1678
70_20_10	3	40	10.7440	198	10.8163	-0.0723
70_20_10	4	175	10.9940	175	10.9940	0.0000
70_20_10	5	155	11.0997	277	11.4691	-0.3693
80_05_15	1	292	11.4672	288	11.3254	0.1418
80_05_15	2	214	10.7339	271	11.0456	-0.3116
80_05_15	3	212	10.8992	238	10.9399	-0.0407
80_05_15	4	8	10.3784	149	10.9940	-0.6156
80_05_15	5	284	11.4285	195	10.4934	0.9351
80_15_05	1	17	10.6734	211	11.0152	-0.3418
80_15_05	2	256	11.2216	249	10.8674	0.3543
80_15_05	3	40	10.7440	152	10.9399	-0.1959
80_15_05	4	123	10.9940	123	10.9940	0.0000
80_15_05	5	264	11.3837	112	11.3306	0.0531
80_20_00	1	166	11.4060	196	11.2575	0.1485
80_20_00	2	248	11.1601	203	11.1167	0.0433
80_20_00	3	40	10.7440	181	10.9399	-0.1959
80_20_00	4	79	10.9940	79	10.9940	0.0000
80_20_00	5	224	11.3422	184	11.3306	0.0116
85_05_10	1	240	11.5038	284	11.0152	0.4885
85_05_10	2	89	10.5935	279	11.3942	-0.8006
85_05_10	3	270	11.1439	132	10.9399	0.2040
85_05_10	4	123	11.0090	119	10.9940	0.0150
85_05_10	5	274	11.3549	114	11.3306	0.0243
85_10_05	1	275	11.6060	247	11.4481	0.1580
85_10_05	2	155	11.2216	29	10.4847	0.7369
85_10_05	3	104	10.9987	132	10.9399	0.0588
85_10_05	4	8	10.3784	134	10.9940	-0.6156
85_10_05	5	282	11.3541	114	11.3306	0.0235
90_05_05	1	172	11.2728	280	11.0152	0.2575
90_05_05	2	244	11.2216	29	10.4847	0.7369
90_05_05	3	104	10.9987	132	10.9399	0.0588
90_05_05	4	187	11.0198	274	11.3491	-0.3293
90_05_05	5	281	11.5906	293	11.3433	0.2474
90_10_00	1	211	11.4060	283	11.0684	0.3376
90_10_00	2	151	11.1116	252	10.9791	0.1325
90_10_00	3	40	10.7440	159	10.9399	-0.1959
90_10_00	4	96	10.9940	96	10.9940	0.0000
90_10_00	5	252	11.4754	293	11.6968	-0.2214

References

1. Melo-Minardi RC, Bastard K, Artiguenave F. Identification of subfamily-specific sites based on active sites modeling and clustering. Bioinformatics. 2010 Dec;26(24):3075–3082.

2. Bastard K, Smith AAT, Vergne-Vaxelaire C, Perret A, Zaparucha A, Melo-Minardi RC, et al. Revealing the hidden functional diversity of an enzyme family. Nat Chem Biol. 2014;10:42–49.
