# Supplementary material for: Isofunctional Protein Subfamily Detection Using Data Integration and Spectral Clustering
Source: PLoS Comput Biol. 2016 Jun 27;12(6):e1005001. doi: 10.1371/journal.pcbi.1005001 (PMC4922564; doi:10.1371/journal.pcbi.1005001)
Supplement: S4 Text — (PDF) [file pcbi.1005001.s004.pdf]

Isofunctional Protein Subfamily Detection using Data Integration and Spectral Clustering

Elisa Boari de Lima[1,2,*], Wagner Meira Júnior[2], Raquel Cardoso de Melo-Minardi[2]

1 Department of Biochemistry and Immunology, Federal University of Minas Gerais, Belo Horizonte, MG, Brazil
2 Department of Computer Science, Federal University of Minas Gerais, Belo Horizonte, MG, Brazil

*** eblima@dcc.ufmg.br**

S4 Text: Dividing the nucleotidyl cyclases into three clusters

In the first level of ASMC's hierarchical clustering, the family was divided into three clusters, whose logos and compositions according to the subfamily labels are presented in Fig. S4.1. One may note that, instead of finding clusters related to the existing subfamilies, ASMC prioritized adenylate cyclase subgroups, while the bulk of the family was put into the same cluster.

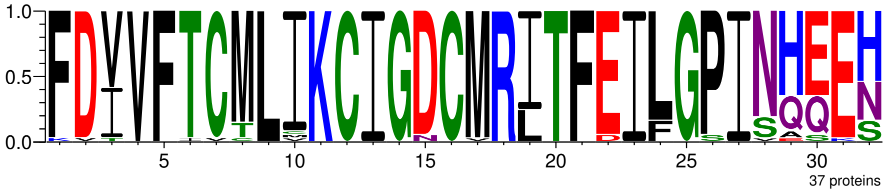

(a) Cluster I: 37 adenylate cyclases

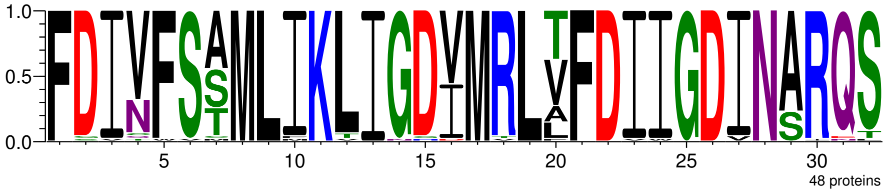

(b) Cluster II: 48 adenylate cyclases

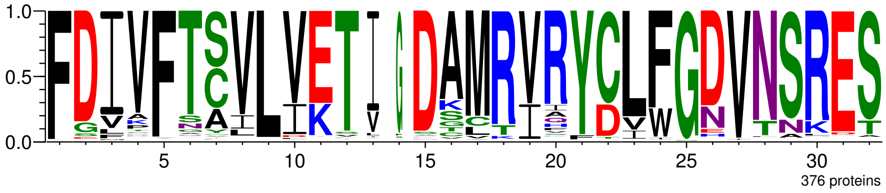

(c) Cluster III: 275 guanylate cyclases and 101 adenylate cyclases

Figure S4.1. Nucleotidyl cyclase division into three clusters in the first level of ASMC's hierarchical clustering.

Given ASMC's criteria for considering as specificity determining positions (SDPs) those positions with p-values smaller than 0.0001 [1], the SDPs per cluster for this clustering are presented in Table S4.1. Although ASMC was unable to separate the adenylate and guanylate subfamilies, the known SDPs for this family, which were listed in the main text, are present among those considered as SDPs by its p-value criteria.

Table S4.1. Cluster SDPs for the three nucleotidyl cyclase clusters produced by ASMC.

Cluster	Positions
I	12_{524}, 16_{528}, $\mathbf{20_{590}}$, 21_{591}, $\mathbf{22_{592}}$, $\mathbf{24_{594}}$, 26_{596}, 27_{598}, 29_{602}, 30_{603}, 32_{606}
II	6_{487}, 8_{498}, 12_{524}, 19_{584}, 21_{591}, $\mathbf{22_{592}}$, $\mathbf{23_{593}}$, $\mathbf{24_{594}}$, 27_{598}, 29_{602}, 31_{605}
III	8_{498}, 10_{522}, $\mathbf{11_{523}}$, 12_{524}, 16_{528}, 19_{584}, $\mathbf{20_{590}}$, 21_{591}, $\mathbf{22_{592}}$, $\mathbf{23_{593}}$, $\mathbf{24_{594}}$, 27_{598}, 29_{602}

Listed in order of active site position. Positions in bold correspond to known SDPs. Subscripted positions correspond to those in PDB structure 3ET6:A.

Despite having already successfully separated the family into its two subfamilies, the GP system was run to divide it into three clusters for comparison with those produced by ASMC. The clusters in ASMC's first hierarchy level have MI = 22.16, while the best clustering obtained by our GP system for three clusters has MI = 22.35 and uses the similarity matrix calculated from the combination of active site scores, difference in molecular weights, and global sequence alignment scores, as shown in the main text. Cluster logos and compositions are presented in Fig. S4.2. One may observe that both techniques found the same 37 adenylate cyclase subgroup. However, unlike ASMC, the GP system maintained the subfamily division in the other two clusters.

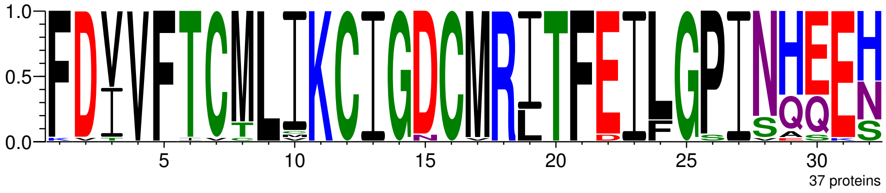

(a) Cluster I: 37 adenylate cyclases

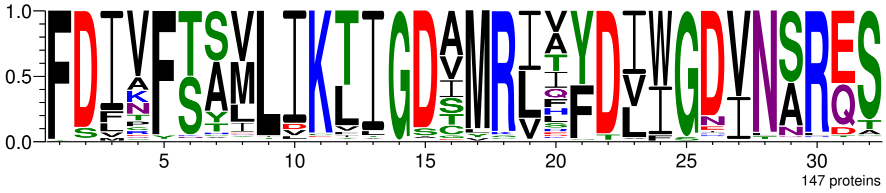

(b) Cluster II: 146 adenylate cyclases and 1 guanylate cyclase

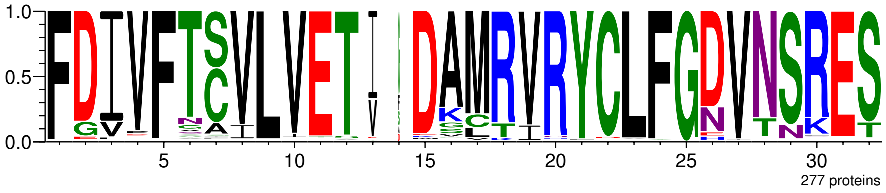

(c) Cluster III: 274 guanylate cyclases and 3 adenylate cyclases

Figure S4.2. Nucleotidyl cyclase division into three clusters by the GP system.

The guanylate cyclase-labeled protein inserted into an adenylate cyclase cluster is Q5UFR4, which we have shown in the main text to have been erroneously labeled. In turn, the three adenylate cyclase-labeled proteins inserted into the guanylate cyclase cluster, namely A0DT50, A0CM46, and A0DT51, are all unreviewed and lack subfamily-specific annotations. Hence, we cannot state that what is correct is the subfamily label adopted in [1] or the clustering produced by the GP system.

The residues which most distinguish each cluster are listed in Table S4.2, in which one may observe the known SDPs for this family are present when they distinguish the corresponding clusters. We note that ASMC's SDP criteria concerns positions, while our partial MI values are able to pinpoint the specific residues in such positions that are most important in distinguishing a cluster.

Table S4.2. Most important residues for the three nucleotidyl cyclase clusters produced by the GP system.

Cluster	Residues
**I**	$C12_{524}$, $E22_{592}$, $P26_{596}$, $C16_{528}$, $\mathbf{L24_{594}}$, $\mathbf{T20_{590}}$
**II**	$\mathbf{D22_{592}}$, $\mathbf{K11_{523}}$, $G14_{526}$, $I10_{522}$, $\mathbf{W24_{594}}$
**III**	$\mathbf{C22_{592}}$, $\mathbf{E11_{523}}$, $\mathbf{R20_{590}}$, $V10_{522}$, $\mathbf{F24_{594}}$, $\mathbf{L23_{593}}$, $V19_{584}$

Listed in decreasing order of partial MI value. Residues in bold correspond to known SDPs. Subscripted positions correspond to those in PDB structure 3ET6:A.

References

1. Melo-Minardi RC, Bastard K, Artiguenave F. Identification of subfamily-specific sites based on active sites modeling and clustering. Bioinformatics. 2010 Dec;26(24):3075–3082.
