# Supplementary material for: Isofunctional Protein Subfamily Detection Using Data Integration and Spectral Clustering
Source: PLoS Comput Biol. 2016 Jun 27;12(6):e1005001. doi: 10.1371/journal.pcbi.1005001 (PMC4922564; doi:10.1371/journal.pcbi.1005001)
Supplement: S5 Text — (PDF) [file pcbi.1005001.s005.pdf]

Isofunctional Protein Subfamily Detection using Data Integration and Spectral Clustering

Elisa Boari de Lima[1,2,*], Wagner Meira Júnior[2], Raquel Cardoso de Melo-Minardi[2]

1 Department of Biochemistry and Immunology, Federal University of Minas Gerais, Belo Horizonte, MG, Brazil
2 Department of Computer Science, Federal University of Minas Gerais, Belo Horizonte, MG, Brazil

*** eblima@dcc.ufmg.br**

S5 Text: Dividing the nucleotidyl cyclases into six clusters

Fig. S5.1 presents the logos and compositions of the six clusters that exist in the second level of ASMC's hierarchical clustering. In order to create a guanylate cyclase cluster, ASMC needed to fragment the adenylate cyclases into five subgroups, even though this subfamily does not present much residue variability in the active site.

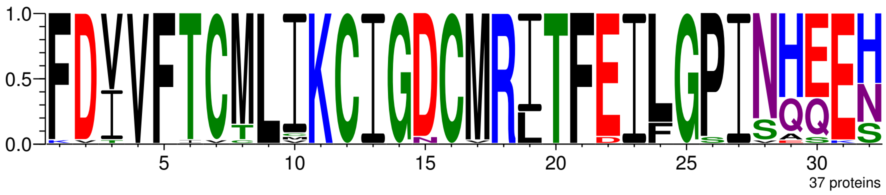

(a) Cluster I: 37 adenylate cyclases

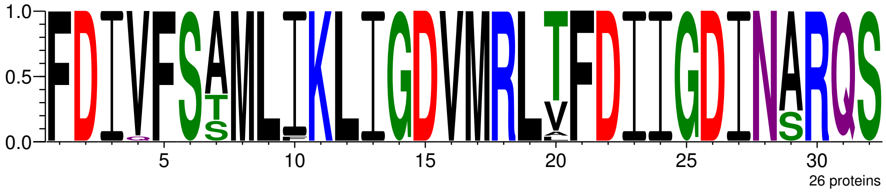

(b) Cluster II.A: 26 adenylate cyclases

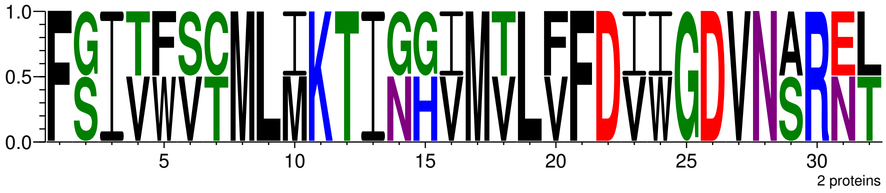

(c) Cluster II.B: 2 adenylate cyclases

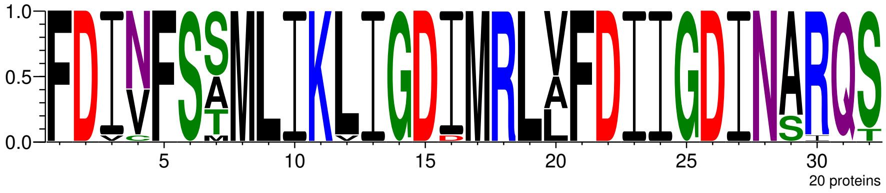

(d) Cluster II.C: 20 adenylate cyclases

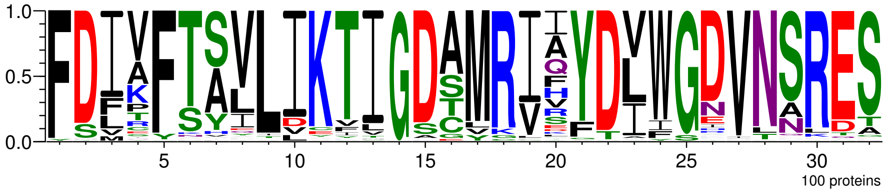

(e) Cluster III.A: 99 adenylate cyclases and 1 guanylate cyclase

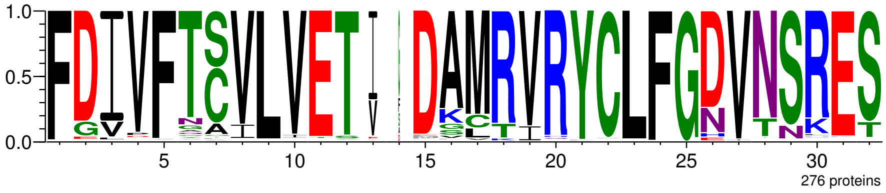

(f) Cluster III.B: 274 guanylate cyclases and 2 adenylate cyclases

Figure S5.1. Nucleotidyl cyclase division into six clusters in the second level of ASMC's hierarchical clustering.

Given ASMC's criteria for considering as specificity determining positions (SDPs) those positions with p-values smaller than 0.0001 [1], the SDPs per cluster for this clustering are presented in Table S5.1. Although some of the family's known SDPs, which were listed in the main text, are present, the residues in such positions do not necessarily distinguish the corresponding clusters from the others, as is the case for positions 22 and 23, considered as SDPs for cluster III.A.

Table S5.1. **Cluster SDPs for the three nucleotidyl cyclase clusters produced by ASMC.**

Cluster	Positions
**I**	12_{524}, 16_{528}, $\mathbf{20_{590}}$, 21_{591}, $\mathbf{22_{592}}$, 24_{594}, 26_{596}, 27_{598}, 29_{602}, 30_{603}, 32_{606}
**II.A**	12_{524}, 31_{605}
**II.B**	15_{527}
**II.C**	4_{484}, 12_{524}, 31_{605}
**III.A**	$\mathbf{22_{592}}$, $\mathbf{23_{593}}$, 24_{594}
**III.B**	8_{498}, 10_{522}, $\mathbf{11_{523}}$, 19_{584}, $\mathbf{20_{590}}$, 21_{591}, $\mathbf{22_{592}}$, $\mathbf{23_{593}}$, 24_{594}

Listed in order of active site position. Positions in bold correspond to known SDPs. Subscripted positions correspond to those in PDB structure 3ET6:A.

For comparison purposes, the GP system was run to divide the nucleotidyl cyclases into six clusters. The data combination that obtained the best result (MI = 16.13) involves six data types, as presented in the main text, with emphasis on active site identity, as expected due to the employed quality measure. The cluster logos and compositions are presented in Fig. S5.2, in which one may observe that each subfamily was divided into three homogeneous subgroups.

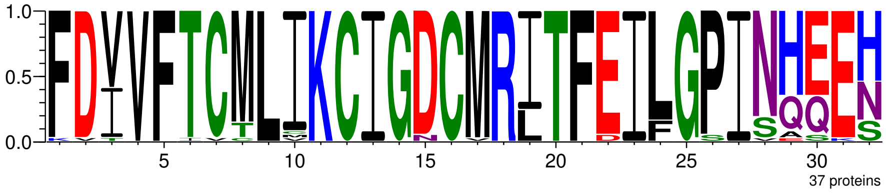

(a) Cluster I: 37 adenylate cyclases

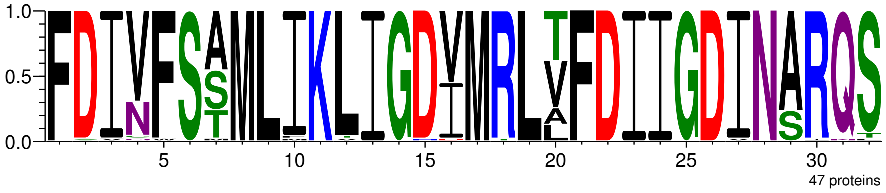

(b) Cluster II: 47 adenylate cyclases

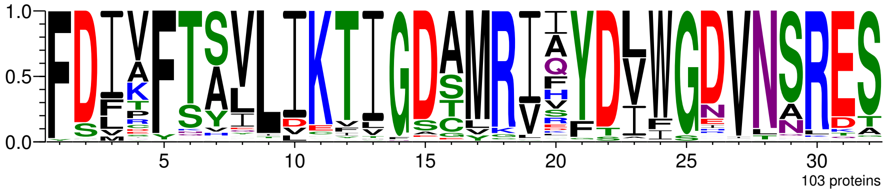

(c) Cluster III: 100 adenylate cyclases and 3 guanylate cyclases

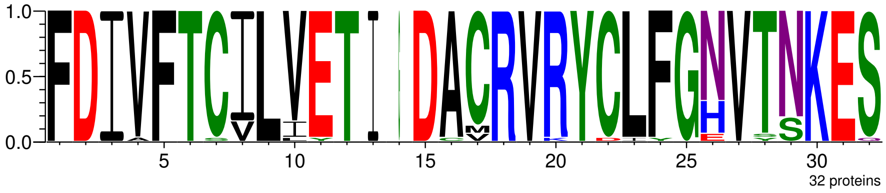

(d) Cluster IV: 31 guanylate cyclases and 1 adenylate cyclase

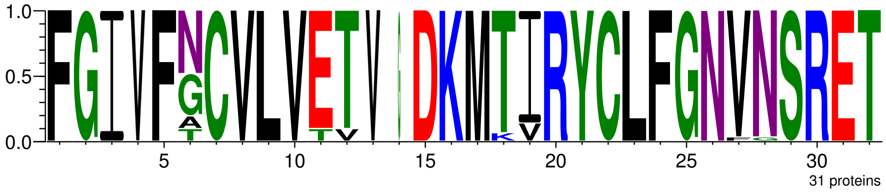

(e) Cluster V: 31 guanylate cyclases

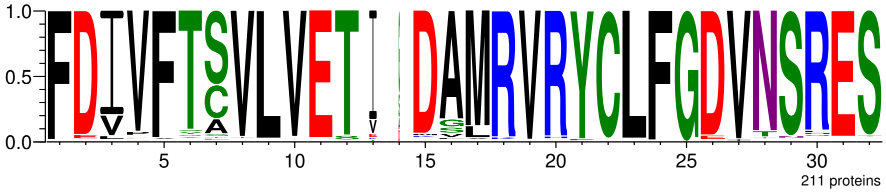

(f) Cluster VI: 210 guanylate cyclases and 1 adenylate cyclase

Figure S5.2. Nucleotidyl cyclase division into six clusters by the GP system.

In Cluster III, which is composed mostly of adenylate cyclases, there are three guanylate cyclase-labeled proteins, namely Q5UFR4, Q4XVA2 and Q4YH96, none of which have been manually reviewed. We have previously established that the first has been incorrectly labeled. As for the others, none has any subfamily-specific annotation apart from the suggested name of *guanylyl cyclase, putative*, which is a weak annotation, but suggests the GP system may have erroneously clustered them. Clusters IV and VI, composed mostly of guanylate cyclases, each have an adenylate cyclase-labeled protein, namely A0DT51 and A7RY93, respectively. None has any subfamily-related annotations, so we cannot state that the labels are wrong or that the GP system erroneously placed them into guanylate cyclase clusters.

The residues which most distinguish each cluster are listed in Table S5.2, in which one may notice that known SDPs are present, although they do not all distinguish among the subclusters for each subfamily. This was to be expected, since the known SDPs are valid for the whole subfamilies.

Table S5.2. Most important residues for the six nucleotidyl cyclase clusters produced by the GP system.

Cluster	Residues
**I**	$C12_{524}$, $\mathbf{E22_{592}}$, $P26_{596}$, $C16_{528}$, $L24_{594}$, $\mathbf{T20_{590}}$, $I27_{598}$, $H29_{602}$, $E30_{603}$
**II**	$L12_{524}$, $Q31_{605}$, $I24_{594}$, $L19_{584}$, $S6_{487}$, $M8_{498}$, $I27_{598}$, $F21_{591}$, $\mathbf{I23_{593}}$
**III**	$W24_{594}$, $\mathbf{D22_{592}}$, $\mathbf{K11_{523}}$
**IV**	$K30_{603}$, $C17_{530}$, $T28_{599}$, $I8_{498}$, $N29_{602}$
**V**	$K16_{528}$, $G2_{482}$, $T18_{571}$, $T32_{606}$, $N26_{596}$
**VI**	$\mathbf{C22_{592}}$, $V10_{522}$, $\mathbf{E11_{523}}$, $V19_{584}$, $F24_{594}$, $\mathbf{R20_{590}}$

Listed in decreasing order of partial MI value. Residues in bold correspond to known SDPs. Subscripted positions correspond to those in PDB structure 3ET6:A.

References

1. Melo-Minardi RC, Bastard K, Artiguenave F. Identification of subfamily-specific sites based on active sites modeling and clustering. Bioinformatics. 2010 Dec;26(24):3075–3082.
