# Supplementary material for: Isofunctional Protein Subfamily Detection Using Data Integration and Spectral Clustering
Source: PLoS Comput Biol. 2016 Jun 27;12(6):e1005001. doi: 10.1371/journal.pcbi.1005001 (PMC4922564; doi:10.1371/journal.pcbi.1005001)
Supplement: S6 Text — (PDF) [file pcbi.1005001.s006.pdf]

Isofunctional Protein Subfamily Detection using Data Integration and Spectral Clustering

Elisa Boari de Lima[1,2,*], Wagner Meira Júnior[2], Raquel Cardoso de Melo-Minardi[2]

1 Department of Biochemistry and Immunology, Federal University of Minas Gerais, Belo Horizonte, MG, Brazil
2 Department of Computer Science, Federal University of Minas Gerais, Belo Horizonte, MG, Brazil

*** eblima@dcc.ufmg.br**

S6 Text: Dividing the protein kinases into three clusters

In the first level of ASMC's hierarchical clustering, the protein kinases were divided into three clusters, whose logos and compositions according to subfamily labels in [1] are presented in Fig. S6.1. Such clusters have MI = 67.46. As mentioned in the main text, the structural models for some of the family's proteins did not align well with the reference structure's active site, which yielded numerous gaps for positions uninvolved in the consensus sequences. ASMC created a cluster containing such proteins. However, gaps are not function-related, so such a clustering is uninformative. Additionally, despite creating an EGFR cluster, 87% of the family was inserted into cluster III, thus mixing the two main subfamilies. This result is very different from the one presented in [1], which shows ASMC is unstable in terms of the clusters it generates, since removing only 9.2% of the proteins yielded a completely different clustering than with the original protein set.

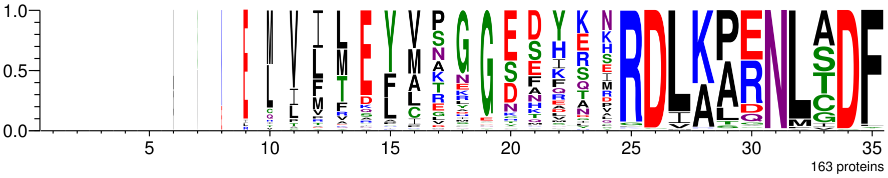

(a) Cluster I: 2 EGFRs, 94 Ser/Thr kinases and 67 Tyr kinases

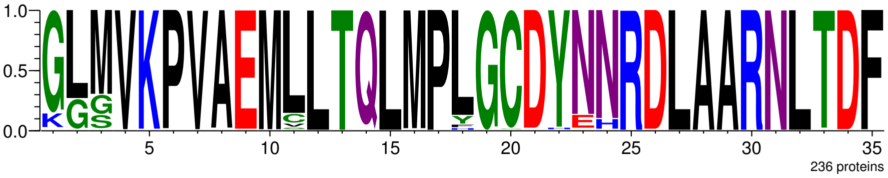

(b) Cluster II: 233 EGFRs and 3 Tyr kinases

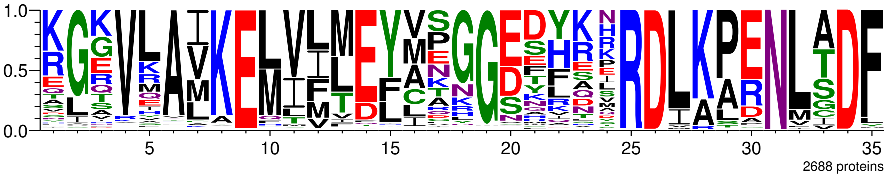

(c) Cluster III: 1.950 Ser/Thr kinases and 738 Tyr kinases

Figure S6.1. Protein kinase division into three clusters in the first level of ASMC's hierarchical clustering.

Given ASMC's criteria for considering as specificity determining positions (SDPs) those positions with p-values smaller than 0.0001 [1], the SDPs per cluster for this clustering are presented in Table S6.1. One may notice the presence of the known SDPs for the family, presented in the main text, despite the subfamily mixture in cluster III, since the Ser/Thr kinases are the cluster's majority subfamily, thus diluting the presence of Tyr kinase-specific residues in such positions. We note that ASMC's SDP criteria concerns positions, while our partial MI values are able to pinpoint the specific residues in such positions that are most important in distinguishing a cluster.

Table S6.1. Cluster SDPs for the three protein kinase clusters produced by ASMC.

Cluster	Positions
**I**	-
**II**	1_{130}, 2_{132}, 3_{133}, 5_{142}, 6_{156}, 7_{157}, 8_{158}, 10_{181}, 11_{190}, 12_{203}, 13_{205}, 14_{206}, 15_{207} 16_{208}, 17_{209}, 18_{210}, 20_{212}, 21_{215}, 22_{216}, 23_{219}, 24_{220}, $\mathbf{28_{254}}$, $\mathbf{29_{255}}$, $\mathbf{30_{256}}$, 33_{269}
**III**	2_{132}, 5_{142}, 6_{156}, 7_{157}, 8_{158}, 10_{181}, 11_{190}, 12_{203}, 13_{205}, 14_{206}, 15_{207}, 18_{210}, 20_{212}, 21_{215}, 22_{216}, 24_{220}, $\mathbf{28_{254}}$, $\mathbf{29_{255}}$, $\mathbf{30_{256}}$

Listed in order of active site position. Positions in bold correspond to known SDPs. Subscripted positions correspond to those in PDB structure 1U46:A.

When the GP system was run to divide the family into three clusters for comparison purposes, the best results presented MI = 102.94 and were obtained combining two data types, as shown in the main text. The cluster logos and compositions are shown in Fig. S6.2, in which one may observe all EGFR-labeled proteins, which are a subset of the Tyr kinases, were inserted into a same uniform cluster, and the other clusters comply almost completely with the subfamily labels, along with the consensus sequences typical of each subfamily.

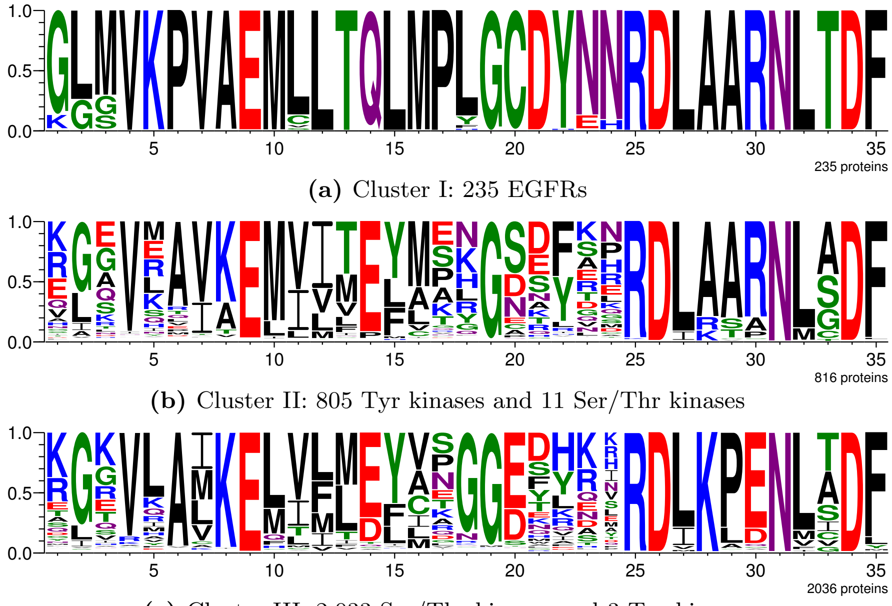

(a) Cluster I: 235 EGFRs

(b) Cluster II: 805 Tyr kinases and 11 Ser/Thr kinases

(c) Cluster III: 2.033 Ser/Thr kinases and 3 Tyr kinases

Figure S6.2. Protein kinase division into three clusters by the GP system.

The most distinguishing residues for each cluster are listed in S6.2, in which one may notice the presence of the family's known SDPs.

Table S6.2. Most important residues for the three protein kinase clusters produced by the GP system.

Cluster	Residues
**I**	P6_{156}, C20_{212}, Q14_{206}, L18_{210}, G1_{130}, A8_{158}, M3_{133}, N23_{219}, K5_{142}, N24_{220}
**II**	$\mathbf{R30_{256}}$, $\mathbf{A28_{254}}$, $\mathbf{A29_{255}}$, F22_{216}, M10_{181}, I12_{203}, S20_{212}
**III**	$\mathbf{K28_{254}}$, $\mathbf{E30_{256}}$, $\mathbf{P29_{255}}$, G18_{210}, E20_{212}, L10_{181}, L5_{142}

Listed in decreasing order of partial MI value. Residues in bold correspond to known SDPs. Subscripted positions correspond to those in PDB structure 1U46:A.

The three Tyr kinase-labeled proteins inserted into Cluster III, whose majority is of Ser/Thr kinases, are unreviewed proteins A2DGV6, Q6K3D4, and O42291. The first is annotated with GO term *protein serine/threonine kinase activity*, which suggests its label may be incorrect and that the GP system correctly included it among the Ser/Thr kinases. The second lacks any subfamily-related annotations, so we cannot evaluate if the label is correct. The latter,

on the other hand, has various Tyr kinase-related annotations, including InterPro domain IPR008266 (*tyrosine-protein kinase, active site*) and GO term *protein tyrosine kinase activity* which, combined, are a strong indicator that the GP system wrongly included this protein with the Ser/Thr kinases.

Considering the eleven Ser/Thr kinase-labeled proteins inserted into the Tyr kinase cluster, two (Q54TM7 and A7J1T0) have been manually curated and annotated as Ser/Thr kinases. All of the remaining (Q4RX00, Q5SMJ0, Q0DE32, Q4S6N1, A7J1T1, A7J1T3, Q5RCD1, Q6NYW1, and A7SFG8) are annotated with InterPro domain IPR008271 (*serine/threonine-protein kinase, active site*). Additionally, three (Q0DE32, Q5RCD1, and Q6NYW1) are annotated with GO term *Ser/Thr kinase activity*. Such annotations indicate the GP system was mistaken about these eleven proteins.

When run for two clusters, the GP system included a set of 64 Tyr kinase-labeled proteins along with the Ser/Thr kinases. However, when considering three clusters, such proteins were grouped with the Tyr kinases, in accordance with their labels in [1]. This reinforces the idea that they may have dual specificity, as well as shows the partitional clustering's ability to repair possible previous errors, as discussed in the main text.

Despite errors for 0.45% of the family's proteins, our framework was able to create relevant clusters with almost total correspondence with the existing protein kinase subfamilies, unlike the clustering produced by ASMC, which prioritized a group of proteins containing numerous gaps.

References

1. Melo-Minardi RC, Bastard K, Artiguenave F. Identification of subfamily-specific sites based on active sites modeling and clustering. Bioinformatics. 2010 Dec;26(24):3075–3082.
