# Supplementary material for: Isofunctional Protein Subfamily Detection Using Data Integration and Spectral Clustering
Source: PLoS Comput Biol. 2016 Jun 27;12(6):e1005001. doi: 10.1371/journal.pcbi.1005001 (PMC4922564; doi:10.1371/journal.pcbi.1005001)
Supplement: S7 Text — (PDF) [file pcbi.1005001.s007.pdf]

Isofunctional Protein Subfamily Detection using Data Integration and Spectral Clustering

Elisa Boari de Lima[1,2,*], Wagner Meira Júnior[2], Raquel Cardoso de Melo-Minardi[2]

1 Department of Biochemistry and Immunology, Federal University of Minas Gerais, Belo Horizonte, MG, Brazil
2 Department of Computer Science, Federal University of Minas Gerais, Belo Horizonte, MG, Brazil

*** eblima@dcc.ufmg.br**

S7 Text: Dividing the protein kinases into seven clusters

The second level of ASMC's [1] hierarchical clustering has seven clusters, whose logos and compositions according to the subfamily labels are presented in Fig. S7.1. One may easily note that, even after increasing the number of clusters, the subfamilies remained mixed. Only the EGFRs were mostly isolated from the other subfamilies, which had already been done in the previous hierarchy level. Furthermore, ASMC generated clusters with irrelevant differences, such as Clusters I.A and I.B. This hierarchy level's utility seems to have been limited to obtaining some relatively uniform Tyr kinase subgroups.

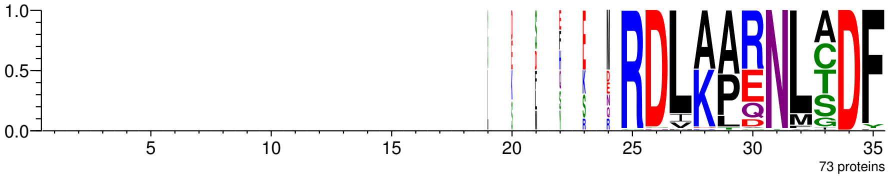

(a) Cluster I.A: 35 Ser/Thr kinases and 38 Tyr kinases

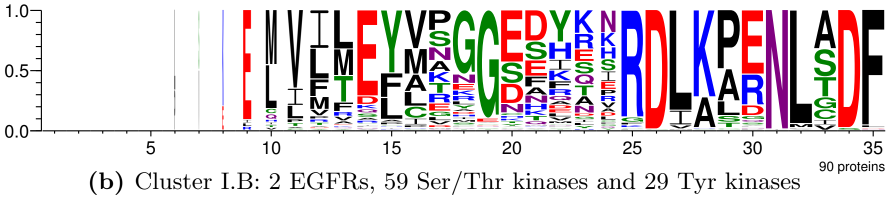

(b) Cluster I.B: 2 EGFRs, 59 Ser/Thr kinases and 29 Tyr kinases

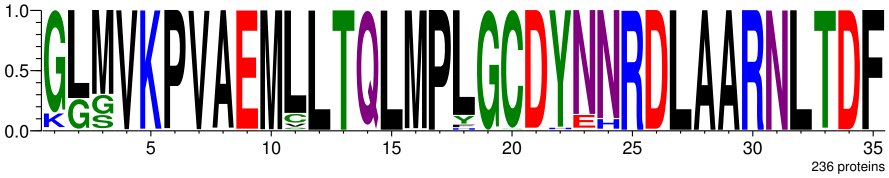

(c) Cluster II: 233 EGFRs and 3 Tyr kinases

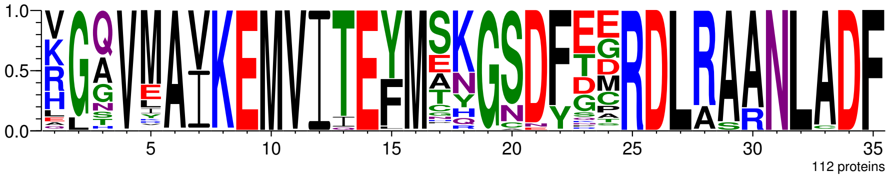

(d) Cluster III.A: 112 Tyr kinases

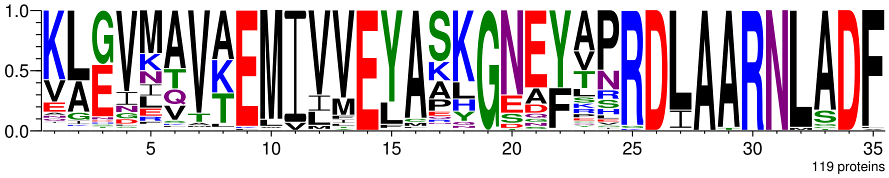

(e) Cluster III.B: 119 Tyr kinases

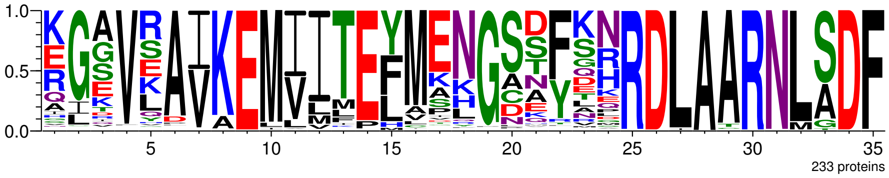

(f) Cluster III.C: 233 Tyr kinases

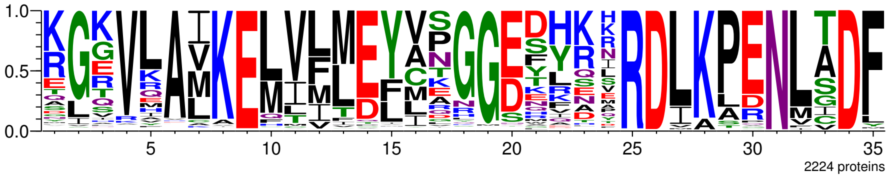

(g) Cluster III.D: 1.950 Ser/Thr kinases and 274 Tyr kinases

Figure S7.1. Protein kinase division into seven clusters in the second level of ASMC's hierarchical clustering.

Given ASMC's criteria for considering as specificity determining positions (SDPs) those positions with p-values smaller than 0.0001 [1], the SDPs per cluster for this clustering are presented in Table S7.1. Again, one may notice the presence of the known SDPs for the family, presented in the main text, although the subfamilies are mixed. This is due to the SDPs being valid for the family as a whole, and to ASMC's SDP criteria concerning positions, rather than specific residues in such positions.

Table S7.1. **Cluster SDPs for the seven protein kinase clusters produced by ASMC.**

Cluster	Positions
**I.A**	-
**I.B**	-
**II**	1_{130}, 2_{132}, 3_{133}, 5_{142}, 6_{156}, 7_{157}, 8_{158}, 10_{181}, 11_{190}, 12_{203}, 13_{205}, 14_{206}, 15_{207} 16_{208}, 17_{209}, 18_{210}, 20_{212}, 21_{215}, 22_{216}, 23_{219}, 24_{220}, $\mathbf{28_{254}}$, $\mathbf{29_{255}}$, $\mathbf{30_{256}}$, 33_{269}
**III.A**	5_{142}, 12_{203}, 18_{210}, 20_{212}, $\mathbf{28_{254}}$, $\mathbf{29_{255}}$, $\mathbf{30_{256}}$
**III.B**	2_{132}, 8_{158}, 11_{190}, 12_{203}, 13_{205}, 18_{210}, 20_{212}, 21_{215}, 24_{220}, $\mathbf{28_{254}}$, $\mathbf{29_{255}}$, $\mathbf{30_{256}}$
**III.C**	10_{181}, 12_{203}, 13_{133}, 16_{208}, 17_{209}, 18_{210}, 20_{212}, 22_{216}, $\mathbf{28_{254}}$, $\mathbf{29_{255}}$, $\mathbf{30_{256}}$, 33_{269}
**III.D**	2_{132}, 3_{133}, 5_{142}, 6_{156}, 8_{158}, 10_{181}, 11_{190}, 13_{205}, 16_{208}, 18_{210}, 20_{212}, 21_{215}, 22_{216}, 23_{219}, $\mathbf{28_{254}}$, $\mathbf{29_{255}}$, $\mathbf{30_{256}}$

Listed in order of active site position. Positions in bold correspond to known SDPs. Subscripted positions correspond to those in PDB structure 1U46:A.

Although the genetic programming (GP) system successfully separated the subfamilies into two and three clusters, it was run with seven clusters in order to compare results with ASMC's hierarchy's second level, which presented MI = 45.99. Obviously, this caused the subfamilies to be broken into subgroups. The best result presented MI = 50.70 and involved four data types, as shown in the main text. The logos and compositions for the seven clusters are presented in Fig. S7.2, while residues which most distinguish each cluster are presented in Table S7.2. One may notice that known SDPs are present for the cases in which the residues which occur at such positions distinguish the corresponding cluster from the others.

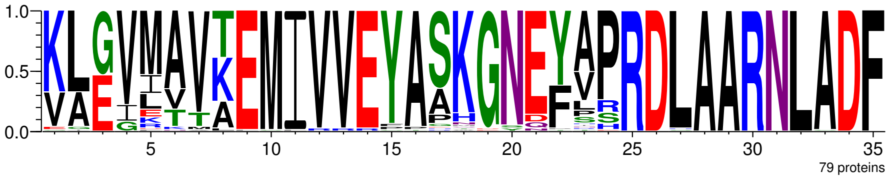

(a) Cluster I: 79 Tyr kinases

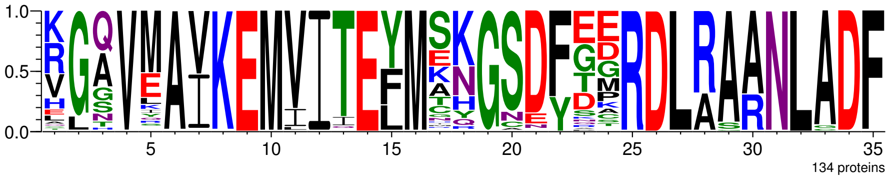

(b) Cluster II: 134 Tyr kinases

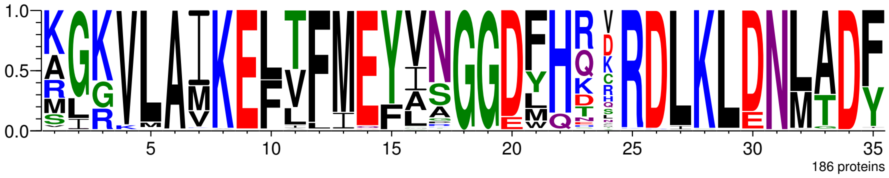

(c) Cluster III: 186 Ser/Thr kinases

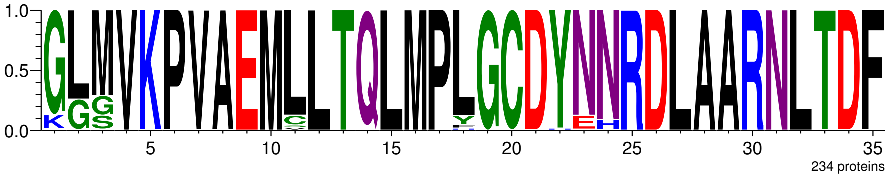

(d) Cluster IV: 234 EGFRs

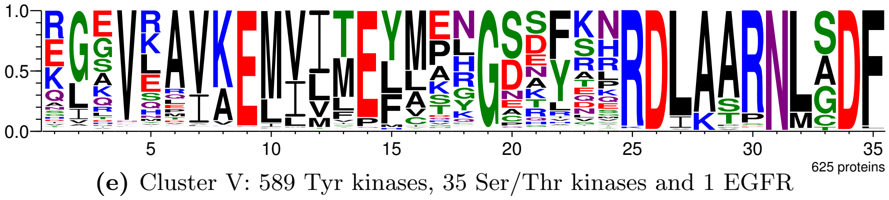

(e) Cluster V: 589 Tyr kinases, 35 Ser/Thr kinases and 1 EGFR

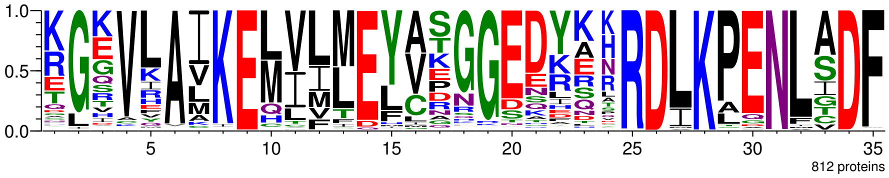

(f) Cluster VI: 807 Ser/Thr kinases and 5 Tyr kinases

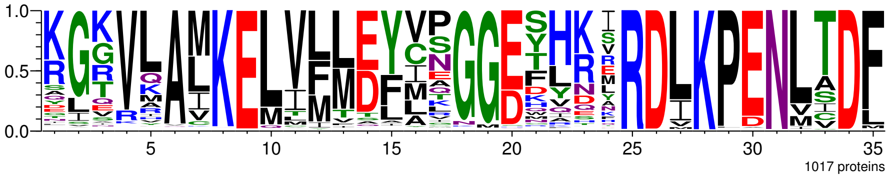

(g) Cluster VII: 1.016 Ser/Thr kinases and 1 Tyr kinase

Figure S7.2. Protein kinase division into seven clusters by the GP system.

Table S7.2. Most important residues for the seven protein kinase clusters produced by the GP system.

Cluster	Residues
**I**	$V13_{205}$, $N20_{212}$, $V12_{203}$, $K18_{210}$, $E21_{215}$, $P24_{220}$, $A16_{208}$
**II**	$\mathbf{R28_{254}}$, $\mathbf{A30_{256}}$, $I12_{203}$, $S20_{212}$, $M16_{208}$, $F22_{216}$, $T13_{205}$
**III**	$\mathbf{L29_{255}}$, $\mathbf{D30_{256}}$, $F12_{203}$, $D20_{212}$, $H22_{216}$, $N17_{209}$, $F10_{181}$
**IV**	$C20_{212}$, $P6_{156}$, $Q14_{206}$, $G1_{130}$, $L18_{210}$, $A8_{158}$, $M3_{133}$, $N23_{219}$, $K5_{142}$, $N24_{220}$
**V**	$\mathbf{R30_{256}}$, $\mathbf{A28_{254}}$, $\mathbf{A29_{255}}$, $V7_{157}$, $F22_{216}$, $I12_{203}$, $S20_{212}$
**VI**	$\mathbf{E30_{256}}$, $\mathbf{K28_{254}}$, $E20_{212}$, $\mathbf{P29_{255}}$, $D21_{215}$
**VII**	$\mathbf{P29_{255}}$, $L10_{181}$, $G18_{210}$, $\mathbf{E30_{256}}$, $\mathbf{K28_{254}}$, $D14_{206}$, $H22_{216}$, $T33_{269}$, $M7_{157}$

Listed in decreasing order of partial MI value. Residues in bold correspond to known SDPs. Subscripted positions correspond to those in PDB structure 1U46:A.

In Cluster V, whose majority is labeled as Tyr kinases, there are 35 Ser/Thr kinase-labeled proteins and one labeled as EGFR (Q2HZD7). The latter is unreviewed and automatically annotated with InterPro domain IPR016245 (*Tyr protein kinase, EGF/ERP/XmrK receptor*), i.e., despite being a Tyr kinase as all EGFRs are, it should have been clustered along with the other EGFRs. Among the 35 labeled as Ser/Thr kinases, two (Q54TM7 and A7J1T0) have been manually reviewed and annotated with InterPro domain IPR008271 (*serine/threonine-protein kinase, active site*), and also with GO term *protein serine/threonine kinase activity*. Only one of the other 33 unreviewed proteins, namely Q5AUJ7, lacks an annotation with the Ser/Thr kinase active site InterPro domain, yet is annotated with domain IPR002290 (*Ser/Thr/dual specificity protein kinase, catalytic domain*), which is present either in Ser/Thr kinases or dual specificity proteins. Such annotations indicate the GP system wrongly placed these 36 proteins in a Tyr kinase cluster.

In Cluster VI, of majority Ser/Thr kinases, there are five Tyr-kinase labeled proteins, all unreviewed. Two of them (Q69U56 and A2DGV6) are annotated with InterPro domain IPR008271 (*serine/threonine-protein kinase, active site*), while the other three (Q5RAR7, Q4T0K5, and A0JN96) are annotated with domain IPR002290 (*Ser/Thr/dual specificity protein kinase, catalytic domain*). Furthermore, among the five, only Q5RAR7 has not been annotated with GO term *protein serine/threonine kinase activity*. Hence, these annotations suggest these five subfamily labels are incorrect and that they were correctly placed into a Ser/Thr kinase cluster by the GP system. Lastly, Cluster VII shows a Tyr kinase-labeled protein among over a thousand Ser/Thr kinases: unreviewed protein Q6K3D4, which lacks subfamily-related annotations, so we cannot affirm if its label is incorrect or if the GP system clustered it improperly.

Despite wrongly positioning 36 proteins, which corresponds to less than 1.2% of the family, the clusters generated by the GP system were shown to be in much more agreement with the subfamily labels, as well as more contrasting to each other, than those produced by ASMC.

References

1. Melo-Minardi RC, Bastard K, Artiguenave F. Identification of subfamily-specific sites based on active sites modeling and clustering. Bioinformatics. 2010 Dec;26(24):3075–3082.
