# Supplementary material for: Isofunctional Protein Subfamily Detection Using Data Integration and Spectral Clustering
Source: PLoS Comput Biol. 2016 Jun 27;12(6):e1005001. doi: 10.1371/journal.pcbi.1005001 (PMC4922564; doi:10.1371/journal.pcbi.1005001)
Supplement: S8 Text — (PDF) [file pcbi.1005001.s008.pdf]

Isofunctional Protein Subfamily Detection using Data Integration and Spectral Clustering

Elisa Boari de Lima[1,2,*], Wagner Meira Júnior[2], Raquel Cardoso de Melo-Minardi[2]

1 Department of Biochemistry and Immunology, Federal University of Minas Gerais, Belo Horizonte, MG, Brazil
2 Department of Computer Science, Federal University of Minas Gerais, Belo Horizonte, MG, Brazil

*** eblima@dcc.ufmg.br**

S8 Text: Dividing the serine proteases into four clusters

The first level of ASMC's hierarchical clustering divided the family into four clusters, whose logos and compositions are presented in Fig. S8.1. One may observe that ASMC was only able to separate the elastases, while chymotrypsins and kallikreins, which represent even smaller percentages of the family, were mixed in one of the three trypsin clusters. This clustering has MI = 16.58.

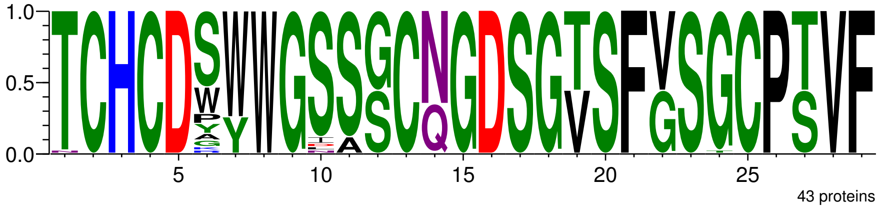

(a) Cluster I: 43 elastases

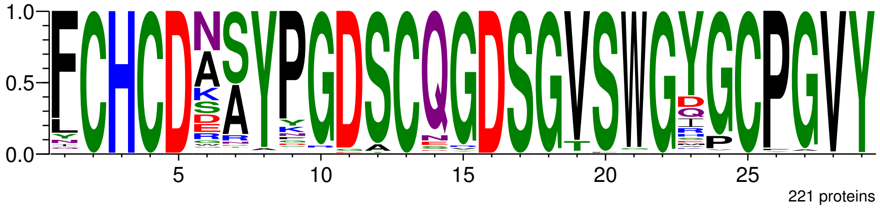

(b) Cluster II: 221 trypsins

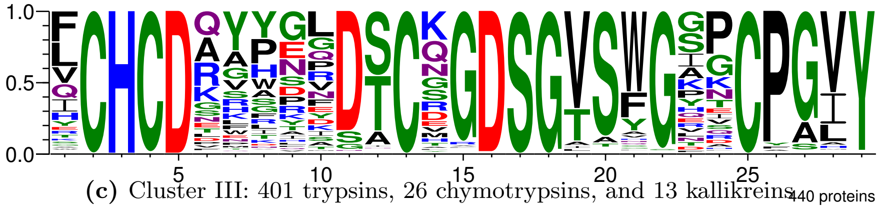

(c) Cluster III: 401 trypsins, 26 chymotrypsins, and 13 kallikreins

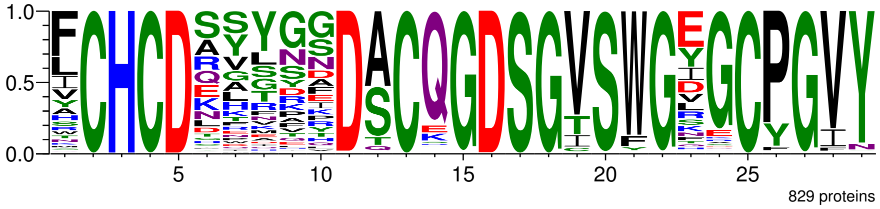

(d) Cluster IV: 829 trypsins

Figure S8.1. Serine protease division into four clusters in the first level of ASMC's hierarchical clustering.

When the GP system was run to separate the family into four clusters, the best result found presents MI = 17.71 and uses equation $ASid + ASscr + seqAliG$. Fig. S8.2 presents cluster logos and compositions. One may observe

that the GP system was also only able to separate the elastases. However, it was able to concentrate a larger amount of trypsins in the same cluster than ASMC did, while also placing chymotrypsins and kallikreins in different clusters, although such subfamilies are still mixed with trypsin subgroups, which dominate the family.

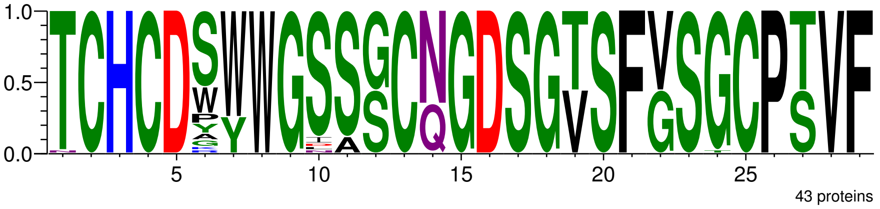

(a) Cluster I: 43 elastases

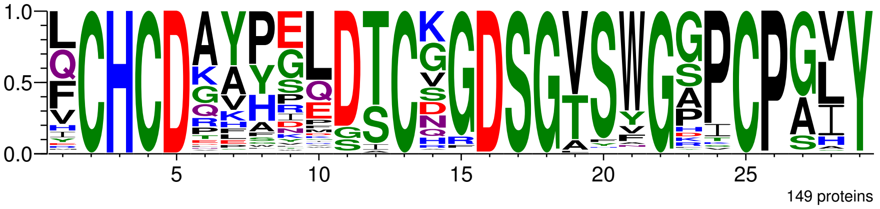

(b) Cluster II: 136 trypsins and 13 kallikreins

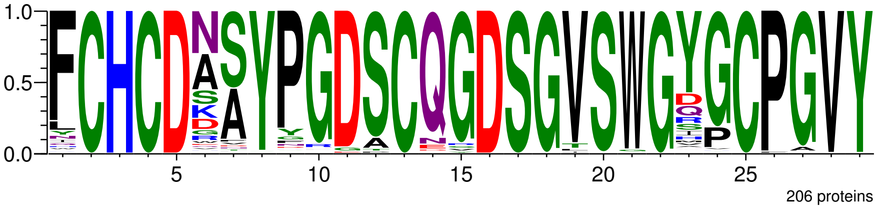

(c) Cluster III: 206 trypsins

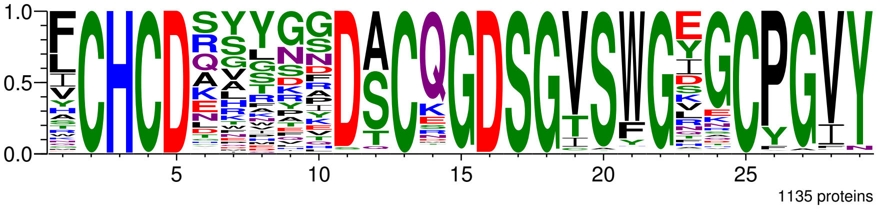

(d) Cluster IV: 1,109 trypsins and 26 chymotrypsins

Figure S8.2. Serine protease division into four clusters by the GP system.

The residues which most distinguish each cluster are listed in Table S8.1. The known SDPs for serine proteases are 172, 189, 216, and 226 considering chain A of PDB structure 5PTP [1], which correspond, respectively, to positions 8, 11, 22, and 27 on the presented active site. The Asp189 in trypsins, which justifies their preference for positive residues, is generally substituted by a Ser residue in elastases and chymotrypsins [1]. Additionally, Gly216 and Gly226 in trypsins and chymotrypsins are usually substituted in elastases by Val and Thr, which causes an active site occlusion that justifies their preference for small aliphatic residues [2]. Finally, Tyr172 in trypsins is substituted by Trp in chymotrypsins and elastases, which determines the conversion between trypsin and chymotrypsin [1]. One may observe that residues W8$_{172}$, S11$_{189}$, V22$_{216}$, and T27$_{226}$ are, in fact, among those considered by the GP system as most important to distinguish cluster I (elastases). Since the other clusters show a subfamily mixture, the residues most important to differentiate them do not correspond to the family's known SDPs.

Table S8.1. Most important residues for the four serine protease clusters produced by the GP system.

Cluster	Residues
**I**	F29$_{229}$, **W8$_{172}$**, T1$_{41}$, **S11$_{189}$**, S23$_{217}$, F21$_{215}$, W7$_{171}$, **V22$_{216}$**, S10$_{174}$, **T27$_{226}$**
**II**	P24$_{219}$, T12$_{190}$, L10$_{174}$, G23$_{217}$, P8$_{172}$, L28$_{227}$
**III**	P9$_{173}$, G10$_{174}$, **Y8$_{172}$**, Y23$_{217}$, F1$_{41}$, S7$_{171}$, S12$_{190}$
**IV**	A12$_{190}$, E23$_{217}$

Listed in decreasing order of partial MI value. Residues in bold correspond to known SDPs. Subscripted positions correspond to those in PDB structure 5PTP:A.

According to ASMC's criteria for considering as SDPs those positions with p-values smaller than 0.0001 [1], the cluster SDPs for ASMC's clustering are presented in Table S8.2. One may observe that known serine protease SDPs 8_{172}, 11_{189}, 22_{216}, and 27_{226} are among those considered as SDPs for cluster I (elastases), so both techniques were able to identify such positions for this subfamily, as well as position 8_{172} for their respective trypsin subclusters containing around 200 proteins. ASMC's p-value criteria also identified positions 11_{189} and 27_{226} as SDPs for cluster IV. However, one may easily note from comparing the cluster logos in Fig. S8.1 that such positions do not distinguish it from clusters II and III. This suggests ASMC's criteria should be revised. We also note that this criteria concerns positions, while our partial MI values are able to pinpoint the specific residues in such positions that are most important in distinguishing a cluster.

Table S8.2. Cluster SDPs for the four serine protease clusters produced by ASMC.

Cluster	Positions
**I**	1_{41}, $\mathbf{8_{172}}$, $\mathbf{11_{189}}$, 12_{190}, 14_{192}, 21_{215}, $\mathbf{22_{216}}$, $\mathbf{27_{226}}$, 29_{229}
**II**	$\mathbf{8_{172}}$, 9_{173}, 10_{174}, 12_{190}, 23_{217}
**III**	14_{192}, 21_{215}, 23_{217}, 24_{219}
**IV**	$\mathbf{11_{189}}$, 12_{190}, 14_{192}, 23_{217}, 24_{219}, $\mathbf{27_{226}}$

Listed in order of active site position. Residues in bold correspond to known SDPs. Subscripted positions correspond to those in PDB structure 5PTP:A.

Due to the immense disparity in the amount of trypsins relative to the other subfamilies, and since the considerable variability among trypsins hampers the identification of small subfamilies, a larger number of clusters is required by both techniques in order to isolate them in specific clusters.

References

1. Melo-Minardi RC, Bastard K, Artiguenave F. Identification of subfamily-specific sites based on active sites modeling and clustering. Bioinformatics. 2010 Dec;26(24):3075–3082.

2. Hannenhalli SS, Russell RB. Analysis and prediction of functional sub-types from protein sequence alignments. J Mol Biol. 2000 Oct;303:61–76.
