# Supplementary material for: Isofunctional Protein Subfamily Detection Using Data Integration and Spectral Clustering
Source: PLoS Comput Biol. 2016 Jun 27;12(6):e1005001. doi: 10.1371/journal.pcbi.1005001 (PMC4922564; doi:10.1371/journal.pcbi.1005001)
Supplement: S9 Text — (PDF) [file pcbi.1005001.s009.pdf]

Isofunctional Protein Subfamily Detection using Data Integration and Spectral Clustering

Elisa Boari de Lima[1,2,*], Wagner Meira Júnior[2], Raquel Cardoso de Melo-Minardi[2]

1 Department of Biochemistry and Immunology, Federal University of Minas Gerais, Belo Horizonte, MG, Brazil
2 Department of Computer Science, Federal University of Minas Gerais, Belo Horizonte, MG, Brazil

*** eblima@dcc.ufmg.br**

S9 Text: Dividing the serine proteases into eleven clusters

The second level of the hierarchical clustering produced by ASMC divided the serine proteases into eleven clusters, whose logos and compositions according to subfamily labels are presented in Fig. S9.1. This clustering has MI = 10.59.

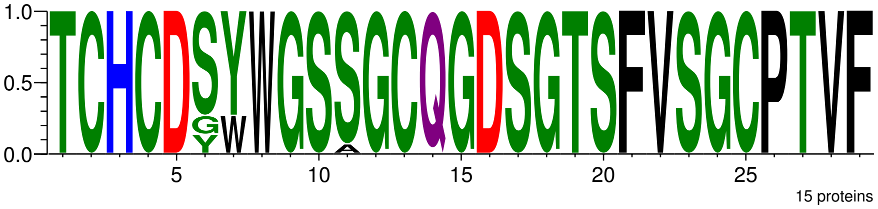

(a) Cluster I.A: 15 elastases

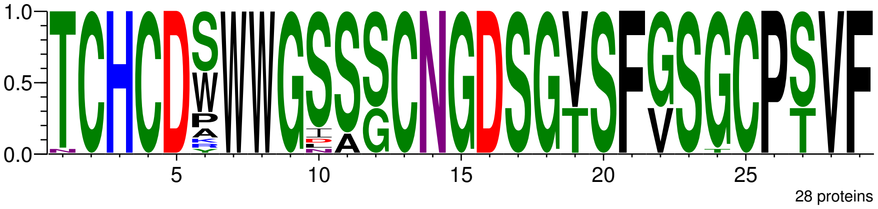

(b) Cluster I.B: 28 elastases

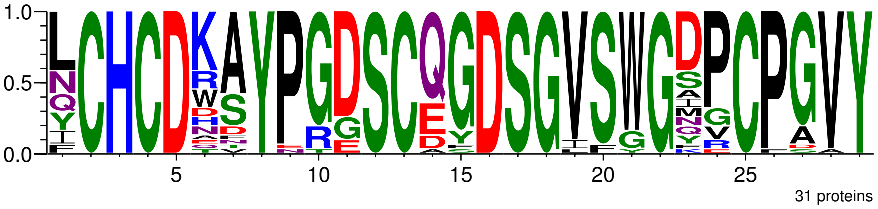

(c) Cluster II.A: 31 trypsins

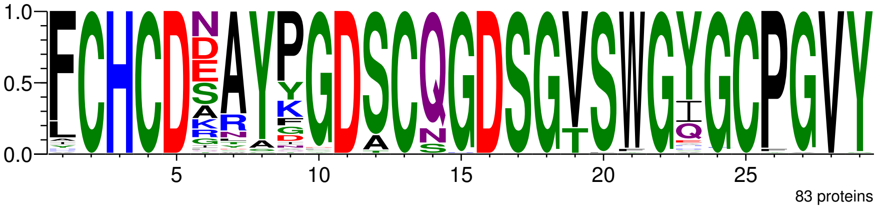

(d) Cluster II.B: 83 trypsins

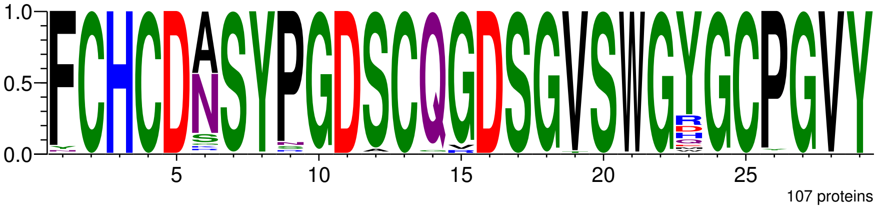

(e) Cluster II.C: 107 trypsins

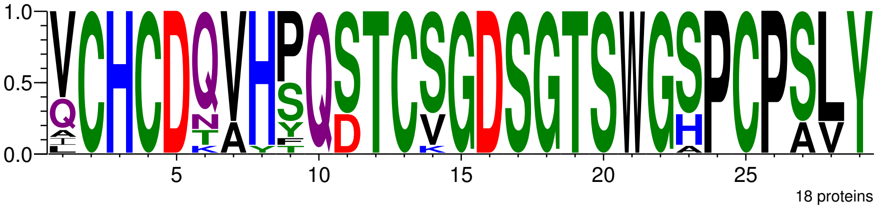

(f) Cluster III.A: 13 kallikreins and 5 trypsins

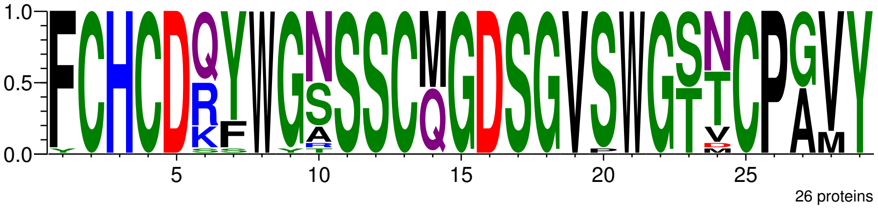

(g) Cluster III.B: 26 chymotrypsins

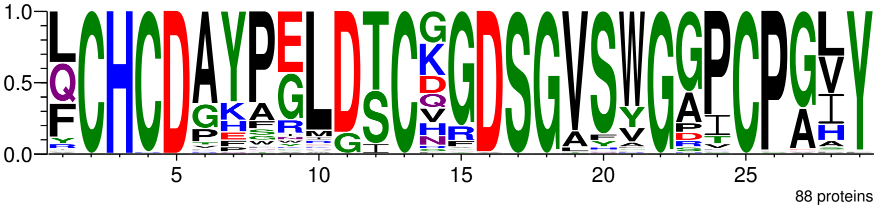

(h) Cluster III.C: 88 trypsins

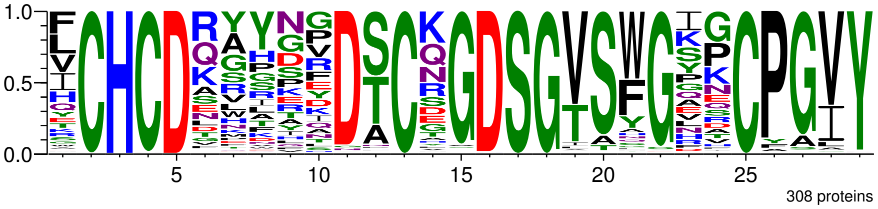

(i) Cluster III.D: 308 trypsins

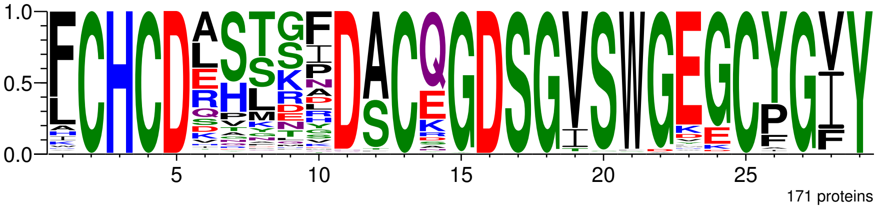

(j) Cluster IV.A: 171 trypsins

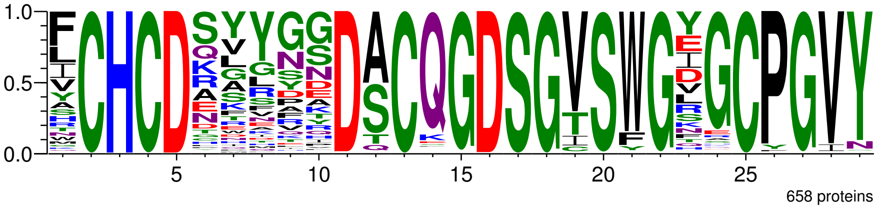

(k) Cluster IV.B: 658 trypsins

Figure S9.1. Serine protease division into eleven clusters in the second level of ASMC's hierarchical clustering.

Given ASMC's criteria for considering as specificity determining positions (SDPs) those positions with p-values smaller than 0.0001 [1], the SDPs per cluster for this clustering are presented in Table S9.1, in which one may notice the presence of the known SDPs for this family, which were listed in the S8 Text.

Table S9.1. Cluster SDPs for the eleven serine protease clusters produced by ASMC.

Cluster	Positions
**I.A**	$\mathbf{11_{189}}$, $\mathbf{22_{216}}$, $\mathbf{27_{226}}$, 29_{173}
**I.B**	$\mathbf{8_{172}}$, $\mathbf{11_{189}}$, 14_{192}, $\mathbf{27_{226}}$, 29_{173}
**II.A**	-
**II.B**	-
**II.C**	7_{171}, 9_{173}
**III.A**	$\mathbf{11_{189}}$, $\mathbf{27_{226}}$, 28_{227}
**III.B**	$\mathbf{11_{189}}$, 14_{192}
**III.C**	$\mathbf{8_{172}}$, 10_{174}, 24_{219}, 28_{227}
**III.D**	21_{215}, 24_{219}
**IV.A**	23_{217}, 26_{225}, 28_{227}
**IV.B**	12_{190}, 14_{192}, 28_{227}

Listed in order of active site position. Positions in bold correspond to known SDPs. Subscripted positions correspond to those in PDB structure 5PTP:A.

The best result obtained by the GP system for eleven clusters has MI = 12.09 and uses a combination of three data types, as shown in the main text. Cluster logos and compositions are presented in Fig. S9.2, while the residues which most distinguish each cluster are listed in S9.2, in which one can note the presence of the known SDPs for the cases in which the residues occurring in such positions distinguish the corresponding cluster from the others.

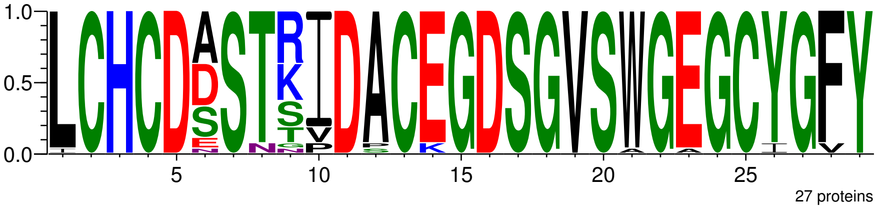

(a) Cluster I: 27 trypsins

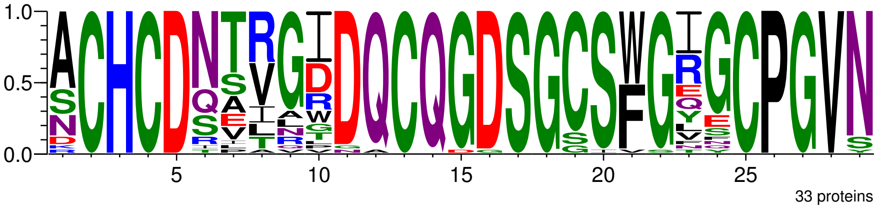

(b) Cluster II: 33 trypsins

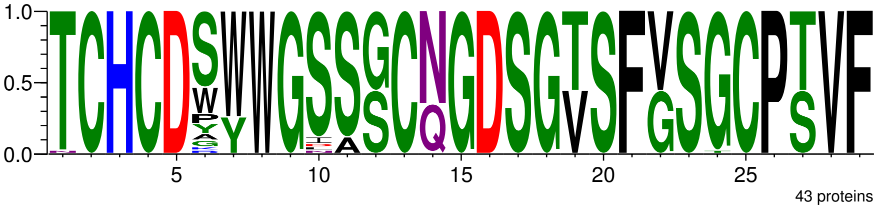

(c) Cluster III: 43 elastases

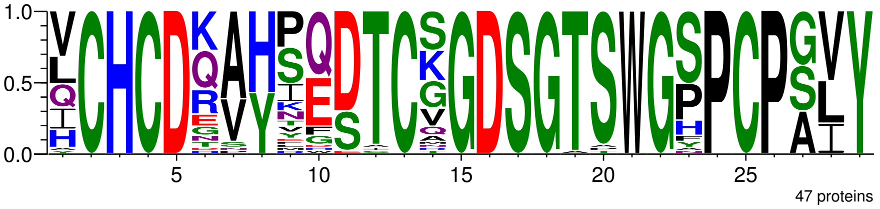

(d) Cluster IV: 34 trypsins and 13 kallikreins

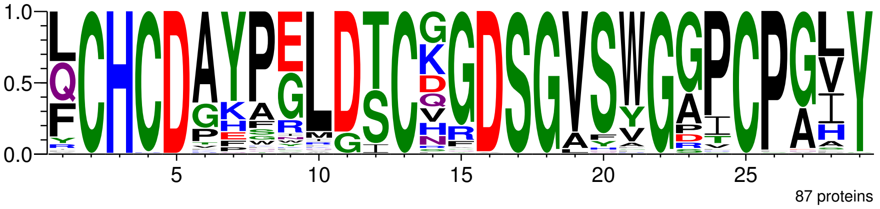

(e) Cluster V: 87 trypsins

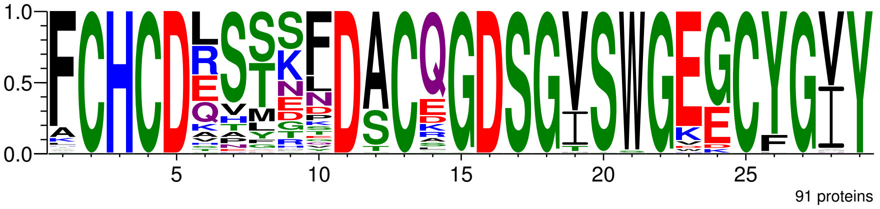

(f) Cluster VI: 91 trypsins

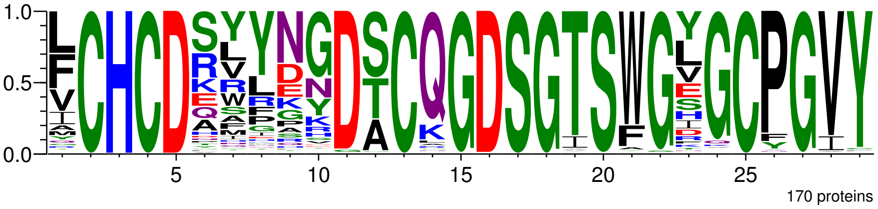

(g) Cluster VII: 170 trypsins

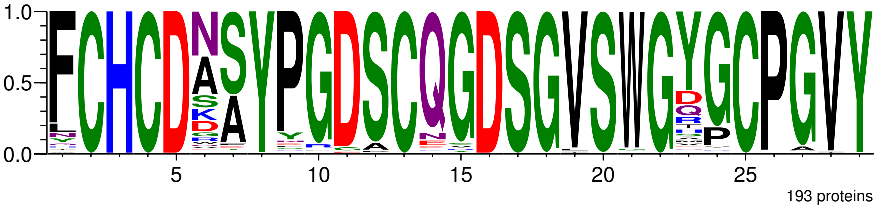

(h) Cluster VIII: 193 trypsins

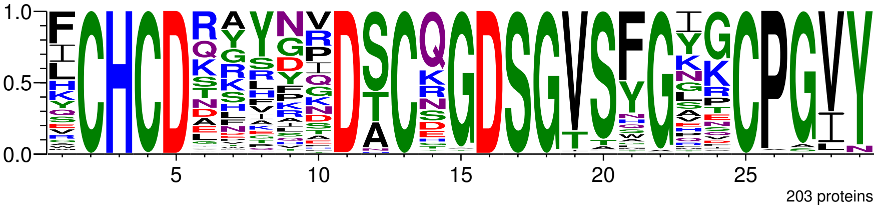

(i) Cluster IX: 203 trypsins

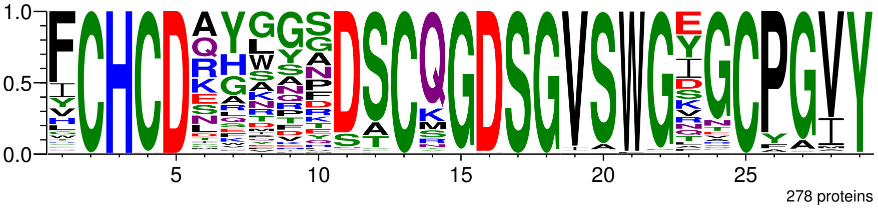

(j) Cluster X: 252 trypsins and 26 chymotrypsins

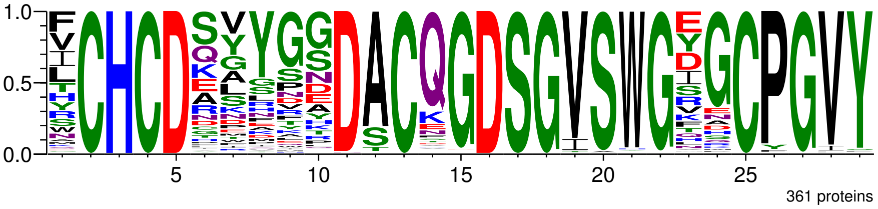

(k) Cluster XI: 361 trypsins

Figure S9.2. Serine protease division into eleven clusters by the GP system.

Table S9.2. Most important residues for the eleven serine protease clusters produced by the GP system.

Cluster	Residues
**I**	$F28_{227}$, $E14_{192}$, $\mathbf{T8_{172}}$, $I10_{174}$, $Y26_{225}$, $L1_{41}$, $E23_{217}$, $\mathbf{S8_{172}}$
**II**	$Q12_{190}$, $C19_{213}$, $N29_{229}$, $A1_{41}$
**III**	$F29_{229}$, $\mathbf{W8_{172}}$, $T1_{41}$, $\mathbf{S11_{189}}$, $F21_{215}$, $W7_{171}$, $\mathbf{V22_{216}}$, $S10_{174}$, $\mathbf{T27_{226}}$
**IV**	$P24_{219}$, $T19_{213}$, $T12_{190}$, $\mathbf{H8_{172}}$
**V**	$L10_{174}$, $\mathbf{P8_{172}}$, $P24_{219}$, $G23_{217}$, $A6_{170}$, $E9_{173}$, $Y7_{171}$
**VI**	$Y26_{225}$, $E23_{217}$, $F10_{174}$, $S8_{171}$
**VII**	$T19_{213}$, $N9_{173}$
**VIII**	$P9_{173}$, $G10_{174}$, $\mathbf{Y8_{172}}$, $S8_{171}$, $F1_{41}$, $S12_{190}$, $Y23_{217}$
**IX**	$F21_{215}$, $Y21_{215}$
**X**	$S12_{190}$, $V19_{213}$, $W21_{215}$
**XI**	$A12_{190}$, $W21_{215}$, $G9_{173}$

Listed in decreasing order of partial MI value. Residues in bold correspond to known SDPs. Subscripted positions correspond to those in PDB structure 5PTP:A.

References

1. Melo-Minardi RC, Bastard K, Artiguenave F. Identification of subfamily-specific sites based on active sites modeling and clustering. Bioinformatics. 2010 Dec;26(24):3075–3082.
