# Supplementary material for: Isofunctional Protein Subfamily Detection Using Data Integration and Spectral Clustering
Source: PLoS Comput Biol. 2016 Jun 27;12(6):e1005001. doi: 10.1371/journal.pcbi.1005001 (PMC4922564; doi:10.1371/journal.pcbi.1005001)
Supplement: S10 Text — (PDF) [file pcbi.1005001.s010.pdf]

Isofunctional Protein Subfamily Detection using Data Integration and Spectral Clustering

Elisa Boari de Lima[1,2,*], Wagner Meira Júnior[2], Raquel Cardoso de Melo-Minardi[2]

1 Department of Biochemistry and Immunology, Federal University of Minas Gerais, Belo Horizonte, MG, Brazil
2 Department of Computer Science, Federal University of Minas Gerais, Belo Horizonte, MG, Brazil

*** eblima@dcc.ufmg.br**

S10 Text: Dividing the serine proteases into twelve clusters

The best result found by the genetic programming (GP) system for dividing the serine proteases into twelve clusters is obtained using equation $3ASid + 2ASscr + go$. Cluster logos and compositions according to subfamily labels are presented in Fig. S10.1. One may observe that a single elastase cluster is maintained, along with the interesting kallikrein and prothrombin clusters mentioned in the paper. Additionally, the GP system was able to isolate the chymotrypsins in their own cluster. The residues most important to distinguish each cluster are listed in Table S10.1, in which we note the presence of the known SDPs for the family, listed in the S8 Text, for the cases in which the residues occurring in such positions distinguish the corresponding cluster from the others.

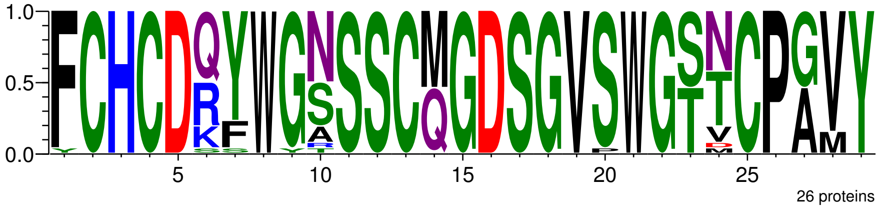

(a) Cluster I: 26 chymotrypsins

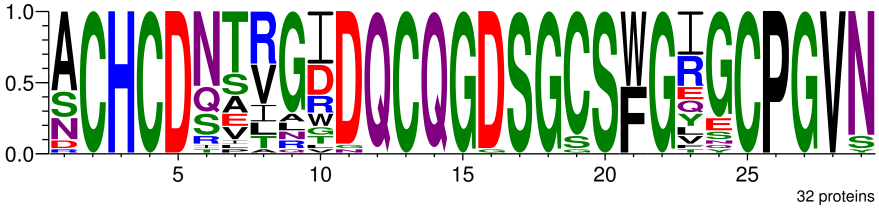

(b) Cluster II: 32 trypsins

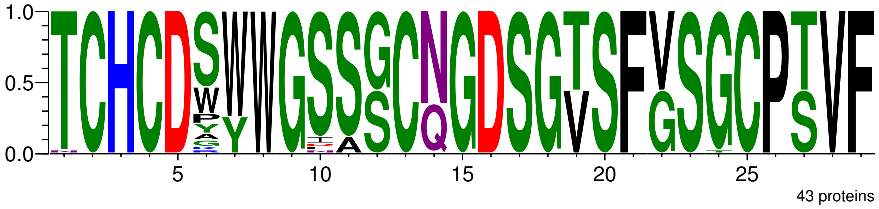

(c) Cluster III: 43 elastases

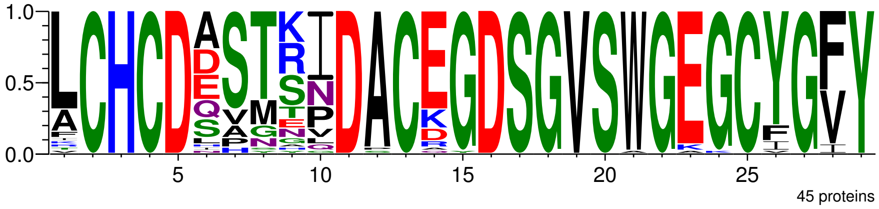

(d) Cluster IV: 45 trypsins

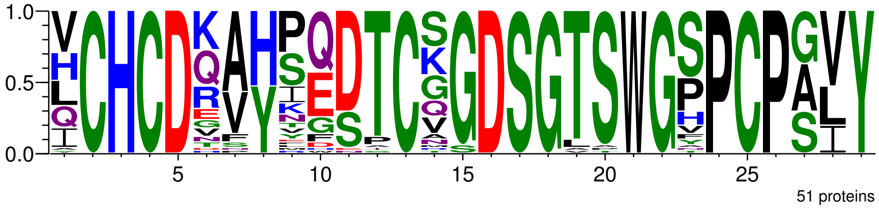

(e) Cluster V: 38 trypsins and 13 kallikreins

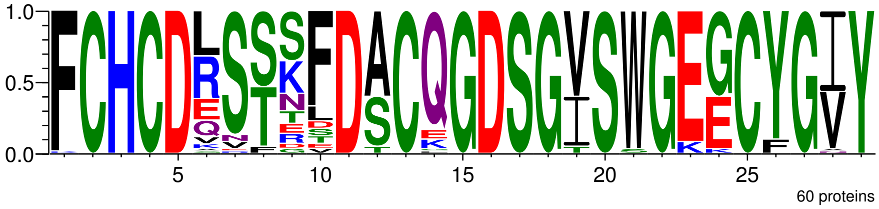

(f) Cluster VI: 60 trypsins

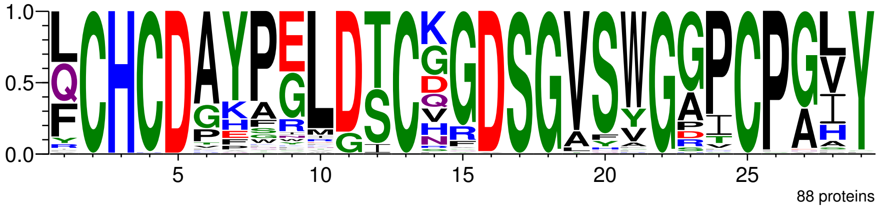

(g) Cluster VII: 88 trypsins

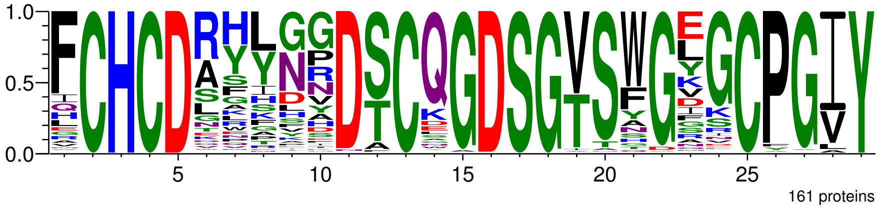

(h) Cluster VIII: 161 trypsins

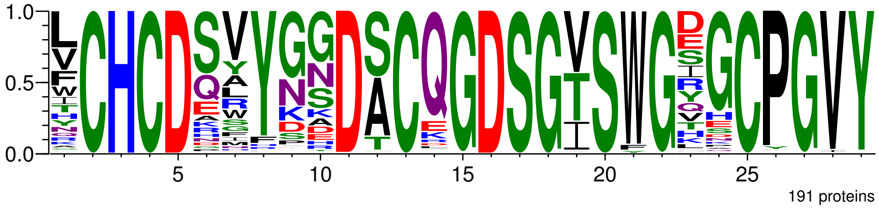

(i) Cluster IX: 191 trypsins

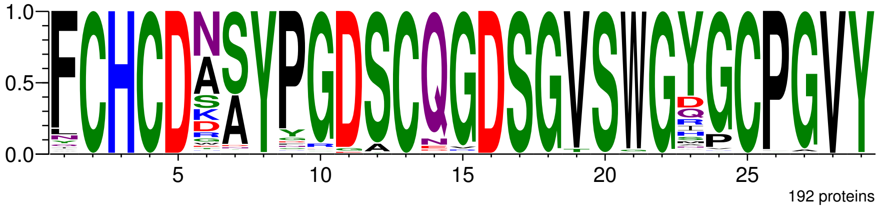

(j) Cluster X: 192 trypsins

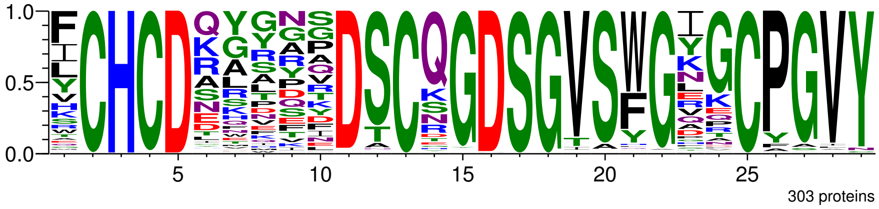

(k) Cluster XI: 303 trypsins

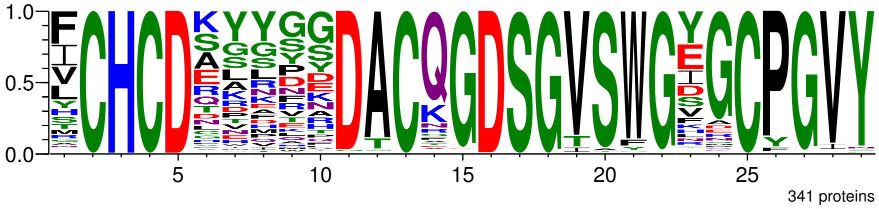

(l) Cluster XII: 341 trypsins

Figure S10.1. Serine protease division into twelve clusters by the GP system.

Table S10.1. Most important residues for the twelve serine protease clusters produced by the GP system.

Cluster	Residues
**I**	**S11$_{189}$**, **W8$_{172}$**, M14$_{192}$, G9$_{173}$
**II**	Q12$_{190}$, C19$_{213}$, N29$_{229}$, A1$_{41}$
**III**	F29$_{229}$, **W8$_{172}$**, T1$_{41}$, **S11$_{189}$**, S23$_{217}$, F21$_{215}$, W7$_{171}$, **V22$_{216}$**, S10$_{174}$, **T27$_{226}$**
**IV**	F28$_{227}$, E14$_{192}$, E23$_{217}$, Y26$_{225}$, **T8$_{172}$**
**V**	P24$_{219}$, T12$_{190}$, T19$_{213}$, **H8$_{172}$**
**VI**	Y26$_{225}$, E23$_{217}$, F10$_{174}$, S7$_{171}$, F1$_{41}$, **S9$_{172}$**
**VII**	L10$_{174}$, **P8$_{172}$**, P24$_{219}$, G23$_{217}$, A6$_{170}$, E9$_{173}$, Y7$_{171}$
**VIII**	I28$_{227}$, **L8$_{172}$**
**IX**	**Y8$_{172}$**, V7$_{171}$, S6$_{170}$
**X**	P9$_{173}$, G10$_{174}$, **Y8$_{172}$**, F1$_{41}$, Y23$_{217}$, S7$_{171}$, S12$_{190}$
**XI**	S12$_{190}$, F21$_{215}$
**XII**	A12$_{190}$

Listed in decreasing order of partial MI value. Residues in bold correspond to known SDPs. Subscripted positions correspond to those in PDB structure 5PTP:A.
