# Supplementary material for: Isofunctional Protein Subfamily Detection Using Data Integration and Spectral Clustering
Source: PLoS Comput Biol. 2016 Jun 27;12(6):e1005001. doi: 10.1371/journal.pcbi.1005001 (PMC4922564; doi:10.1371/journal.pcbi.1005001)
Supplement: S11 Text — (PDF) [file pcbi.1005001.s011.pdf]

Isofunctional Protein Subfamily Detection using Data Integration and Spectral Clustering

Elisa Boari de Lima[1,2,*], Wagner Meira Júnior[2], Raquel Cardoso de Melo-Minardi[2]

1 Department of Biochemistry and Immunology, Federal University of Minas Gerais, Belo Horizonte, MG, Brazil
2 Department of Computer Science, Federal University of Minas Gerais, Belo Horizonte, MG, Brazil

*** eblima@dcc.ufmg.br**

S11 Text: Dividing the crotonase superfamily into twelve clusters

The best result found by the genetic programming (GP) system for dividing the crotonases into twelve clusters is obtained using equation $ASid + seqAliG + strAliId$. Cluster logos and compositions according to the SFLD [1] family labels are presented in Fig. S11.1. The residues most important to distinguish each cluster are listed in Table S11.1.

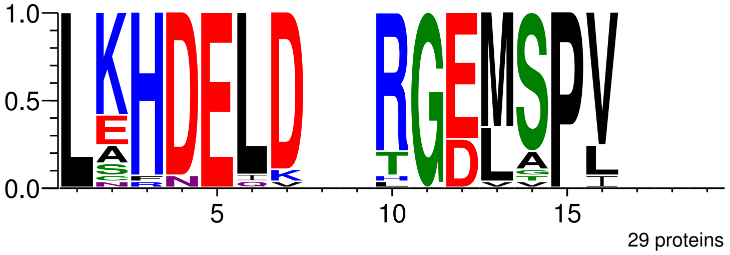

(a) Cluster I: 29 methylmalonyl-CoA decarboxylases

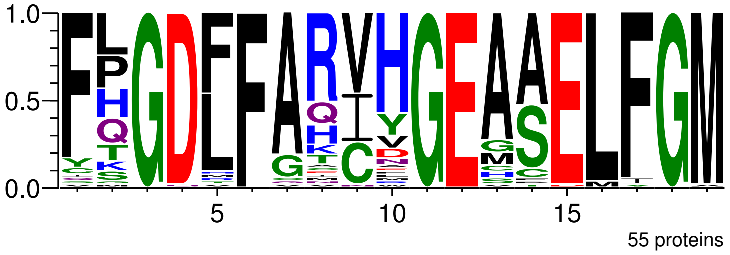

(b) Cluster II: 55 diffusible signal factor (DSF) synthases

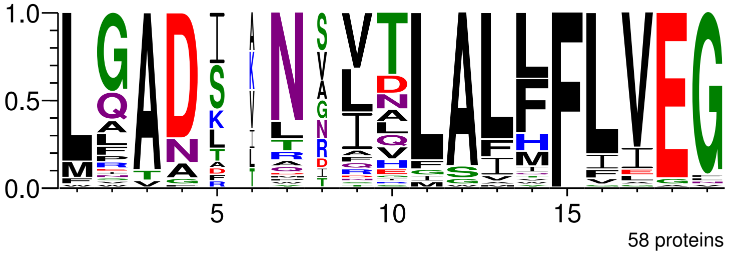

(c) Cluster III: 58 dodecenoyl-CoA delta-isomerases (peroxisomal)

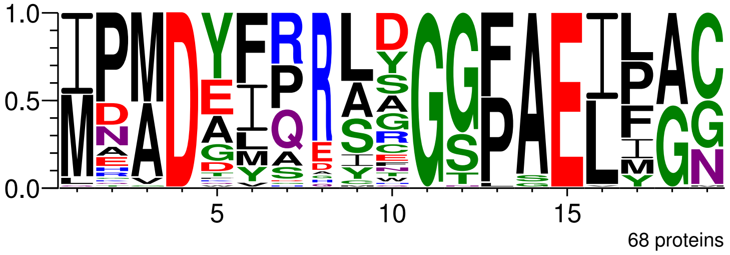

(d) Cluster IV: 35 polyketide biosynthesis enoyl-CoA hydratases and 33 feruloyl-CoA hydratase/lyases

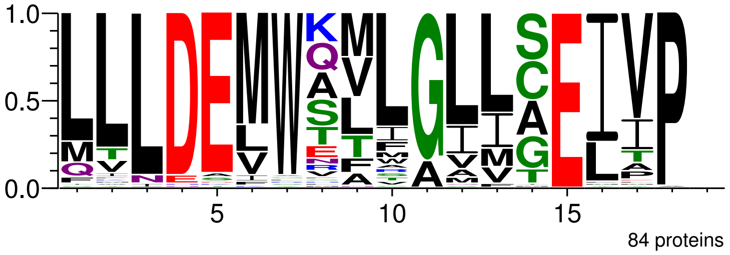

(e) Cluster V: 84 dodecenoyl-CoA delta-isomerases (mitochondrial)

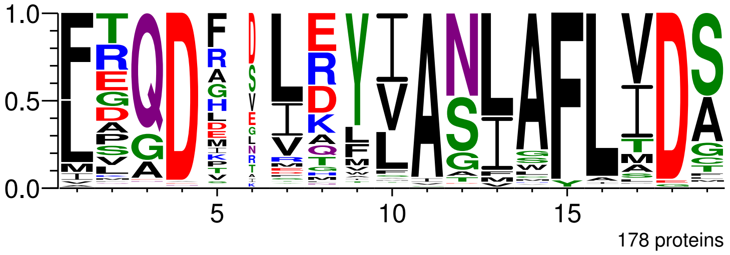

(f) Cluster VI: 143 1,2-epoxyphenylacetyl-CoA isomerases, 34 enoyl-CoA hydratases, and 1 dodecenoyl-CoA delta-isomerase (peroxisomal)

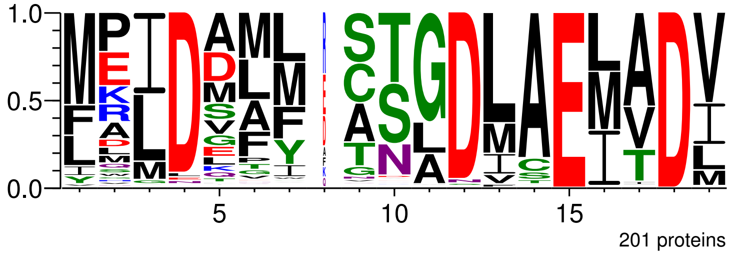

(g) Cluster VII: 201 delta(3,5)-delta(2,4)-dienoyl-CoA isomerases

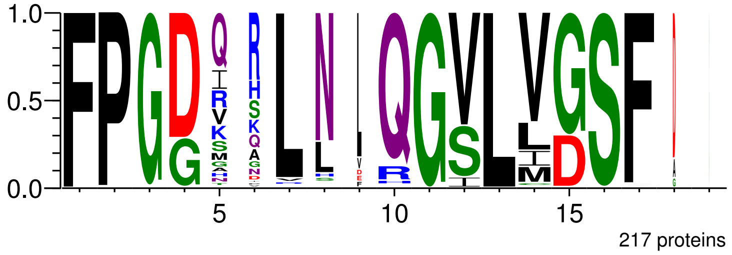

(h) Cluster VIII: 217 1,4-dihydroxy-2-napthoyl-CoA synthases

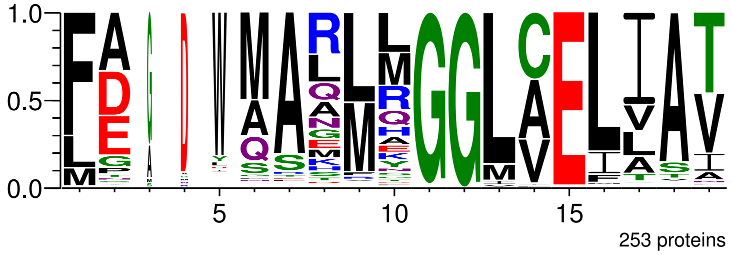

(i) Cluster IX: 252 methylglutaconyl-CoA hydratases 2 and 1 polyketide biosynthesis enoyl-CoA hydratase

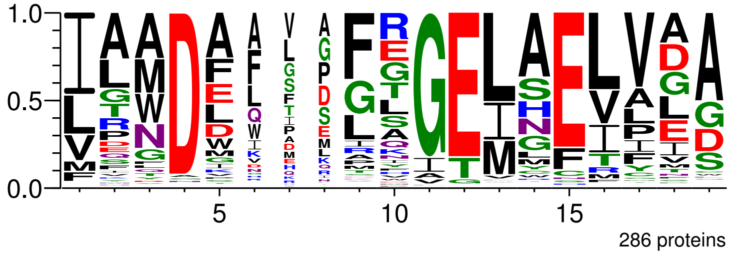

(j) Cluster X: 227 enoyl-CoA hydratases, 47 crotonobetainyl-CoA hydratases, 4 polyketide biosynthesis enoyl-CoA hydratases , 3 dodecenoyl-CoA delta-isomerases (peroxisomal), 3 methylglutaconyl-CoA hydratase 2, 1 dodecenoyl-CoA delta-isomerase (mitochondrial), and 1 methylmalonyl-CoA decarboxylase

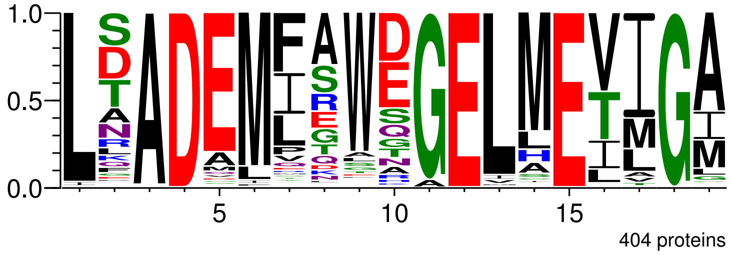

(k) Cluster XI: 404 enoyl-CoA hydratases

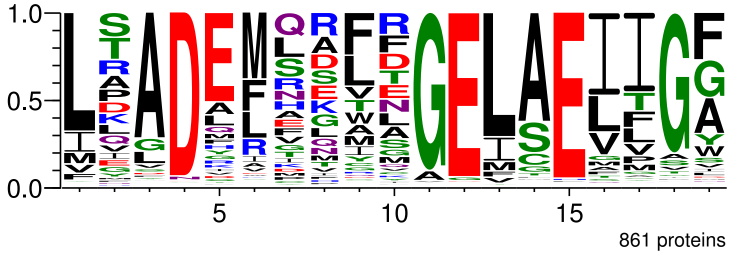

(l) Cluster XII: 842 enoyl-CoA hydratases, 14 methylglutaconyl-CoA hydratase 2, 3 dodecenoyl-CoA delta-isomerases (peroxisomal), and 2 dodecenoyl-CoA delta-isomerases (mitochondrial)

Figure S11.1. Crotonase superfamily division into twelve clusters by the GP system.

Table S11.1. Most important residues for the twelve crotonase superfamily clusters produced by the GP system.

Cluster	Residues
**I**	$\mathbf{P15_{164}}$, $\mathbf{H3_{98}}$, $D7_{117}$, $L6_{113}$
**II**	$M19_{173}$, $A13_{145}$, $F6_{113}$, $\mathbf{G3_{98}}$, $F17_{170}$, $H10_{121}$, $A7_{117}$
**III**	$\mathbf{E18_{172}}$, $A12_{144}$, $\mathbf{L11_{141}}$, $\mathbf{F15_{164}}$, $G19_{173}$, $N7_{117}$
**IV**	$P13_{145}$, $C19_{173}$, $F13_{145}$, $\mathbf{M3_{98}}$
**V**	$\mathbf{P18_{172}}$, $W7_{117}$, $L12_{144}$, $\mathbf{L3_{98}}$, $L2_{63}$, $L10_{121}$
**VI**	$\mathbf{F15_{164}}$, $\mathbf{A11_{141}}$, $\mathbf{Q3_{98}}$, $\mathbf{D18_{172}}$, $N12_{144}$, $Y9_{120}$, $S19_{173}$, $L16_{169}$, $I10_{121}$
**VII**	$D12_{144}$, $\mathbf{D18_{172}}$, $\mathbf{I3_{98}}$, $A17_{170}$, $V19_{173}$, $M1_{61}$, $M16_{169}$
**VIII**	$S16_{169}$, $P2_{63}$, $Q10_{121}$, $\mathbf{G15_{164}}$, $F17_{170}$, $V12_{144}$, $F1_{61}$, $V14_{148}$, $\mathbf{G3_{98}}$, $L7_{117}$, $\mathbf{N8_{118}}$, $\mathbf{D15_{164}}$
**IX**	$G12_{144}$, $A7_{117}$, $\mathbf{A18_{172}}$, $T19_{173}$, $W5_{102}$, $F1_{61}$, $L16_{169}$, $M9_{120}$, $L9_{120}$, $C14_{148}$
**X**	$I1_{61}$
**XI**	$W9_{120}$, $M14_{148}$, $M6_{113}$, $\mathbf{A3_{98}}$, $\mathbf{G18_{172}}$, $E5_{102}$, $L1_{61}$, $E12_{144}$, $A19_{173}$, $V16_{169}$, $F7_{117}$, $T16_{169}$
**XII**	$E12_{144}$, $\mathbf{G18_{172}}$, $\mathbf{A3_{98}}$, $F19_{173}$, $I16_{169}$, $A14_{148}$

Listed in decreasing order of partial MI value. Subscripted positions correspond to those in PDB structure 1MJ3:A. Residues in bold are in known catalytic residue positions FOR 1MJ3:A.

References

1. Akiva E, Brown S, Almonacid DE, Barber 2nd AE, Custer AF, Hicks MA, et al. The Structure-Function Linkage Database. Nucl Acids Res. 2014 Jan;42(D1):D521–30.
