# Supplementary material for: Isofunctional Protein Subfamily Detection Using Data Integration and Spectral Clustering
Source: PLoS Comput Biol. 2016 Jun 27;12(6):e1005001. doi: 10.1371/journal.pcbi.1005001 (PMC4922564; doi:10.1371/journal.pcbi.1005001)
Supplement: S12 Text — (PDF) [file pcbi.1005001.s012.pdf]

Isofunctional Protein Subfamily Detection using Data Integration and Spectral Clustering

Elisa Boari de Lima[1,2,*], Wagner Meira Júnior[2], Raquel Cardoso de Melo-Minardi[2]

1 Department of Biochemistry and Immunology, Federal University of Minas Gerais, Belo Horizonte, MG, Brazil
2 Department of Computer Science, Federal University of Minas Gerais, Belo Horizonte, MG, Brazil

*** eblima@dcc.ufmg.br**

S12 Text: Dividing the enolase superfamily into twelve clusters

The best result found by the genetic programming (GP) system for dividing the enolases into twelve clusters is obtained using equation $3APid + go + 2seqAliG$. Cluster logos and compositions according to the SFLD [1] family labels are presented in Fig. S12.1. The residues most important to distinguish each cluster are listed in Table S12.1.

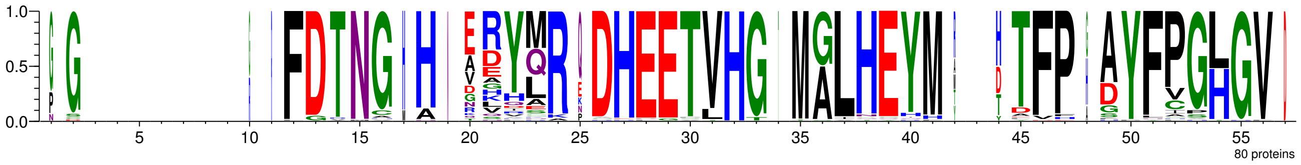

(a) Cluster I: 80 mannonate dehydratases

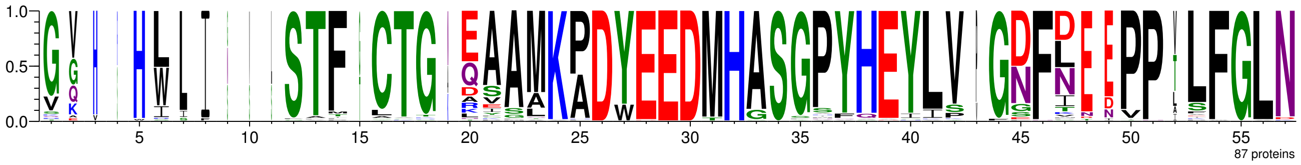

(b) Cluster II: 87 rhamnonate dehydratases

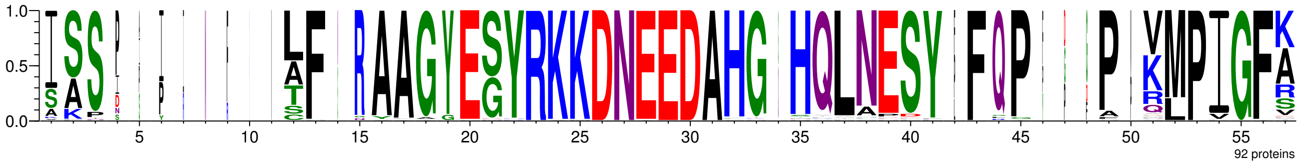

(c) Cluster III: 92 D-tartrate dehydratases

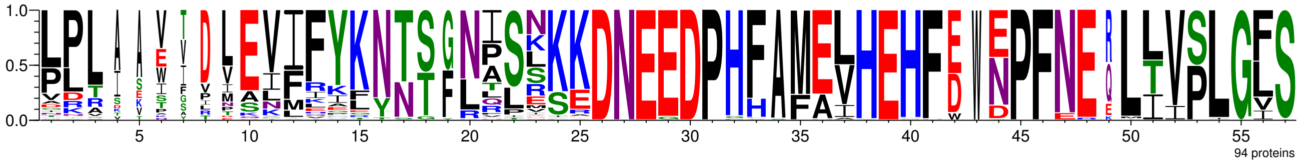

(d) Cluster IV: 94 L-talarate/galactarate dehydratases

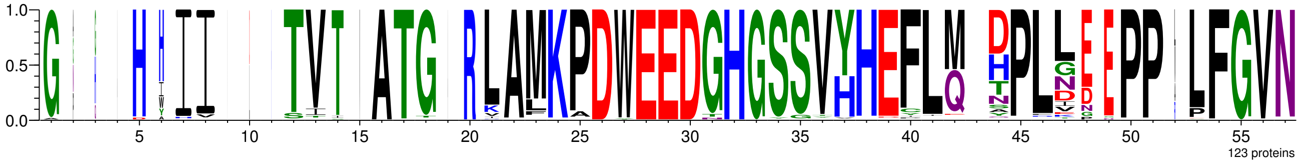

(e) Cluster V: 123 rhamnonate dehydratases

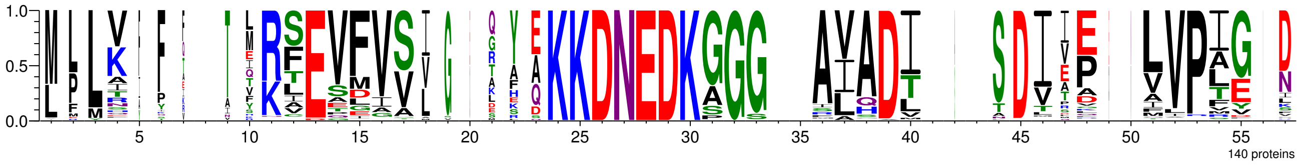

(f) Cluster VI: 140 o-succinylbenzoate synthases

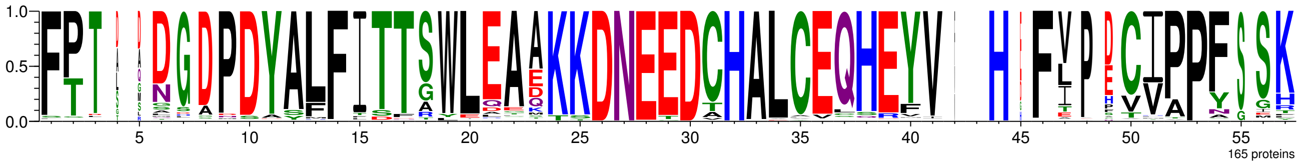

(g) Cluster VII: 165 L-fuconate dehydratases

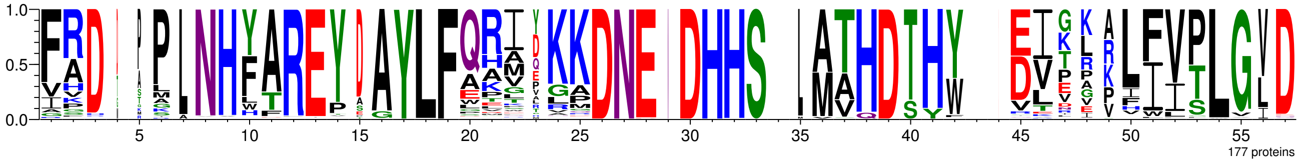

(h) Cluster VIII: 177 glucarate dehydratases

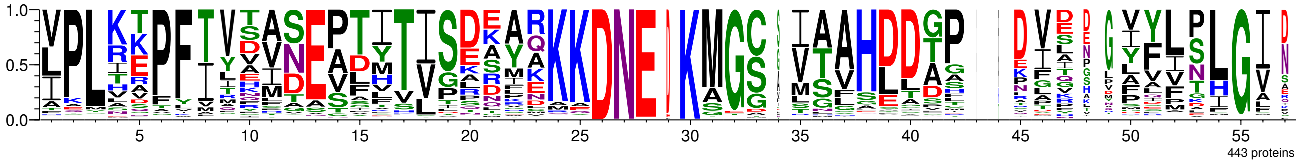

(i) Cluster IX: 363 dipeptide epimerases, 61 N-succinylamino acid racemase 2_{17}, and 19 o-succinylbenzoate synthases

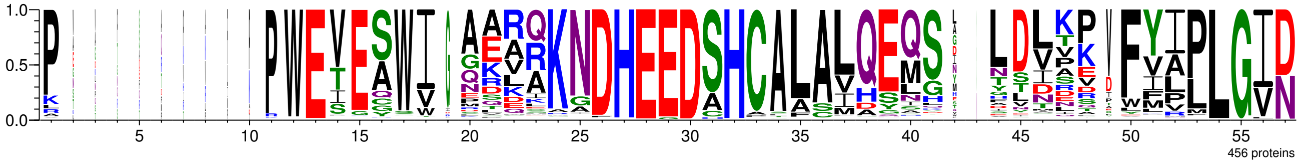

(j) Cluster X: 456 D-galactonate dehydratases

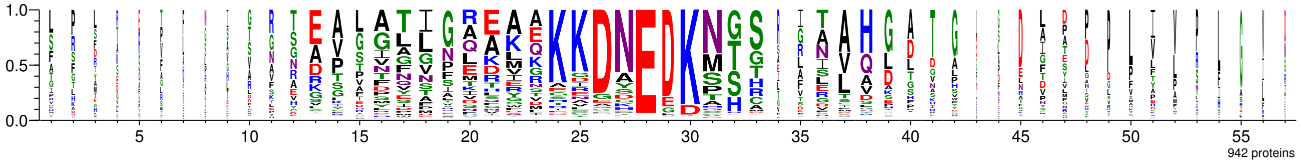

(k) Cluster XI: 513 enolases, 204 o-succinylbenzoate synthases, 82 dipeptide epimerases, 57 methylaspartate ammonia-lyases, 18 L-fuconate dehydratases, 17 D-galactonate dehydratases, 16 glucarate dehydratases, 12 rhamnonate dehydratases, 9 N-succinylamino acid racemase 2_{17}, 6 D-tartrate dehydratases, 4 L-talarate/galactarate dehydratases, and 4 mannonate dehydratases

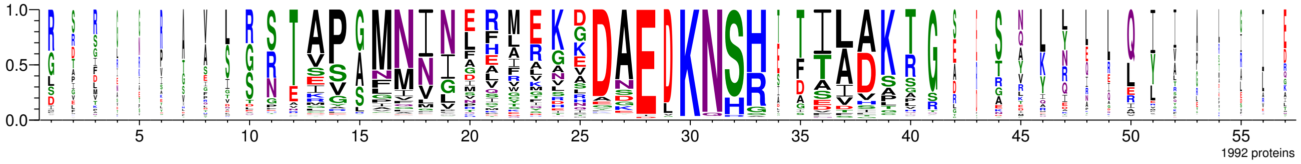

(l) Cluster XII: 1979 enolases, 7 o-succinylbenzoate synthases, 3 dipeptide epimerases, 2 rhamnonate dehydratases, and 1 D-galactonate dehydratase

Figure S12.1. Enolase superfamily division into twelve clusters by the GP system.

Table S12.1. Most important residues for the twelve enolase clusters produced by the GP system.

Cluster	Residues
**I**	$\mathbf{T30_{270}}$, $M41_{319}$, $N15_{54}$, $H18_{140}$, $F12_{34}$, $Y50_{330}$, $V31_{272}$, $G53_{340}$, $D13_{50}$, $R24_{164}$, $P47_{327}$
**II**	$T13_{50}$, $P36_{301}$, $G44_{323}$, $G35_{300}$, $L56_{347}$, $P51_{332}$, $C16_{138}$, $Y37_{304}$, $V42_{320}$, $Y27_{197}$
**III**	$Q36_{301}$, $H35_{300}$, $F43_{321}$, $Y41_{319}$, $F56_{347}$, $N38_{305}$, $A17_{139}$, $F13_{50}$, $I54_{345}$, $A31_{272}$, $M52_{339}$, $S40_{318}$, $P49_{329}$, $Y22_{155}$
**IV**	$F41_{319}$, $S57_{350}$, $H40_{318}$, $P31_{272}$, $F33_{298}$, $N47_{327}$, $P45_{324}$, $K15_{54}$
**V**	$W27_{197}$, $S35_{300}$, $V36_{301}$, $F40_{318}$, $P51_{332}$, $P50_{330}$, $\mathbf{P25_{166}}$, $L41_{319}$, $G31_{272}$, $S34_{299}$, $P45_{324}$, $F54_{345}$, $I7_{22}$, $G18_{140}$, $V56_{347}$, $I8_{24}$, $V13_{50}$, $N57_{350}$, $M23_{158}$
**VI**	$V16_{138}$, $\mathbf{D39_{317}}$, $\mathbf{G32_{297}}$, $V52_{339}$
**VII**	$C35_{300}$, $W19_{141}$, $Y11_{32}$, $L34_{299}$, $Q37_{304}$, $V41_{319}$, $F14_{52}$, $H44_{323}$, $P9_{29}$, $T16_{138}$, $D10_{31}$, $A33_{298}$, $A12_{34}$, $C50_{330}$, $C31_{272}$, $F1_{16}$, $L13_{50}$, $E36_{301}$, $F46_{326}$, $Y40_{318}$, $P52_{339}$, $L20_{151}$, $K57_{350}$, $D6_{21}$, $S56_{347}$, $I15_{54}$, $T3_{18}$, $P53_{340}$
**VIII**	$H31_{272}$, $Y17_{139}$, $N8_{24}$, $R12_{34}$, $F19_{141}$, $H9_{29}$, $H41_{319}$, $L18_{140}$, $D3_{18}$, $\mathbf{D39_{317}}$, $S33_{298}$, $A11_{32}$, $D57_{350}$, $A16_{138}$, $Y14_{52}$
**IX**	$L3_{18}$, $\mathbf{G32_{297}}$, $F7_{22}$, $P2_{17}$, $P6_{21}$, $M31_{272}$, $D40_{318}$, $S19_{141}$, $N27_{197}$, $P42_{320}$, $T17_{139}$
**X**	$W12_{34}$, $W17_{139}$, $H27_{197}$, $E15_{54}$, $C33_{298}$, $F50_{330}$, $\mathbf{N25_{166}}$, $L35_{300}$, $A34_{299}$, $S31_{272}$, $L54_{345}$, $Q38_{305}$, $\mathbf{D30_{270}}$, $E29_{247}$, $P1_{16}$, $S41_{319}$, $E13_{50}$, $P11_{32}$, $I56_{347}$, $A36_{301}$, $\mathbf{H32_{297}}$, $G55_{346}$, $Q40_{318}$, $L44_{323}$, $S16_{138}$, $\mathbf{E39_{317}}$
**XI**	-
**XII**	$N31_{272}$, $\mathbf{S32_{297}}$, $A27_{197}$, $H33_{298}$, $M16_{138}$, $N17_{139}$, $\mathbf{K39_{317}}$, $\mathbf{K30_{270}}$, $I36_{301}$

Listed in decreasing order of partial MI value. Subscripted positions correspond to those in PDB structure 1MDR:A. Residues in bold are in known catalytic residue positions for 1MDR:A.

References

1. Akiva E, Brown S, Almonacid DE, Barber 2nd AE, Custer AF, Hicks MA, et al. The Structure-Function Linkage Database. Nucl Acids Res. 2014 Jan;42(D1):D521–30.
