# Supplementary material for: Isofunctional Protein Subfamily Detection Using Data Integration and Spectral Clustering
Source: PLoS Comput Biol. 2016 Jun 27;12(6):e1005001. doi: 10.1371/journal.pcbi.1005001 (PMC4922564; doi:10.1371/journal.pcbi.1005001)
Supplement: S13 Text — (PDF) [file pcbi.1005001.s013.pdf]

Isofunctional Protein Subfamily Detection using Data Integration and Spectral Clustering

Elisa Boari de Lima[1,2,*], Wagner Meira Júnior[2], Raquel Cardoso de Melo-Minardi[2]

1 Department of Biochemistry and Immunology, Federal University of Minas Gerais, Belo Horizonte, MG, Brazil
2 Department of Computer Science, Federal University of Minas Gerais, Belo Horizonte, MG, Brazil

*** eblima@dcc.ufmg.br**

S13 Text: Analysis of the species distribution among clusters

In this Supplementary Text we perform a brief analysis of the distribution of species of origin among the clusters. We consider our most extreme subdivision case, namely the scattering of trypsin-labeled proteins when clustering the serine proteases into twelve clusters. Table S13.1 presents the amount of trypsin-labeled proteins [1] from each species in each cluster.

Table S13.1. Species distribution for the trypsin-labeled proteins when dividing the serine proteases into twelve clusters.

Cluster	Amount	Kingdom	Phylum	Class	Order	Family	Genus	Species
II	1	animalia	arthropoda	insecta	lepidoptera	bombycidae	bombyx	b. mori
II	1	animalia	arthropoda	insecta	lepidoptera	crambidae	ostrinia	o. furnacalis
II	6	animalia	arthropoda	insecta	lepidoptera	crambidae	ostrinia	o. nubilalis
II	7	animalia	arthropoda	insecta	lepidoptera	noctuidae	helicoverpa	h. armigera
II	1	animalia	arthropoda	insecta	lepidoptera	noctuidae	heliothis	h. virescens
II	1	animalia	arthropoda	insecta	lepidoptera	noctuidae	lacanobia	l. oleracea
II	6	animalia	arthropoda	insecta	lepidoptera	noctuidae	sesamia	s. nonagrioides
II	1	animalia	arthropoda	insecta	lepidoptera	pyralidae	galleria	g. mellonella
II	3	animalia	arthropoda	insecta	lepidoptera	pyralidae	plodia	p. interpunctella
II	3	animalia	arthropoda	insecta	lepidoptera	sphingidae	manduca	m. sexta
II	2	animalia	arthropoda	insecta	lepidoptera	tortricidae	choristoneura	c. fumiferana
IV	1	animalia	chordata	actinopterygii	acipenseriformes	acipenseridae	acipenser	a. transmontanus
IV	1	animalia	chordata	actinopterygii	cypriniformes	cyprinidae	danio	d. rerio
IV	2	animalia	chordata	actinopterygii	salminiformes	salmonidae	oncorhynchus	o. mykiss
IV	2	animalia	chordata	actinopterygii	tetraodontiformes	tetraodontidae	takifugu	t. rubripes
IV	4	animalia	chordata	actinopterygii	tetraodontiformes	tetraodontidae	tetraodon	t. nigroviridis
IV	3	animalia	chordata	amphibia	anura	pipidae	xenopus	x. laevis
IV	1	animalia	chordata	amphibia	anura	pipidae	xenopus	x. tropicalis
IV	1	animalia	chordata	amphibia	caudata	salamandridae	cynops	c. pyrrhogaster
IV	1	animalia	chordata	aves	galliformes	phasianidae	gallus	g. gallus
IV	1	animalia	chordata	aves	struthioniformes	struthionidae	struthio	s. camelus
IV	3	animalia	chordata	mammalia	artiodactyla	suidae	sus	s. scrofa
IV	2	animalia	chordata	mammalia	carnivora	canidae	canis	c. lupus familiaris
IV	3	animalia	chordata	mammalia	cetartiodactyla	bovidae	bos	b. taurus
IV	3	animalia	chordata	mammalia	lagomorpha	leporidae	oryctolagus	o. cuniculus
IV	1	animalia	chordata	mammalia	primates	cercopithecidae	macaca	m. mulatta
IV	3	animalia	chordata	mammalia	primates	hominidae	homo	h. sapiens
IV	1	animalia	chordata	mammalia	primates	hominidae	pongo	p. abelii
IV	5	animalia	chordata	mammalia	rodentia	muridae	mus	m. musculus
IV	4	animalia	chordata	mammalia	rodentia	muridae	rattus	r. norvegicus
IV	1	animalia	chordata	myxini	myxiniformes	myxinidae	eptatretus	e. stoutii
IV	1	animalia	chordata	reptilia	squamata	gekkonidae	gekko	g. gecko
IV	1	animalia	cnidaria	anthozoa	actiniaria	edwardsiidae	nematostella	n. vectensis
V	1	animalia	chordata	mammalia	artiodactyla	bovidae	ovis	o. aries
V	2	animalia	chordata	mammalia	carnivora	canidae	canis	c. lupus familiaris
V	2	animalia	chordata	mammalia	cetartiodactyla	bovidae	bos	b. taurus
V	3	animalia	chordata	mammalia	eulipotyphla	soricidae	blarina	b. brevicauda
V	1	animalia	chordata	mammalia	perissodactyla	equidae	equus	e. ferus caballus
V	1	animalia	chordata	mammalia	primates	cercopithecidae	cercopithecus	c. cephus
V	1	animalia	chordata	mammalia	primates	cercopithecidae	erythrocebus	e. patas
V	1	animalia	chordata	mammalia	primates	cercopithecidae	papio	p. anubis
V	4	animalia	chordata	mammalia	primates	hominidae	homo	h. sapiens
V	1	animalia	chordata	mammalia	primates	hominidae	pan	p. paniscus

Table S13.1. (continuation)

Cluster	Amount	Kingdom	Phylum	Class	Order	Family	Genus	Species
V	1	animalia	chordata	mammalia	primates	hominidae	pan	p. troglodytes
V	1	animalia	chordata	mammalia	primates	hominidae	pongo	p. pygmaeus
V	10	animalia	chordata	mammalia	rodentia	muridae	mus	m. musculus
V	1	animalia	chordata	mammalia	rodentia	muridae	praomys	p. natalensis
V	8	animalia	chordata	mammalia	rodentia	muridae	rattus	r. norvegicus
VI	9	animalia	chordata	actinopterygii	cypriniformes	cyprinidae	danio	d. rerio
VI	3	animalia	chordata	actinopterygii	tetraodontiformes	tetraodontidae	takifugu	t. rubripes
VI	3	animalia	chordata	actinopterygii	tetraodontiformes	tetraodontidae	tetraodon	t. nigroviridis
VI	2	animalia	chordata	amphibia	anura	pipidae	xenopodinae	x. laevis
VI	2	animalia	chordata	amphibia	anura	pipidae	xenopodinae	x. tropicalis
VI	2	animalia	chordata	aves	galliformes	phasianidae	gallus	g. gallus
VI	1	animalia	chordata	mammalia	artiodactyla	bovidae	ovis	o. aries
VI	2	animalia	chordata	mammalia	artiodactyla	suidae	sus	s. scrofa
VI	1	animalia	chordata	mammalia	carnivora	canidae	canis	c. lupus familiaris
VI	1	animalia	chordata	mammalia	carnivora	felidae	felis	f. silvestris catus
VI	4	animalia	chordata	mammalia	cetartiodactyla	bovidae	bos	b. taurus
VI	2	animalia	chordata	mammalia	lagomorpha	leporidae	oryctolagus	o. cuniculus
VI	1	animalia	chordata	mammalia	monotremata	ornithorhynchidae	ornithorhynchus	o. anatinus
VI	2	animalia	chordata	mammalia	primates	cercopithecidae	macaca	m. mulatta
VI	3	animalia	chordata	mammalia	primates	hominidae	homo	h. sapiens
VI	2	animalia	chordata	mammalia	primates	hominidae	pan	p. troglodytes
VI	1	animalia	chordata	mammalia	rodentia	caviidae	cavia	c. porcellus
VI	7	animalia	chordata	mammalia	rodentia	muridae	mus	m. musculus
VI	2	animalia	chordata	mammalia	rodentia	muridae	rattus	r. norvegicus
VI	1	animalia	chordata	reptilia	squamata	elapidae	notechis	n. scutatus
VI	1	animalia	chordata	reptilia	squamata	elapidae	oxyuranus	o. microlepidotus
VI	1	animalia	chordata	reptilia	squamata	elapidae	oxyuranus	o. scutellatus
VI	1	animalia	chordata	reptilia	squamata	elapidae	pseudechis	p. porphyriacus
VI	3	animalia	chordata	reptilia	squamata	elapidae	pseudonaja	p. textilis
VI	1	animalia	chordata	reptilia	squamata	elapidae	pseudonaja	p. textilis textilis
VI	2	animalia	chordata	reptilia	squamata	elapidae	tropidechis	t. carinatus
VII	1	animalia	chordata	amphibia	anura	pipidae	xenopodinae	x. laevis
VII	19	animalia	chordata	mammalia	primates	hominidae	homo	h. sapiens
VII	11	animalia	chordata	mammalia	rodentia	muridae	mus	m. musculus
VII	1	animalia	chordata	reptilia	squamata	colubridae	philodryas	p. olfersii
VII	1	animalia	chordata	reptilia	squamata	elapidae	hydrophis	h. hardwickii
VII	7	animalia	chordata	reptilia	squamata	viperidae	agkistrodon	a. acutus
VII	1	animalia	chordata	reptilia	squamata	viperidae	agkistrodon	a. bilineatus
VII	1	animalia	chordata	reptilia	squamata	viperidae	agkistrodon	a. caliginosus
VII	2	animalia	chordata	reptilia	squamata	viperidae	agkistrodon	a. contortrix
VII	2	animalia	chordata	reptilia	squamata	viperidae	agkistrodon	a. halys blomhoffi
VII	1	animalia	chordata	reptilia	squamata	viperidae	agkistrodon	a. halys brevicaudus
VII	7	animalia	chordata	reptilia	squamata	viperidae	agkistrodon	a. halys pallas

Table S13.1. (continuation)

Cluster	Amount	Kingdom	Phylum	Class	Order	Family	Genus	Species
VII	1	animalia	chordata	reptilia	squamata	viperidae	agkistrodon	a. rhodostoma
VII	1	animalia	chordata	reptilia	squamata	viperidae	bothrops	b. insularis
VII	4	animalia	chordata	reptilia	squamata	viperidae	bothrops	b. jararaca
VII	1	animalia	chordata	reptilia	squamata	viperidae	crotalus	c. atrox
VII	1	animalia	chordata	reptilia	squamata	viperidae	crotalus	c. durissus durissus
VII	1	animalia	chordata	reptilia	squamata	viperidae	crotalus	c. durissus terrificus
VII	9	animalia	chordata	reptilia	squamata	viperidae	deinagkistrodon	d. acutus
VII	1	animalia	chordata	reptilia	squamata	viperidae	gloydius	g. saxatilis
VII	1	animalia	chordata	reptilia	squamata	viperidae	gloydius	g. shedaoensis
VII	4	animalia	chordata	reptilia	squamata	viperidae	gloydius	g. ussuriensis
VII	1	animalia	chordata	reptilia	squamata	viperidae	lachesis	l. muta muta
VII	1	animalia	chordata	reptilia	squamata	viperidae	lachesis	l. stenophrys
VII	2	animalia	chordata	reptilia	squamata	viperidae	protobothrops	p. elegans
VII	1	animalia	chordata	reptilia	squamata	viperidae	rhinocerophis	r. alternatus
VII	2	animalia	chordata	reptilia	squamata	viperidae	trimeresurus	t. albolabris
VII	2	animalia	chordata	reptilia	squamata	viperidae	trimeresurus	t. flavoviridis
VII	2	animalia	chordata	reptilia	squamata	viperidae	trimeresurus	t. gramineus
VII	2	animalia	chordata	reptilia	squamata	viperidae	trimeresurus	t. jerdonii
VII	2	animalia	chordata	reptilia	squamata	viperidae	trimeresurus	t. mucrosquamatus
VII	12	animalia	chordata	reptilia	squamata	viperidae	trimeresurus	t. stejnegeri
VII	1	animalia	chordata	reptilia	squamata	viperidae	vipera	v. lebetina
VII	5	animalia	chordata	reptilia	squamata	viperidae	viridovipera	v. stejnegeri
VIII	1	animalia	arthropoda	arachnida	ixodida	ixodidae	ixodes	i. scapularis
VIII	1	animalia	arthropoda	insecta	coleoptera	chrysomelidae	phaedon	p. cochleariae
VIII	1	animalia	arthropoda	insecta	coleoptera	scarabaeidae	holotrichia	h. diomphalia
VIII	1	animalia	arthropoda	insecta	diptera	cecidomyiidae	mayetiola	m. destructor
VIII	19	animalia	arthropoda	insecta	diptera	culicidae	aedes	a. aegypti
VIII	5	animalia	arthropoda	insecta	diptera	culicidae	anopheles	a. gambiae
VIII	1	animalia	arthropoda	insecta	diptera	culicidae	anopheles	a. gambiae str
VIII	12	animalia	arthropoda	insecta	diptera	drosophilidae	drosophila	d. melanogaster
VIII	5	animalia	arthropoda	insecta	diptera	drosophilidae	drosophila	d. pseudoobscura
VIII	1	animalia	arthropoda	insecta	diptera	psychodidae	phlebotomus	p. ariasi
VIII	1	animalia	arthropoda	insecta	lepidoptera	bombycidae	bombyx	b. mandarina
VIII	2	animalia	arthropoda	insecta	lepidoptera	bombycidae	bombyx	b. mori
VIII	2	animalia	arthropoda	insecta	lepidoptera	sphingidae	manduca	m. sexta
VIII	2	animalia	arthropoda	insecta	siphonaptera	pulicidae	ctenocephalides	c. felis
VIII	1	animalia	arthropoda	malacostraca	decapoda	nephropidae	homarus	h. americanus
VIII	4	animalia	chordata	actinopterygii	beloniformes	adrianichthyidae	oryzias	o. latipes
VIII	8	animalia	chordata	actinopterygii	cypriniformes	cyprinidae	danio	d. rerio
VIII	1	animalia	chordata	actinopterygii	salmoniformes	salmonidae	oncorhynchus	o. mykiss
VIII	1	animalia	chordata	actinopterygii	salmoniformes	salmonidae	salvelinus	s. fontinalis
VIII	2	animalia	chordata	actinopterygii	tetraodontiformes	tetraodontidae	tetraodon	t. nigroviridis
VIII	1	animalia	chordata	amphibia	anura	pipidae	xenopodinae	x. laevis
VIII	2	animalia	chordata	amphibia	anura	pipidae	xenopodinae	x. tropicalis

Table S13.1. (continuation)

Cluster	Amount	Kingdom	Phylum	Class	Order	Family	Genus	Species
VIII	2	animalia	chordata	ascidiacea	enterogona	cionidae	ciona	c. intestinalis
VIII	1	animalia	chordata	aves	galliformes	numididae	numida	n. meleagris
VIII	1	animalia	chordata	aves	galliformes	phasianidae	meleagris	m. gallopavo
VIII	2	animalia	chordata	mammalia	artiodactyla	bovidae	ovis	o. aries
VIII	3	animalia	chordata	mammalia	artiodactyla	suidae	sus	s. scrofa
VIII	2	animalia	chordata	mammalia	carnivora	canidae	canis	c. lupus familiaris
VIII	7	animalia	chordata	mammalia	cetartiodactyla	bovidae	bos	b. taurus
VIII	1	animalia	chordata	mammalia	diprotodontia	macropodidae	macropus	m. eugenii
VIII	1	animalia	chordata	mammalia	eulipotyphla	erinaceidae	erinaceus	e. europaeus
VIII	1	animalia	chordata	mammalia	lagomorpha	leporidae	oryctolagus	o. cuniculus
VIII	2	animalia	chordata	mammalia	perissodactyla	equidae	equus	e. ferus caballus
VIII	2	animalia	chordata	mammalia	primates	cercopithecidae	macaca	m. fascicularis
VIII	1	animalia	chordata	mammalia	primates	cercopithecidae	macaca	m. mulatta
VIII	1	animalia	chordata	mammalia	primates	cercopithecidae	papio	p. hamadryas
VIII	3	animalia	chordata	mammalia	primates	hominidae	gorilla	g. gorilla
VIII	1	animalia	chordata	mammalia	primates	hominidae	pan	p. troglodytes
VIII	5	animalia	chordata	mammalia	primates	hominidae	pongo	p. abelii
VIII	1	animalia	chordata	mammalia	rodentia	caviidae	cavia	c. porcellus
VIII	1	animalia	chordata	mammalia	rodentia	muridae	meriones	m. unguiculatus
VIII	5	animalia	chordata	mammalia	rodentia	muridae	rattus	r. norvegicus
VIII	1	animalia	chordata	myxini	myxiniformes	myxinidae	eptatretus	e. burgeri
VIII	1	animalia	chordata	reptilia	squamata	viperidae	agkistrodon	a. acutus
VIII	1	animalia	chordata	reptilia	squamata	viperidae	bothrops	b. jararacussu
VIII	1	animalia	chordata	reptilia	squamata	viperidae	deinagkistrodon	d. acutus
VIII	3	animalia	cnidaria	anthozoa	actiniaria	edwardsiidae	nematostella	n. vectensis
VIII	1	animalia	nematoda	chromadorea	rhabditida	rhabditidae	caenorhabditis	c. elegans
VIII	1	bacteria	chloroflexi	chloroflexi	chloroflexales	chloroflexaceae	roseiflexus	roseiflexus sp
VIII	2	bacteria	cyanobacteria	synechococcales		synechococcaceae	synechococcus	synechococcus sp.
VIII	1	bacteria	proteobacteria	gammaproteobacteria	alteromonadales	colwelliaceae	colwellia	c. psychrerythraea
VIII	1	bacteria	proteobacteria	gammaproteobacteria	oceanospirillales	hahellaceae	hahella	h. chejuensis
VIII	1	chromalveolata	heterokontophyta	oomycetes	saprolegniales	leptolegniaceae	aphanomyces	a. astaci
IX	1	animalia	arthropoda	insecta	diptera	calliphoridae	lucilia	l. cuprina
IX	2	animalia	arthropoda	insecta	diptera	culicidae	aedes	a. aegypti
IX	5	animalia	arthropoda	insecta	diptera	culicidae	anopheles	a. gambiae
IX	10	animalia	arthropoda	insecta	diptera	drosophilidae	drosophila	d. melanogaster
IX	2	animalia	arthropoda	insecta	diptera	drosophilidae	drosophila	d. pseudoobscura
IX	1	animalia	arthropoda	insecta	diptera	glossinidae	glossina	g. morsitans
IX	2	animalia	arthropoda	insecta	diptera	oestridae	hypoderma	h. lineatum
IX	1	animalia	arthropoda	insecta	lepidoptera	noctuidae	agrotis	a. ipsilon
IX	1	animalia	chordata	actinopterygii	cypriniformes	cyprinidae	cyprinus	c. carpio
IX	13	animalia	chordata	actinopterygii	cypriniformes	cyprinidae	danio	d. rerio
IX	1	animalia	chordata	actinopterygii	gadiformes	gadidae	gadus	g. morhua
IX	1	animalia	chordata	actinopterygii	pleuronectiformes	paralichthyidae	paralichthys	p. olivaceus
IX	4	animalia	chordata	actinopterygii	tetraodontiformes	tetraodontidae	tetraodon	t. nigroviridis

Table S13.1. (continuation)

Cluster	Amount	Kingdom	Phylum	Class	Order	Family	Genus	Species
IX	5	animalia	chordata	amphibia	anura	pipidae	xenopus	x. laevis
IX	3	animalia	chordata	amphibia	anura	pipidae	xenopus	x. tropicalis
IX	1	animalia	chordata	appendicularia	copelata	oikopleuridae	oikopleura	o. dioica
IX	1	animalia	chordata	ascidiacea	stolidobranchia	styelidae	polyandrocarpa	p. misakiensis
IX	1	animalia	chordata	aves	anseriformes	anatidae	anas	a. platyrhynchos
IX	1	animalia	chordata	aves	galliformes	phasianidae	coturnix	c. coturnix
IX	1	animalia	chordata	aves	galliformes	phasianidae	meleagris	m. gallopavo
IX	1	animalia	chordata	chondrichthyes	carcharhiniformes	trikidae	triakis	t. scyllium
IX	1	animalia	chordata	mammalia	artiodactyla	bovidae	ovis	o. aries
IX	4	animalia	chordata	mammalia	artiodactyla	suidae	sus	s. scrofa
IX	1	animalia	chordata	mammalia	carnivora	canidae	canis	c. lupus familiaris
IX	5	animalia	chordata	mammalia	cetartiodactyla	bovidae	bos	b. taurus
IX	4	animalia	chordata	mammalia	chiroptera	phyllostomidae	desmodus	d. rotundus
IX	2	animalia	chordata	mammalia	lagomorpha	leporidae	oryctolagus	o. cuniculus
IX	1	animalia	chordata	mammalia	primates	callitrichidae	saguinus	s. labiatus
IX	2	animalia	chordata	mammalia	primates	cercopithecidae	macaca	m. mulatta
IX	1	animalia	chordata	mammalia	primates	cercopithecidae	papio	p. cynocephalus
IX	1	animalia	chordata	mammalia	primates	cercopithecidae	pygathrix	p. nemaeus
IX	1	animalia	chordata	mammalia	primates	cercopithecidae	rhinopithecus	r. bieti
IX	1	animalia	chordata	mammalia	primates	cercopithecidae	trachypithecus	t. francoisi
IX	1	animalia	chordata	mammalia	primates	cercopithecidae	trachypithecus	t. phayrei
IX	2	animalia	chordata	mammalia	primates	hominidae	gorilla	g. gorilla
IX	28	animalia	chordata	mammalia	primates	hominidae	homo	h. sapiens
IX	1	animalia	chordata	mammalia	primates	hominidae	pan	p. paniscus
IX	2	animalia	chordata	mammalia	primates	hominidae	pan	p. troglodytes
IX	4	animalia	chordata	mammalia	primates	hominidae	pongo	p. abelii
IX	1	animalia	chordata	mammalia	primates	hominidae	pongo	p. pygmaeus
IX	1	animalia	chordata	mammalia	primates	hylobatidae	hoolock	h. hoolock
IX	1	animalia	chordata	mammalia	primates	hylobatidae	nomascus	n. leucogenys
IX	1	animalia	chordata	mammalia	primates	hylobatidae	symphalangus	s. syndactylus
IX	36	animalia	chordata	mammalia	rodentia	muridae	mus	m. musculus
IX	16	animalia	chordata	mammalia	rodentia	muridae	rattus	r. norvegicus
IX	1	animalia	chordata	mammalia	rodentia	muridae	rattus	r. rattus
IX	7	animalia	cnidaria	anthozoa	actiniaria	edwardsiidae	nematostella	n. vectensis
IX	1	animalia	echinodermata	asteroidea	valvatida	asterinidae	patiria	p. pectinifera
IX	1	animalia	echinodermata	echinoidea	echinoida	strongylocentrotidae	strongylocentrotus	s. purpuratus
IX	1	animalia	nematoda	chromadorea	rhabditida	rhabditidae	caenorhabditis	c. elegans
IX	3	bacteria	actinobacteria	actinobacteria	actinomycetales	pseudonocardiaceae	saccharopolyspora	s. erythraea
IX	1	bacteria	actinobacteria	actinobacteria	actinomycetales	streptomycetaceae	streptomyces	s. grieus
X	1	animalia	arthropoda	arachnida	astigmata	glycyphagidae	glycyphagus	g. domesticus
X	1	animalia	arthropoda	arachnida	astigmata	glycyphagidae	lepidoglyphus	l. destructor
X	1	animalia	arthropoda	insecta	coleoptera	bostrichidae	rhyzopertha	r. dominica
X	2	animalia	arthropoda	insecta	coleoptera	lampyridae	pyrocoelia	p. rufa
X	1	animalia	arthropoda	insecta	siphonaptera	pulicidae	ctenocephalides	c. felis

Table S13.1. (continuation)

Cluster	Amount	Kingdom	Phylum	Class	Order	Family	Genus	Species
X	16	animalia	arthropoda	maxillopoda	siphonostomatoida	caligidae	lepeophtheirus	l. salmonis
X	1	animalia	chordata	actinopterygii	anguilliformes	anguillidae	anguilla	a. japonica
X	1	animalia	chordata	actinopterygii	beloniformes	adrianichthyidae	oryzias	o. latipes
X	2	animalia	chordata	actinopterygii	clupeiformes	engraulidae	engraulis	e. japonicus
X	1	animalia	chordata	actinopterygii	cypriniformes	catostomidae	myxocyprinus	m. asiaticus
X	1	animalia	chordata	actinopterygii	cypriniformes	cyprinidae	carassius	c. auratus
X	4	animalia	chordata	actinopterygii	cypriniformes	cyprinidae	danio	d. rerio
X	1	animalia	chordata	actinopterygii	perciformes	carangidae	seriola	s. quinqueradiata
X	2	animalia	chordata	actinopterygii	perciformes	cichlidae	oreochromis	o. aureus
X	2	animalia	chordata	actinopterygii	perciformes	cichlidae	oreochromis	o. niloticus
X	1	animalia	chordata	actinopterygii	perciformes	labridae	tautogolabrus	t. adspersus
X	5	animalia	chordata	actinopterygii	perciformes	nototheniidae	dissostichus	d. mawsoni
X	1	animalia	chordata	actinopterygii	perciformes	nototheniidae	notothenia	n. coriiceps
X	1	animalia	chordata	actinopterygii	perciformes	nototheniidae	paranotothenia	p. magellanica
X	3	animalia	chordata	actinopterygii	perciformes	sparidae	sparus	s. aurata
X	4	animalia	chordata	actinopterygii	pleuronectiformes	paralichthyidae	paralichthys	p. olivaceus
X	1	animalia	chordata	actinopterygii	pleuronectiformes	pleuronectidae	kareius	k. bicoloratus
X	1	animalia	chordata	actinopterygii	pleuronectiformes	pleuronectidae	pleuronectes	p. platessa
X	2	animalia	chordata	actinopterygii	pleuronectiformes	pleuronectidae	pseudopleuronectes	p. americanus
X	6	animalia	chordata	actinopterygii	pleuronectiformes	soleidae	solea	s. senegalensis
X	1	animalia	chordata	actinopterygii	salmoniformes	salmonidae	oncorhynchus	o. keta
X	3	animalia	chordata	actinopterygii	salmoniformes	salmonidae	salmo	s. salar
X	1	animalia	chordata	actinopterygii	siluriformes	pangasiidae	pangasianodon	p. hypophthalmus
X	1	animalia	chordata	actinopterygii	tetraodontiformes	tetraodontidae	takifugu	t. rubripes
X	2	animalia	chordata	actinopterygii	tetraodontiformes	tetraodontidae	takifugu	t. rubripes
X	3	animalia	chordata	actinopterygii	tetraodontiformes	tetraodontidae	tetraodon	t. nigroviridis
X	3	animalia	chordata	actinopterygii	gadiformes	gadidae	gadus	g. morhua
X	11	animalia	chordata	amphibia	anura	pipidae	xenopodinae	x. laevis
X	8	animalia	chordata	amphibia	anura	pipidae	xenopodinae	x. tropicalis
X	2	animalia	chordata	ascidiacea	pleurogona	styelidae	botryllus	b. schlosseri
X	1	animalia	chordata	ascidiacea	stolidobranchia	styelidae	polyandrocarpa	p. misakiensis
X	4	animalia	chordata	aves	galliformes	phasianidae	gallus	g. gallus
X	1	animalia	chordata	aves	struthioniformes	struthionidae	struthio	s. camelus
X	1	animalia	chordata	chondrichthyes	squaliformes	squalidae	squalus	s. acanthias
X	4	animalia	chordata	hyperoartia	petromyzontiformes	petromyzontidae	petromyzon	p. marinus
X	1	animalia	chordata	mammalia	artiodactyla	suidae	sus	s. scrofa
X	1	animalia	chordata	mammalia	carnivora	canidae	canis	c. lupus familiaris
X	2	animalia	chordata	mammalia	carnivora	canidae	canis	c. lupus familiaris
X	6	animalia	chordata	mammalia	cetartiodactyla	bovidae	bos	b. taurus
X	1	animalia	chordata	mammalia	primates	callitrichidae	saguinus	s. oedipus
X	7	animalia	chordata	mammalia	primates	cercopithecidae	macaca	m. mulatta
X	23	animalia	chordata	mammalia	primates	hominidae	homo	h. sapiens
X	2	animalia	chordata	mammalia	rodentia	caviidae	cavia	c. porcellus
X	26	animalia	chordata	mammalia	rodentia	muridae	mus	m. musculus

Table S13.1. (continuation)

Cluster	Amount	Kingdom	Phylum	Class	Order	Family	Genus	Species
X	8	animalia	chordata	mammalia	rodentia	muridae	rattus	r. norvegicus
X	5	animalia	cnidaria	anthozoa	actiniaria	edwardsiidae	nematostella	n. vectensis
X	1	animalia	echinodermata	asteroidea	paxillosida	luidiidae	luidia	l. foliolata
X	1	fungi	ascomycota	sordariomycetes	hypocreales	hypocreaceae	trichoderma	t. harzianum
XI	2	animalia	arthropoda	arachnida	acariformes	pyroglyphidae	dermatophagoides	d. farinae
XI	3	animalia	arthropoda	arachnida	acariformes	pyroglyphidae	dermatophagoides	d. pteronyssinus
XI	1	animalia	arthropoda	arachnida	araneae	agelenidae	agelenopsis	a. aperta
XI	1	animalia	arthropoda	arachnida	astigmata	pyroglyphidae	euroglyphus	e. maynei
XI	1	animalia	arthropoda	arachnida	ixodida	ixodidae	dermacentor	d. andersoni
XI	1	animalia	arthropoda	arachnida	ixodida	ixodidae	dermacentor	d. variabilis
XI	1	animalia	arthropoda	arachnida	sarcoptiformes	acaridae	tyrophagus	t. putrescentiae
XI	1	animalia	arthropoda	arachnida	sarcoptiformes	sarcoptidae	sarcoptes	s. scabiei
XI	1	animalia	arthropoda	insecta	blattodea	blattellidae	blattella	b. germanica
XI	1	animalia	arthropoda	insecta	coleoptera	curculionidae	anthonomus	a. grandis
XI	1	animalia	arthropoda	insecta	coleoptera	curculionidae	diaprepes	d. abbreviatus
XI	1	animalia	arthropoda	insecta	coleoptera	enebrionidae	tenebrio	t. molitor
XI	2	animalia	arthropoda	insecta	coleoptera	scarabaeidae	holotrichia	h. diomphalia
XI	3	animalia	arthropoda	insecta	diptera	ceratopogonidae	culicoides	c. sonorensis
XI	46	animalia	arthropoda	insecta	diptera	culicidae	aedes	a. aegypti
XI	2	animalia	arthropoda	insecta	diptera	culicidae	anopheles	a. dirus
XI	28	animalia	arthropoda	insecta	diptera	culicidae	anopheles	a. gambiae
XI	3	animalia	arthropoda	insecta	diptera	culicidae	anopheles	a. gambiae str
XI	1	animalia	arthropoda	insecta	diptera	culicidae	culex	c. quinquefasciatus
XI	35	animalia	arthropoda	insecta	diptera	drosophilidae	drosophila	d. melanogaster
XI	20	animalia	arthropoda	insecta	diptera	drosophilidae	drosophila	d. pseudoobscura
XI	1	animalia	arthropoda	insecta	diptera	psychodidae	lutzomyia	l. longipalpis
XI	1	animalia	arthropoda	insecta	diptera	psychodidae	phlebotomus	p. papatasi
XI	1	animalia	arthropoda	insecta	diptera	simuliidae	simulium	s. vittatum
XI	2	animalia	arthropoda	insecta	hemiptera	delphacidae	nilaparvata	n. lugens
XI	1	animalia	arthropoda	insecta	hymenoptera	apidae	bombus	b. pensylvanicus
XI	1	animalia	arthropoda	insecta	hypenoptera	vespidae	polistes	p. dominula
XI	1	animalia	arthropoda	insecta	lepidoptera	arctiidae	hyphantria	h. cunea
XI	3	animalia	arthropoda	insecta	lepidoptera	bombycidae	bombyx	b. mori
XI	1	animalia	arthropoda	insecta	lepidoptera	crambidae	ostrinia	o. nubilalis
XI	1	animalia	arthropoda	insecta	lepidoptera	plutellidae	plutella	p. xylostella
XI	1	animalia	arthropoda	insecta	lepidoptera	saturniidae	lonomia	l. obliqua
XI	7	animalia	arthropoda	insecta	lepidoptera	sphingidae	manduca	m. sexta
XI	1	animalia	arthropoda	insecta	phthiraptera	pediculidae	pediculus	p. humanus
XI	5	animalia	arthropoda	insecta	siphonaptera	pulicidae	ctenocephalides	c. felis
XI	1	animalia	arthropoda	malacostraca	decapoda	astacidae	astacus	a. fluvitilis
XI	1	animalia	arthropoda	malacostraca	decapoda	astacidae	astacus	a. leptodactylus
XI	2	animalia	arthropoda	malacostraca	decapoda	astacidae	pacifastacus	p. leniusculus
XI	1	animalia	arthropoda	malacostraca	decapoda	palinuridae	panulirus	p. argus
XI	1	animalia	arthropoda	malacostraca	decapoda	penaeidae	litopenaeus	l. vannamei

Table S13.1. (continuation)

Cluster	Amount	Kingdom	Phylum	Class	Order	Family	Genus	Species
XI	1	animalia	arthropoda	malacostraca	decapoda	penaeidae	penaeus	p. japonicus
XI	3	animalia	arthropoda	malacostraca	decapoda	penaeidae	penaeus	p. vannamei
XI	1	animalia	arthropoda	malacostraca	decapoda	portunidae	callinectes	c. sapidus
XI	4	animalia	arthropoda	merostomata	xiphosura	limulidae	tachypleus	t. tridentatus
XI	1	animalia	chordata	actinopterygii	cypriniformes	cyprinidae	cyprinus	c. carpio
XI	7	animalia	chordata	actinopterygii	cypriniformes	cyprinidae	danio	d. rerio
XI	1	animalia	chordata	actinopterygii	tetraodontiformes	tetraodontidae	takifugu	t. rubripes
XI	3	animalia	chordata	actinopterygii	tetraodontiformes	tetraodontidae	tetraodon	t. nigroviridis
XI	6	animalia	chordata	amphibia	anura	pipidae	xenopodinae	x. laevis
XI	2	animalia	chordata	amphibia	anura	pipidae	xenopodinae	x. tropicalis
XI	1	animalia	chordata	ascidiacea	pleurogona	pyuridae	halocynthia	h. roretzi
XI	1	animalia	chordata	ascidiacea	pleurogona	pyuridae	herdmania	h. momus
XI	1	animalia	chordata	ascidiacea	stolidobranchia	pyuridae	boltenia	b. villosa
XI	3	animalia	chordata	aves	galliformes	phasianidae	gallus	g. gallus
XI	1	animalia	chordata	leptocardii	amphioxiformes	branchiostomidae	branchiostoma	b. belcheri
XI	1	animalia	chordata	mammalia	artiodactyla	bovidae	bubalus	b. bubalis
XI	1	animalia	chordata	mammalia	artiodactyla	bovidae	ovis	o. aries
XI	3	animalia	chordata	mammalia	artiodactyla	suidae	sus	s. scrofa
XI	1	animalia	chordata	mammalia	carnivora	canidae	canis	c. lupus familiaris
XI	4	animalia	chordata	mammalia	cetartiodactyla	bovidae	bos	b. taurus
XI	1	animalia	chordata	mammalia	primates	cercopithecidae	macaca	m. mulatta
XI	14	animalia	chordata	mammalia	primates	hominidae	homo	h. sapiens
XI	1	animalia	chordata	mammalia	primates	hominidae	pan	p. paniscus
XI	1	animalia	chordata	mammalia	primates	hominidae	pan	p. troglodytes
XI	2	animalia	chordata	mammalia	primates	hominidae	pongo	p. pygmaeus
XI	13	animalia	chordata	mammalia	rodentia	muridae	mus	m. musculus
XI	3	animalia	chordata	mammalia	rodentia	muridae	rattus	r. norvegicus
XI	3	animalia	cnidaria	anthozoa	actiniaria	edwardsiidae	nematostella	n. vectensis
XI	1	animalia	echinodermata	echinoidea	echinoida	strongylocentrotidae	strongylocentrotus	s. purpuratus
XI	1	animalia	mollusca	gastropoda		lymnaeidae	radix	r. peregra
XI	1	animalia	mollusca	gastropoda		planorbidae	biompalaria	b. glabrata
XI	3	animalia	nematoda	adenophorea	trichocephalida		trichinella	t. spiralis
XI	1	animalia	porifera	demospongie	verongida	aplysinidae	aplysina	a. fistularis
XI	1	bacteria	proteobacteria	alphaproteobacteria	rhodobacteriales	rhodobacteraceae	roseovarius	r. nubinhibens
XI	1	bacteria	proteobacteria	delta proteobacteria	myxococcales	myxococcaceae	myxococcus	m. xanthus
XI	1	bacteria	proteobacteria	deltaproteobacteria	bdellovibrionales	bdellovibrionaceae	bdellovibrio	b. bacteriovorus
XI	1	bacteria	proteobacteria	gammaproteobacteria	alteromonadales	psychromonadaceae	psychromonas	p. ingrahamii
XI	1	bacteria	proteobacteria	gammaproteobacteria	oceanospiriallales	oceanospirillaceae	bermanella	b. marisrubri
XI	2	bacteria	proteobacteria	gammaproteobacteria	oceanospirillales	hahellaceae	hahella	h. chejuensis
XI	2	bacteria	proteobacteria	gammaproteobacteria	vibrionales	vibrionaceae	vibrio	v. antiquarius
XI	1	bacteria	proteobacteria	gammaproteobacteria	vibrionales	vibrionaceae	vibrio	v. campbellii
XI	1	bacteria	proteobacteria	gammaproteobacteria	vibrionales	vibrionaceae	vibrio	v. cholerae
XI	1	bacteria	proteobacteria	gammaproteobacteria	vibrionales	vibrionaceae	vibrio	v. parahaemolyticus
XI	1	bacteria	proteobacteria	gammaproteobacteria	vibrionales	vibrionaceae	vibrio	v. parahaemolyticus

Table S13.1. (continuation)

Cluster	Amount	Kingdom	Phylum	Class	Order	Family	Genus	Species
XI	2	bacteria	proteobacteria	gammaproteobacteria	vibrionales	vibrionaceae	vibrio	v. vulnificus
XI	1	bacteria	proteobacteria	gammaproteobacteria	vibrionales	vibrionaceae	vibrionales	v. bacterium
XI	1	fungi	ascomycota	dothideomycetes	pleosporales	phaeosphaeriaceae	parastagonospora	p. nodorum
XI	1	fungi	ascomycota	dothideomycetes	pleosporales	pleosporaceae	cochliobolus	c. carbonum
XI	1	fungi	ascomycota	sordariomycetes	hypocreales	hypocreaceae	trichoderma	t. harzianum
XI	1	fungi	ascomycota	sordariomycetes	hypocreales	incertae sedis	verticillium	v. dahliae
XI	1	fungi	ascomycota	sordariomycetes	hypocreales	nectriaceae	fusarium	f. oxysporum
XI	1	fungi	zygomycota	incertae sedis	entomophthorales	entomophthoraceae	zoophthora	z. radicans
XI	1	fungi	ascomycota	leotiomycetes	helotiales	sclerotiniaceae	sclerotinia	s. sclerotiorum
XI	1	plantae	heterokontophyta	oomycetes	peronosporales	pythiaceae	phytophthora	p. infestans
XII	1	animalia	annelida	polychaeta	capitellida	arenicolidae	arenicola	a. marina
XII	1	animalia	arthropoda	arachnida	ixodida	ixodidae	ixodes	i. scapularis
XII	1	animalia	arthropoda	arachnida	sarcoptiformes	acaridae	aleuroglyphus	a. ovatus
XII	2	animalia	arthropoda	arachnida	sarcoptiformes	glycyphagidae	blomia	b. tropicalis
XII	1	animalia	arthropoda	chilopoda	scolopendromorpha	scolopendridae	scolopendra	s. subspinipes
XII	1	animalia	arthropoda	insecta	blattodea	blattidae	periplaneta	p. americana
XII	1	animalia	arthropoda	insecta	blattodea	corydiidae	eupolyphaga	e. sinensis
XII	2	animalia	arthropoda	insecta	coleoptera	bostrichidae	rhyzopertha	r. dominica
XII	1	animalia	arthropoda	insecta	coleoptera	chrysomelidae	zabrotes	z. subfasciatus
XII	4	animalia	arthropoda	insecta	coleoptera	enebrionidae	tenebrio	t. molitor
XII	6	animalia	arthropoda	insecta	diptera	cecidomyiidae	mayetiola	m. destructor
XII	1	animalia	arthropoda	insecta	diptera	ceratopogonidae	culicoides	c. sonorensis
XII	23	animalia	arthropoda	insecta	diptera	culicidae	aedes	a. aegypti
XII	19	animalia	arthropoda	insecta	diptera	culicidae	anopheles	a. gambiae
XII	2	animalia	arthropoda	insecta	diptera	culicidae	anopheles	a. gambiae str
XII	2	animalia	arthropoda	insecta	diptera	culicidae	anopheles	a. stephensi
XII	1	animalia	arthropoda	insecta	diptera	culicidae	culex	c. pipiens
XII	1	animalia	arthropoda	insecta	diptera	culicidae	culex	c. pipiens pallens
XII	2	animalia	arthropoda	insecta	diptera	culicidae	culex	c. quinquefasciatus
XII	7	animalia	arthropoda	insecta	diptera	drosophilidae	drosophila	d. erecta
XII	40	animalia	arthropoda	insecta	diptera	drosophilidae	drosophila	d. melanogaster
XII	21	animalia	arthropoda	insecta	diptera	drosophilidae	drosophila	d. pseudoobscura
XII	3	animalia	arthropoda	insecta	diptera	drosophilidae	drosophila	d. simulans
XII	2	animalia	arthropoda	insecta	diptera	drosophilidae	drosophila	d. virilis
XII	2	animalia	arthropoda	insecta	diptera	ioestridae	hypoderma	h. lineatum
XII	2	animalia	arthropoda	insecta	diptera	muscidae	stomoxys	s. calcitrans
XII	3	animalia	arthropoda	insecta	diptera	psychodidae	lutzomyia	l. longipalpis
XII	2	animalia	arthropoda	insecta	diptera	psychodidae	phlebotomus	p. papatasi
XII	1	animalia	arthropoda	insecta	diptera	sarcophagidae	sarcophaga	s. bullata
XII	1	animalia	arthropoda	insecta	diptera	sarcophagidae	sarcophaga	s. peregrina
XII	1	animalia	arthropoda	insecta	hemiptera	miridae	lygus	l. hesperus
XII	4	animalia	arthropoda	insecta	hemiptera	miridae	lygus	l. lineolaris
XII	1	animalia	arthropoda	insecta	hymenoptera	diprionidae	neodiprion	n. abietis
XII	1	animalia	arthropoda	insecta	hymenoptera	diprionidae	neodiprion	n. lecontei

Table S13.1. (continuation)

Cluster	Amount	Kingdom	Phylum	Class	Order	Family	Genus	Species
XII	1	animalia	arthropoda	insecta	hymenoptera	diprionidae	neodiprion	n. sertifer
XII	4	animalia	arthropoda	insecta	lepidop	noctuidae	sesamia	s. nonagrioides
XII	5	animalia	arthropoda	insecta	lepidoptera	bombycidae	bombyx	b. mori
XII	1	animalia	arthropoda	insecta	lepidoptera	crambidae	ostrinia	o. nubilalis
XII	1	animalia	arthropoda	insecta	lepidoptera	noctuidae	agrotis	a. ipsilon
XII	1	animalia	arthropoda	insecta	lepidoptera	noctuidae	spodoptera	s. litura
XII	2	animalia	arthropoda	insecta	lepidoptera	sphingidae	manduca	m. sexta
XII	2	animalia	arthropoda	insecta	orthoptera	gryllidae	gryllys	g. firmus
XII	1	animalia	arthropoda	insecta	orthoptera	gryllidae	gryllys	g. pennsylvanicus
XII	1	animalia	arthropoda	insecta	orthoptera	gryllidae	gryllys	g. veletis
XII	2	animalia	arthropoda	insecta	phthiraptera	pediculidae	pediculus	p. humanus subsp
XII	1	animalia	arthropoda	insecta	siphonaptera	pulicidae	ctenocephalides	c. felis
XII	1	animalia	arthropoda	malacostraca	decapoda	lithodidae	paralithodes	p. camtschaticus
XII	2	animalia	arthropoda	malacostraca	decapoda	penaeidae	penaeus	p. monodon
XII	1	animalia	arthropoda	malacostraca	decapoda	portunidae	portunus	p. pelagicus
XII	1	animalia	arthropoda	malacostraca	decapoda	sesarmidae	chiromantes	c. haematocheir
XII	1	animalia	arthropoda	maxillopoda	siphonostomatoida	caligidae	lepeophtheirus	l. salmonis
XII	1	animalia	chordata	actinopterygii	beloniformes	adrianichthyidae	oryzias	o. latipes
XII	9	animalia	chordata	actinopterygii	cypriniformes	cyprinidae	danio	d. rerio
XII	1	animalia	chordata	actinopterygii	siluriformes	ictaluridae	ictalurus	i. punctatus
XII	4	animalia	chordata	actinopterygii	tetraodontiformes	tetraodontidae	tetraodon	t. nigroviridis
XII	18	animalia	chordata	amphibia	anura	pipidae	xenopodinae	x. laevis
XII	6	animalia	chordata	amphibia	anura	pipidae	xenopodinae	x. tropicalis
XII	1	animalia	chordata	appendicularia	copelata	oikopleuridae	oikopleura	o. dioica
XII	1	animalia	chordata	ascidiacea	enterogona	cionidae	ciona	c. intestinalis
XII	1	animalia	chordata	aves	galliformes	phasianidae	gallus	g. gallus
XII	1	animalia	chordata	cephalaspidomorphi	petromyzontiformes	petromyzontidae	lethenteron	l. japonica
XII	2	animalia	chordata	leptocardii	amphioxiformes	branchiostomidae	branchiostoma	b. belcheri
XII	2	animalia	chordata	mammalia	artiodactyla	suidae	sus	s. scrofa
XII	9	animalia	chordata	mammalia	cetartiodactyla	bovidae	bos	b. taurus
XII	1	animalia	chordata	mammalia	lagomorpha	leporidae	oryctolagus	o. cuniculus
XII	15	animalia	chordata	mammalia	primates	hominidae	homo	h. sapiens
XII	5	animalia	chordata	mammalia	primates	hominidae	pongo	p. abelii
XII	1	animalia	chordata	mammalia	rodentia	caviidae	cavia	c. porcellus
XII	25	animalia	chordata	mammalia	rodentia	muridae	mus	m. musculus
XII	10	animalia	chordata	mammalia	rodentia	muridae	rattus	r. norvegicus
XII	1	animalia	chordata	reptilia	squamata	varanidae	varanus	v. acanthurus
XII	1	animalia	chordata	reptilia	squamata	varanidae	varanus	v. mitchelli
XII	12	animalia	cnidaria	anthozoa	actiniaria	edwardsiidae	nematostella	n. vectensis
XII	2	animalia	mollusca	bivalvia	pectinoida	pectinidae	azumapecten	a. farreri
XII	1	animalia	mollusca	gastropoda		planorbidae	biompalaria	b. glabrata
XII	3	bacteria	actinobacteria	actinobacteria	actinomycetales	streptomycetaceae	streptomyces	s. avermitilis
XII	1	bacteria	actinobacteria	actinobacteria	actinomycetales	streptomycetaceae	streptomyces	s. coelicolor
XII	1	bacteria	actinobacteria	actinobacteria	actinomycetales	streptomycetaceae	streptomyces	s. exfoliatus

Table S13.1. (continuation)

Cluster	Amount	Kingdom	Phylum	Class	Order	Family	Genus	Species
XII	1	bacteria	actinobacteria	actinobacteria	actinomycetales	streptomycetaceae	streptomyces	s. fradiae
XII	1	bacteria	actinobacteria	actinobacteria	actinomycetales	streptomycetaceae	streptomyces	s. glaucescens
XII	3	bacteria	actinobacteria	actinobacteria	actinomycetales	streptomycetaceae	streptomyces	s. griseus
XII	2	bacteria	actinobacteria	actinovacteria	actinomycetales	pseudonocardiaceae	saccharopolyspora	s. erythraea
XII	1	bacteria	proteobacteria	gammaproteobacteria	vibrionales	vibrionaceae	vibrio	v. campbellii
XII	1	bacteria	proteobacteria	gammaproteobacteria	vibrionales	vibrionaceae	vibrio	v. cholerae
XII	1	fungi	ascomycota	dothideomycetes	pleosporales	phaeosphaeriaceae	parastagonospora	p. nodorum
XII	1	fungi	ascomycota	eurotiomycetes	eurotiales	trichocomaceae	emericella	e. nidulans
XII	3	fungi	ascomycota	sordariomycetes	hypocreales	clavicipitaceae	metarhizium	m. ansiopliae
XII	1	plantae	chlorophyta	prasinophyceae	mamiellales	bathycoccaceae	ostreococcus	o. lucimarinus

One may observe that, although the larger clusters present mixtures even at the kingdom level, some interesting associations exist: all 32 trypsins in cluster II are from the lepidoptera order of the insecta class; all 38 in cluster V are from the mammalia class; all trypsins in clusters VI and VII, as well as all but one from cluster IV, are from the chordata phylum, although there are representatives from multiple classes. Almost all trypsins in cluster VII are from snake species. The trypsins in the remaining clusters, however, are a mixture.

This analysis shows that the trypsin subdivision is not phylogeny-related except for the aforementioned cases. The mixture in the larger clusters indicates a larger number of clusters would be required in order to create subdivisions for specific phylogenetic clades.

References

1. Melo-Minardi RC, Bastard K, Artiguenave F. Identification of subfamily-specific sites based on active sites modeling and clustering. Bioinformatics. 2010 Dec;26(24):3075–3082.
