# Supplementary material for: Isofunctional Protein Subfamily Detection Using Data Integration and Spectral Clustering
Source: PLoS Comput Biol. 2016 Jun 27;12(6):e1005001. doi: 10.1371/journal.pcbi.1005001 (PMC4922564; doi:10.1371/journal.pcbi.1005001)
Supplement: S14 Text — (PDF) [file pcbi.1005001.s014.pdf]

Isofunctional Protein Subfamily Detection using Data Integration and Spectral Clustering

Elisa Boari de Lima[1,2,*], Wagner Meira Júnior[2], Raquel Cardoso de Melo-Minardi[2]

1 Department of Biochemistry and Immunology, Federal University of Minas Gerais, Belo Horizonte, MG, Brazil
2 Department of Computer Science, Federal University of Minas Gerais, Belo Horizonte, MG, Brazil

*** eblima@dcc.ufmg.br**

S14 Text: Analysis of the EC Number distribution among clusters

In this Supplementary Text we perform a brief analysis of the distribution of Enzyme Commission (EC) numbers among the clusters generated by the proposed framework. Complete EC numbers were only available for a subset of the studied protein families, namely 200 of the 461 nucleotidyl cyclases, 320 of the 1,533 serine proteases, and 956 of the 3,087 protein kinases.

As described in the main text, when a ground-truth exists, external validation measures may be used to calculate a clustering's agreement with it. In this analysis, we will consider the EC numbers as a ground-truth classification, even though the EC system has been shown to be unreliable as a gold standard [1]. Nevertheless, to compare the clusters to the known EC numbers, we use precision, recall, the F_1 score, the Rand index, and the Jaccard coefficient.

The quality measures for the nucleotidyl cyclases are presented in Table S14.1. One may observe all measurements decrease as the number of clusters increases. This is due to this family only having two distinct EC numbers, namely 4.6.1.1 and 4.6.1.2. Thus, when more than two clusters exist, the EC numbers are inevitably scattered among different clusters, consequently decreasing the agreement between clustering and EC numbers.

Table S14.1. External quality measures for the clusterings produced by the GP System for the nucleotidyl cyclase family.

Measure	2 Clusters	3 Clusters	6 Clusters
**Rand index**	0.94	0.94	0.79
**Jaccard coefficient**	0.93	0.93	0.76
**Precision**	99.57%	99.57%	99.47%
**Recall**	93.81%	93.81%	76.64%
**F_1 score**	0.97	0.97	0.87

Table S14.2 presents the quality measures calculated for the serine proteases. One may observe all measurements except for the recall increase as the number of clusters increases. This family comprises proteins with 33 distinct EC numbers, thus the increase in the quality measurements shows that the clusterings are in better agreement with the EC numbers as the number of clusters increases. Hence, the subclusters for the proteins labeled as trypsins in [2] are justified by there actually existing subdivisions among them, which the proposed framework was capable of finding, as reflected by the high Rand indices. However, experiments with even more clusters are required in order to evaluate if the GP system is capable of creating EC number-specific clusters.

Table S14.2. External quality measures for the clusterings produced by the GP System for the serine protease family.

Measure	4 Clusters	11 Clusters	12 Clusters
**Rand index**	0.47	0.85	0.87
**Jaccard coefficient**	0.11	0.24	0.26
**Precision**	11.97%	35.52%	42.20%
**Recall**	59.16%	40.99%	40.43%
**F_1 score**	0.20	0.38	0.41

Lastly, the quality measures calculated for the protein kinases are presented in Table S14.3. One may observe an increase in the Rand index and precision as the number of clusters increases, yet the Jaccard coefficient, recall, and F_1 score decrease. Sixteen different complete EC numbers exist among the proteins studied in this family. The Rand index's initial high value and increase along with the number of clusters shows that the additional clusters aid the separation into EC number-specific clusters. The increase in precision shows that there are more same-cluster pairs that actually have the same EC number, however the decrease in recall shows that there are more same-EC number protein pairs being put in different clusters as the amount of clusters increases. This may be related to the EC system's inadequacy as a gold standard.

Table S14.3. External quality measures for the clusterings produced by the GP System for the protein kinase family.

Measure	2 Clusters	3 Clusters	7 Clusters
**Rand index**	0.72	0.76	0.83
**Jaccard coefficient**	0.46	0.43	0.42
**Precision**	45.71%	49.50%	69.68%
**Recall**	100%	75.11%	50.77%
**F_1 score**	0.63	0.60	0.59

Overall, the analysis of the EC number distribution among clusters justified the existence of subclusters: they exist because there actually exist more specific classifications than those reflected by the subfamily labels employed in [2]. Thus, the clusters generated by the proposed framework are in accordance with the existing EC number annotations, although experiments with larger numbers of clusters are required in order to test if the framework is capable of creating EC-specific clusters.

References

1. Silveira SA, de Melo-Minardi RC, da Silveira CH, Santoro MM, Meira Jr W. ENZYMAP: Exploiting Protein Annotation for Modeling and Predicting EC number changes in UniProt/Swiss-Prot. PLOS One. 2014 Feb;9(2):e89162.

2. Melo-Minardi RC, Bastard K, Artiguenave F. Identification of subfamily-specific sites based on active sites modeling and clustering. Bioinformatics. 2010 Dec;26(24):3075–3082.
