# Supplementary material for: Isofunctional Protein Subfamily Detection Using Data Integration and Spectral Clustering
Source: PLoS Comput Biol. 2016 Jun 27;12(6):e1005001. doi: 10.1371/journal.pcbi.1005001 (PMC4922564; doi:10.1371/journal.pcbi.1005001)
Supplement: S15 Text — (PDF) [file pcbi.1005001.s015.pdf]

Isofunctional Protein Subfamily Detection using Data Integration and Spectral Clustering

Elisa Boari de Lima[1,2,*], Wagner Meira Júnior[2], Raquel Cardoso de Melo-Minardi[2]

1 Department of Biochemistry and Immunology, Federal University of Minas Gerais, Belo Horizonte, MG, Brazil
2 Department of Computer Science, Federal University of Minas Gerais, Belo Horizonte, MG, Brazil

*** eblima@dcc.ufmg.br**

S15 Text: Analysis of the correlations among the studied data types

In this Supplementary Text we perform a brief analysis to better understand the redundancy that exists among the data types used as pieces of functional similarity evidence. We calculated the pairwise correlations among data types, presenting such correlations in a heatmap depicted in Figure S15.1.

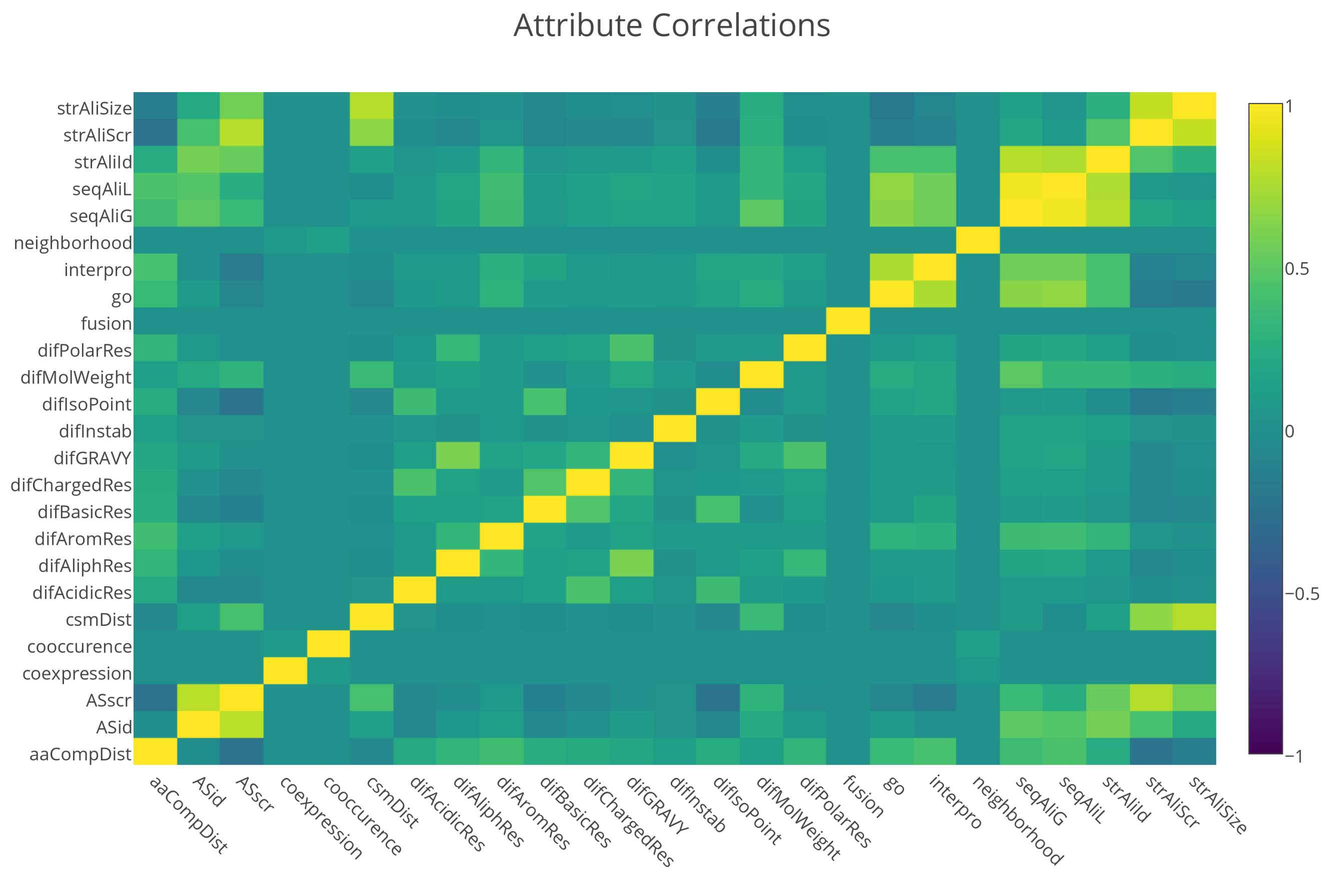

Figure S15.1. Heatmap of the pairwise correlations among data types. The color range represents the [-1, 1] correlation interval.

One may notice a strong correlation between the global and local sequence alignment scores (seqAliG and seqAliL, 0.96 correlation), the structural alignment scores and sizes (strAliScr and strAliSize, 0.82 correlation), and the active site identities and scores (ASid and ASscr, 0.79 correlation). Such correlations were to be expected given the data type pairs originate from the same sources.

Some more interestingly correlated pairs are the active site scores and structural alignment scores (ASscr and strAliScr, 0.78 correlation), the distance among structural signatures and structural alignment scores (csmDist and strAliSize, 0.78 correlation), the global sequence alignment scores and structural alignment identities (seqAliG and strAliId, 0.78 correlation), the local sequence alignment scores and structural alignment identities (seqAliL and strAliId, 0.76 correlation), and the InterPro and GO annotation similarities (interpro and go, 0.75 correlation).

This correlation analysis shows that redundancy exists among some of the data types used as functional similarity evidence. Eliminating redundant data types from the Genetic Programming system might ease the semantic analysis of the obtained data combinations, as well as improve the quality of the generated clusters. Despite the redundancy that exists for some pairs of data types, one may observe most are highly diversified, given they present low correlation to the others, which indicates they add important information to the clustering process.
