# Supplementary material for: Isofunctional Protein Subfamily Detection Using Data Integration and Spectral Clustering
Source: PLoS Comput Biol. 2016 Jun 27;12(6):e1005001. doi: 10.1371/journal.pcbi.1005001 (PMC4922564; doi:10.1371/journal.pcbi.1005001)
Supplement: S1 Table — (PDF) [file pcbi.1005001.s016.pdf]

# Isofunctional Protein Subfamily Detection using Data Integration and Spectral Clustering

Elisa Boari de Lima[1,2,*], Wagner Meira Júnior[2], Raquel Cardoso de Melo-Minardi[2]

**1 Department of Biochemistry and Immunology, Federal University of Minas Gerais, Belo Horizonte, MG, Brazil**
**2 Department of Computer Science, Federal University of Minas Gerais, Belo Horizonte, MG, Brazil**

*** eblima@dcc.ufmg.br**

**Table S1. Enzymatic activity distribution among the seven clusters produced by manually altering ASMC's hierarchical clustering.**

| Nature | Substrate | G1 | G2 | G3 | G4 | G5 | G6 | G7 |
|---|---|---|---|---|---|---|---|---|
| Cationic | (S)-KAH | - | 8 | - | - | 11 | - | - |
| | dehydrocarnitine | 1 | - | 1 | - | 52 | - | - |
| Anionic | β-ketoadipate | - | - | 4 | 25 | - | - | - |
| | β-ketoadipate_R | - | - | - | 18 | - | - | - |
| | β-ketoglutarate | - | - | - | 15 | - | 16 | - |
| | β-ketoglutarate_R | - | - | - | 18 | - | - | - |
| Non-ionic polar | 3,5-dioxohexanoate | 10 | - | 4 | - | - | - | - |
| | 5-hydroxy-β-ketohexanoate | 37 | - | 2 | - | - | - | - |
| | 6-acetamido-β-ketohexanoate | 14 | - | 4 | 8 | 21 | - | - |
| Non-polar | β-ketopentanoate | 1 | 1 | - | - | - | - | - |
| | β-ketopentanoate_R | 37 | - | 4 | - | | - | - |
| | β-ketoisocaproate | 28 | - | 4 | 2 | 13 | - | - |
| | β-ketoisocaproate_R | 24 | - | 2 | - | - | - | - |
| | (E)-β-ketohex-4-enoate_R | 30 | - | 2 | - | - | - | - |
| | β-ketohexanoate | 7 | - | - | - | - | - | - |
| | β-ketohexanoate_R | 53 | - | 8 | 1 | 5 | - | - |
| | 7-methyl-β-ketooct-6-enoate | 6 | - | 2 | 6 | 2 | - | - |
| | β-ketooctanoate_R | 25 | - | 4 | - | 2 | - | - |
| | β-ketododecanoate | 27 | - | 2 | 2 | - | - | - |
| | benzoylacetate_R | - | - | 4 | - | - | - | - |
| | 4-hydroxybenzoylacetate | 2 | 1 | 1 | 2 | 3 | 1 | - |
| | 2-formamidobenzoylacetate | 1 | - | - | 2 | 2 | - | - |

Data published as supplementary material for [1]. _*R*_ denotes tests for reversed reactions.

## References

1. Bastard K, Smith AAT, Vergne-Vaxelaire C, Perret A, Zaparucha A, Melo-Minardi RC, et al. Revealing the hidden functional diversity of an enzyme family. Nat Chem Biol. 2014;10:42–49.
