# Supplementary material for: Isofunctional Protein Subfamily Detection Using Data Integration and Spectral Clustering
Source: PLoS Comput Biol. 2016 Jun 27;12(6):e1005001. doi: 10.1371/journal.pcbi.1005001 (PMC4922564; doi:10.1371/journal.pcbi.1005001)
Supplement: S2 Table — (PDF) [file pcbi.1005001.s017.pdf]

Isofunctional Protein Subfamily Detection using Data Integration and Spectral Clustering

Elisa Boari de Lima[1,2,*], Wagner Meira Júnior[2], Raquel Cardoso de Melo-Minardi[2]

1 Department of Biochemistry and Immunology, Federal University of Minas Gerais, Belo Horizonte, MG, Brazil
2 Department of Computer Science, Federal University of Minas Gerais, Belo Horizonte, MG, Brazil

*** eblima@dcc.ufmg.br**

Table S2. Most important residues for the seven DUF849 clusters produced by the GP system.

Cluster	Residues
**I**	K16, F20, D15, W1, Q2, A19, P12, A14, Q17, G11
**II**	G17, G5, W2, I7, S8, S6, A9, E1
**III**	N21, F18, I14, E6, R2, R17
**IV**	S5, S11, S1, Y13
**V**	A18, N11, P8, I2, S7, Y15, F13
**VI**	G2, C16, D11, M7, I17, T10
**VII**	F13, V17, G1, E2, A10

Listed in decreasing order of partial MI value.
